# Anionic Diels–Alder Chemistry of Cyclic Sodium Dien-1-olates Delivering Highly Stereoselective and Functionalized Polycyclic Adducts

**DOI:** 10.1021/acs.orglett.1c01807

**Published:** 2021-07-21

**Authors:** Jing-Kai Huang, Kak-Shan Shia

**Affiliations:** Institute of Biotechnology and Pharmaceutical Research, National Health Research Institutes, Miaoli County 5, 35053 Taiwan, R.O.C.

## Abstract

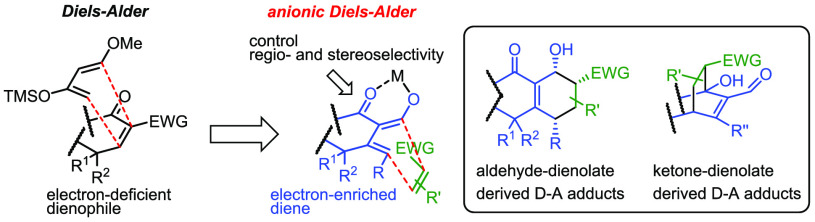

Anionic Diels–Alder chemistry of electron-deficient cross-conjugated vinylogous alkenones, providing highly stable sodium dienolate ion pairs as electron-rich dienes in the presence of a weak sodium base in THF, has been newly developed, leading to a single Diels–Alder adduct, in racemic form, in moderate to high yields (up to 97%, 37 examples).

Diels–Alder cycloaddition reactions remain and continue to be of extreme utility in synthetic organic chemistry particularly in terms of their extraordinary capacity to construct, in one step, fused polycyclic skeletons in a highly regio- and stereoselective manner.^1^ As shown in Figure 1, 2-cyclohexenone (I) and its α-activated analogue (II) have been well studied in their Diels–Alder chemistry as dienophiles, and several vital conclusions can be derived: (1) Diels–Alder cycloaddition of 2-cycloalkenone (I) is a rather poor process; (2) employing Lewis acid as catalyst and/or introducing an additional electron-withdrawing group at its α to ketone position can significantly enhance the dienophilicity of the carbon–carbon double bond; (3) introducing a second double bond into the ring can enhance the secondary effect; (4) C-4 substituent can control the facial selectivity by steric hindrance.^2,3^

**Figure 1:**
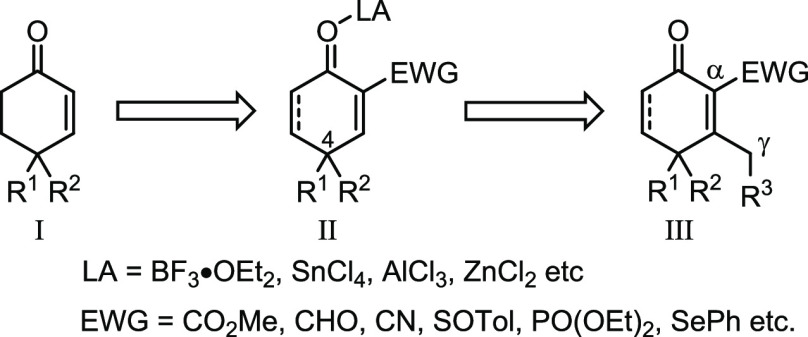
α-Activated 2-cyclohexenones are efficient dienophiles and Michael acceptors.

In our long-lasting interest in Diels–Alder chemistry of 2-cycloalkenones, β-substituted α-activated 2-cyclohexenones (III) are further designed to evaluate whether they are as synthetically useful as their enone counterparts (II). Unfortunately, they have experimentally proved to be rather poor dienophiles for Diels–Alder reactions, most likely because of steric hindrance imposed on the β substituent as indicated by many historic cases bearing a similar structure.^4^ Instead, they are found to be desirable donors for Michael-type [4 + 2] anionic annulation when EWG is an ester group^5^ and excellent dienes for unexpected anionic Diels–Alder reactions when EWG is an aldehyde group (the present work, Figure 2d).

**Figure 2:**
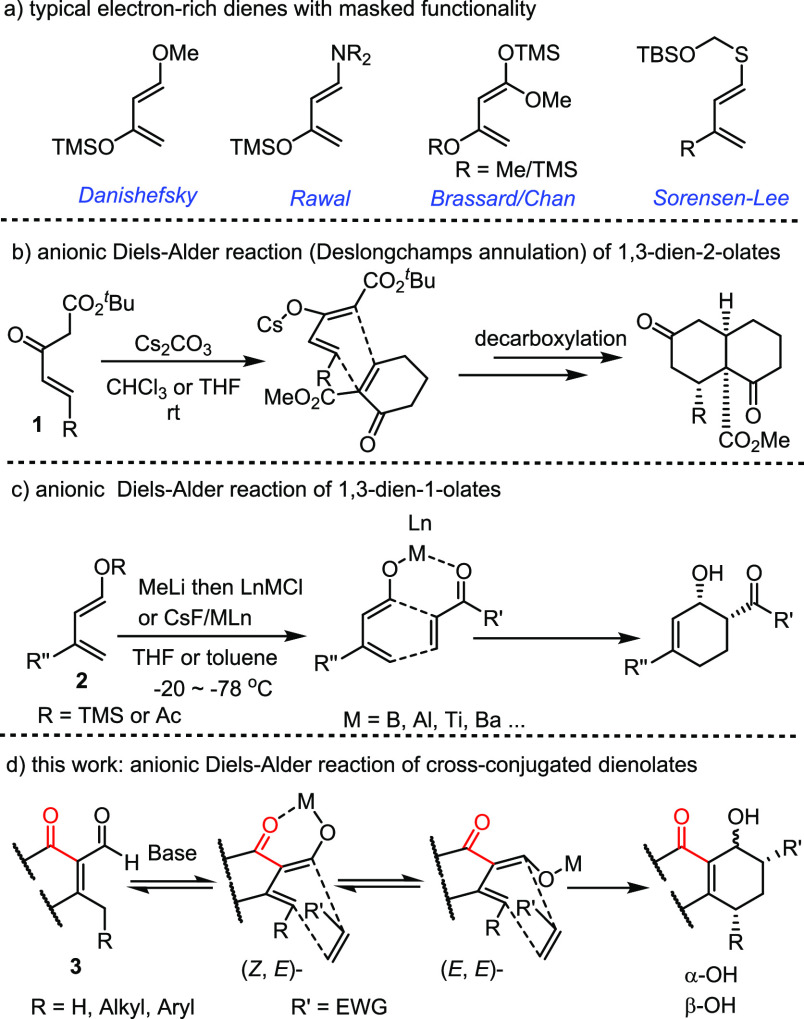
(a) Neutral electron-rich dienes. (b) Nazarov 1,3-dien-2-olates as dienes. (c) 1,3-Dien-1-olates as dienes. (d) 2′-Oxo-1,3-dien-1-olates as dienes.

Regularly, dienes in Diels–Alder chemistry are referred to as 1,3-butadienes, usually installed with an electron-rich functional group(s) as represented by various classical reagents (Figure 2a).^6^ Though some Nazarov reagents, as typified by **1** in Figure 2b, could undergo a base-catalyzed Diels–Alder reaction with a conjugated olefin, mechanistically many turned out to proceed with a tandem double-Michael addition rather than a concerted cycloaddition.^7^ Several dienolate salts of **2** (Figure 2c), generated *in situ* through transmetalation, also have been reported to undergo Diels–Alder reactions effectively, but they must be prepared and operated at low temperature (−78 to −20 °C) because of thermal instability.^8^ Herein, we wish to report that a novel series of dienolate salts (Figure 2d), derived *in situ* from the cross-conjugated vinylogous alkenones **3** with base, are found to be highly thermally stable, allowing reaction with a broad diversity of dienophiles to afford a variety of highly oxygenated Diels–Alder adducts in moderate to high yields. Details of these studies are presented in the following.

According to Scheme 1, using 1,3-dioxin **4** as starting material,^9^ cyclohexenone **7** was readily prepared as a model compound via a three-step synthetic sequence, involving repeated α-methylation, Stork–Danheiser methylation,^10^ and Dess–Martin oxidation,^11^ in an overall yield of *ca*. 60%. Not unexpectedly, different from α-activated 2-cyclohexenones (II) serving as versatile dienophiles, α-aldehyde **7** with a β substituent is a rather poor dienophile for Diels–Alder reactions under either thermal or Lewis acid-catalyzed conditions. Instead, its γ protons can be easily deprotonated and isomerized to form 1,3-dien-1-olates to serve as an electron-rich diene.

**Scheme 1:**
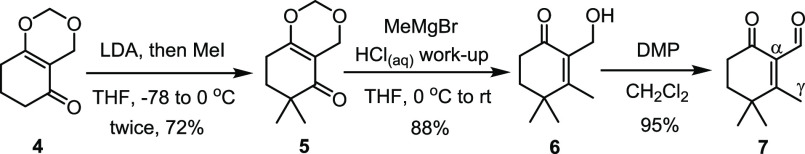
Preparation of Cyclohexenone 7 as a Model Compound

In principle, a cycloaddition product obtained from reacting the 1,3-dien-1-olate of **7** with an electron-deficient olefin **8** can be explained by either a sequential double-Michael addition or a Diels–Alder concerted cycloaddition. Preliminary results of this study are listed in Table 1 and discussed below. As seen in entries 1–3, all tested reactions using a strong, medium or weak lithium base in a less polar solvent THF turned out to be fruitless at either ambient or elevated temperature. To further activate the lithium–enolate ion pair, solvation of lithium cation by HMPA in THF (V/V = 1/4) was then examined;^12^ however, reactions resulted in a complex unidentified mixture as observed on TLC (entry 4). Interestingly, when lithium carbonate was applied in the more polar solvent DMF at higher temperature (entry 5, 100 °C), products **9** and **10** were obtained in 70% and 15%, respectively, of which the relative configuration is unambiguously determined by a single-crystal X-ray analysis.^13^

**Table 1:**
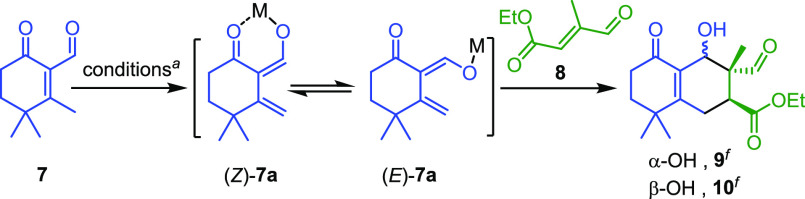
Screening of Optimal Conditions for Anionic [4 + 2] Annulation

entry	reagent (1.2 equiv)	solvent	*T* (°C)/*t*	isolated yield (%) **9**/**10**
1	Li_2_CO_3_	THF	66/48 h	0^b^
2	LiO*^t^*Bu	THF	0–66/24 h	0^b^
3	LiHMDS	THF	0–66/24 h	0^c^
4	LiHMDS	HMPA/THF	0–66/15 h	0^c^
5	Li_2_CO_3_	DMF	100/4 h	70/15
6	NaHCO_3_	THF	66/20 h	89/e
7	Na_2_CO_3_	THF	rt/16 h	91/e
**8**	**Na**_**2**_**CO**_**3**_	**THF**	66/30**min**	**97/**e
9	cat. Na_2_CO_3_^d^	THF	66/20 h	88/e
10	Na_2_CO_3_	MeCN	rt/12 h	88/6
11	Na_2_CO_3_	DMF	rt/1 h	62/30
12	K_2_CO_3_	THF	rt/4 h	85/5
13	Cs_2_CO_3_	CH_2_Cl_2_	rt/30 min	71/10
14	Cs_2_CO_3_	THF	rt/15 min	70/16
15	Cs_2_CO_3_	MeCN	rt/10 min	43/35
16	Cs_2_CO_3_	DMF	rt/<5 min	20/65
17	NEt_3_	CH_2_Cl_2_	rt/48 h	0^b^
18	MgBr_2_·OEt_2_/NEt_3_	CH_2_Cl_2_	rt/15 h	81/trace
19	DBU	THF	rt/30 min	22/30
20	DBU	DMF	rt/<5 min	9/72

a All reactions were performed in solvent (0.2 M) as indicated above under N_2_.

b Reactants **7** and **8** were recovered intact.

c A complex unidentified mixture was observed on TLC.

d 20 mol % of sodium carbonate was used as base.

e Product **10** was not detected in crude ^1^H NMR.

f The relative configuration was unambiguously determined by a single-crystal X-ray analysis.

Encouraged by these results, attention was then paid to using other alkali-metal carbonates. When reactions were tested with sodium base, such as Na_2_CO_3_ and NaHCO_3_, they all proceeded efficiently in THF to afford a single diastereomer **9** in high yields (entries 6 and 7). More importantly, when the reaction was performed in refluxing THF, the reaction rate could be significantly accelerated and completed in *ca*. 30 min (entry 8), affording product **9** in quantitative yield (97%). Thus, the reaction system (Na_2_CO_3_ (1.2 equiv)/THF/66 °C) is tentatively considered to be optimal for this newly developed [4 + 2] annulation process. When the quantity of Na_2_CO_3_ was further reduced to a catalytic amount (20 mol %; entry 9), product **9** was also produced in high yield (88%), but reaction time should be prolonged overnight (20 h), indicating that the annulation process can proceed with a cost-effective catalytic cycle. Also noticed is that as reactions are carried out using Na_2_CO_3_ as base at room temperature (entries 7, 10, and 11), rate acceleration is reflected by the increase of solvent polarity (THF, 16 h; CH_3_CN, 12 h; DMF, 1 h). Interestingly, the formation of **10** is also significantly increased when more polar solvents (CH_3_CN, 6%; DMF, 30%) are employed, which is totally not detected in a less polar solvent (THF, 0%) by the crude ^1^H NMR spectrum.

Similarly, when Cs_2_CO_3_ is used as base at room temperature, product **10** is formed increasingly with the increase of solvent polarity, culminating in a maximal yield of 65% in DMF (entries 13–16). As well, reaction rates are dramatically enhanced and completed within 5–30 min whether in less or more polar solvents. The size of the cation counterion appears to affect both product distribution and reaction rate, as seen in entries 7, 12, and 14. The Hünig base, trimethylamine, is apparently too weak to deprotonate γ acidic protons in CH_2_Cl_2_. As a result, no reaction occurred, and reactants **7** and **8** were recovered intact (entry 17). However, when an extra Lewis acid was added (entry 18), the reaction was triggered and proceeded smoothly to afford **9** in 81% along with a trace of **10**, as detected by the crude ^1^H NMR. In sharp contrast, when a strong base DBU was used, a high selectivity for product **10** over **9** was observed, particularly in a more polar solvent DMF (entry 19 vs 20). According to Table 1, not only was the *trans* isomerism of dienophile **8** constantly preserved in products, but also no Michael-addition intermediates were detected in all cases examined.^14^ To elucidate these outcomes, a plausible mechanism is proposed as follows. Obtaining merely a pair of products **9** and **10** is actually hard to be justified by simply applying an *exo* or *endo* addition rule to a single dienolate (*Z*)-**7a** or (*E*)-**7a** because they are basically in equilibrium (Figure 3). Instead, they are thought to be formed by a concerted addition of dienophile **8** to both dienolates following the *endo* approach A and B, respectively. Because product distribution and reaction rate are highly dependent on the base and solvent used, conformers (*Z*)-**7a** and (*E*)-**7a** are assumed to be interconvertible with a small energy barrier.

**Figure 3:**
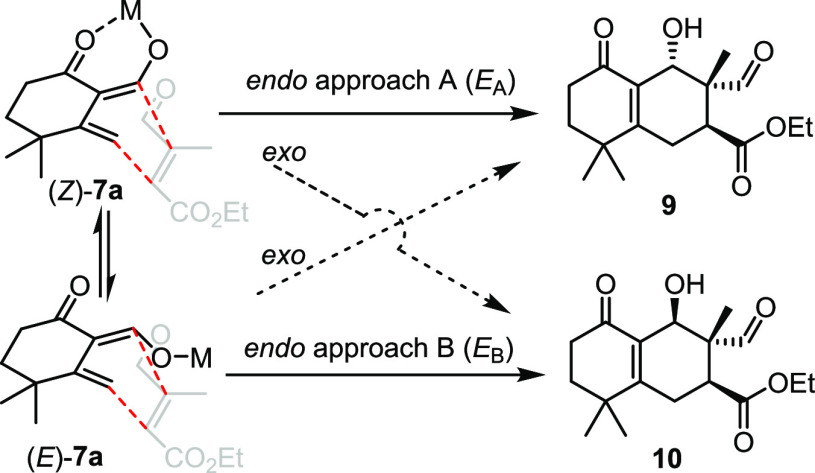
A proposed *endo* approach for Diels–Alder products **9** and/or **10**.

In addition, (*Z*)-**7a** is assigned to have a lower ground-state energy than (*E*)-**7a** because of forming the more stable six-membered ring ion pairs. The reaction-energy profile is conceptually drawn in Figure S1 to interpret their relative relationships along the reaction course. Lithium cation (Li^+^), because of its exceptional oxophilicity,^15^ might reduce electron density on the oxy anion significantly and thus stop enriching dienolates in sufficient electron density from activating the cycloaddition process (entries 1–4). However, this high degree of cation coordination appears to be loosened/disrupted under harsh reaction conditions in a polar solvent (entry 5). Analogous to the anionic oxy-Cope rearrangement,^16^ we believe the negative charge on the oxygen of dienolates should play a crucial role to promote the observed Diels–Alder chemistry. Sodium carbonate in a noncoordinating solvent like THF allows Na^+^ to form a stable chelated bridge with two oxygen atoms of the (Z)-dienolate, leading to product **9** exclusively in high yields. However, the well-coordinating solvent DMF can solvate Na^+^ such that Na^+^ is free and two partially negative charged oxygen atoms tend to be as far apart as possible, as in (*E*)-dienolate. Collectively, it is concluded that anionic Diels–Alder reactions proceed primarily through the *endo* approach A in a less polar solvent with a small countercation Li^+^ or Na^+^ but shift significantly toward the *endo* approach B in a more polar solvent with a bulky countercation K^+^ or Cs^+^. The reactivity trend of base and solvent is found to be in descending order of cation size and polarity, namely, Cs^+^ > K^+^ > Na^+^ > Li^+^ and DMF > CH_3_CN > THF > CH_2_Cl_2_. Thus, a maximum synergistic effect on reaction rate (*ca*. 5 min) was observed when the reaction was carried out in combination with Cs_2_CO_3_ and DMF (entry 16). Lewis acid MgBr_2_ (entry 18) appears to be an effective catalyst to intensify the formation of (*Z*)-**7a** isomer through bidentate chelation, leading to product **9** predominantly. When DBU was applied (entry 20), the reaction rate was dramatically enhanced, suggesting that the conjugate acid DBUH^+^ could behave like a bulky cation Cs^+^ (entry 16) to shift the equilibrium to isomer (*E*)-**7a**. The standard protocol depicted in entry 8 is then employed to explore the scope and limitation of the methodology. Results are listed in Table 2, wherein Diels–Alder adducts highlighted in blue are dienolate parts generated *in situ* from the corresponding α-aldehyde cycloalkenones, including 5–8 membered ring, verbenones, cumarins, and cinnamates, and those parts in green belong to structurally different dienophiles, individually comprising a cyclic maleimide, cumarin, *p*-quinone, acylic α-substituted acrolein, fumarate, or isobutylidene malonate unit. Expected products were commonly obtained in moderate to excellent yields as a single diastereomer, indicating that the methodology is synthetically practicable.

**Table 2:**
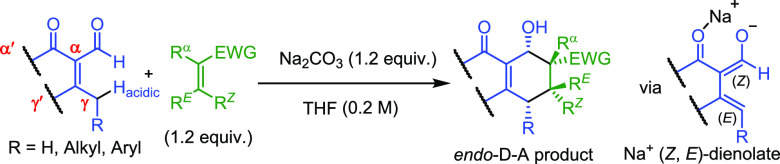

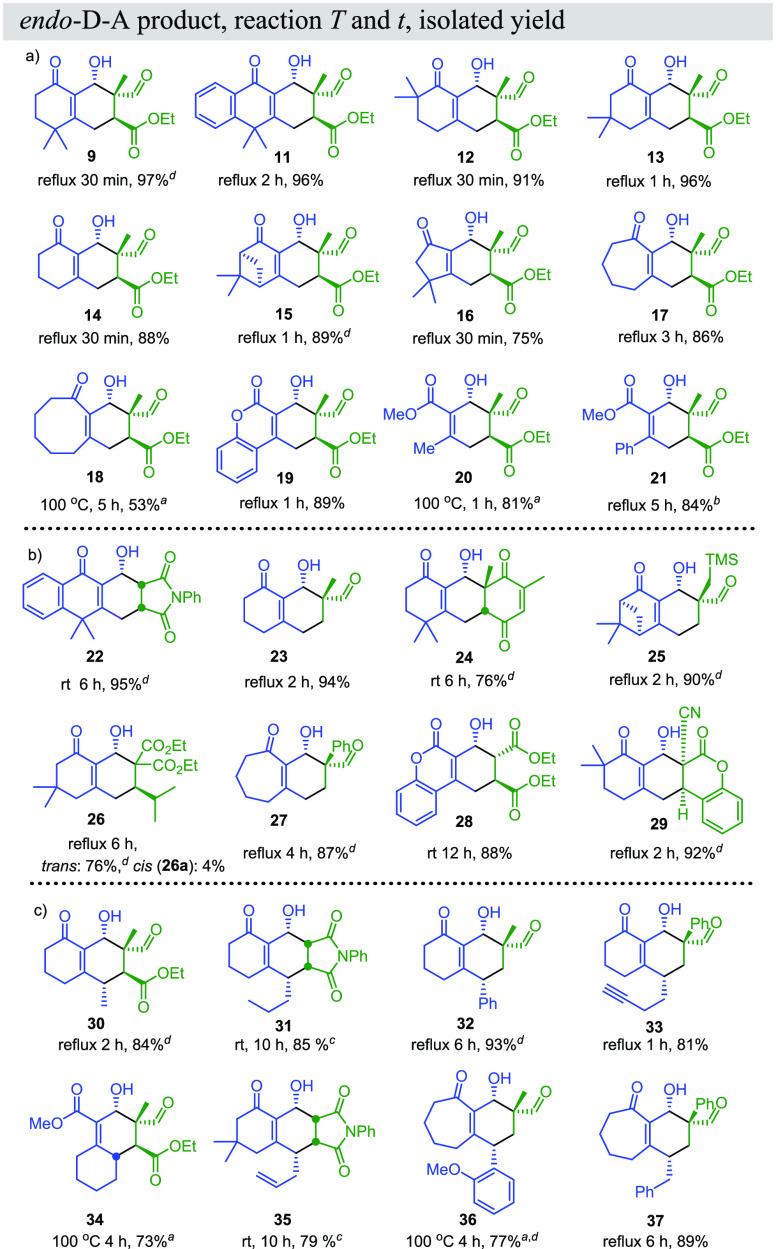
Diels–Alder Adducts Derived from Aldehyde-dienolates

a Reaction was carried out in a sealed tube.

b A mixture of (*E*)- and (*Z*)-cinnamate ester was used.

c Product **31a** (8%) and **35a** (10%) was individually isolated.

d The relative configuration was determined by an X-ray analysis.

More importantly, many are structurally unambiguously identified by X-ray analysis, lending substantial evidence to Diels–Alder chemistry claimed for this novel [4 + 2] annulation. For example, products **15** (89%) and **25** (90%),^13^ formed exclusively in high yields as a single stereoisomer, are considered to be typically governed by the *ortho* and *endo* addition rule with complete face selectivity *via* effectively shielding the gem-dimethyl side of verbenone. Product **30** (84%),^13^ containing four contiguous stereogenic centers precisely predicted by the *ortho* and *endo* rule, also provides strong support for a concerted Diels–Alder approach. Encouragingly, when starting α-aldehyde β-methyl alkenones allow both γ and γ′ sites to undergo deprotonation, the desired aldehyde-dienolate products are still constantly formed in good to excellent yields (77–96%) as seen in **12**–**14**, **17**, **18**, **23**, **26**, **27**, **29**–**33**, and **35**–**37**, with the exception of **18** (53%) in a moderate yield, presumably because of the obstruction of the transannular strain in medium rings. Nevertheless, when products **31** (85%) and **35** (79%) were isolated, the corresponding ketone-dienolate adducts **31a** and **35a** (see the Supporting Information) were also individually identified in 8% and 10% yield, indicating that *cisoid* dienolates through enolization of γ′ protons could also be formed and captured by certain active dienophiles such as *N*-phenylmaleic imide. A single diastereomer **21** (84%) obtained exclusively also supports that a concerted approach should be adopted as both *cisoid* and *transoid* dienolates were generated during the reaction. Indeed, the highly conserved configuration of the dienophile during the transformation into the corresponding product is hard to explain if a two-step Michael–Aldol addition is thought to be a preferred pathway.

To further confirm whether ketone-dienolate D–A adducts are synthetically useful and general, a series of α-aldehyde cycloalkenones, containing only γ′ protons, or α-ester cycloalkenones, containing γ and/or γ′ protons, were designed in order to generate merely the ketone-type *cisoid* dienolate. Results are listed in Table 3. As predicted by a concerted *endo*-addition approach, all products **38**–**46** were obtained with high regio- and stereoselectivity in good to high yields (71∼95%).^17^ Products **38**–**41**, produced at higher temperature (100 °C) than their α-aldehyde counterparts **42**–**46** (66 °C), are somewhat contradictory because dienolates containing a weaker electron-withdrawing ester group should be more reactive in terms of inductive effects. These reverse outcomes might result from the steric hindrance caused by the ester group during cycloaddition.

**Table 3:**
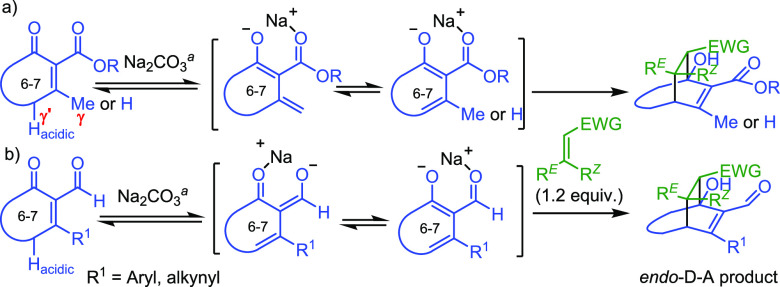

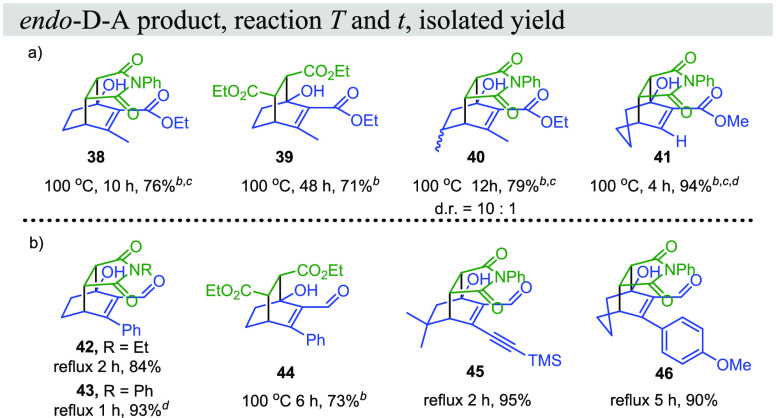
Diels–Alder Adducts Derived from Ketone-dienolates

a Reactions were performed in THF (0.2 M) with Na_2_CO_3_ (1.2 equiv) under N_2_.

b Reaction was carried out in a sealed tube.

c 2.0 equiv of dienophile was used instead.

d The relative configuration was determined by a single-crystal X-ray analysis.

In conclusion, unprecedented anionic Diels–Alder chemistry of highly electron-deficient cross-conjugated vinylogous systems has been newly developed, in which the cyclic sodium dienolate ion pairs, generated *in situ* in the presence of a weak sodium base in THF, are highly thermally stable and operationally simple to play the role of electron-rich dienes during reactions. Products thus obtained contain multiple contiguous chiral centers, whose stereochemical arrangements could be accurately predicted by the *ortho* and *endo* rule, thus strongly supporting a concerted [4 + 2] cycloaddition rather than a consecutive Michael–Aldol type annulation.

## Supporting Information Available

Experimental procedures and spectroscopic data for all new compounds (PDF)

## Accession Codes

CCDC 2007692–2007693, 2074040, 2074048–2074049, 2074053, 2074055, 2074071–2074076, and 2074078 contain the supplementary crystallographic data for this paper. These data can be obtained free of charge via www.ccdc.cam.ac.uk/data_request/cif, or by emailing data_request@ccdc.cam.ac.uk, or by contacting The Cambridge Crystallographic Data Centre, 12 Union Road, Cambridge CB2 1EZ, UK; fax: +44 1223 336033.
