# Supplementary material for: Anionic Diels–Alder Chemistry of Cyclic Sodium Dien-1-olates Delivering Highly Stereoselective and Functionalized Polycyclic Adducts
Source: Org Lett. 2021 Jul 21;23(15):5709–13. doi: 10.1021/acs.orglett.1c01807 (PMC8397436; doi:10.1021/acs.orglett.1c01807)
Supplement: Supplementary file 1 — ol1c01807_si_001.pdf [file ol1c01807_si_001.pdf]

***Supporting Information***

# Anionic Diels-Alder Chemistry of Cyclic Sodium Dien-1-olates Delivering Highly Stereoselective and Functionalized Polycyclic Adducts

Jing-Kai Huang and Kak-Shan Shia*

*Institute of Biotechnology and Pharmaceutical Research, National Health Research Institutes, Miaoli County 35053, Taiwan, R.O.C.*

E-mail: ksshia@nhri.edu.tw

**Table of Contents**

Proposed reaction-energy profiles for dienolates (*Z*)-**7a** and (*E*)-**7a** ion pairs and products **9** and **10** …………………………………………………………………......……….….…..S2

Materials and Methods ………………………………………………….…………..…..S3

Synthetic procedures and characterization of various cross-conjugated enones and precursors ……………………………………………………………………………….S4

Synthetic procedures and characterization of new anionic Diels-Alder products …........…S18

X-ray crystal structure and crystal parameters of anionic Diels-Alder products **9**, **10**, **15**, **22**, **24-27**, **29**, **30**, **32**, **36**, **41**, **43**…………..…………………………….….……………..….S40

References ………………………………………………………………….………....S68

$^{1}$H, $^{13}$C NMR, DEPT, $^{1}$H-$^{1}$H COSY and NOESY spectra for all new compounds ………...S69

2D NOESY spectrum for compound **31a**............................................................................S154

1D NOESY spectra for compound **35a**...............................................................................S172

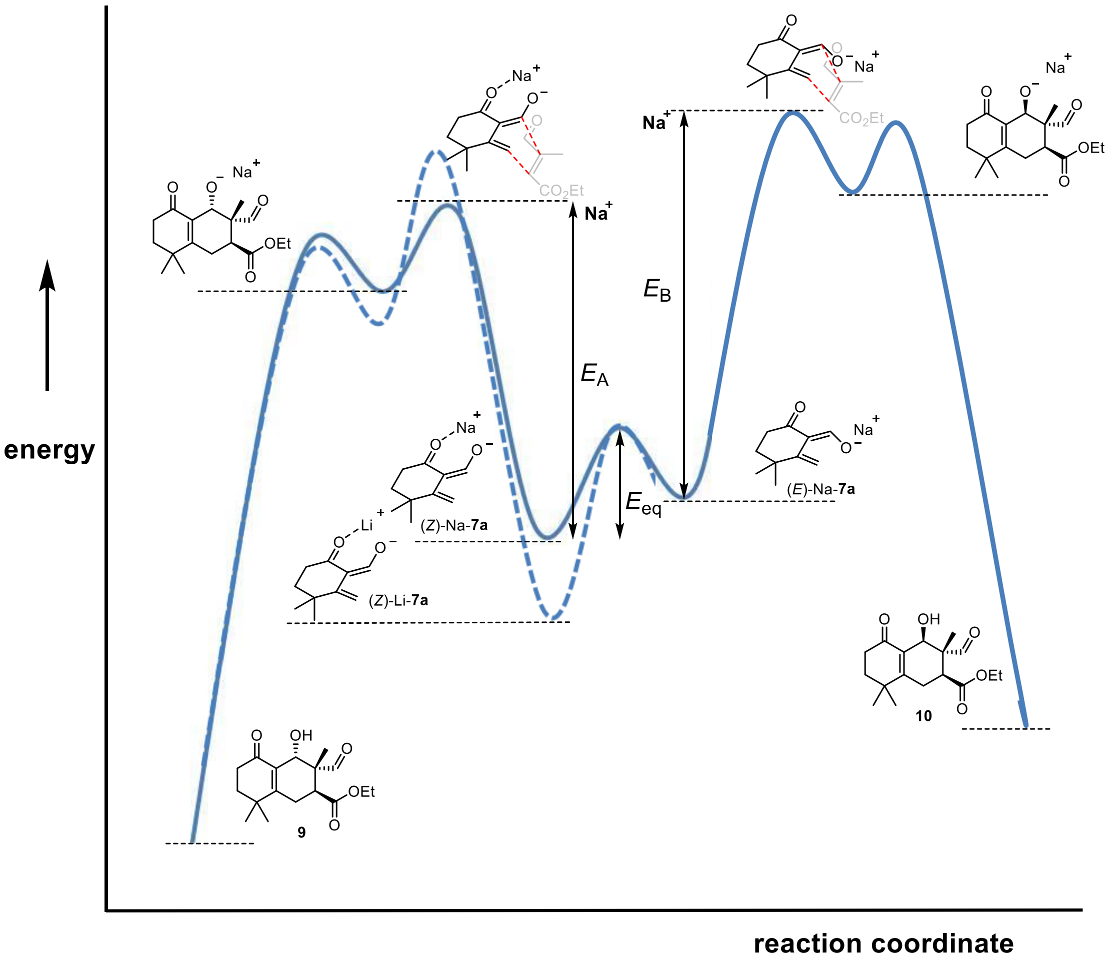


**Figure S1.** Proposed reaction-energy profiles for dienolates (*Z*)-**7a** and (*E*)-**7a** ion pairs and products **9** and **10**. The vertical axis represents the total energy. The transition state is the highest point on the graph, and the activation energy, $E_A$ and $E_B$, is the energy difference between the reactants and the transition state. $E_{eq}$ is the equilibrium energy barrier between conformers (*Z*)-**7a** and (*E*)-**7a**.

**Materials and Methods**

All reactions were performed under nitrogen using flame-dried glassware and stir bars unless otherwise stated. Room temperature refers to 23-26 ℃. A pre-heated oil bath was used to maintain reaction temperature when it was higher than room temperature. All reagents were employed as received without further purification. All solvents were dried and distilled by standard techniques. Tetrahydrofuran was distilled from potassium under $N_2$. Dichloromethane and toluene were distilled from calcium hydride under $N_2$. Analytical thin layer chromatography was performed on $SiO_2$ 60 F-254 plates and flash column chromatography was carried out using $SiO_2$ 60 (particle size 0.040-0.055 mm, 230–400 mesh). Visualization was performed under UV irradiation at 254 nm followed by staining with aqueous potassium permanganate and charring by heat gun. Infrared spectra (IR) were recorded on a FT-IR spectrometer and expressed in $cm^{-1}$. $^{1}H$ and $^{13}C$-NMR spectra were recorded by VNMR-300, VNMR-400, Bruker-400 or Bruker-600. Chemical shifts are expressed in ppm using TMS in $CDCl_3$ ($\delta$ = 0.00) as internal standard in $^{1}H$-NMR spectra. $^{13}C$-NMR spectra were recorded in $CDCl_3$, using the central resonances of $CDCl_3$ ($\delta$ = 77.00) as the internal references. Multiplicities are recorded as s (singlet), d (doublet), t (triplet), q (quartet), quint (quintet), dd (doublet of doublets), dt (doublet of triplets), tt (triplet of triplets), ddd (doublet of doublet of doublets), m (multiplet), br (broad). Coupling constants (*J*) are expressed in Hz. HRMS was obtained on a triple quadrupole mass analysis using electrospray ionization (ESI) source, and spectral data were recorded as m/z values. The single-crystal X-ray diffraction data of crystals were individually collected on a Bruker D8 Venture diffractometer equipped with a Cu-target (Kα =1.54178 Å) or Mo-target (Kα = 0.71073 Å) microfocus X-ray generators and a PHOTON-II CMOS detector. The temperature was adjusted with a nitrogen flow (Oxford Cryosystems). After collection, the data were integrated with the Bruker SAINT software package using a narrow-frame algorithm and were corrected for absorption effects using the Multi-Scan method (SADABS). Then, the molecular structure was solved and refined by the Bruker SHELXTL Software Package and the final anisotropic full-matrix least-squares method was used to refine on F2 with variables parameters to determine crystal structure. Melting points were measured using an Electrothermal instrument.

**Synthetic procedures and characterization of various cross-conjugated enones and their precursors**

General procedure for preparation of 2-(hydroxymethyl)-3-alkyl (aryl or alkynyl) cyclic enones

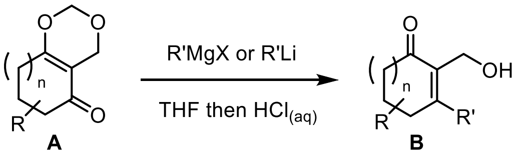


Condition A:

To a stirred solution of 1,3-dioxin **A** (5.0 mmol) in dry THF (15 mL) at 0 °C was added Grignard reagent (7.5 mmol) dropwise under $N_2$. The mixture was then allowed to react at 0 °C to room temperature for 2 h under $N_2$. After the reaction was complete, 5% $HCl_{(aq)}$ (20 mL) was added at 0 °C to acidify the reaction. The reaction mixture then allowed to react at room temperature for 30 min. After the reaction was complete, the organic layer was separated, and the aqueous layer was extracted with EtOAc (30 mL × 2). The organic portions were combined, washed with sat. $NaHCO_{3(aq)}$, dried over $MgSO_4$, filtered and concentrated to give the crude residue, which was purified by chromatography on silical gel to afford the 2-(hydroxymethyl) cyclic enones **B**, including compounds: **6**, **S1-S3**, **S5**, **S9** and **S14-S16** as indicated below.

Condition B:

To a stirred solution of 1,3-dioxin **A** (5.0 mmol) in dry THF (15 mL) at -78 °C was added organic lithium reagent (7.5 mmol) dropwise under $N_2$. The mixture was then allowed to react at -78 to 0 °C for 2 h under $N_2$. After the reaction was complete, 5% $HCl_{(aq)}$ (20 mL) was added at 0 °C to acidify the reaction. The reaction mixture then allowed to react at room temperature for 30 min. After the reaction was complete, the organic layer was separated, and the aqueous layer was extracted with EtOAc (30 mL × 2). The organic portions were combined, washed with sat. $NaHCO_{3(aq)}$, dried over $MgSO_4$, filtered and concentrated to give the crude residue, which was purified by chromatography on silical gel to afford the 2-(hydroxymethyl) cyclic enones **B**, including compounds: **S4**, **S6**, **S8**, **S10**, **S12**, **S17** and **S19** as indicated below.

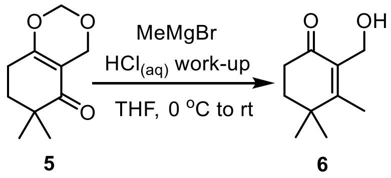


**2-(Hydroxymethyl)-3,4,4-trimethylcyclohex-2-en-1-one (6).** Following the general procedure of condition A, 2-(hydroxymethyl) enone **6** (740 mg, 88% yield) was prepared from 1,3-dioxin **5** (911 mg, 5.0 mmol) and MeMgBr (2.5 mL, 3.0 M in diethyl ether) as a

colorless oil: IR ($CH_2Cl_2$ cast, $cm^{-1}$) $\nu_{max}$ 3432 (br), 2963, 2927, 2869, 1660, 1609; $^1H$ NMR ($CDCl_3$, 400 MHz): $\delta$ 1.18 (s, 6H), 1.84 (t, $J$ = 7.6 Hz, 2H), 1.97 (s, 3H), 2.48 (t, $J$ = 7.6 Hz, 2H), 2.80 (t, $J$ = 6.8 Hz, 1H), 4.35 (d, $J$ = 6.8 Hz, 2H); $^{13}C$ NMR ($CDCl_3$, 150 MHz): $\delta$ 15.5 ($CH_3$), 26.3 ($CH_3$), 34.2 ($CH_2$), 36.1 (C), 36.7 ($CH_2$), 58.0 ($CH_2$), 133.2 (C), 165.3 (C), 200.1 (CO); HRMS (ESI) m/z: $[M + Na]^+$ calcd. for $C_{10}H_{16}O_2Na$ 191.1043, found 191.1035.

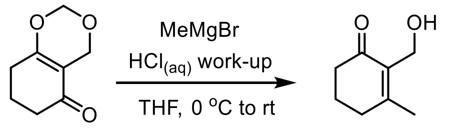


4 S1

**2-(Hydroxymethyl)-3-methylcyclohex-2-en-1-one (S1).** Following the general procedure of condition A, 2-(hydroxymethyl) enone **S1** (568 mg, 81% yield) was prepared from 1,3-dioxin **4** (771 mg, 5.0 mmol) and MeMgBr (2.5 mL, 3.0 M in diethyl ether) as a colorless oil: IR ($CH_2Cl_2$ cast, $cm^{-1}$) $\nu_{max}$ 3420 (br), 2927, 2890, 1654, 1627; $^1H$ NMR ($CDCl_3$, 400 MHz): $\delta$ 1.94-2.00 (m, 2H), 2.03 (s, 3H), 2.39 (t, $J$ = 6.0 Hz, 2H), 2.42 (t, $J$ = 6.4 Hz, 2H), 2.88 (br s, 1H), 4.37 (s, 2H); $^{13}C$ NMR ($CDCl_3$, 150 MHz): $\delta$ 20.8 ($CH_3$), 21.9 ($CH_2$), 32.8 ($CH_2$), 37.5 ($CH_2$), 57.3 ($CH_2$), 134.1 (C), 158.9 (C), 200.6 (CO); HRMS (ESI) m/z: $[M + Na]^+$ calcd. for $C_8H_{12}O_2Na$ 163.0730, found 163.0726.

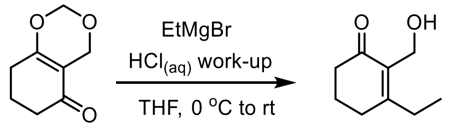


4 S2

**3-Ethyl-2-(hydroxymethyl)cyclohex-2-en-1-one (S2).** Following the general procedure of condition A, 2-(hydroxymethyl) enone **S2** (702 mg, 91% yield) was prepared from 1,3-dioxin **4** (771 mg, 5.0 mmol) and EtMgBr (7.5 mL, 1.0 M in diethyl ether) as a colorless oil: IR ($CH_2Cl_2$ cast, $cm^{-1}$) $\nu_{max}$ 3436 (br), 2965, 2937, 2878, 1656, 1622; $^1H$ NMR ($CDCl_3$, 400 MHz): $\delta$ 1.12 (t, $J$ = 7.6 Hz, 3H), 1.94-2.01 (m, 2H), 2.35 (q, $J$ = 7.6 Hz, 2H), 2.41 (t, $J$ = 6.8 Hz, 2H), 2.42 (t, $J$ = 6.0 Hz, 2H), 2.87 (br s, 1H), 4.36 (s, 2H); $^{13}C$ NMR ($CDCl_3$, 150 MHz): $\delta$ 12.8 ($CH_3$), 22.2 ($CH_2$), 27.7 ($CH_2$), 30.4 ($CH_2$), 37.7 ($CH_2$), 57.0 ($CH_2$), 133.5 (C), 164.3 (C), 201.1 (CO); HRMS (ESI) m/z: $[M + Na]^+$ calcd. for $C_9H_{14}O_2Na$ 177.0886, found 177.0875.

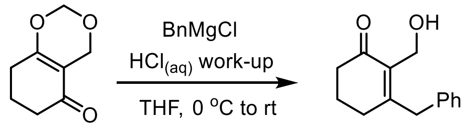


4 S3

**3-Benzyl-2-(hydroxymethyl)cyclohex-2-en-1-one (S3).** Following the general procedure of condition A, 2-(hydroxymethyl) enone **S3** (876 mg, 81% yield) was prepared from 1,3-dioxin **4** (771 mg, 5.0 mmol) and benzylmagnesium chloride (7.5 mL, 1.0 M in diethyl ether) as a white solid: mp = 72–74 °C; IR (KBr, $cm^{-1}$) $\nu_{max}$ 3429 (br), 3084, 3060, 3027, 2940, 2888, 1660, 1624, 1602, 1494, 1453, 1426; $^1$H NMR ($CDCl_3$, 400 MHz): $\delta$ 1.91 (quint, $J$ = 6.4 Hz, 2H), 2.31 (t, $J$ = 6.4 Hz, 2H), 2.43 (t, $J$ = 6.4 Hz, 2H), 2.88 (br s, 1H), 3.69 (s, 2H), 4.51 (s, 2H), 7.17 (d, $J$ = 7.2 Hz, 2H), 7.22-7.33 (m, 3H); $^{13}$C NMR ($CDCl_3$, 100 MHz): $\delta$ 22.0 ($CH_2$), 30.4 ($CH_2$), 37.7 ($CH_2$), 40.1 ($CH_2$), 57.1 ($CH_2$), 126.7 (CH), 128.6 (CH), 128.7 (CH), 134.7 (C), 137.3 (C), 160.1 (C), 200.9 (CO); HRMS (ESI) m/z: $[M + Na]^+$ calcd. for $C_{14}H_{16}O_2Na$ 239.1043, found 239.1040.

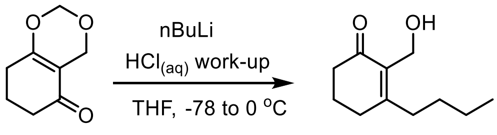


**4** **S4**

**3-Butyl-2-(hydroxymethyl)cyclohex-2-en-1-one (S4).** Following the general procedure of condition B, 2-(hydroxymethyl) enone S**4** (747 mg, 82% yield) was prepared from 1,3-dioxin **4** (771 mg, 5.0 mmol) and *n*-butyllithium (3 mL, 2.5 M in *n*-hexane) as a colorless oil: IR ($CH_2Cl_2$ cast, $cm^{-1}$) $\nu_{max}$ 3436 (br), 2956, 2931, 2871, 1657, 1622; $^1$H NMR ($CDCl_3$, 400 MHz): $\delta$ 0.93 (t, $J$ = 7.2 Hz, 3H), 1.32-1.41 (m, 2H), 1.43-1.51 (m, 2H), 1.93-2.00 (m, 2H), 2.32 (t, $J$ = 7.6 Hz, 2H), 2.40 (t, $J$ = 6.0 Hz, 2H), 2.42 (t, $J$ = 6.8 Hz, 2H), 2.94 (t, $J$ = 6.8 Hz, 1H), 4.35 (d, $J$ = 6.8 Hz, 2H); $^{13}$C NMR ($CDCl_3$, 150 MHz): $\delta$ 13.8 ($CH_3$), 22.2 ($CH_2$), 22.8 ($CH_2$), 30.5 ($CH_2$), 30.9 ($CH_2$), 34.4 ($CH_2$), 37.7 ($CH_2$), 57.1 ($CH_2$), 133.9 (C), 163.0 (C), 201.1 (CO); HRMS (ESI) m/z: $[M + Na]^+$ calcd. for $C_{11}H_{18}O_2Na$ 205.1199, found 205.1187.

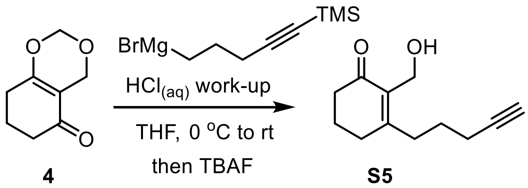


**4** **S5**

**2-(Hydroxymethyl)-3-(pent-4-yn-1-yl)cyclohex-2-en-1-one (S5).** Following the general procedure of condition A, 2-(hydroxymethyl) enone **S5** (682 mg, 71% yield over 2 steps) was prepared from 1,3-dioxin **4** (771 mg, 5.0 mmol) and (5-(trimethylsilyl)pent-4-yn-1-yl) magnesium bromide (7.5 mL, 1 M in THF) and TBAF (10 mL, 1.0 M in THF) as a light yellow oil: IR ($CH_2Cl_2$ cast, $cm^{-1}$) $\nu_{max}$ 3436 (br), 3290, 2937, 2870, 2215, 1656, 1622; $^1$H NMR ($CDCl_3$, 400 MHz): $\delta$ 1.68-1.76 (m, 2H), 1.98 (quint, $J$ = 6.0 Hz, 2H), 2.02 (t, $J$ = 2.8 Hz, 1H), 2.25 (td, $J$ = 6.8, 2.8 Hz, 2H), 2.40-2.48 (m, 6H), 2.82 (t, $J$ = 6.8 Hz, 1H), 4.37 (d, $J$ = 6.8 Hz, 2H); $^{13}$C NMR ($CDCl_3$, 100 MHz): $\delta$ 18.3 ($CH_2$), 22.1 ($CH_2$), 27.0 ($CH_2$), 30.8

($CH_2$), 33.4 ($CH_2$), 37.6 ($CH_2$), 56.9 ($CH_2$), 69.4 (C), 83.2 (CH), 134.6 (C), 161.5 (C), 200.8 (CO); HRMS (ESI) m/z: $[M + Na]^+$ calcd. for $C_{12}H_{16}O_2Na$ 215.1043, found 215.1047.

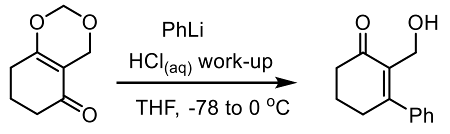


**4** **S6**

**2-(Hydroxymethyl)-5,6-dihydro-[1,1'-biphenyl]-3(4*H*)-one (S6).** Following the general procedure of condition B, 2-(hydroxymethyl) enone S**6** (849 mg, 84% yield) was prepared from 1,3-dioxin **4** (771 mg, 5.0 mmol) and phenyllithium (7.5 mL, 1.0 M in THF) as a white solid: mp = 86–88 °C; IR (KBr, $cm^{-1}$) $\nu_{max}$ 3450 (br), 3057, 2921, 2850, 1660, 1614, 1572, 1424, 1361; $^1H$ NMR ($CDCl_3$, 400 MHz): $\delta$ 2.10-2.17 (m, 2H), 2.55 (t, $J$ = 7.2 Hz, 2H), 2.71 (t, $J$ = 6.0 Hz, 2H), 2.92 (t, $J$ = 7.2 Hz, 1H), 4.21 (d, $J$ = 7.2 Hz, 2H), 7.27-7.31 (m, 2H), 7.35-7.44 (m, 3H); $^{13}C$ NMR ($CDCl_3$, 150 MHz): $\delta$ 22.4 ($CH_2$), 32.7 ($CH_2$), 37.7 ($CH_2$), 58.7 ($CH_2$), 127.3 (CH), 128.5 (CH), 128.8 (CH), 134.5 (C), 139.4 (C), 159.8 (C), 201.5 (CO); HRMS (ESI) m/z: $[M + Na]^+$ calcd. for $C_{13}H_{14}O_2Na$ 225.0886, found 225.0888.

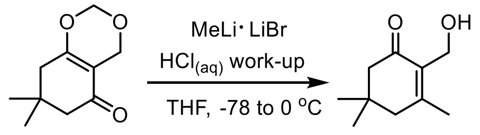


**S7** **S8**

**2-(Hydroxymethyl)-3,5,5-trimethylcyclohex-2-en-1-one (S8).** Following the general procedure of condition B, 2-(hydroxymethyl) enone **S8** (732 mg, 87% yield) was prepared from 1,3-dioxin **S7** (911 mg, 5.0 mmol) and MeLi·LiBr (7.5 mL, 1.0 M in diethyl ether) as a white solid: mp = 51–52 °C; IR ($CH_2Cl_2$ cast, $cm^{-1}$) $\nu_{max}$ 3420, 2957, 2893, 2887, 1661, 1635; $^1H$ NMR ($CDCl_3$, 400 MHz): $\delta$ 1.03 (s, 6H), 1.61 (s, 2H), 2.00 (s, 2H), 2.28 (s, 3H), 2.79 (t, $J$ = 6.0 Hz, 1H), 4.38 (d, $J$ = 6.0 Hz, 2H); $^{13}C$ NMR ($CDCl_3$, 150 MHz): $\delta$ 20.9 ($CH_3$), 28.2 ($CH_3$), 32.7 (C), 46.9 ($CH_2$), 51.1 ($CH_2$), 57.0 ($CH_2$), 132.9 (C), 156.3 (C), 200.8 (CO); HRMS (ESI) m/z: $[M + Na]^+$ calcd. for $C_{10}H_{16}O_2Na$ 191.1043, found 191.1043.

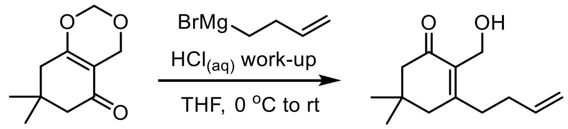


**S7** **S9**

**3-(But-3-en-1-yl)-2-(hydroxymethyl)-5,5-dimethylcyclohex-2-en-1-one (S9).** Following the general procedure of condition A, 2-(hydroxymethyl) enone **S9** (781 mg, 75% yield) was prepared from 1,3-dioxin **S7** (911 mg, 5.0 mmol) and but-3-en-1-ylmagnesium bromide (7.5 mL, 1.0 M in THF) as a colorless oil: IR ($CH_2Cl_2$ cast, $cm^{-1}$) $\nu_{max}$ 3445 (br), 3078, 2957, 2891,

2869, 1662, 1628, 1412, 1368; $^1$H NMR ($CDCl_3$, 400 MHz): $\delta$ 1.03 (s, 6H), 2.21-2.27 (m, 2H), 2.28 (s, 2H), 2.29 (s, 2H), 2.40 (t, $J$ = 7.6 Hz, 2H), 2.77 (t, $J$ = 6.8 Hz, 1H), 4.36 (d, $J$ = 6.8 Hz, 2H), 5.02 (dd, $J$ = 7.6, 0.8 Hz, 1H), 5.06 (dd, $J$ = 17.6, 1.6 Hz, 1H), 5.79 (ddt, $J$ = 17.6, 7.6, 6.0 Hz, 1H); $^{13}$C NMR ($CDCl_3$, 100 MHz): $\delta$ 28.1 ($CH_3$), 31.9 ($CH_2$), 32.9 (C), 33.8 ($CH_2$), 44.9 ($CH_2$), 51.1 ($CH_2$), 56.8 ($CH_2$), 115.8 ($CH_2$), 133.3 (C), 136.7 (CH), 159.0 (C), 201.1 (CO); HRMS (ESI) m/z: $[M + Na]^+$ calcd. for $C_{13}H_{20}O_2Na$ 231.1356, found 231.1359.

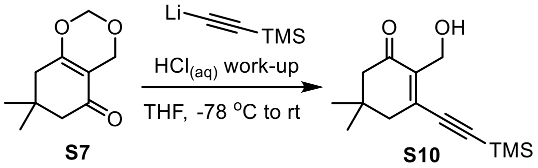


**2-(Hydroxymethyl)-5,5-dimethyl-3-((trimethylsilyl)ethynyl)cyclohex-2-en-1-one (S10).** Following the general procedure of condition A, 2-(hydroxymethyl) enone **S10** (776 mg, 62% yield) was prepared from 1,3-dioxin **S7** (911 mg, 5.0 mmol) and Lithium (trimethylsilyl)acetylide (15 mL, 0.5 M in THF) as a light yellow oil: IR ($CH_2Cl_2$ cast, $cm^{-1}$) $\nu_{max}$ 3469 (br), 2959, 2898, 2871, 2140, 1663, 1598, 1412, 1389, 1359; $^1$H NMR ($CDCl_3$, 400 MHz): $\delta$ 0.23 (s, 9H), 1.06 (s, 6H), 2.32 (s, 2H), 2.41 (s, 2H), 2.92 (t, $J$ = 7.2 Hz, 1H), 4.54 (d, $J$ = 7.2 Hz, 2H); $^{13}$C NMR ($CDCl_3$, 150 MHz): $\delta$ 0.42 ($CH_3$), 27.9 ($CH_3$), 33.4 (C), 44.7 ($CH_2$), 51.5 ($CH_2$), 60.0 ($CH_2$), 101.7 (C), 110.9 (C), 137.2 (C), 139.8 (C), 200.4 (CO); HRMS (ESI) m/z: $[M + Na]^+$ calcd. for $C_{14}H_{22}O_2SiNa$ 273.1281, found 273.1272.

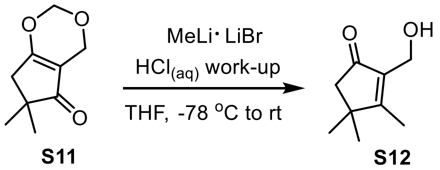


**2-(Hydroxymethyl)-3,4,4-trimethylcyclopent-2-en-1-one (S12).** Following the general procedure of condition B, 2-(hydroxymethyl) enone **S12** (663 mg, 86% yield) was prepared from 1,3-dioxin **S11** (841 mg, 5.0 mmol) and MeLi·LiBr ( 7.5 mL, 1.0 M in diethyl ether) as a colorless oil: IR ($CH_2Cl_2$ cast, $cm^{-1}$) $\nu_{max}$ 3412 (br), 2962, 2928, 2871, 1693, 1642; $^1$H NMR ($CDCl_3$, 400 MHz): $\delta$ 1.22 (s, 6H), 2.01 (s, 3H), 2.32 (s, 2H), 2.87 (br s, 1H), 4.33 (s, 2H); $^{13}$C NMR ($CDCl_3$, 150 MHz): $\delta$ 11.6 ($CH_3$), 26.6 ($CH_3$), 41.7 (C), 50.7 ($CH_2$), 56.0 ($CH_2$), 136.7 (C), 179.4 (C), 208.7 (CO); HRMS (ESI) m/z: $[M + Na]^+$ calcd. for $C_9H_{14}O_2Na$ 177.0886, found 177.1882.

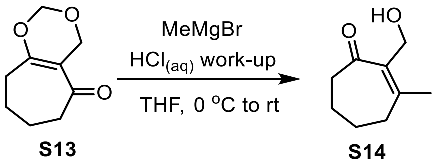

**2-(Hydroxymethyl)-3-methylcyclohept-2-en-1-one (S14).** Following the general procedure of condition A, 2-(hydroxymethyl) enone **S14** (709 mg, 92% yield) was prepared from 1,3-dioxin **S13** (841 mg, 5.0 mmol) and MeMgBr (2.5 mL, 3.0 M in diethyl ether) as a colorless oil: IR ($CH_2Cl_2$ cast, $cm^{-1}$) $\nu_{max}$ 3424 (br), 2939, 2867, 1712, 1655, 1458, 1424, 1375; $^1H$ NMR ($CDCl_3$, 400 MHz): $\delta$ 1.72-1.80 (m, 4H), 2.06 (s, 3H), 2.44 (dd, $J$ = 6.4, 4.4 Hz, 2H), 2.58 (dd, $J$ = 6.8, 6.4 Hz, 2H), 2.81 (br s, 1H), 4.31 (s, 2H); $^{13}C$ NMR ($CDCl_3$, 150 MHz): $\delta$ 20.8 ($CH_2$), 23.0 ($CH_3$), 23.7 ($CH_2$), 34.7 ($CH_2$), 41.8 ($CH_2$), 59.8 ($CH_2$), 137.1 (C), 155.1 (C), 207.5 (CO); HRMS (ESI) m/z: $[M + Na]^+$ calcd. for $C_9H_{14}O_2Na$ 177.0886, found 177.0882.

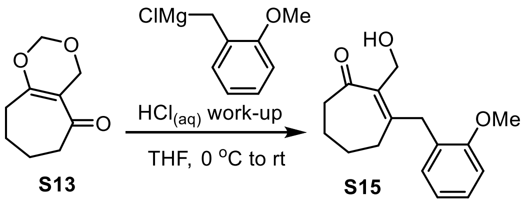


**2-(Hydroxymethyl)-3-(2-methoxybenzyl)cyclohept-2-en-1-one (S15).** Following the general procedure of condition A, 2-(hydroxymethyl) enone **S15** (729 mg, 56% yield) was prepared from 1,3-dioxin **S13** (841 mg, 5.0 mmol) and 2-methoxybenzylmagnesium chloride (20 mL, 0.25 M in THF) as a light yellow oil: IR ($CH_2Cl_2$ cast, $cm^{-1}$) $\nu_{max}$ 3447 (br), 3060, 2937, 2864, 1654, 1599, 1556, 1492, 1458, 1439; $^1H$ NMR ($CDCl_3$, 400 MHz): $\delta$ 1.44-1.51 (m, 2H), 1.68-1.75 (m, 2H), 2.34 (t, $J$ = 6.4 Hz, 2H), 2.55 (t, $J$ = 6.4 Hz, 2H), 2.81 (br s, 1H), 3.68 (s, 2H), 3.83 (s, 3H), 4.46 (s, 2H), 6.87 (dd, $J$ = 8.2, 0.8 Hz, 1H), 6.91 (td, $J$ = 7.4, 1.2 Hz, 1H), 7.09 (dd, $J$ = 7.4, 1.6 Hz, 1H), 7.24 (td, $J$ = 8.2, 1.6 Hz, 1H); $^{13}C$ NMR ($CDCl_3$, 150 MHz): $\delta$ 20.7 ($CH_2$), 24.1 ($CH_2$), 31.7 ($CH_2$), 36.0 ($CH_2$), 41.7 ($CH_2$), 55.3 ($CH_3$), 59.7 ($CH_2$), 110.4 (CH), 120.7 (CH), 125.8 (C), 128.0 (CH), 130.3 (CH), 138.0 (C), 154.7 (C), 157.6 (C), 208.4 (CO); HRMS (ESI) m/z: $[M + Na]^+$ calcd. for $C_{16}H_{20}O_3Na$ 283.1305, found 283.1308.

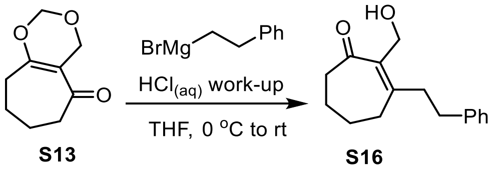


**2-(Hydroxymethyl)-3-phenethylcyclohept-2-en-1-one (S16).** Following the general procedure of condition A, 2-(hydroxymethyl) enone **S16** (941 mg, 77% yield) was prepared from 1,3-dioxin **S13** (841 mg, 5.0 mmol) and (7.5 mL, 1.0 M in THF) as a colorless oil: IR ($CH_2Cl_2$ cast, $cm^{-1}$) $\nu_{max}$ 3435 (br), 3084, 3061, 3026, 2937, 2865, 1654, 1496, 1454; $^1H$ NMR ($CDCl_3$, 400 MHz): $\delta$ 1.70-1.81 (m, 4H), 2.45 (dd, $J$ = 6.8, 4.4 Hz, 2H), 2.52 (t, $J$ = 6.4Hz, 1H), 2.56 (dd, $J$ = 6.8, 4.8 Hz, 2H), 2.63 (dd, $J$ = 8.2, 7.2 Hz, 2H), 2.80 (dd, $J$ = 8.2, 7.6 Hz, 2H), 4.16 (d, $J$ = 6.4 Hz, 2H), 7.17-7.24 (m, 3H), 7.28-7.32 (m, 2H); $^{13}C$ NMR ($CDCl_3$, 100

MHz): $\delta$ 20.7 ($CH_2$), 24.2 ($CH_2$), 33.0 ($CH_2$), 34.6 ($CH_2$), 38.8 ($CH_2$), 41.7 ($CH_2$), 59.5 ($CH_2$), 126.3 (CH), 128.3 (CH), 128.5 (CH), 137.9 (C), 140.6 (C), 156.5 (C), 207.9 (CO); HRMS (ESI) m/z: $[M + Na]^+$ calcd. for $C_{16}H_{20}O_2Na$ 267.1356, found 267.1356.

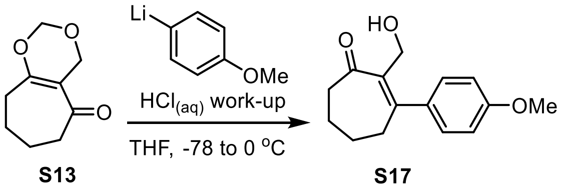


**2-(Hydroxymethyl)-3-(4-methoxyphenyl)cyclohept-2-en-1-one (S17).** Following the general procedure of condition B, 2-(hydroxymethyl) enone **S17** (985 mg, 80% yield) was prepared from 1,3-dioxin **S13** (841 mg, 5.0 mmol) and 4-methoxyphenyllithium (7.5 mL, 1.0 M in THF) as a light yellow oil: IR ($CH_2Cl_2$ cast, $cm^{-1}$) $\nu_{max}$ 3446 (br), 3062, 2936, 2864, 1654, 1606, 1570, 1509, 1458; $^1H$ NMR ($CDCl_3$, 400 MHz): $\delta$ 1.84-1.94 (m, 4H), 2.68 (t, $J$ = 6.0 Hz, 2H), 2.75 (t, $J$ = 6.0 Hz, 2H), 2.86 (td, $J$ = 6.4, 4.0, 1H), 3.83 (s, 3H), 4.15 (d, $J$ = 6.4 Hz, 2H), 6.91 (d, $J$ = 8.8 Hz, 2H), 7.22 (d, $J$ = 8.8 Hz, 2H); $^{13}C$ NMR ($CDCl_3$, 100 MHz): $\delta$ 20.8 ($CH_2$), 24.2 ($CH_2$), 34.8 ($CH_2$), 41.8 ($CH_2$), 55.2 ($CH_3$), 61.3 ($CH_2$), 113.6 (CH), 128.9 (CH), 134.2 (C), 137.3 (C), 156.0 (C), 159.5 (C), 208.4 (CO); HRMS (ESI) m/z: $[M + Na]^+$ calcd. for $C_{15}H_{18}O_3Na$ 269.1148, found 269.1146.

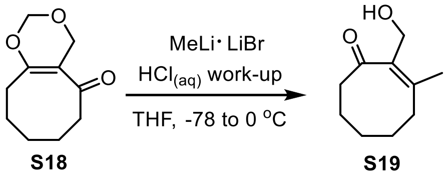


**(*Z*)-2-(Hydroxymethyl)-3-methylcyclooct-2-en-1-one (S19).** Following the general procedure of condition B, 2-(hydroxymethyl) enone **S19** (740 mg, 88% yield) was prepared from 1,3-dioxin **S18** (911 mg, 5.0 mmol) and MeLi·LiBr (7.5 mL, 1.0 M in diethyl ether) as a colorless oil: IR ($CH_2Cl_2$ cast, $cm^{-1}$) $\nu_{max}$ 3429 (br), 2930, 2870, 1677, 1454, 1420, 1407; $^1H$ NMR ($CDCl_3$, 600 MHz): $\delta$ 1.57-1.61 (m, 2H), 1.63-1.67 (m, 2H), 1.80-1.84 (m, 2H), 1.92 (s, 3H), 2.38 (t, $J$ = 6.0 Hz, 2H), 2.41 (t, $J$ = 4.2 Hz, 1H), 2.65 (t, $J$ = 6.6 Hz, 2H), 4.29 (d, $J$ = 4.2 Hz, 2H); $^{13}C$ NMR ($CDCl_3$, 150 MHz): $\delta$ 21.2 ($CH_3$), 22.5 ($CH_2$), 23.4 ($CH_2$), 26.8 ($CH_2$), 35.9 ($CH_2$), 45.1 ($CH_2$), 60.9 ($CH_2$), 135.3 (C), 144.9 (C), 211.5 (CO); HRMS (ESI) m/z: $[M + Na]^+$ calcd. for $C_{10}H_{16}O_2Na$ 191.1043, found 191.1037.

The following known precursors were prepared by reported procedures: compounds **4**,[1] **5**,[2] **S7**,[3] **S13**,[1] **S20**,[4] **S26**,[5] **S28**,[6] **S36**,[7] **S37**[8] and **S38**.[9] The remaining precursors **7**, **S18**, **S22**, **S25**, **S27**, **S29**, **S31**, **S33** and **S35** were prepared by the following procedures.

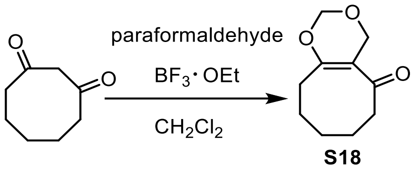


**4,6,7,8,9,10-Hexahydro-5*H*-cycloocta[*d*][1,3]dioxin-5-one (S18).**[1] To a stirred solution of cycloctane-1,3-dione (2.80 g, 20.0 mmol) and paraformaldehyde (3.03 g, 100.0 mmol) in dry $CH_2Cl_2$ (200 mL) at room temperature was added $BF_3$·$OEt_2$ (6.28 mL, 50.0 mmol) under $N_2$. The mixture was then allowed to react at room temperature for 3 h under $N_2$. After the reaction was complete, sat. $NaHCO_{3(aq)}$ (200 mL) was added to quench the reaction at 0 °C, and the resulting mixture was filtrated with celite. The organic portions were washed with sat. $NaHCO_{3(aq)}$ and brine, dried over $MgSO_4$, filtered and concentrated to give the crude residue, which was purified by chromatography on silical gel to afford the 1,3-dioxin **S18** (2.48 g, 68% yield) as a colorless oil: IR ($CH_2Cl_2$ cast, $cm^{-1}$) $\nu_{max}$ 2929, 2859, 2795, 1613, 1479, 1411, 1385; $^1H$ NMR ($CDCl_3$, 400 MHz): $\delta$ 1.57-1.63 (m, 2H),7.72 (quint, $J$ = 6.8 Hz, 2H), 1.74 (quint, $J$ = 7.2 Hz, 2H), 2.79 (t, $J$ = 7.2 Hz, 2H), 2.81 (t, $J$ = 6.8 Hz, 2H), 4.48 (s, 2H), 5.07 (s, 2H); $^{13}C$ NMR ($CDCl_3$, 150 MHz): $\delta$ 23.1 ($CH_2$), 23.2 ($CH_2$), 23.5 ($CH_2$), 32.9 ($CH_2$), 41.9 ($CH_2$), 64.4 ($CH_2$), 91.0 ($CH_2$), 116.6 (C), 166.2 (C), 198.7 (CO); HRMS (ESI) m/z: [M + Na]$^+$ calcd. for $C_{10}H_{14}O_3Na$ 205.0835, found 205.0834.

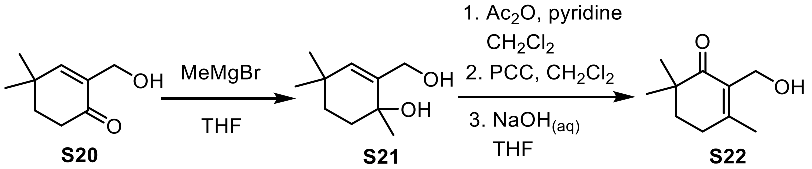


**2-(Hydroxymethyl)-1,4,4-trimethylcyclohex-2-en-1-ol (S21).**[10] To a stirred solution of 2-(hydroxymethyl) enone **S20** (1.54 g, 10.0 mmol) in dry THF (50 mL) at 0 °C was added MeMgBr solution (8 mL, 3.0 M in $Et_2O$, xx mmol) dropwise under $N_2$. The mixture was then allowed to react at 0 °C to room temperature for 1 h under $N_2$. After the reaction was complete, 5% $HCl_{(aq)}$ (20 mL) was added to quench the reaction at 0 °C, and the resulting mixture was extracted with EtOAc (50 mL × 2). The organic portions were combined, washed with sat. $NaHCO_{3(aq)}$ and brine, dried over $MgSO_4$, filtered and concentrated to give the crude residue, which was recrystallized from EtOAc/*n*-hexane to afford diol **S21** (1.41 g, 83% yield) as a white solid: mp = 124–126 °C; IR (KBr, $cm^{-1}$) $\nu_{max}$ 3254 (br), 2960, 2933, 2920, 2852, 1458, 1359; $^1H$ NMR ($CDCl_3$, 400 MHz): $\delta$ 0.95 (s, 3H), 1.05 (s, 3H), 1.40 (s, 3H), 1.41-1.48 (m, 1H), 1.58-1.65 (m, 1H), 1.71-1.83 (m, 2H), 2.41 (br s, 2H), 3.99 (d, $J$ = 12.0 Hz, 1H), 4.46 (d,

$J$ = 12.0 Hz, 1H), 5.43 (s, 1H); $^{13}C$ NMR ($CDCl_3$, 150 MHz): $\delta$ 27.5 ($CH_3$), 28.5 ($CH_3$), 29.5 ($CH_3$), 32.2 (C), 33.8 ($CH_2$), 36.4 ($CH_2$), 65.5 ($CH_2$), 70.9 (C), 137.6 (C), 138.1 (CH); HRMS (ESI) m/z: $[M + Na]^+$ calcd. for $C_{10}H_{18}O_2Na$ 193.1199, found 193.1196.

**2-(Hydroxymethyl)-3,6,6-trimethylcyclohex-2-en-1-one (S22).**[10] To a stirred solution of the diol **S21** (851 mg, 5.0 mmol) and pyridine (4 mL) in dry $CH_2Cl_2$ (6 mL) was added $Ac_2O$ (613 mg, 6.0 mmol) in one portion at 0 °C. The resulting mixture was then stirred at 40 °C under $N_2$ for 3 h. After reaction was complete, the reaction mixture was quenched with 5% $HCl_{(aq)}$ (20 mL) and extracted with $CH_2Cl_2$ (20 mL × 2). The combined organic extract was washed with saturated $NaHCO_{3(aq)}$ and brine, dried over $MgSO_4$, filtered and concentrated to give the crude acetyl intermediate. To a stirred solution of the acetyl intermediate and celite (3 g) in dry $CH_2Cl_2$ (30 mL) was added PCC (1.62 g, 7.5 mmol) in one portion at 0 °C. The resulting mixture was then stirred at room temperature under $N_2$ for 6 h. After reaction was complete, the reaction mixture was filtered and concentrated to give the crude enone acetate intermediate. To a stirred solution of the crude enone acetate intermediate in THF (20 mL) was added 4N $NaOH_{(aq)}$ (5 mL, 20.0 mmol) in one portion at 0 °C. The resulting mixture was then stirred at room temperature under $N_2$ for 10 h. After reaction was complete, the reaction mixture was extracted with EtOAc (20 mL × 3). The organic portions were combined, washed with water and brine, dried over $MgSO_4$, filtered and concentrated to give the crude residue, which was purified by chromatography on silical gel to afford the 2-(hydroxymethyl) enone **S22** (639 mg, 76% yield over 3 steps) as a colorless oil: IR ($CH_2Cl_2$ cast, $cm^{-1}$) $\nu_{max}$ 3448 (br), 2963, 2922, 2869, 1660, 1636, 1472, 1452, 1425, 1385; $^1H$ NMR ($CDCl_3$, 400 MHz): $\delta$ 1.11 (s, 6H), 1.80 (t, $J$ = 6.2 Hz, 2H), 2.00 (s, 3H), 2.39 (t, $J$ = 6.2 Hz, 2H), 2.84 (br s, 1H), 4.34 (s, 2H); $^{13}C$ NMR ($CDCl_3$, 100 MHz): $\delta$ 20.6 ($CH_3$), 24.2 ($CH_3$), 29.6 ($CH_2$), 35.3 ($CH_2$), 40.1 (C), 57.5 ($CH_2$), 132.2 (C), 156.6 (C), 205.4 (CO); HRMS (ESI) m/z: $[M + Na]^+$ calcd. for $C_{10}H_{16}O_2Na$ 191.1043, found 191.1025.

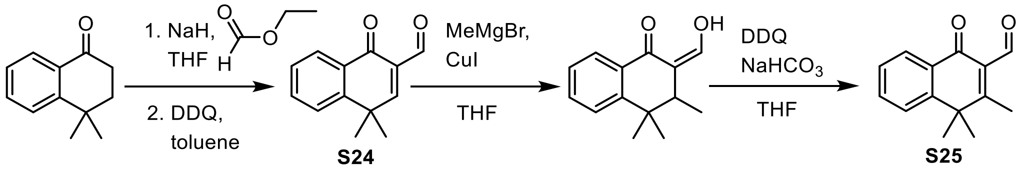


**4,4-Dimethyl-1-oxo-1,4-dihydronaphthalene-2-carbaldehyde (S24).** To a stirred solution of 4,4-dimethyl-3,4-dihydronaphthalen-1(2*H*)-one (3.48 g, 20.0 mmol) and ethyl formate (7.41 g, 100.0 mmol) in dry THF (60 mL) at room temperature was added 60% NaH (1.20 g, 30.0 mmol) portionwise under $N_2$. The mixture was then allowed to react at reflux for 6 h under $N_2$. After the reaction was complete, 5% $HCl_{(aq)}$ (40 mL) was added to quench the reaction at 0 °C, and the resulting mixture was extracted with EtOAc (60 mL × 2). The organic portions were combined, washed with sat. $NaHCO_{3(aq)}$ and brine, dried over $MgSO_4$, filtered and concentrated to give the 2-formylketone intermediate. To a stirred solution of the

2-formylketone intermediate in dry toluene (60 mL) was added DDQ (4.99 g, 22.0 mmol) in one portion. The resulting mixture was then stirred at 60 °C under $N_2$ for 2 h. After reaction was complete, the reaction mixture was filtrated with celite, and quenched with saturated $NaHCO_{3(aq)}$ (60 mL) and extracted with EtOAc (50 mL × 3). The combined organic extract was washed with saturated $NaHCO_{3(aq)}$ and brine, dried over $MgSO_4$, filtered and concentrated to give the crude residue, which was purified by chromatography on silical gel to afford 2-formylenone **S24** (2.92 g, 73% yield over 2 steps) as a white solid: mp = 102–105 °C; IR (KBr, $cm^{-1}$) $\nu_{max}$ 3198, 2973, 2919, 2874, 2850, 1706, 1663, 1620, 1600; $^{1}H$ NMR ($CDCl_3$, 400 MHz): $\delta$ 1.58 (s, 6H), 7.46 (td, *J* = 7.2, 1.2 Hz, 1H), 7.57 (dd, *J* = 8.0, 1.2 Hz, 1H), 7.65 (td, *J* = 7.2, 1.2 Hz, 1H), 7.72 (s, 1H), 8.26 (dd, *J* = 8.0, 1.2 Hz, 1H), 10.40 (s, 1H); $^{13}C$ NMR ($CDCl_3$, 100 MHz): $\delta$ 29.1 ($CH_3$), 37.7 (C), 126.2 (CH), 126.9 (CH), 127.2 (CH), 130.1 (C), 130.6 (C), 133.5 (CH), 162.1 (CH), 183.5 (C), 190.1 (CHO); HRMS (ESI) m/z: $[M + Na]^+$ calcd. for $C_{13}H_{12}O_2Na$ 223.0730, found 223.0735.

**3,4,4-Trimethyl-1-oxo-1,4-dihydronaphthalene-2-carbaldehyde (S25).** To a stirred solution of CuI (2.29 g, 12.0 mmol) in dry THF (20 mL) at 0 °C was added MeMgBr solution (4 mL, 3.0 M in $Et_2O$, 12.0 mmol) dropwise under $N_2$. The resulting mixture was then allowed to react at 0 °C for 30 min under $N_2$. To this stirred mixture at 0 °C was then added the 2-formylenone **S24** solution (2.00 g in 10 mL dry THF, 10.0 mmol) dropwise under $N_2$ for 30 min. After the reaction was complete, sat. $NH_4Cl_{(aq)}$ (20 mL) was added to quench the reaction at 0 °C, and the resulting mixture was diluted with EtOAc (100 mL) and filtrated with celite. The organic layer was separated, and the aqueous layer was extracted with EtOAc (25 mL × 2). The organic portions were combined, washed with water and brine, dried over $MgSO_4$, filtered and concentrated to give the crude residue, which was purified by chromatography on silical gel to afford 3-methyl-2-ketone intermediate. To a stirred solution of the 3-methyl-2-ketone intermediate and $NaHCO_3$ (840 mg, 10.0 mmol) in dry THF (50 mL), which was previously degassed with argon, was added DDQ (2.27 g, 10.0 mmol) in one portion at 0 °C. The resulting mixture was then stirred at room temperature under argon for 6 h. After reaction was complete, the reaction mixture was quenched with saturated $NaHCO_{3(aq)}$ (50 mL) and extracted with EtOAc (50 mL × 3). The combined organic extract was washed with saturated $NaHCO_{3(aq)}$ (50 mL × 3) and brine, dried over $MgSO_4$, filtered and concentrated to give the crude residue, which was purified by chromatography on neutralized silica gel to afford 3-methyl-2-formylenone **S25** (1.09 g, 51% yield over 2 steps) as a white solid: mp = 131–133 °C; IR (KBr, $cm^{-1}$) $\nu_{max}$ 3054, 2989, 2936, 2870, 1699, 1648, 1602, 1571, 1472, 1454; $^{1}H$ NMR ($CDCl_3$, 400 MHz): $\delta$ 1.60 (s, 6H), 2.50 (s, 3H), 7.41-7.45 (m, 1H), 7.59-7.66 (m, 2H), 8.22-8.24 (m, 1H), 10.61 (s, 1H); $^{13}C$ NMR ($CDCl_3$, 75 MHz): $\delta$ 17.7 ($CH_3$), 28.4 (CH), 41.5 (C), 126.4 (CH), 127.0 (CH), 129.2 (C), 129.3 (C), 133.3 (CH), 149.8 (C), 173.8 (C), 184.0 (C), 194.5 (CHO); HRMS (ESI) m/z: $[M + Na]^+$ calcd. for $C_{14}H_{14}O_2Na$ 237.0886, found 237.0881.

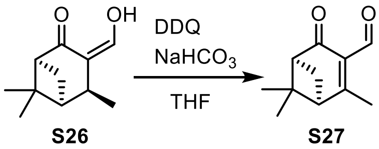


**(1*R*,5*S*)-2,6,6-Trimethyl-4-oxobicyclo[3.1.1]hept-2-ene-3-carbaldehyde (S27).** To a stirred solution of the 3-methyl-2-formylketone **S26** (1.80 g, 10.0 mmol) and $NaHCO_3$ (840 mg, 10.0 mmol) in dry THF (50 mL), which was previously degassed with argon, was added DDQ (2.27 g, 10.0 mmol) in one portion at 0 °C. The resulting mixture was then stirred at room temperature under argon for 10 min. After reaction was complete, the reaction mixture was quenched with saturated $NaHCO_{3(aq)}$ (50 mL) and extracted with EtOAc (50 mL × 3). The combined organic extract was washed with saturated $NaHCO_{3(aq)}$ (50 mL × 3) and brine, dried over $MgSO_4$, filtered and concentrated to give the crude residue, which was purified by chromatography on neutralized silica gel to afford 3-methyl-2-formylenone **S27** (1.34 g, 75% yield) as a light yellow oil: IR ($CH_2Cl_2$ cast, cm$^{-1}$) $\nu_{max}$ 2957, 2871, 1683, 1648, 1587; $^1$H NMR ($CDCl_3$, 400 MHz): $\delta$ 1.01 (s, 3H), 1.53 (s, 3H), 2.10 (d, *J* = 9.6 Hz, 1H), 2.51 (s, 3H), 2.65 (dt, *J* = 6.0, 5.6 Hz, 1H), 2.79 (t, *J* = 6.0 Hz, 1H), 2.84 (dt, *J* = 9.6, 5.6 Hz, 1H), 10.25 (s, 1H); $^{13}$C NMR ($CDCl_3$, 75 MHz): $\delta$ 21.9 ($CH_3$), 22.2 ($CH_3$), 26.4 ($CH_3$), 38.5 ($CH_2$), 52.7 (C), 53.4 (CH), 57.3 (CH), 126.4 (C), 181.7 (C), 191.2(C), 201.5 (CHO); HRMS (ESI) m/z: [M + Na]$^+$ calcd. for $C_{11}H_{14}O_2Na$ 201.0886, found 201.0886.

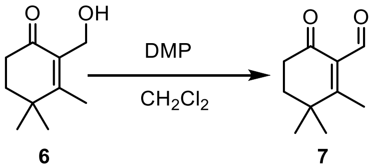


**2,3,3-Trimethyl-6-oxocyclohex-1-ene-1-carbaldehyde (7).** To a stirred solution of the 2-(hydroxymethyl) enone **6** (168 mg, 1.0 mmol) in dry $CH_2Cl_2$ (10 mL) was added Dess–Martin periodinane (551 mg, 1.3 mmol) in one portion at 0 °C. The resulting mixture was then stirred at room temperature under $N_2$ for 30 min. After reaction was complete, the reaction mixture was quenched with 5% $Na_2S_2O_{3(aq)}$ (20 mL). The resulting solution was then stirred at room temperature for 20 min. The aquous layer was extracted with $CH_2Cl_2$ (10 mL × 2). The organic portions were combined, washed with sat. $NaHCO_{3(aq)}$ and brine, dried over $MgSO_4$, filtered and concentrated to give the 3-methyl-2-formylenone **7** (158 mg, 95% yield) as a light yellow solid: mp = 63–65 °C; IR (KBr, cm$^{-1}$) $\nu_{max}$ 2959, 2932, 2863, 2774, 1694, 1665, 1567; $^1$H NMR ($CDCl_3$, 400 MHz): $\delta$ 1.25 (s, 6H), 1.89 (t, *J* = 6.8 Hz, 2H), 2.28 (s, 3H), 2.54 (t, *J* = 6.8 Hz, 2H), 10.25 (s, 1H); $^{13}$C NMR ($CDCl_3$, 100 MHz): $\delta$ 17.1 ($CH_3$), 26.2 ($CH_3$), 34.3 ($CH_2$), 36.0 ($CH_2$), 37.7 (C), 130.3 (C), 178.4 (C), 193.9 (CHO), 198.2 (CO); HRMS (ESI) m/z: [M + Na]$^+$ calcd. for $C_{10}H_{14}O_2Na$ 189.0886, found 189.0887.

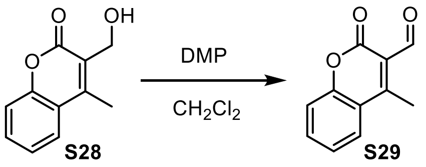

**4-Methyl-2-oxo-2*H*-chromene-3-carbaldehyde (S29).** To a stirred solution of the 2-(hydroxymethyl) enone **S28** (190 mg, 1.0 mmol) in dry $CH_2Cl_2$ (10 mL) was added Dess–Martin periodinane (551 mg, 1.3 mmol) in one portion at 0 °C. The resulting mixture was then stirred at room temperature under $N_2$ for 15 h. After reaction was complete, the reaction mixture was quenched with 5% $Na_2S_2O_{3(aq)}$ (50 mL). The resulting solution was then stirred at room temperature for 20 min. The aquous layer was extracted with $CH_2Cl_2$ (10 mL × 2). The organic portions were combined, washed with sat. $NaHCO_{3(aq)}$ and brine, dried over $MgSO_4$, filtered and concentrated to give the 3-methyl-2-formylenone **S29** (175 mg, 93% yield) as a light yellow solid: mp = 136–139 °C; IR ($CH_2Cl_2$ cast, $cm^{-1}$) $\nu_{max}$ 3113, 3078, 3056, 2918, 2873, 2850, 1719, 1695, 1608, 1596, 1555, 1452; $^1$H NMR ($CDCl_3$, 400 MHz): $\delta$ 2.88 (s, 3H), 7.37-7.41 (m, 2H), 7.64-7.69 (m, 1H), 7.88-7.91 (m, 1H), 10.47 (s, 1H); $^{13}$C NMR ($CDCl_3$, 150 MHz): $\delta$ 14.7 ($CH_3$), 117.4 (CH), 119.0 (C), 120.2 (C), 125.0 (CH), 126.9 (CH), 134.3 (CH), 153.9 (C), 158.7 (C), 160.9 (C), 191.5 (CHO); HRMS (ESI) m/z: $[M + Na]^+$ calcd. for $C_{11}H_8O_3Na$ 211.0366, found 211.0371.

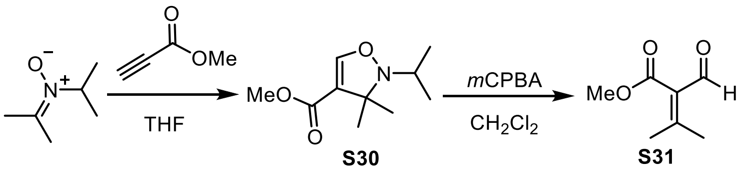

**Methyl 2-isopropyl-3,3-dimethyl-2,3-dihydroisoxazole-4-carboxylate (S30).**[11] To a stirred solution of the *N*-(propan-2-yl)propanimine oxide (1.15 g, 10.0 mmol) in dry THF (30 mL) was added methyl propilote (1.68 g, 20.0 mmol) in one portion. The resulting mixture was then stirred at 40 °C under $N_2$ for 3 h. After reaction was complete, the reaction mixture was concentrated to give the crude residue, which was purified by chromatography on silical gel to afford isoxazoline **S30** (1.65 g, 83% yield) as a colorless oil: IR ($CH_2Cl_2$ cast, $cm^{-1}$) $\nu_{max}$ 3093, 2978, 2951, 2876, 1711, 1626, 1458, 1438; $^1$H NMR ($CDCl_3$, 400 MHz): $\delta$ 1.19 (d, $J$ = 6.4 Hz, 6H), 1.49 (s, 6H), 3.38 (septet, $J$ = 6.4 Hz, 1H), 3.70 (s, 3H), 7.26 (s, 1H); $^{13}$C NMR ($CDCl_3$, 100 MHz): $\delta$ 21.2 ($CH_3$), 24.1 ($CH_3$), 50.8 ($CH_3$), 52.7 (CH), 67.2 (C), 115.0 (C), 152.9 (CH), 164.1 (C); HRMS (ESI) m/z: $[M + Na]^+$ calcd. for $C_{10}H_{17}NO_3Na$ 222.1101, found 222.1105.

**Methyl 2-formyl-3-methylbut-2-enoate (S31).**[12] To a stirred solution of the isoxazoline **S30** (996 mg, 5.0 mmol) in dry $CH_2Cl_2$ (50 mL) was added 70% *m*CPBA (1.12 g, 6.5 mmol) in one portion at 0 °C. The resulting mixture was then stirred at room temperature under $N_2$

for 30 min. After reaction was complete, the reaction mixture was quenched with 5% $Na_2S_2O_{3(aq)}$ (50 mL). The resulting solution was then stirred at room temperature for 30 min. The aquous layer was extracted with $CH_2Cl_2$ (20 mL). The organic portions were combined, washed with sat. $NaHCO_{3(aq)}$ and brine, dried over $MgSO_4$, filtered and concentrated to give the cross-conjugated enone **S31** (654 mg, 92% yield) as a colorless oil: IR ($CH_2Cl_2$ cast, $cm^{-1}$) $\nu_{max}$ 2999, 2954, 2919, 2850, 1736, 1666, 1632, 1601, 1436, 1375, 1317; $^1$H NMR ($CDCl_3$, 400 MHz): $\delta$ 2.10 (s, 3H), 2.27 (s, 3H), 3.84 (s, 3H), 9.96 (s, 3H); $^{13}$C NMR ($CDCl_3$, 150 MHz): $\delta$ 20.9 ($CH_3$), 24.9 ($CH_3$), 52.1 ($CH_3$), 132.4 (C), 161.7 (C), 166.9 (CO), 188.0 (CHO); HRMS (ESI) m/z: $[M + Na]^+$ calcd. for $C_7H_{10}O_3Na$ 165.0522, found 165.0522.

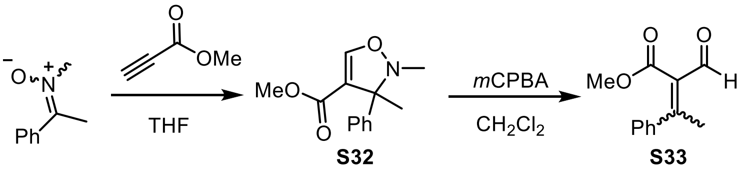


**Methyl 2,3-dimethyl-3-phenyl-2,3-dihydroisoxazole-4-carboxylate (S32).** According to the synthetic procedures similar to compound **S30**, isoxazoline **S32** (1.77 g, 76% yield) was prepared from the corresponding nitrone (1.49 g, 10.0 mmol) and methyl propilote (1.68 g, 20.0 mmol) as a colorless oil: IR ($CH_2Cl_2$ cast, $cm^{-1}$) $\nu_{max}$ 3089, 3059, 3024, 2986, 2951, 2920, 2888, 2850, 1712, 1619, 1437, 1343; $^1$H NMR ($CDCl_3$, 400 MHz): $\delta$ 1.83 (s, 3H), 2.60 (s, 3H), 3.65 (s, 3H), 7.25-7.30 (m, 1H), 7.33-7.37 (m, 2H), 7.46 (s, 1H), 7.48-7.50 (m, 2H); $^{13}$C NMR ($CDCl_3$, 100 MHz): $\delta$ 21.1 ($CH_3$), 39.5 ($CH_3$), 51.0 ($CH_3$), 72.0 (C), 114.1 (C), 126.9 (CH), 127.6 (CH), 128.1 (CH), 141.5 (C), 153.8 (CH), 164.0 (CO); HRMS (ESI) m/z: $[M + Na]^+$ calcd. for $C_{13}H_{15}NO_3$ 256.0944, found 256.1953.

**Methyl (E)/(*Z*)-2-formyl-3-phenylbut-2-enoate (S33).** According to the synthetic procedures similar to compound **S31**, a mixture of cross-conjugated enones (*E*) and (*Z*)-**S33** ( 950 mg, 93% yield) was prepared from the corresponding isoxazoline **S32** (1.17 g, 5.0 mmol) as a light yellow oil: IR ($CH_2Cl_2$ cast, $cm^{-1}$) $\nu_{max}$ 3058, 3000, 2953, 2919, 2851, 2741, 1736, 1673, 1616, 1573, 1491, 1435; $^1$H NMR ($CDCl_3$, 400 MHz) mixture of *E*/*Z* = 10/1: $\delta$ 2.36 (s, 3H), 2.58 (s, 0.3H), 3.54 (s, 0.3H), 3.90 (s, 3H), 7.29-7.46 (m, 5.5H), 9.36 (s, 1H), 10.10 (s, 0.1H); $^{13}$C NMR ($CDCl_3$, 150 MHz) major *E*-form: $\delta$ 24.2 ($CH_3$), 52.4 ($CH_3$), 128.4 (CH), 128.6 (CH), 129.7 (CH), 134.5 (C), 137.4 (C), 161.4 (C), 166.8 (CO), 189.2 (CHO); HRMS (ESI) m/z: $[M + Na]^+$ calcd. for $C_{12}H_{12}O_3Na$ 227.0679, found 227.0671.

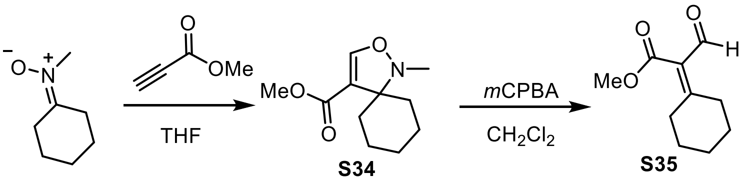

**Methyl 1-methyl-2-oxa-1-azaspiro[4.5]dec-3-ene-4-carboxylate (S34).** According to the synthetic procedures similar to compound **S30**, isoxazoline **S34** (1.52 g, 72% yield) was prepared from the corresponding nitrone (1.27 g, 10.0 mmol) and methyl propilote (1.68 g, 20.0 mmol) as a colorless oil: IR ($CH_2Cl_2$ cast, $cm^{-1}$) $\nu_{max}$ 3091, 2931, 2858, 1713, 1616, 1436; $^1H$ NMR ($CDCl_3$, 400 MHz): $\delta$ 1.22-1.35 (m, 2H), 1.58-1.69 (m, 8H), 2.72 (d, $J$ = 0.8 Hz, 3H), 3.71 (s, 3H), 7.33 (q, $J$ = 0.8 Hz, 1H); $^{13}C$ NMR ($CDCl_3$, 150 MHz): $\delta$ 22.8 (br, $CH_2$), 25.1 ($CH_2$), 35.7 (br, $CH_2$), 39.9 ($CH_3$), 50.9 ($CH_3$), 69.6 (C), 112.7 (C), 154.2 (CH), 164.6 (CO); HRMS (ESI) m/z: $[M + Na]^+$ calcd. for $C_{11}H_{17}NO_3Na$ 234.1101, found 234.1103.

**Methyl 2-cyclohexylidene-3-oxopropanoate (S35).** According to the synthetic procedures similar to compound **S31**, cross-conjugated enone **S35** (866 mg, 95% yield) was prepared from the corresponding isoxazoline **S34** (1.06 g, 5.0 mmol) as a colorless oil: IR ($CH_2Cl_2$ cast, $cm^{-1}$) $\nu_{max}$ 2936, 2859, 1737, 1668, 1624, 1446, 1436; $^1H$ NMR ($CDCl_3$, 400 MHz): $\delta$ 1.64-1.70 (m, 2H), 1.74-1.82 (m, 4H), 2.37 (t, $J$ = 6.4 Hz, 2H), 2.75 (t, $J$ = 6.4 Hz, 2H), 3.82 (s, 3H), 10.01 (s, 1H); $^{13}C$ NMR ($CDCl_3$, 100 MHz): $\delta$ 25.9 ($CH_2$), 28.2 ($CH_2$), 28.3 ($CH_2$), 29.7 ($CH_2$), 52.2 ($CH_3$), 131.0 (C), 167.0 (C), 167.2 (CO), 187.0 (CHO); HRMS (ESI) m/z: $[M + H]^+$ calcd. for $C_{10}H_{15}O_3$ 183.1016, found 183.1017.

**Synthetic procedures and characterization of new anionic Diels-Alder compounds**

General procedure for aldehyde-type anionic Diels-Alder reaction:

Condition A (one step):

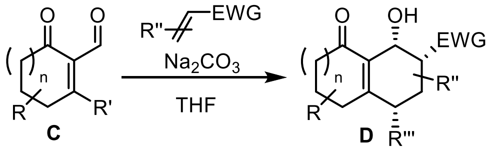


To a stirred solution of the cross-conjugated enone **C** (1.0 mmol) and $Na_2CO_3$ (127 mg, 1.2 mmol) in dry THF (5 mL), which was previously degassed with argon, was added dienophile (1.2 mmol) in one portion at room temperature. The resulting mixture was then stirred under individual temperature and time as indicated in Table 2. After reaction was complete, the reaction mixture was quenched with sat. $NH_4Cl_{(aq)}$ and extracted with EtOAc (10 mL × 2). The combined organic extract was washed with water and brine, dried over $MgSO_4$, filtered and concentrated to give the crude residue, which was purified by chromatography on silica gel to afford anionic Diels-Alder adduct **D**, including compounds **9**, **11**, **15**, **19-22**, **24**, **25**, **28** and **34** as indicated below.

Condition B (two steps):

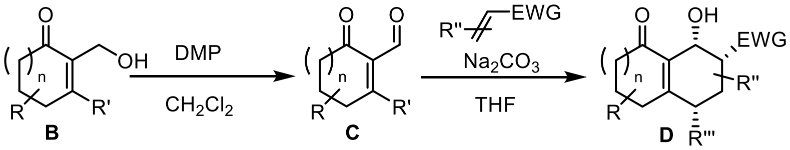


To a stirred solution of the 2-(hydroxymethyl) enone **B** (1.0 mmol) in dry $CH_2Cl_2$ (10 mL) was added Dess–Martin periodinane (551 mg, 1.3 mmol) in one portion at 0 °C. The resulting mixture was then stirred at room temperature under $N_2$ for 30 min. After reaction was complete, the reaction mixture was quenched with 5% $Na_2S_2O_{3(aq)}$ (20 mL). The resulting solution was then stirred at room temperature for 20 min. The aqueous layer was extracted with $CH_2Cl_2$ (10 mL × 2). The organic portions were combined, washed with sat. $NaHCO_{3(aq)}$ and brine, dried over $MgSO_4$, filtered and concentrated to give the cross-conjugated enone **C** individually. To a stirred solution of the crude cross-conjugated enone **C** and $Na_2CO_3$ (127 mg, 1.2 mmol) in dry THF (5 mL), which was previously degassed with argon, was added dienophile (1.2 mmol) in one portion at room temperature. The resulting mixture was then stirred under individual temperature and time as indicated in Table 2. After reaction was complete, the reaction mixture was quenched with sat. $NH_4Cl_{(aq)}$ and extracted with EtOAc (10 mL × 2). The combined organic extract was washed with water and brine, dried over $MgSO_4$, filtered and concentrated to give the crude residue, which was purified by chromatography on silica gel to afford anionic Diels-Alder adduct **D**, including compounds **12-14**, **16-18**, **23**, **26**, **27**, **29-33** and **35-37** as indicated below.

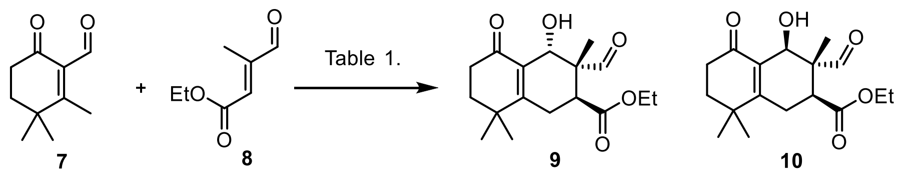


**7** **8** **9** **10**

**Ethyl (2*S**,3*R**,4*R**)-3-formyl-4-hydroxy-3,8,8-trimethyl-5-oxo-1,2,3,4,5,6,7,8-octahydronaphthalene-2-carboxylate (9).** Following the general procedure of condition A, compound **9** (299 mg, 97% yield) was prepared from enone **7** (166 mg, 1.0 mmol) and dienophile **8** (171 mg, 1.2 mmol) as a white solid: mp = 97–98 °C; IR (KBr, $cm^{-1}$) $\nu_{max}$ 3454 (br), 2967, 2934, 2872, 1729, 1667, 1621; $^{1}H$ NMR ($CDCl_3$, 400 MHz): $\delta$ 0.99 (s, 3H), 1.21 (s, 3H), 1.24 (s, 3H), 1.27 (t, $J$ = 7.2 Hz, 3H), 1.85-1.91 (m, 2H), 2.46-2.59 (m, 3H), 2.71 (dd, $J$ = 20.0, 5.6 Hz, 1H), 2.83 (d, $J$ = 3.2 Hz, 1H), 3.39 (dd, $J$ = 11.2, 5.6 Hz, 1H), 4.13-4.23 (m, 2H), 4.56 (d, $J$ = 3.2 Hz, 1H), 9.81 (s, 1H); $^{13}C$ NMR ($CDCl_3$, 100 MHz): $\delta$ 12.5 ($CH_3$), 14.0 ($CH_3$), 25.7 ($CH_3$), 26.1 ($CH_2$), 27.2 ($CH_3$), 34.1 ($CH_2$), 35.3 (C), 36.8 ($CH_2$), 38.1 (CH), 49.2 (C), 61.1 ($CH_2$), 69.1 (CH), 130.8 (C), 164.2 (C), 173.2 (CO), 198.9 (CO), 204.4 (CHO); HRMS (ESI) m/z: $[M + Na]^+$ calcd. for $C_{17}H_{24}O_5Na$ 331.1516, found 331.1514.

**Ethyl (2*S**,3*R**,4*S**)-3-formyl-4-hydroxy-3,8,8-trimethyl-5-oxo-1,2,3,4,5,6,7,8-octahydronaphthalene-2-carboxylate (10).** As listed in Table 1, isomer **10** was also isolated as a white solid: mp = 65–67 °C; IR (KBr, $cm^{-1}$) $\nu_{max}$ 3474 (br), 2966, 2932, 2852, 1731, 1648, 1613; $^{1}H$ NMR ($CDCl_3$, 400 MHz): $\delta$ 1.19 (s, 3H), 1.20 (s, 3H), 1.23 (s, 3H), 1.25 (t, $J$ = 7.2 Hz, 3H), 1.80 (dt, $J$ = 13.6, 5.2 Hz, 1H), 1.93 (ddd, $J$ = 13.6, 10.8, 6.0 Hz, 1H), 2.45-2.54 (m, 2H), 2.55-2.67 (m, 2H), 2.89 (dd, $J$ = 10.0, 6.0 Hz, 1H), 4.08-4.21 (m, 2H), 4.37 (d, $J$ = 2.4 Hz, 1H), 4.83 (d, $J$ = 2.4 Hz, 1H), 9.66 (s, 1H); $^{13}C$ NMR ($CDCl_3$, 100 MHz): $\delta$ 9.3 ($CH_3$), 14.0 ($CH_3$), 25.2 ($CH_3$), 25.8 ($CH_2$), 27.3 ($CH_3$), 34.3 ($CH_2$), 35.3 (C), 36.3 ($CH_2$), 42.3 (CH), 50.2 (C), 61.3 ($CH_2$), 68.6 (CH), 130.4 (C), 163.7 (C), 171.6 (CO), 200.6 (CO), 203.3 (CHO); HRMS (ESI) m/z: $[M + Na]^+$ calcd. for $C_{17}H_{24}O_5Na$ 331.1516, found 331.1513.

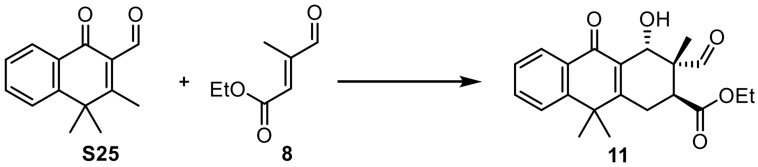

**S25** **8** **11**

**Ethyl (2*S**,3*R**,4*R**)-3-formyl-4-hydroxy-3,9,9-trimethyl-10-oxo-1,2,3,4,9,10-hexahydroanthracene-2-carboxylate (11).** Following the general procedure of condition A, compound **11** (342 mg, 96% yield) was prepared from enone **S25** (214 mg, 1.0 mmol) and dienophile **8** (171 mg, 1.2 mmol) as a white solid: mp = 152–154 °C; IR (KBr, $cm^{-1}$) $\nu_{max}$ 3462 (br), 3065, 2980, 2935, 1726, 1649, 1600, 1575, 1467; $^{1}H$ NMR ($CDCl_3$, 400 MHz): $\delta$ 1.06 (s, 3H), 1.29 (t, $J$ =

7.2 Hz, 3H), 1.56 (s, 3H), 1.59 (s, 3H), 2.69 (dd, *J* = 19.6, 11.6 Hz, 1H), 2.94 (dd, *J* = 19.6, 5.2 Hz, 1H), 3.17 (d, *J* = 2.8 Hz, 1H), 3.57 (dd, *J* = 11.6, 5.2 Hz, 1H), 4.14-4.29 (m, 2H), 4.83 (d, *J* = 2.8 Hz, 1H), 7.40-7.44 (m, 1H), 7.62-7.63 (m, 2H), 8.18 (d, 8.0 Hz, 1H), 9.91 (s, 1H); $^{13}$C NMR ($CDCl_3$, 100 MHz): $\delta$ 12.5 ($CH_3$), 14.1 ($CH_3$), 26.0 ($CH_2$), 27.7 ($CH_3$), 30.2 ($CH_3$), 38.4 (CH), 39.8 (C), 49.3 (C), 61.2 ($CH_2$), 69.6 (CH), 126.1 (CH), 126.6 (CH), 126.9 (CH), 129.5 (C), 130.7 (C), 133.1 (CH), 150.6 (C), 160.3 (C), 173.2 (CO), 184.4 (CO), 204.3 (CHO); HRMS (ESI) m/z: $[M + Na]^+$ calcd. for $C_{21}H_{24}O_5Na$ 379.1516, found 379.1509.

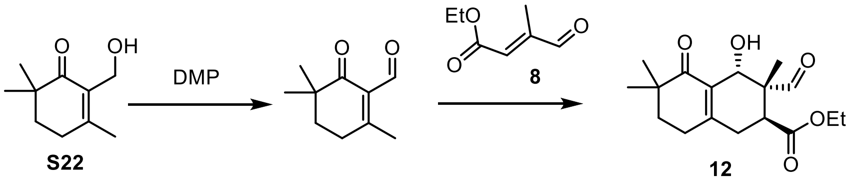


**Ethyl (2*S**,3*R**,4*R**)-3-formyl-4-hydroxy-3,6,6-trimethyl-5-oxo-1,2,3,4,5,6,7,8-octahydronaphthalene-2-carboxylate (12).** Following the general procedure of condition B, compound **12** (281 mg, 91% yield over 2 steps) was prepared from 2-(hydroxymethyl) enone **S22** (168 mg, 1.0 mmol) and dienophile **8** (171 mg, 1.2 mmol) as a white solid: mp = 138–140 °C; IR (KBr, $cm^{-1}$) $\nu_{max}$ 3430 (br), 2977, 2920, 2861, 1729, 1640; $^1$H NMR ($CDCl_3$, 400 MHz): $\delta$ 1.00 (s, 3H), 1.12 (s, 3H), 1.13 (s, 3H), 1.26 (t, *J* = 7.2 Hz, 3H), 1.84 (t, *J* = 6.4 Hz, 2H), 2.38 (t, *J* = 6.4 Hz, 2H), 2.52 (dd, *J* = 20.0, 10.8 Hz, 1H), 2.61 (dd, *J* = 20.0, 6.4 Hz, 1H), 2.72 (d, *J* = 3.2 Hz, 1H), 3.47 (dd, *J* = 10.8, 6.4 Hz, 1H), 4.12-4.21 (m, 2H), 4.53 (d, *J* = 3.2 Hz, 1H), 9.83 (s, 1H); $^{13}$C NMR ($CDCl_3$, 100 MHz): $\delta$ 12.8 ($CH_3$), 14.1 ($CH_3$), 24.0 ($CH_3$), 24.4 ($CH_3$), 27.4 ($CH_2$), 30.8 ($CH_2$), 35.3 ($CH_2$), 38.1 (CH), 40.7 (C), 49.8 (C), 61.1 ($CH_2$), 69.1 (CH), 130.3 (C), 155.5 (C), 173.0 (CO), 204.1 (CO), 204.5 (CHO); HRMS (ESI) m/z: $[M + Na]^+$ calcd. for $C_{17}H_{24}O_5Na$ 331.1516, found 331.1511.

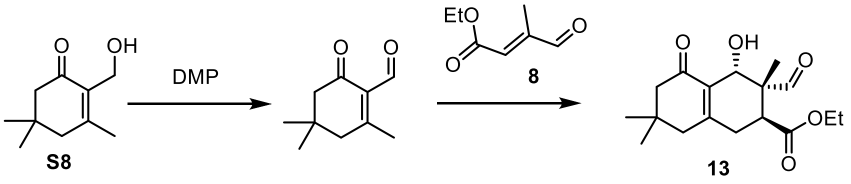


**Ethyl (2*S**,3*R**,4*R**)-3-formyl-4-hydroxy-3,7,7-trimethyl-5-oxo-1,2,3,4,5,6,7,8-octahydronaphthalene-2-carboxylate (13).** Following the general procedure of condition B, compound adduct **13** (296 mg, 96% yield over 2 steps) was prepared from 2-(hydroxymethyl) enone **S8** (168 mg, 1.0 mmol) and dienophile **8** (171 mg, 1.2 mmol) as a colorless oil: IR ($CH_2Cl_2$ cast, $cm^{-1}$) $\nu_{max}$ 3442 (br), 2958, 2903, 2871, 1728, 1666; $^1$H NMR ($CDCl_3$, 400 MHz): $\delta$ 1.00 (s,

3H), 1.01 (s, 3H), 1.08 (s, 3H), 1.26 (t, $J$ = 7.2 Hz, 3H), 2.22 (d, $J$ = 18.0 Hz, 1H), 2.31 (d, $J$ = 18.0 Hz, 1H), 2.32 (s, 2H), 2.50 (dd, $J$ = 20.0, 10.8 Hz, 1H), 2.60 (dd, $J$ = 20.0, 6.4 Hz, 1H), 2.82 (br s, 1H), 3.49 (dd, $J$ = 10.8, 6.4 Hz, 1H), 4.13-4.22 (m, 2H), 4.59 (s, 1H), 9.84 (s, 1H); $^{13}$C NMR ($CDCl_3$, 100 MHz): $\delta$ 12.6 ($CH_3$), 14.0 ($CH_3$), 27.2 ($CH_3$), 29.1 ($CH_3$), 31.2 ($CH_2$), 33.2 (C), 38.1 (CH), 44.4 ($CH_2$), 49.7 (C), 51.1 ($CH_2$), 61.1 ($CH_2$), 68.2 (CH), 131.0 (C), 155.6 (C), 172.9 (CO), 199.1 (CO), 204.6 (CHO); HRMS (ESI) m/z: [M + Na]$^+$ calcd. for $C_{17}H_{24}O_5Na$ 331.1516, found 331.1512.

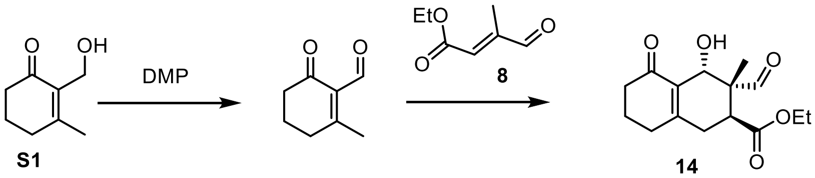


S1 14

**Ethyl (2*S**,3*R**,4*R**)-3-formyl-4-hydroxy-3-methyl-5-oxo-1,2,3,4,5,6,7,8-octahydronaphthalene-2-carboxylate (14).** Following the general procedure of condition B, compound **14** (247 mg, 88% yield over 2 steps) was prepared from 2-(hydroxymethyl) enone **S1** (140 mg, 1.0 mmol) and dienophile **8** (171 mg, 1.2 mmol) as a white solid: mp = 98–99 °C; IR (KBr, cm$^{-1}$) $\nu_{max}$ 3448 (br), 2980, 2921, 2850, 1727, 1663, 1621; $^1$H NMR ($CDCl_3$, 400 MHz): $\delta$ 1.01 (s, 3H), 1.26 (t, $J$ = 7.2 Hz, 3H), 1.94-2.09 (m, 2H), 2.39 (t, $J$ = 6.0 Hz, 2H), 2.40-2.55 (m, 2H), 2.55 (dd, $J$ = 20.4, 10.8 Hz, 1H), 2.61 (dd, $J$ = 10.8, 6.4 Hz, 1H), 2.86 (d, $J$ = 3.2 Hz, 1H), 3.48 (dd, $J$= 10.8, 6.4 Hz, 1H), 4.12-4.21 (m, 2H), 4.58 (d, $J$ = 3.2 Hz, 1H), 9.83 (s, 1H); $^{13}$C NMR ($CDCl_3$, 100 MHz): $\delta$ 12.6 ($CH_3$), 14.0 ($CH_3$), 21.9 ($CH_2$), 30.3 ($CH_2$), 31.0 ($CH_2$), 37.5 ($CH_2$), 38.0 (CH), 49.7 (C), 61.1 ($CH_2$), 68.5 (CH), 132.0 (C), 157.9 (C), 172.9 (CO), 198.9 (CO), 204.5 (CHO); HRMS (ESI) m/z: [M + Na]$^+$ calcd. for $C_{15}H_{20}O_5Na$ 303.1203, found 303.1206.

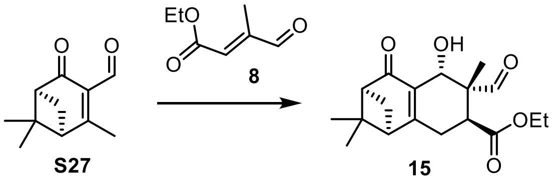


S27 15

**Ethyl (1*R**,3*S**,5*R**,6*R**,7*S**)-6-formyl-5-hydroxy-2,2,6-trimethyl-4-oxo-1,2,3,4,5,6,7,8-octahydro-1,3-methanonaphthalene-7-carboxylate (15).** Following the general procedure of condition A, compound **15** (285 mg, 89% yield) was prepared from enone **S27** (178 mg, 1.0 mmol) and dienophile **8** (171 mg, 1.2 mmol) as a white solid: mp = 121–123 °C; IR (KBr, cm$^{-1}$) $\nu_{max}$ 3447 (br), 2980, 2941, 2873, 1729, 1676, 1623; $^1$H NMR ($CDCl_3$, 400 MHz): $\delta$ 0.99 (s, 3H), 1.09 (s, 3H), 1.26 (t, $J$ = 7.2 Hz, 3H), 1.52 (s, 3H), 2.15 (d, $J$ = 9.6 Hz, 1H), 2.45 (t, $J$ = 5.6 Hz, 1H), 2.51 (dd, $J$ = 20.4, 9.6 Hz, 1H), 2.72-2.79 (m, 3H), 2.88 (dt, $J$ = 9.6, 5.6

Hz, 1H), 3.46 (dd, $J$ = 9.6, 6.2 Hz, 1H), 4.13-4.20 (m, 2H), 4.65 (d, $J$ = 2.4 Hz, 1H), 9.85 (s, 1H); $^{13}$C NMR ($CDCl_3$, 75 MHz): $\delta$ 13.2 ($CH_3$), 14.1 ($CH_3$), 22.2 ($CH_3$), 26.6 ($CH_3$), 30.4 ($CH_2$), 39.2 (CH), 41.0 ($CH_2$), 48.3 ($CH_3$), 49.7 (C), 54.6 (C), 57.6 (CH), 61.1 ($CH_2$), 67.3 (CH), 127.5 (C), 165.0 (C), 172.9 (CO), 202.9 (CO), 204.6 (CHO); HRMS (ESI) m/z: [M + Na]$^+$ calcd. for $C_{18}H_{24}O_5Na$ 343.1516, found 343.1514.

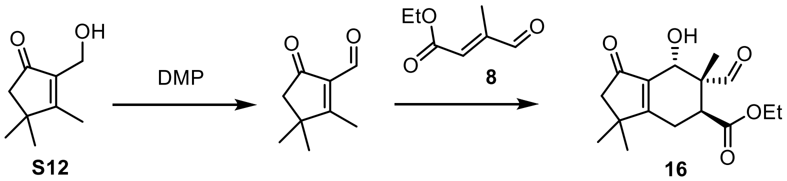

S12 16

**Ethyl (5S*,6R*,7R*)-6-formyl-7-hydroxy-3,3,6-trimethyl-1-oxo-2,3,4,5,6,7-hexahydro-1*H*-indene-5-carboxylate (16).** Following the general procedure of condition B, compound **16** (221 mg, 75% yield over 2 steps) was prepared from 2-(hydroxymethyl) enone **S12** (154 mg, 1.0 mmol) and dienophile **8** (171 mg, 1.2 mmol) as a white solid: mp = 129–131 °C; IR (KBr, cm$^{-1}$) $\nu_{max}$ 3426 (br), 2962, 2919, 2850, 1729, 1710, 1651; $^1$H NMR ($CDCl_3$, 400 MHz): $\delta$ 1.04 (s, 3H), 1.24 (s, 3H), 1.27 (s, 3H), 1.28 (t, $J$ = 7.2 Hz, 3H), 2.36 (d, $J$ = 18.8 Hz, 1H), 2.39 (d, $J$ = 18.8 Hz, 1H), 2.53 (dd, $J$ = 20.0, 9.6 Hz, 1H), 2.78 (dd, $J$ = 20.0, 6.0 Hz, 1H), 2.99 (d, $J$ = 3.0 Hz, 1H), 3.48 (dd, $J$ = 9.6, 6.0 Hz, 1H), 4.13-4.26 (m, 2H), 4.48 (d, $J$ = 3.0 Hz, 1H), 9.84 (s, 1H); $^{13}$C NMR ($CDCl_3$, 100 MHz): $\delta$ 13.1 ($CH_3$), 14.1 ($CH_3$), 23.4 ($CH_2$), 26.4 ($CH_3$), 26.8 ($CH_3$), 39.6 (CH), 40.8 (C), 50.3 (C), 51.5 ($CH_2$), 61.3 ($CH_2$), 67.0 (CH), 135.8 (C), 172.9 (C), 180.6 (CO), 204.5 (CHO), 206.3 (CO); HRMS (ESI) m/z: [M + Na]$^+$ calcd. for $C_{16}H_{22}O_5Na$ 317.1359, found 317.1361.

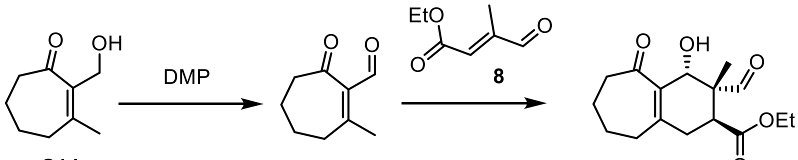

S14 17

**Ethyl (2*S**,3*R**,4*R**)-3-formyl-4-hydroxy-3-methyl-5-oxo-2,3,4,5,6,7,8,9-octahydro-1*H*-benzo[7]annulene-2-carboxylate (17).** Following the general procedure of condition B, compound **17** (253 mg, 86% yield over 2 steps) was prepared from 2-(hydroxymethyl) enone **S14** (154 mg, 1.0 mmol) and dienophile **8** (171 mg, 1.2 mmol) as a colorless oil: IR ($CH_2Cl_2$ cast, cm$^{-1}$) $\nu_{max}$ 3446 (br), 2979, 2939, 2869, 1728, 1652; $^1$H NMR ($CDCl_3$, 400 MHz): $\delta$ 0.98 (s, 3H), 1.26 (t, $J$ = 7.2 Hz, 3H), 1.71-1.87 (m, 4H), 2.37-2.72 (m, 6H), 2.92 (br s, 1H), 3.52 (dd, $J$ = 10.4, 7.2 Hz, 1H), 4.10-4.23 (m, 2H), 4.46 (s, 1H), 9.83 (s, 1H); $^{13}$C NMR ($CDCl_3$, 100

MHz): $\delta$ 12.4 ($CH_3$), 14.0 ($CH_3$), 20.6 ($CH_2$), 23.8 ($CH_3$), 32.9 ($CH_2$), 33.1 ($CH_2$), 38.2 (CH), 41.7 ($CH_2$), 49.4 (C), 61.0 ($CH_2$), 71.1 (CH), 134.9 (C), 154.1 (C), 173.0 (CO), 204.9 (CHO), 205.5 (CO); HRMS (ESI) m/z: $[M + Na]^+$ calcd. for $C_{16}H_{22}O_5Na$ 317.1359, found 317.1365.

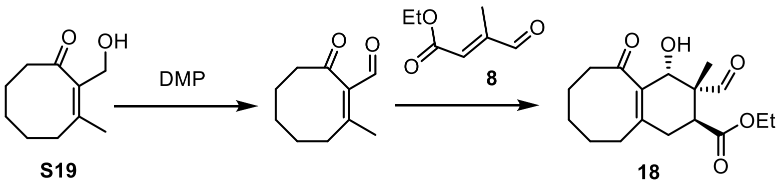


**Ethyl (2*S**,3*R**,4*R**)-3-formyl-4-hydroxy-3-methyl-5-oxo-1,2,3,4,5,6,7,8,9,10-decahydrobenzo[8]annulene-2-carboxylate (18).** Following the general procedure of condition B, compound **18** (163 mg, 53% yield over 2 steps) was prepared from 2-(hydroxymethyl) enone **S19** (168 mg, 1.0 mmol) and dienophile **8** (171 mg, 1.2 mmol) as a white solid: mp = 113–115 °C; IR (KBr, $cm^{-1}$) $\nu_{max}$ 3456 (br), 2979, 2931, 2856, 1727, 1671, 1648; $^1H$ NMR ($CDCl_3$, 400 MHz): $\delta$ 1.01 (s, 3H), 1.52-1.59 (m 2H), 1.66-1.89 (m 4H), 2.44-2.71 (m, 5H), 2.84 (ddd, $J$ = 17.2, 8.4, 6.0 Hz, 1H), 3.09 (br s, 1H), 3.53 (dd, $J$ = 10.8, 7.2 Hz, 1H), 4.10-4.23 (m, 2H), 4.34 (s, 1H), 9.82 (s, 1H); $^{13}C$ NMR ($CDCl_3$, 100 MHz): $\delta$ 12.4 ($CH_3$), 14.0 ($CH_3$), 22.5 ($CH_2$), 23.5 ($CH_2$), 25.0 ($CH_2$), 32.6 ($CH_2$), 34.2 ($CH_2$), 38.2 (CH), 43.4 ($CH_2$), 49.5 (C), 61.0 ($CH_2$), 72.3 (CH), 134.5 (C), 147.7 (C), 173.1 (CO), 204.9 (CHO), 207.7 (CO); HRMS (ESI) m/z: $[M + Na]^+$ calcd. for $C_{17}H_{24}O_5Na$ 331.1516, found 331.1513.

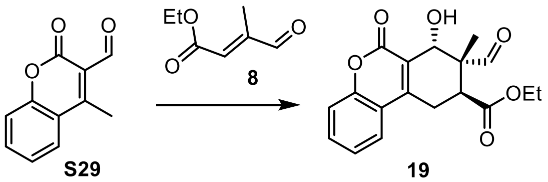


**Ethyl (7*R**,8*R**,9*S**)-8-formyl-7-hydroxy-8-methyl-6-oxo-7,8,9,10-tetrahydro-6*H*-benzo[*c*]chromene-9-carboxylate (19).** Following the general procedure of condition A, compound **19** (294 mg, 89% yield) was prepared from enone **S29** (188 mg, 1.0 mmol) and dienophile **8** (171 mg, 1.2 mmol) as a white solid: mp = 138–140 °C; IR (KBr, $cm^{-1}$) $\nu_{max}$ 3448 (br), 3362, 3080, 2980, 2918, 2850, 1720, 1630, 1608, 1453; $^1H$ NMR ($CDCl_3$, 400 MHz): $\delta$ 1.10 (s, 3H), 1.31 (t, $J$ = 7.2 Hz, 3H), 3.04 (dd, $J$ = 19.6, 11.2 Hz, 1H), 3.16 (d, $J$ = 3.4 Hz, 1H), 3.37 (dd, $J$ = 19.6, 6.0 Hz, ), 3.71 (dd, $J$ = 11.2, 6.0 Hz, 1H), 4.18-4.31 (m, 2H), 4.83 (d, $J$ = 3.4 Hz, 1H), 7.35-7.39 (m, 2H), 7.59 (td, $J$ = 7.6, 1.6 Hz, 1H), 7.70 (dd, $J$ = 8.0, 1.2 Hz, 1H), 9.93 (s, 1H); $^{13}C$ NMR ($CDCl_3$, 100 MHz): $\delta$ 12.6 ($CH_3$), 14.0 ($CH_3$), 25.1 ($CH_2$), 37.7 (CH), 49.6 (C), 61.5 ($CH_2$), 69.6 (CH), 117.1 (CH), 118.7 (C), 122.2 (C), 124.1 (CH), 124.7 (CH), 132.1 (CH),

147.2 (C), 152.5 (C), 161.2 (CO), 172.6 (CO), 203.7 (CHO); HRMS (ESI) m/z: $[M + Na]^+$ calcd. for $C_{18}H_{18}O_6Na$ 353.0996, found 353.0999.

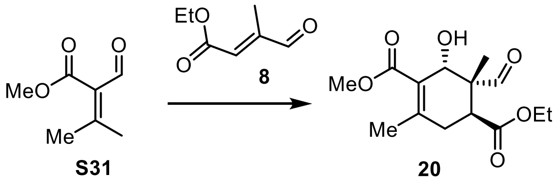


**4-Ethyl 1-methyl (4S*,5R*,6R*)-5-formyl-6-hydroxy-2,5-dimethylcyclohex-1-ene-1,4-dicarboxylate (20).** Following the general procedure of condition A, compound **20** (230 mg, 81% yield) was prepared from enone **S31** (142 mg, 1.0 mmol) and dienophile **8** (171 mg, 1.2 mmol) as a colorless oil: IR ($CH_2Cl_2$ cast, $cm^{-1}$) $\nu_{max}$ 3483 (br), 2983, 2951, 2851, 1724, 1645; $^1H$ NMR ($CDCl_3$, 400 MHz): $\delta$ 1.01 (s, 3H), 1.26 (t, $J$ = 7.2 Hz, 3H), 2.15 (s, 3H), 2.53 (dd, $J$ = 20.0, 11.2 Hz, 1H), 2.62 (dd, $J$ = 20.0, 6.4 Hz, 1H), 3.52 (dd, $J$ = 11.2, 6.4 Hz, 1H), 3.79 (s, 3H), 4.11-4.23 (m, 2H), 4.41 (s, 1H), 9.83 (s, 1H); $^{13}C$ NMR ($CDCl_3$, 150 MHz): $\delta$ 12.4 ($CH_3$), 14.1 ($CH_3$), 21.5 ($CH_3$), 33.4 ($CH_2$), 37.9 (CH), 49.6 (C), 51.7 ($CH_3$), 61.1 ($CH_2$), 71.9 (CH), 124.5 (C), 150.4 (C), 167.7 (CO), 172.9 (CO), 204.9 (CHO); HRMS (ESI) m/z: $[M + Na]^+$ calcd. for $C_{14}H_{20}O_6Na$ 307.1152, found 307.1151.

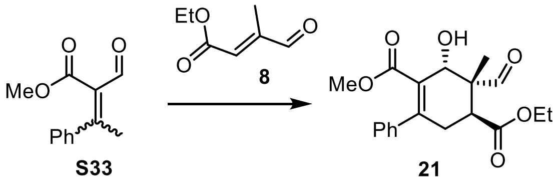


**5-Ethyl 2-methyl (3R*,4R*,5S*)-4-formyl-3-hydroxy-4-methyl-3,4,5,6-tetrahydro-[1,1'-biphenyl]-2,5-dicarboxylate (21).** Following the general procedure of condition A, compound **21** (291 mg, 84% yield) was prepared from the mixture enones (*E*)/(*Z*)-**S33** (204 mg, 1.0 mmol) and dienophile **8** (171 mg, 1.2 mmol) as a colorless oil: IR ($CH_2Cl_2$ cast, $cm^{-1}$) $\nu_{max}$ 3473 (br), 3057, 3022, 2982, 2938, 2853, 1726, 1648, 1435; $^1H$ NMR ($CDCl_3$, 400 MHz): $\delta$ 1.17 (s, 3H), 1.27 (t, $J$ = 7.2 Hz, 3H), 2.71 (dd, $J$ = 19.6, 11.2 Hz, 1H), 2.87 (dd, $J$ = 19.6, 6.0 Hz, 1H), 2.96 (d, $J$ = 2.8 Hz, 1H), 3.45 (s, 3H), 3.64 (dd, $J$ = 11.2, 6.0 Hz, 1H), 4.12-4.21 (m, 2H), 4.52 (d, $J$ = 2.8 Hz, 1H), 7.13-7.16 (m, 2H), 7.32-7.38 (m, 3H), 9.88 (s, 1H); $^{13}C$ NMR ($CDCl_3$, 100 MHz): $\delta$ 12.5 ($CH_3$), 14.0 ($CH_3$), 32.6 ($CH_2$), 38.3 (CH), 49.6 (C), 51.7 ($CH_3$), 61.1 ($CH_2$), 72.1 (CH), 126.4 (CH), 126.7 (C), 128.0 (CH), 128.2 (CH), 140.7 (C), 149.4 (C), 168.5 (CO), 172.7 (CO), 204.6 (CHO); HRMS (ESI) m/z: $[M + Na]^+$ calcd. for $C_{19}H_{22}O_6Na$ 369.1309, found 369.1305.

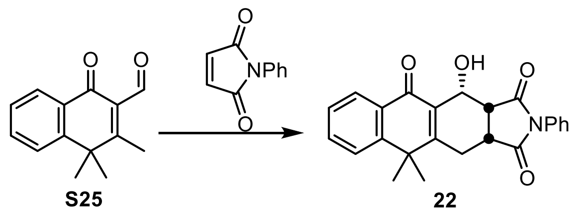

**(3a*R**,4*R**,11a*R**)-4-Hydroxy-10,10-dimethyl-2-phenyl-4,10,11,11a-tetrahydro-1*H*-naphtho[2,3-*f*]isoindole-1,3,5(2*H*,3a*H*)-trione (22).** Following the general procedure of condition A, compound **22** (368 mg, 95% yield) was prepared from enone **S25** (214 mg, 1.0 mmol) and *N*-phenylmaleimide ( 208 mg, 1.2 mmol) as a white solid: mp = 236–239 °C; IR (KBr, $cm^{-1}$) $\nu_{max}$ 3448 (br), 3064, 2976, 2920, 2850, 1778, 1708, 1644, 1599, 1499, 1467, 1388; $^1$H NMR ($CDCl_3$, 400 MHz): $\delta$ 1.56 (s, 3H), 1.59 (s, 3H), 2.59 (d, $J$ = 4.4 Hz, 1H), 3.02 (dt, $J$ = 15.2, 8.8 Hz, 1H), 3.18 (dd, $J$ = 8.8, 4.4, Hz, 1H), 3.18-3.32 (m, 2H), 5.98 (t, $J$ = 4.4 Hz, 1H), 7.26-7.34 (m, 2H), 7.40-7.44 (m, 2H), 7.44-7.51 (m, 2H), 7.60-7.66 (m, 2H), 8.23 (d, $J$ = 8.4 Hz, 1H); $^{13}$C NMR ($CDCl_3$, 150 MHz): $\delta$ 24.7 ($CH_2$), 27.2 ($CH_3$), 27.9 ($CH_3$), 37.9 (CH), 39.8 (C), 46.0 (CH), 60.7 (CH), 126.4 (CH), 126.6 (CH), 126.9 (CH), 127.0 (CH), 128.7 (CH), 129.1 (CH), 129.5 (C), 131.9 (C), 132.9 (CH), 133.1 (C), 150.1 (C), 164.1 (C), 175.8 (CO), 178.5 (CO), 181.4 (CO); HRMS (ESI) m/z: $[M + Na]^+$ calcd. for $C_{24}H_{21}NO_4Na$ 410.1363, found 410.1363.

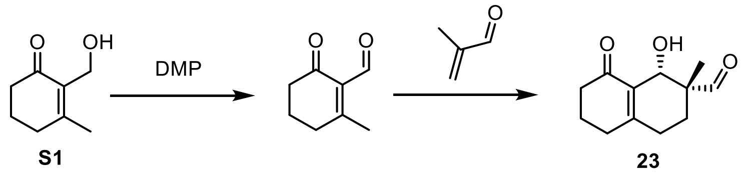

**(1*R**,2*R**)-1-Hydroxy-2-methyl-8-oxo-1,2,3,4,5,6,7,8-octahydronaphthalene-2-carbaldehyde (23).** Following the general procedure of condition B, compound **23** (196 mg, 94% yield over 2 steps) was prepared from 2-(hydroxymethyl) enone **S1** (140 mg, 1.0 mmol) and methacrolein (84 mg, 1.2 mmol) as a colorless oil: IR ($CH_2Cl_2$ cast, $cm^{-1}$) $\nu_{max}$ 3448 (br), 2934, 2875, 2825, 2715, 1726, 1656, 1632; $^1$H NMR ($CDCl_3$, 400 MHz): $\delta$ 1.01 (s, 3H), 1.56-1.62 (m, 1H), 1.91-2.08 (m, 4H), 2.22-2.52 (m, 5H), 3.28 (br s, 1H), 4.66 (s, 1H), 9.75 (s, 1H); $^{13}$C NMR ($CDCl_3$, 100 MHz): $\delta$ 17.2 ($CH_3$), 21.9 ($CH_2$), 22.9 ($CH_2$), 28.4 ($CH_2$), 30.8 ($CH_2$), 37.7 ($CH_2$), 48.1 (C), 67.2 (CH), 132.1 (C), 160.3 (C), 200.1 (CO), 205.0 (CHO); HRMS (ESI) m/z: $[M + Na]^+$ calcd. for $C_{12}H_{16}O_3Na$ 231.0992, found 231.0990.

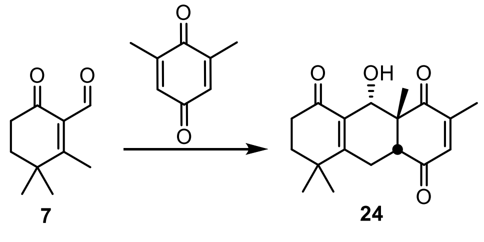

**(4a*R**,9a*R**,10*R**)-10-Hydroxy-3,4a,8,8-tetramethyl-6,7,8,9,9a,10-hexahydroanthracene-1,4,5(4a*H*)-trione (24).** Following the general procedure of condition A, anionic compound **24** (230 mg, 76% yield) was prepared from enone **7** (166 mg, 1.0 mmol) and 2,6-dimethylbenzoquinone (127 mg, 1.2 mmol) as a white solid: mp = 173–175 °C; IR (KBr, $cm^{-1}$) $\nu_{max}$ 3480 (br), 2965, 2928, 1677, 1622, 1468, 1377; $^1$H NMR ($CDCl_3$, 400 MHz): $\delta$ 1.22 (s, 3H), 1.23 (s, 3H), 1.35 (s, 3H), 1.81-1.95 (m, 2H), 2.01 (d, *J* = 1.6 Hz, 3H), 2.24 (dd, *J* = 20.0, 7.2 Hz, 1H), 2.41-2.58 (m, 2H), 2.66 (d, *J* = 2.8 Hz, 1H), 2.96 (dd, *J* = 7.2, 0.8 Hz, 1H), 3.31 (dd, *J* = 20.0, 0.8 Hz, 1H), 4.42 (d, *J* = 2.8 Hz, 1H), 6.68 (q, J = 1.6 Hz, 1H); $^{13}$C NMR ($CDCl_3$, 100 MHz): $\delta$ 16.4 ($CH_3$), 20.2 ($CH_3$), 21.7 ($CH_2$), 25.6 ($CH_3$), 27.2 ($CH_3$), 34.1 ($CH_2$), 35.4 (C), 36.6 ($CH_2$), 48.1 (CH), 50.1 (C), 68.9 (CH), 130.3 (C), 138.1 (CH), 150.5 (C), 163.5 (C), 196.8 (CO), 199.0 (CO), 201.4 (CO); HRMS (ESI) m/z: $[M + Na]^+$ calcd. for $C_{18}H_{22}O_4Na$ 325.1410, found 325.1407.

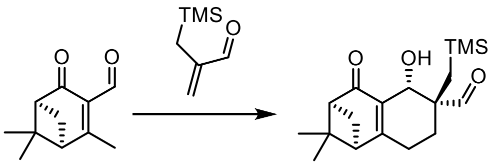


**S27** **25**

**(1*R**,3*S**,5*S**,6*S**)-5-Hydroxy-2,2-dimethyl-4-oxo-6-((trimethylsilyl)methyl)-1,2,3,4,5,6,7,8-octahydro-1,3-methanonaphthalene-6-carbaldehyde (25).** Following the general procedure of condition A, compound **25** (288 mg, 90% yield) was prepared from enone **S27** (178 mg, 1.0 mmol) and 2-((trimethylsilyl)methyl)acrolein (171 mg, 1.2 mmol) as a white solid: mp = 164–166 °C; IR (KBr, $cm^{-1}$) $\nu_{max}$ 3444 (br), 2951, 2925, 2851, 1725, 1663, 1636; $^1$H NMR ($CDCl_3$, 400 MHz): $\delta$ 0.05 (s, 9H), 0.83 (d, *J* = 15.2 Hz, 1H), 0.90 (d, *J* = 15.2 Hz, 1H), 0.98 (s, 3H), 1.50 (s, 3H), 1.62 (dt, *J* = 13.6, 5.2 Hz, 1H), 2.06-2.22 (m, 4H), 2.38 (t, *J* = 5.6 Hz, 1H), 2.53 (dtd, *J* = 18.8, 5.2, 1.2 Hz, 1H), 2.71 (t, *J* = 5.6 Hz, 1H), 2.82 (dt, *J* = 9.2, 5.6 Hz, 1H), 3.37 (d, *J* = 2.8 Hz, 1H), 4.71 (d, *J* = 2.8 Hz, 1H), 9.79 (s, 1H); $^{13}$C NMR ($CDCl_3$, 100 MHz): $\delta$ 0.51 ($CH_3$), 20.8 ($CH_2$), 22.2 ($CH_3$), 25.0 ($CH_2$), 26.6 ($CH_3$), 28.2 ($CH_2$), 40.8 ($CH_2$), 48.5 (CH), 51.0 (C), 55.0 (C), 57.8 (CH), 68.4 (CH), 127.9 (C), 167.5 (C), 204.1 (CO), 205.5 (CHO); HRMS (ESI) m/z: $[M + Na]^+$ calcd. for $C_{18}H_{28}O_3SiNa$ 343.1700, found 343.1700.

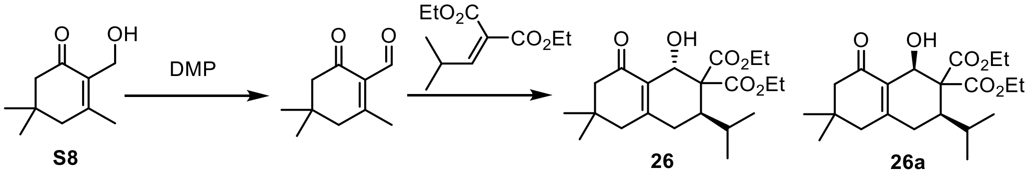


**S8** **26** **26a**

**Diethyl (1*S**,3*R**)-1-hydroxy-3-isopropyl-6,6-dimethyl-8-oxo-3,4,5,6,7,8-hexahydronaphthalene-**

**2,2(1*H*)-dicarboxylate (26).** Following the general procedure of condition B, compound **26** (289 mg, 76% yield over 2 steps) was prepared from 2-(hydroxymethyl) enone **S8** (168 mg, 1.0 mmol) and diethyl isobutylidenemalonate (257 mg, 1.2 mmol) as a white solid: mp = 112–114 °C; IR (KBr, $cm^{-1}$) $\nu_{max}$ 3450 (br), 2960, 2928, 2870, 1752, 1726, 1657; $^1$H NMR ($CDCl_3$, 400 MHz): $\delta$ 0.82 (d, *J* = 6.8 Hz, 3H), 0.99 (s, 3H), 1.05 (d, *J* = 6.8 Hz, 3H), 1.06 (s, 3H), 1.20 (t, *J* = 7.2 Hz, 3H), 1.29 (t, *J* = 7.2 Hz, 3H), 2.11-2.37 (m, 7H), 2.47 (ddd, *J* = 10.8, 6.0, 2.4 Hz, 1H), 3.24 (d, *J* = 3.2 Hz, 1H), 4.00-4.27 (m, 4H), 5.08 (d, *J* = 3.2 Hz, 1H); $^{13}$C NMR ($CDCl_3$, 100 MHz): $\delta$ 13.8 ($CH_3$), 14.0 ($CH_3$), 18.6 ($CH_3$), 24.0 ($CH_3$), 26.7 ($CH_3$), 27.5 (CH), 29.5 ($CH_3$), 30.9 (CH), 33.2 (C), 37.4 ($CH_3$), 44.7 ($CH_2$), 51.1 ($CH_2$), 60.9 ($CH_2$), 61.0 (C), 61.2 ($CH_2$), 65.1 (CH), 130.8 (C), 157.5 (C), 169.0 (CO), 170.6 (CO), 198.6 (CO); HRMS (ESI) m/z: $[M + Na]^+$ calcd. for $C_{21}H_{32}O_6Na$ 403.2091, found 403.2091.

**Diethyl (1*R**,3*R**)-1-hydroxy-3-isopropyl-6,6-dimethyl-8-oxo-3,4,5,6,7,8-hexahydronaphthalene-2,2(1*H*)-dicarboxylate (26a).** *cis*-isomer **26a** (15 mg, 4% yield) was also isolated as a white solid: mp = 78–80 °C; IR (KBr, $cm^{-1}$) $\nu_{max}$ 3488 (br), 2958, 2928, 2870, 1732, 1661; $^1$H NMR ($CDCl_3$, 400 MHz): $\delta$ 0.87 (d, *J* = 9.2 Hz, 3H), 0.99 (s, 3H), 1.01 (d, *J* = 9.2 Hz, 3H), 1.06 (s, 3H), 1.22 (t, *J* = 7.2 Hz, 3H), 1.28 (t, *J* = 7.2 Hz, 3H), 2.12-2.35 (m, 7H), 2.42-2.46 (m, 1H), 3.74 (d, *J* = 3.6 Hz, 1H), 4.13-4.18 (m, 2H), 4.21-4.27 (m, 2H), 5.27 (br s, 1H); $^{13}$C NMR ($CDCl_3$, 100 MHz): $\delta$ 13.9 ($CH_3$), 14.0 ($CH_3$), 19.2 ($CH_3$), 24.5 ($CH_3$), 27.7 ($CH_3$), 28.4 ($CH_3$), 28.7 (CH), 31.7 ($CH_2$), 33.0 (C), 40.8 (CH), 44.8 ($CH_2$), 51.3 ($CH_2$), 61.2 ($CH_2$), 61.6 ($CH_2$), 61.9 (C), 66.1 (CH), 130.6 (C), 156.7 (C), 169.5 (CO), 169.8 (CO), 199.1 (CO); HRMS (ESI) m/z: $[M + Na]^+$ calcd. for $C_{21}H_{32}O_6Na$ 403.2091, found 403.2093.

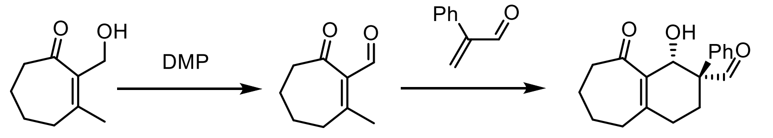


S14 27

**(1*R**,2*S**)-1-Hydroxy-9-oxo-2-phenyl-2,3,4,5,6,7,8,9-octahydro-1*H*-benzo[7]annulene-2-carbaldehyde (27).** Following the general procedure of condition B, compound **27** (247 mg, 87% yield over 2 steps) was prepared from 2-(hydroxymethyl) enone **S14** (154 mg, 1.0 mmol) and 2-phenylpropenal (159 mg, 1.2 mmol) as a white solid: mp = 175–177 °C; IR (KBr, $cm^{-1}$) $\nu_{max}$ 3378 (br), 3058, 2918, 2989, 2869, 1726, 1637, 1624, 1495, 1454; $^1$H NMR ($CDCl_3$, 400 MHz): $\delta$ 1.50-1.62 (m, 1H), 1.72-1.91 (m, 4H), 2.18-2.43 (m, 5H), 2.68 (t, *J* = 6.4 Hz, 2H), 2.96 (d, *J* = 3.6 Hz, 1H), 5.50 (d, *J* = 3.6 Hz, 1H), 7.11-7.14 (m, 2H), 7.25-7.35 (m, 3H), 9.55 (s, 1H); $^{13}$C NMR ($CDCl_3$, 100 MHz): $\delta$ 20.8 ($CH_2$), 22.6 ($CH_2$), 23.8 ($CH_2$), 31.1 ($CH_2$), 33.3 ($CH_2$), 41.8 ($CH_2$), 57.0 (C), 64.8 (CH), 127.5 (CH), 127.7 (CH), 128.8 (CH), 135.0 (C), 135.3 (C), 158.7 (C), 199.2 (CO), 205.5 (CO); HRMS (ESI) m/z: $[M + Na]^+$ calcd. for $C_{18}H_{20}O_3Na$ 307.1305, found 307.1314.

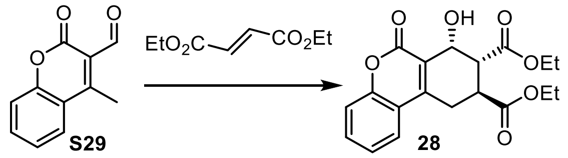

**Diethyl (7*R**,8*R**,9*S**)-7-hydroxy-6-oxo-7,8,9,10-tetrahydro-6*H*-benzo[*c*]chromene-8,9-dicarboxylate (28).** Following the general procedure of condition A, compound **28** (317 mg, 88% yield) was prepared from enone **S29** (188 mg, 1.0 mmol) and diethyl fumarate (207 mg, 1.2 mmol) as a white solid: mp = 129–130 °C; IR (KBr, $cm^{-1}$) $\nu_{max}$ 3482 (br), 3067, 2981, 2917, 2848, 1734, 1719, 1628, 1608; $^1$H NMR ($CDCl_3$, 400 MHz): $\delta$ 1.32 (t, $J$ = 7.2 Hz, 3H), 1.34 (t, $J$ = 7.2 Hz, 3H), 2.78 (dd, $J$ = 16.0, 3.6 Hz, 1H), 3.01 (d, $J$ = 3.2 Hz, 1H), 3.05 (dd, $J$ = 12.4, 3.6 Hz, 1H), 3.33-3.42 (m, 2H), 4.19-4.34 (m, 4H), 5.30 (t, $J$ = 3.2 Hz, 1H), 7.34 (t, $J$ = 8.2 Hz, 1H), 7.37 (d, $J$= 8.2 Hz, 1H), 7.57 (t, $J$ = 8.2 Hz, 1H), 7.60 (d, $J$ = 8.2 Hz, 1H); $^{13}$C NMR ($CDCl_3$, 100 MHz): $\delta$ 14.1 ($CH_3$) × 2, 28.9 ($CH_2$), 35.6 (CH), 46.8 (CH), 61.2 ($CH_2$) × 2, 62.8 (CH), 117.1 (CH), 118.6 (C), 123.1 (C), 124.0 (CH), 124.7 (CH), 132.0 (CH), 146.7 (C), 152.5 (C), 161.0 (CO), 170.9 (CO), 174.4 (CO); HRMS (ESI) m/z: $[M + Na]^+$ calcd. for $C_{19}H_{20}O_7Na$ 383.1101, found 383.1100.

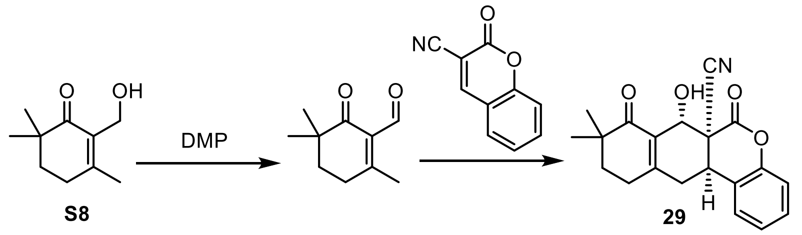

**(6a*R**,7*S**,12a*R**)-7-Hydroxy-9,9-dimethyl-6,8-dioxo-8,9,10,11,12,12a-hexahydro-6*H*-naphtho[2,3-*c*]chromene-6a(7*H*)-carbonitrile (29).** Following the general procedure of condition B, compound **29** (310 mg, 92% yield over 2 steps) was prepared from 2-(hydroxymethyl) enone **S8** (168 mg, 1.0 mmol) and 3-cyanocoumarin (205 mg, 1.2 mmol) as a white solid: mp = 194–197 °C; IR (KBr, $cm^{-1}$) $\nu_{max}$ 3357 (br), 3051, 2924, 2851, 1773, 1654; $^1$H NMR ($CDCl_3$, 400 MHz): $\delta$ 1.12 (s, 3H), 1.15 (s, 3H), 1.85 (t, $J$ = 6.0 Hz, 2H), 2.24-2.39 (m, 3H), 2.75 (dd, $J$ = 20.4, 6.4 Hz, 1H), 3.44 (d, $J$ = 4.0 Hz, 1H), 3.73 (dd, $J$ = 10.0, 6.4 Hz, 1H), 5.32 (d, $J$ = 4.0 Hz, 1H), 7.17-7.22 (m, 1H), 7.24 (dd, $J$ = 7.6, 1.2 Hz, 1H), 7.30 (dd, $J$ = 7.6, 1.6 Hz, 1H), 7.39-7.43 (m, 1H); $^{13}$C NMR ($CDCl_3$, 100 MHz): $\delta$ 24.0 ($CH_3$), 24.1 ($CH_3$), 27.3 ($CH_2$), 34.1 (CH), 34.5 ($CH_2$), 34.9 ($CH_2$), 40.8 (C), 47.7 (C), 62.4 (CH), 114.8 (C), 117.5 (CH), 123.9 (C), 125.8 (CH), 127.5 (CH), 129.1 (C), 130.1 (CH), 150.2 (C), 152.0 (C), 160.4 (CO), 203.0 (CO); HRMS (ESI) m/z: $[M + Na]^+$ calcd. for $C_{20}H_{19}NO_4Na$ 360.1206, found 360.1208.

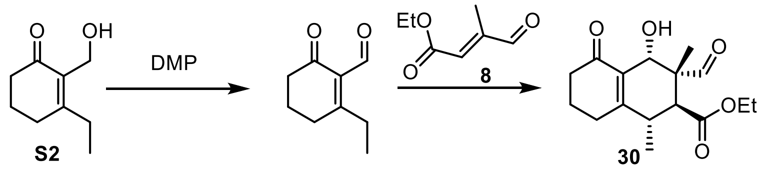

**Ethyl (1S*,2S*,3R*,4R*)-3-formyl-4-hydroxy-1,3-dimethyl-5-oxo-1,2,3,4,5,6,7,8-octahydronaphthalene-2-carboxylate (30).** Following the general procedure of condition B, compound **30** (247 mg, 84% yield over 2 steps) was prepared from 2-(hydroxymethyl) enone **S2** (154 mg, 1.0 mmol) and dienophile **8** (171 mg, 1.2 mmol) as a white solid: mp = 125–126 °C; IR (KBr, $cm^{-1}$) $\nu_{max}$ 3448 (br), 2980, 2938, 2882, 1726, 1664, 1630; $^1$H NMR ($CDCl_3$, 400 MHz): $\delta$ 0.96 (s, 3H), 1.23 (d, $J$ = 7.2 Hz, 3H), 1.27 (t, $J$ = 7.2 Hz, 3H), 1.84-1.95 (m, 1H), 2.05-2.13 (m, 1H), 2.73-2.40 (m, 2H), 2.47-2.55 (m, 2H), 2.74-2.81 (m, 1H), 2.78 (d, $J$ = 3.2 Hz, 1H), 3.16 (d, $J$ = 10.4 Hz, 1H), 4.13-4.24 (m, 2H), 4.59 (d, $J$ = 3.2 Hz, 1H), 9.83 (s, 1H); $^{13}$C NMR ($CDCl_3$, 100 MHz): $\delta$ 12.8 ($CH_3$), 14.0 ($CH_3$), 17.3 ($CH_3$), 22.1 ($CH_2$), 28.6 ($CH_2$), 35.4 (CH), 37.2 ($CH_2$), 45.7 (CH), 50.3 (C), 60.9 ($CH_2$), 67.5 (CH), 131.3 (C), 161.9 (C), 173.2 (CO), 199.0 (CO), 204.8 (CHO); HRMS (ESI) m/z: [M + Na]$^+$ calcd. for $C_{16}H_{22}O_5Na$ 317.1359, found 317.1360.

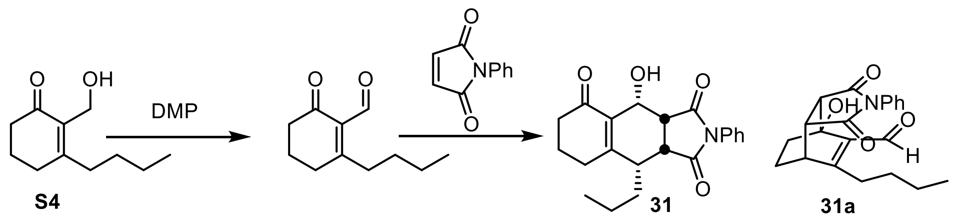

**(3a*R**,4*R**,9*S**,9a*R**)-4-Hydroxy-2-phenyl-9-propyl-4,6,7,8,9,9a-hexahydro-1*H*-benzo[*f*]isoindole-1,3,5(2*H*,3a*H*)-trione (31).** Following the general procedure of condition B, compound **31** (300 mg, 85% yield over 2 steps) was prepared from 2-(hydroxymethyl) enone **S4** (182 mg, 1.0 mmol) and *N*-phenylmaleimide (208 mg , 1.2 mmol) as a white solid: mp = 159–161 °C; IR (KBr, $cm^{-1}$) $\nu_{max}$ 3469, 2957, 2931, 2872, 1775, 1709, 1662, 1628, 1498, 1387; $^1$H NMR ($CDCl_3$, 400 MHz): $\delta$ 0.91 (t, $J$ = 7.2 Hz, 3H), 1.29-1.49 (m, 2H), 1.78-1.87 (m, 1H), 1.94-2.11 (m, 3H), 2.43-2.62 (m, 4H), 2.93 (ddd, $J$ = 12.8, 10.0, 7.2 Hz, 1H), 3.15 (d, $J$ = 2.8 Hz, 1H), 3.27 (dd, $J$ = 10.2, 6.8 Hz, 1H), 3.35 (dd, $J$ = 10.2, 7.2 Hz, 1H), 5.54 (dd, $J$ = 6.8, 2.8 Hz, 1H), 7.26-7.30 (m, 2H), 7.41 (tt, $J$ = 7.2, 1.2 Hz, 1H), 7.47-7.51 (m, 2H); $^{13}$C NMR ($CDCl_3$, 100 MHz): $\delta$ 14.3 ($CH_3$), 21.8 ($CH_2$), 22.1 ($CH_2$), 32.4 ($CH_2$), 34.0 ($CH_2$), 37.4 ($CH_2$), 41.1 (CH), 43.2 (CH), 45.3 (CH), 58.4 (CH), 126.5 (CH), 128.8 (CH), 129.2 (CH), 131.7 (C), 133.6 (C), 166.3 (C), 176.2 (CO), 176.7 (CO), 196.7 (CO); HRMS (ESI) m/z: [M + Na]$^+$ calcd. for $C_{21}H_{23}NO_4Na$ 376.1519, found 376.1524.

**(3a*R**,4*R**,7*R**,7a*R**)-6-Butyl-4-hydroxy-1,3-dioxo-2-phenyl-2,3,3a,4,7,7a-hexahydro-1*H*-4,7-ethanoisoindole-5-carbaldehyde (31a).** Ketone-type cyclic adduct **31a** (28 mg, 8% yield over 2 steps) was also isolated as a white solid: mp = 102–104 °C; IR (KBr, $cm^{-1}$) $\nu_{max}$ 3465 (br), 2957, 2930, 2872, 1774, 1710, 1651, 1597, 1498, 1380; $^1H$ NMR ($CDCl_3$, 400 MHz): $\delta$ 0.88 (t, $J$ = 7.2 Hz, 3H), 1.25-1.39 (m, 2H), 1.41-1.53 (m, 2H), 1.58-1.66 (m, 1H), 1.70-1.78 (m, 1H), 1.87-1.97 (m, 2H), 2.36 (ddd, $J$ = 12.8, 8.4, 6.4 Hz, 1H), 2.81 (ddd, $J$ = 12.8, 8.8, 7.2 Hz, 1H), 3.08 (d, $J$ = 8.4 Hz, 1H), 3.23-3.26 (m, 2H), 5.85 (br s, 1H), 7.08-7.11 (m, 2H), 7.37 (tt, $J$ = 7.2, 1.2 Hz, 1H), 7.41-7.45 (m, 2H), 10.02 (s, 1H); $^{13}C$ NMR ($CDCl_3$, 100 MHz): $\delta$ 13.6 ($CH_3$), 22.6 ($CH_2$), 23.7 ($CH_2$), 30.0 ($CH_2$), 30.6 ($CH_2$), 32.9 ($CH_2$), 38.6 (CH), 44.4 (CH), 49.3 (CH), 75.6 (C), 126.0 (CH), 128.7 (CH), 129.1 (CH), 131.2 (C), 134.8 (C), 163.5 (C), 175.5 (CO), 176.0 (CO), 189.1 (CHO); HRMS (ESI) m/z: $[M + Na]^+$ calcd. for $C_{21}H_{23}NO_4Na$ 376.1519, found 376.1517.

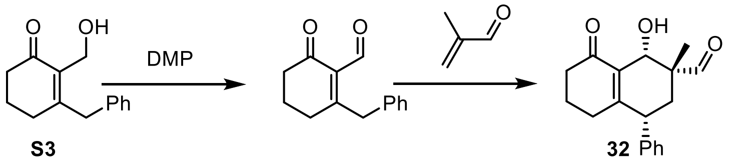


S3 32

**(1*R**,2*R**,4*R**)-1-Hydroxy-2-methyl-8-oxo-4-phenyl-1,2,3,4,5,6,7,8-octahydronaphthalene-2-carbaldehyde (32).** Following the general procedure of condition B, compound **32** (264 mg, 93% yield over 2 steps) was prepared from 2-(hydroxymethyl) enone **S3** (216 mg, 1.0 mmol) and methacrolein (84 mg, 1.2 mmol) as a white solid: mp = 158–160 °C; IR (KBr, $cm^{-1}$) $\nu_{max}$ 3461 (br), 3082, 3059, 3025, 2932, 2874, 2807, 2713, 1725, 1655, 1627, 1600, 1493, 1455; $^1H$ NMR ($CDCl_3$, 400 MHz): $\delta$ 1.04 (s, 3H), 1.79-1.94 (m, 3H), 1.98-2.11 (m, 2H), 2.26 (dd, $J$ = 13.6, 11.6 Hz, 1H), 2.39 (ddd, $J$ = 17.2, 10.8, 5.6 Hz, 1H), 2.49 (dt, $J$ = 17.2, 5.2 Hz, 1H), 3.17 (br s, 1H), 3.45 (dd, $J$ = 11.6, 6.4 Hz, 1H), 4.81 (s, 1H), 7.19-7.21 (m, 2H), 7.24-7.28 (m, 1H), 7.31-7.34 (m, 2H), 9.68 (s, 1H); $^{13}C$ NMR ($CDCl_3$, 100 MHz): $\delta$ 16.4 ($CH_3$), 22.1 ($CH_2$), 29.4 ($CH_2$), 31.9 ($CH_2$), 37.6 ($CH_2$), 45.6 (CH), 48.3 (C), 65.2 (CH), 127.0 (CH), 128.5 (CH), 128.8 (CH), 133.0 (C), 142.1 (C), 161.3 (C), 199.8 (CO), 204.1 (CHO); HRMS (ESI) m/z: $[M + Na]^+$ calcd. for $C_{18}H_{20}O_3Na$ 307.1305, found 307.1307.

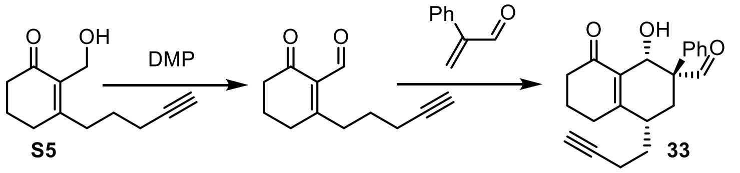


S5 33

**(1*R**,2*S**,4*S**)-4-(But-3-yn-1-yl)-1-hydroxy-8-oxo-2-phenyl-1,2,3,4,5,6,7,8-octahydronaphthalene-2-carbaldehyde (33).** Following the general procedure of condition B, compound **33** (261 mg, 81% yield over 2 steps) was prepared from 2-(hydroxymethyl) enone **S5** (192 mg, 1.0 mmol) and 2-phenylpropenal (159 mg, 1.2 mmol) as a white solid: mp = 103–105 °C; IR

(KBr, $cm^{-1}$) $\nu_{max}$ 3444, 3289, 3088, 3058, 2929, 2869, 2825, 2719, 2116, 1724, 1660, 1622, 1495, 1448; $^{1}$H NMR ($CDCl_3$, 400 MHz): $\delta$ 1.50-1.59 (m, 1H), 1.70-1.82 (m, 1H), 1.92 (t, $J$ = 2.8 Hz, 1H), 1.95-2.14 (m, 5H), 2.33-2.58 (m, 6H), 2.98 (br s, 1H), 5.62 (s, 1H), 7.10-7.13 (m, 2H), 7.24-7.34 (m, 3H), 9.51 (s, 1H); $^{13}$C NMR ($CDCl_3$, 100 MHz): $\delta$ 15.6 ($CH_2$), 22.2 ($CH_2$), 26.6 ($CH_2$), 28.6 ($CH_2$), 30.2 ($CH_2$), 36.6 (CH), 37.3 ($CH_2$), 56.8 (C), 62.2 (CH), 69.4 (C), 82.8 (C), 127.5 (CH), 127.7 (CH), 128.9 (CH), 132.9 (C), 134.5 (C), 164.0 (C), 198.8 (CHO), 199.6 (CO); HRMS (ESI) m/z: $[M + Na]^+$ calcd. for $C_{21}H_{22}O_3Na$ 345.1461, found 345.1461.

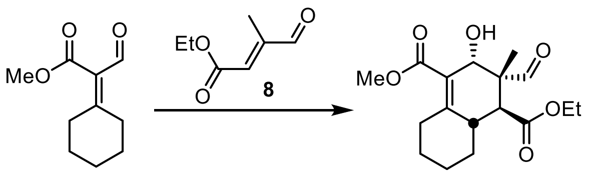


S35 34

**1-Ethyl 4-methyl (1*S**,2*R**,3*R**,8a*S**)-2-formyl-3-hydroxy-2-methyl-1,2,3,5,6,7,8,8a-octahydronaphthalene-1,4-dicarboxylate (34).** Following the general procedure of condition A, compound **34** (237 mg, 73% yield) was prepared from enone **S35** (182 mg, 1.0 mmol) and dienophile **8** (171 mg, 1.2 mmol) as a colorless oil: IR (KBr, $cm^{-1}$) $\nu_{max}$ 3484 (br), 2981, 2933, 2858, 1722, 1638; $^{1}$H NMR ($CDCl_3$, 400 MHz): $\delta$ 0.95 (s, 3H), 1.20-1.31 (m, 1H), 1.25 (t, $J$ = 7.2 Hz, 3H), 1.36-1.60 (m, 2H), 1.76-1.94 (m, 3H), 2.07-2.12 (m, 1H), 2.55 (ddd, $J$ = 12.4, 10.0, 4.8 Hz, 1H), 2.73 (br s, 1H), 3.16 (d, $J$ = 10.0 Hz, 1H), 3.48-3.53 (m, 1H), 3.79 (s, 3H), 4.11-4.23 (m, 2H), 4.36 (s, 1H), 9.82 (s, 1H); $^{13}$C NMR ($CDCl_3$, 100 MHz): $\delta$ 13.0 ($CH_3$), 14.0 ($CH_3$), 25.5 ($CH_2$), 27.2 ($CH_2$), 31.0 ($CH_2$), 34.5 ($CH_2$), 39.2 (CH), 44.9 (CH), 50.2 (C), 51.7 ($CH_3$), 60.9 ($CH_2$), 71.6 (CH), 122.6 (C), 155.0 (C), 168.3 (CO), 173.2 (CO), 205.2 (CHO); HRMS (ESI) m/z: $[M + Na]^+$ calcd. for $C_{17}H_{24}O_6Na$ 347.1465, found 347.1466.

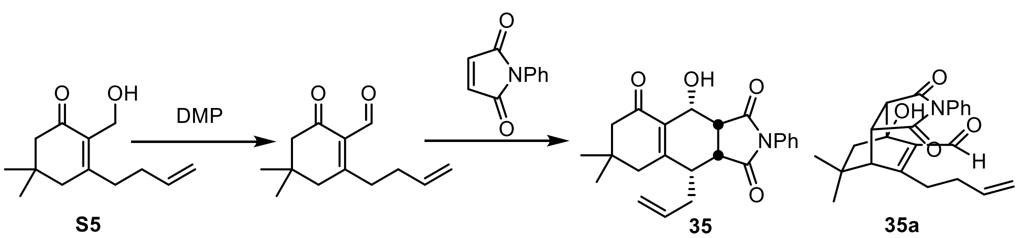


S5 35 35a

**(3a*R**,4*R**,9*S**,9a*R**)-9-Allyl-4-hydroxy-7,7-dimethyl-2-phenyl-4,6,7,8,9,9a-hexahydro-1*H*-benzo[*f*]isoindole-1,3,5(2*H*,3a*H*)-trione (35).** Following the general procedure of condition B, compound **35** (300 mg, 79% yield over 2 steps) was prepared from 2-(hydroxymethyl) enone **S5** (208 mg, 1.0 mmol) and *N*-phenylmaleimide (208 mg, 1.2 mmol) as a white solid: mp = 204–206 °C; IR (KBr, $cm^{-1}$) $\nu_{max}$ 3440 (br), 3071, 2957, 2923, 2852, 1778, 1708, 1665, 1630, 1498, 1386; $^{1}$H NMR ($CDCl_3$, 400 MHz): $\delta$ 1.04 (s, 3H), 1.06 (s, 3H),

2.32 (d, $J$ = 16.0 Hz, 1H), 2.33 (d, $J$ = 16.0 Hz, 1H), 2.34 (d, $J$ = 18.8 Hz, 1H), 2.48 (d, $J$ = 18.8 Hz, 1H), 2.70 (dt, $J$ = 13.2, 6.0 Hz, 1H), 2.79 (dt, $J$ = 13.2, 8.4 Hz, 1H), 2.93 (ddd, $J$ = 8.4, 6.8, 6.0 Hz, 1H), 3.19 (d, $J$ = 4.0 Hz, 1H), 3.29 (dd, $J$ = 10.4, 6.4 Hz, 1H), 3.36 (dd, $J$ = 10.4, 6.8 Hz, 1H), 5.05 (d, $J$ = 10.0 Hz, 1H), 5.11 (d, $J$ = 17.2 Hz, 1H), 5.52 (dd, $J$ = 6.4, 4.0 Hz, 1H), 5.75-5.86 (m, 1H), 7.26-7.30 (m, 2H), 7.39-7.43 (m, 1H), 7.46-7.51 (m, 2H); $^{13}$C NMR ($CDCl_3$, 100 MHz): $\delta$ 27.7 ($CH_3$), 29.1 ($CH_3$), 33.5 (C), 35.9 ($CH_2$), 40.9 (CH), 42.7 (CH), 45.5 (CH), 46.0 ($CH_2$), 51.0 ($CH_2$), 58.7 (CH), 118.0 ($CH_2$), 126.5 (CH), 128.8 (CH), 129.2 (CH), 131.6 (C), 132.8 (C), 135.8 (CH), 162.9 (C), 175.9 (CO), 176.5 (CO), 197.1 (CO); HRMS (ESI) m/z: $[M + Na]^+$ calcd. for $C_{23}H_{25}NO_4Na$ 402.1676, found 402.1676.

**(3a*R**,4*R**,7*S**,7a*R**)-6-(But-3-en-1-yl)-4-hydroxy-8,8-dimethyl-1,3-dioxo-2-phenyl-2,3,3a,4,7,7a-hexahydro-1*H*-4,7-ethanoisoindole-5-carbaldehyde (35a).** Ketone-type cyclic adduct **35a** (38 mg, 10% yield over 2 steps) was also isolated as a white solid: mp = 72–75 °C; IR (KBr, cm$^{-1}$) $\nu_{max}$ 3470 (br), 3361, 3068, 2959, 2920, 2850, 1774, 1711, 1654, 1597, 1498, 1385; $^1$H NMR ($CDCl_3$, 400 MHz): $\delta$ 1.02 (s, 3H), 1.25 (s, 3H), 1.59 (d, $J$ = 12.8 Hz, 1H), 1.66 (d, $J$ = 12.8 Hz, 1H), 2.10-2.20 (m, 1H), 2.22-2.32 (m, 1H), 2.58-2.72 (m, 2H), 2.87 (d, $J$ = 3.6 Hz, 1H), 3.00 (d, $J$ = 8.4 Hz, 1H), 3.55 (dd, $J$ = 8.4, 3.6 Hz, 1H), 4.99 (dq, $J$ = 10.4, 1.2 Hz, 1H), 5.03 (dq, $J$ = 17.2, 1.6 Hz, 1H), 5.63 (br s, 1H), 5.75 (ddt, $J$ = 17.2, 10.4, 6.4 Hz, 1H), 7.07-7.10 (m, 2H), 7.37 (tt, $J$ = 7.2, 1.6 Hz, 1H), 7.41-7.46 (m, 2H), 10.05 (s, 1H); $^{13}$C NMR ($CDCl_3$, 100 MHz): $\delta$ 29.3 ($CH_3$), 31.2 ($CH_3$), 31.5 ($CH_2$), 31.8 ($CH_2$), 34.1 (C), 41.8 (CH), 48.2 (CH), 48.2 ($CH_2$), 50.1 (CH), 76.4 (C), 116.1 ($CH_2$), 126.1 (CH), 128.8 (CH), 129.2 (CH), 131.2 (C), 134.0 (C), 136.4 (CH), 162.9 (C), 175.5 (CO), 176.8 (CO), 189.5 (CHO); HRMS (ESI) m/z: $[M + Na]^+$ calcd. for $C_{23}H_{25}NO_4Na$ 402.1676, found 402.1678.

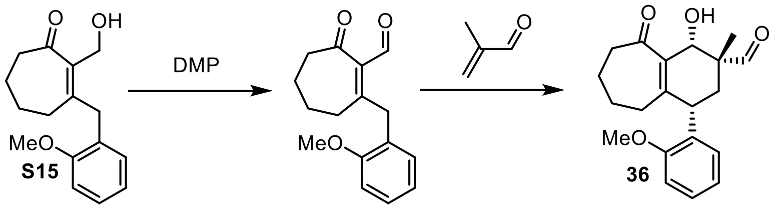


**(1*R**,2*R**,4*S**)-1-Hydroxy-4-(2-methoxyphenyl)-2-methyl-9-oxo-2,3,4,5,6,7,8,9-octahydro-1*H*-benzo[7]annulene-2-carbaldehyde (36).** Following the general procedure of condition B, compound **36** (253 mg, 77% yield over 2 steps) was prepared from 2-(hydroxymethyl) enone **S15** (260 mg, 1.0 mmol) and 2-phenylpropenal (159 mg, 1.2 mmol) as a white solid: mp = 120–123 °C; IR (KBr, cm$^{-1}$) $\nu_{max}$ 3449 (br), 3061, 2937, 2867, 2837, 2713, 1726, 1654, 1624, 1598, 1493, 1460; $^1$H NMR ($CDCl_3$, 400 MHz): $\delta$ 1.04 (s, 3H), 1.40-1.58 (m, 2H), 1.75 (quint, $J$ = 6.8 Hz, 2H), 1.80-1.89 (m, 1H), 2.09-2.25 (m, 3H), 2.57 (dt, $J$ = 15.2, 6.0 Hz, 1H), 2.65 (dt, $J$ = 15.2, 6.8 Hz, 1H), 2.72 (br s, 1H), 3.83 (s, 3H), 3.98 (br s, 1H), 4.81 (d, $J$ = 4.4 Hz, 1H), 6.90 (d, $J$ = 8.0 Hz, 1H), 6.94 (t, $J$ = 7.6 Hz, 1H), 7.12 (d, $J$ = 6.4 Hz, 1H), 7.25 (t, $J$ = 7.6 Hz, 1H); $^{13}$C NMR ($CDCl_3$, 100 MHz): $\delta$ 16.3 ($CH_3$), 20.7 ($CH_2$), 24.4 ($CH_2$), 30.9 (br,

$CH_2$), 38.5 (br, C), 41.5 ($CH_2$), 48.6 (C), 55.2 ($CH_3$), 67.5 (CH), 110.7 (br, CH), 121.0 (CH), 128.1 (br, CH), 157.0 (C), 204.7 (CHO), 205.8 (CO); HRMS (ESI) m/z: $[M + Na]^+$ calcd. for $C_{20}H_{24}O_4Na$ 351.1567, found 351.1564.

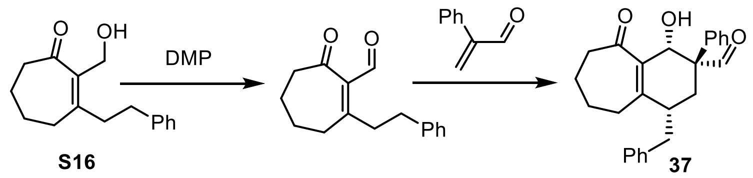


**(1*R**,2*S**,4*S**)-4-Benzyl-1-hydroxy-9-oxo-2-phenyl-2,3,4,5,6,7,8,9-octahydro-1*H*-benzo[7]annulene-2-carbaldehyde (37).** Following the general procedure of condition B, compound **37** (333 mg, 89% yield over 2 steps) was prepared from 2-(hydroxymethyl) enone **S16** (244 mg, 1.0 mmol) and 2-phenylpropenal (159 mg, 1.2 mmol) as a colorless oil: IR ($CH_2Cl_2$ cast, $cm^{-1}$) $\nu_{max}$ 3360 (br), 3026, 2919, 2850, 1723, 1652, 1634, 1494, 1470, 1454; $^1H$ NMR ($CDCl_3$, 400 MHz): $\delta$ 1.37-1.47 (m, 1H), 1.74-1.88 (m, 3H), 2.01-2.19 (m, 3H), 2.34-2.58 (m, 4H), 2.61 (dt, $J$ = 14.8, 5.2 Hz, 1H), 2.75 (dt, $J$ = 14.8, 6.0 Hz, 1H), 3.26 (dd, $J$ = 17.6, 9.0 Hz, 1H), 5.74 (d, $J$ = 3.6 Hz, 1H), 7.02-7.05 (m, 2H), 7.16-7.18 (m, 2H), 7.21-7.35 (m, 6H), 9.43 (s, 1H); $^{13}C$ NMR ($CDCl_3$, 100 MHz): $\delta$ 21.1 ($CH_2$), 24.9 ($CH_2$), 27.6 ($CH_2$), 31.2 ($CH_2$), 39.2 ($CH_2$), 41.5 ($CH_2$), 41.9 (CH), 57.2 (C), 64.2 (CH), 126.4 (CH), 127.4 (CH), 127.5 (CH), 128.6 (CH), 128.8 (CH), 128.8 (CH), 134.8 (C), 136.6 (C), 139.2 (C), 160.6 (C), 199.4 (CHO), 205.1 (CO); HRMS (ESI) m/z: $[M + Na]^+$ calcd. for $C_{25}H_{26}O_3Na$ 397.1774, found 397.1773.

General procedure for ketone-type anionic Diels-Alder reaction:

Condition C:

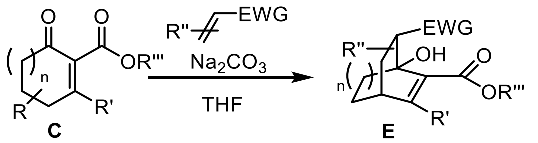


To a stirred solution of the cross-conjugated enone **C** (1.0 mmol) and $Na_2CO_3$ (127 mg, 1.2 mmol) in dry THF (5 mL), which was previously degassed with argon, was added dienophile (1.2 mmol) in one portion at room temperature. The resulting mixture was then stirred under individual temperature and time as indicated in Table 3. After reaction was complete, the reaction mixture was quenched with sat. $NH_4Cl_{(aq)}$ and extracted with EtOAc (10 mL × 2). The combined organic extract was washed with water and brine, dried over $MgSO_4$, filtered and concentrated to give the crude residue, which was purified by chromatography on silica gel to afford anionic Diels-Alder adduct **E**, including compounds **38-41** as indicated below.

Condition D:

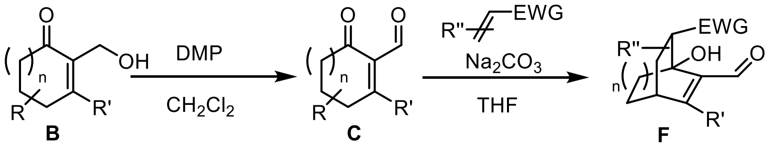


To a stirred solution of the 2-(hydroxymethyl) enone **B** (1.0 mmol) in dry $CH_2Cl_2$ (10 mL) was added Dess–Martin periodinane (551 mg, 1.3 mmol) in one portion at 0 °C. The resulting mixture was then stirred at room temperature under $N_2$ for 30 min. After reaction was complete, the reaction mixture was quenched with 5% $Na_2S_2O_{3(aq)}$ (20 mL). The resulting solution was then stirred at room temperature for 20 min. The aquous layer was extracted with $CH_2Cl_2$ (10 mL × 2). The organic portions were combined, washed with sat. $NaHCO_{3(aq)}$ and brine, dried over $MgSO_4$, filtered and concentrated to give the cross-conjugated enone **C** individually. To a stirred solution of the crude cross-conjugated enone **C** and $Na_2CO_3$ (127 mg, 1.2 mmol) in dry THF (5 mL), which was previously degassed with argon, was added dienophile (1.2 mmol) in one portion at room temperature. The resulting mixture was then stirred under individual temperature and time as indicated in Table 3. After reaction was complete, the reaction mixture was quenched with sat. $NH_4Cl_{(aq)}$ and extracted with EtOAc (10 mL × 2). The combined organic extract was washed with water and brine, dried over $MgSO_4$, filtered and concentrated to give the crude residue, which was purified by chromatography on silica gel to afford anionic Diels-Alder adduct **F**, including compounds **42-46** as indicated below.

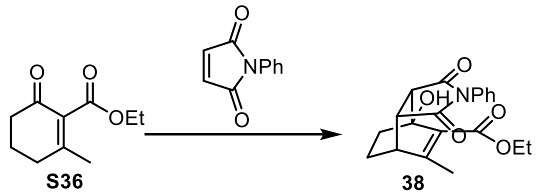


**Ethyl (3a*R**,4*R**,7*R**,7a*R**)-4-hydroxy-6-methyl-1,3-dioxo-2-phenyl-2,3,3a,4,7,7a-hexahydro-1*H*-4,7-ethanoisoindole-5-carboxylate (38).** Following the general procedure of condition C, compound **38** (270 mg, 76% yield) was prepared from enone **S36** (182 mg, 1.0 mmol) and *N*-phenylmaleimide (208 mg, 1.2 mmol) as a white solid: mp = 111–113 °C; IR (KBr, $cm^{-1}$) $\nu_{max}$ 3057 (br), 3361, 3196, 3067, 2919, 2875, 2850, 1775, 1710, 1634, 1598, 1499, 1382; $^{1}H$ NMR ($CDCl_3$, 600 MHz): $\delta$ 1.29 (t, *J* = 7.2 Hz, 3H), 1.65-1.74 (m, 2H), 1.77-1.90 (m, 2H), 2.04 (s, 3H), 3.02 (d, *J* = 8.4 Hz, 1H), 3.09-3.11 (m, 1H), 3.22 (dd, *J* = 8.4, 3.6 Hz, 1H), 4.22-4.32 (m, 2H), 4.88 (s, 1H), 7.15 (d, *J* = 7.2 Hz, 2H), 7.37 (t, *J* = 7.2 Hz, 1H), 7.44 (t, *J* = 7.2 Hz, 2H); $^{13}C$ NMR ($CDCl_3$, 150 MHz): $\delta$ 14.2 ($CH_3$), 19.8 ($CH_3$), 22.5 ($CH_2$), 32.7 ($CH_2$), 39.0 (CH), 44.9 (CH), 49.3 (CH), 61.1 ($CH_2$), 74.4 (C), 126.4 (CH), 128.7 (CH), 129.1 (CH), 130.5 (C), 131.5 (C), 147.3 (C), 165.2 (CO), 175.8 (CO), 176.4 (CO); HRMS (ESI) m/z: $[M + Na]^+$ calcd. for $C_{20}H_{21}NO_5Na$ 378.1312, found 378.1307.

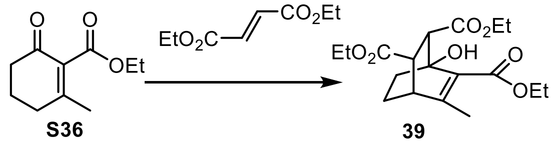


**Triethyl (1*R**,2*S**,3*R**,4*R**)-4-hydroxy-6-methylbicyclo[2.2.2]oct-5-ene-2,3,5-tricarboxylate (39).** Following the general procedure of condition C, compound **39** (252 mg, 71% yield) was prepared from enone **S36** (182 mg, 1.0 mmol) and diethyl fumarate (207 mg, 1.2 mmol) as a colorless oil: IR ($CH_2Cl_2$ cast, $cm^{-1}$) $\nu_{max}$ 3362 (br), 2981, 2960, 2938, 2919,, 2876, 2850, 1731, 1675, 1633; $^{1}H$ NMR ($CDCl_3$, 400 MHz): $\delta$ 1.27 (t, *J* = 7.2 Hz, 3H), 1.28 (t, *J* = 7.2 Hz, 3H), 1.34 (t, *J* = 7.2 Hz, 3H), 1.35-1.44 (m, 1H), 1.56 (td, *J* = 12.0, 4.0 Hz, 1H), 1.66-1.74 (m, 1H), 1.82 (ddd, *J* = 12.0, 10.0, 4.4 Hz, 1H), 2.22 (s, 3H), 2.79 (q, *J* = 2.8 Hz, 1H), 2.90 (ddd, *J* = 5.6, 2.8, 2.0 Hz, 1H), 3.26 (d, *J* = 5.6 Hz, 1H), 4.12-4.31 (m, 6H), 5.69 (s, 1H); $^{13}C$ NMR ($CDCl_3$, 150 MHz): $\delta$ 14.2 ($CH_3$), 14.2 ($CH_3$), 14.2 ($CH_3$), 19.7 ($CH_3$), 20.3 ($CH_2$), 33.7 ($CH_2$), 40.7 (CH), 46.0 (CH), 51.8 (CH), 60.8 ($CH_2$), 61.1 ($CH_2$), 61.2 ($CH_2$), 74.3 (C), 128.5 (C), 151.9 (C), 166.8 (CO), 172.3 (CO), 173.0 (CO); HRMS (ESI) m/z: $[M + Na]^+$ calcd. for $C_{18}H_{26}O_7Na$ 377.1571, found 377.1564.

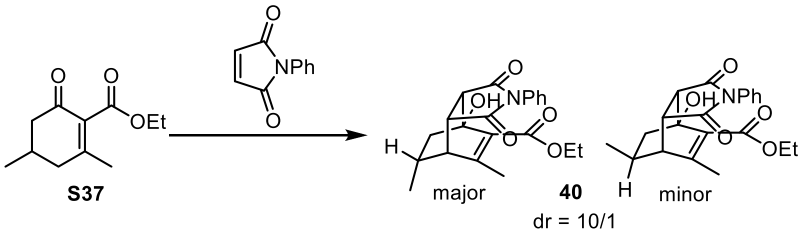


**Ethyl (3a*R**,4*R**,7*R**,7a*R**,9*R**)-7-hydroxy-5,9-dimethyl-1,3-dioxo-2-phenyl-2,3,3a,4,7,7a-hexahydro-1*H*-4,7-ethanoisoindole-6-carboxylate (40).** Following the general procedure of condition C, compound **40** (292 mg, 79% yield) was prepared from enone **S37** (196 mg, 1.0 mmol) and *N*-phenylmaleimide (208 mg, 1.2 mmol) as a white solid: mp = 129–132 °C; IR (KBr, $cm^{-1}$) $\nu_{max}$ 3462 (br), 3063, 2938, 2857, 1773, 1708, 1631, 1597, 1498, 1387; $^{1}$H NMR ($CDCl_3$, 600 MHz) mixture of diastereomers: $\delta$ 0.97 (d, $J$ = 6.0 Hz, 3H), 1.17 (d, $J$ = 6.0 Hz, 0.3H), 1.24-1.27 (m, 1.1 H), 1.29 (t, $J$ = 7.2 Hz, 3.3 H), 2.04 (s, 0.3 H), 2.05 (s, 3H), 2.08-2.11 (m, 2.2H), 2.83-2.85 (m, 0.1H), 2.94-2.95 (m, 1H), 2.97 (d, $J$ = 8.4 Hz, 1H), 2.99 (d, $J$ = 8.4 Hz, 0.1H), 3.23 (dd, $J$ = 8.4, 3.6 Hz, 1H), 3.42 (dd, $J$ = 8.4, 3.0 Hz, 0.1H), 4.21-4.32 (m, 2.2H), 4.86 (br s, 1H), 7.14 (d, $J$ = 7.2 Hz, 2H), 7.37 (t, $J$ = 7.2 Hz, 1.1H), 7.43 (t, $J$ = 7.2 Hz, 2.2H); $^{13}$C NMR($CDCl_3$, 150 MHz) major: $\delta$ 14.2 ($CH_3$), 21.6 ($CH_3$), 21.6 ($CH_3$), 30.3 (CH), 41.5 ($CH_2$), 45.3 (CH), 45.6 (CH), 48.5 (CH), 61.1 ($CH_2$), 74.6 (C), 126.3 (CH), 128.7 (CH), 129.1 (CH), 130.0 (C), 131.6 (C), 146.3 (C), 165.2 (CO), 175.7 (CO), 176.3 (CO); HRMS (ESI) m/z: $[M + Na]^+$ calcd. for $C_{21}H_{23}NO_5Na$ 392.1468, found 392.1471.

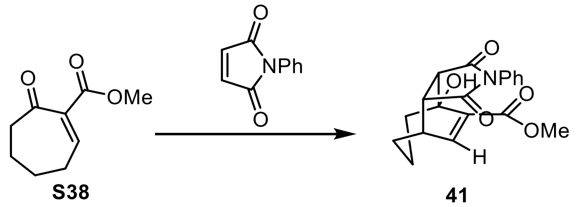


**Methyl (3a*R**,8*R**,8a*R**)-8-hydroxy-1,3-dioxo-2-phenyl-1,2,3,3a,4,5,6,7,8,8a-decahydro-4,8-ethenocyclohepta[*c*]pyrrole-9-carboxylate (41).** Following the general procedure of condition C, compound **41** (321 mg, 94% yield) was prepared from enone **S38** (168 mg, 1.0 mmol) and *N*-phenylmaleimide (208 mg, 1.2 mmol) as a white solid: mp = 161–163 °C; IR (KBr, $cm^{-1}$) $\nu_{max}$ 3462 (br), 3063, 2938, 2857, 1773, 1708, 1631, 1597, 1498, 1387; $^{1}$H NMR ($CDCl_3$, 400 MHz): $\delta$ 1.67-1.94 (m, 5H), 2.06-2.13 (m, 1H), 3.25-3.29 (m, 1H), 3.28 (dd, $J$ = 8.8, 1.6 Hz, 1H), 3.41 (d, $J$ = 8.8 Hz, 1H), 3.78 (s, 3H), 5.20 (s, 1H), 6.78 (d, $J$ = 8.0 Hz, 1H), 7.19-7.22 (m, 2H), 7.39 (tt, $J$ = 7.2, 1.2 Hz, 1H), 7.43-7.48 (m, 2H); $^{13}$C NMR ($CDCl_3$, 150 MHz): $\delta$ 21.7 ($CH_2$), 26.3 ($CH_2$), 35.0 (CH), 38.6 ($CH_2$), 44.2 (CH), 50.1 (CH), 52.2 ($CH_3$), 73.5 (C),

126.4 (C), 128.8 (CH), 129.1 (CH), 131.4 (C), 137.5 (C), 139.6 (CH), 166.2 (CO), 176.9 (CO), 177.4 (CO); HRMS (ESI) m/z: $[M + Na]^+$ calcd. for $C_{19}H_{19}NO_5Na$ 364.1155, found 364.1156.

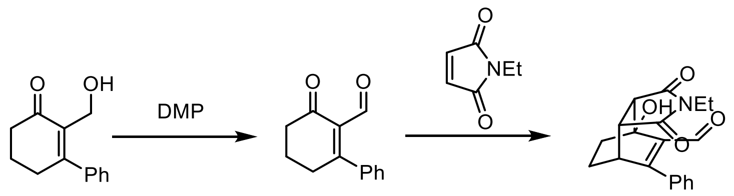


S6 42

**(3a*R**,4*R**,7*R**,7a*S**)-2-Ethyl-4-hydroxy-1,3-dioxo-6-phenyl-2,3,3a,4,7,7a-hexahydro-1*H*-4,7-ethanoisoindole-5-carbaldehyde (42).** Following the general procedure of condition D, compound adduct **42** (273 mg, 84% yield over 2 steps) was prepared from 2-(hydroxymethyl) enone **S6** (202 mg, 1.0 mmol) and *N*-ethylmaleimide (150 mg, 1.2 mmol) as a white solid: mp = 170–172 °C; IR (KBr, $cm^{-1}$) $\nu_{max}$ 3446 (br), 2919, 2850, 1770, 1697, 1651, 1568; $^1H$ NMR ($CDCl_3$, 400 MHz): $\delta$ 0.94 (t, $J$ = 7.2 Hz, 3H), 1.68-1.84 (m ,2H), 1.91-2.02 (m, 2H), 3.03 (d, $J$ = 8.4 Hz, 1H), 3.17 (dd, $J$ = 8.4, 3.2 Hz, 1H), 3.42-3.51 (m, 2H), 3.54 (q, $J$ = 3.2 Hz, 1H), 6.22 (s, 1H), 7.29-7.32 (m, 2H), 7.41-7.48 (m, 3H), 9.54 (s, 1H); $^{13}C$ NMR ($CDCl_3$, 150 MHz): $\delta$ 13.2 ($CH_3$), 23.3 ($CH_2$), 32.5 ($CH_2$), 33.5 ($CH_2$), 40.1 (CH), 44.8 (CH), 49.8 (CH), 76.1 (C), 128.8 (CH), 129.4 (CH), 130.2 (CH), 133.7 (C), 135.1 (C), 161.4 (C), 175.7 (CO), 177.2 (CO), 191.5 (CHO); HRMS (ESI) m/z: $[M + Na]^+$ calcd. for $C_{19}H_{19}NO_4Na$ 348.1206, found 348.1208.

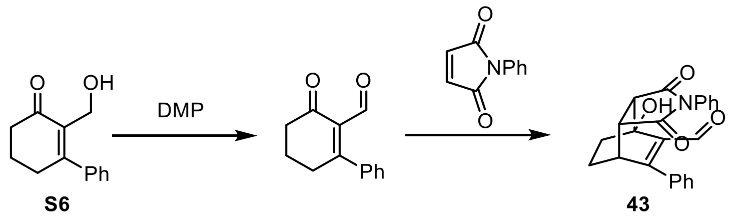


S6 43

**(3a*R**,4*R**,7*R**,7a*R**)-4-hydroxy-1,3-dioxo-2,6-diphenyl-2,3,3a,4,7,7a-hexahydro-1*H*-4,7-ethanoisoindole-5-carbaldehyde (43).** Following the general procedure of condition D, compound **43** (347 mg, 93% yield over 2 steps) was prepared from 2-(hydroxymethyl) enone **S6** (202 mg, 1.0 mmol) and *N*-phenylmaleimide (208 mg, 1.2 mmol) as a white solid: mp = 178–180 °C; IR (KBr, $cm^{-1}$) $\nu_{max}$ 3464 (br), 3061, 2917, 2874, 2850, 1774, 1711, 1648, 1597, 1498, 1383; $^1H$ NMR ($CDCl_3$, 400 MHz): $\delta$ 1.73-1.90 (m, 2H), 1.96-2.10 (m, 2H), 3.22 (d, $J$ = 8.8 Hz, 1H), 3.37 (dd, $J$ = 8.8, 3.2 Hz, 1H), 3.64 (q, $J$ = 3.2 Hz, 1H), 6.25 (s, 1H), 7.05-7.08 (m, 2H), 7.30-7.48 (m, 8H), 9.64 (s, 1H); $^{13}C$ NMR ($CDCl_3$, 100 MHz): $\delta$ 23.4 ($CH_2$), 32.6 ($CH_2$), 40.3 (CH), 44.9 (CH), 49.9 (CH), 76.2 (C), 126.0 (CH), 128.7 (CH), 128.9 (CH), 129.1 (CH), 129.4 (CH), 130.3 (CH), 131.2 (C), 133.7 (C), 135.4 (C), 161.4 (C), 174.9 (CO), 176.3 (CO), 191.6 (CO); HRMS (ESI) m/z: $[M + Na]^+$ calcd. for $C_{23}H_{19}NO_4$ 396.1206, found 396.1207.

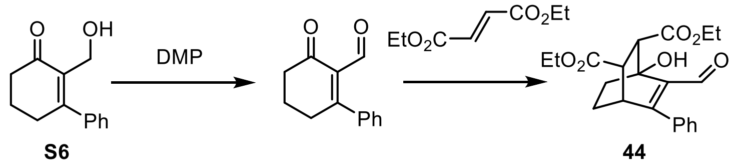


**Diethyl (1*R**,2*R**,3*S**,4*R**)-6-formyl-1-hydroxy-5-phenylbicyclo[2.2.2]oct-5-ene-2,3-dicarboxylate (44).** Following the general procedure of condition D, compound **44** (272 mg, 73% yield over 2 steps) was prepared from 2-(hydroxymethyl) enone **S6** (202 mg, 1.0 mmol) and diethyl fumarate (207 mg, 1.2 mmol) as a colorless oil: IR ($CH_2Cl_2$ cast, $cm^{-1}$) $\nu_{max}$ 3401 (br), 3058, 1980, 2940, 2875, 1732, 1650, 1594; $^1$H NMR ($CDCl_3$, 400 MHz): $\delta$ 1.27 (t, *J* = 7.2 Hz, 3H), 1.28 (t, *J* = 7.2 Hz, 3H), 1.59-1.74 (m, 2H), 1.90-1.99 (m, 2H), 2.93 (dt, *J* = 5.6, 2.8 Hz, 1H), 3.31 (q, *J* = 2.8 Hz, 1H), 3.37 (d, *J* = 5.6 Hz, 1H), 4.09-4.24 (m, 4H), 6.59 (s, 1H), 7.36-7.39 (m, 2H), 7.45-7.48 (m, 3H), 9.62 (s, 1H); $^{13}$C NMR ($CDCl_3$, 150 MHz): $\delta$ 14.2 ($CH_3$),14.2 ($CH_3$), 21.0 ($CH_2$), 33.6 ($CH_2$), 40.9 (CH), 46.8 (CH), 51.9 (CH), 61.1 ($CH_2$), 61.4 ($CH_2$), 76.5 (C), 128.6 (CH), 129.1 (CH), 129.8 (CH), 134.7 (C), 135.9 (C), 162.4 (C), 171.7 (CO), 173.4 (CO), 193.0 (CO); HRMS (ESI) m/z: [M + Na]$^+$ calcd. for $C_{21}H_{24}O_6Na$ 395.1465, found 395.1464.

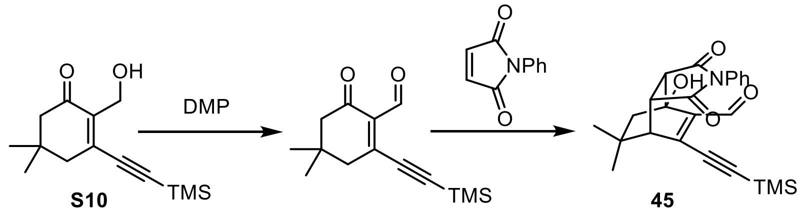


**(3a*R**,4*R**,7*S**,7a*R**)-4-Hydroxy-8,8-dimethyl-1,3-dioxo-2-phenyl-6-((trimethylsilyl)ethynyl)-2,3,3a,4,7,7a-hexahydro-1*H*-4,7-ethanoisoindole-5-carbaldehyde (45).** Following the general procedure of condition D, compound **45** (400 mg, 95% yield over 2 steps) was prepared from 2-(hydroxymethyl) enone **S10** (250 mg, 1.0 mmol) and *N*-phenylmaleimide (208 mg, 1.2 mmol) as a white solid: mp = 135–137 °C; IR (KBr, $cm^{-1}$) $\nu_{max}$ 3478 (br), 3065, 2962, 2925, 2871, 2851, 2139, 1778, 1714, 1662, 1598, 1570, 1500, 1385; $^1$H NMR ($CDCl_3$, 400 MHz): $\delta$ 0.23 (s, 9H), 1.09 (s, 3H), 1.24 (s, 3H), 1.63 (d, *J* = 12.8 Hz, 1H), 1.69 (d, *J* = 12.8, 1H), 3.05 (d, *J* = 3.2 Hz, 1H), 3.06 (d, *J* = 8.4 Hz, 1H), 3.53 (dd, *J* = 8.4, 3.2 Hz, 1H), 5.73 (s, 1H), 7.10-7.13 (m, 2H), 7.35-7.45 (m, 3H), 10.06 (s, 1H); $^{13}$C NMR ($CDCl_3$, 150 MHz): $\delta$ 0.5 ($CH_3$), 28.9 ($CH_3$), 30.4 ($CH_3$), 34.2 (C), 41.3 (CH), 47.9 ($CH_2$), 48.9 (CH), 51.5 (CH), 76.5 (C), 98.1 (C), 111.5 (C), 126.2 (CH), 128.8 (CH), 129.1 (CH), 131.4 (C), 141.5 (C), 142.8 (C), 174.6 (CO), 175.8 (CO), 189.9 (CO); HRMS (ESI) m/z: [M + Na]$^+$ calcd. for $C_{24}H_{27}NO_4SiNa$ 444.1602, found 444.1604.

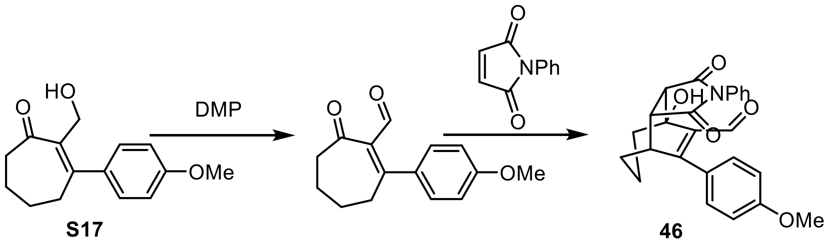

S17 46

**(3a*R**,8*R**,8a*R**)-8-hydroxy-10-(4-methoxyphenyl)-1,3-dioxo-2-phenyl-1,2,3,3a,4,5,6,7,8, 8a-decahydro-4,8-ethenocyclohepta[*c*]pyrrole-9-carbaldehyde (46).** Following the general procedure of condition D, product **46** (376 mg, 90% yield over 2 steps) was prepared from 2-(hydroxymethyl) enone **S17** (246 mg, 1.0 mmol) and *N*-phenylmaleimide (208 mg, 1.2 mmol) as a white solid: mp = 167–169 °C; IR (KBr, $cm^{-1}$) $\nu_{max}$ 3502 (br), 3065, 2922, 2851, 1774, 1709, 1647, 1602, 1509, 1386; $^1$H NMR ($CDCl_3$, 400 MHz): $\delta$ 1.74-1.83 (m, 3H), 1.85-1.96 (m, 1H), 2.02-2.09 (m, 1H), 2.14-2.21 (m, 1H), 3.40 (dd, $J$ = 8.8, 1.6 Hz, 1H), 3.52 (d, $J$ = 8.8 Hz, 1H), 3.57-3.59 (m, 1H), 3.83 (s, 3H), 6.91-6.94 (m, 2H), 7.13-7.16 (m, 2H), 7.19-7.22 (m, 2H), 7.35-7.45 (m, 3H), 9.53 (s, 1H); $^{13}$C NMR ($CDCl_3$, 100 MHz): $\delta$ 21.9 ($CH_2$), 26.2 ($CH_2$), 40.8 ($CH_2$), 43.7 (CH), 43.9 (CH), 50.3 (CH), 55.3 (CH), 75.6 (C), 114.1 (CH), 126.2 (CH), 127.6 (C), 128.7 (CH), 129.1 (CH), 130.7 (CH), 131.4 (C), 136.9 (C), 161.1 (C), 161.3 (C), 175.4 (CO), 177.4 (CO), 193.0 (CHO); HRMS (ESI) m/z: $[M + Na]^+$ calcd. for $C_{25}H_{23}NO_5Na$ 440.1468, found 440.1473.

**X-ray crystal structure of anionic Diels-Alder compounds**

The recrystallization of **9** was carried out by the vapor diffusion method: A 4-mL vial containing a solution of **9** (15 mg) in ehtyl acetate (2 mL) was placed into a 20 mL vial containing *n*-hexane (10 mL). The outer bottle was capped and allowed to stand at 4 ℃ for 3 days to afford the compound **9** crystals.

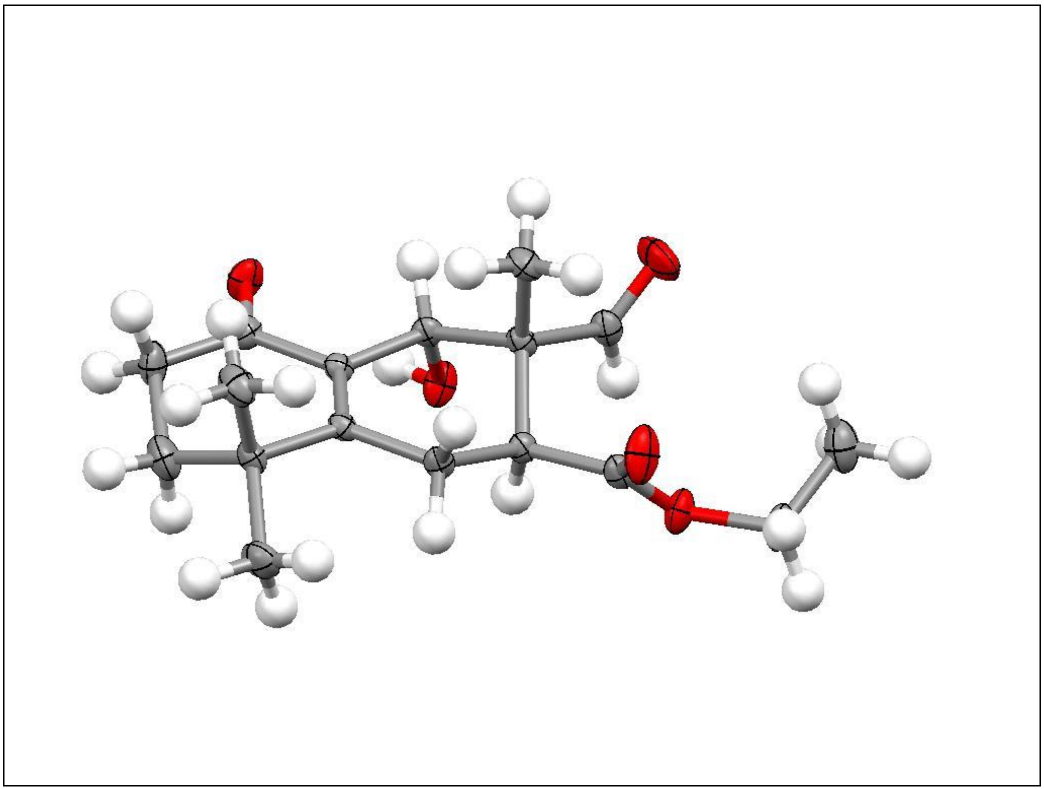

X-ray crystal structure of **9** (CCDC 2074048)
Thermal ellipsoid plot at 50% probability level.

Table S1. Crystal data and structure refinement for **9** (d22713).

| | | |
|---|---|---|
| Identification code | d22713 | |
| Empirical formula | $C_{17}H_{24}O_5$ | |
| Formula weight | 308.36 | |
| Temperature | 200(2) K | |
| Wavelength | 0.71073 Å | |
| Crystal system | Monoclinic | |
| Space group | P 21/c | |
| Unit cell dimensions | a = 7.7174(4) Å | a= 90°. |
| | b = 17.1948(6) Å | b= 102.4860(10)°. |
| | c = 12.3310(6) Å | g = 90°. |
| Volume | 1597.61(13) Å $^3$ | |
| Z | 4 | |
| Density (calculated) | 1.282 Mg/m$^3$ | |
| Absorption coefficient | 0.093 mm$^{-1}$ | |
| F(000) | 664 | |
| Crystal size | 0.68 x 0.55 x 0.47 mm$^3$ | |
| Theta range for data collection | 2.37 to 25.06°. | |
| Index ranges | -8<=h<=9, -19<=k<=20, -13<=l<=14 | |
| Reflections collected | 24255 | |
| Independent reflections | 2817 [R(int) = 0.0836] | |
| Completeness to theta = 25.06° | 99.3 % | |
| Absorption correction | multi-scan | |
| Max. and min. transmission | 0.9574 and 0.9392 | |
| Refinement method | Full-matrix least-squares on F$^2$ | |
| Data / restraints / parameters | 2817 / 0 / 203 | |
| Goodness-of-fit on F$^2$ | 1.032 | |
| Final R indices [I>2sigma(I)] | R1 = 0.0558, wR2 = 0.1520 | |
| R indices (all data) | R1 = 0.0642, wR2 = 0.1598 | |
| Largest diff. peak and hole | 0.607 and -0.281 e.Å $^{-3}$ | |

The recrystallization of **10** was carried out by the vapor diffusion method: A 4-mL vial containing a solution of **10** (15 mg) in diethyl ether (2 mL) was placed into a 20 mL vial containing *n*-hexane (10 mL). The outer bottle was capped and allowed to stand at -20 °C for 10 days to afford the compound **10** crystals.

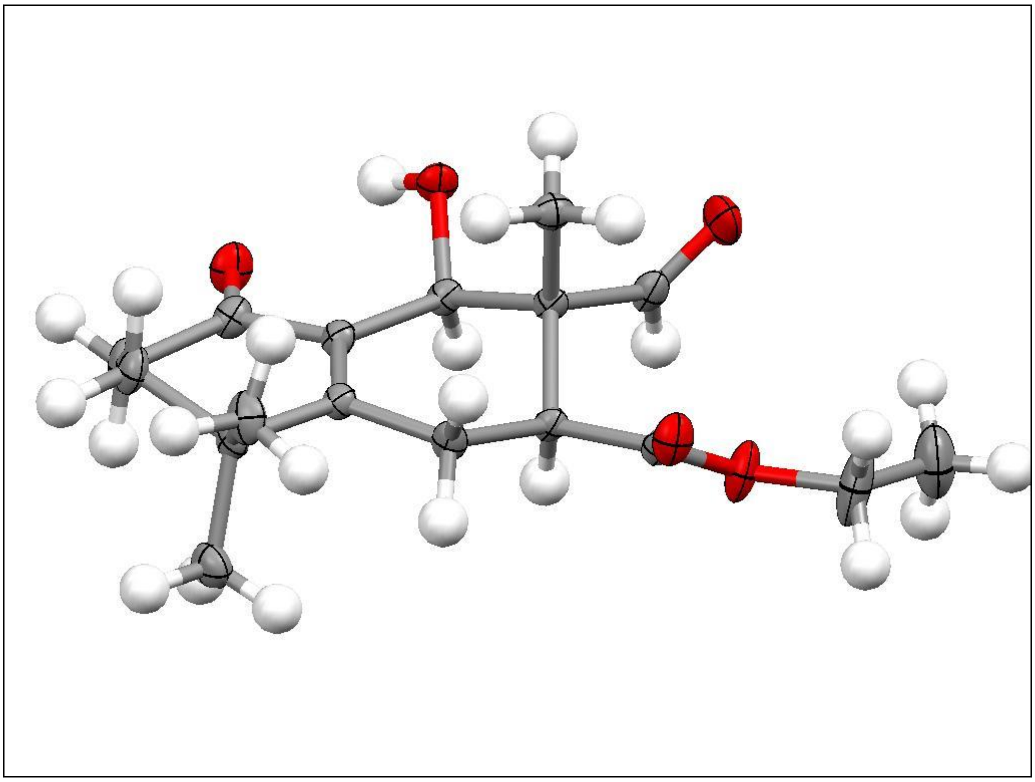

X-ray crystal structure of **10** (CCDC 2074049)
Thermal ellipsoid plot at 50% probability level.

Table S2. Crystal data and structure refinement for **10** (d22829).

| | | |
|---|---|---|
| Identification code | d22829 | |
| Empirical formula | $C_{17}H_{24}O_5$ | |
| Formula weight | 308.36 | |
| Temperature | 200(2) K | |
| Wavelength | 0.71073 Å | |
| Crystal system | Triclinic | |
| Space group | P -1 | |
| Unit cell dimensions | a = 10.4190(6) Å | a= 113.236(2)°. |
| | b = 12.7565(7) Å | b= 90.229(2)°. |
| | c = 14.1228(8) Å | g = 106.772(2)°. |
| Volume | 1636.55(16) $Å^3$ | |
| Z | 4 | |
| Density (calculated) | 1.252 $Mg/m^3$ | |
| Absorption coefficient | 0.091 $mm^{-1}$ | |
| F(000) | 664 | |
| Crystal size | 0.65 x 0.62 x 0.07 $mm^3$ | |
| Theta range for data collection | 2.06 to 25.12°. | |
| Index ranges | -12<=h<=12, -15<=k<=15, -16<=l<=16 | |
| Reflections collected | 42139 | |
| Independent reflections | 5792 [R(int) = 0.1064] | |
| Completeness to theta = 25.12° | 99.1 % | |
| Absorption correction | multi-scan | |
| Max. and min. transmission | 0.9936 and 0.9431 | |
| Refinement method | Full-matrix least-squares on $F^2$ | |
| Data / restraints / parameters | 5792 / 0 / 405 | |
| Goodness-of-fit on $F^2$ | 1.034 | |
| Final R indices [I>2sigma(I)] | R1 = 0.0785, wR2 = 0.2149 | |
| R indices (all data) | R1 = 0.0995, wR2 = 0.2391 | |
| Largest diff. peak and hole | 0.489 and -0.578 e.$Å^{-3}$ | |

The recrystallization of **15** was carried out by the vapor diffusion method: A 4-mL vial containing a solution of **15** (15 mg) in diethyl ether (2 mL) was placed into a 20 mL vial containing *n*-hexane (6 mL). The outer bottle was capped and allowed to stand at 4 °C for 5 days to afford the compound **15** crystals.

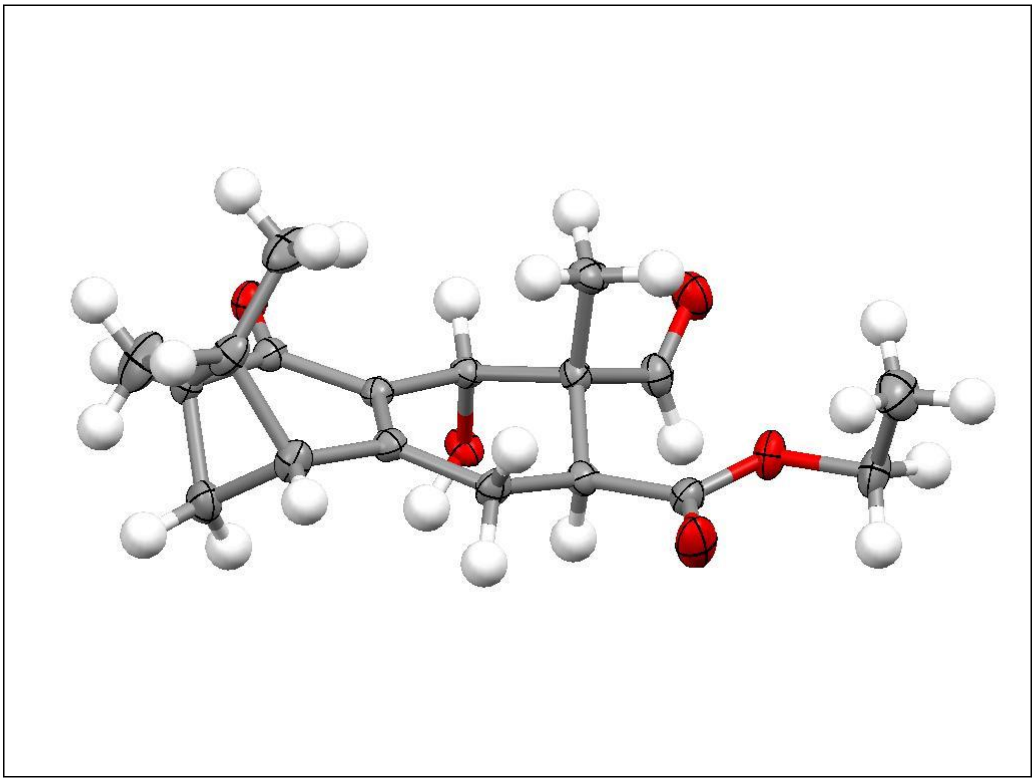

X-ray crystal structure of **15** (CCDC 2007692)

Thermal ellipsoid plot at 50% probability level.

Table S3. Crystal data and structure refinement for **15** (d22403).

| | | |
|---|---|---|
| Identification code | d22403 | |
| Empirical formula | $C_{18}H_{24}O_5$ | |
| Formula weight | 320.37 | |
| Temperature | 200(2) K | |
| Wavelength | 0.71073 Å | |
| Crystal system | Monoclinic | |
| Space group | P 21 | |
| Unit cell dimensions | a = 10.403(3) Å | a= 90°. |
| | b = 7.4914(16) Å | b= 90.839(7)°. |
| | c = 10.777(3) Å | g = 90°. |
| Volume | 839.8(3) Å $^3$ | |
| Z | 2 | |
| Density (calculated) | 1.267 Mg/m$^3$ | |
| Absorption coefficient | 0.092 mm$^{-1}$ | |
| F(000) | 344 | |
| Crystal size | 0.79 x 0.13 x 0.04 mm$^3$ | |
| Theta range for data collection | 2.70 to 25.14°. | |
| Index ranges | -12<=h<=12, -8<=k<=8, -11<=l<=12 | |
| Reflections collected | 13459 | |
| Independent reflections | 2978 [R(int) = 0.0628] | |
| Completeness to theta = 25.14° | 99.6 % | |
| Absorption correction | multi-scan | |
| Max. and min. transmission | 0.9963 and 0.9312 | |
| Refinement method | Full-matrix least-squares on F$^2$ | |
| Data / restraints / parameters | 2978 / 1 / 212 | |
| Goodness-of-fit on F$^2$ | 1.057 | |
| Final R indices [I>2sigma(I)] | R1 = 0.0416, wR2 = 0.0970 | |
| R indices (all data) | R1 = 0.0551, wR2 = 0.1035 | |
| Absolute structure parameter | -1.4(12) | |
| Largest diff. peak and hole | 0.144 and -0.147 e.Å $^{-3}$ | |

The compound **22** (30 mg) in a screw-capped vial (20 mL vial) was dissolved in $CH_2Cl_2$ (10 mL). The 20 mL vial was closed gently with a screw cap and stands it for 5 days until most of solvent was completed evaporate. The crystals of compound **22** was formed and subjected to singlecrystal X-ray analysis.

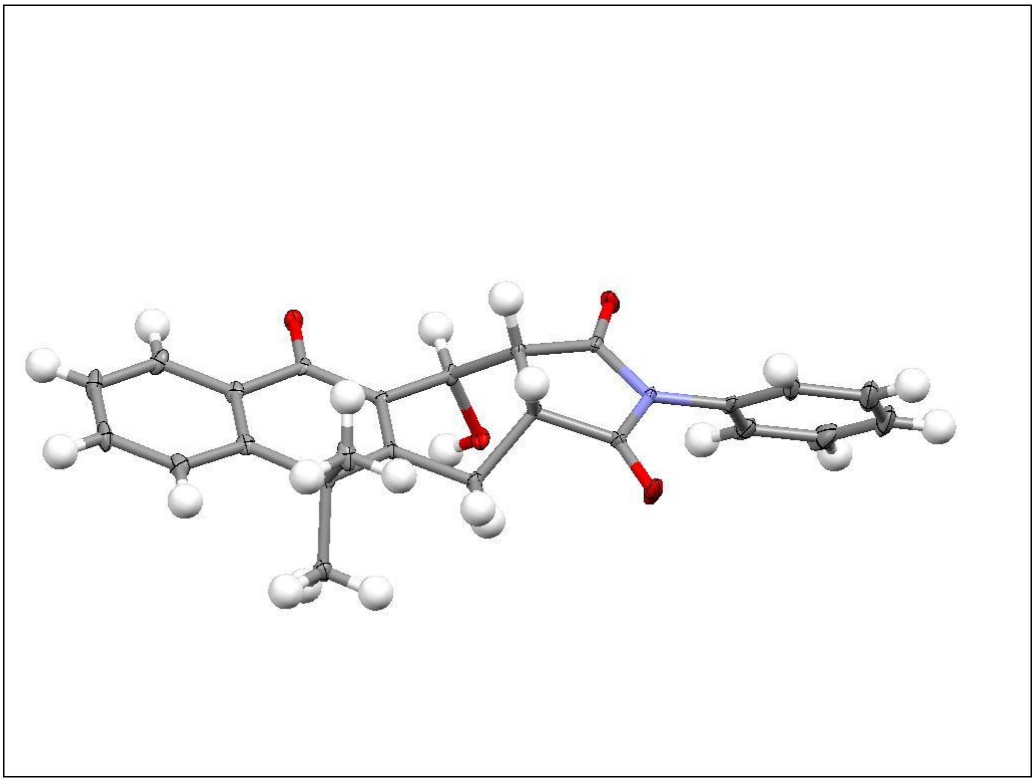

X-ray crystal structure of **22** (CCDC 2074040)

Thermal ellipsoid plot at 35% probability level.

Table S4. Crystal data and structure refinement for **22** (cu_200208lt_cu_0m_a).

| | | |
|---|---|---|
| Identification code | cu_200208lt_cu_0m_a | |
| Empirical formula | $C_{147}H_{132}Cl_6N_6O_{24}$ | |
| Formula weight | 2579.28 | |
| Temperature | 100(2) K | |
| Wavelength | 1.54178 Å | |
| Crystal system | Monoclinic | |
| Space group | $P2_1/n$ | |
| Unit cell dimensions | a = 30.0942(9) Å | a= 90°. |
| | b = 12.1684(4) Å | b= 97.433(2)°. |
| | c = 34.5883(11) Å | g = 90°. |
| Volume | 12559.7(7) Å $^3$ | |
| Z | 4 | |
| Density (calculated) | 1.364 Mg/m$^3$ | |
| Absorption coefficient | 1.881 mm$^{-1}$ | |
| F(000) | 5400 | |
| Crystal size | 0.20 x 0.06 x 0.06 mm$^3$ | |
| Theta range for data collection | 1.832 to 66.683°. | |
| Index ranges | -33<=h<=34, -12<=k<=14, -37<=l<=41 | |
| Reflections collected | 118819 | |
| Independent reflections | 21469 [R(int) = 0.0474] | |
| Completeness to theta = 67.679° | 94.4 % | |
| Absorption correction | Semi-empirical from equivalents | |
| Max. and min. transmission | 0.7528 and 0.6322 | |
| Refinement method | Full-matrix least-squares on F$^2$ | |
| Data / restraints / parameters | 21469 / 0 / 1666 | |
| Goodness-of-fit on F$^2$ | 1.051 | |
| Final R indices [I>2sigma(I)] | R1 = 0.0908, wR2 = 0.2511 | |
| R indices (all data) | R1 = 0.1007, wR2 = 0.2620 | |
| Extinction coefficient | n/a | |
| Largest diff. peak and hole | 1.783 and -1.235 e.Å $^{-3}$ | |

The recrystallization of **24** was carried out by the vapor diffusion method: A 4-mL vial containing a solution of **24** (15 mg) in ehtyl acetate (2 mL) was placed into a 20 mL vial containing *n*-hexane (6 mL). The outer bottle was capped and allowed to stand at 4 °C for 3 days to afford the compound **24** crystals.

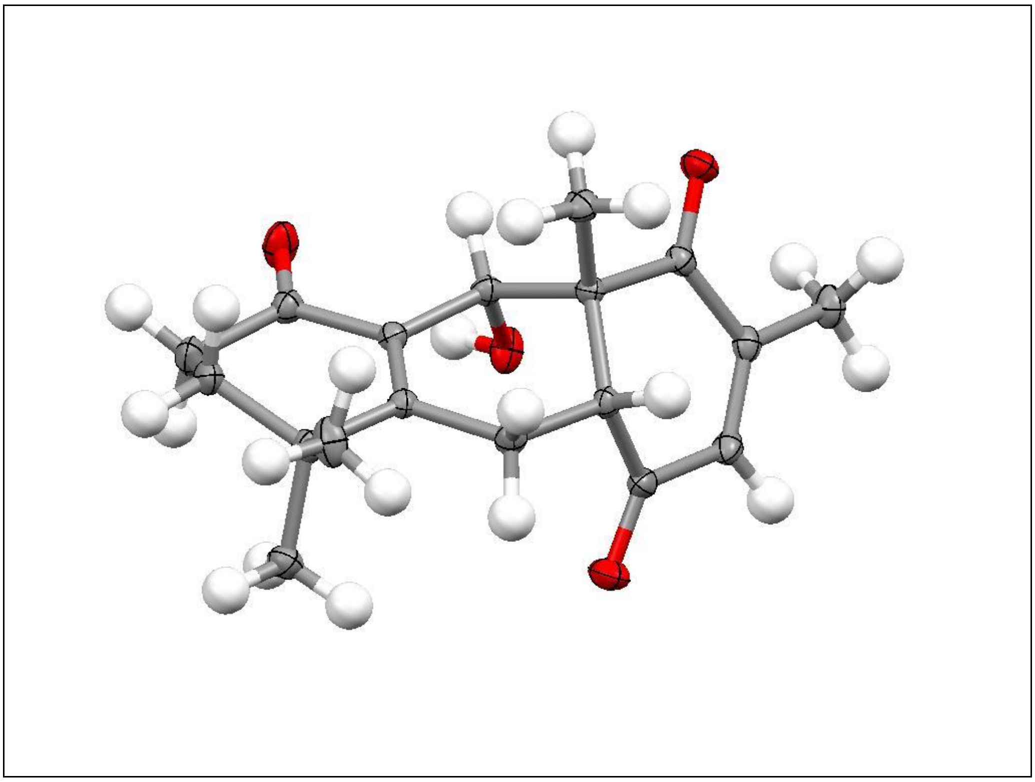

X-ray crystal structure of **24** (CCDC 2074071)

Thermal ellipsoid plot at 50% probability level.

Table S5. Crystal data and structure refinement for **24** (d22830).

| | | |
|---|---|---|
| Identification code | d22830 | |
| Empirical formula | $C_{18}H_{22}O_4$ | |
| Formula weight | 302.36 | |
| Temperature | 200(2) K | |
| Wavelength | 0.71073 Å | |
| Crystal system | Monoclinic | |
| Space group | P 21/c | |
| Unit cell dimensions | a = 8.1849(8) Å | a= 90°. |
| | b = 10.1802(9) Å | b= 99.363(3)°. |
| | c = 18.3743(18) Å | g = 90°. |
| Volume | 1510.6(2) Å $^3$ | |
| Z | 4 | |
| Density (calculated) | 1.329 Mg/m$^3$ | |
| Absorption coefficient | 0.093 mm$^{-1}$ | |
| F(000) | 648 | |
| Crystal size | 0.46 x 0.42 x 0.40 mm$^3$ | |
| Theta range for data collection | 2.29 to 25.08°. | |
| Index ranges | -9<=h<=9, -12<=k<=11, -21<=l<=21 | |
| Reflections collected | 30060 | |
| Independent reflections | 2670 [R(int) = 0.0509] | |
| Completeness to theta = 25.08° | 99.4 % | |
| Absorption correction | multi-scan | |
| Max. and min. transmission | 0.9638 and 0.9585 | |
| Refinement method | Full-matrix least-squares on F$^2$ | |
| Data / restraints / parameters | 2670 / 0 / 203 | |
| Goodness-of-fit on F$^2$ | 1.020 | |
| Final R indices [I>2sigma(I)] | R1 = 0.0392, wR2 = 0.1136 | |
| R indices (all data) | R1 = 0.0456, wR2 = 0.1211 | |
| Largest diff. peak and hole | 0.231 and -0.246 e.Å $^{-3}$ | |

The recrystallization of **25** was carried out by the vapor diffusion method: A 4-mL vial containing a solution of **25** (15 mg) in diethyl ether (2 mL) was placed into a 20 mL vial containing *n*-hexane (6 mL). The outer bottle was capped and allowed to stand at 4 °C for 5 days to afford the compound **25** crystals.

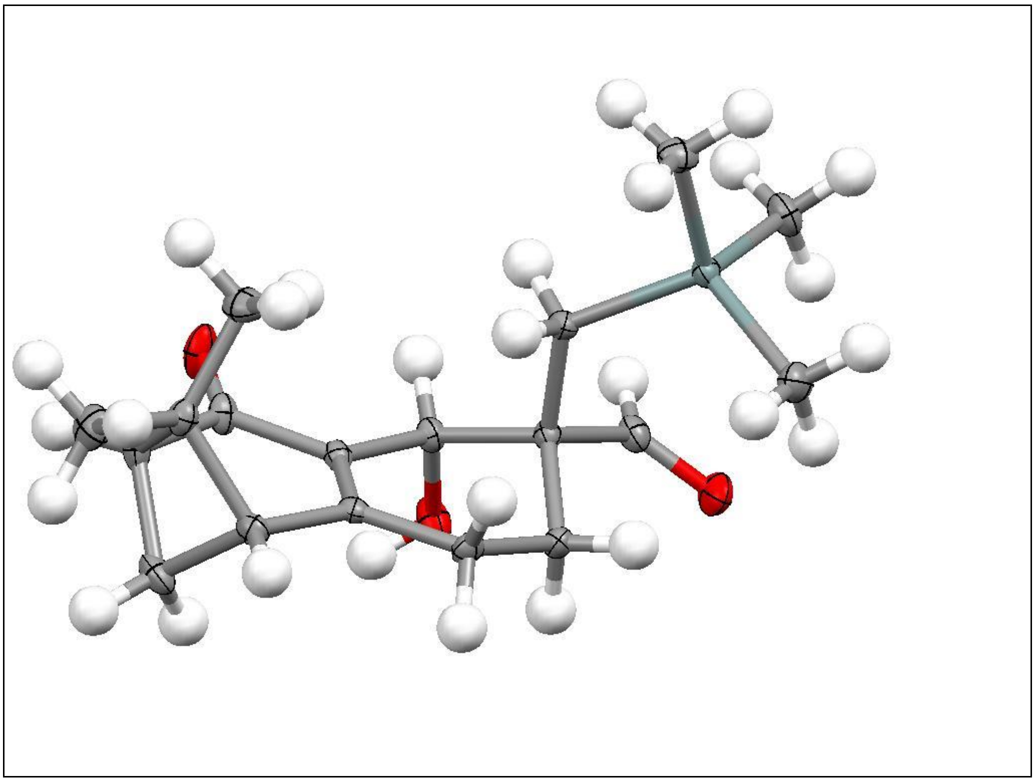

X-ray crystal structure of **25** (CCDC 2007693)

Thermal ellipsoid plot at 50% probability level.

Table S6. Crystal data and structure refinement for **25** (d22404).

| | | |
|---|---|---|
| Identification code | d22404 | |
| Empirical formula | $C_{18}H_{28}O_3Si$ | |
| Formula weight | 320.49 | |
| Temperature | 200(2) K | |
| Wavelength | 0.71073 Å | |
| Crystal system | Orthorhombic | |
| Space group | P 21 21 21 | |
| Unit cell dimensions | a = 6.4125(3) Å | a= 90°. |
| | b = 12.7287(6) Å | b= 90°. |
| | c = 21.8917(9) Å | g = 90°. |
| Volume | 1786.86(14) $Å^3$ | |
| Z | 4 | |
| Density (calculated) | 1.191 $Mg/m^3$ | |
| Absorption coefficient | 0.141 $mm^{-1}$ | |
| F(000) | 696 | |
| Crystal size | 0.51 x 0.39 x 0.23 $mm^3$ | |
| Theta range for data collection | 3.20 to 25.04°. | |
| Index ranges | -7<=h<=7, -15<=k<=15, -23<=l<=26 | |
| Reflections collected | 25393 | |
| Independent reflections | 3123 [R(int) = 0.0302] | |
| Completeness to theta = 25.04° | 98.6 % | |
| Absorption correction | multi-scan | |
| Max. and min. transmission | 0.9682 and 0.9313 | |
| Refinement method | Full-matrix least-squares on $F^2$ | |
| Data / restraints / parameters | 3123 / 0 / 206 | |
| Goodness-of-fit on $F^2$ | 1.084 | |
| Final R indices [I>2sigma(I)] | R1 = 0.0251, wR2 = 0.0660 | |
| R indices (all data) | R1 = 0.0256, wR2 = 0.0663 | |
| Absolute structure parameter | 0.02(10) | |
| Largest diff. peak and hole | 0.194 and -0.155 e.$Å^{-3}$ | |

The recrystallization of **26** was carried out by the vapor diffusion method: A 4-mL vial containing a solution of **26** (15 mg) in $CH_2Cl_2$ (2 mL) was placed into a 20 mL vial containing *n*-hexane (6 mL). The outer bottle was capped and allowed to stand at 4 ℃ for 5 days to afford the compound **26** crystals.

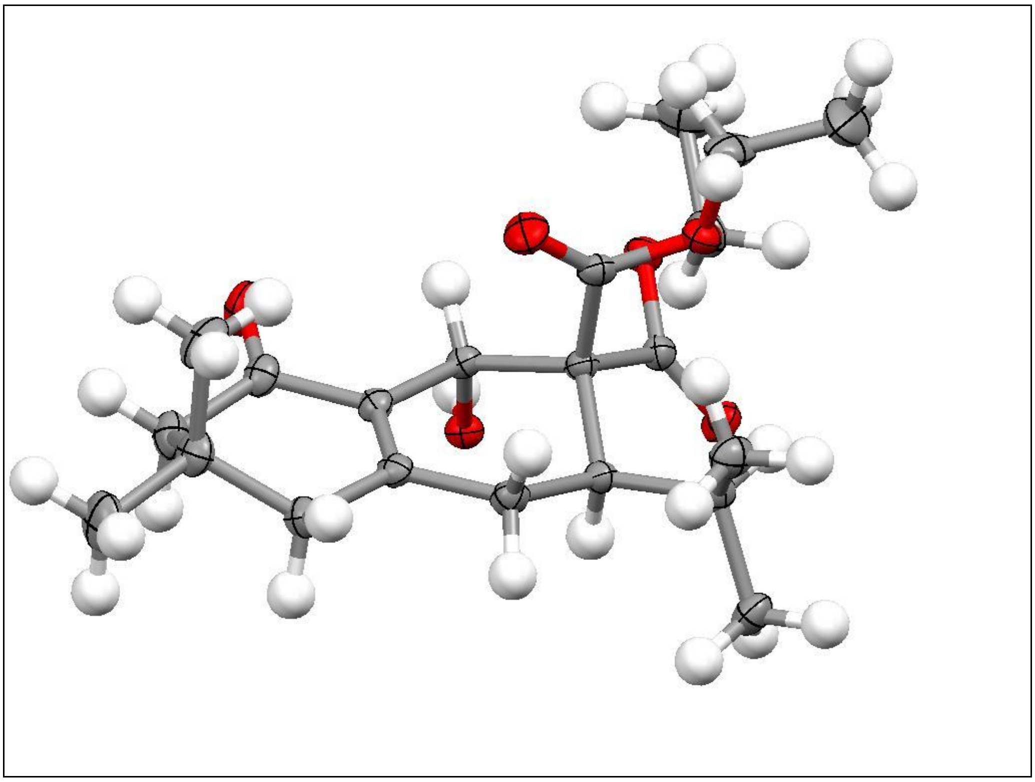

X-ray crystal structure of **26** (CCDC 2074072)
Thermal ellipsoid plot at 50% probability level.

Table S7. Crystal data and structure refinement for **26** (d22969).

| | | |
|---|---|---|
| Identification code | d22969 | |
| Empirical formula | $C_{21}H_{32}O_6$ | |
| Formula weight | 380.47 | |
| Temperature | 200(2) K | |
| Wavelength | 0.71073 Å | |
| Crystal system | Monoclinic | |
| Space group | C 2/c | |
| Unit cell dimensions | a = 26.5949(5) Å | a= 90°. |
| | b = 11.8063(3) Å | b= 104.6430(10)°. |
| | c = 28.2854(6) Å | g = 90°. |
| Volume | 8592.8(3) Å $^3$ | |
| Z | 16 | |
| Density (calculated) | 1.176 Mg/m$^3$ | |
| Absorption coefficient | 0.085 mm$^{-1}$ | |
| F(000) | 3296 | |
| Crystal size | 0.50 x 0.47 x 0.44 mm$^3$ | |
| Theta range for data collection | 1.90 to 25.05°. | |
| Index ranges | -27<=h<=31, -14<=k<=14, -33<=l<=33 | |
| Reflections collected | 67960 | |
| Independent reflections | 7598 [R(int) = 0.0458] | |
| Completeness to theta = 25.05° | 99.7 % | |
| Absorption correction | multi-scan | |
| Max. and min. transmission | 0.9636 and 0.9587 | |
| Refinement method | Full-matrix least-squares on F$^2$ | |
| Data / restraints / parameters | 7598 / 0 / 503 | |
| Goodness-of-fit on F$^2$ | 1.046 | |
| Final R indices [I>2sigma(I)] | R1 = 0.0401, wR2 = 0.1082 | |
| R indices (all data) | R1 = 0.0518, wR2 = 0.1209 | |
| Largest diff. peak and hole | 0.266 and -0.225 e.Å $^{-3}$ | |

The recrystallization of **27** was carried out by the vapor diffusion method: A 4-mL vial containing a solution of **27** (15 mg) in ethyl acetate (2 mL) was placed into a 20 mL vial containing *n*-hexane (6 mL). The outer bottle was capped and allowed to stand at 4 °C for 10 days to afford the compound **27** crystals.

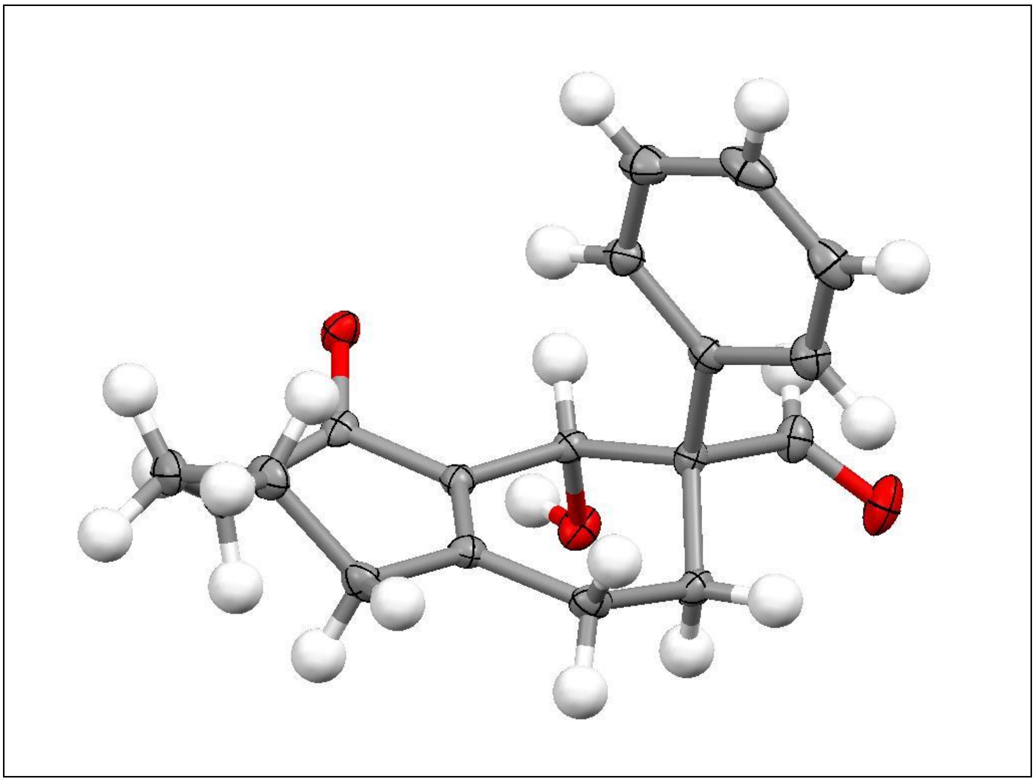

X-ray crystal structure of **27** (CCDC 2074073)
Thermal ellipsoid plot at 50% probability level.

Table S8. Crystal data and structure refinement for **27** (d22970).

| | | |
|---|---|---|
| Identification code | d22970 | |
| Empirical formula | $C_{18}H_{20}O_3$ | |
| Formula weight | 284.34 | |
| Temperature | 200(2) K | |
| Wavelength | 0.71073 Å | |
| Crystal system | Triclinic | |
| Space group | P -1 | |
| Unit cell dimensions | a = 7.1806(3) Å | a= 87.5350(10)°. |
| | b = 9.7298(3) Å | b= 86.4550(10)°. |
| | c = 10.3457(4) Å | g = 88.8730(10)°. |
| Volume | 720.66(5) Å $^3$ | |
| Z | 2 | |
| Density (calculated) | 1.310 Mg/m$^3$ | |
| Absorption coefficient | 0.088 mm$^{-1}$ | |
| F(000) | 304 | |
| Crystal size | 0.39 x 0.30 x 0.06 mm$^3$ | |
| Theta range for data collection | 2.82 to 25.17°. | |
| Index ranges | -8<=h<=8, -11<=k<=11, -12<=l<=12 | |
| Reflections collected | 25742 | |
| Independent reflections | 2580 [R(int) = 0.0565] | |
| Completeness to theta = 25.17° | 99.2 % | |
| Absorption correction | multi-scan | |
| Max. and min. transmission | 0.9947 and 0.9665 | |
| Refinement method | Full-matrix least-squares on F$^2$ | |
| Data / restraints / parameters | 2580 / 0 / 192 | |
| Goodness-of-fit on F$^2$ | 1.002 | |
| Final R indices [I>2sigma(I)] | R1 = 0.0357, wR2 = 0.1071 | |
| R indices (all data) | R1 = 0.0406, wR2 = 0.1142 | |
| Largest diff. peak and hole | 0.265 and -0.199 e.Å $^{-3}$ | |

The recrystallization of **29** was carried out by the vapor diffusion method: A 4-mL vial containing a solution of **29** (15 mg) in $CH_2Cl_2$ (2 mL) was placed into a 20 mL vial containing *n*-hexane (10 mL). The outer bottle was capped and allowed to stand at room temperature for 3 days to afford the compound **29** crystals.

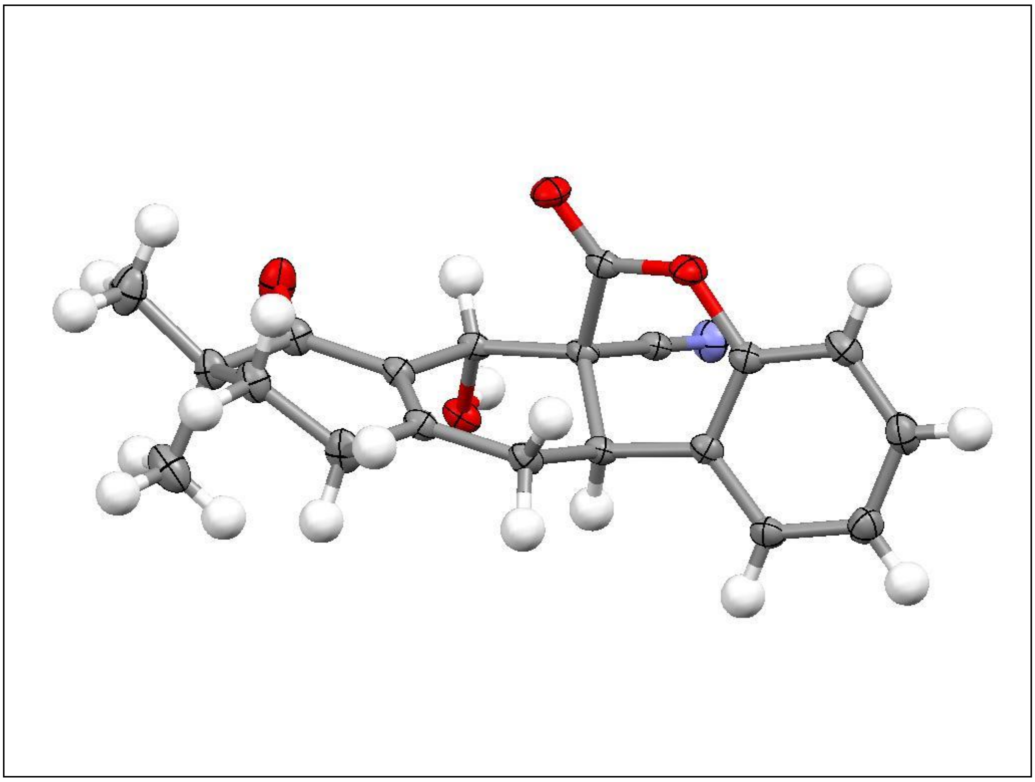

X-ray crystal structure of **29** (CCDC 2074074)

Thermal ellipsoid plot at 50% probability level.

Table S9. Crystal data and structure refinement for **29** (d23038).

| | | |
|---|---|---|
| Identification code | d23038 | |
| Empirical formula | $C_{20}H_{19}NO_4$ | |
| Formula weight | 337.36 | |
| Temperature | 200(2) K | |
| Wavelength | 0.71073 Å | |
| Crystal system | Monoclinic | |
| Space group | P 21/n | |
| Unit cell dimensions | a = 7.2284(5) Å | a= 90°. |
| | b = 20.5421(11) Å | b= 95.141(2)°. |
| | c = 11.3844(7) Å | g = 90°. |
| Volume | 1683.63(18) Å $^3$ | |
| Z | 4 | |
| Density (calculated) | 1.331 Mg/m$^3$ | |
| Absorption coefficient | 0.093 mm$^{-1}$ | |
| F(000) | 712 | |
| Crystal size | 0.72 x 0.05 x 0.02 mm$^3$ | |
| Theta range for data collection | 2.68 to 25.21°. | |
| Index ranges | -8<=h<=8, -24<=k<=24, -13<=l<=12 | |
| Reflections collected | 28885 | |
| Independent reflections | 3004 [R(int) = 0.0821] | |
| Completeness to theta = 25.21° | 98.9 % | |
| Absorption correction | multi-scan | |
| Max. and min. transmission | 0.9981 and 0.9361 | |
| Refinement method | Full-matrix least-squares on F$^2$ | |
| Data / restraints / parameters | 3004 / 0 / 230 | |
| Goodness-of-fit on F$^2$ | 1.044 | |
| Final R indices [I>2sigma(I)] | R1 = 0.0388, wR2 = 0.0982 | |
| R indices (all data) | R1 = 0.0500, wR2 = 0.1089 | |
| Largest diff. peak and hole | 0.205 and -0.206 e.Å $^{-3}$ | |

The recrystallization of **30** was carried out by the vapor diffusion method: A 4-mL vial containing a solution of **30** (15 mg) in ehtyl acetate (2 mL) was placed into a 20 mL vial containing *n*-hexane (6 mL). The outer bottle was capped and allowed to stand at 4 °C for 3 days to afford the compound **30** crystals.

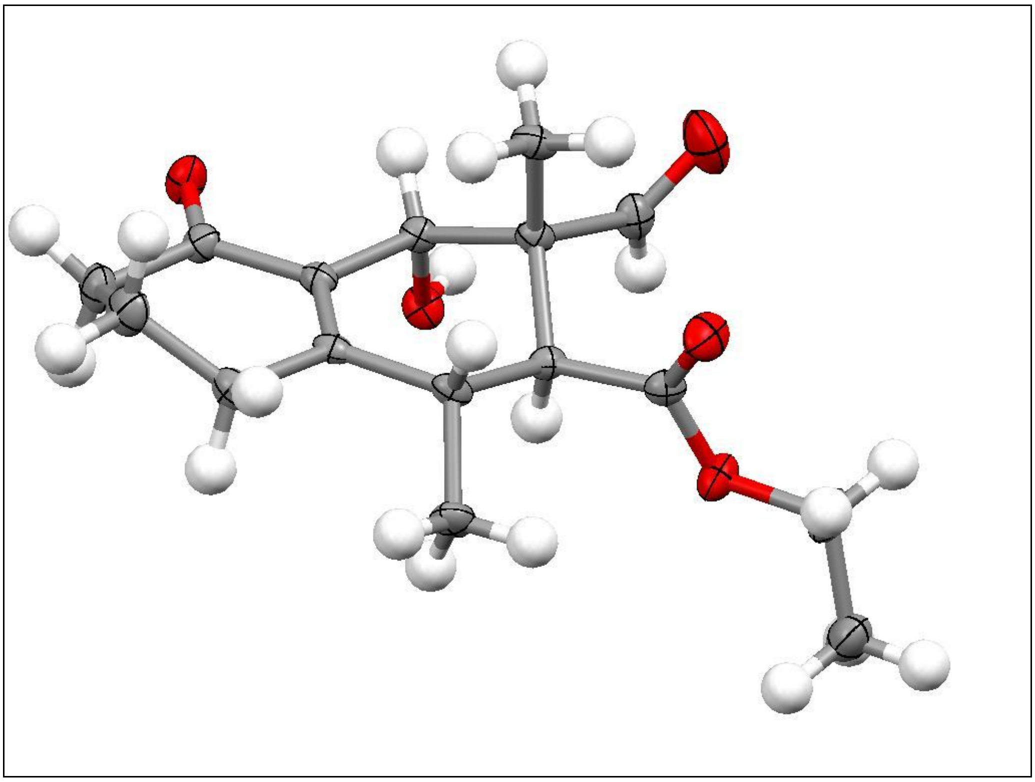

X-ray crystal structure of **30** (CCDC 2074075)

Thermal ellipsoid plot at 50% probability level.

Table S10. Crystal data and structure refinement for **30** (d23039).

| | | |
|---|---|---|
| Identification code | d23039 | |
| Empirical formula | $C_{16}H_{22}O_5$ | |
| Formula weight | 294.34 | |
| Temperature | 200(2) K | |
| Wavelength | 0.71073 Å | |
| Crystal system | Triclinic | |
| Space group | P -1 | |
| Unit cell dimensions | a = 7.9928(16) Å | a= 91.834(5)°. |
| | b = 9.3677(18) Å | b= 93.703(5)°. |
| | c = 10.3408(19) Å | g = 103.042(5)°. |
| Volume | 751.8(3) $Å^3$ | |
| Z | 2 | |
| Density (calculated) | 1.300 $Mg/m^3$ | |
| Absorption coefficient | 0.096 $mm^{-1}$ | |
| F(000) | 316 | |
| Crystal size | 0.47 x 0.02 x 0.01 $mm^3$ | |
| Theta range for data collection | 1.98 to 25.07°. | |
| Index ranges | -9<=h<=9, -11<=k<=11, -12<=l<=12 | |
| Reflections collected | 19647 | |
| Independent reflections | 2648 [R(int) = 0.1523] | |
| Completeness to theta = 25.07° | 99.5 % | |
| Absorption correction | multi-scan | |
| Max. and min. transmission | 0.9990 and 0.9563 | |
| Refinement method | Full-matrix least-squares on $F^2$ | |
| Data / restraints / parameters | 2648 / 0 / 195 | |
| Goodness-of-fit on $F^2$ | 1.008 | |
| Final R indices [I>2sigma(I)] | R1 = 0.0665, wR2 = 0.1475 | |
| R indices (all data) | R1 = 0.1289, wR2 = 0.1783 | |
| Largest diff. peak and hole | 0.518 and -0.254 e.$Å^{-3}$ | |

The recrystallization of **32** was carried out by the vapor diffusion method: A 4-mL vial containing a solution of **32** (15 mg) in ehtyl acetate (2 mL) was placed into a 20 mL vial containing *n*-hexane (10 mL). The outer bottle was capped and allowed to stand at 4 ℃ for 5 days to afford the compound **32** crystals.

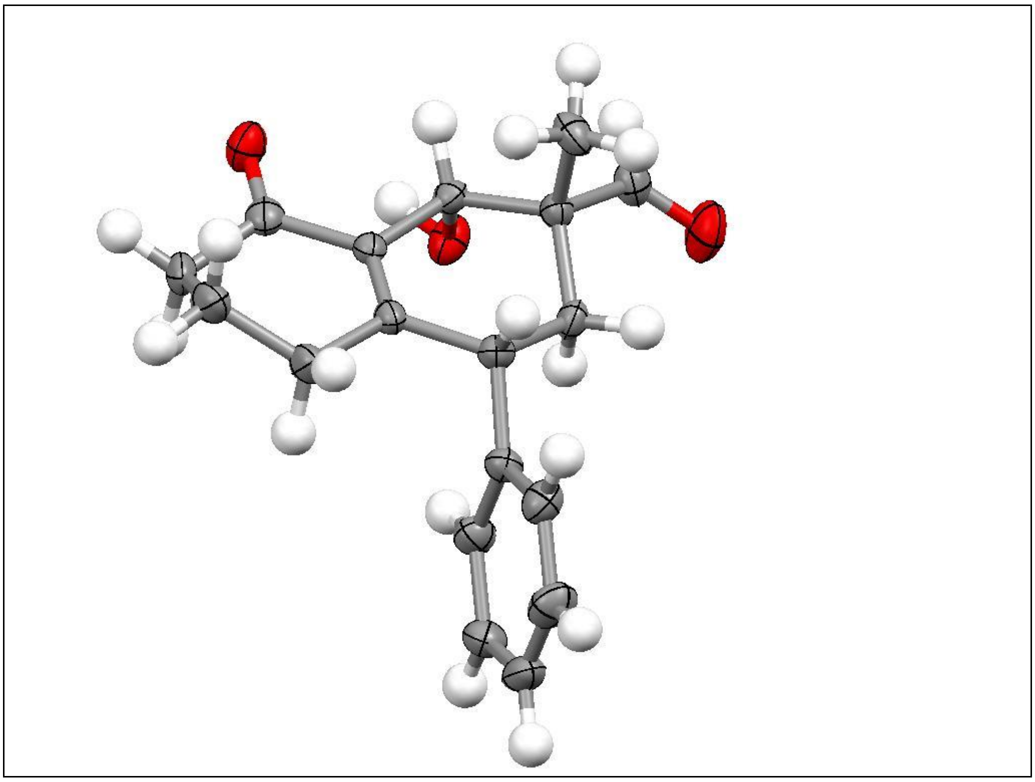

X-ray crystal structure of **32** (CCDC 2074055)
Thermal ellipsoid plot at 50% probability level.

Table S11. Crystal data and structure refinement for **32** (d22827).

| | | |
|---|---|---|
| Identification code | d22827 | |
| Empirical formula | $C_{18}H_{20}O_3$ | |
| Formula weight | 284.34 | |
| Temperature | 200(2) K | |
| Wavelength | 0.71073 Å | |
| Crystal system | Orthorhombic | |
| Space group | P 21 21 21 | |
| Unit cell dimensions | a = 5.8485(5) Å | a= 90°. |
| | b = 15.7132(15) Å | b= 90°. |
| | c = 16.2264(17) Å | g = 90°. |
| Volume | 1491.2(2) Å $^3$ | |
| Z | 4 | |
| Density (calculated) | 1.267 Mg/m$^3$ | |
| Absorption coefficient | 0.085 mm$^{-1}$ | |
| F(000) | 608 | |
| Crystal size | 0.79 x 0.23 x 0.02 mm$^3$ | |
| Theta range for data collection | 2.51 to 25.03°. | |
| Index ranges | -6<=h<=6, -15<=k<=18, -12<=l<=19 | |
| Reflections collected | 9204 | |
| Independent reflections | 2620 [R(int) = 0.1024] | |
| Completeness to theta = 25.03° | 99.8 % | |
| Absorption correction | multi-scan | |
| Max. and min. transmission | 0.9983 and 0.9359 | |
| Refinement method | Full-matrix least-squares on F$^2$ | |
| Data / restraints / parameters | 2620 / 0 / 191 | |
| Goodness-of-fit on F$^2$ | 0.986 | |
| Final R indices [I>2sigma(I)] | R1 = 0.0542, wR2 = 0.1101 | |
| R indices (all data) | R1 = 0.0855, wR2 = 0.1233 | |
| Absolute structure parameter | 0.2(19) | |
| Largest diff. peak and hole | 0.222 and -0.197 e.Å $^{-3}$ | |

The recrystallization of **36** was carried out by the vapor diffusion method: A 4-mL vial containing a solution of **36** (15 mg) in ehtyl acetate (2 mL) was placed into a 20 mL vial containing *n*-hexane (6 mL). The outer bottle was capped and allowed to stand at 4 °C for 10 days to afford the compound **36** crystals.

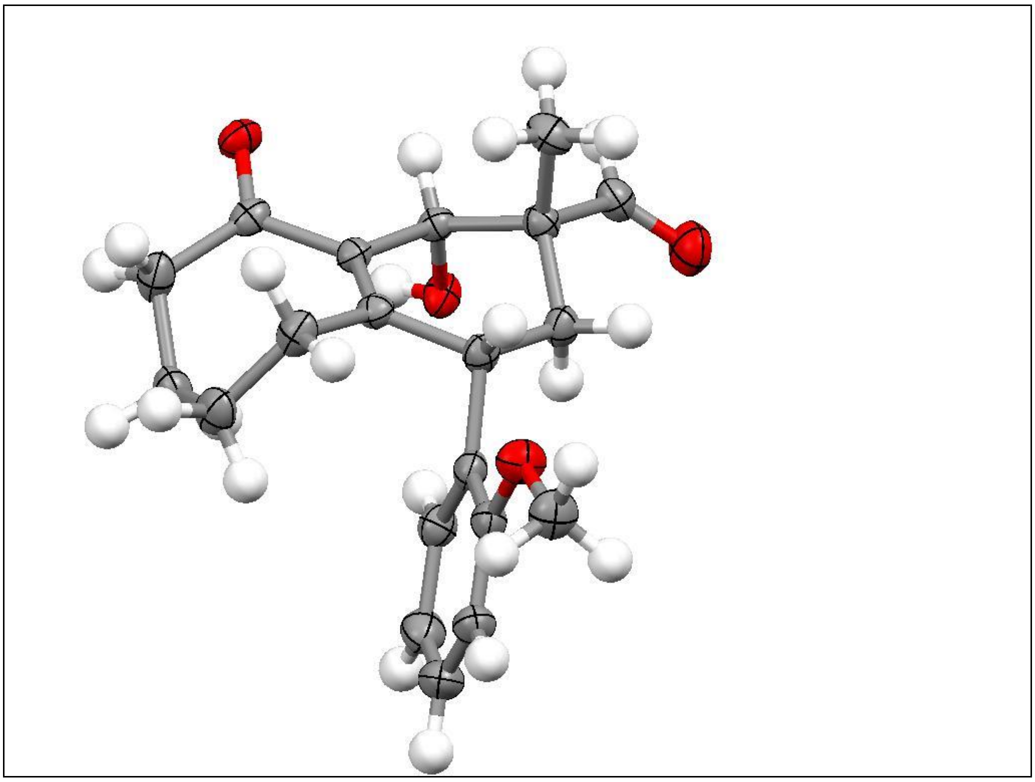

X-ray crystal structure of **36** (CCDC 2074053)
Thermal ellipsoid plot at 50% probability level.

Table S12. Crystal data and structure refinement for **36** (d22714).

| | | |
|---|---|---|
| Identification code | d22714 | |
| Empirical formula | $C_{20}H_{24}O_4$ | |
| Formula weight | 328.39 | |
| Temperature | 200(2) K | |
| Wavelength | 0.71073 Å | |
| Crystal system | Orthorhombic | |
| Space group | P b c a | |
| Unit cell dimensions | a = 7.4767(2) Å | a= 90°. |
| | b = 14.1191(5) Å | b= 90°. |
| | c = 32.2835(9) Å | g = 90°. |
| Volume | 3407.98(18) Å $^3$ | |
| Z | 8 | |
| Density (calculated) | 1.280 Mg/m$^3$ | |
| Absorption coefficient | 0.088 mm$^{-1}$ | |
| F(000) | 1408 | |
| Crystal size | 0.18 x 0.15 x 0.03 mm$^3$ | |
| Theta range for data collection | 2.52 to 25.10°. | |
| Index ranges | -8<=h<=6, -12<=k<=16, -38<=l<=29 | |
| Reflections collected | 16421 | |
| Independent reflections | 3007 [R(int) = 0.0420] | |
| Completeness to theta = 25.10° | 99.2 % | |
| Absorption correction | multi-scan | |
| Max. and min. transmission | 0.9974 and 0.9843 | |
| Refinement method | Full-matrix least-squares on F$^2$ | |
| Data / restraints / parameters | 3007 / 0 / 221 | |
| Goodness-of-fit on F$^2$ | 1.070 | |
| Final R indices [I>2sigma(I)] | R1 = 0.0413, wR2 = 0.0928 | |
| R indices (all data) | R1 = 0.0559, wR2 = 0.1021 | |
| Largest diff. peak and hole | 0.296 and -0.162 e.Å $^{-3}$ | |

The recrystallization of **41** was carried out by the vapor diffusion method: A 4-mL vial containing a solution of **41** (15 mg) in ehtyl acetate (2 mL) was placed into a 20 mL vial containing *n*-hexane (10 mL). The outer bottle was capped and allowed to stand at room temperature for 5 days to afford the compound **41** crystals.

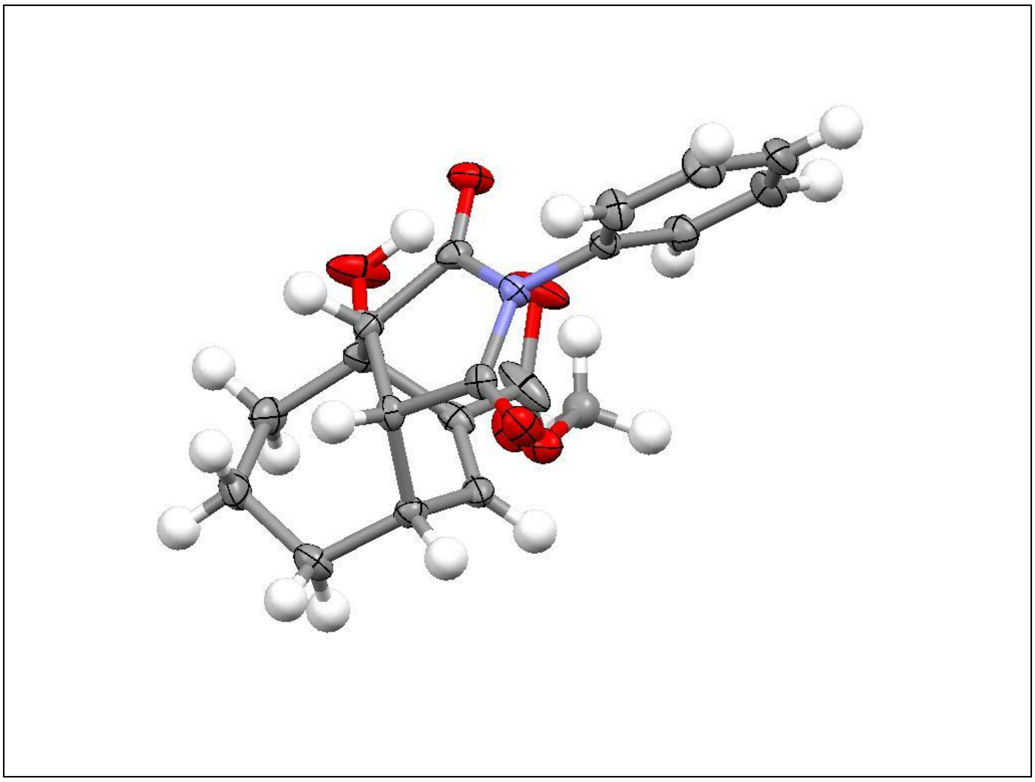

X-ray crystal structure of **41** (CCDC 2074076)
Thermal ellipsoid plot at 50% probability level.

Table S13. Crystal data and structure refinement for **41** (d23173).

| | | |
|---|---|---|
| Identification code | d23173 | |
| Empirical formula | $C_{19}H_{19}NO_5$ | |
| Formula weight | 341.35 | |
| Temperature | 200(2) K | |
| Wavelength | 0.71073 Å | |
| Crystal system | Monoclinic | |
| Space group | P 21/c | |
| Unit cell dimensions | a = 8.4700(4) Å | a= 90°. |
| | b = 18.7943(8) Å | b= 111.1640(10)°. |
| | c = 11.0135(5) Å | g = 90°. |
| Volume | 1634.96(13) Å $^{3}$ | |
| Z | 4 | |
| Density (calculated) | 1.387 Mg/m$^{3}$ | |
| Absorption coefficient | 0.101 mm$^{-1}$ | |
| F(000) | 720 | |
| Crystal size | 0.45 x 0.25 x 0.17 mm$^{3}$ | |
| Theta range for data collection | 2.80 to 25.11°. | |
| Index ranges | -10<=h<=8, -22<=k<=22, -13<=l<=13 | |
| Reflections collected | 23001 | |
| Independent reflections | 2906 [R(int) = 0.0395] | |
| Completeness to theta = 25.11° | 99.8 % | |
| Absorption correction | multi-scan | |
| Max. and min. transmission | 0.9830 and 0.9560 | |
| Refinement method | Full-matrix least-squares on F$^{2}$ | |
| Data / restraints / parameters | 2906 / 2 / 245 | |
| Goodness-of-fit on F$^{2}$ | 1.045 | |
| Final R indices [I>2sigma(I)] | R1 = 0.0433, wR2 = 0.1061 | |
| R indices (all data) | R1 = 0.0510, wR2 = 0.1132 | |
| Largest diff. peak and hole | 0.253 and -0.413 e.Å $^{-3}$ | |

The recrystallization of **43** was carried out by the vapor diffusion method: A 4-mL vial containing a solution of **43** (15 mg) in $CH_2Cl_2$ (2 mL) was placed into a 20 mL vial containing *n*-hexane (10 mL). The outer bottle was capped and allowed to stand at room temperature for 3 days to afford the compound **43** crystals.

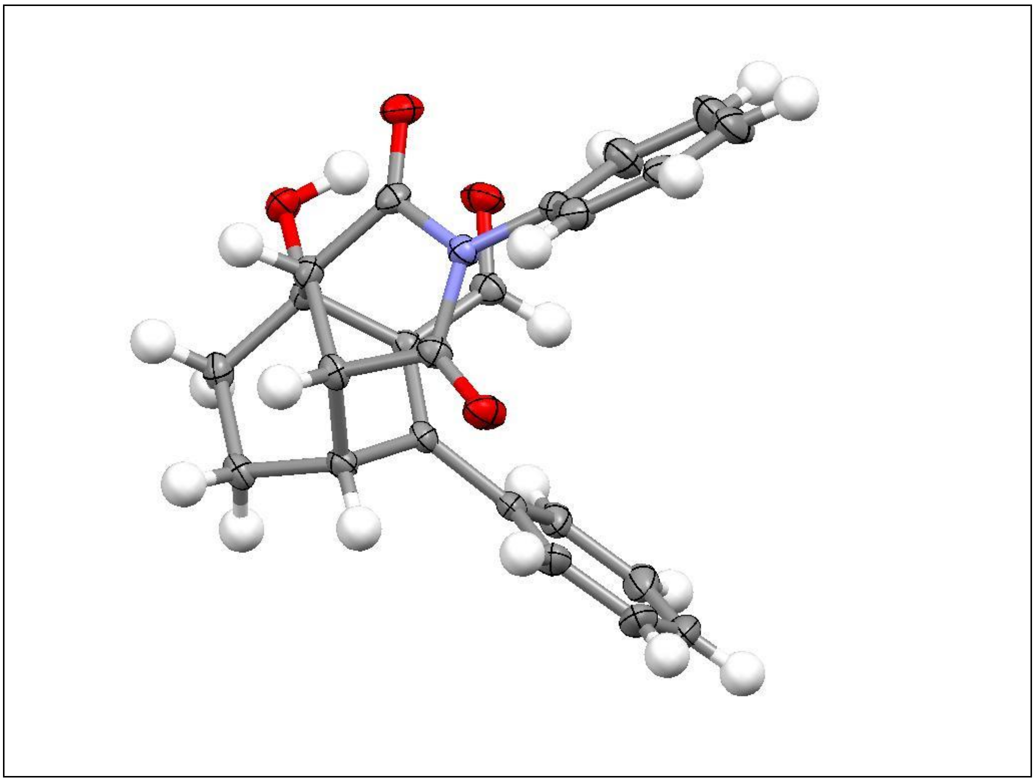

X-ray crystal structure of **43** (CCDC 2074078)
Thermal ellipsoid plot at 50% probability level.

Table S14. Crystal data and structure refinement for **43** (d23119).

| | | |
|---|---|---|
| Identification code | d23119 | |
| Empirical formula | $C_{23}H_{19}NO_4$ | |
| Formula weight | 373.39 | |
| Temperature | 200(2) K | |
| Wavelength | 0.71073 Å | |
| Crystal system | Monoclinic | |
| Space group | P 21/n | |
| Unit cell dimensions | a = 14.2305(13) Å | a= 90°. |
| | b = 7.4723(5) Å | b= 99.991(3)°. |
| | c = 17.0649(15) Å | g = 90°. |
| Volume | 1787.1(3) Å $^3$ | |
| Z | 4 | |
| Density (calculated) | 1.388 Mg/m$^3$ | |
| Absorption coefficient | 0.095 mm$^{-1}$ | |
| F(000) | 784 | |
| Crystal size | 0.20 x 0.11 x 0.07 mm$^3$ | |
| Theta range for data collection | 2.42 to 25.25°. | |
| Index ranges | -16<=h<=17, -8<=k<=8, -20<=l<=20 | |
| Reflections collected | 33679 | |
| Independent reflections | 3183 [R(int) = 0.0495] | |
| Completeness to theta = 25.26° | 98.4 % | |
| Absorption correction | multi-scan | |
| Max. and min. transmission | 0.9934 and 0.9812 | |
| Refinement method | Full-matrix least-squares on F$^2$ | |
| Data / restraints / parameters | 3183 / 0 / 253 | |
| Goodness-of-fit on F$^2$ | 1.080 | |
| Final R indices [I>2sigma(I)] | R1 = 0.0374, wR2 = 0.0998 | |
| R indices (all data) | R1 = 0.0459, wR2 = 0.1063 | |
| Largest diff. peak and hole | 0.386 and -0.210 e.Å $^{-3}$ | |

## Reference


[1] A. B. Smith, B. D. Dorsey, M. Ohba, A. T. Lupo, M. S. Malamas, *J. Org. Chem.* **1988**, *53*, 4314-4325.

[2] I. Kolodziej, J. R. Green, *Org. Biomol. Chem.* **2015**, *13*, 10852-10864.

[3] a) A. Parida, M. Sharique, B. N. Kakde, S. Ghosh, A. Bisai, *Synthesis* **2015**, *47*, 2965-2970; b) B. N. Kakde, S. Bhunia, A. Bisai, *Tetrahedron Lett.* **2013**, *54*, 1436-1439.

[4] a) F. Su, Y. Lu, L. Kong, J. Liu, T. Luo, **2018**, *57*, 760-764; b) C. Zheng, I. Dubovyk, K. E. Lazarski, R. J. Thomson, *J. Am. Chem. Soc.* **2014**, *136*, 17750-17756; c) A. Porzelle, C. M. Williams, B. D. Schwartz, I. R. Gentle, *Synlett* **2005**, *2005*, 2923-2926.

[5] A. Kmieciak, M. P. Krzemiński, *Beilstein J. Org. Chem.* **2019,** *15*, 2493–2499.

[6] T. Rajale, S. Sharma, D. A. Stroud, D. K. Unruh, E. Miaou, K. Lai, D. M. Birney, *Tetrahedron Lett.* **2014**, *55*, 6627-6630.

[7] a) Y.-T. Liu, J.-Q. Chen, L.-P. Li, X.-Y. Shao, J.-H. Xie, Q.-L. Zhou, *Org. Lett.* **2017**, *19*, 3231-3234; b) T. Nishizuka, S. Hirosawa, S. Kondo, D. Ikeda, T. Takeuchi, *J. Antibiot.* **1997**, *50*, 755-764.

[8] G. D. Brown, Q. Shi, G. V. Delucca, D. G. Batt, M. A. Galella, M.-E. Cvijic, R.-Q. Liu, F. Qiu, Q. Zhao, J. C. Barrish, P. H. Carter, *Bioorg. Med. Chem. Lett.* **2016**, *26*, 662-666.

[9] S. Ikeda, M. Shibuya, N. Kanoh, Y. Iwabuchi, *Org. Lett.* **2009**, *11*, 1833-1836.

[10] F. Rezgui, M. M. El Gaïed, *J. Chem. Res. Synop.* **1999**, 576-577.

[11] U. Chiachio, A. Liguori, A. Rescifina, G. Romeo, F. Rossano, G. Sindona, N. Uccella, *Tetrahedron* **1992**, *48*, 123-132.

[12] a) D. P. Canterbury, I. R. Herrick, J. Um, K. N. Houk, A. J. Frontier, *Tetrahedron* **2009**, *65*, 3165-3179; b) A. Padwa, U. Chiacchio, D. N. Kline, J. Perumattam, *J. Org. Chem.* **1988**, *53*, 2238-2245.

**$^{1}H$, $^{13}C$ NMR, DEPT, $^{1}H$-$^{1}H$ COSY and NOESY spectra for all new compounds:**

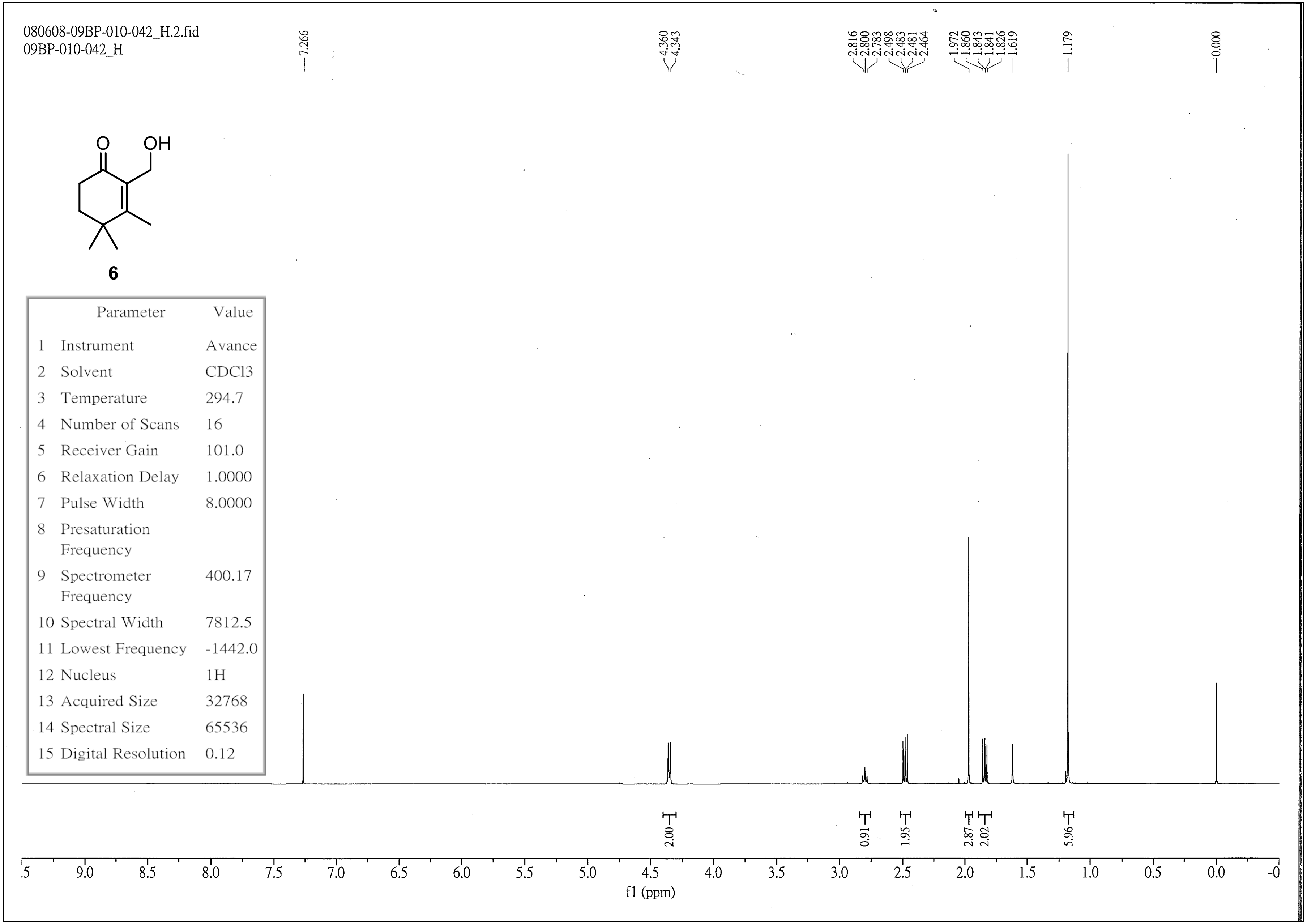


| | Parameter | Value |
|---|---|---|
| 1 | Instrument | Avance |
| 2 | Solvent | CDCl3 |
| 3 | Temperature | 294.7 |
| 4 | Number of Scans | 16 |
| 5 | Receiver Gain | 101.0 |
| 6 | Relaxation Delay | 1.0000 |
| 7 | Pulse Width | 8.0000 |
| 8 | Presaturation Frequency | |
| 9 | Spectrometer Frequency | 400.17 |
| 10 | Spectral Width | 7812.5 |
| 11 | Lowest Frequency | -1442.0 |
| 12 | Nucleus | 1H |
| 13 | Acquired Size | 32768 |
| 14 | Spectral Size | 65536 |
| 15 | Digital Resolution | 0.12 |

$^{1}H$ NMR spectrum for compound **6**

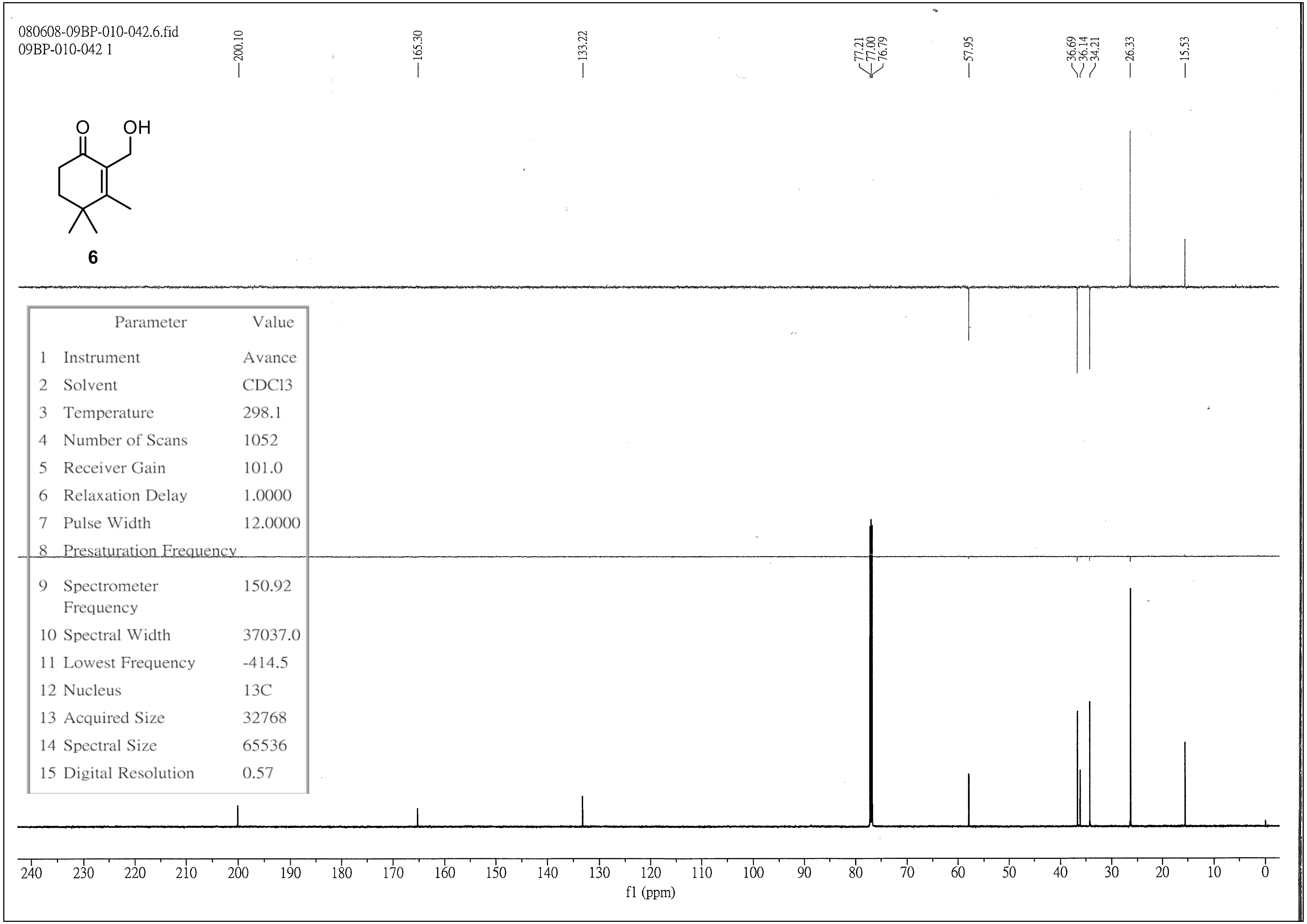


| | Parameter | Value |
|---|---|---|
| 1 | Instrument | Avance |
| 2 | Solvent | CDCl3 |
| 3 | Temperature | 298.1 |
| 4 | Number of Scans | 1052 |
| 5 | Receiver Gain | 101.0 |
| 6 | Relaxation Delay | 1.0000 |
| 7 | Pulse Width | 12.0000 |
| 8 | Presaturation Frequency | |
| 9 | Spectrometer Frequency | 150.92 |
| 10 | Spectral Width | 37037.0 |
| 11 | Lowest Frequency | -414.5 |
| 12 | Nucleus | 13C |
| 13 | Acquired Size | 32768 |
| 14 | Spectral Size | 65536 |
| 15 | Digital Resolution | 0.57 |

$^{13}$C NMR + DEPT spectra for compound **6**

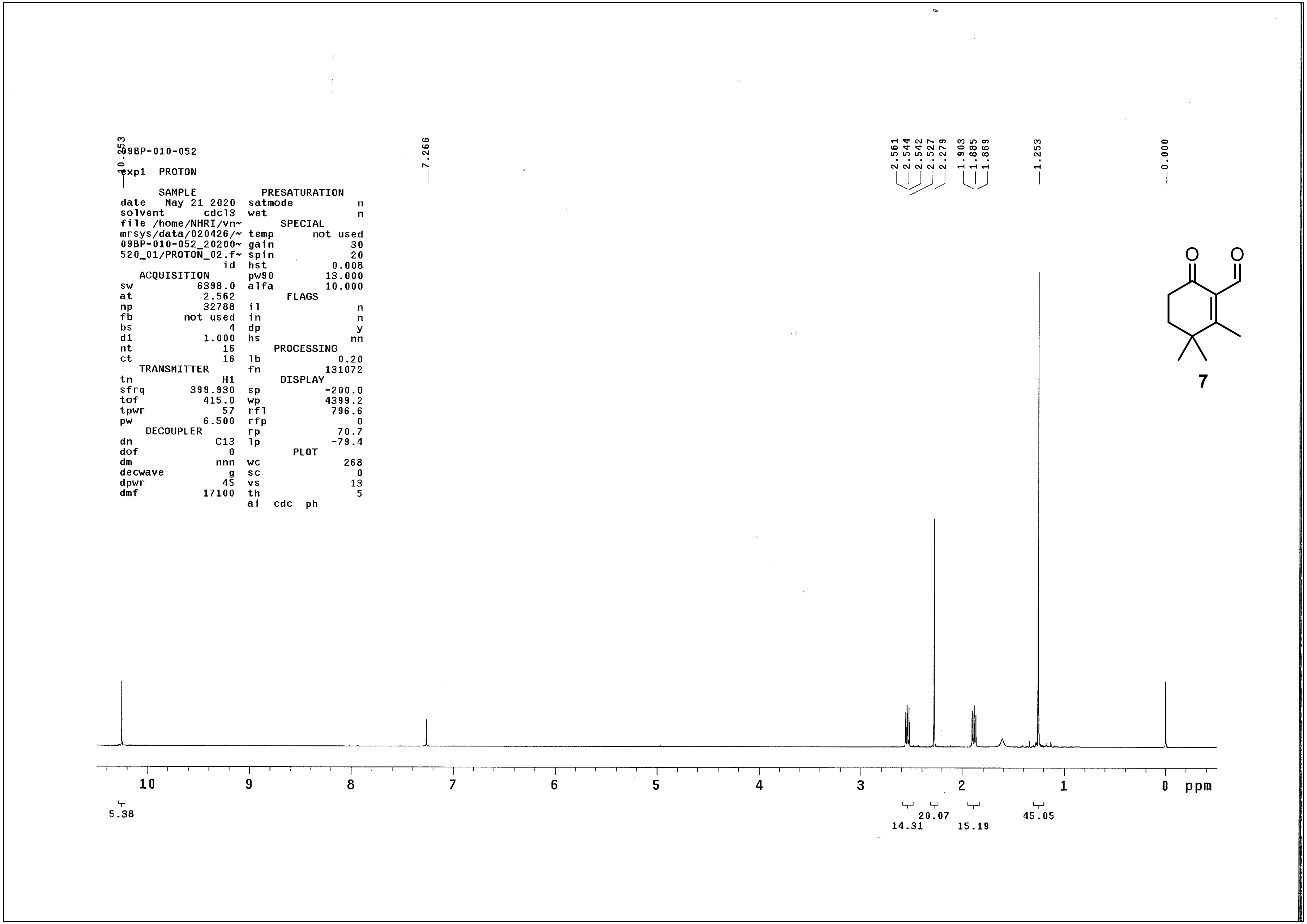


$^{1}$H NMR spectrum for compound **7**

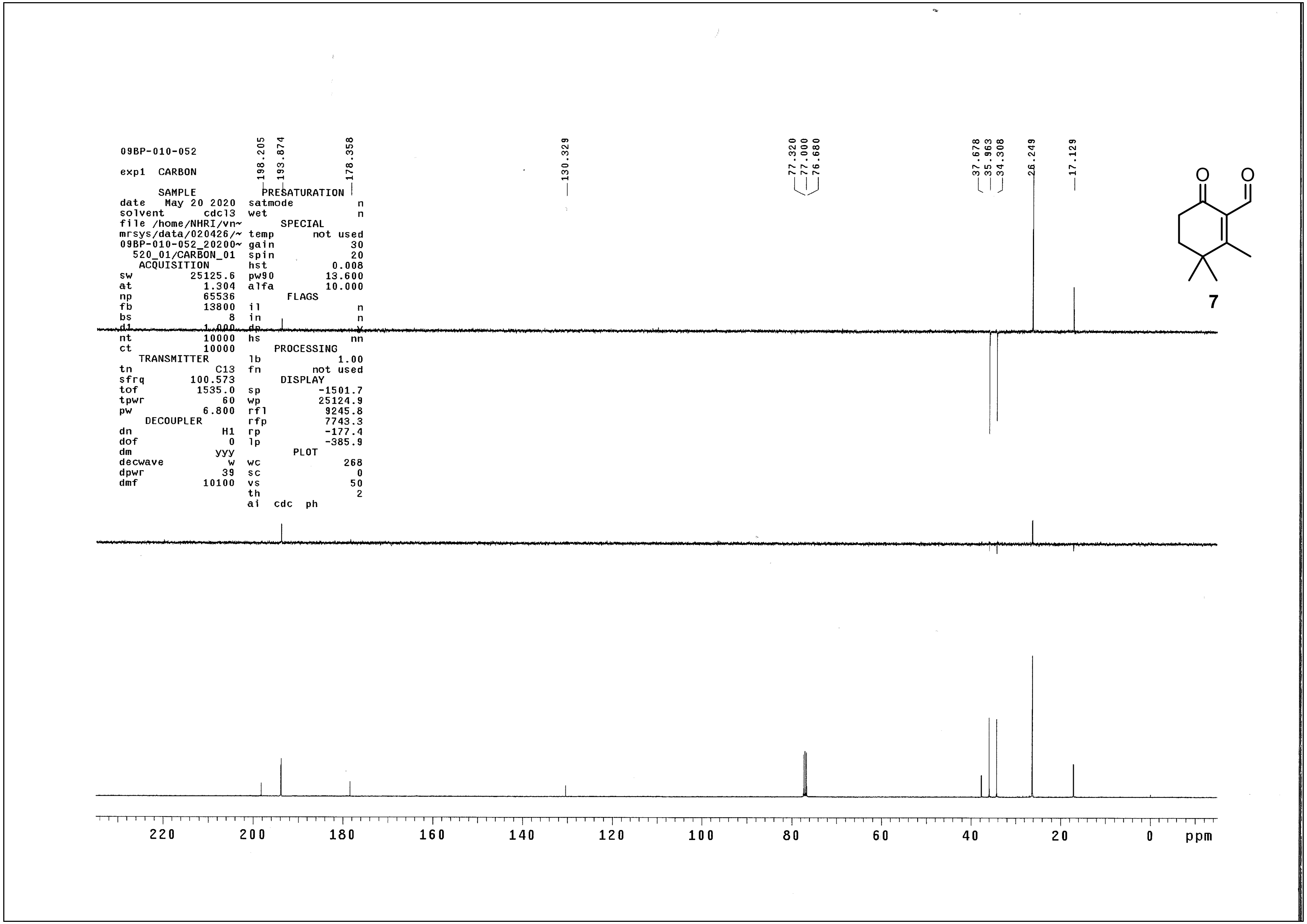


$^{13}$C NMR + DEPT spectra for compound **7**

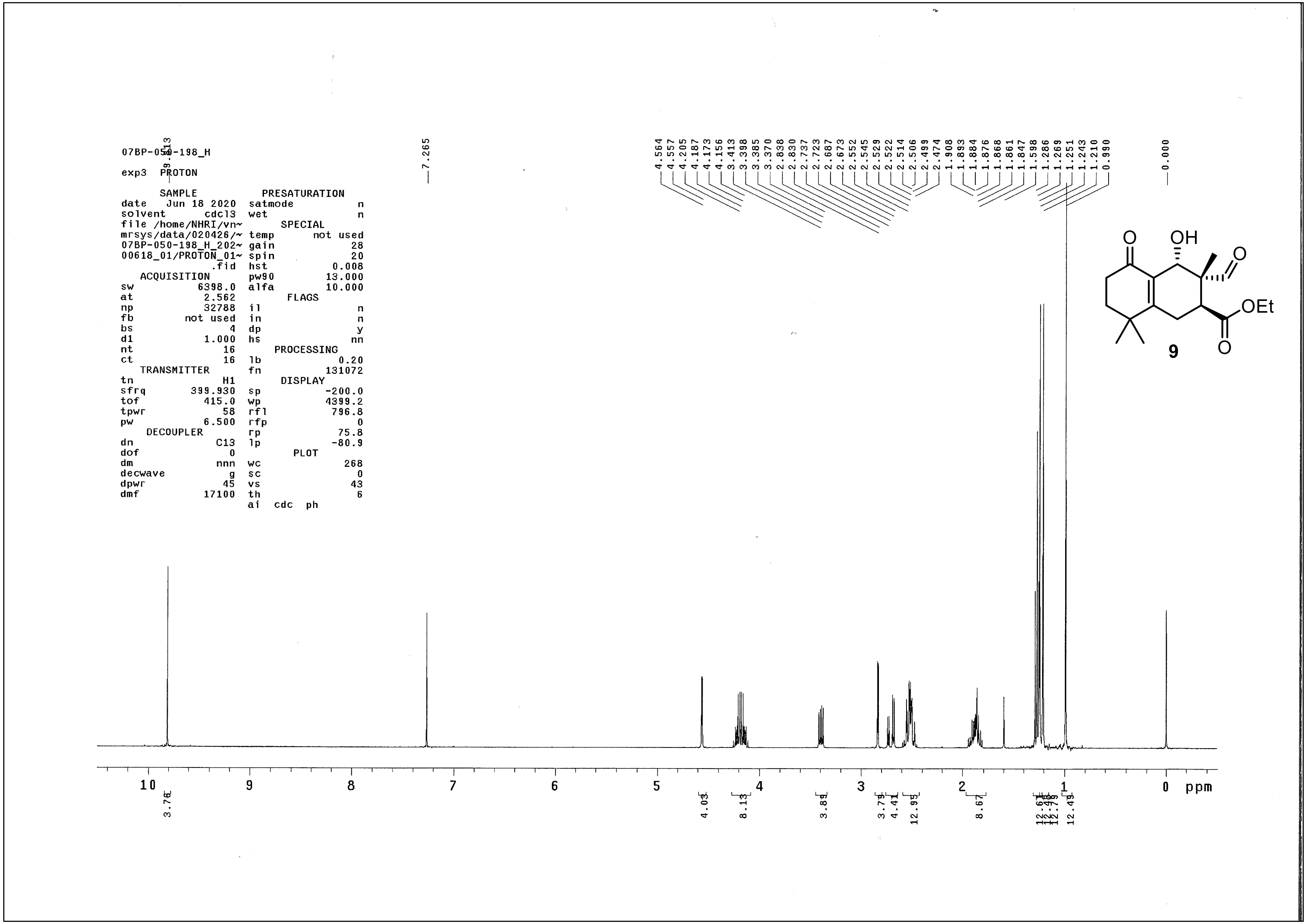

$^{1}$H NMR spectrum for compound **9**

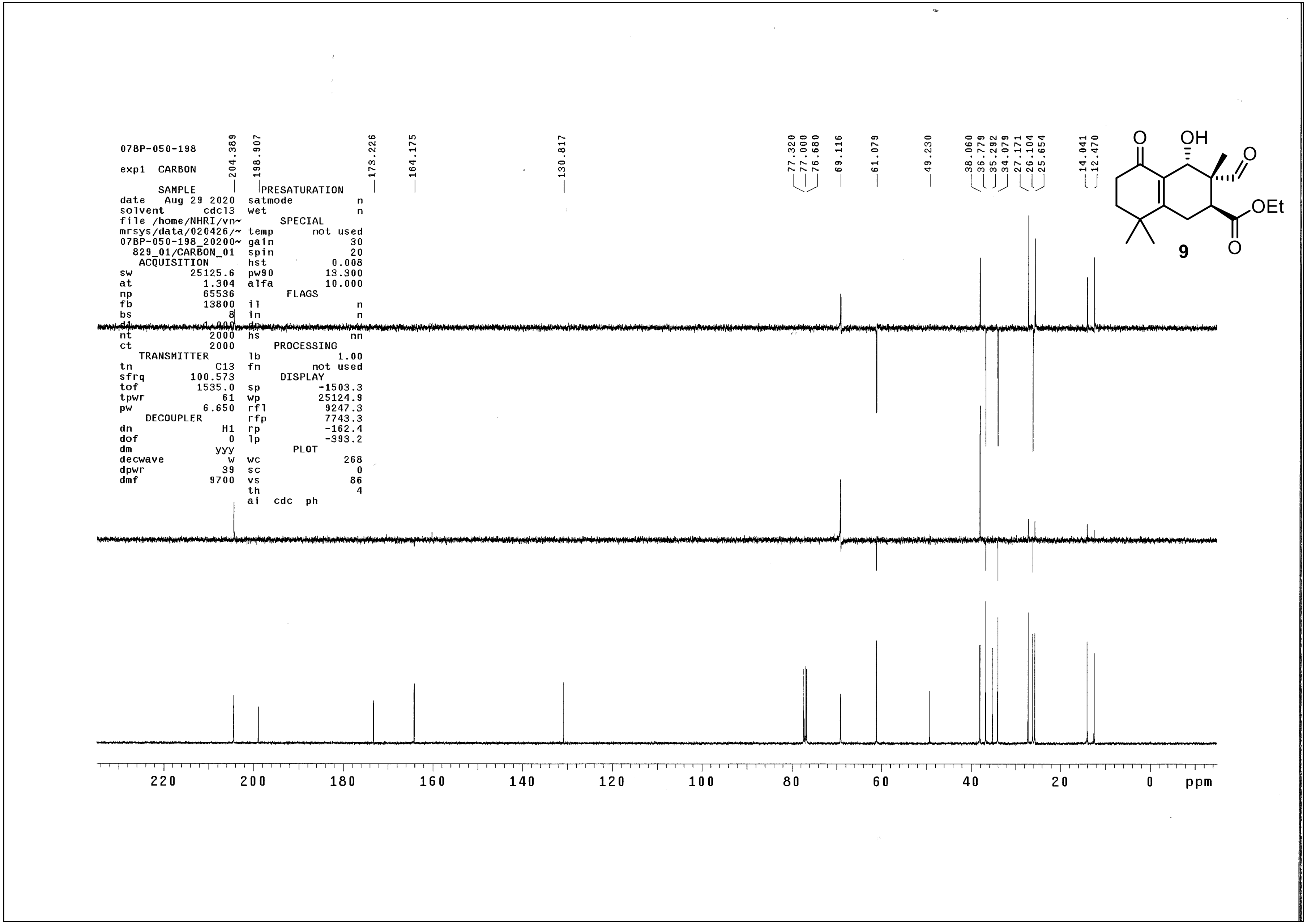


$^{13}$C NMR + DEPT spectra for compound **9**

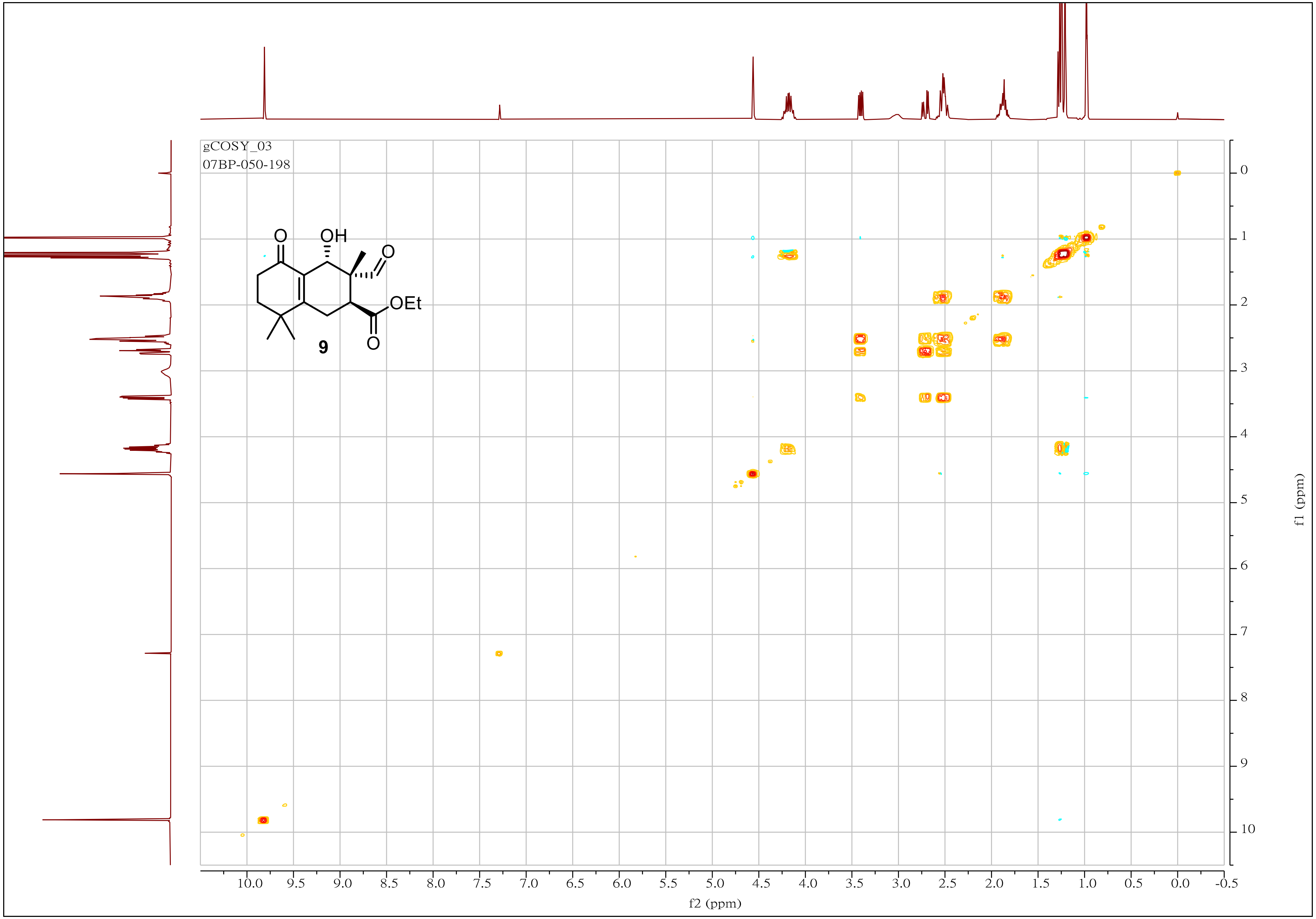


$^{1}H$-$^{1}H$ COSY spectrum for compound **9**

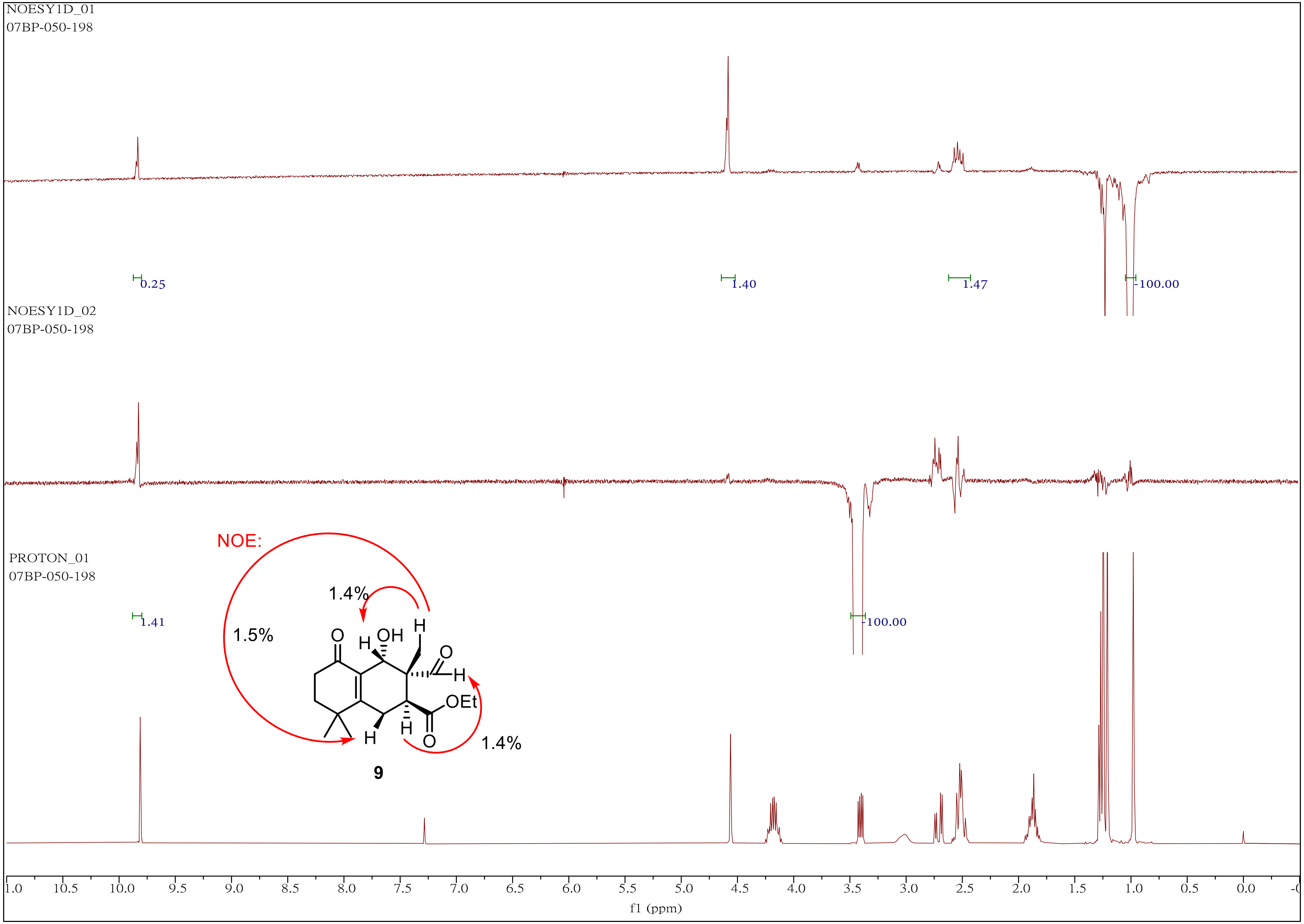


1D NOESY spectra for compound **9**

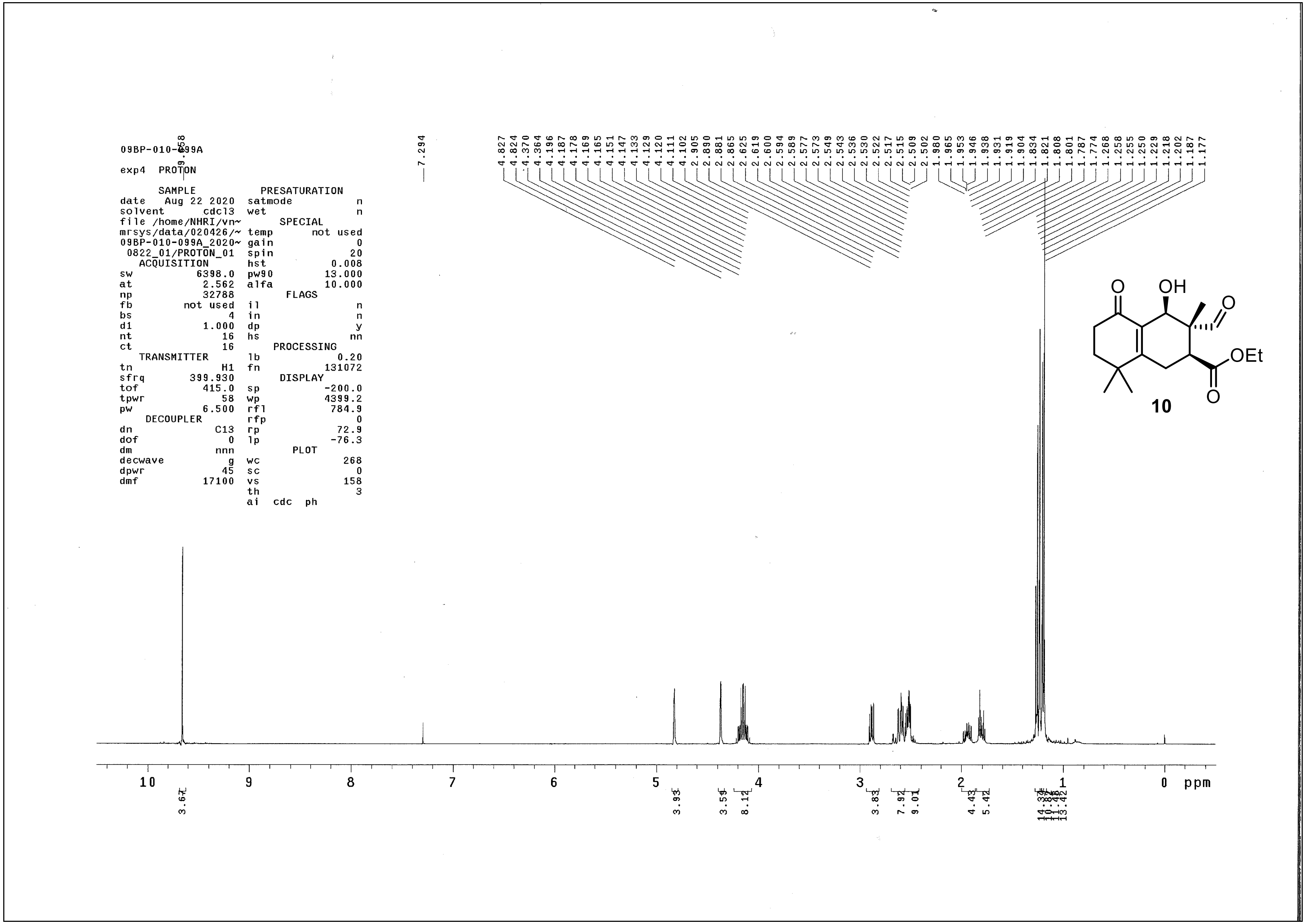


$^{1}$H NMR spectrum for compound **10**

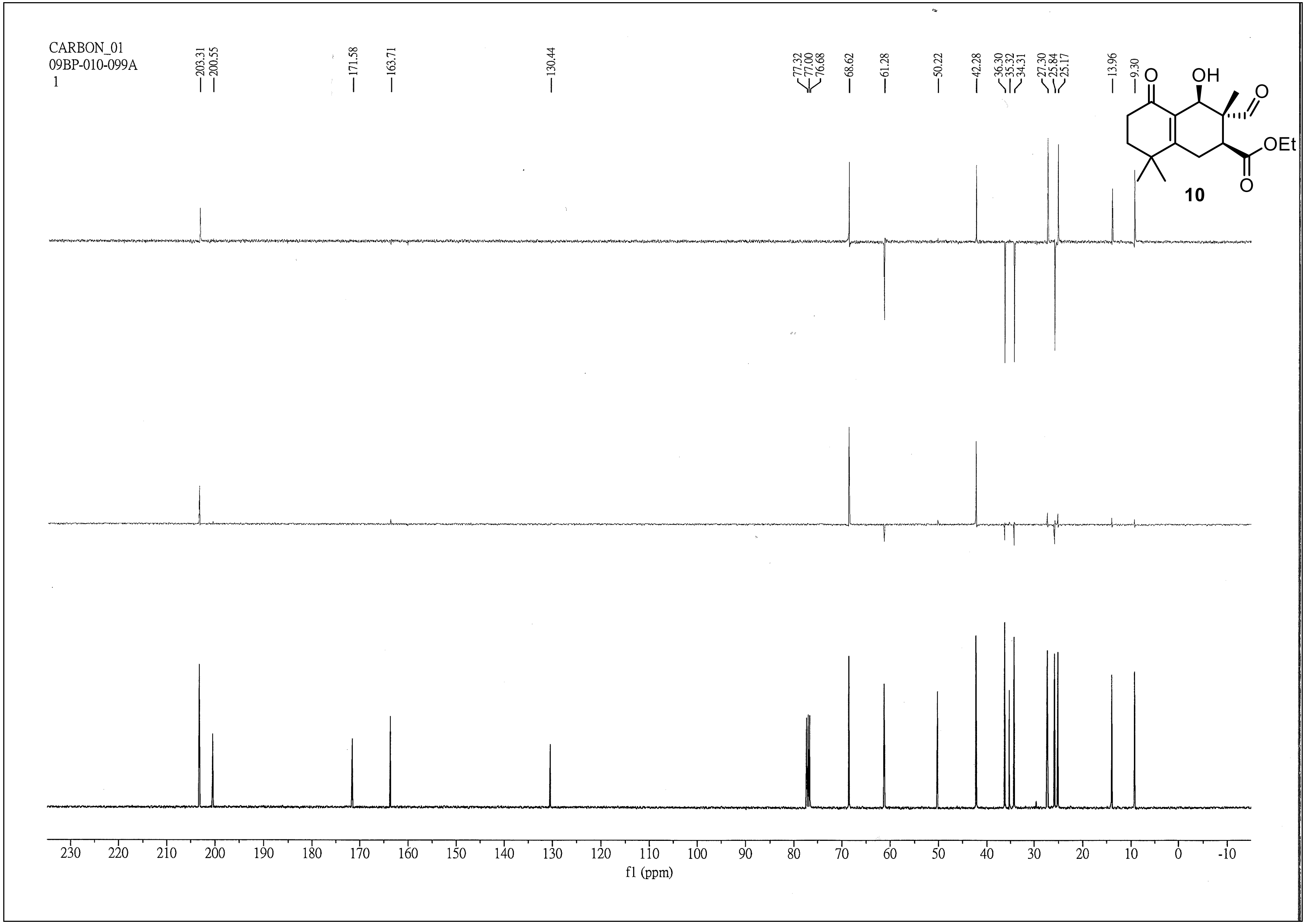


$^{13}C$ NMR + DEPT spectra for compound **10**

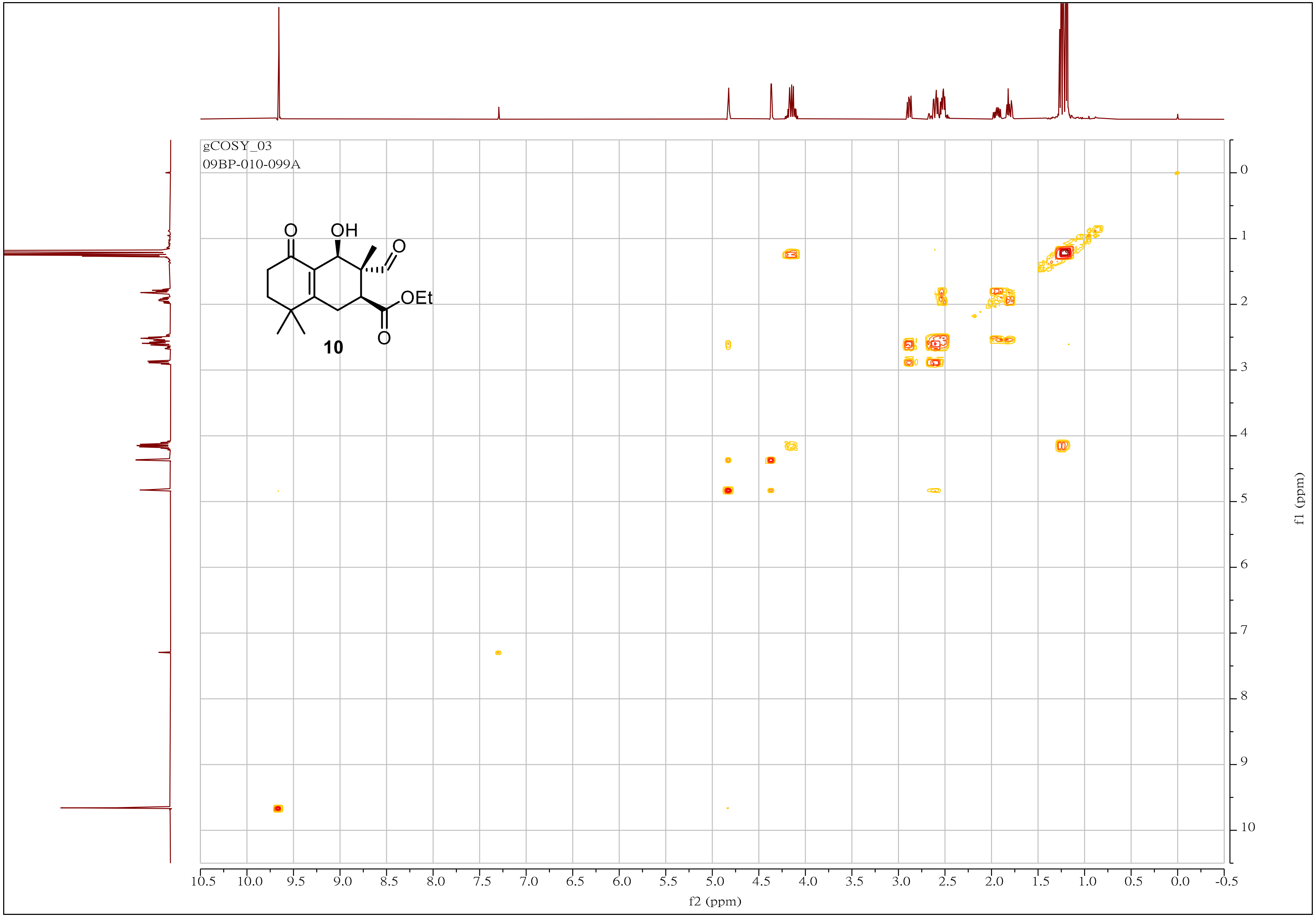


$^{1}$H-$^{1}$H COSY spectrum for compound **10**

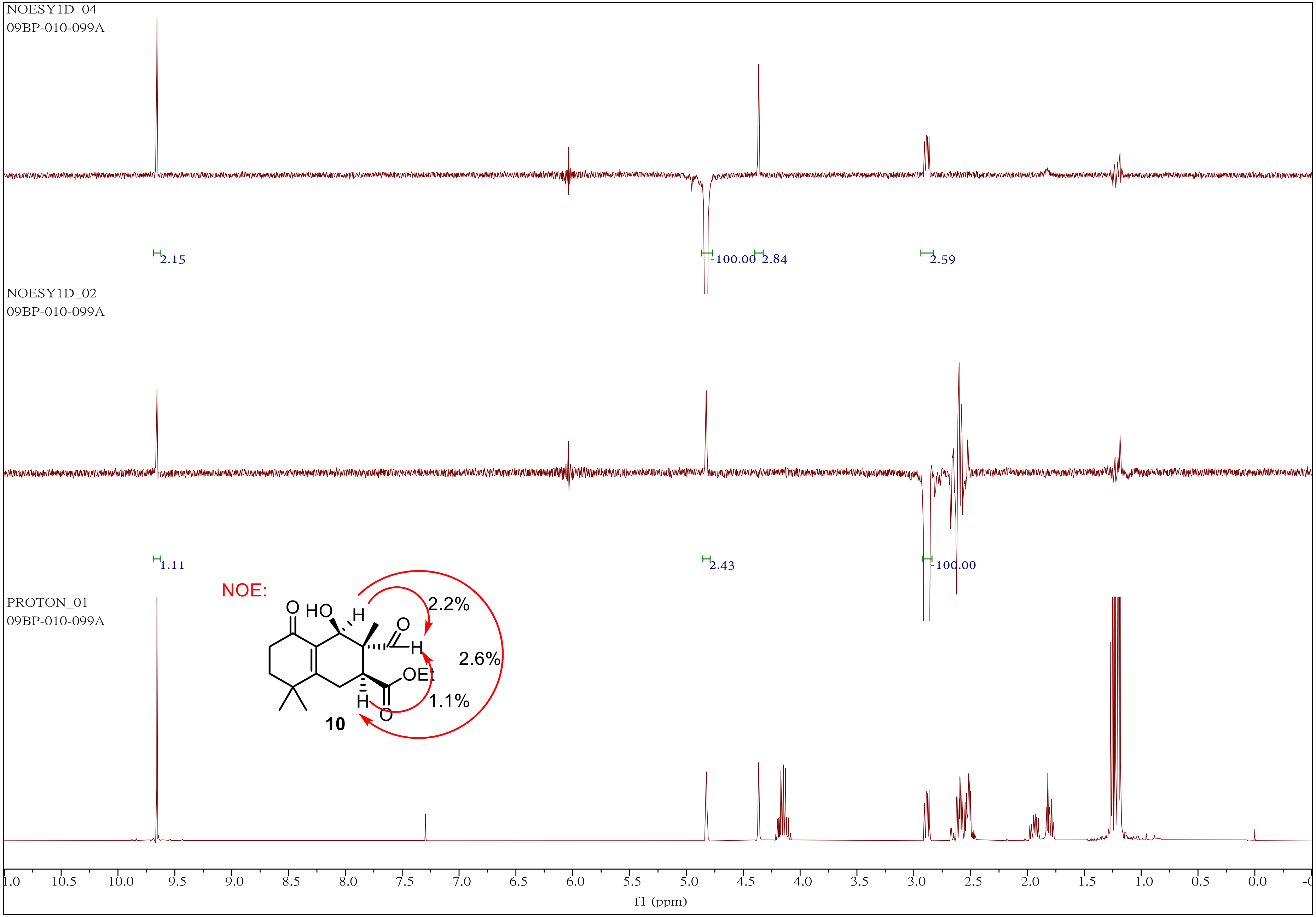


1D NOESY spectra for compound **10**

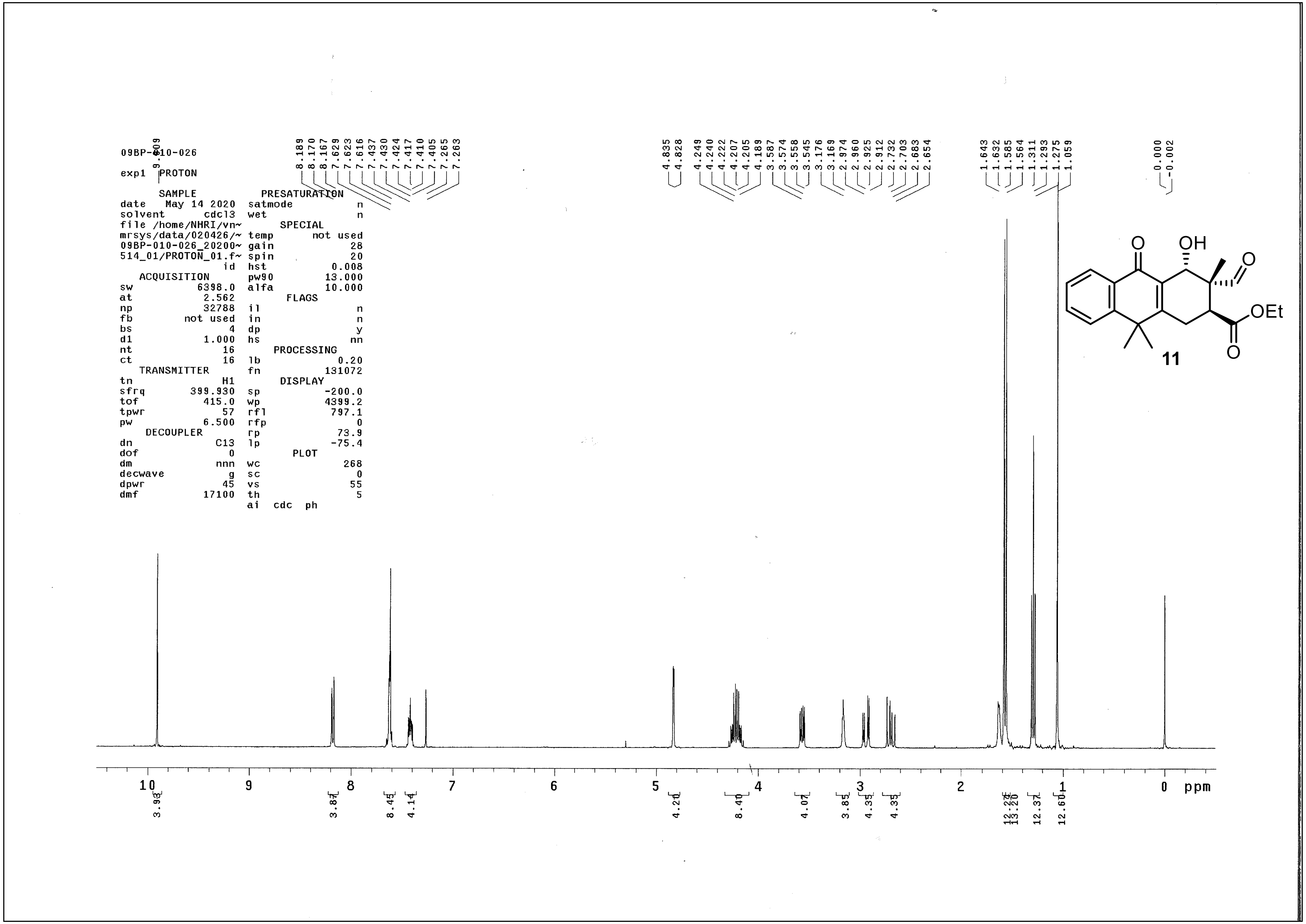

$^{1}$H NMR spectrum for compound **11**

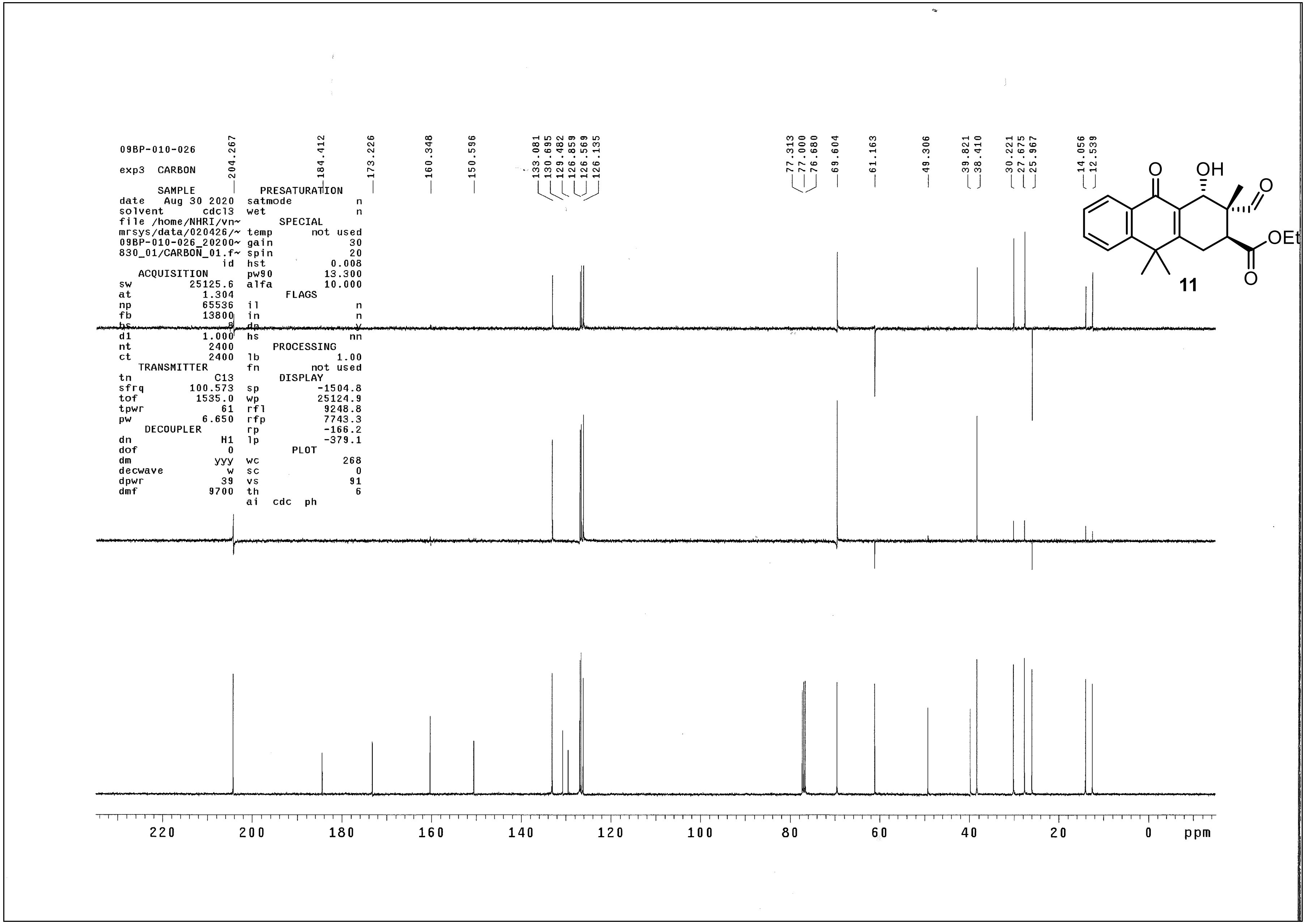


$^{13}$C NMR + DEPT spectra for compound **11**

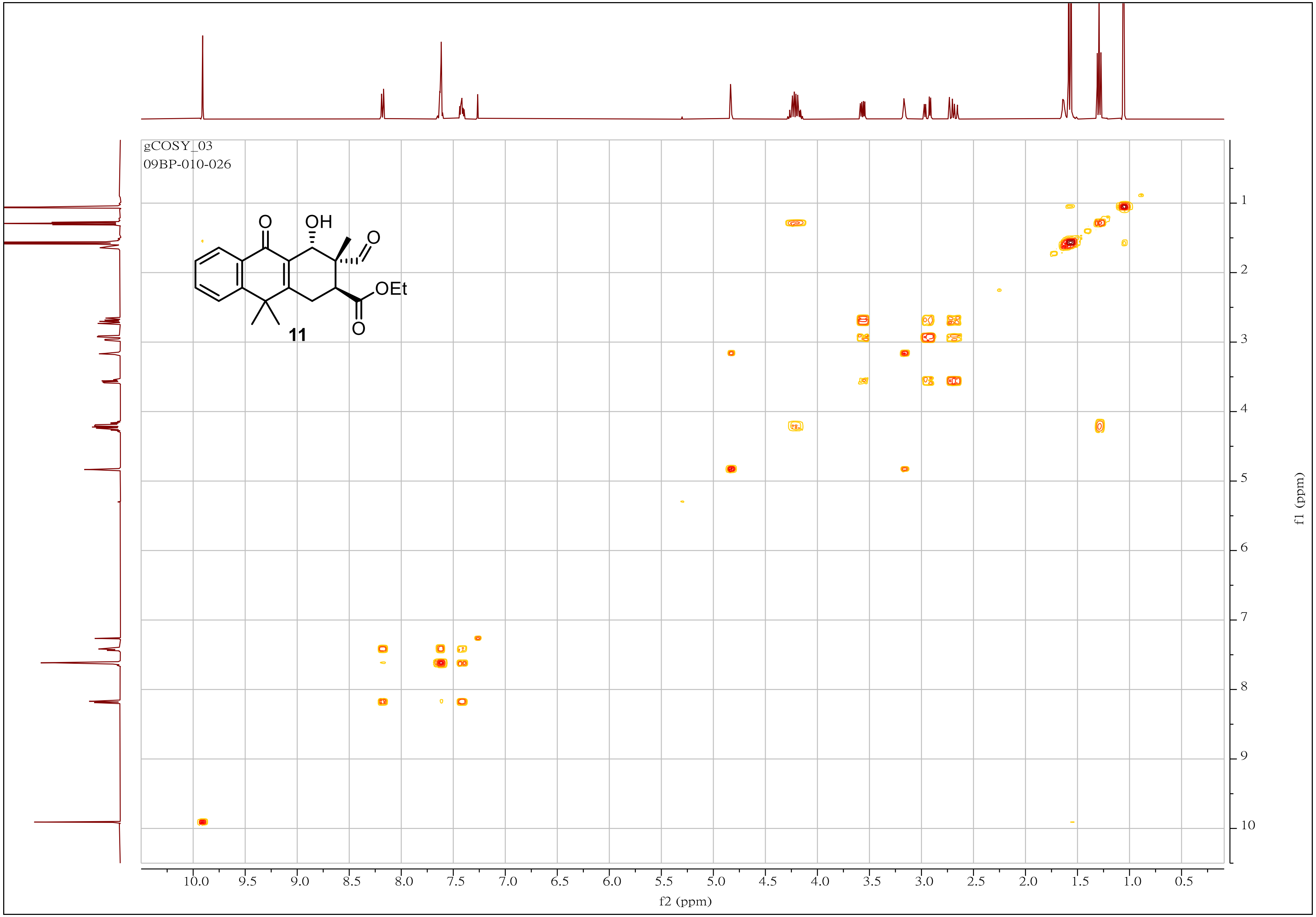


$^1$H-$^1$H COSY spectrum for compound **11**

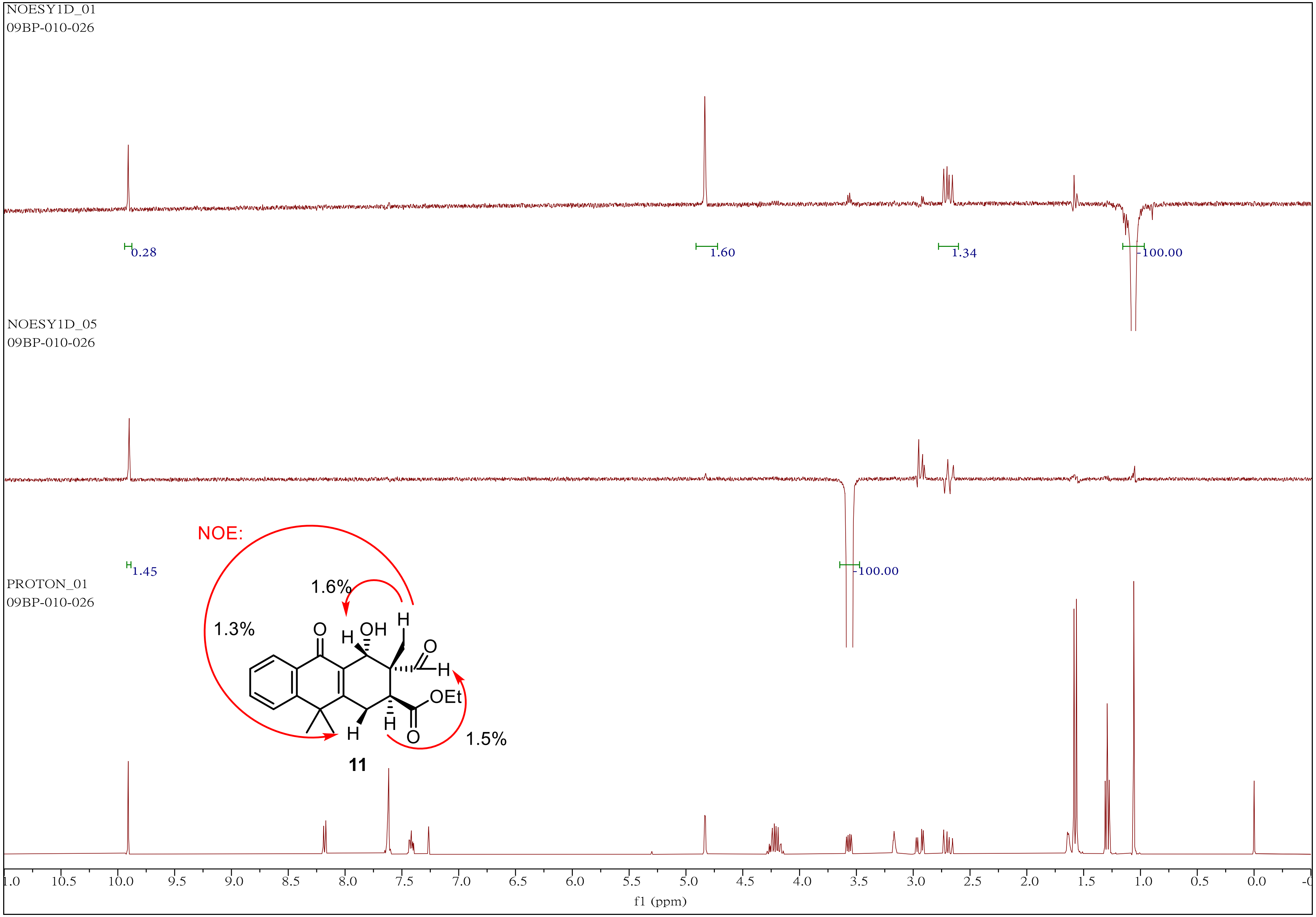


1D NOESY spectra for compound **11**

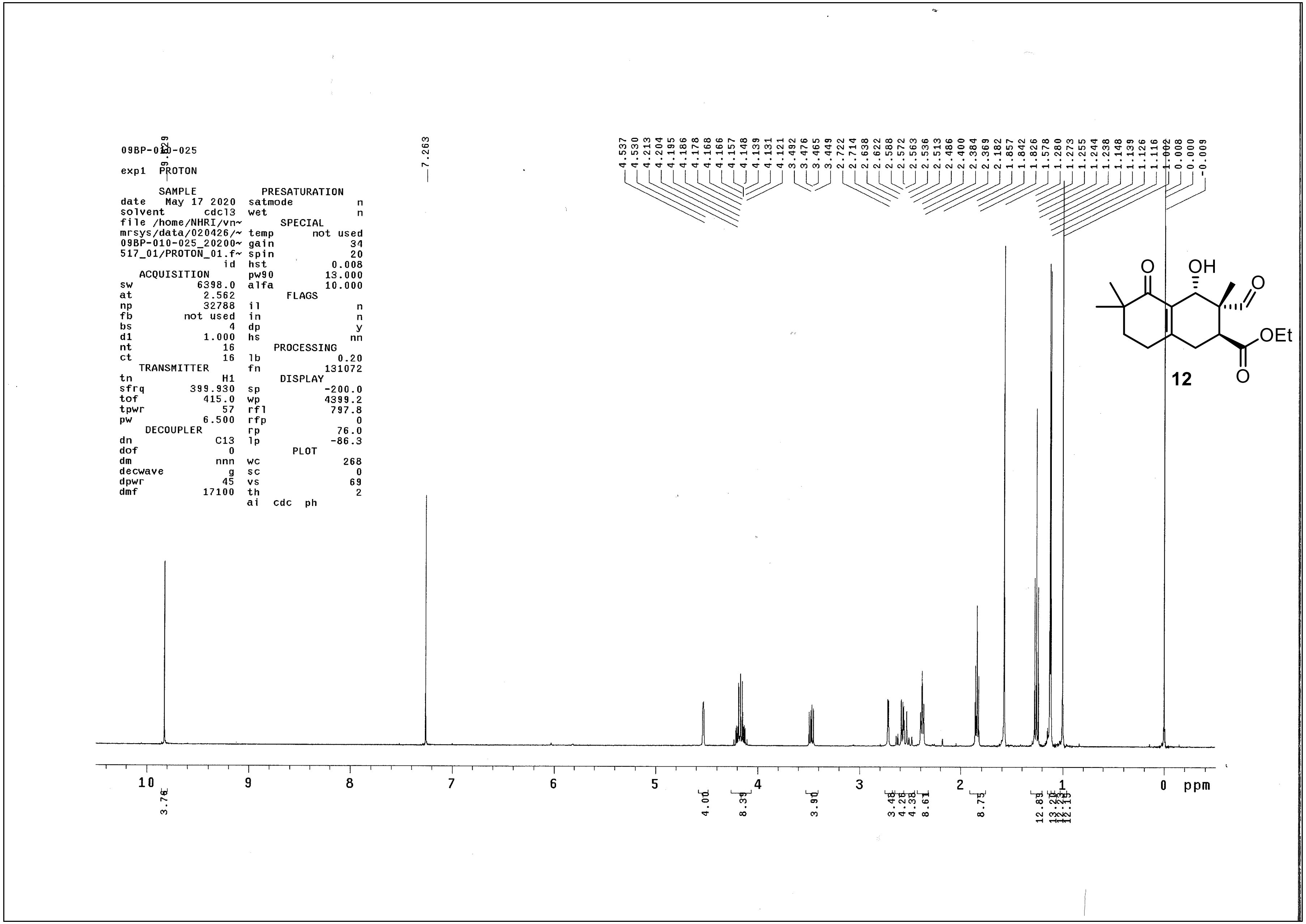

$^{1}$H NMR spectrum for compound **12**

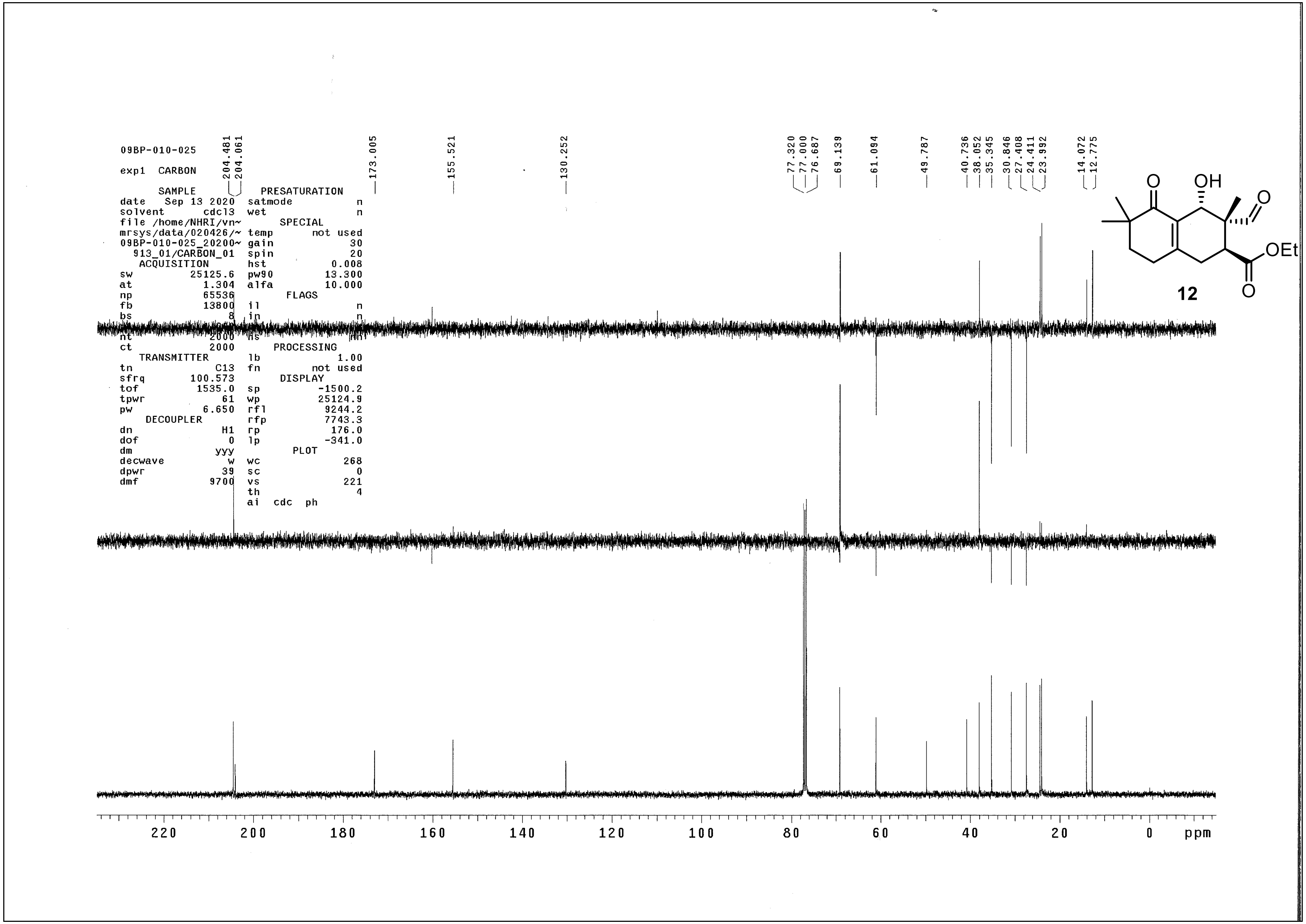

$^{13}$C NMR + DEPT spectra for compound **12**

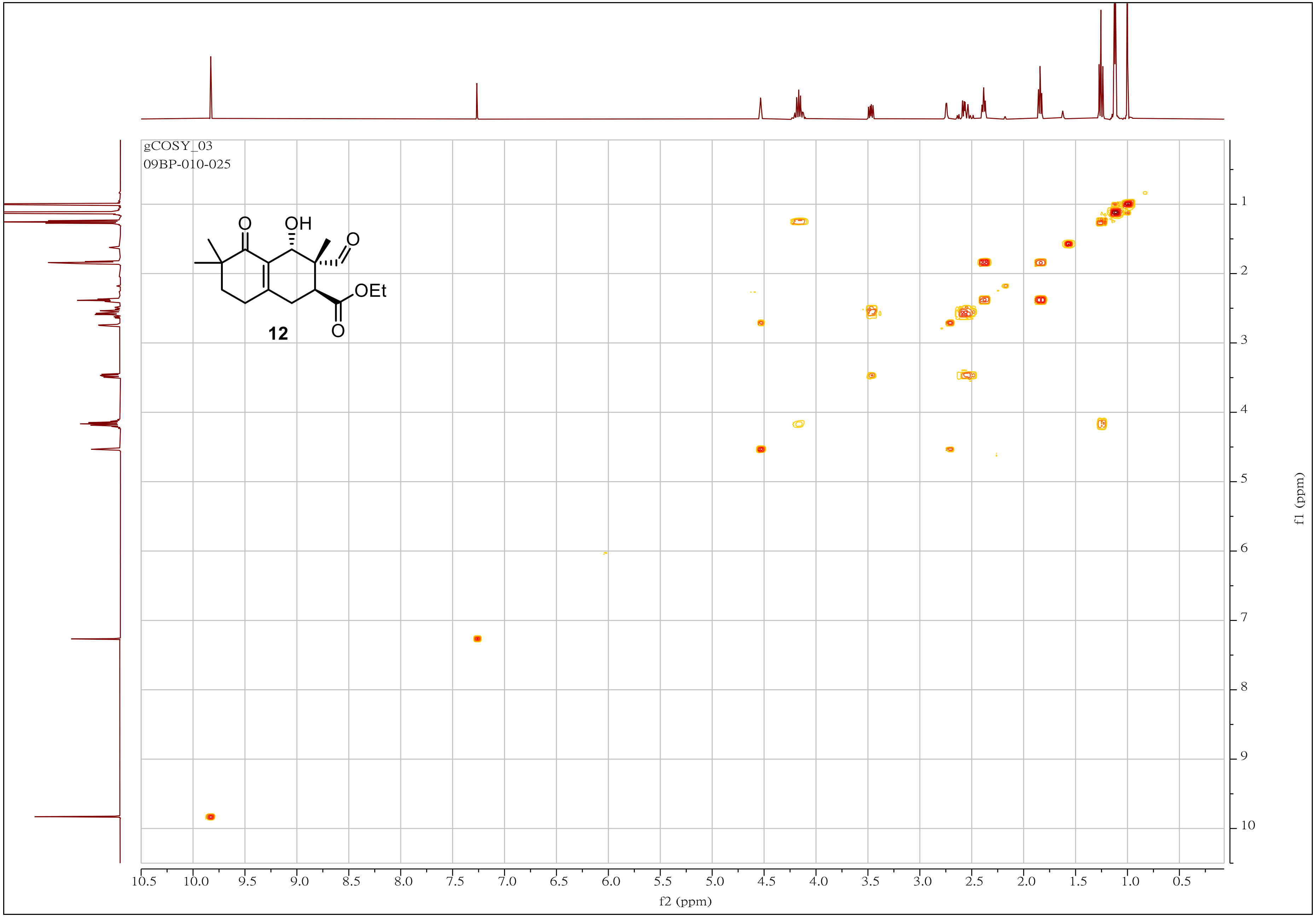


$^1H$-$^1H$ COSY spectrum for compound **12**

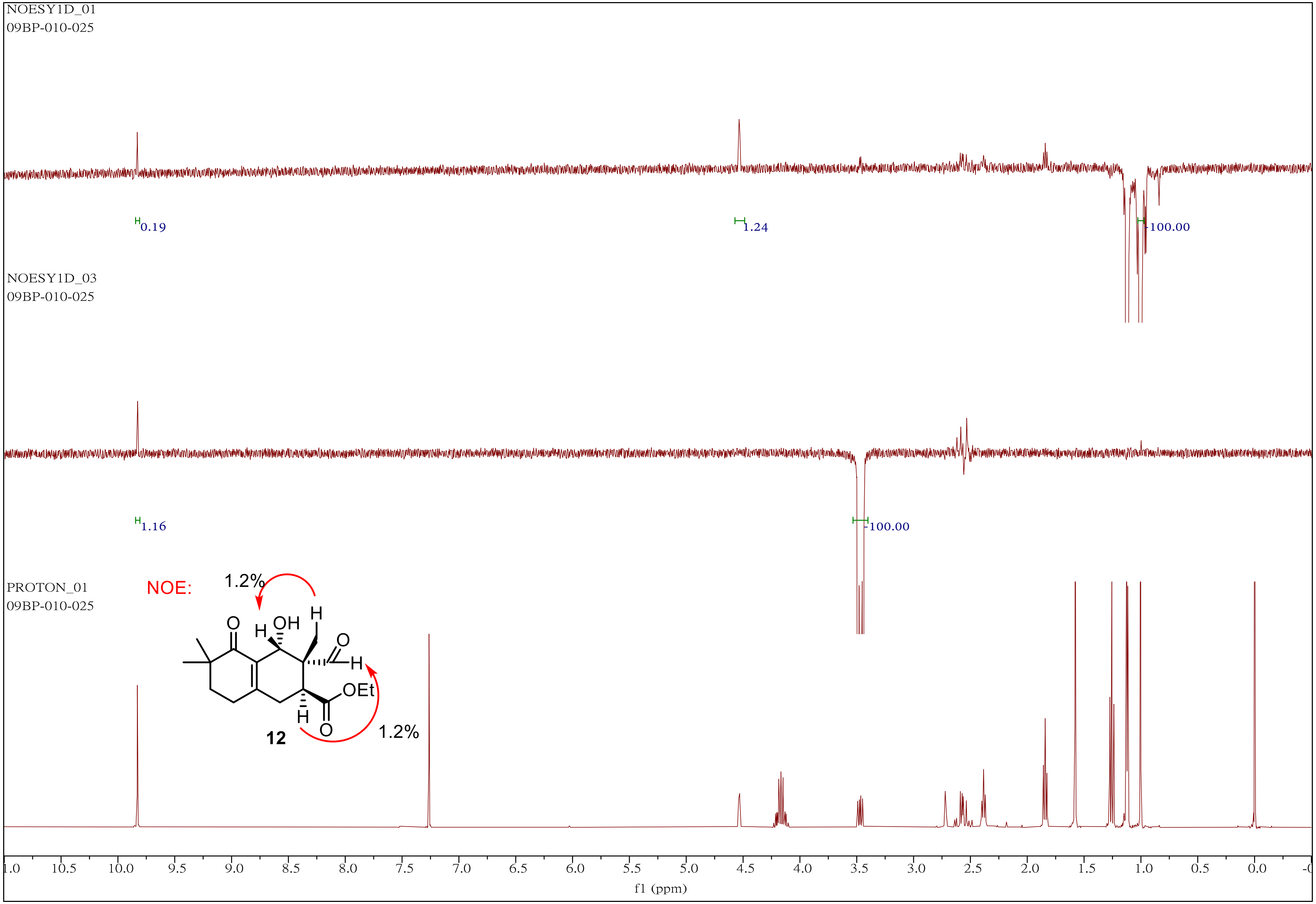


1D NOESY spectra for compound **12**

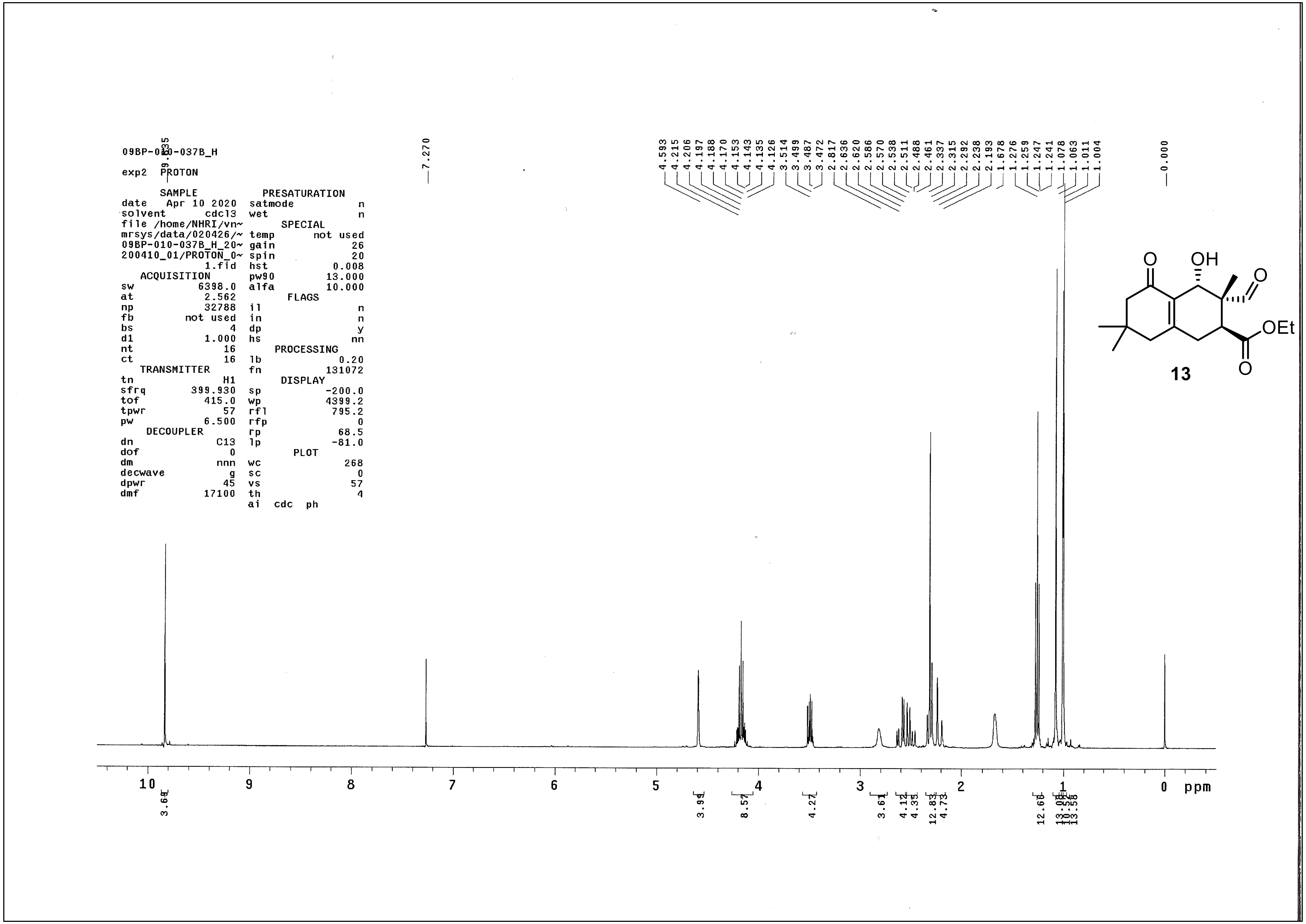


$^{1}$H NMR spectrum for compound **13**

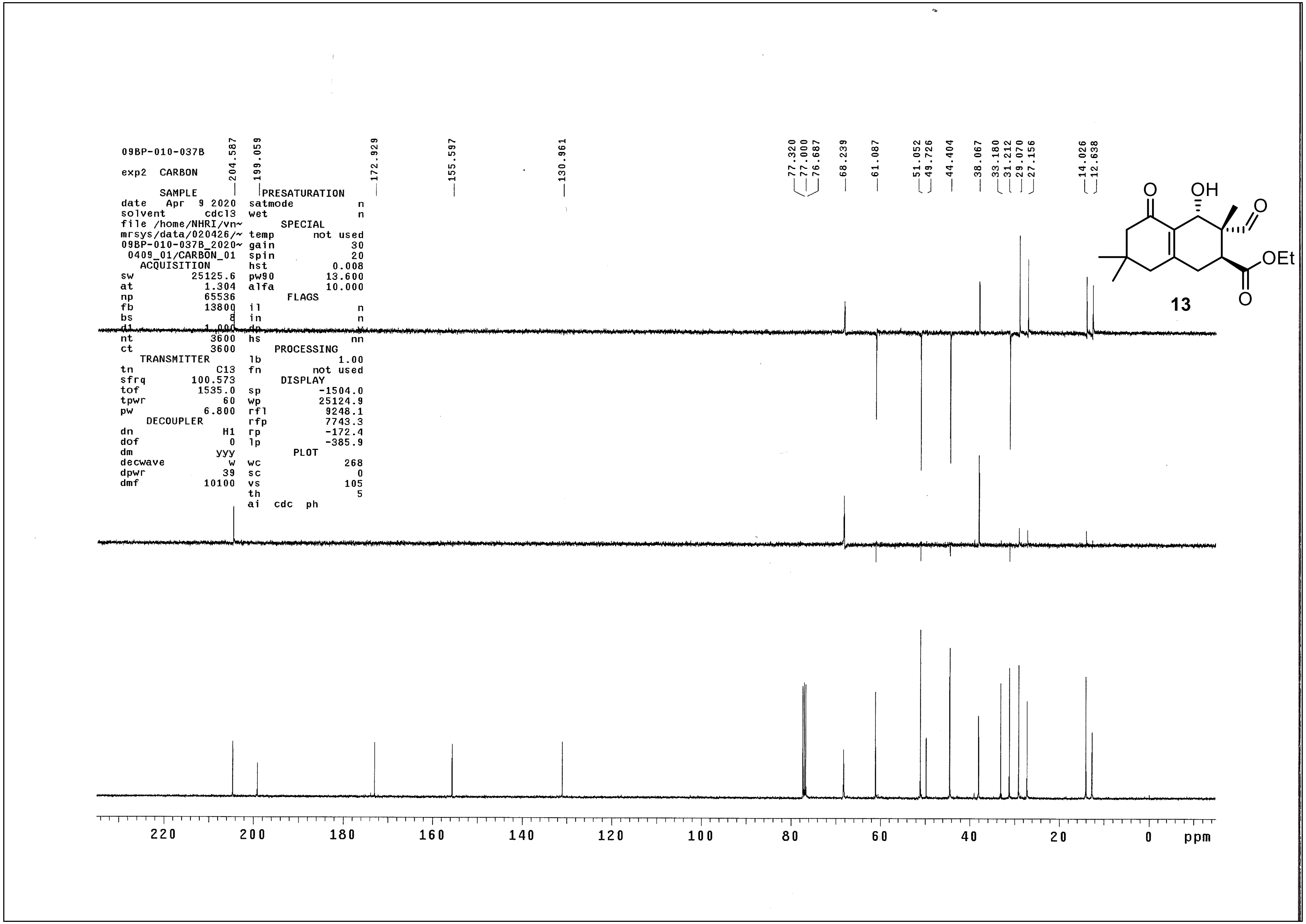

$^{13}$C NMR + DEPT spectra for compound **13**

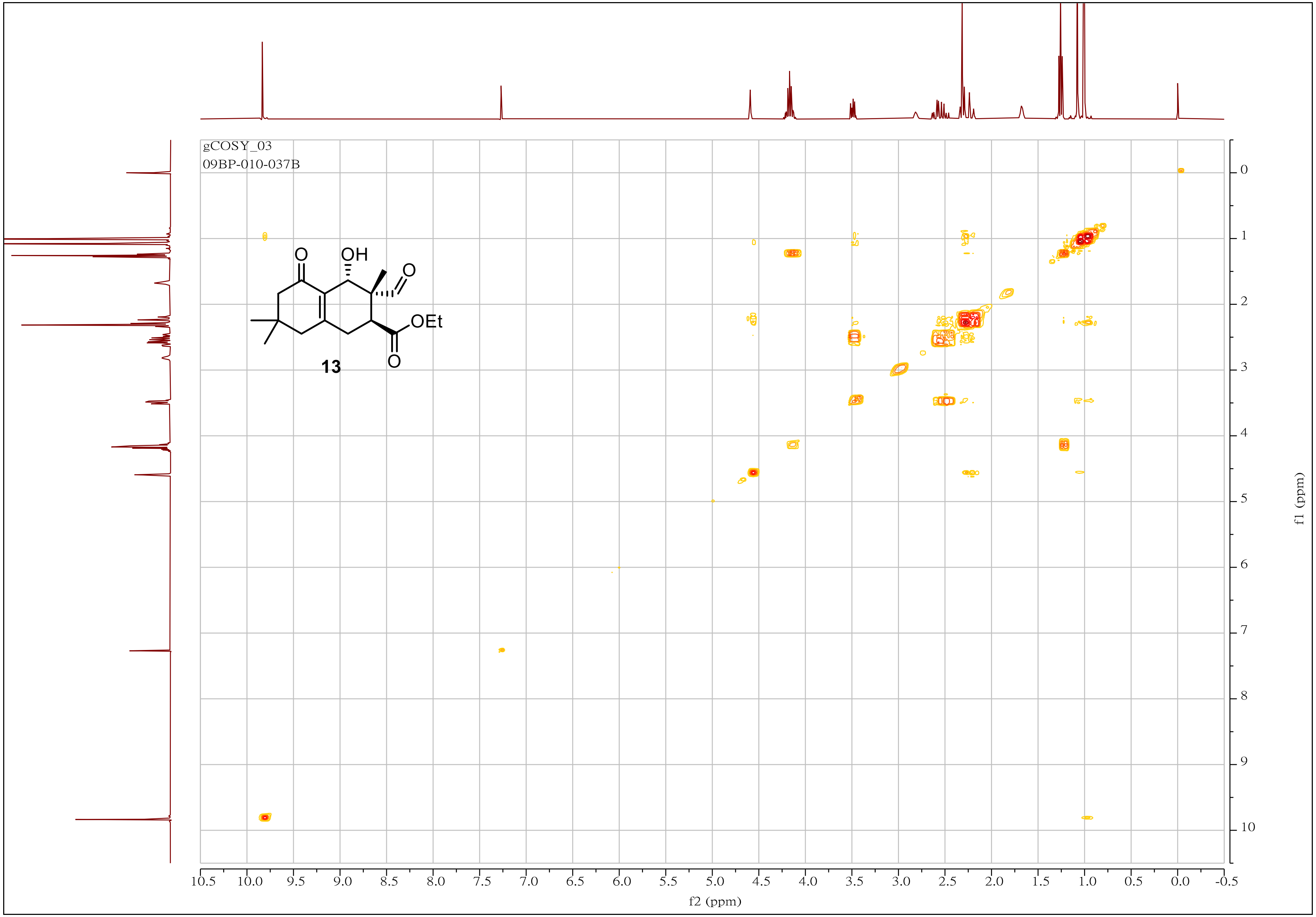


$^{1}$H-$^{1}$H COSY spectrum for compound **13**

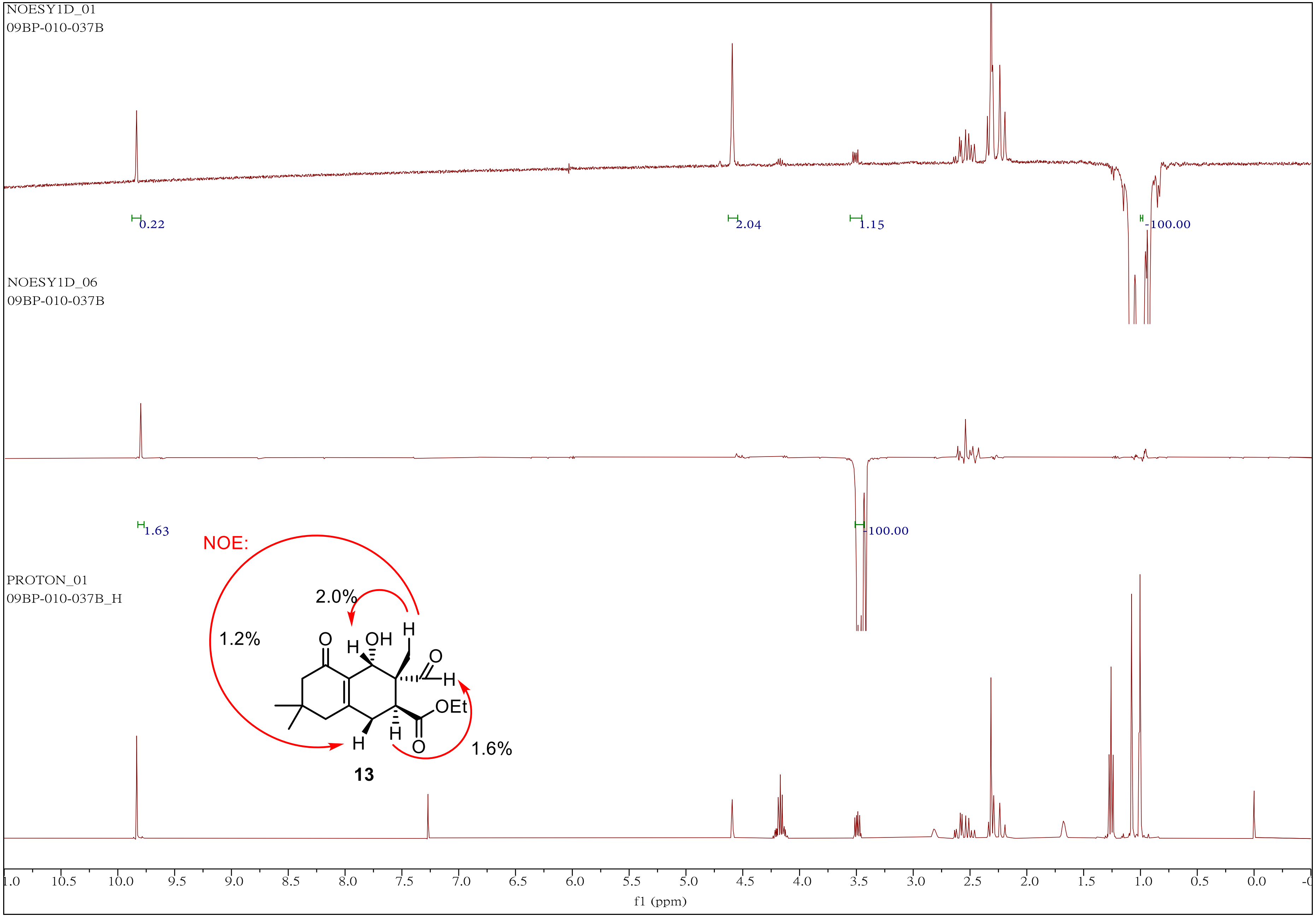


1D NOESY spectra for compound **13**

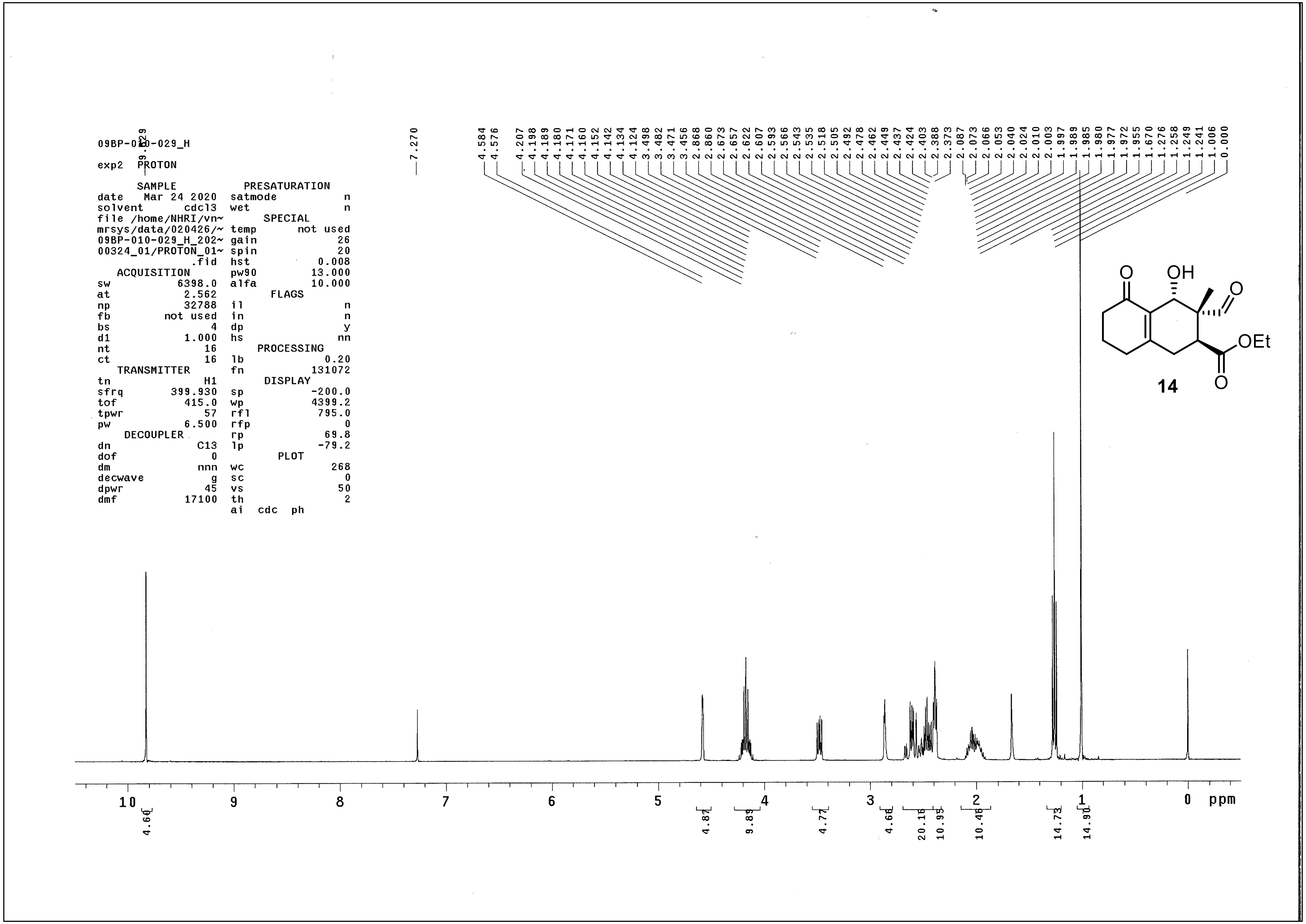


$^{1}$H NMR spectrum for compound **14**

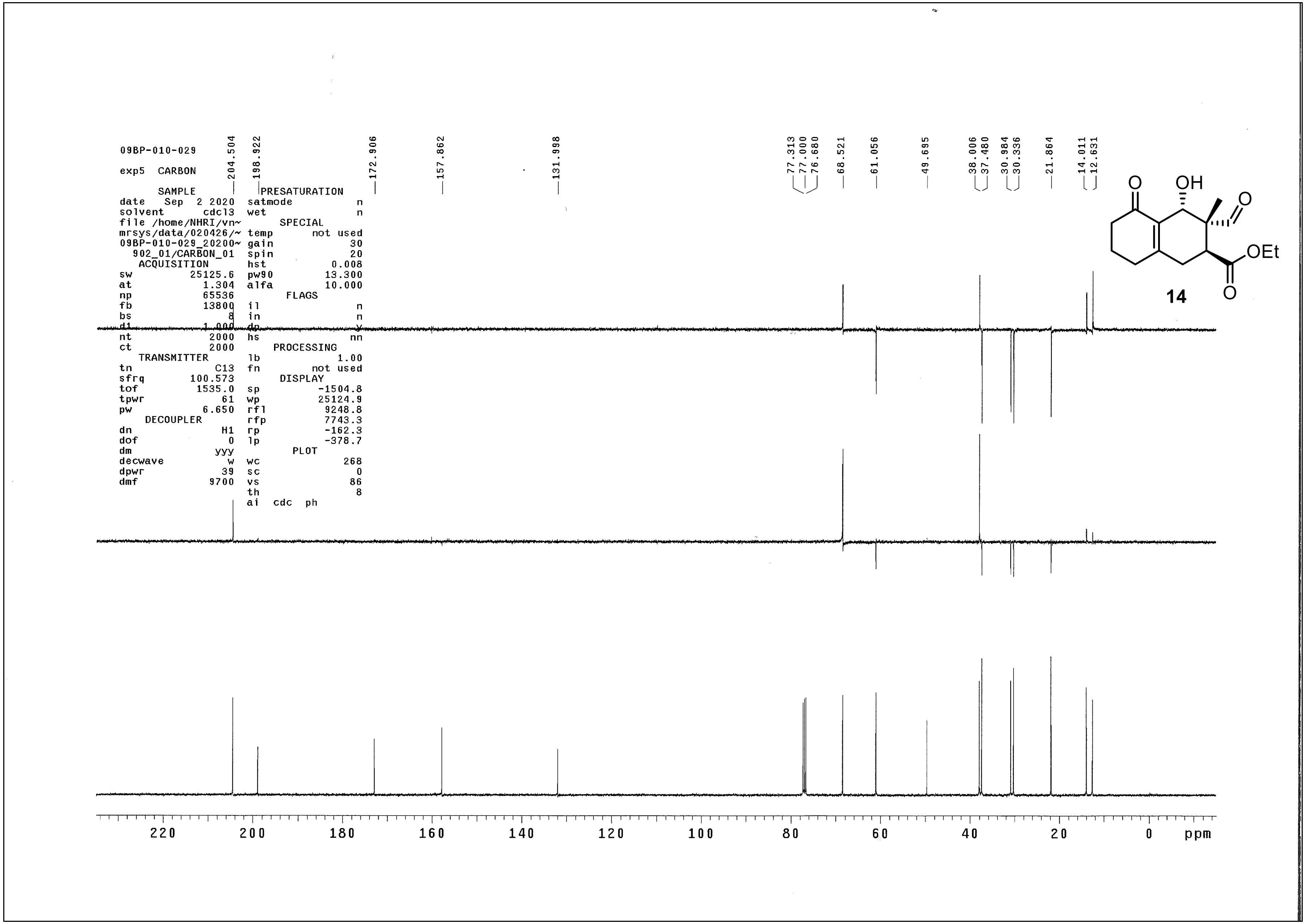

$^{13}$C NMR + DEPT spectra for compound **14**

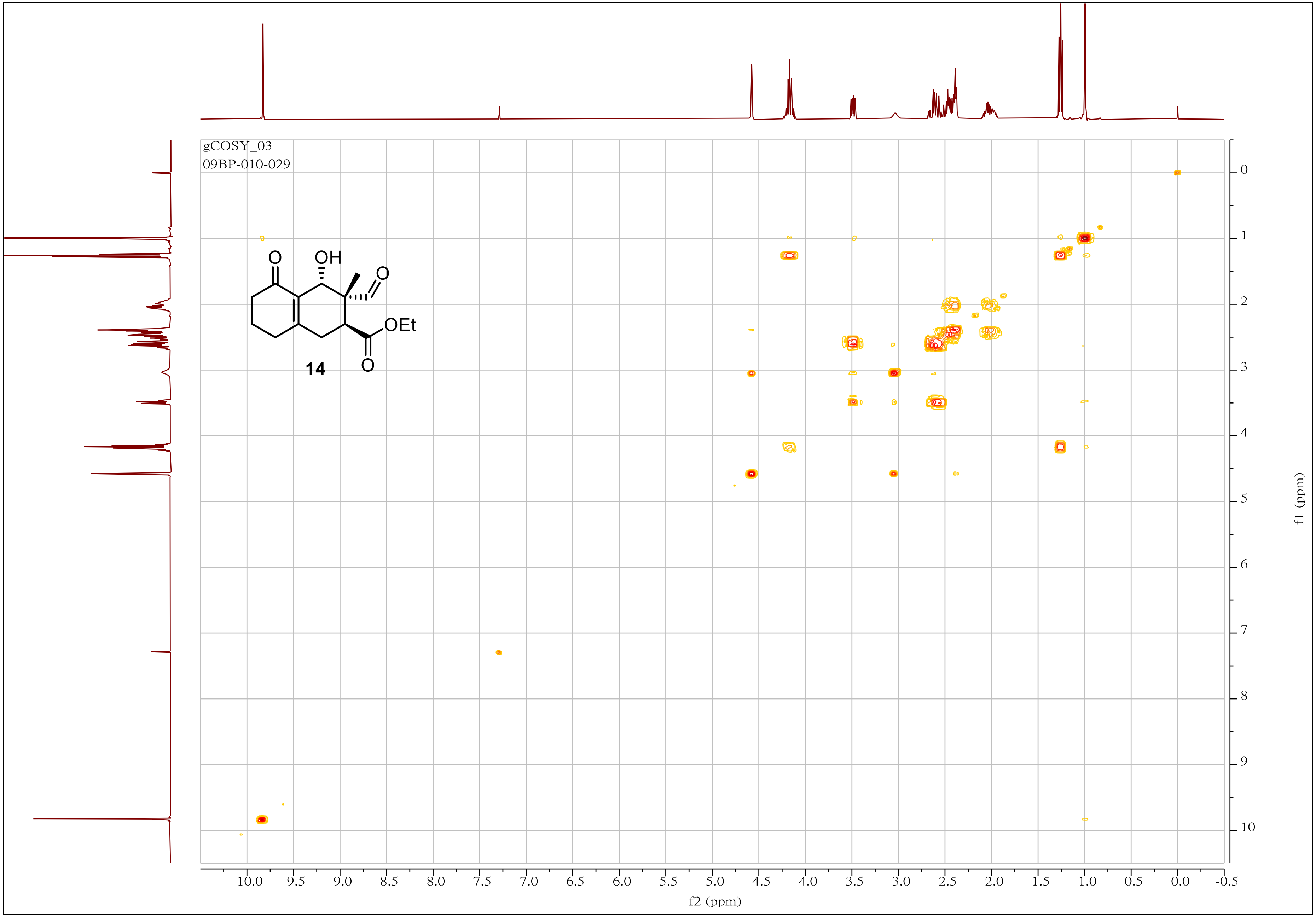


$^{1}$H-$^{1}$H COSY spectrum for compound **14**

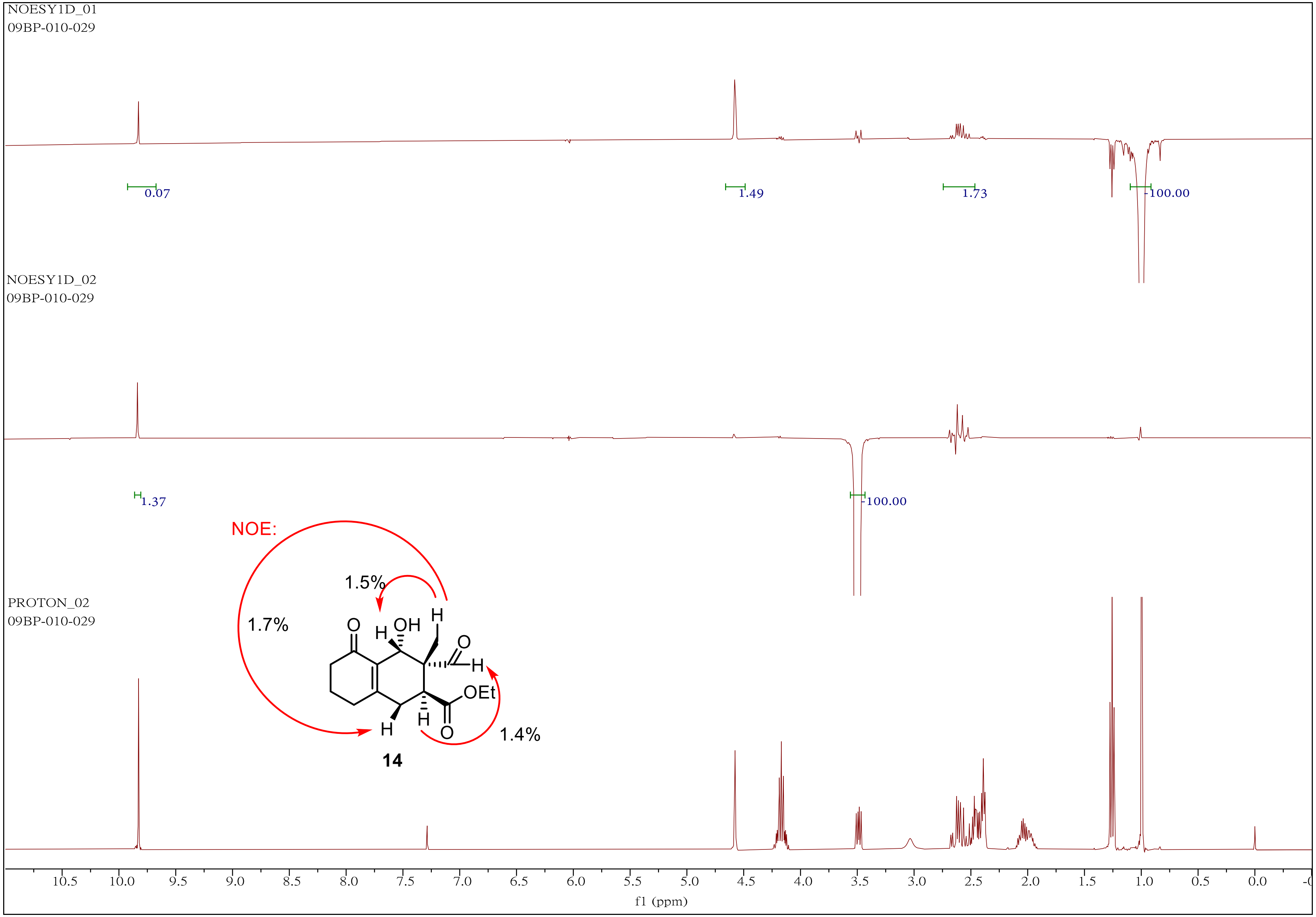


1D NOESY spectra for compound **14**

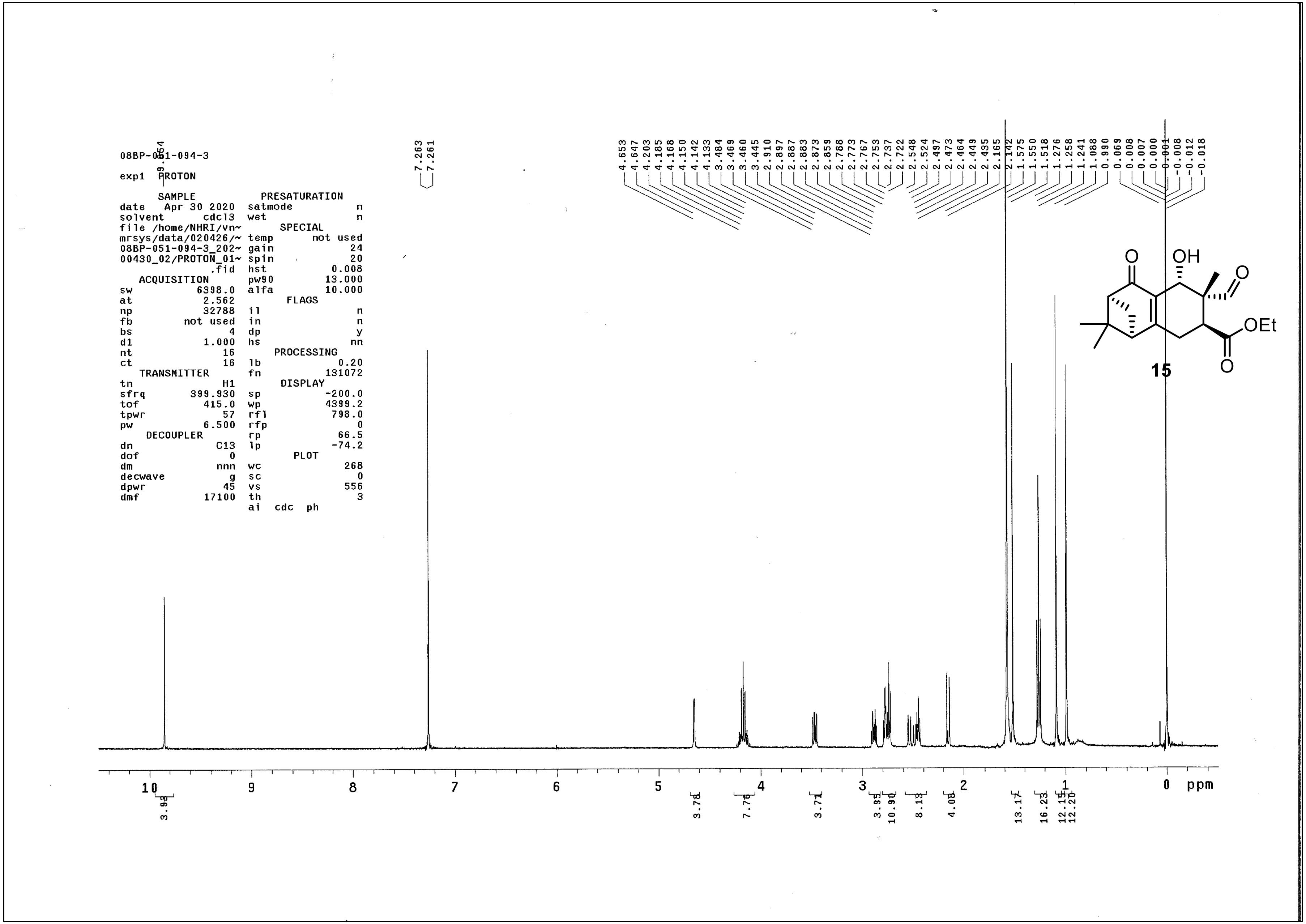

$^{1}$H NMR spectrum for compound **15**

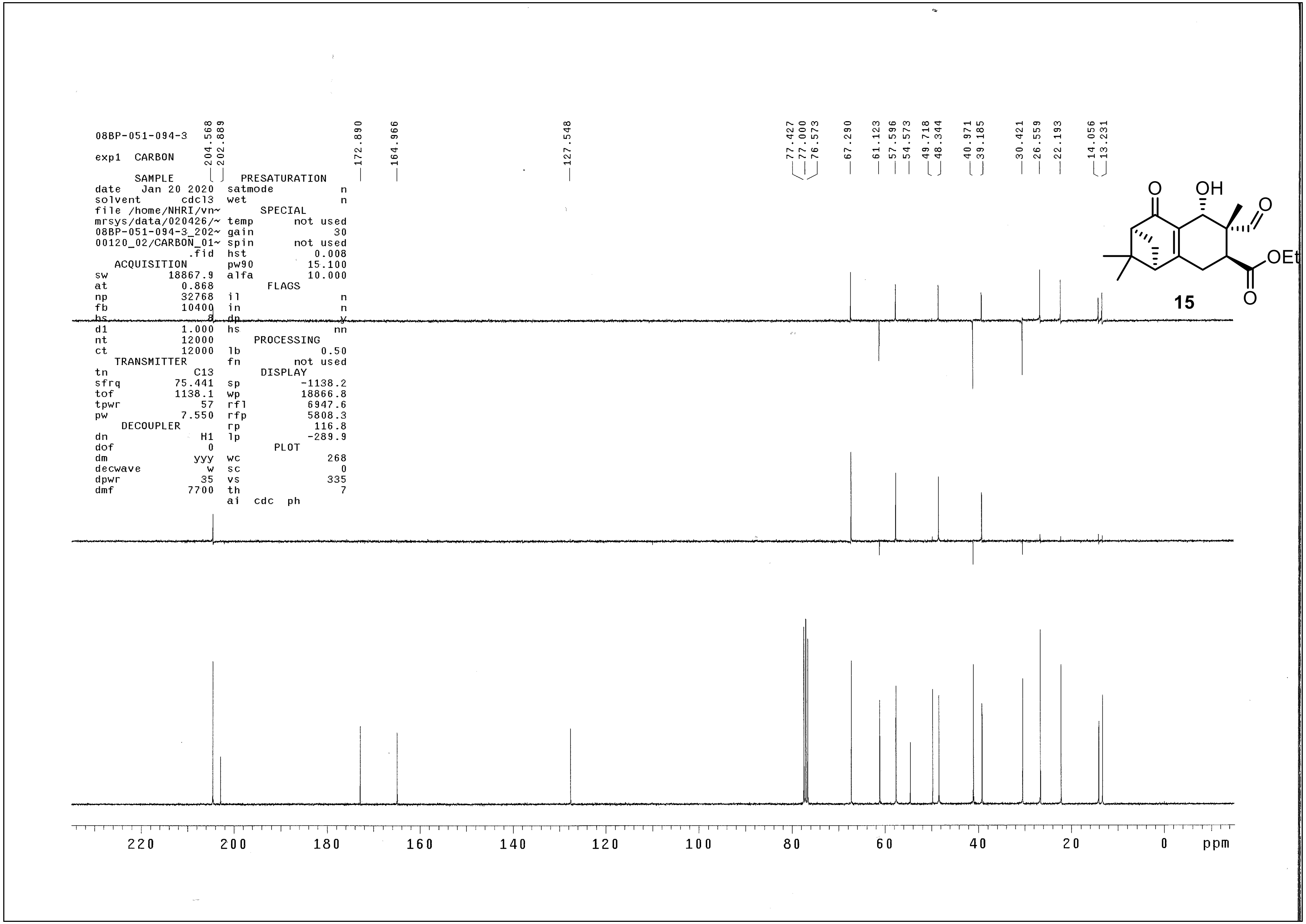

$^{13}$C NMR + DEPT spectra for compound **15**

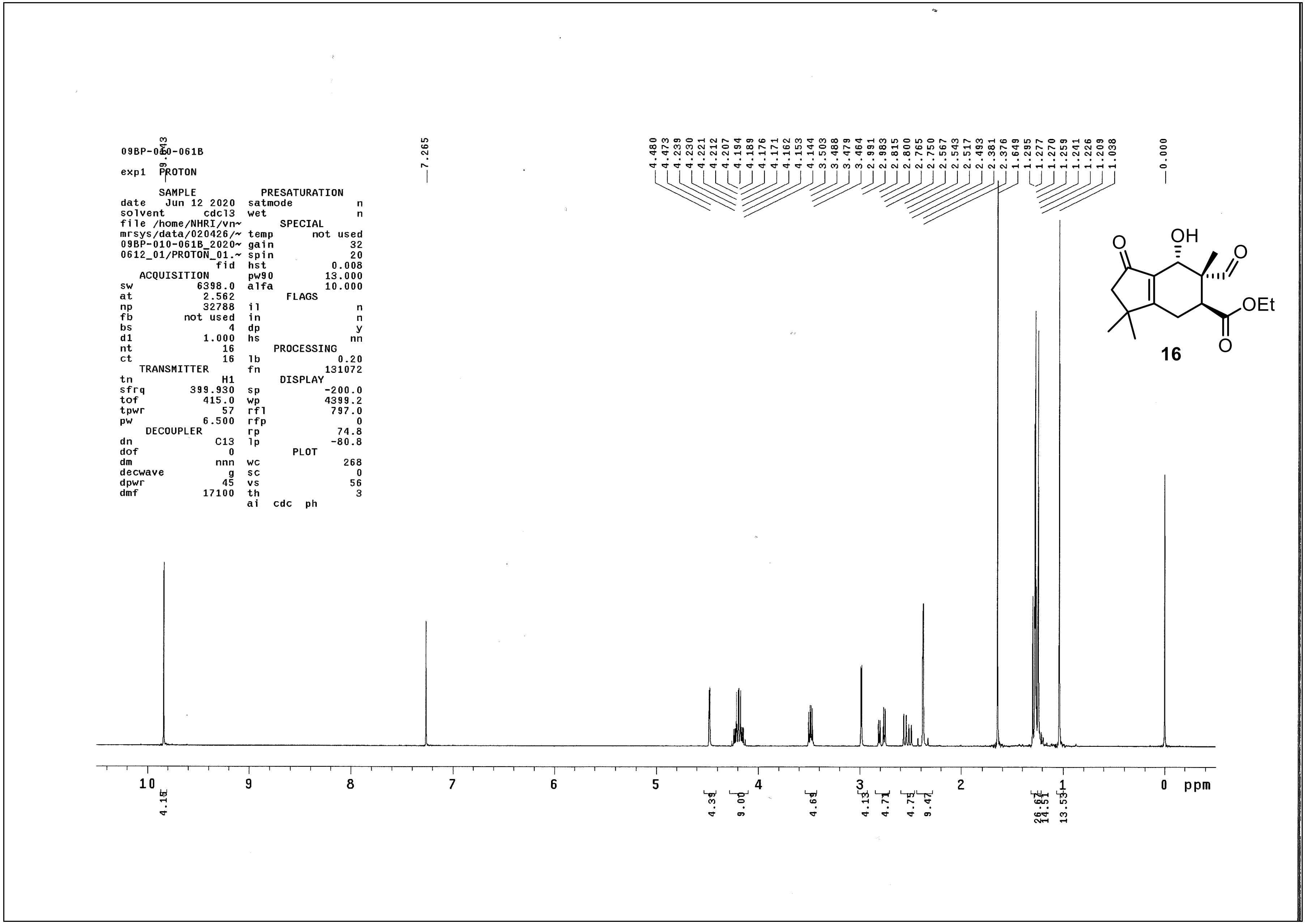

$^{1}$H NMR spectrum for compound **16**

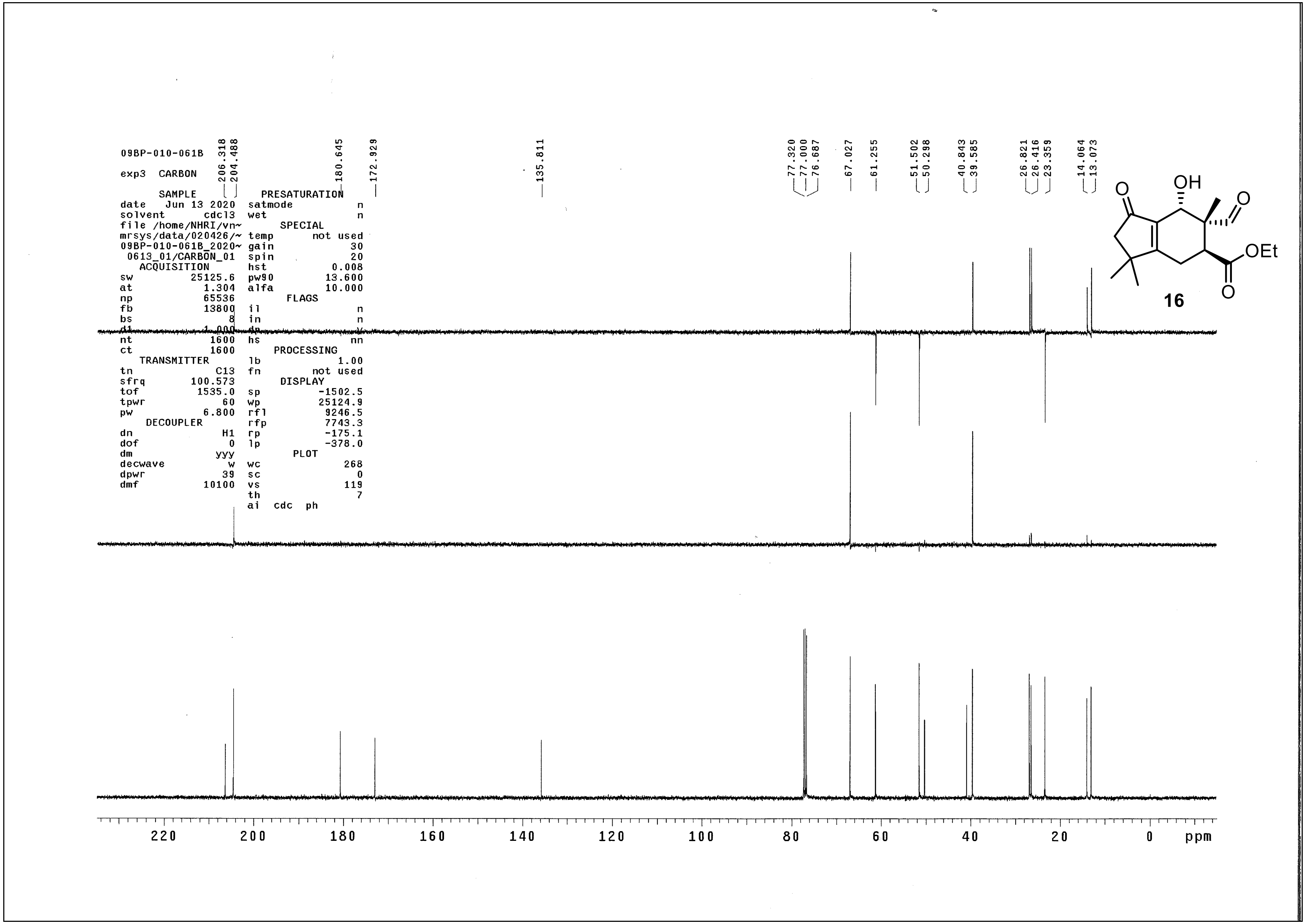

$^{13}$C NMR + DEPT spectra for compound **16**

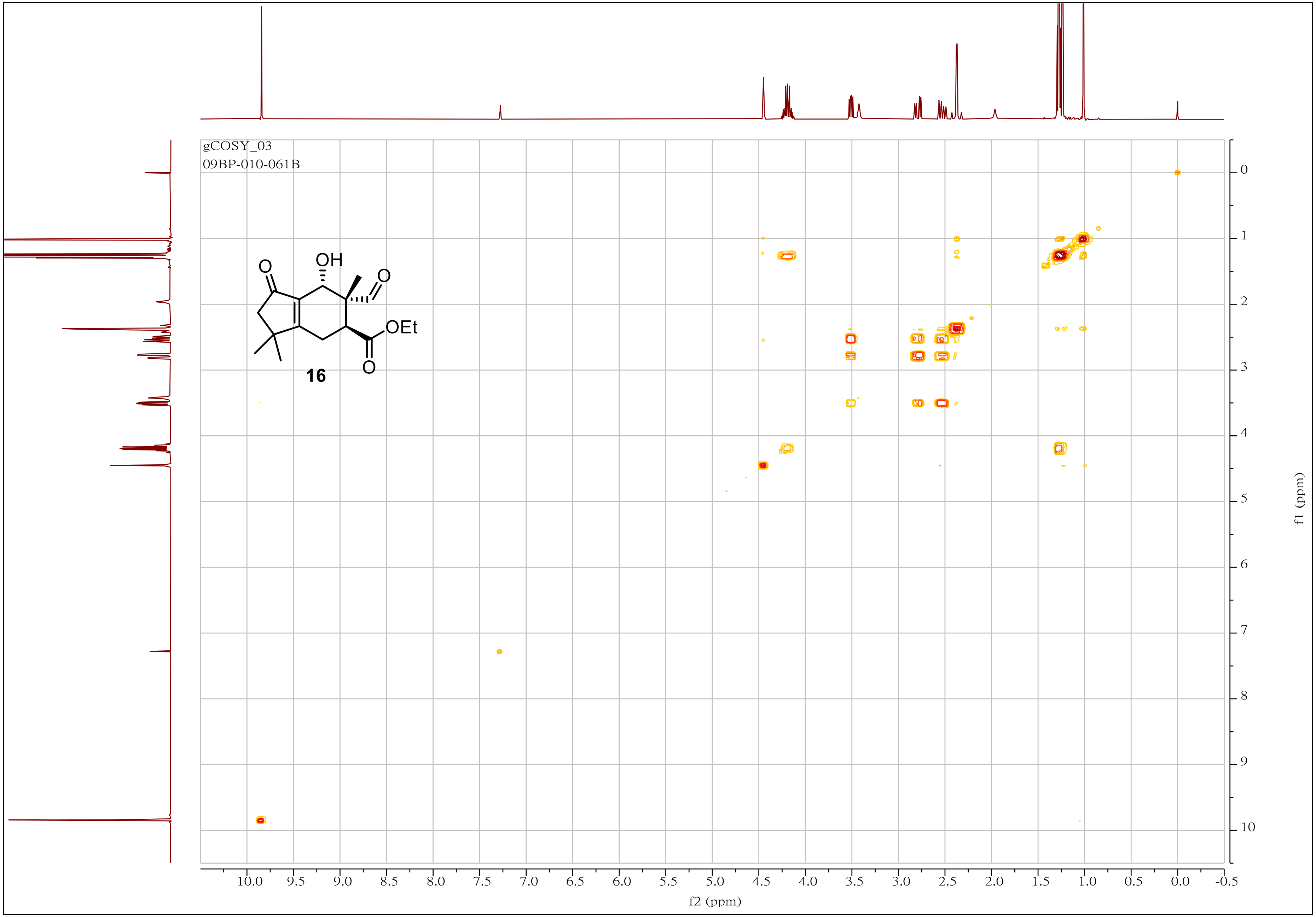


$^1$H-$^1$H COSY spectrum for compound **16**

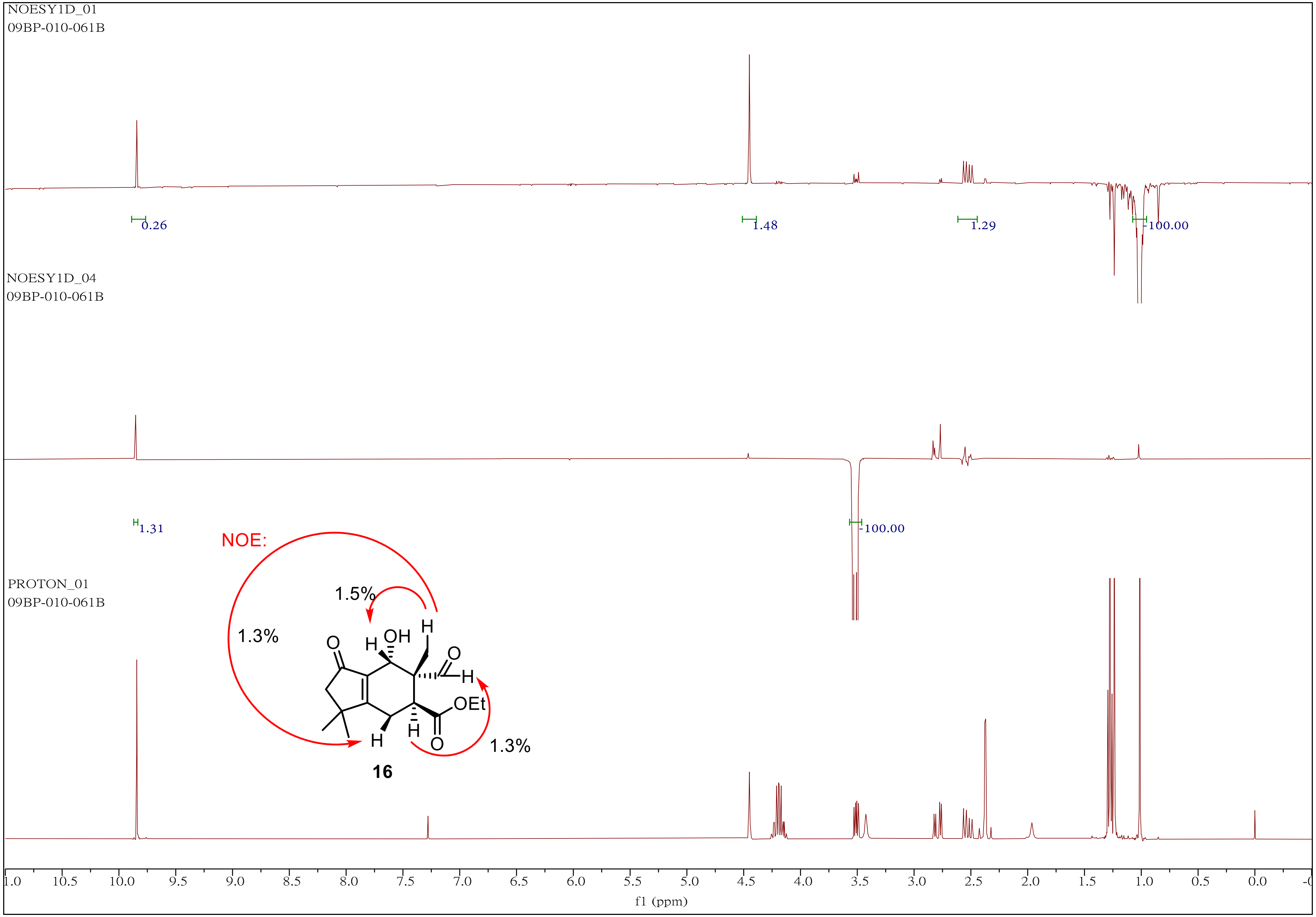


1D NOESY spectra for compound **16**

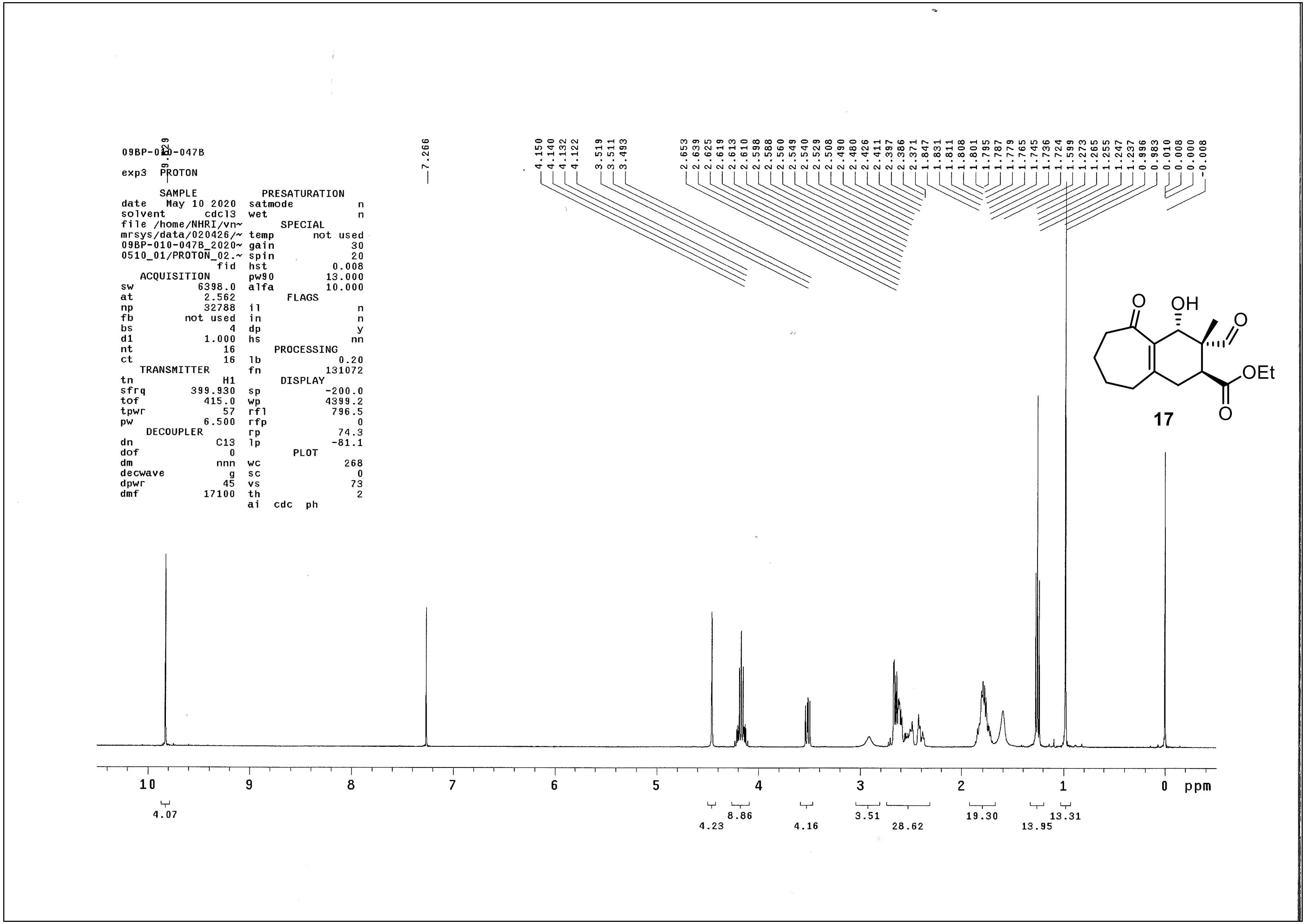

$^{1}$H NMR spectrum for compound **17**

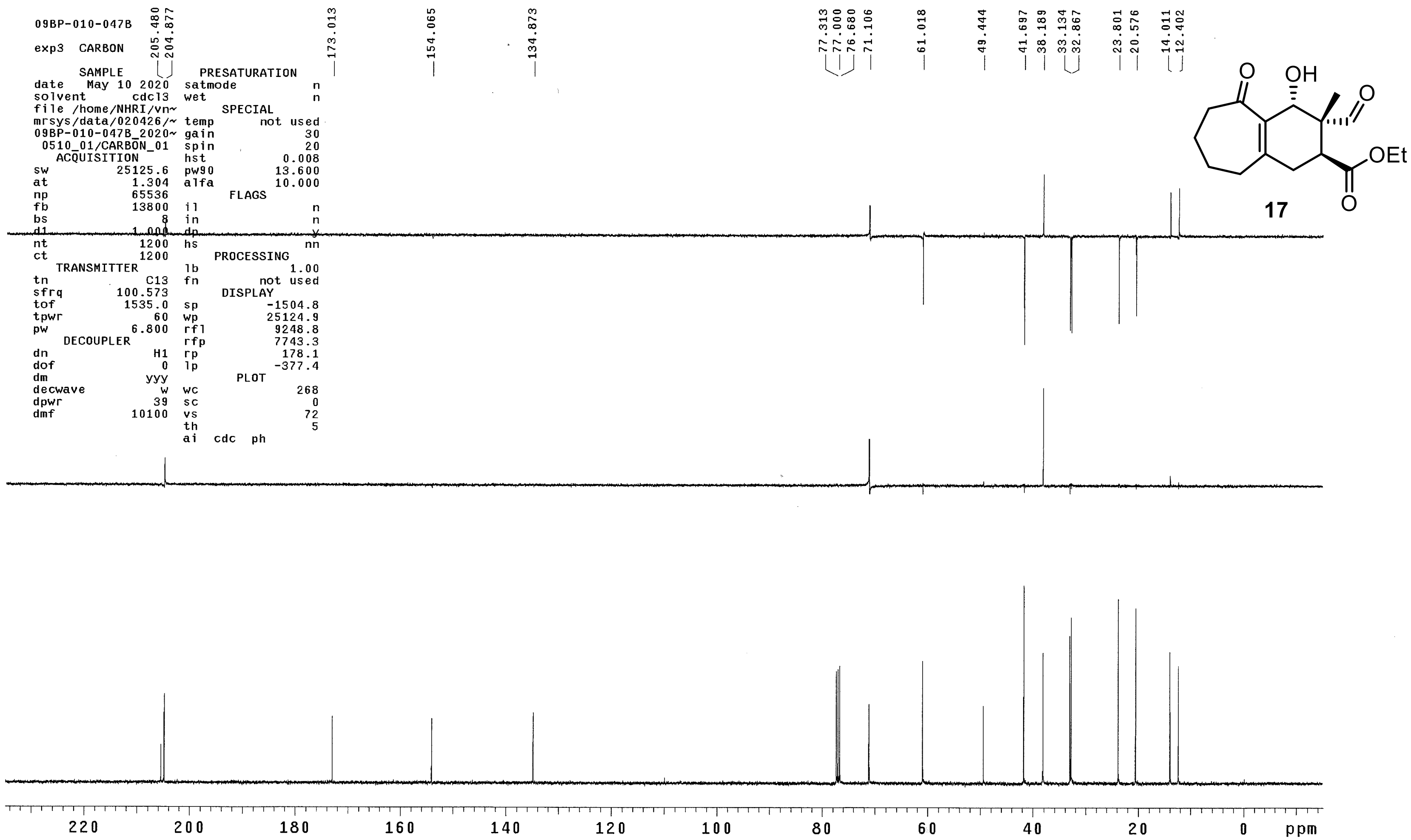


$^{13}$C NMR + DEPT spectra for compound **17**

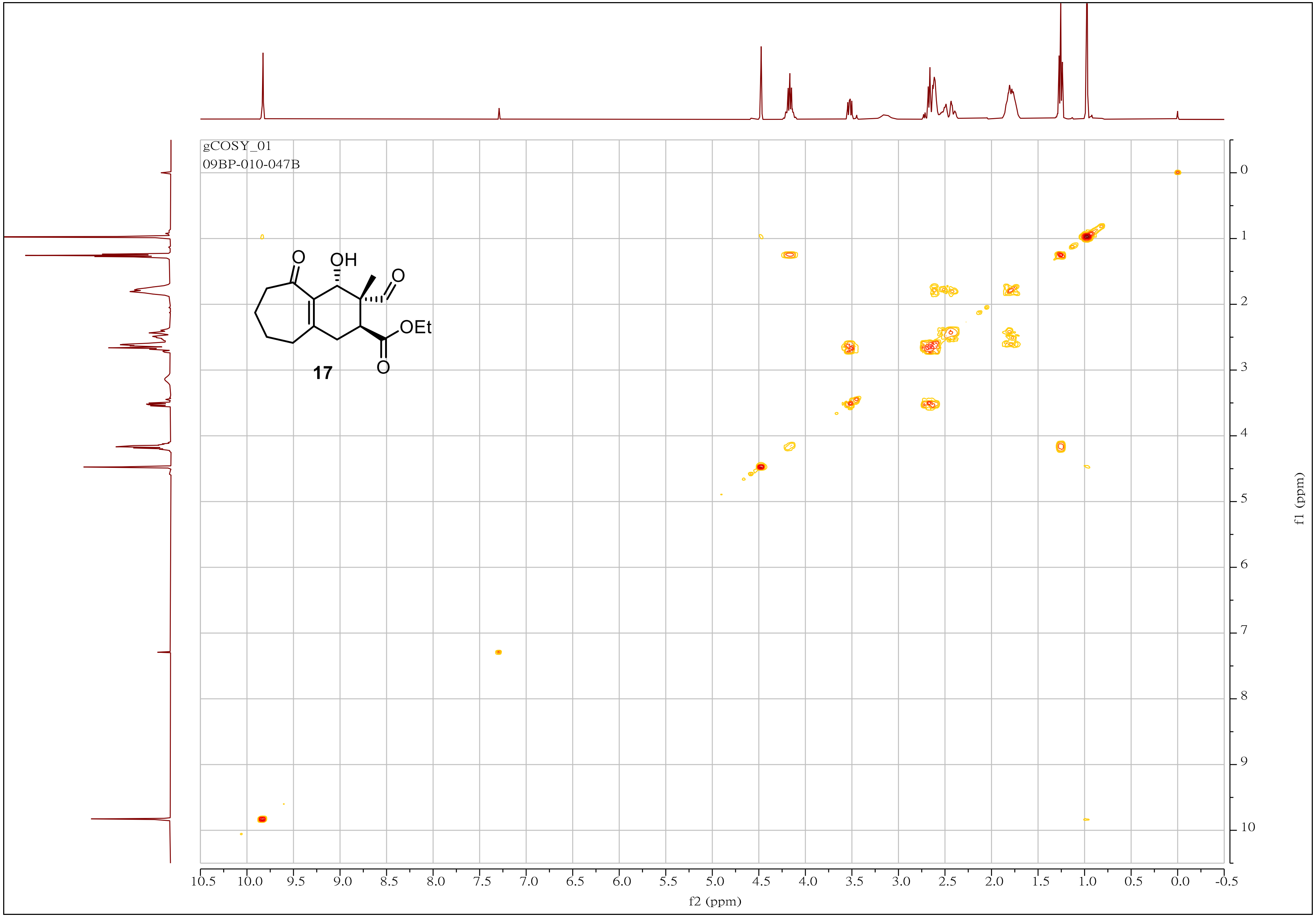


$^{1}H$-$^{1}H$ COSY spectrum for compound **17**

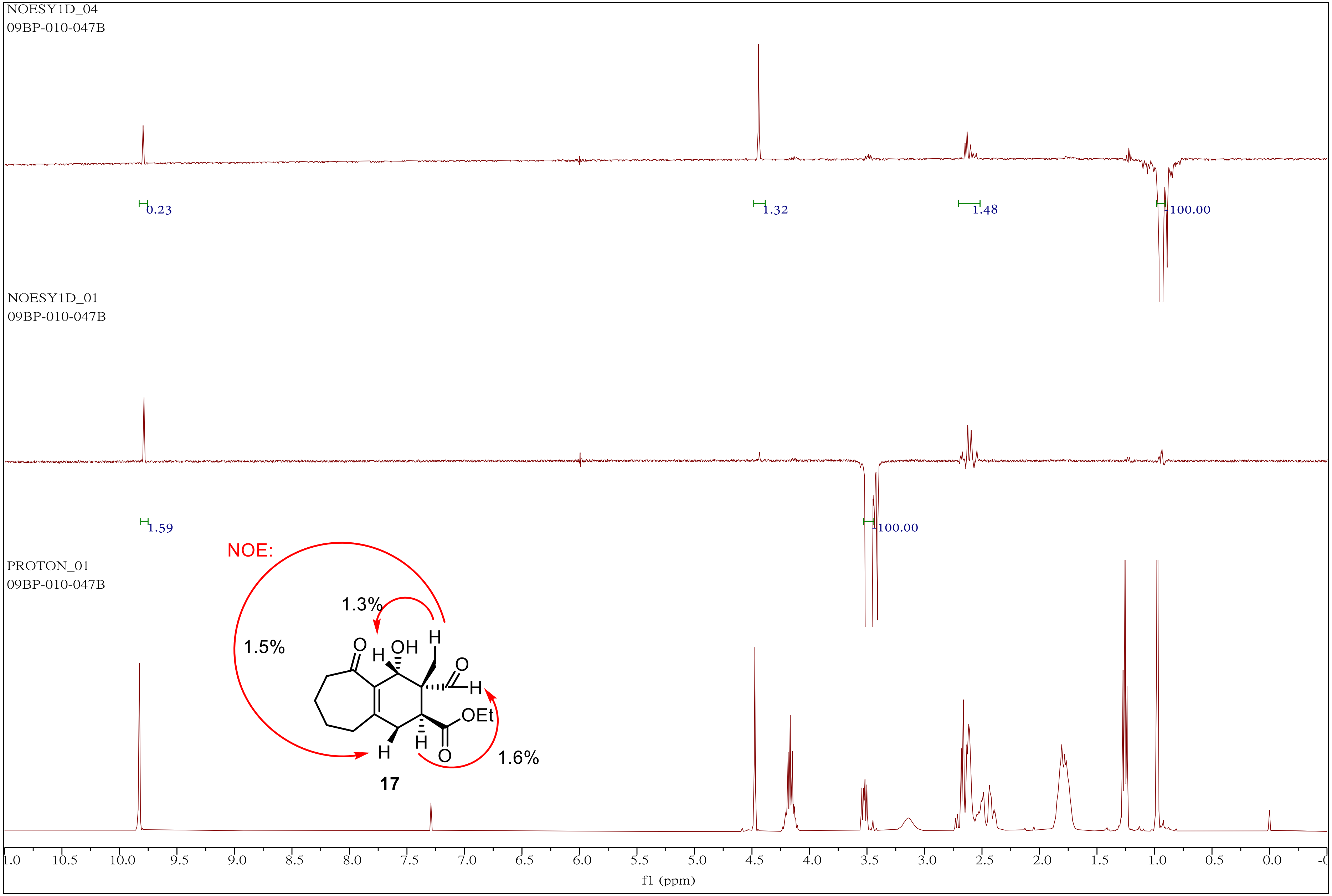


1D NOESY spectra for compound **17**

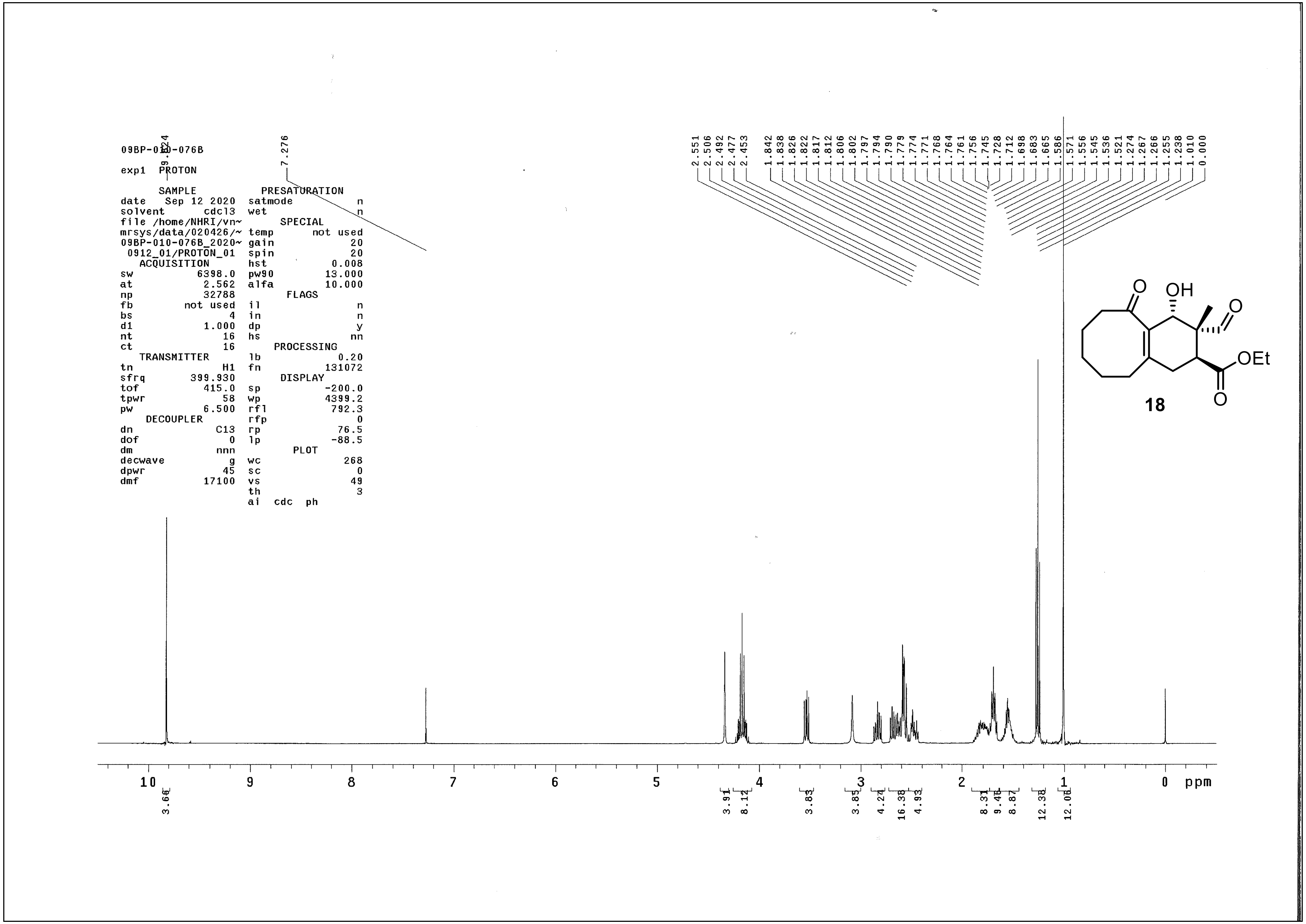


$^{1}$H NMR spectrum for compound **18**

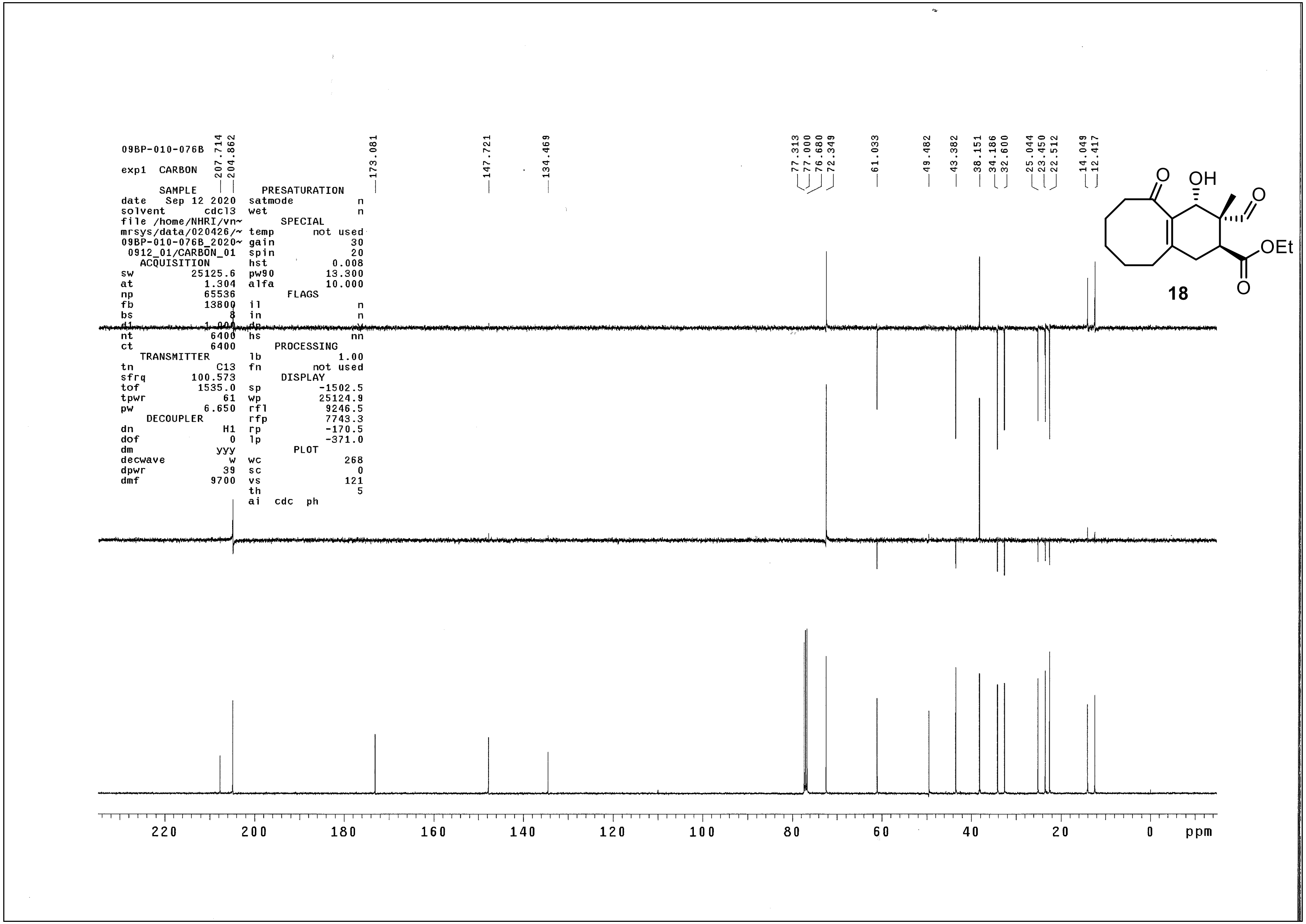


$^{13}$C NMR + DEPT spectra for compound **18**

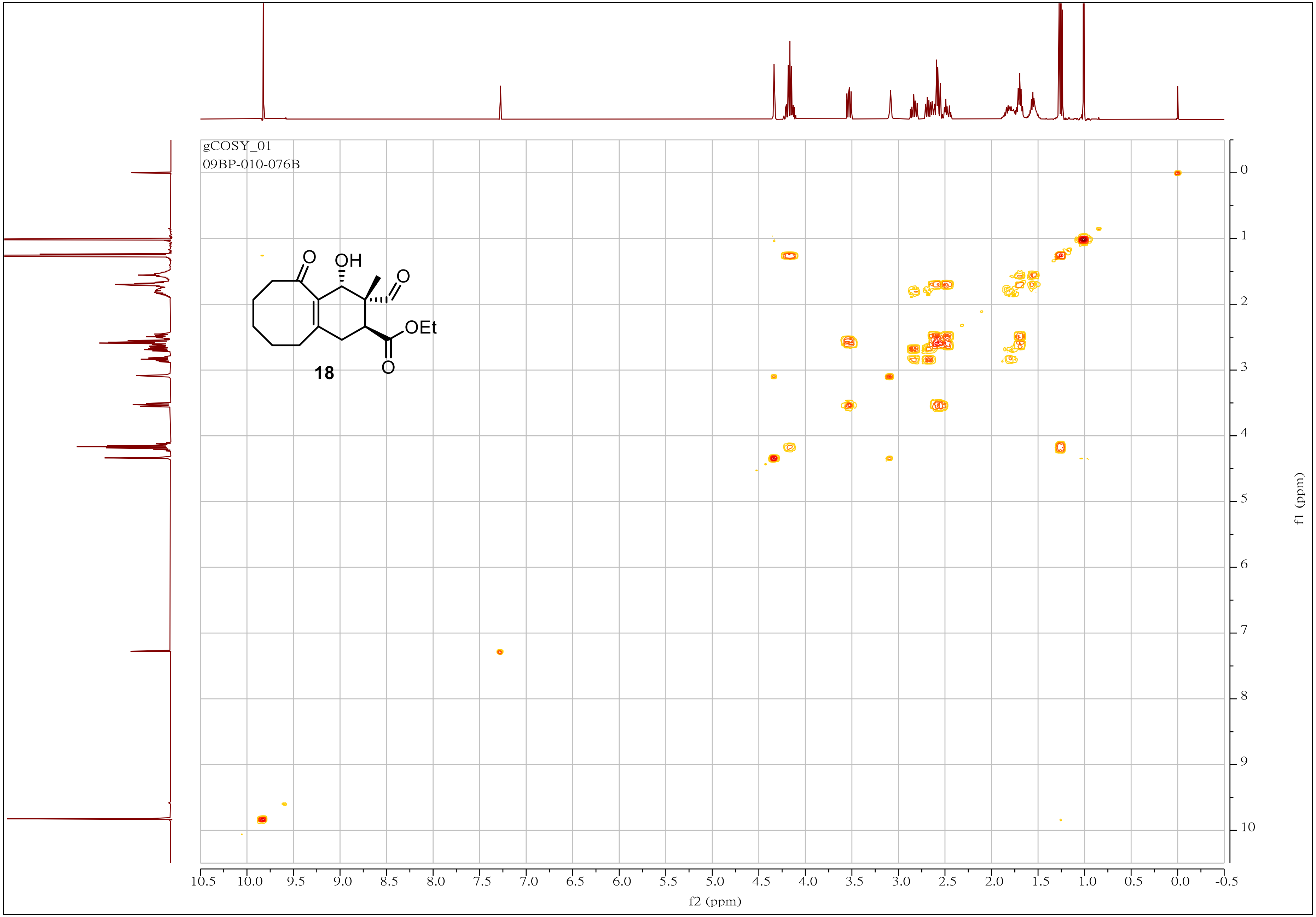


$^{1}$H-$^{1}$H COSY spectrum for compound **18**

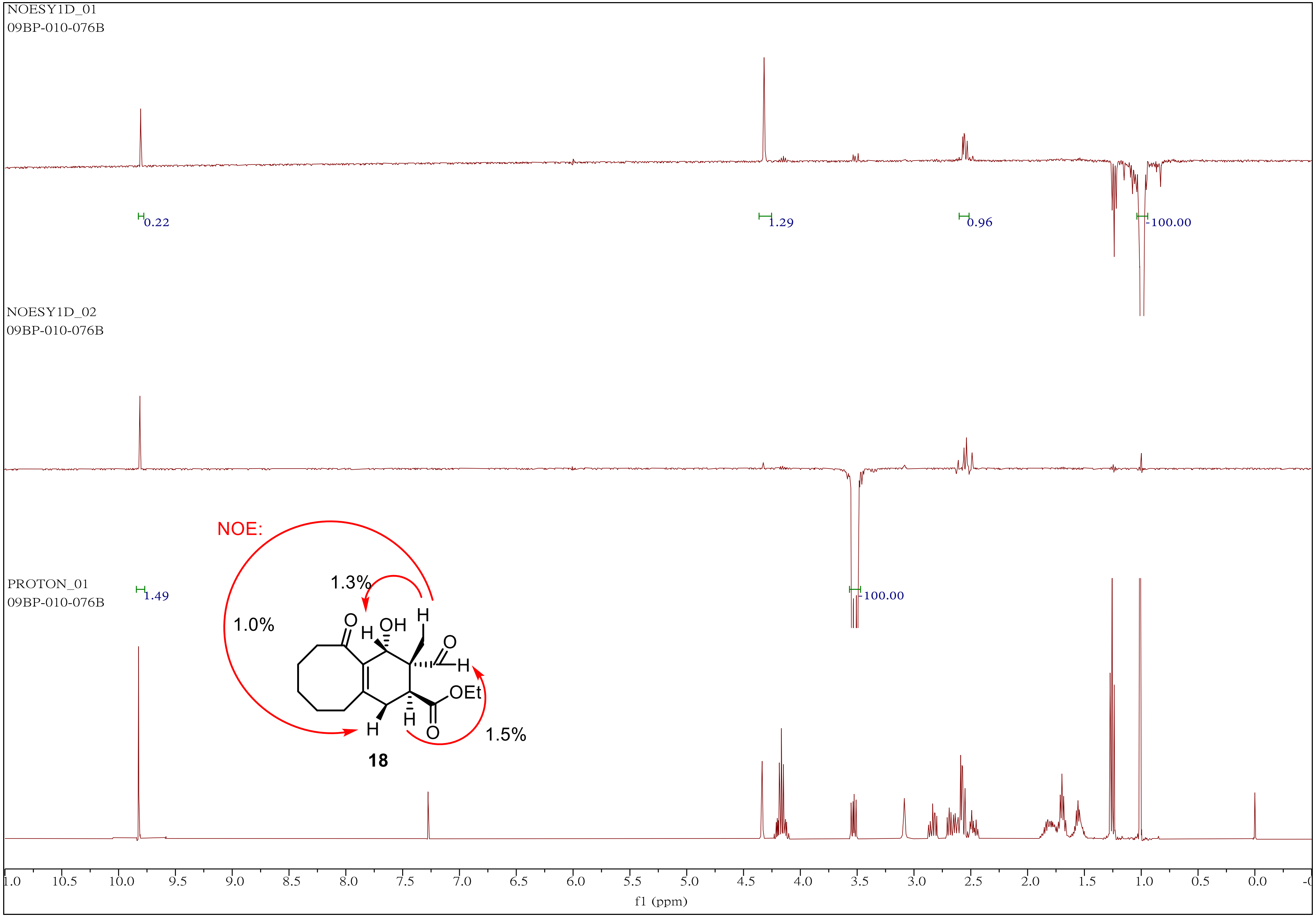


1D NOESY spectra for compound **18**

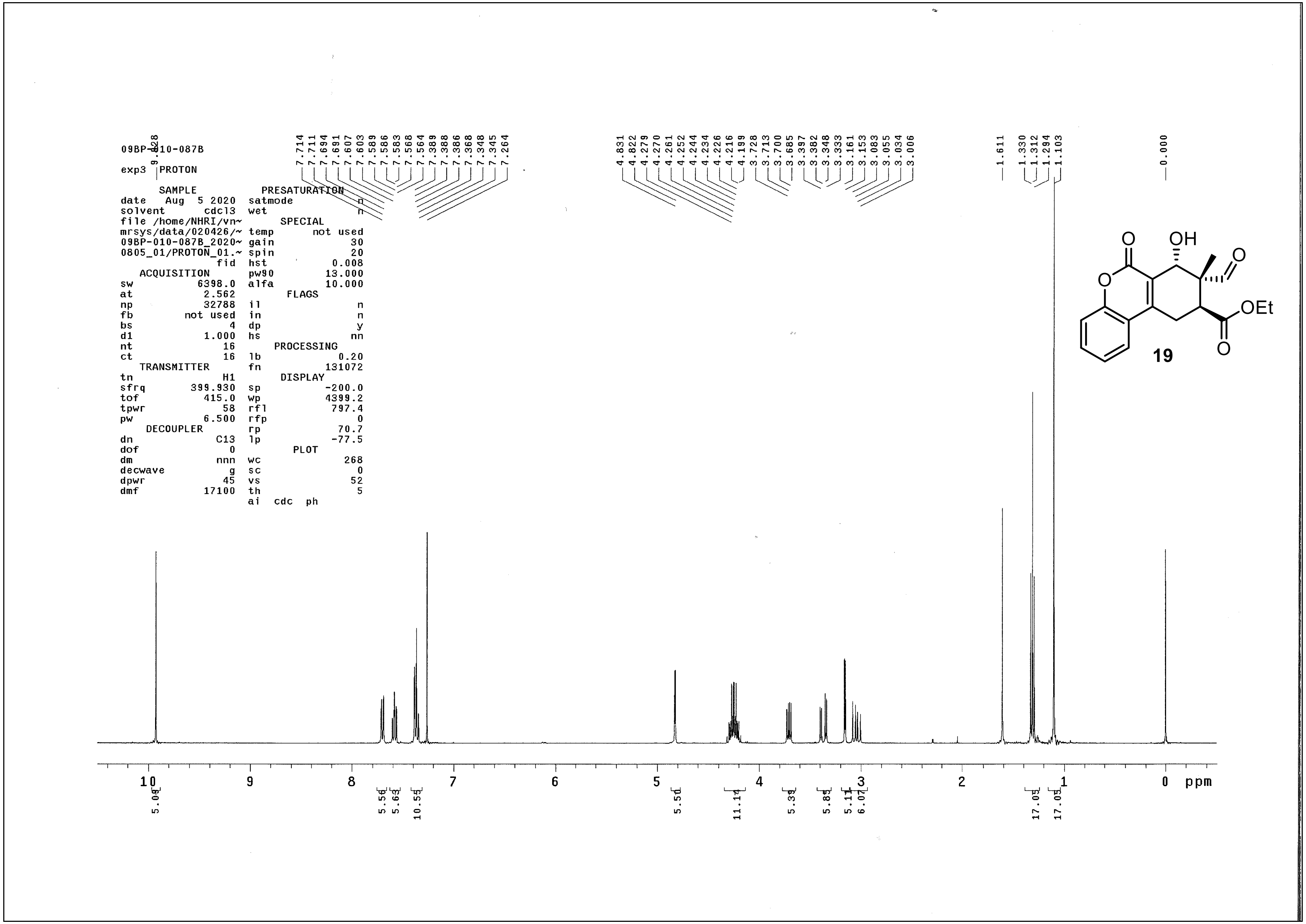

$^{1}$H NMR spectrum for compound **19**

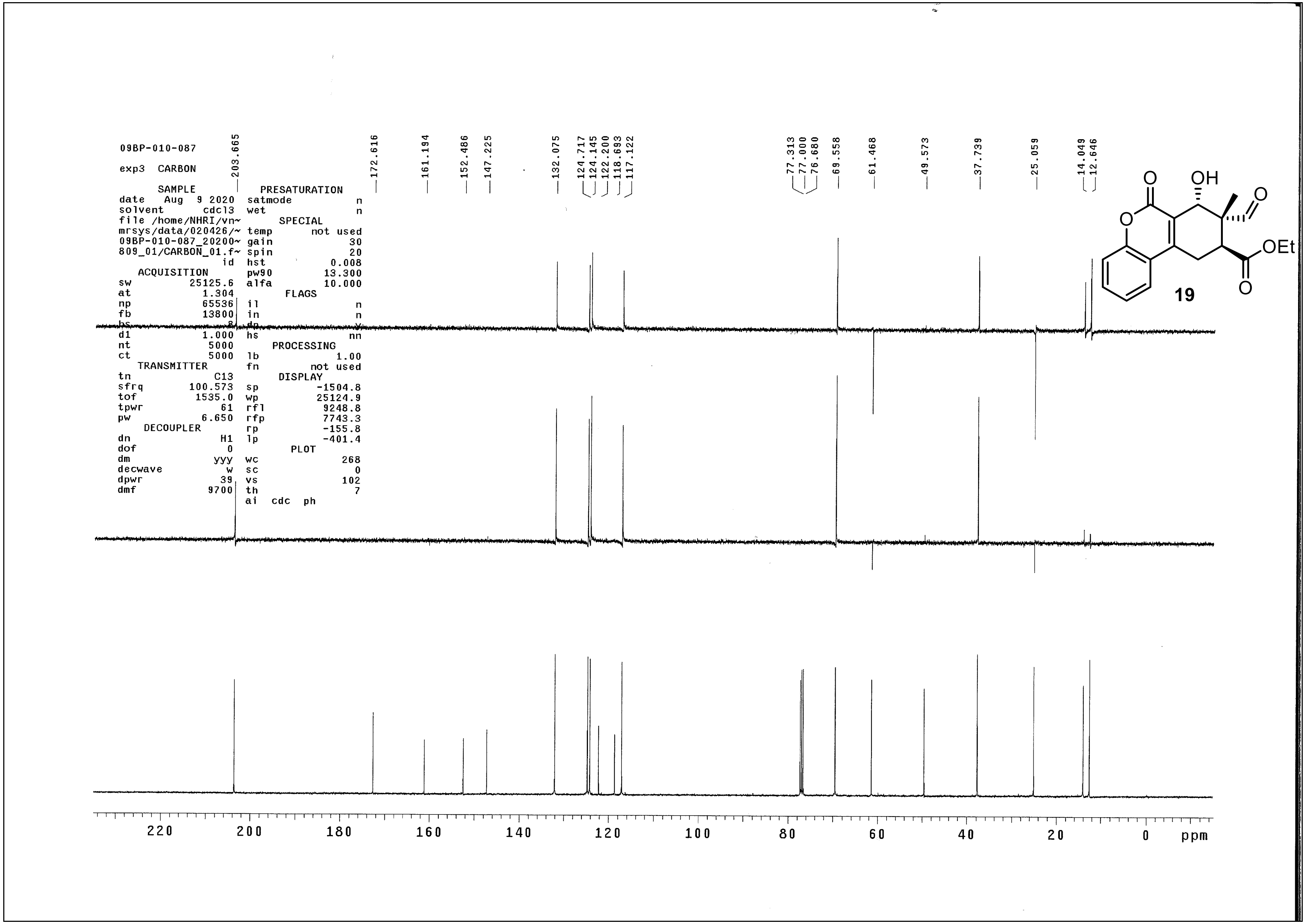


$^{13}$C NMR + DEPT spectra for compound **19**

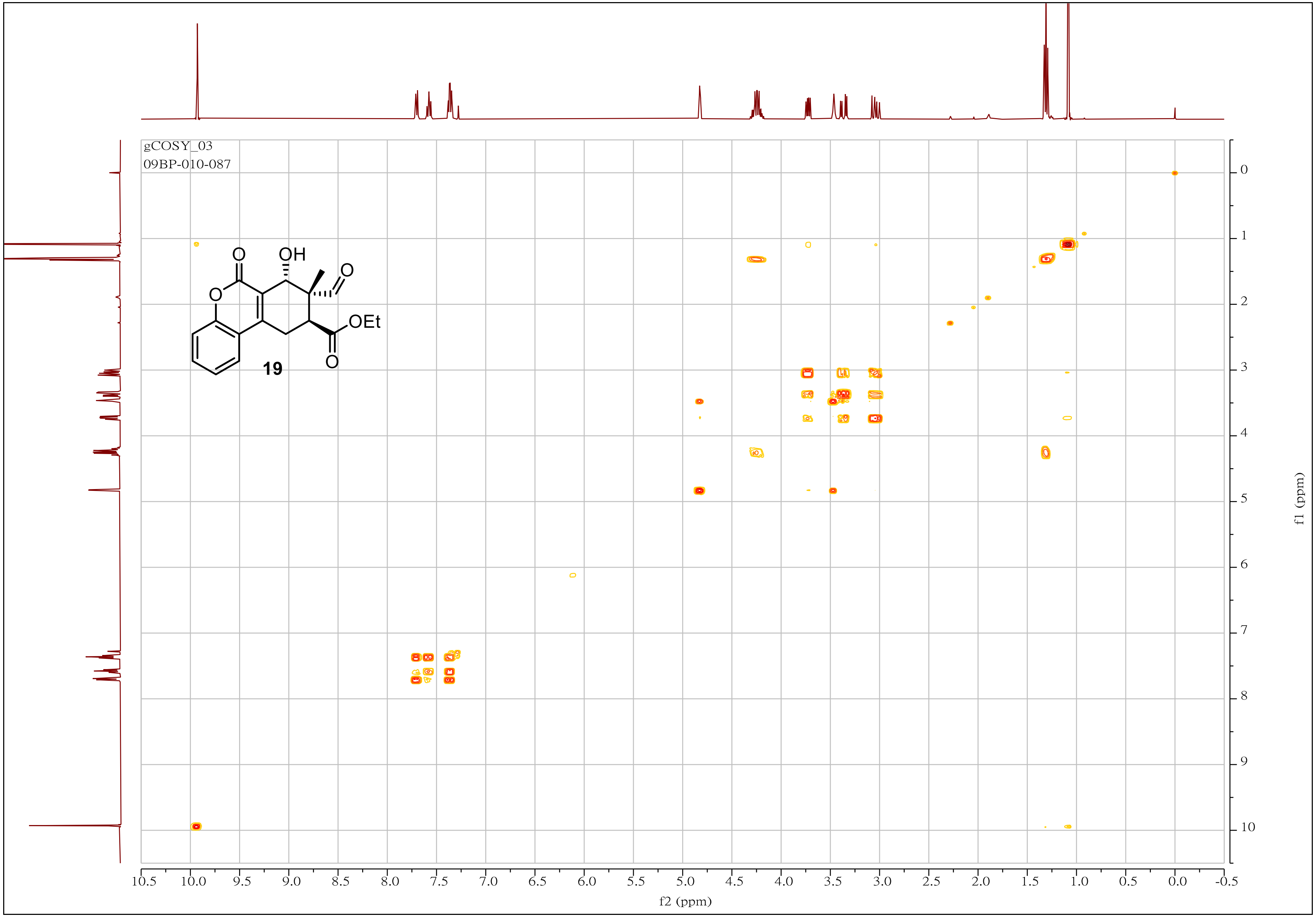


$^{1}$H-$^{1}$H COSY spectrum for compound **19**

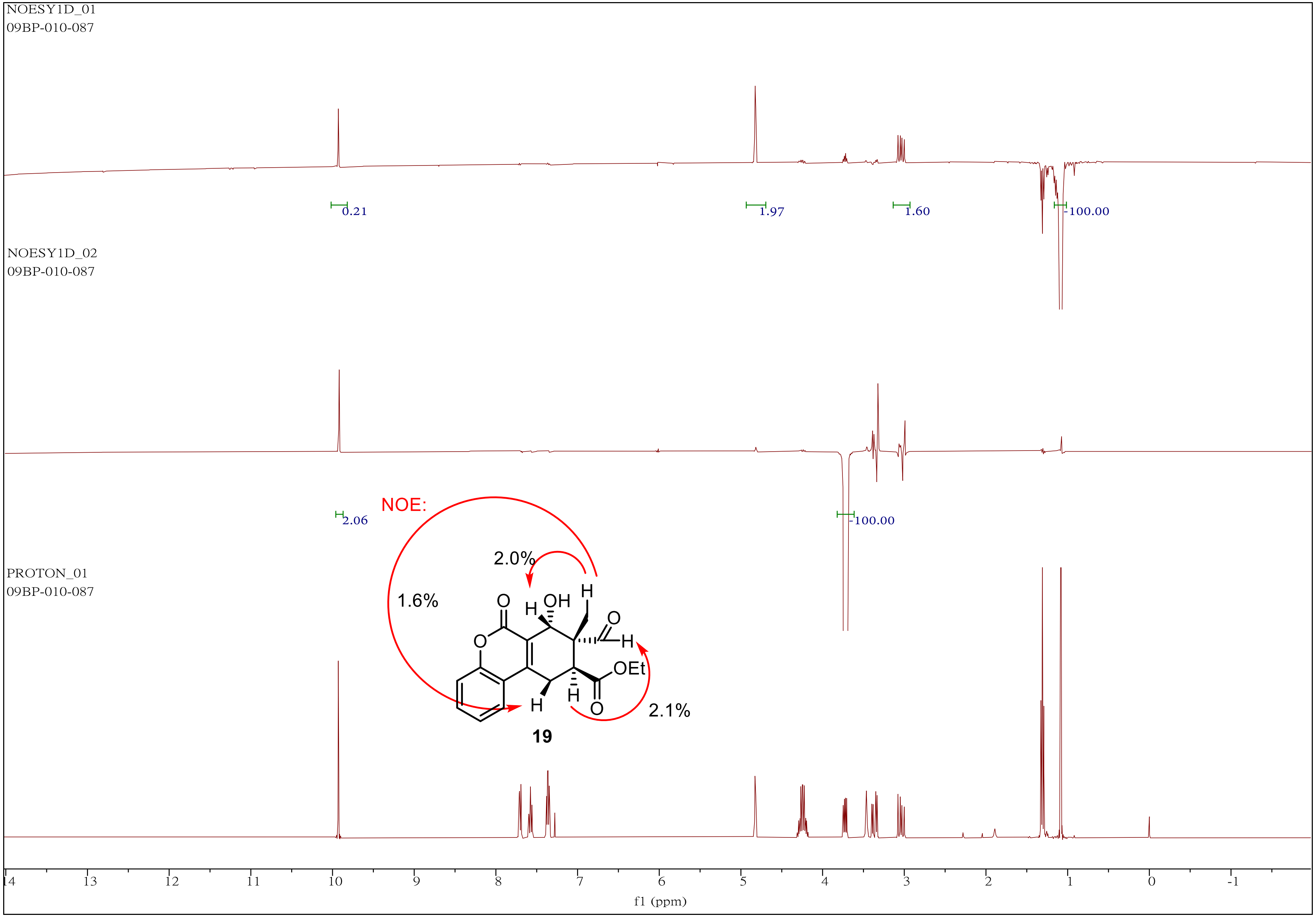


1D NOESY spectra for compound **19**

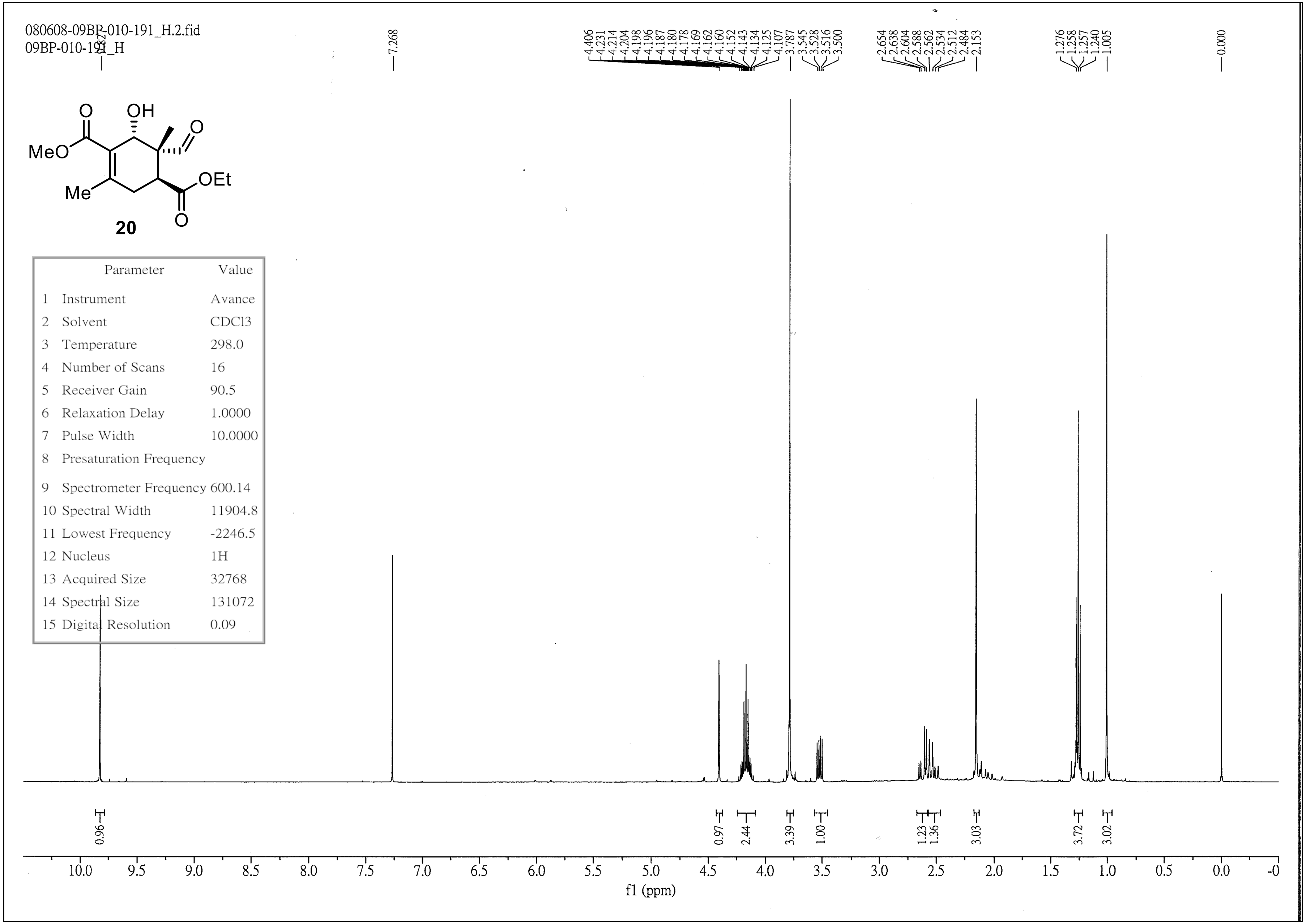


| | Parameter | Value |
|---|---|---|
| 1 | Instrument | Avance |
| 2 | Solvent | CDCl3 |
| 3 | Temperature | 298.0 |
| 4 | Number of Scans | 16 |
| 5 | Receiver Gain | 90.5 |
| 6 | Relaxation Delay | 1.0000 |
| 7 | Pulse Width | 10.0000 |
| 8 | Presaturation Frequency | |
| 9 | Spectrometer Frequency | 600.14 |
| 10 | Spectral Width | 11904.8 |
| 11 | Lowest Frequency | -2246.5 |
| 12 | Nucleus | 1H |
| 13 | Acquired Size | 32768 |
| 14 | Spectral Size | 131072 |
| 15 | Digital Resolution | 0.09 |

$^{1}$H NMR spectrum for compound **20**

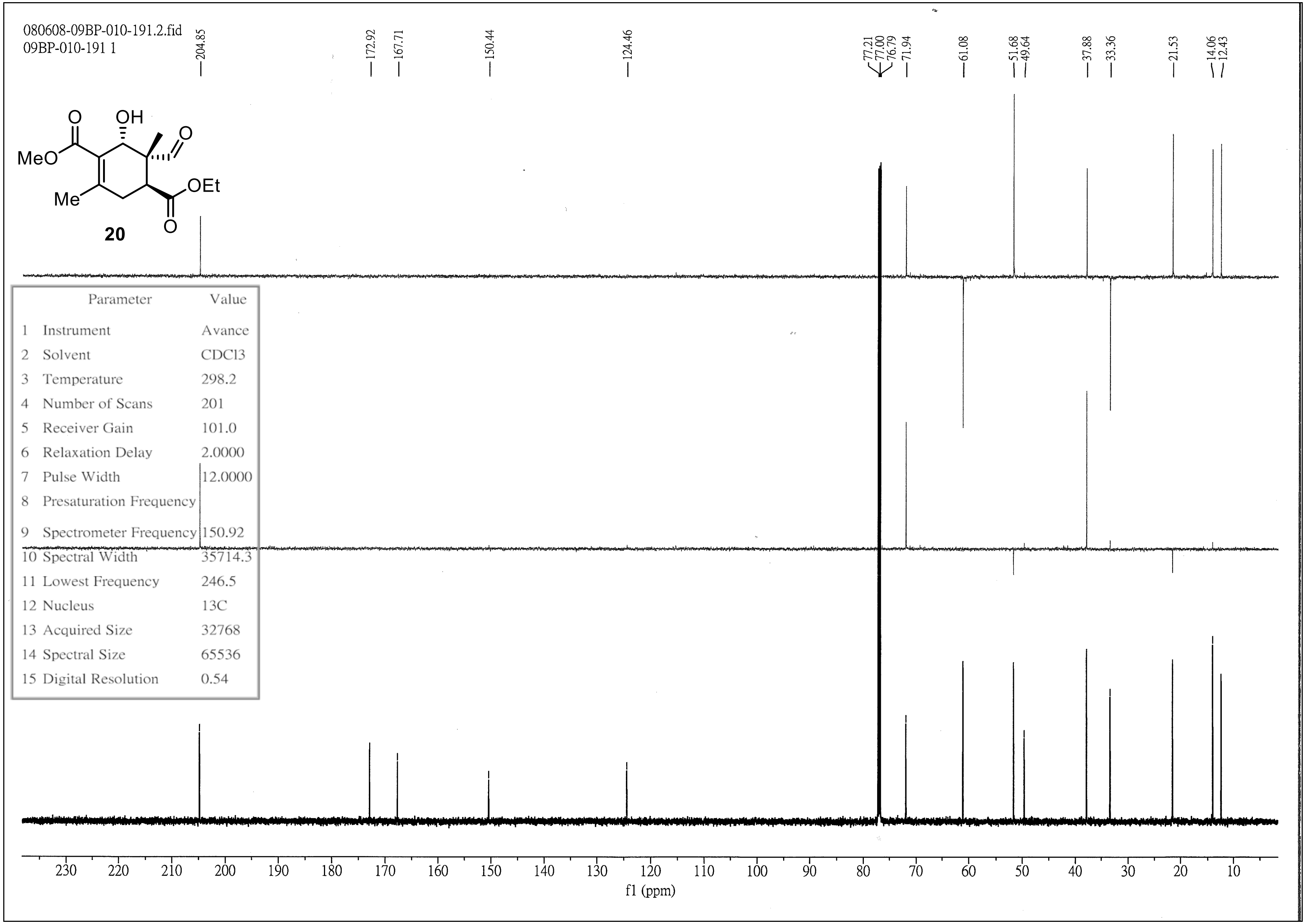


| Parameter | | Value |
|---|---|---|
| 1 | Instrument | Avance |
| 2 | Solvent | CDCl3 |
| 3 | Temperature | 298.2 |
| 4 | Number of Scans | 201 |
| 5 | Receiver Gain | 101.0 |
| 6 | Relaxation Delay | 2.0000 |
| 7 | Pulse Width | 12.0000 |
| 8 | Presaturation Frequency | |
| 9 | Spectrometer Frequency | 150.92 |
| 10 | Spectral Width | 35714.3 |
| 11 | Lowest Frequency | 246.5 |
| 12 | Nucleus | 13C |
| 13 | Acquired Size | 32768 |
| 14 | Spectral Size | 65536 |
| 15 | Digital Resolution | 0.54 |

$^{13}$C NMR + DEPT spectra for compound **20**

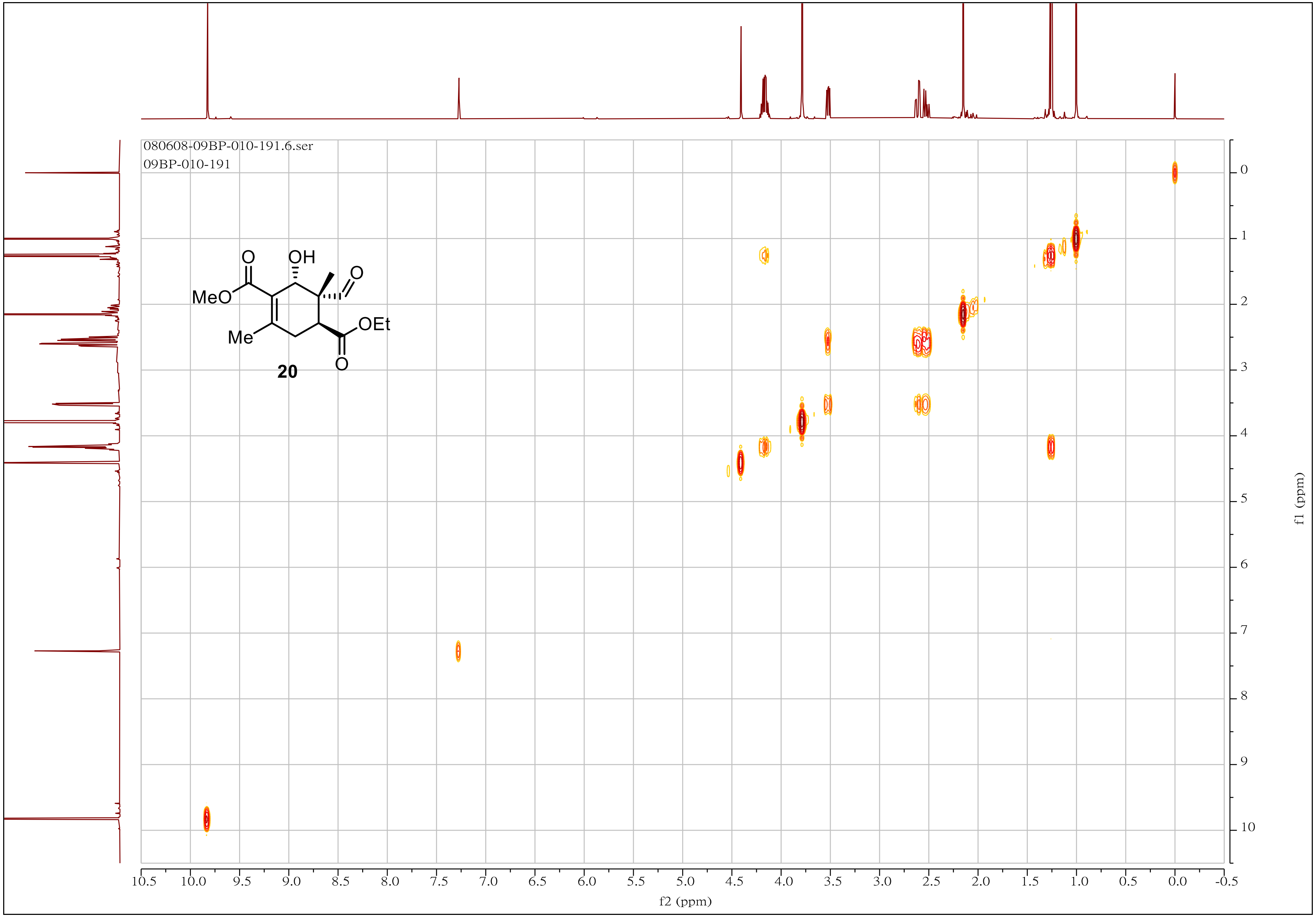


$^{1}$H-$^{1}$H COSY spectrum for compound **20**

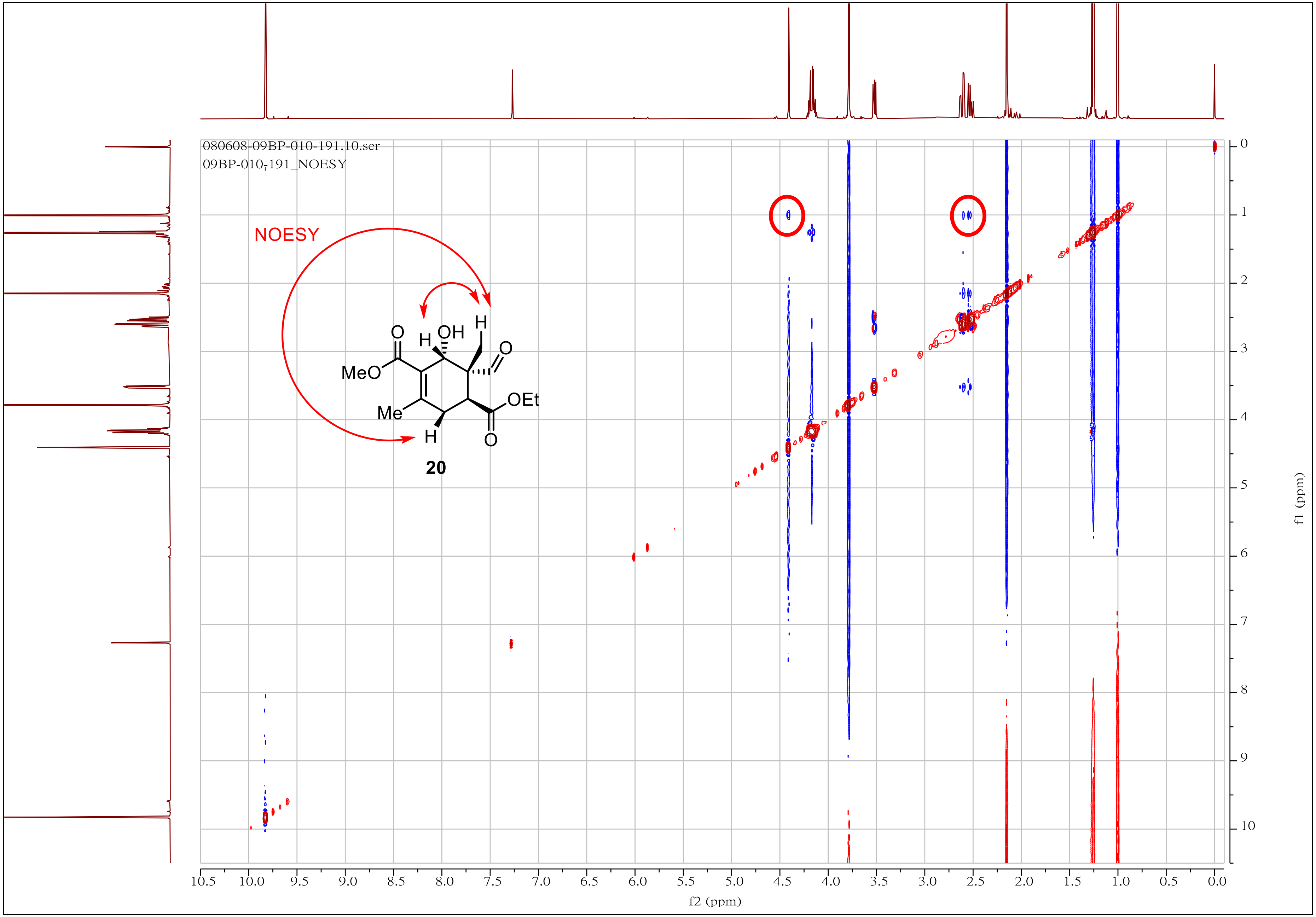


2D NOESY spectrum for compound **20**

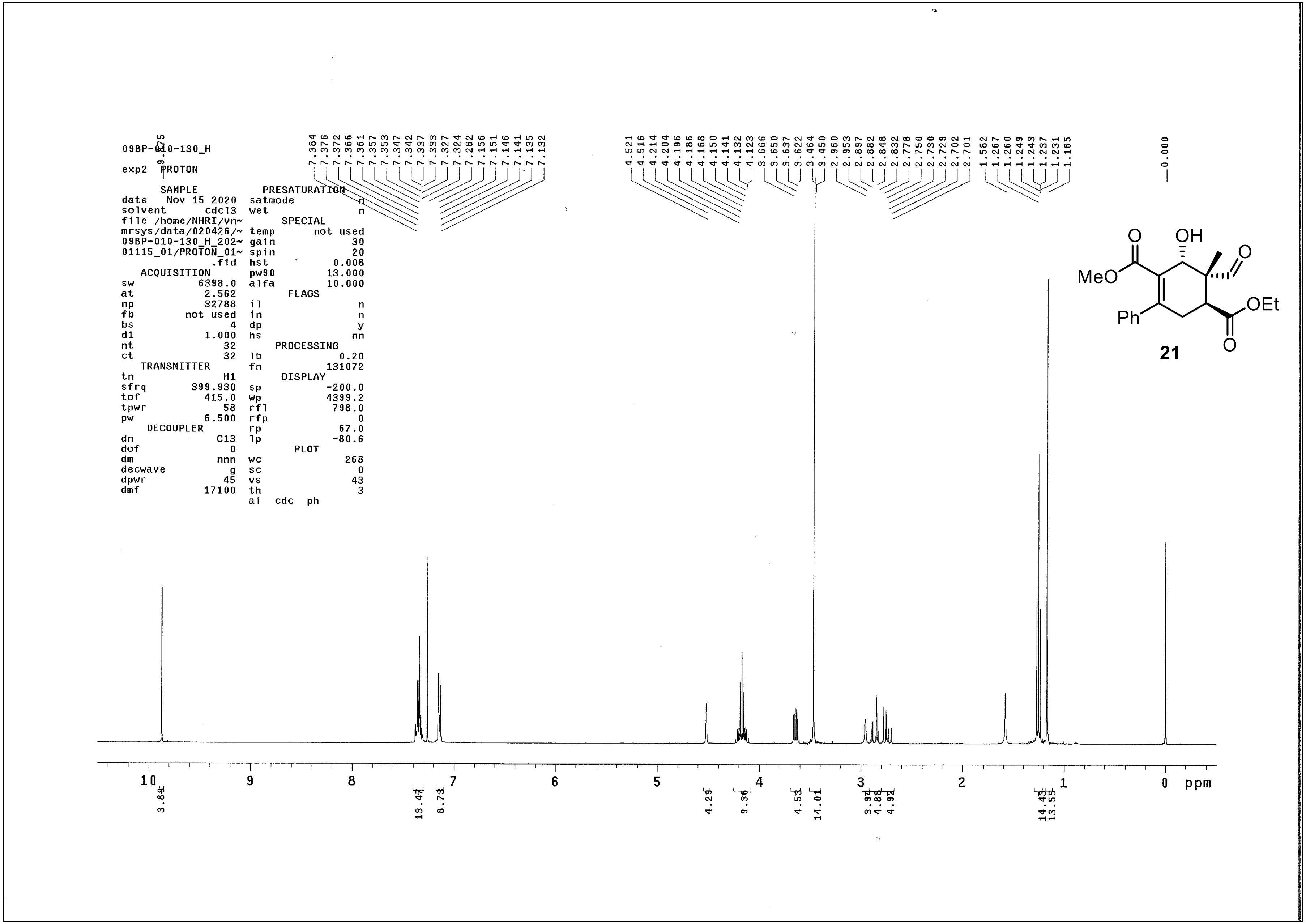

$^{1}$H NMR spectrum for compound **21**

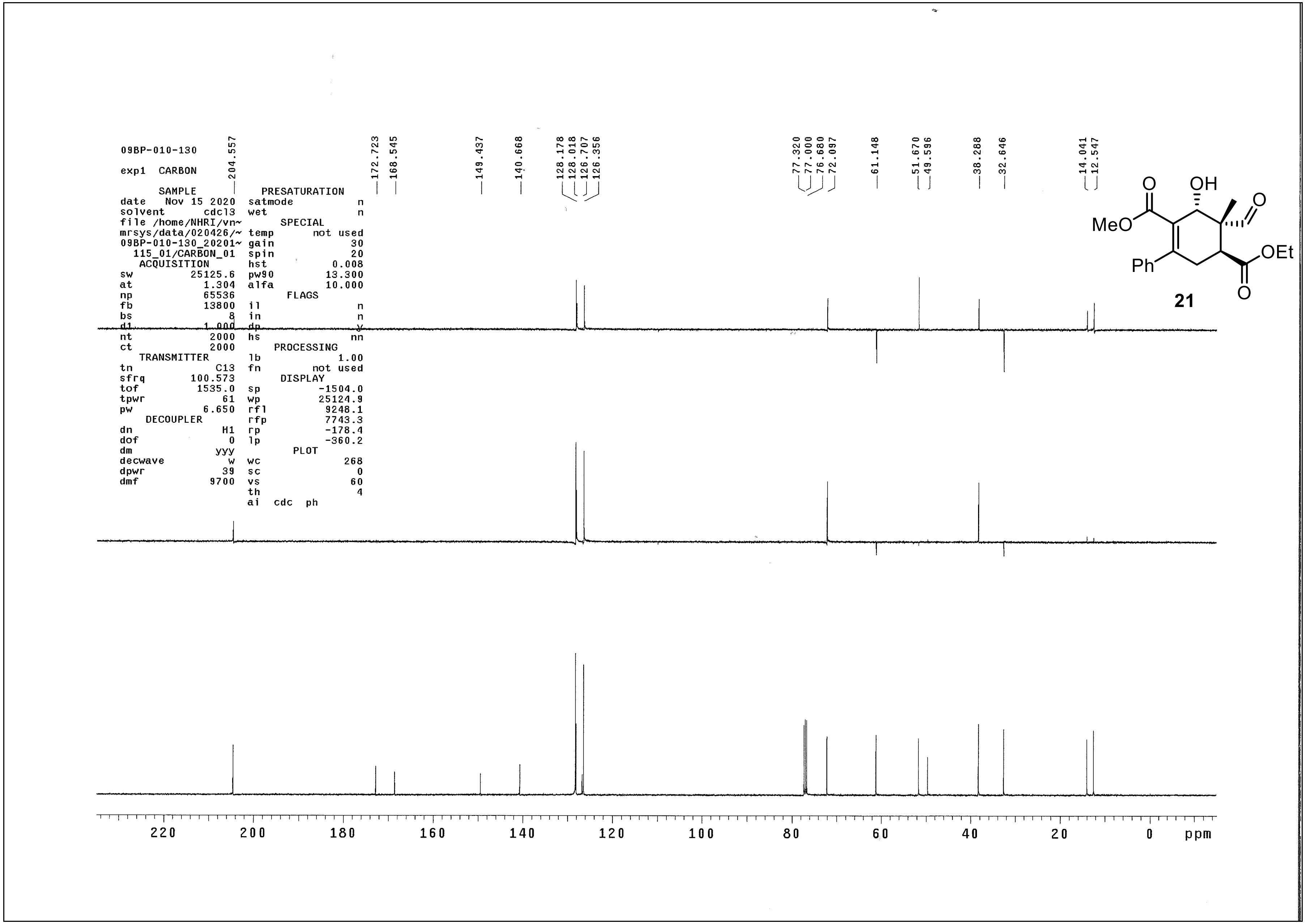


$^{13}$C NMR + DEPT spectra for compound **21**

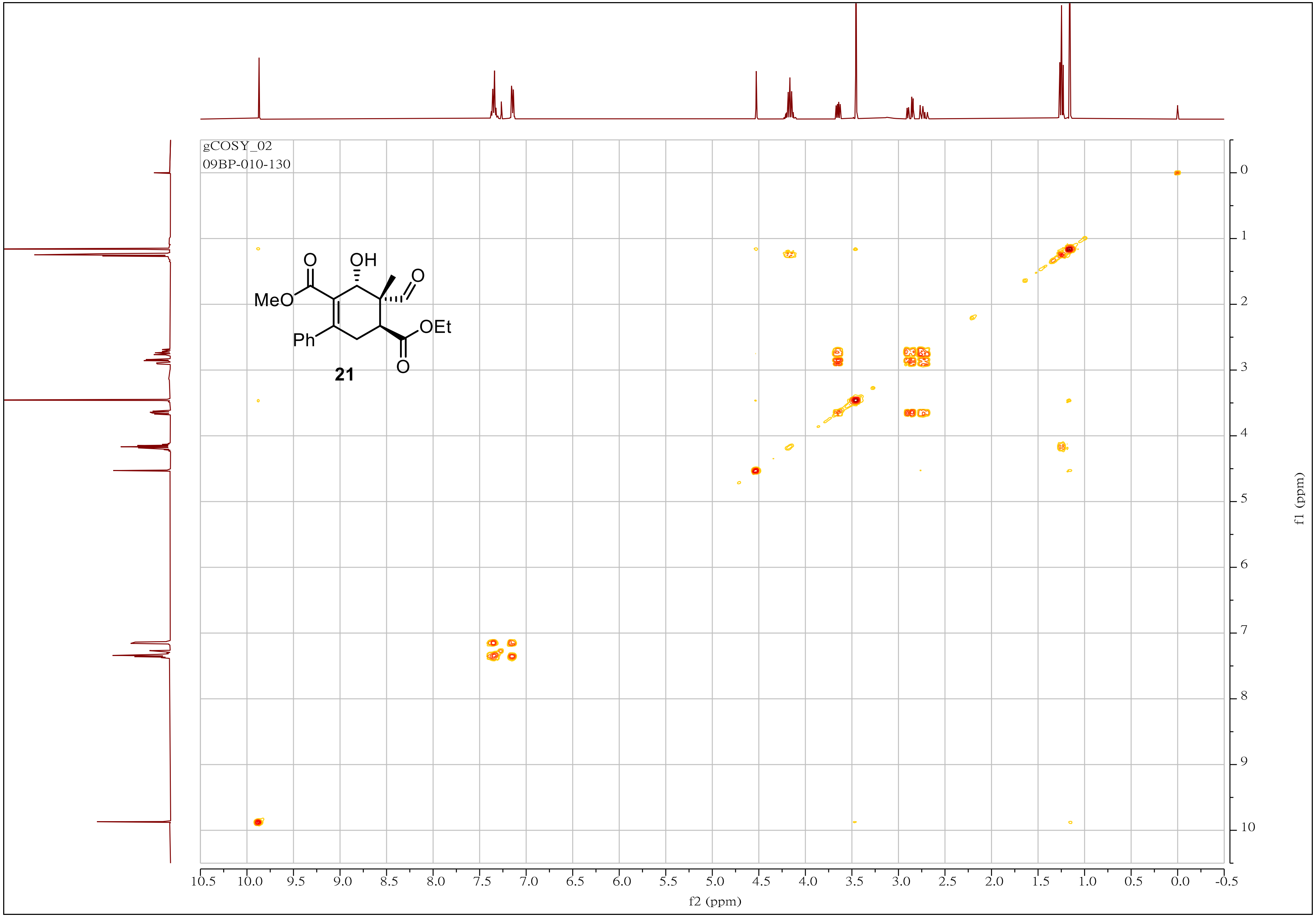


$^1$H-$^1$H COSY spectrum for compound **21**

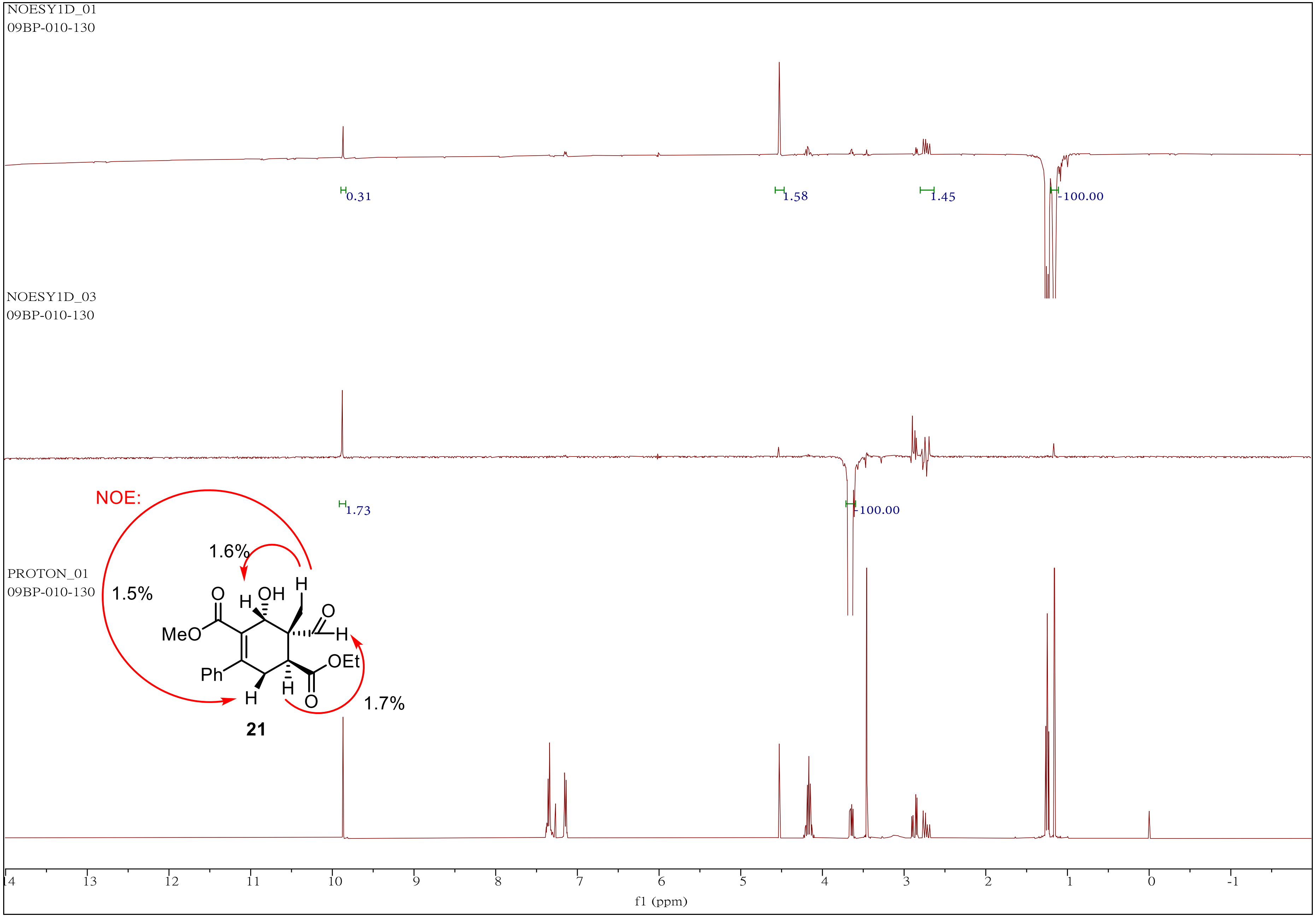


1D NOESY spectra for compound **21**

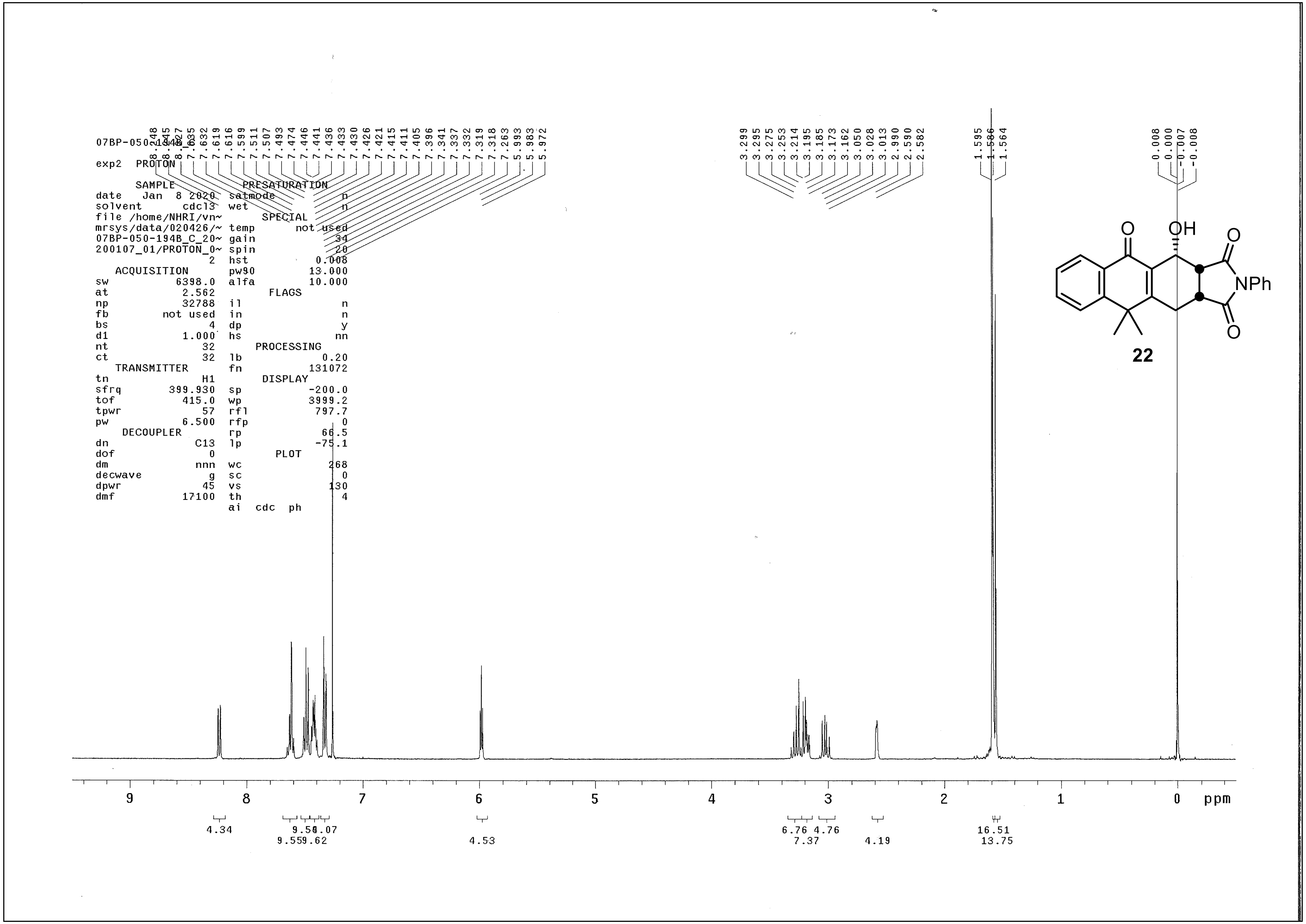

$^{1}$H NMR spectrum for compound **22**

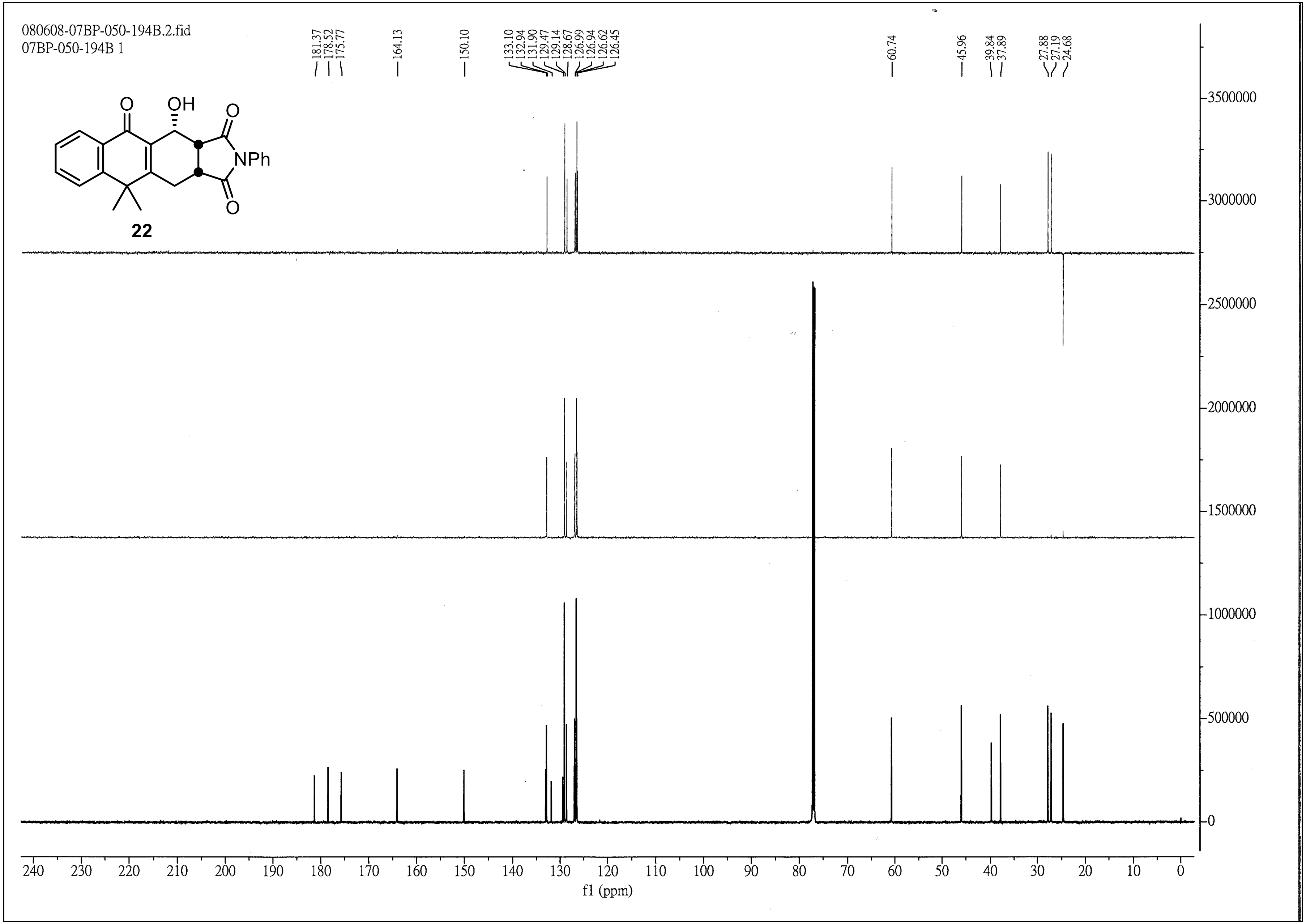


$^{13}$C NMR + DEPT spectra for compound **22**

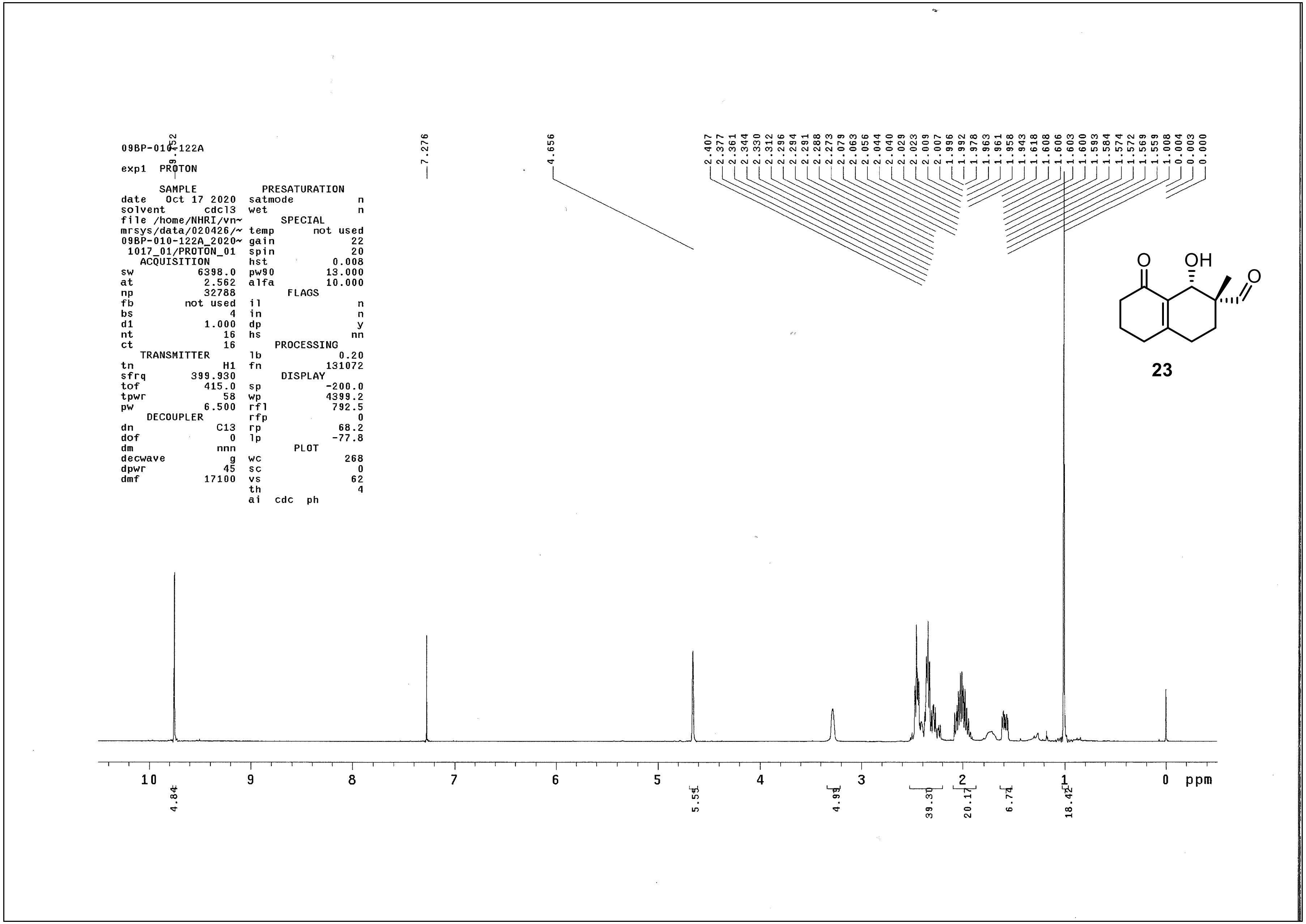


$^1$H NMR spectrum for compound **23**

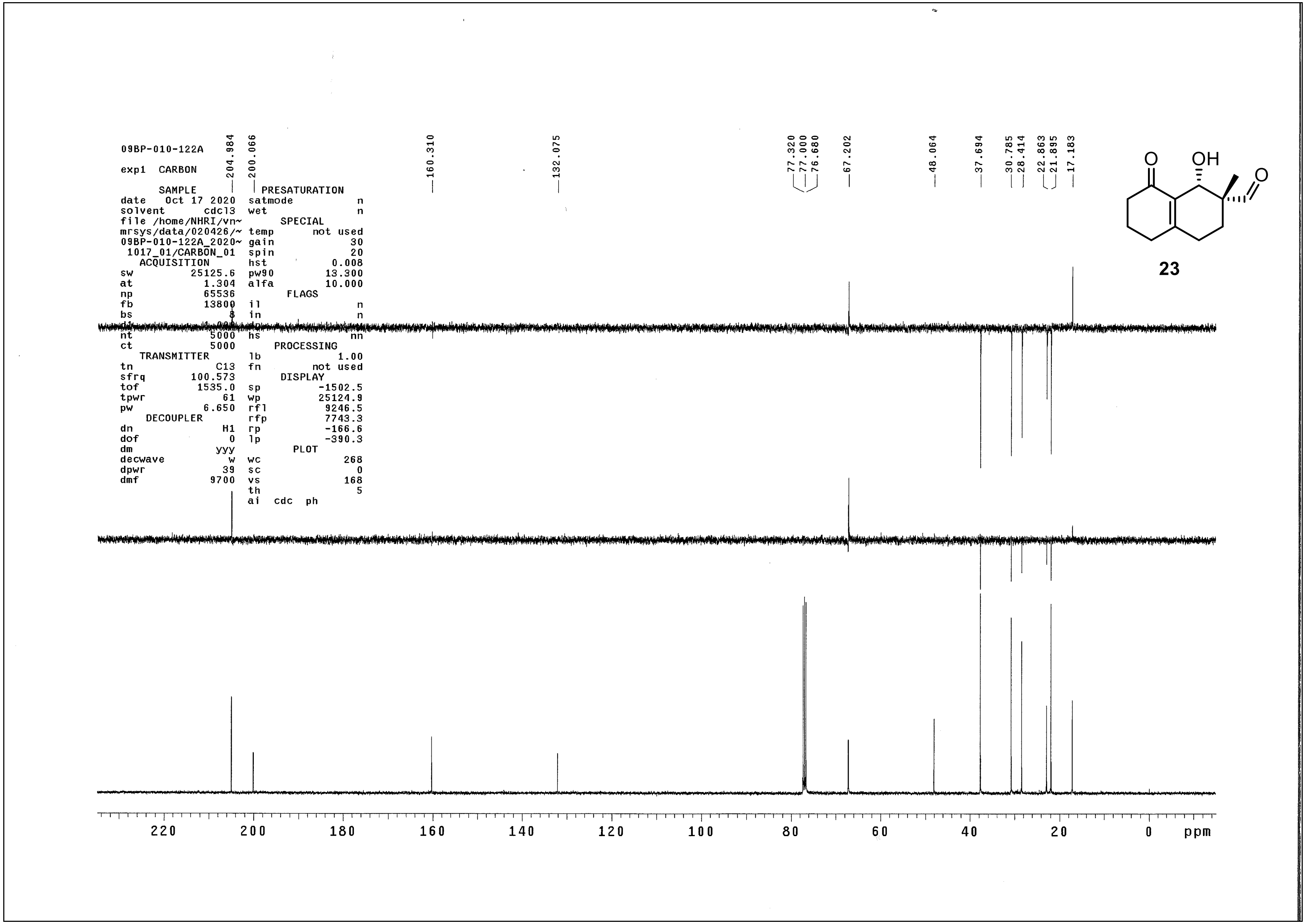


$^{13}$C NMR + DEPT spectra for compound **23**

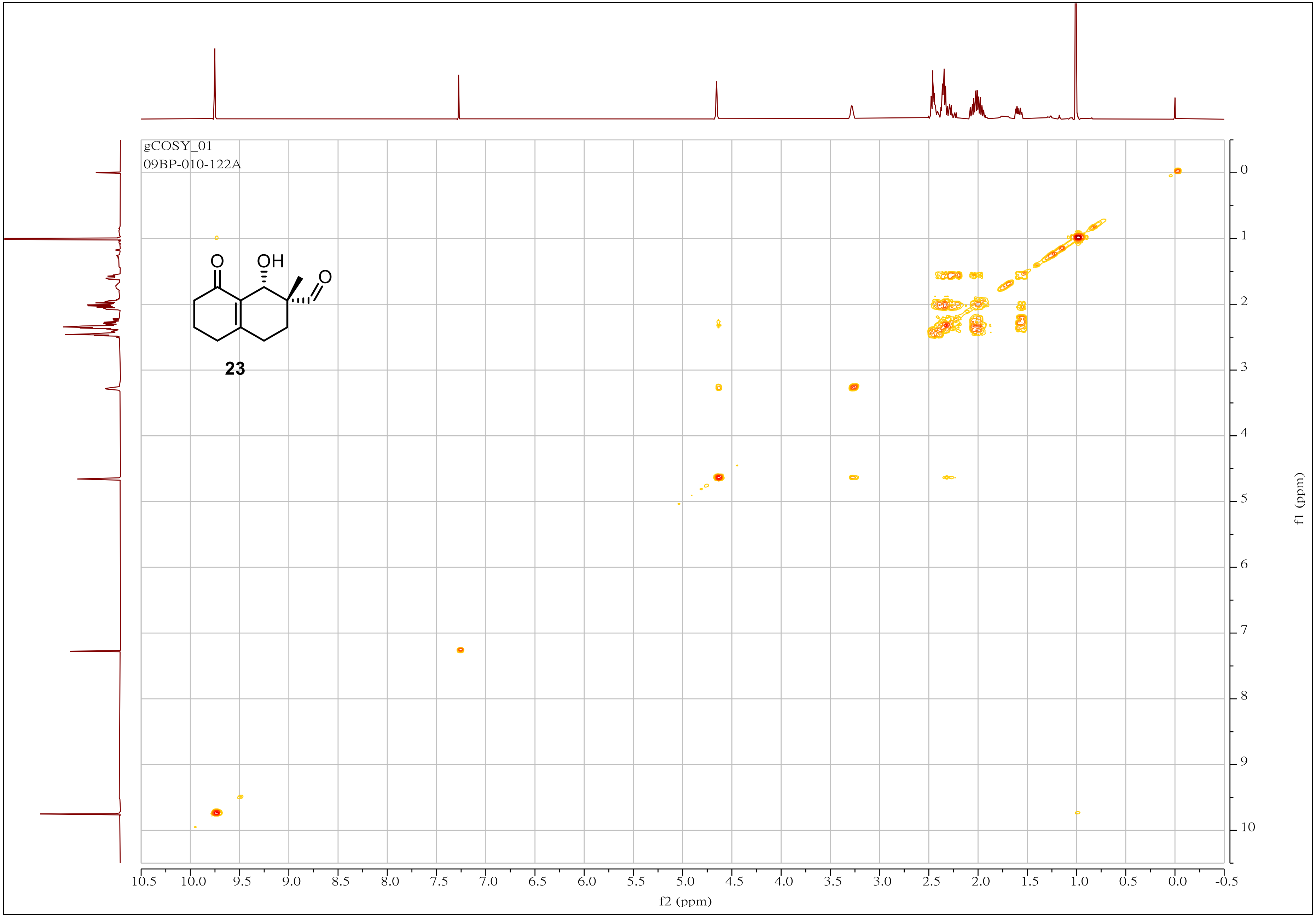


$^{1}$H-$^{1}$H COSY spectrum for compound **23**

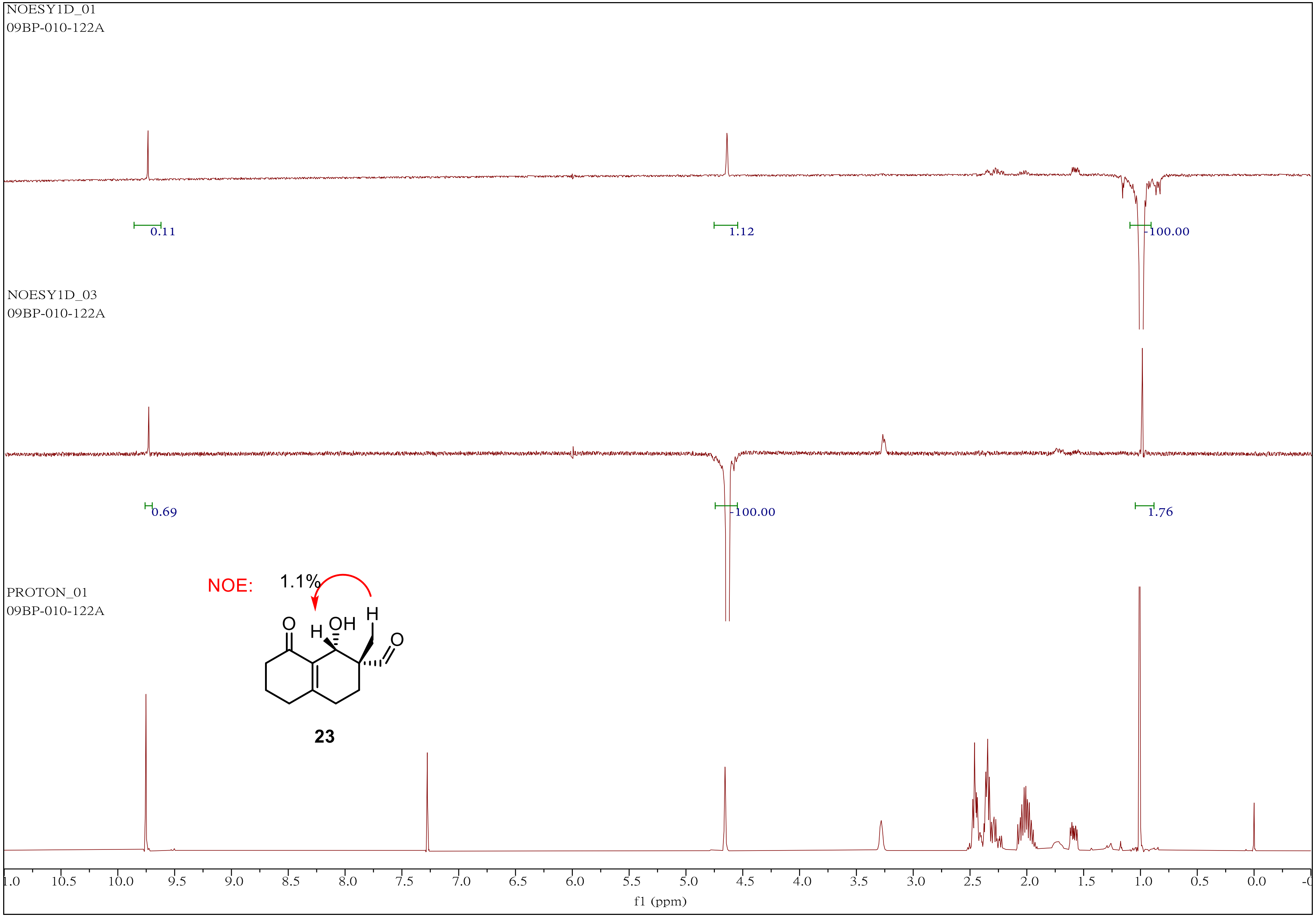


1D NOESY spectra for compound **23**

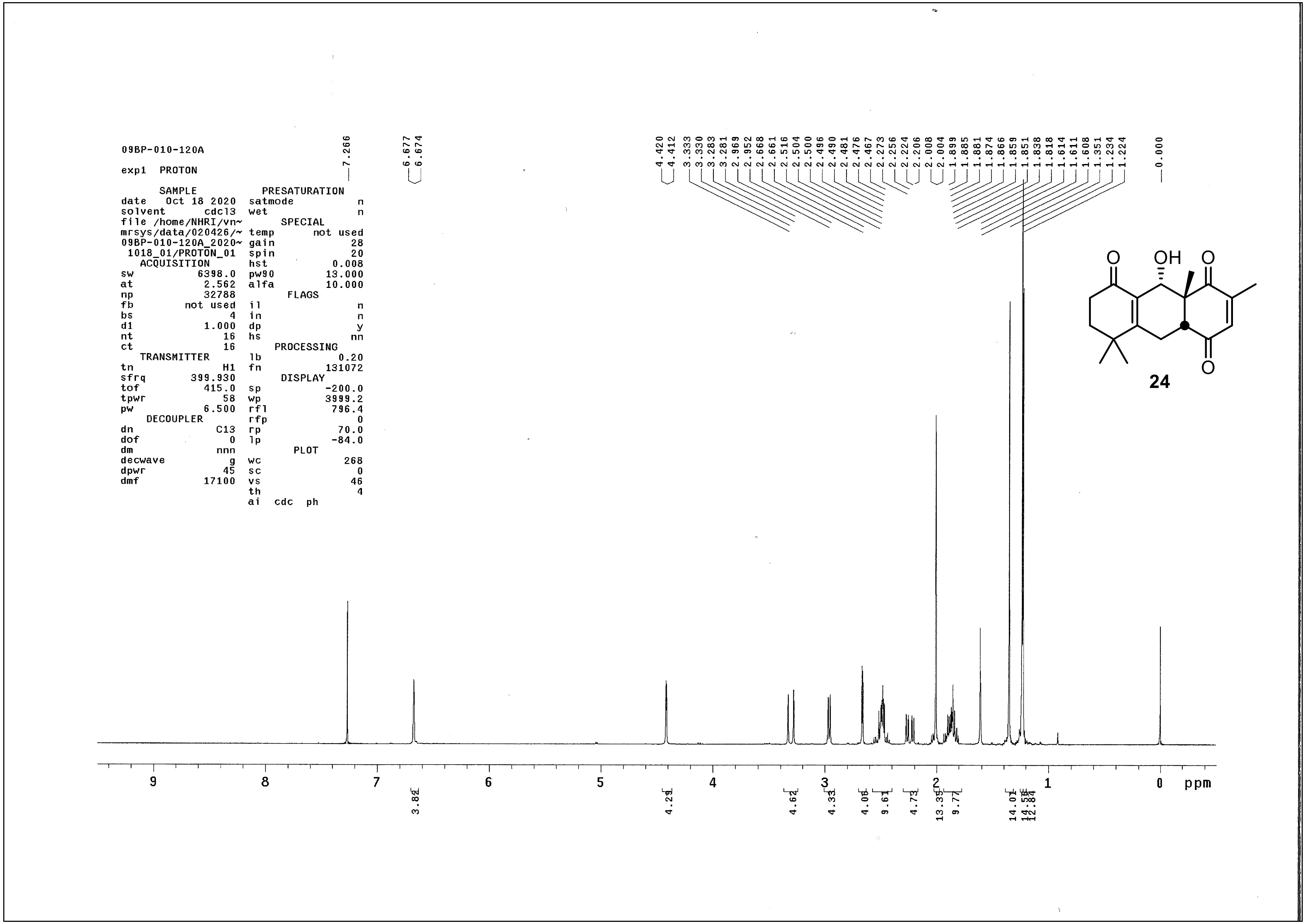

$^{1}$H NMR spectrum for compound **24**

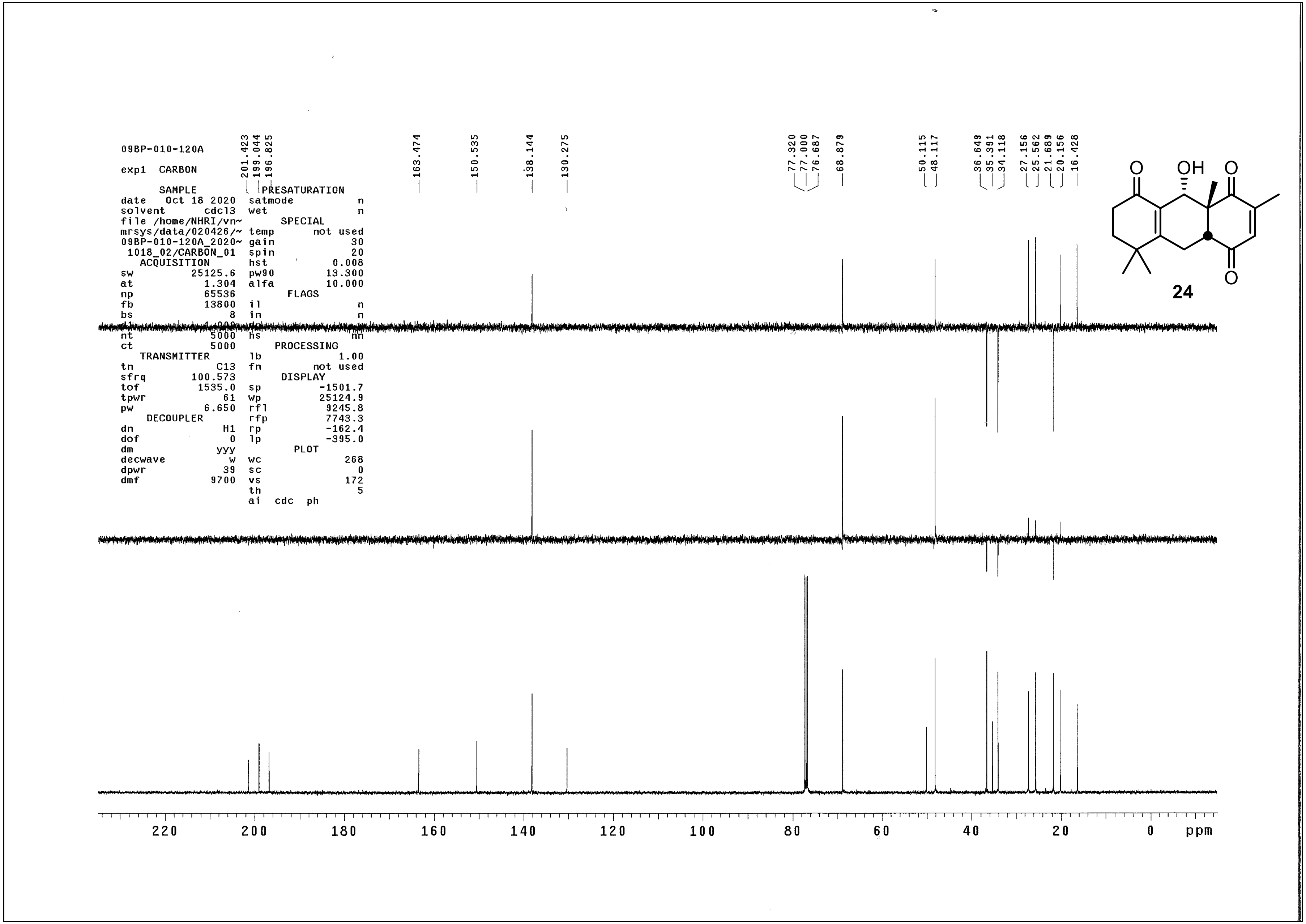


$^{13}$C NMR + DEPT spectra for compound **24**

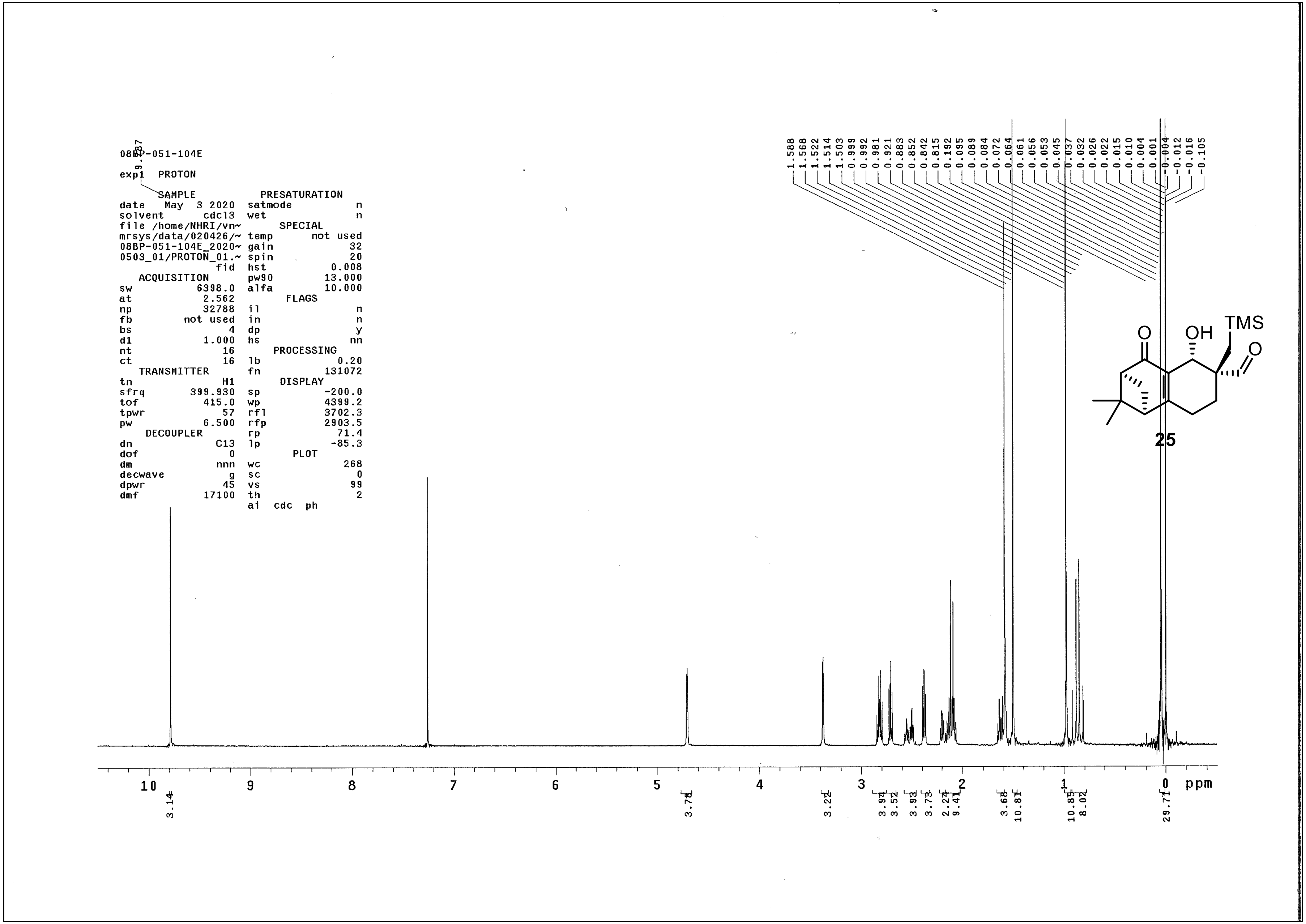

$^{1}$H NMR spectrum for compound **25**

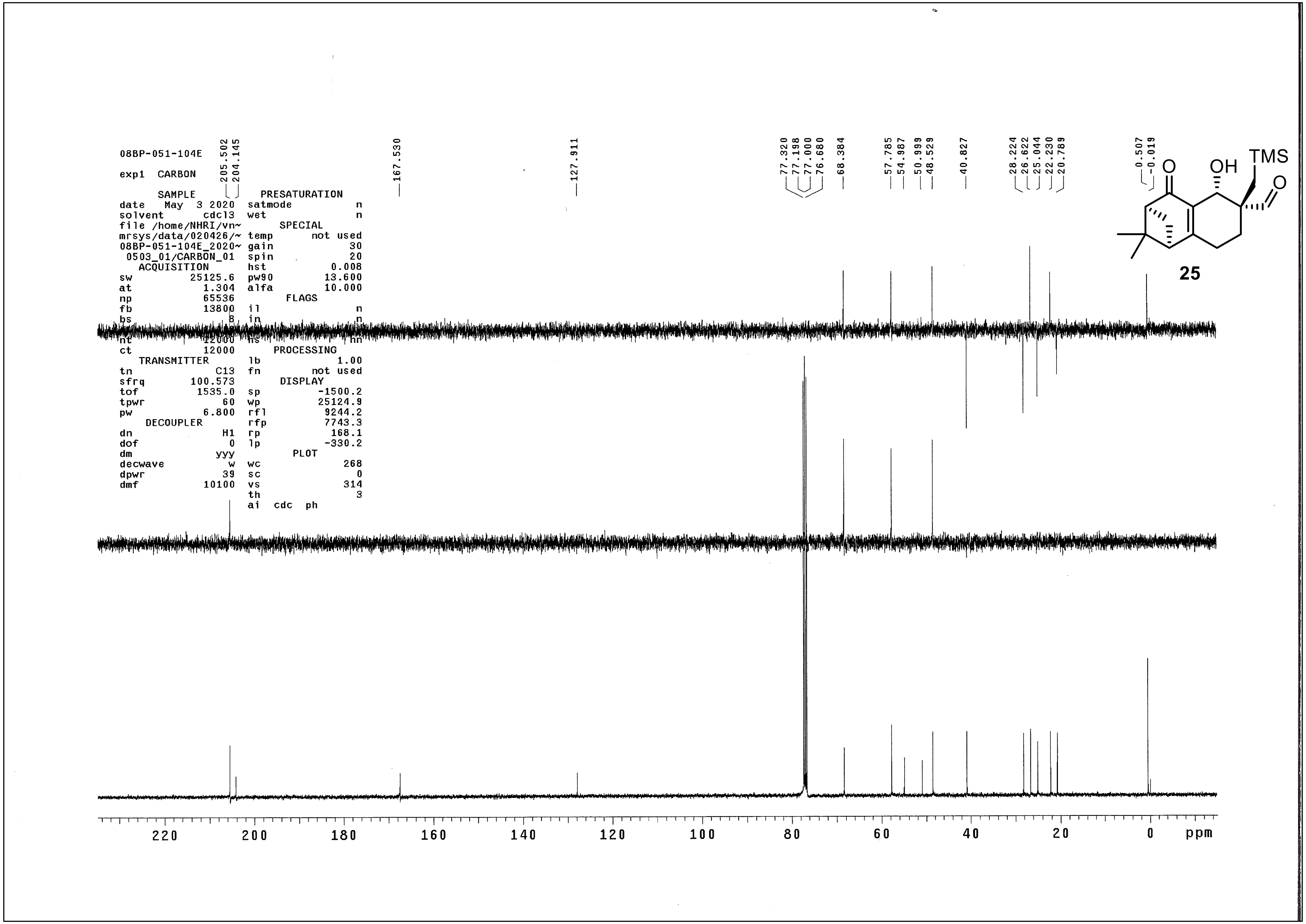


$^{13}$C NMR + DEPT spectra for compound **25**

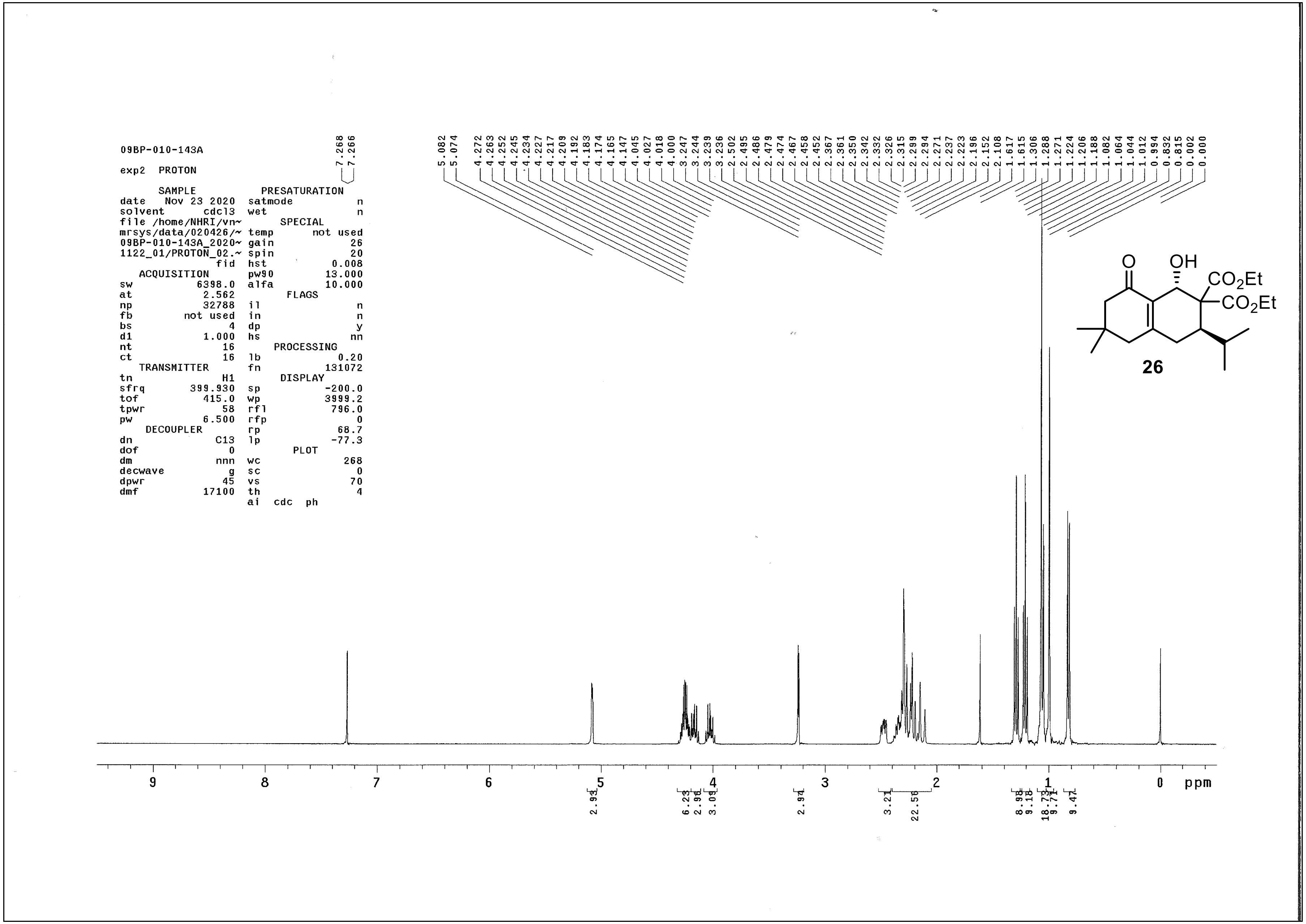

$^{1}$H NMR spectrum for compound **26**

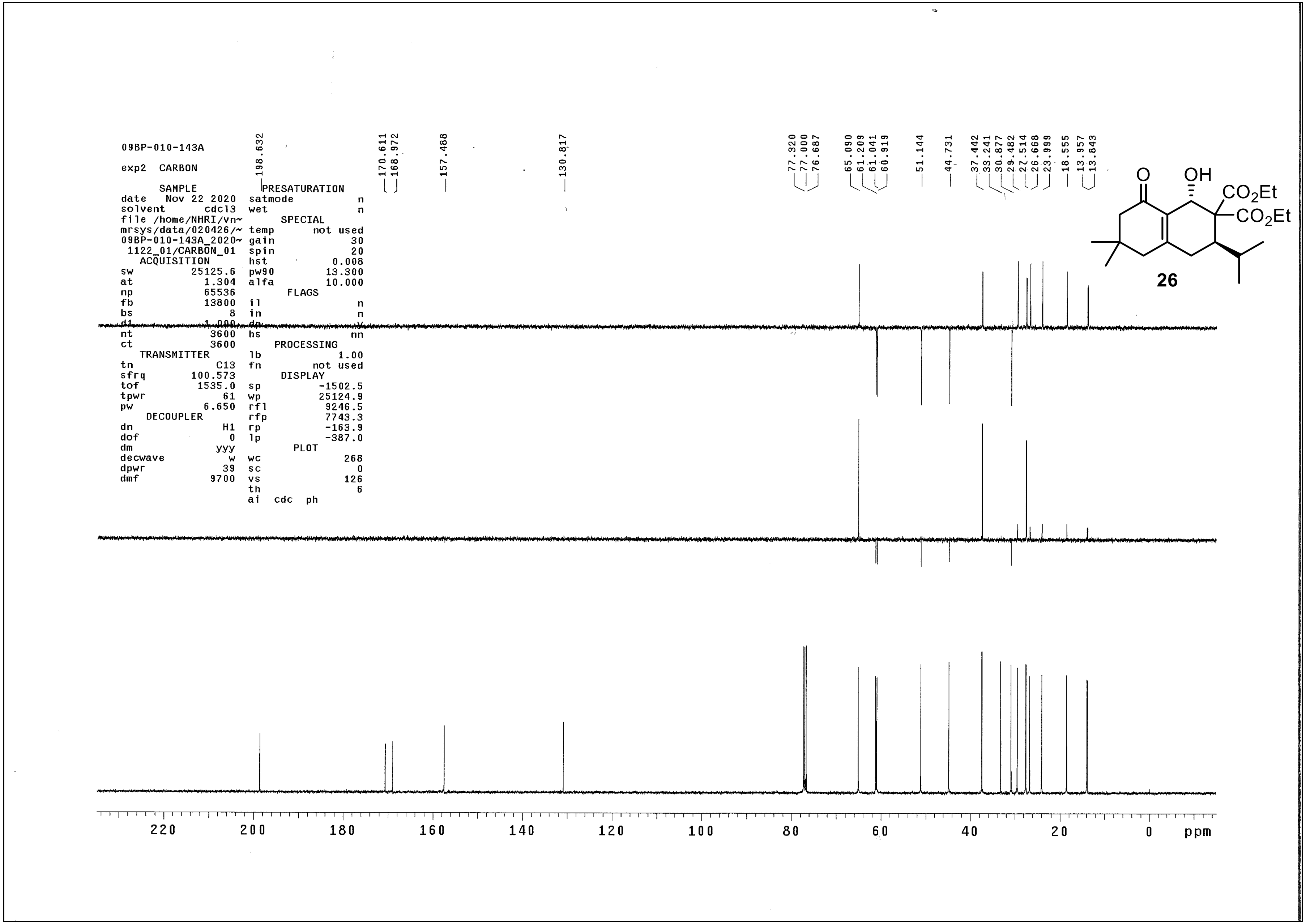

$^{13}C$ NMR + DEPT spectra for compound **26**

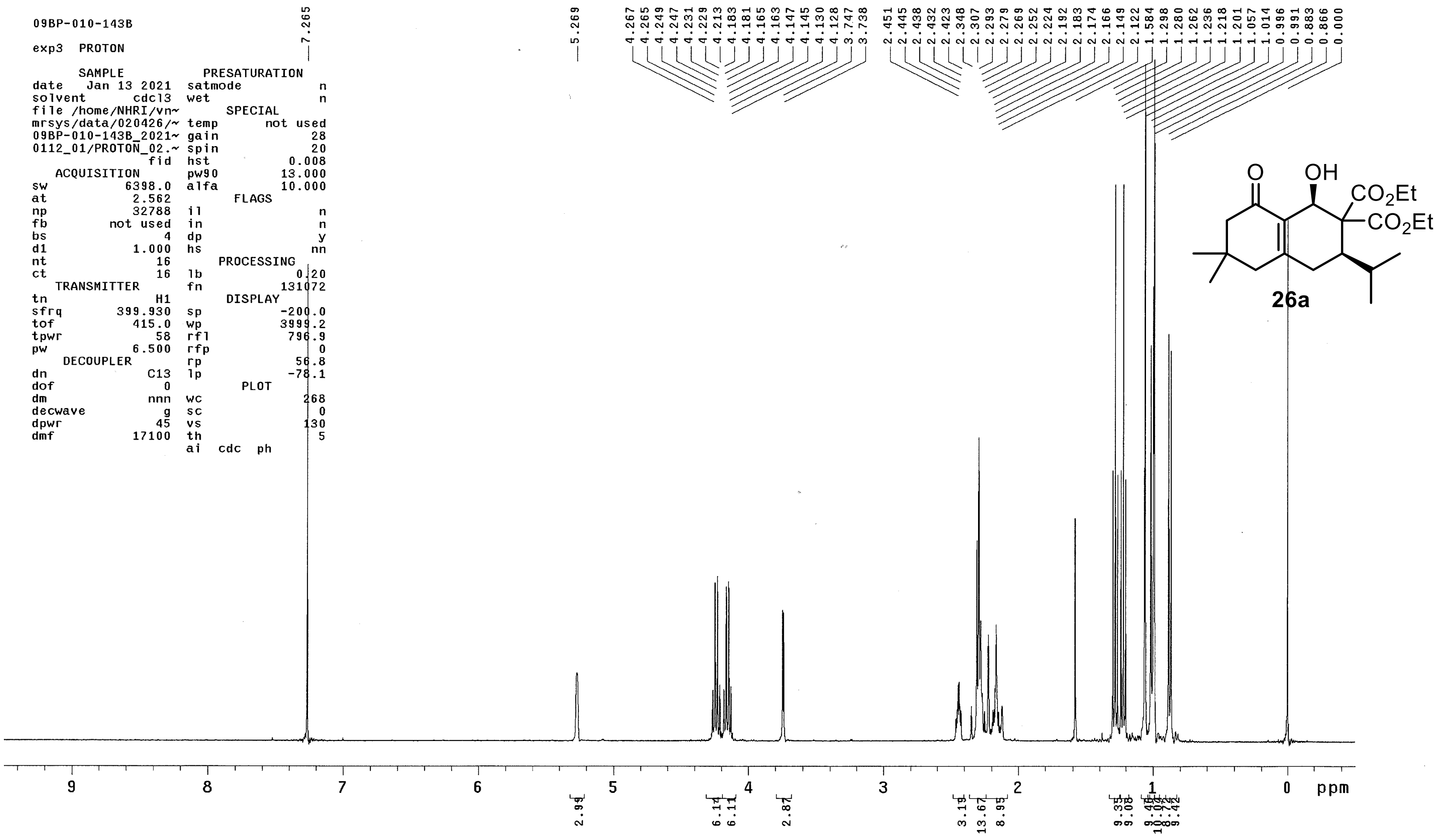


$^{1}$H NMR spectrum for compound **26a**

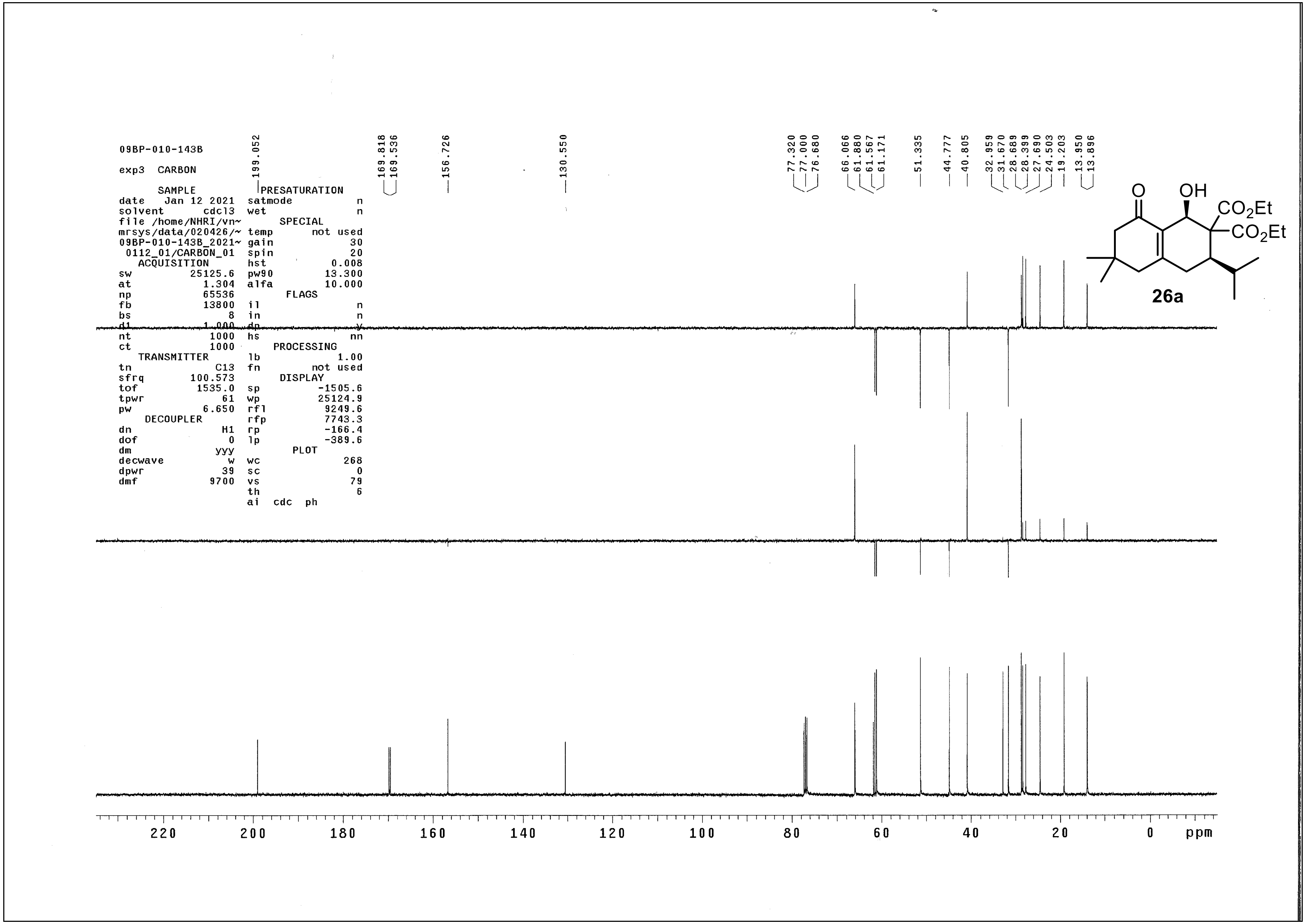

$^{13}$C NMR + DEPT spectra for compound **26a**

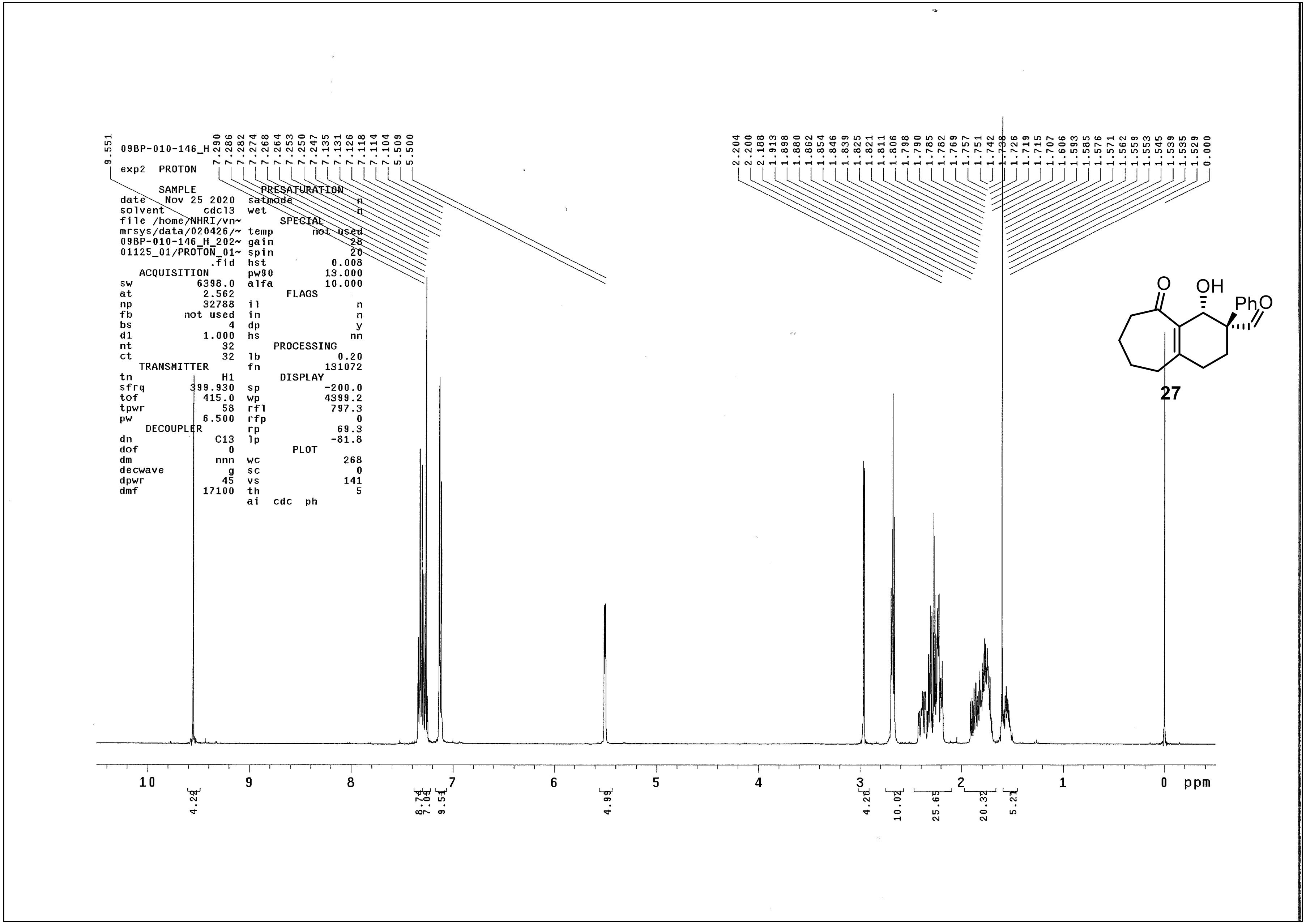

$^{1}$H NMR spectrum for compound **27**

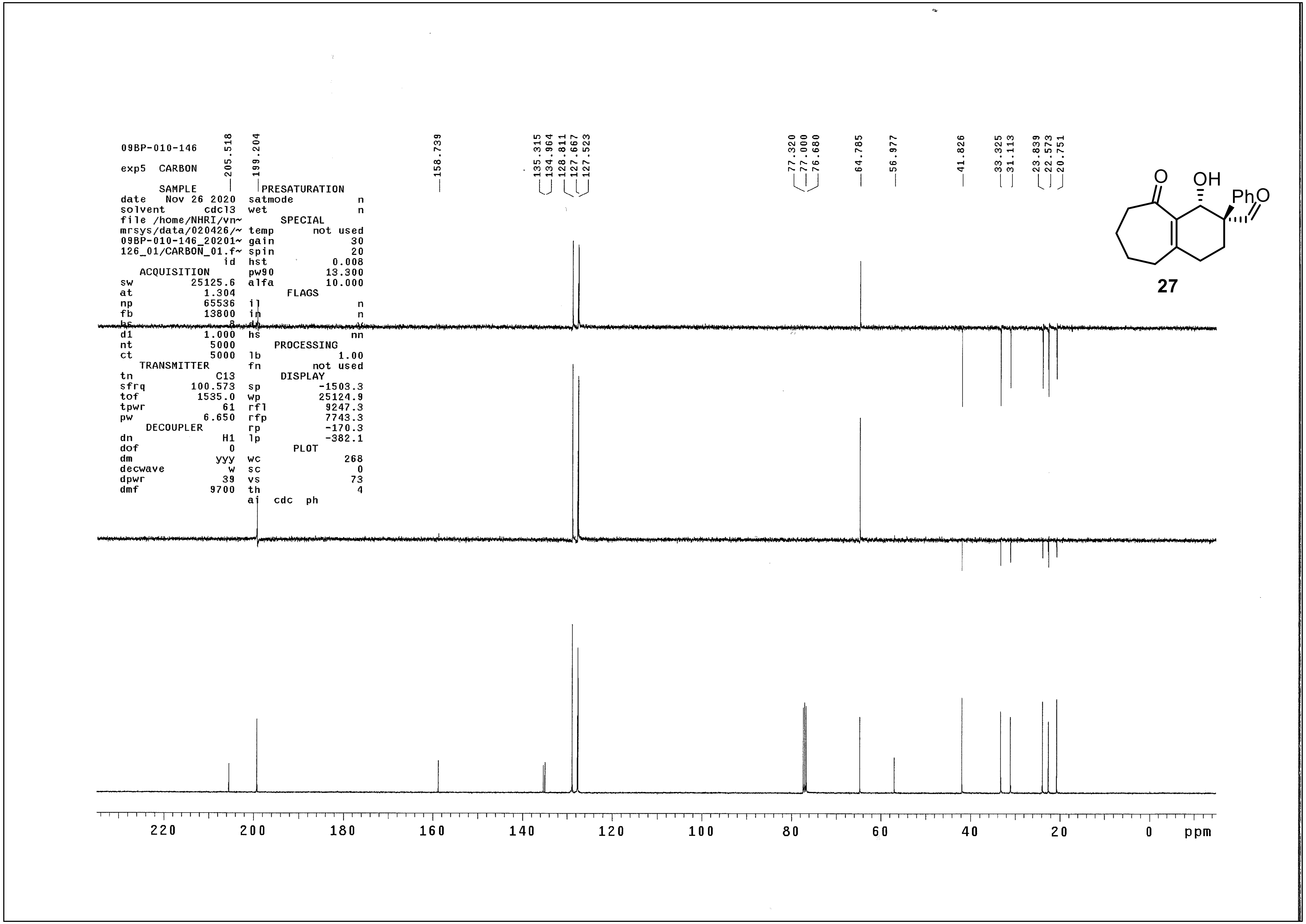


$^{13}$C NMR + DEPT spectra for compound **27**

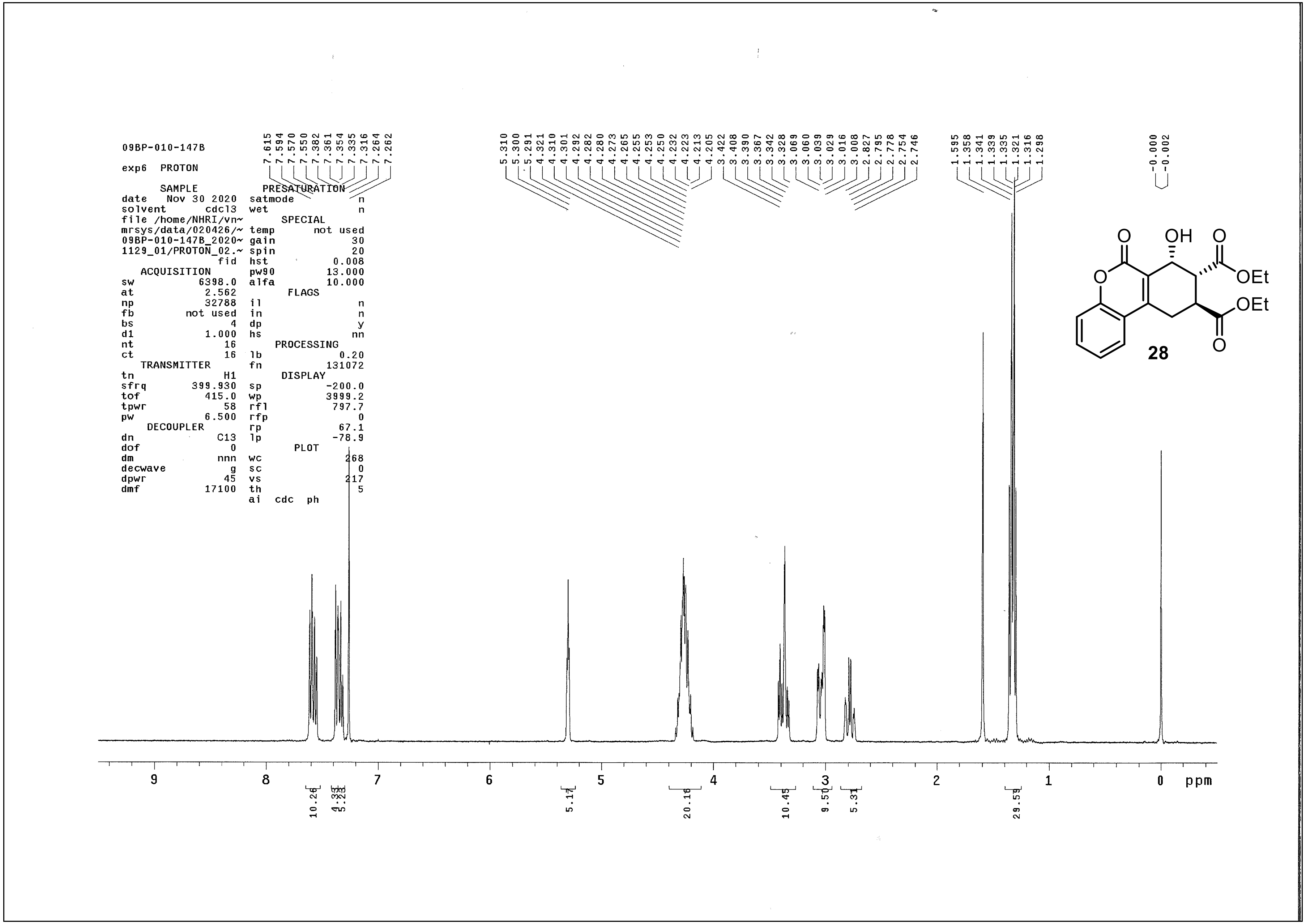

$^{1}$H NMR spectrum for compound **28**

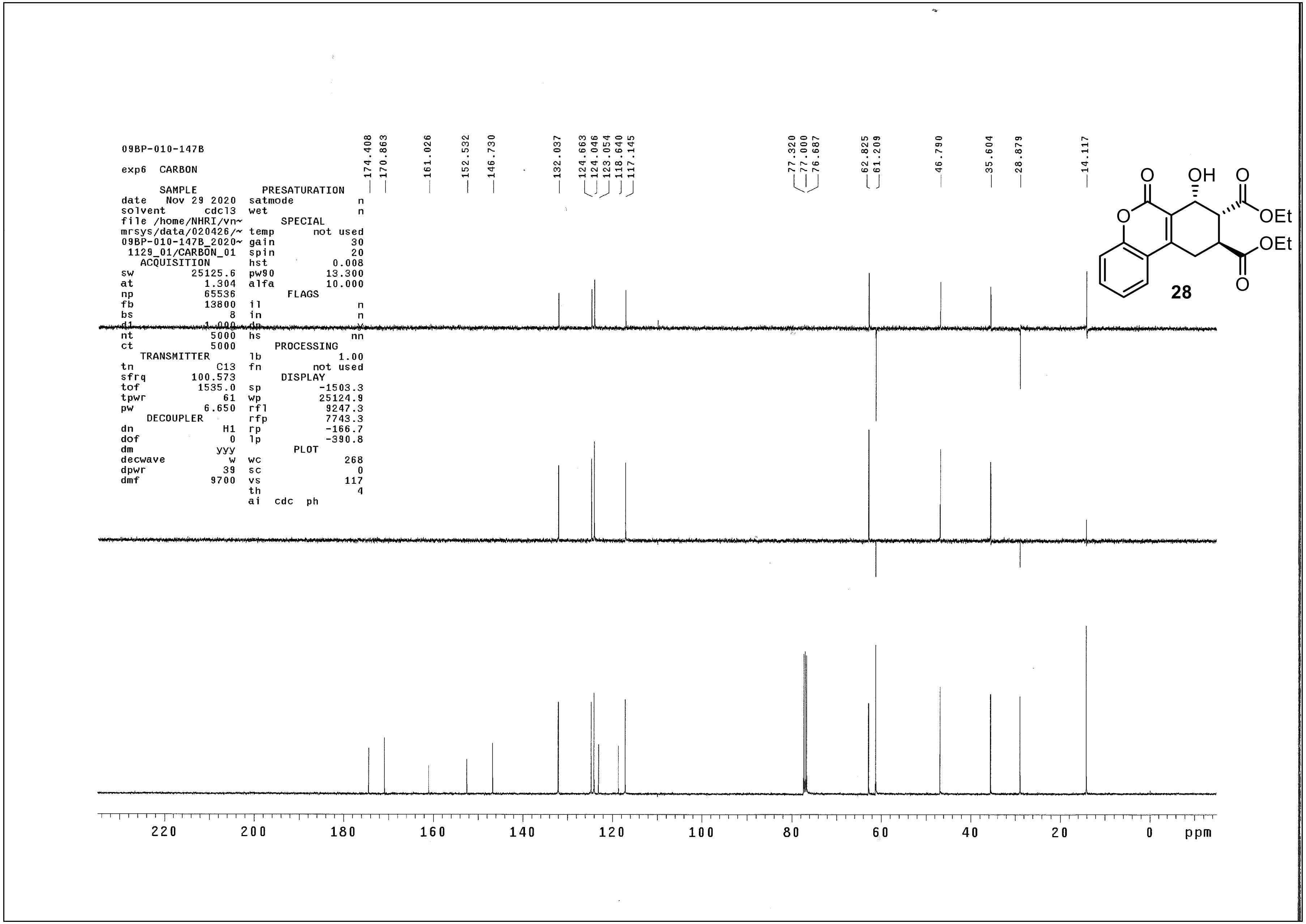


$^{13}$C NMR + DEPT spectra for compound **28**

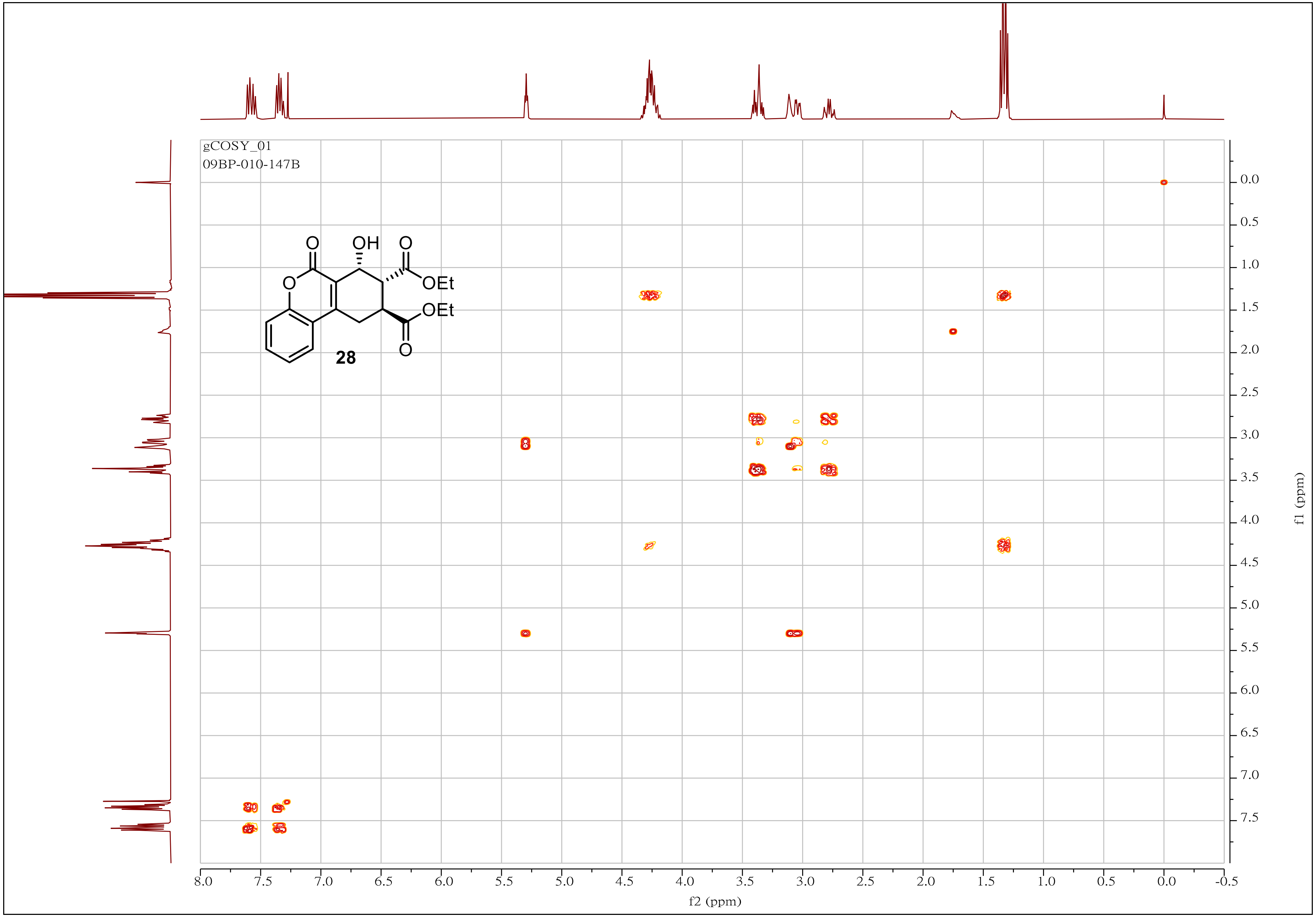


$^{1}$H-$^{1}$H COSY spectrum for compound **28**

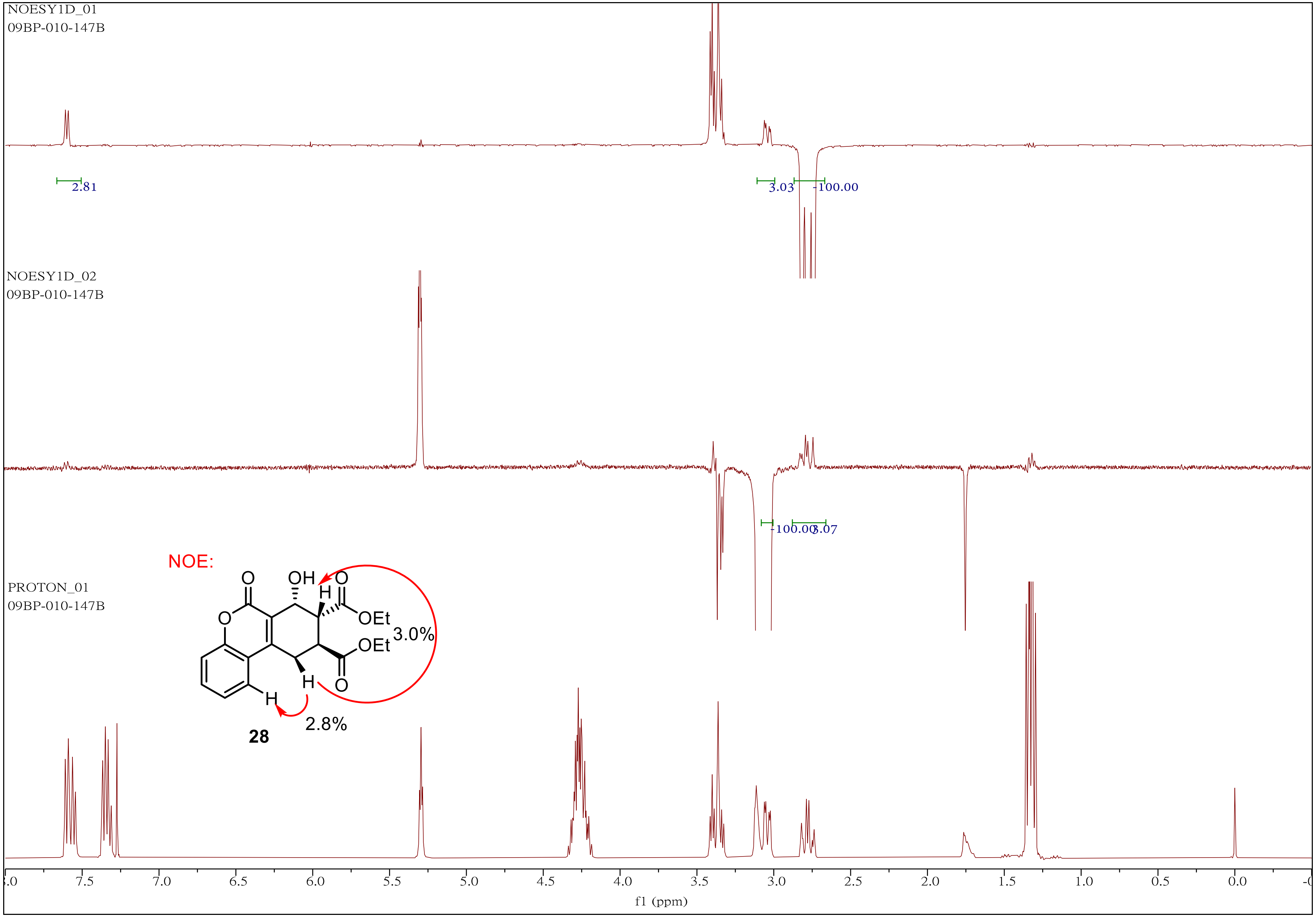


1D NOESY spectra for compound **28**

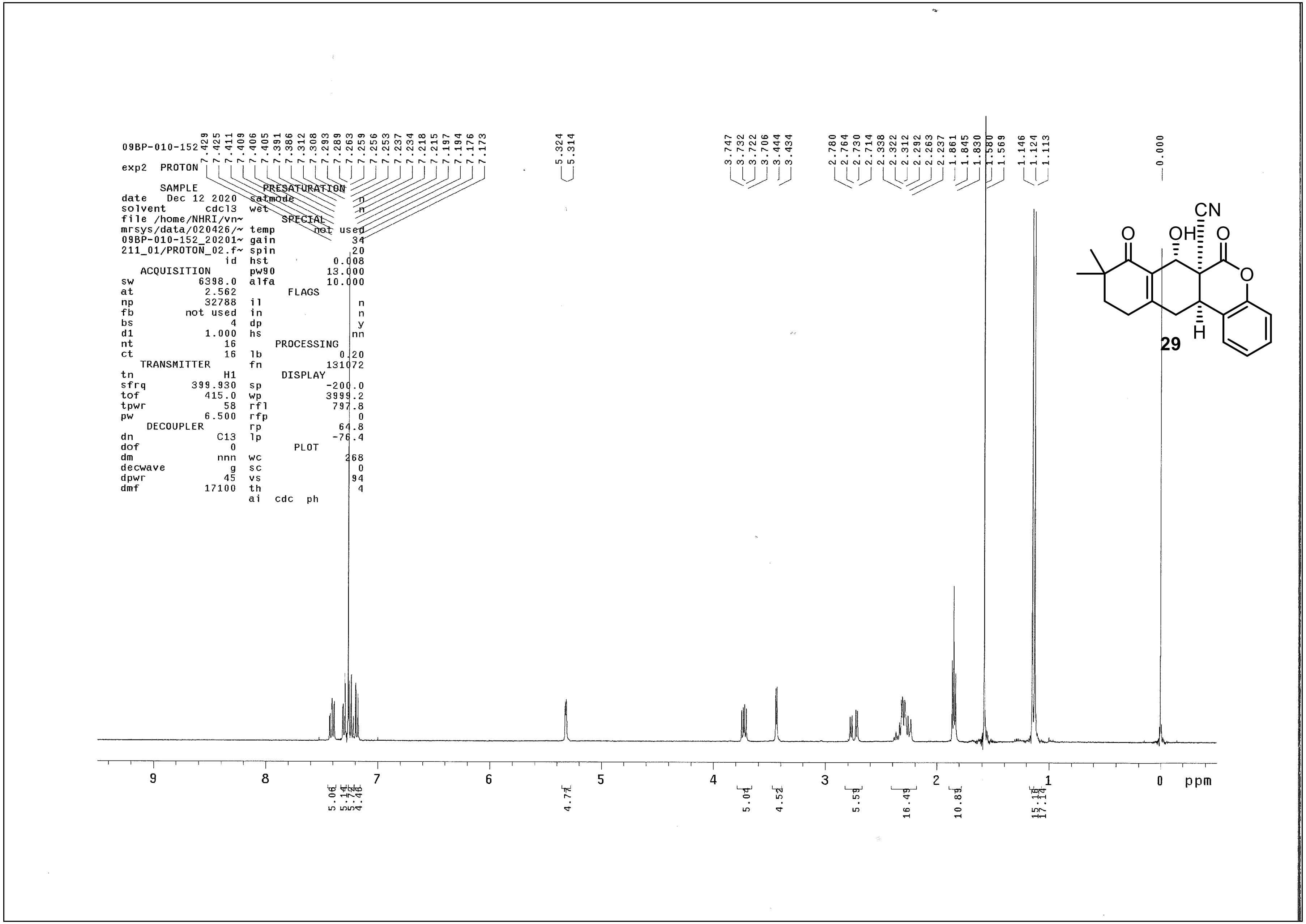

$^{1}$H NMR spectrum for compound **29**

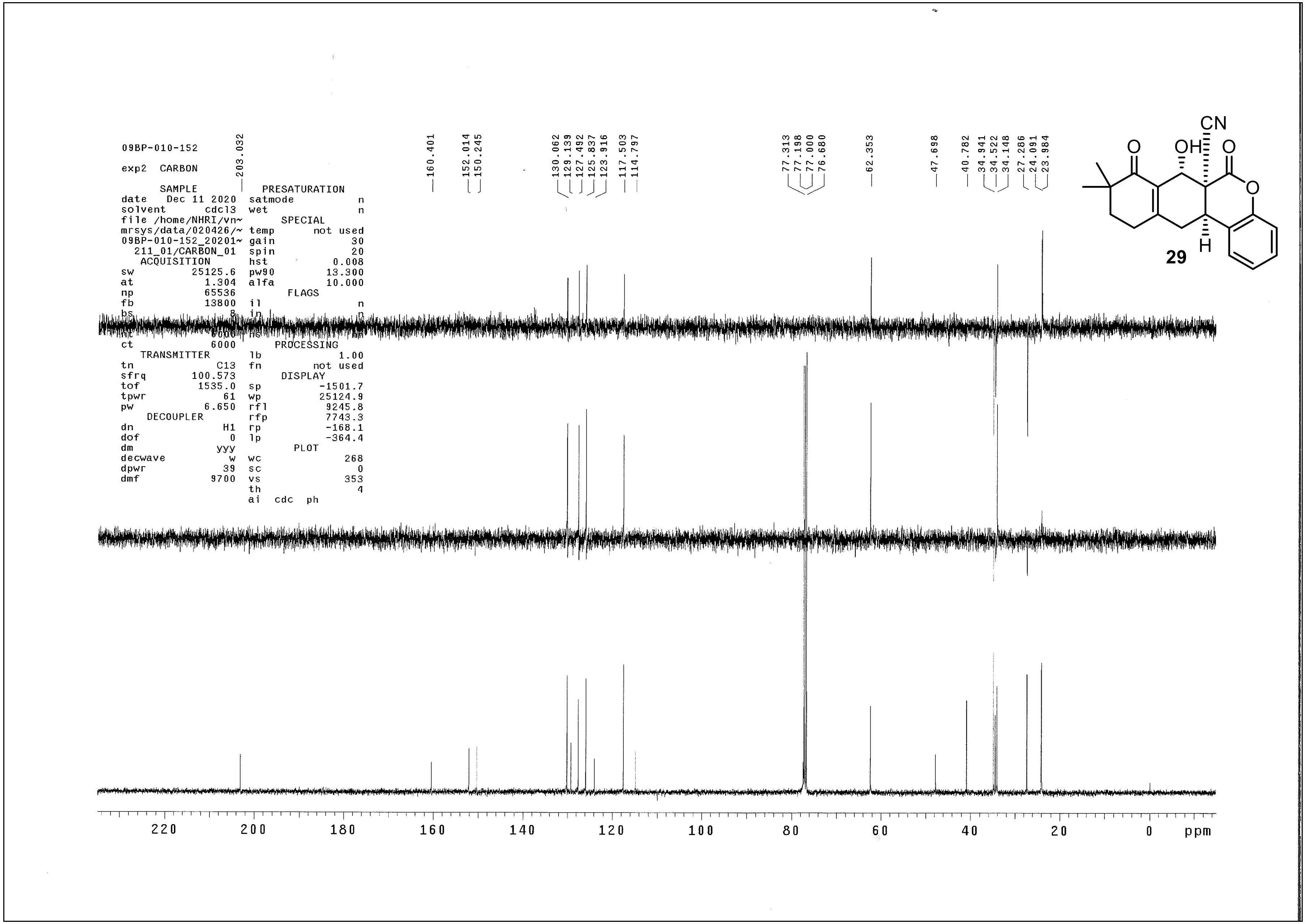


$^{13}$C NMR + DEPT spectra for compound **29**

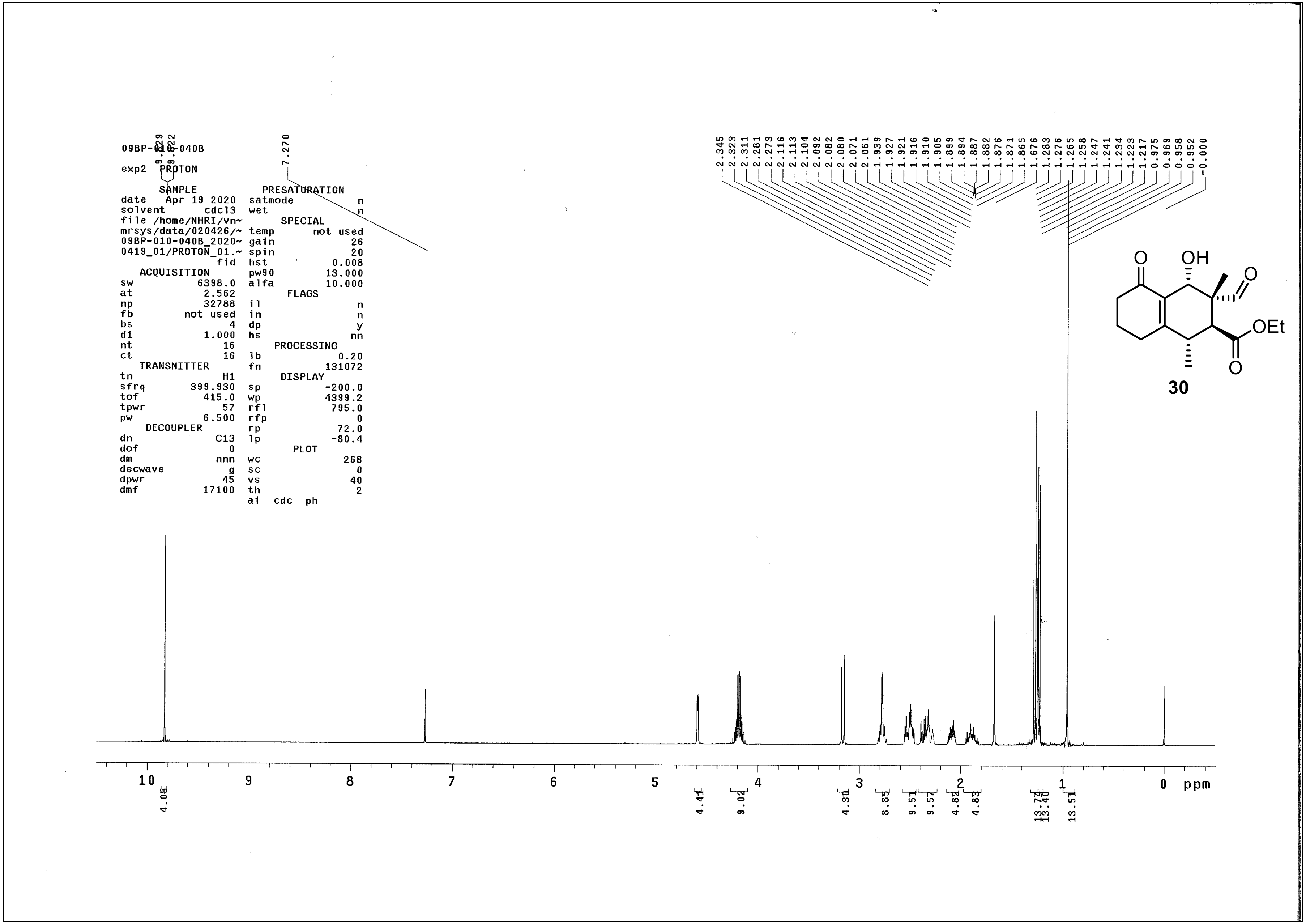

$^{1}$H NMR spectrum for compound **30**

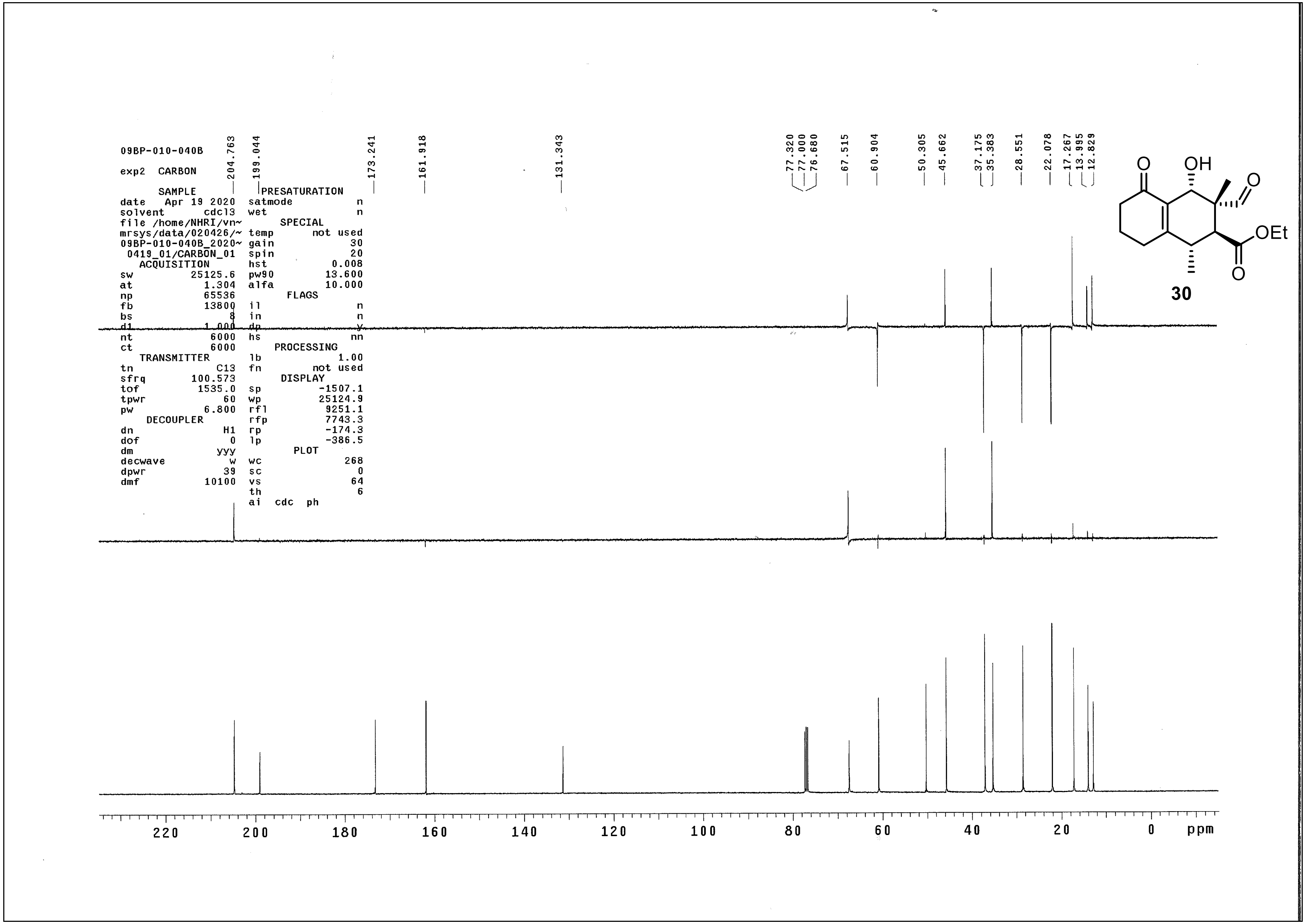


$^{13}$C NMR + DEPT spectra for compound **30**

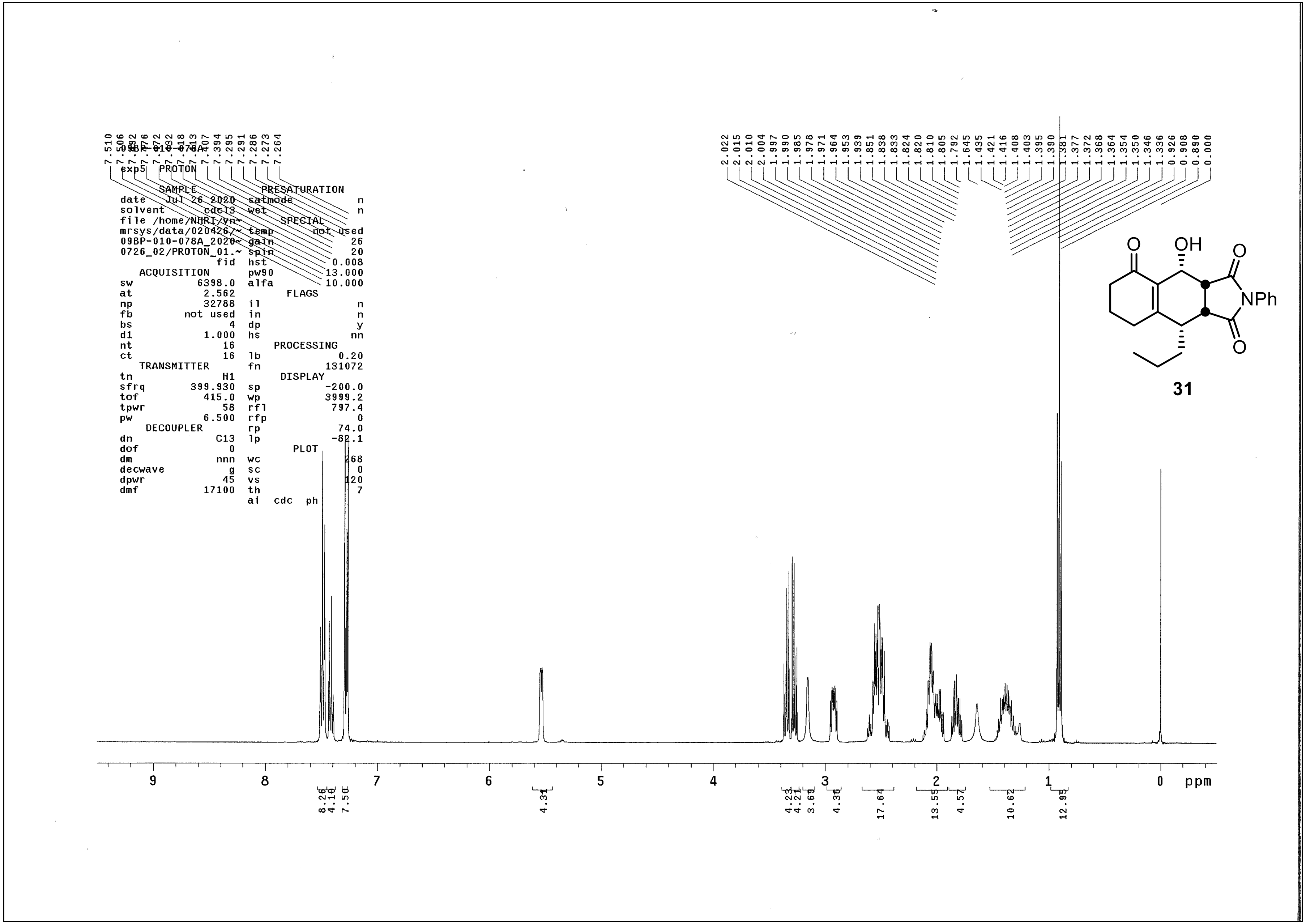

$^{1}$H NMR spectrum for compound **31**

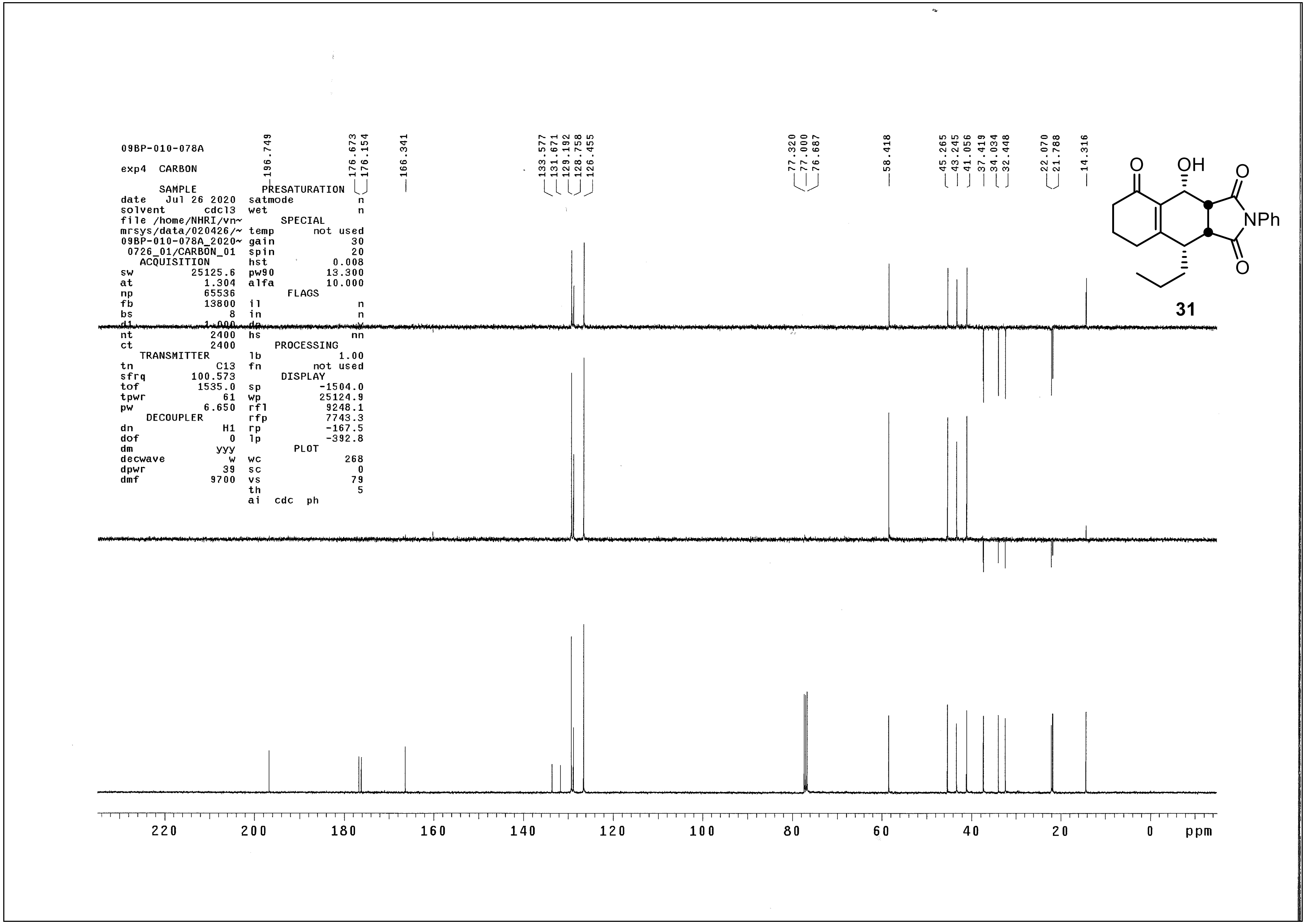

$^{13}C$ NMR + DEPT spectra for compound **31**

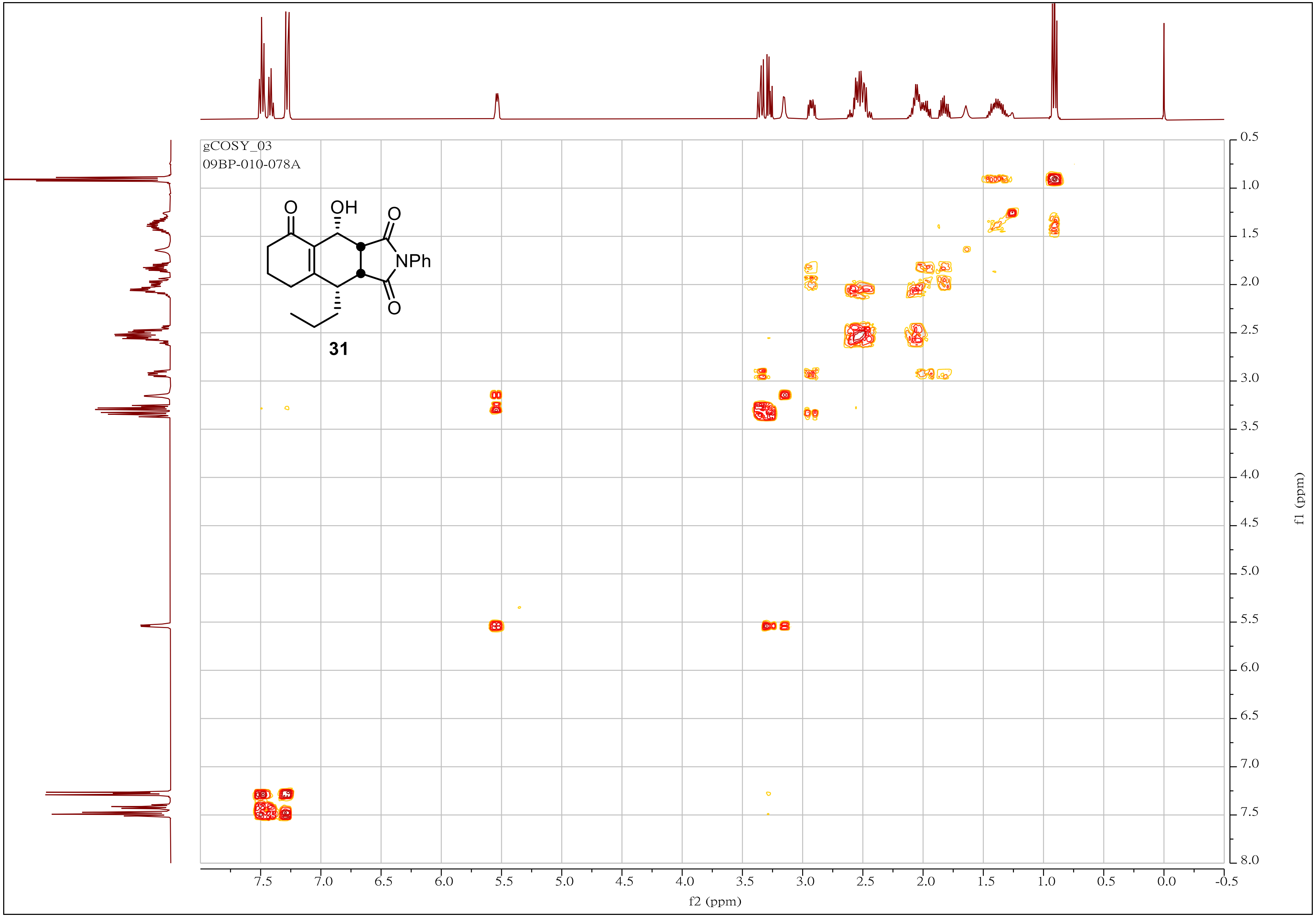


$^{1}$H-$^{1}$H COSY spectrum for compound **31**

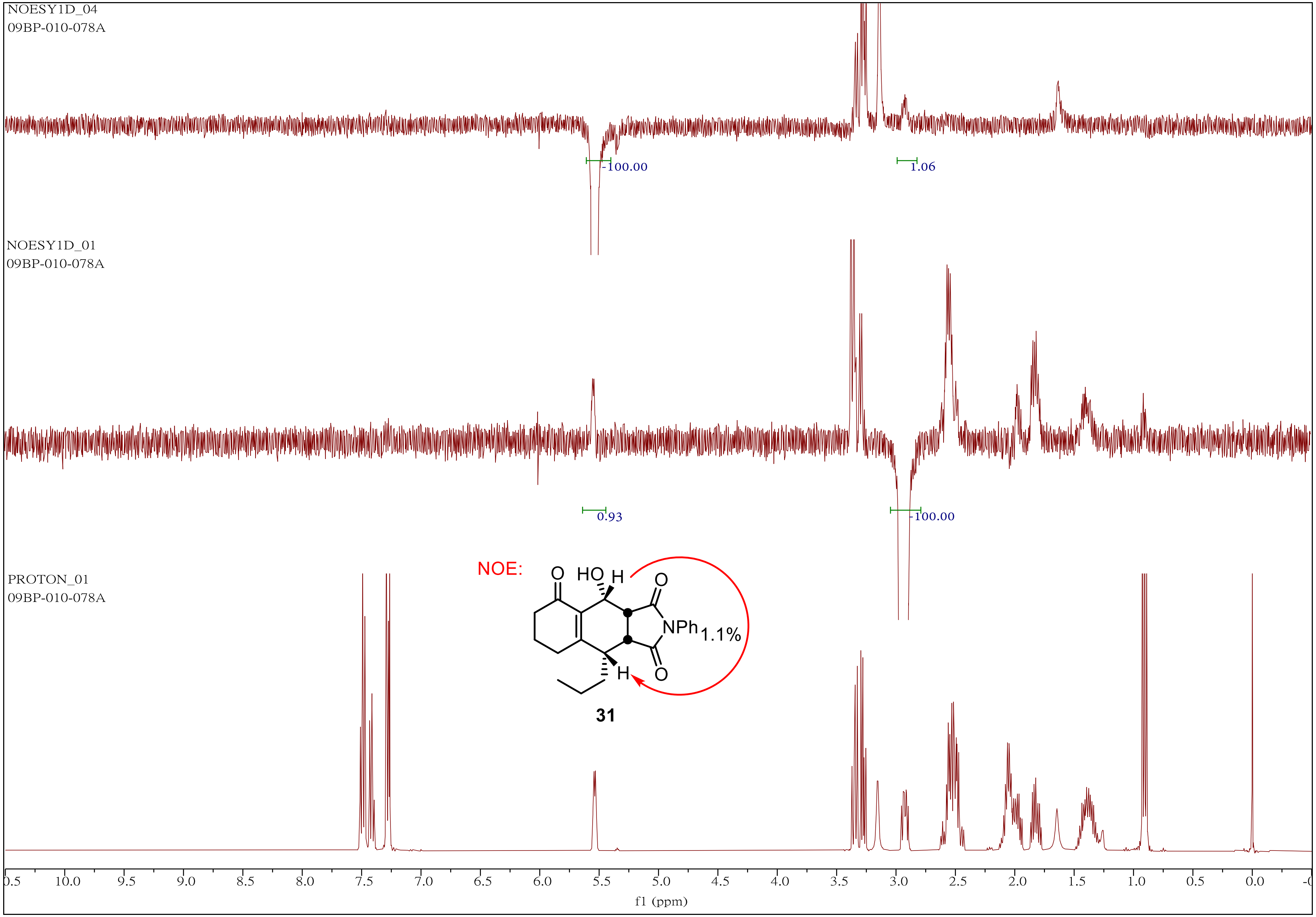


1D NOESY spectra for compound **31**

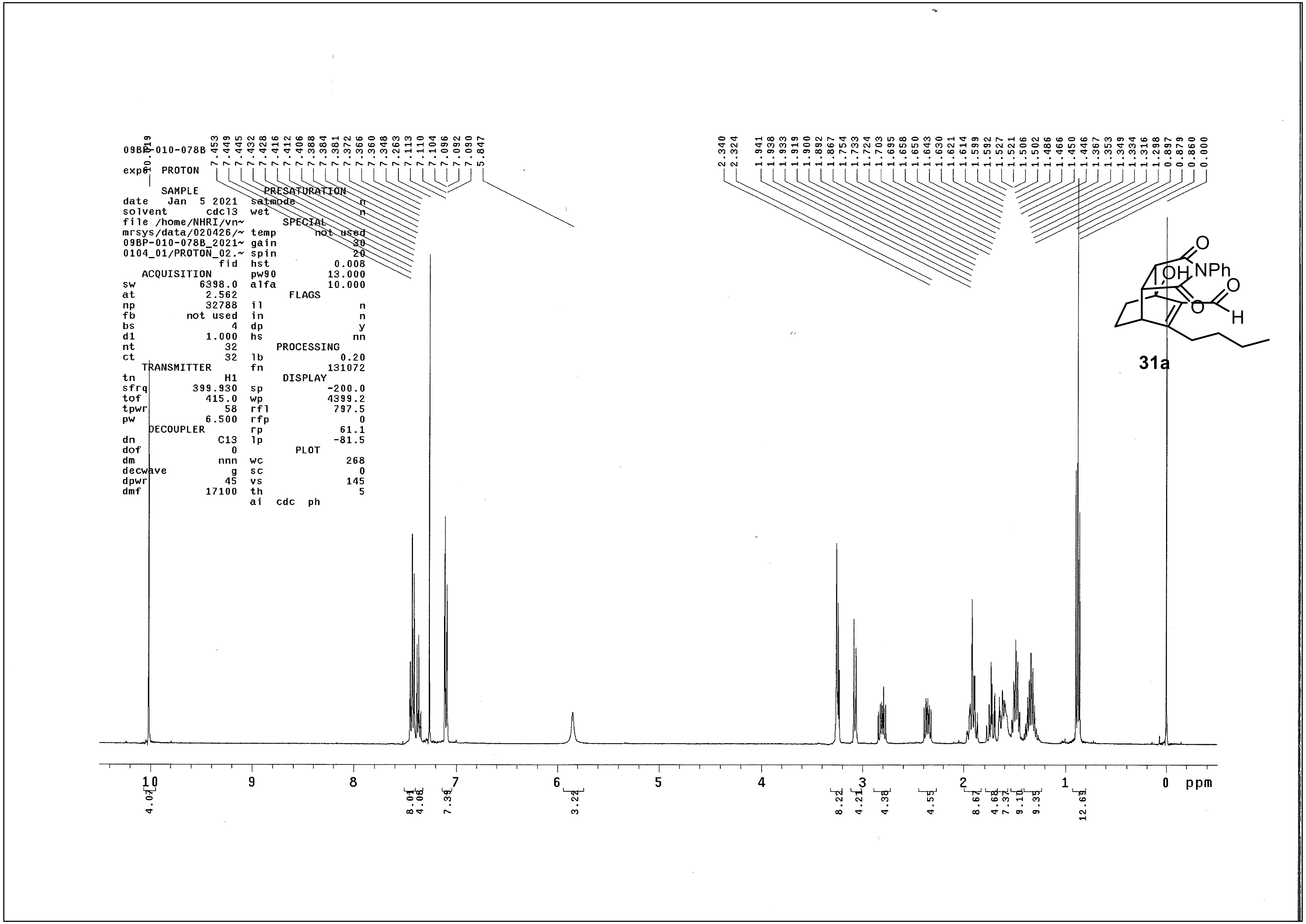

$^{1}$H NMR spectrum for compound **31a**

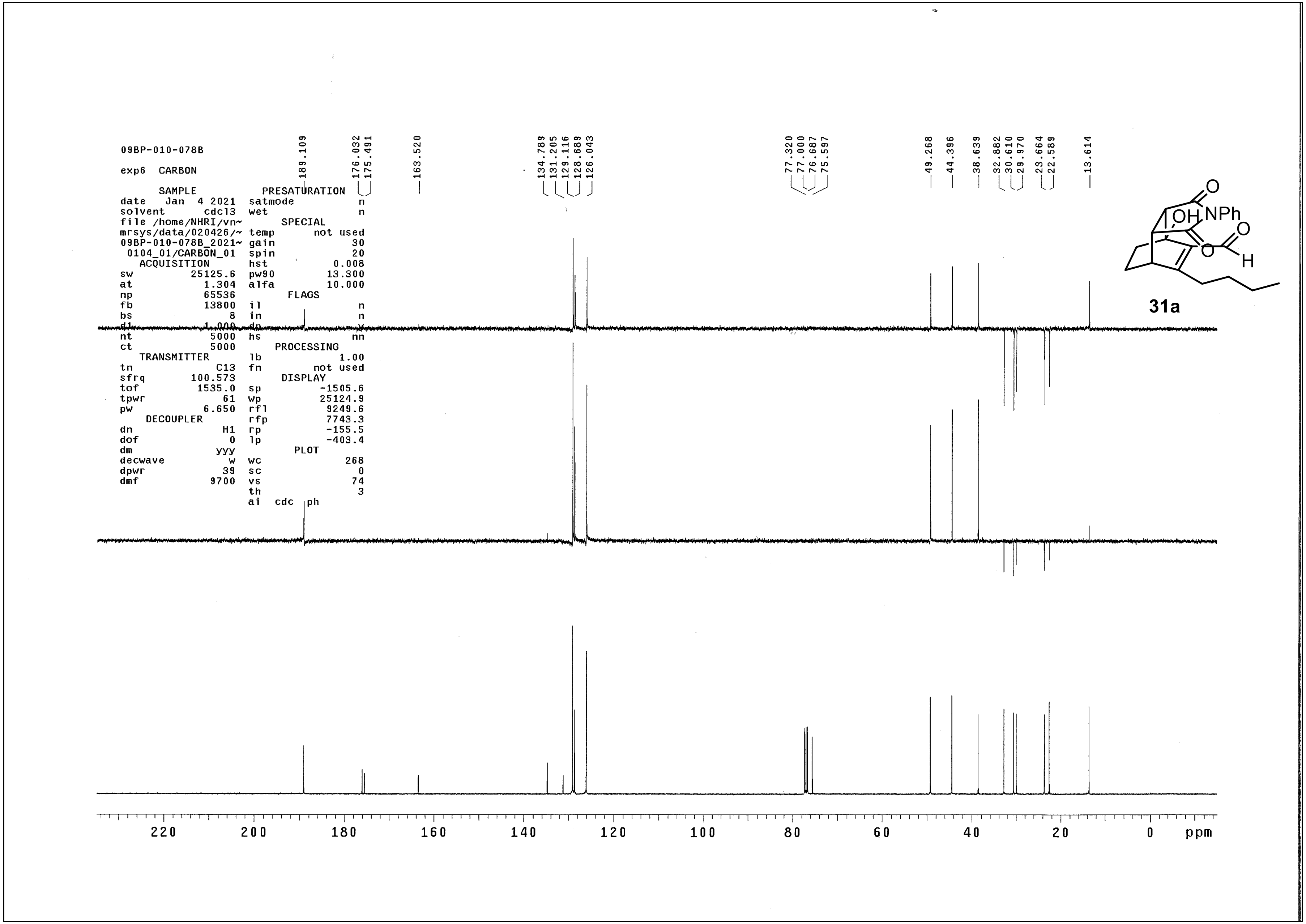


$^{13}$C NMR + DEPT spectra for compound **31a**

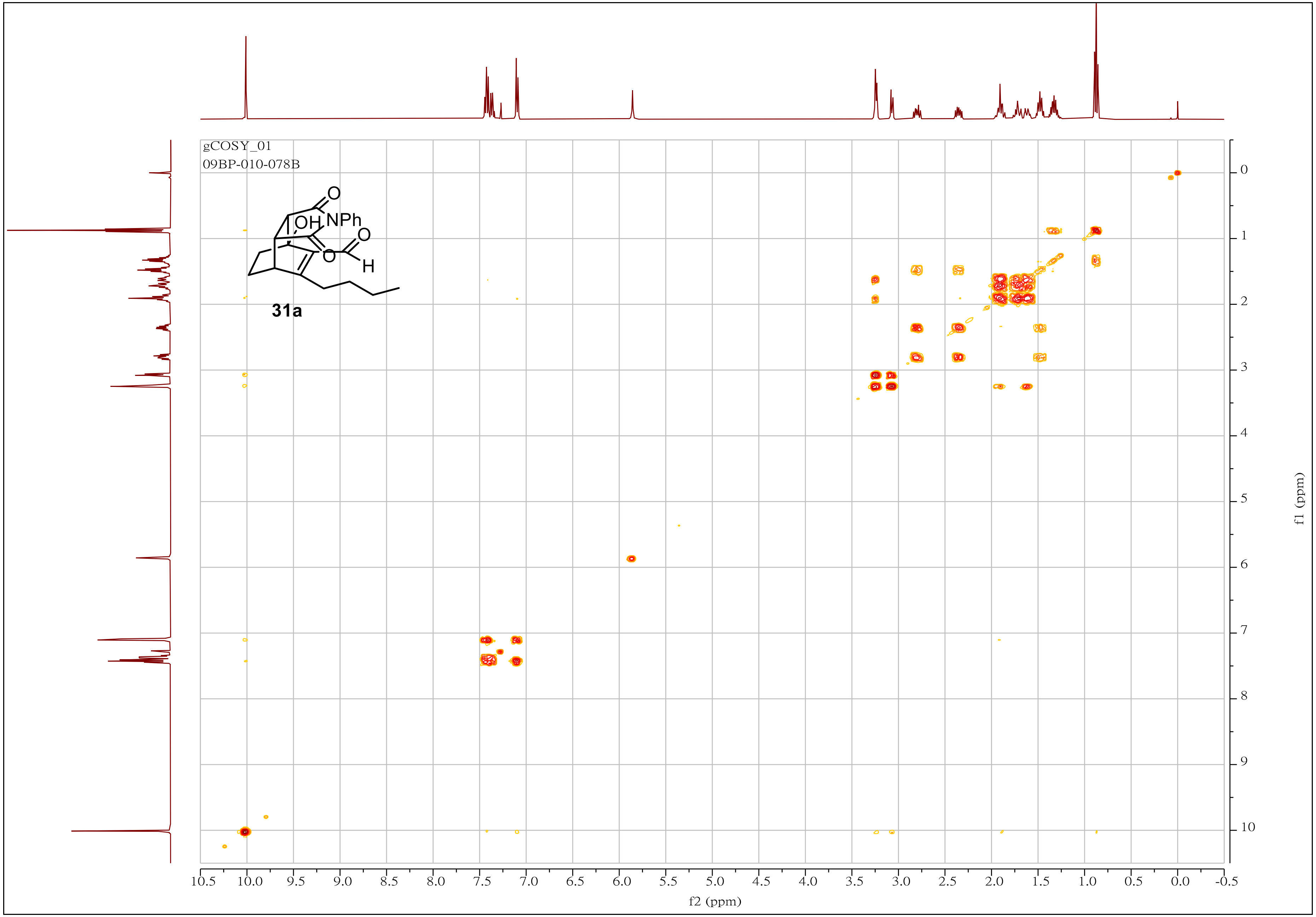


$^{1}H$-$^{1}H$ COSY spectrum for compound **31a**

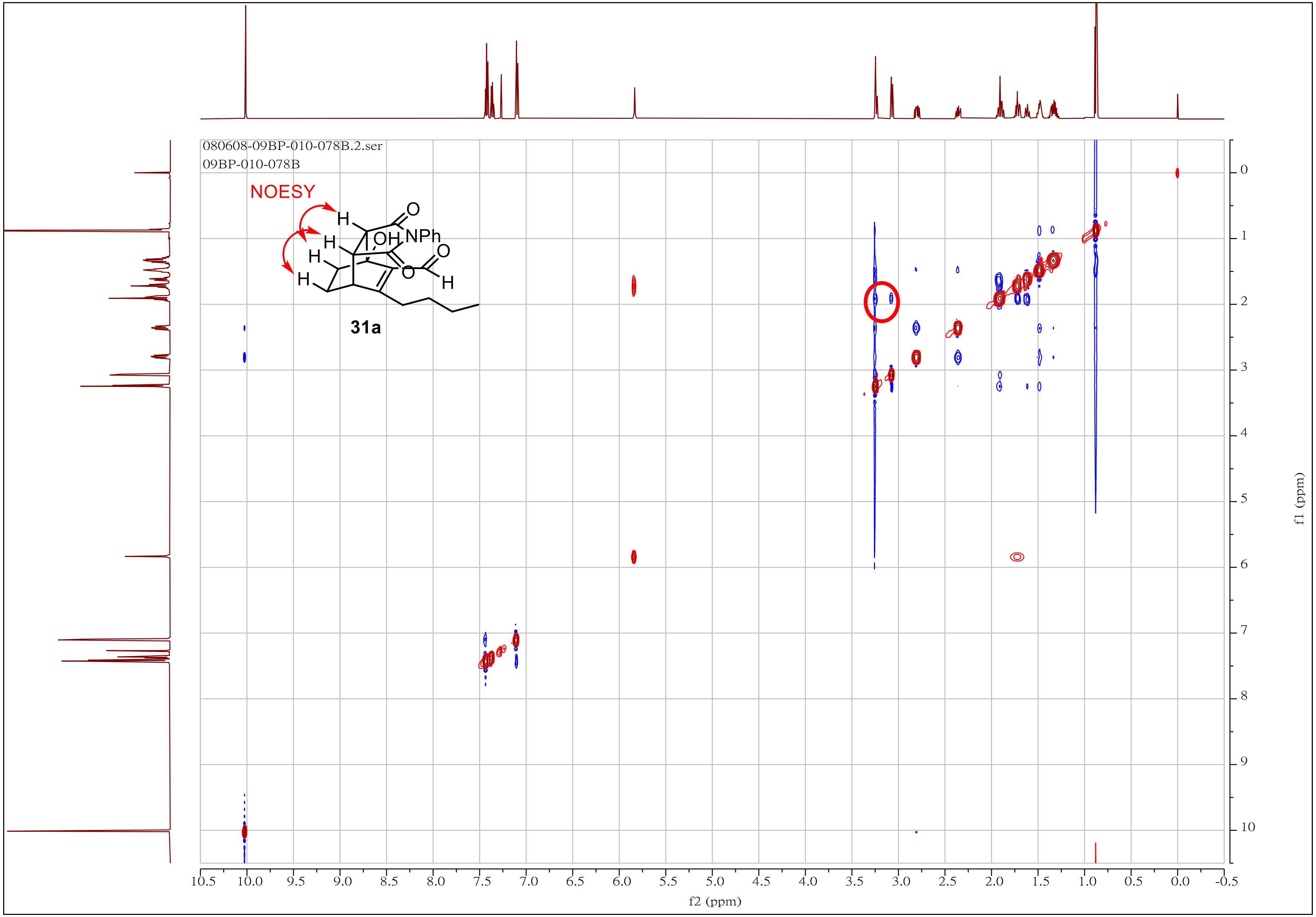


2D NOESY spectrum for compound **31a**

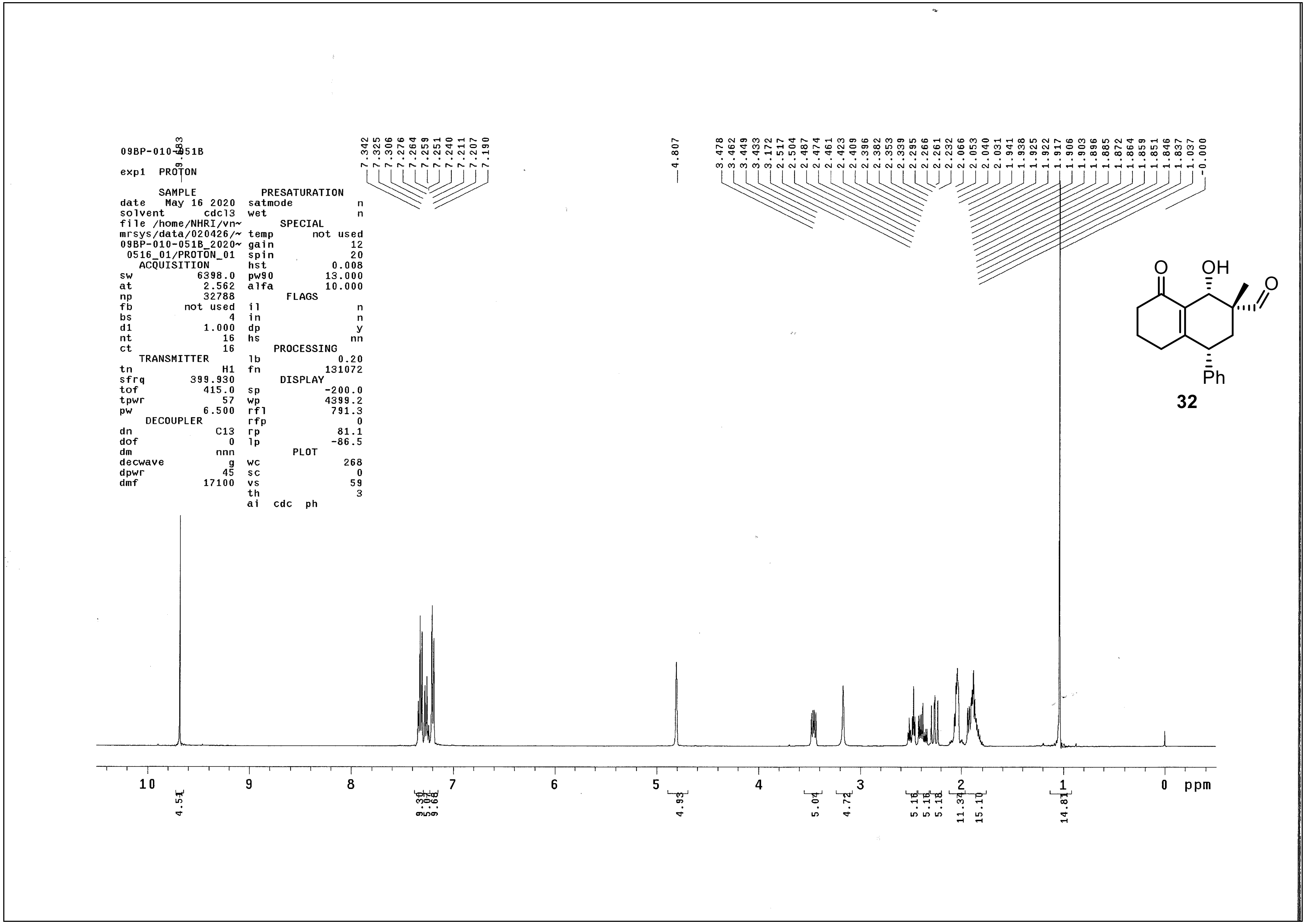


$^{1}$H NMR spectrum for compound **32**

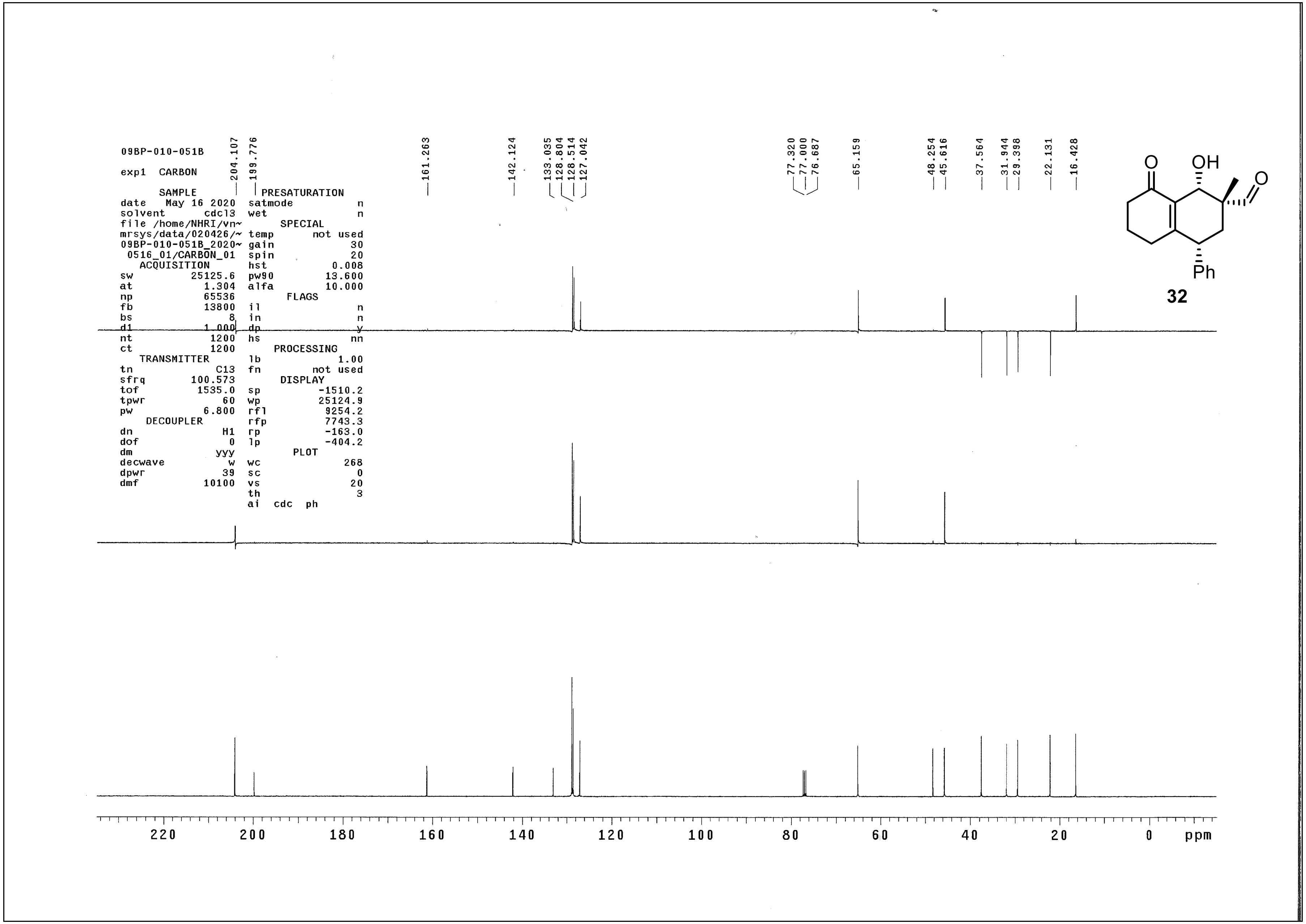


$^{13}$C NMR + DEPT spectra for compound **32**

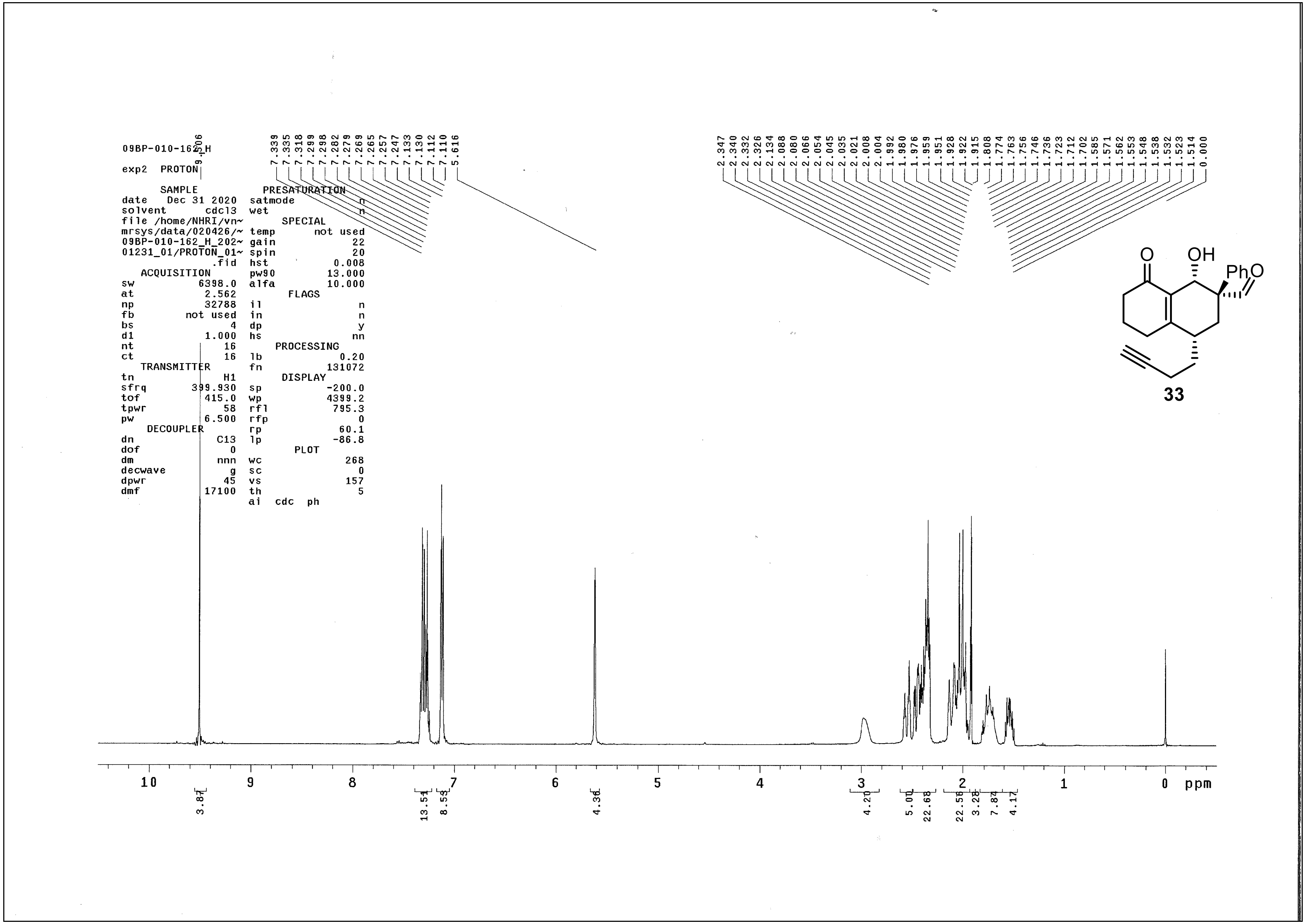

$^{1}$H NMR spectrum for compound **33**

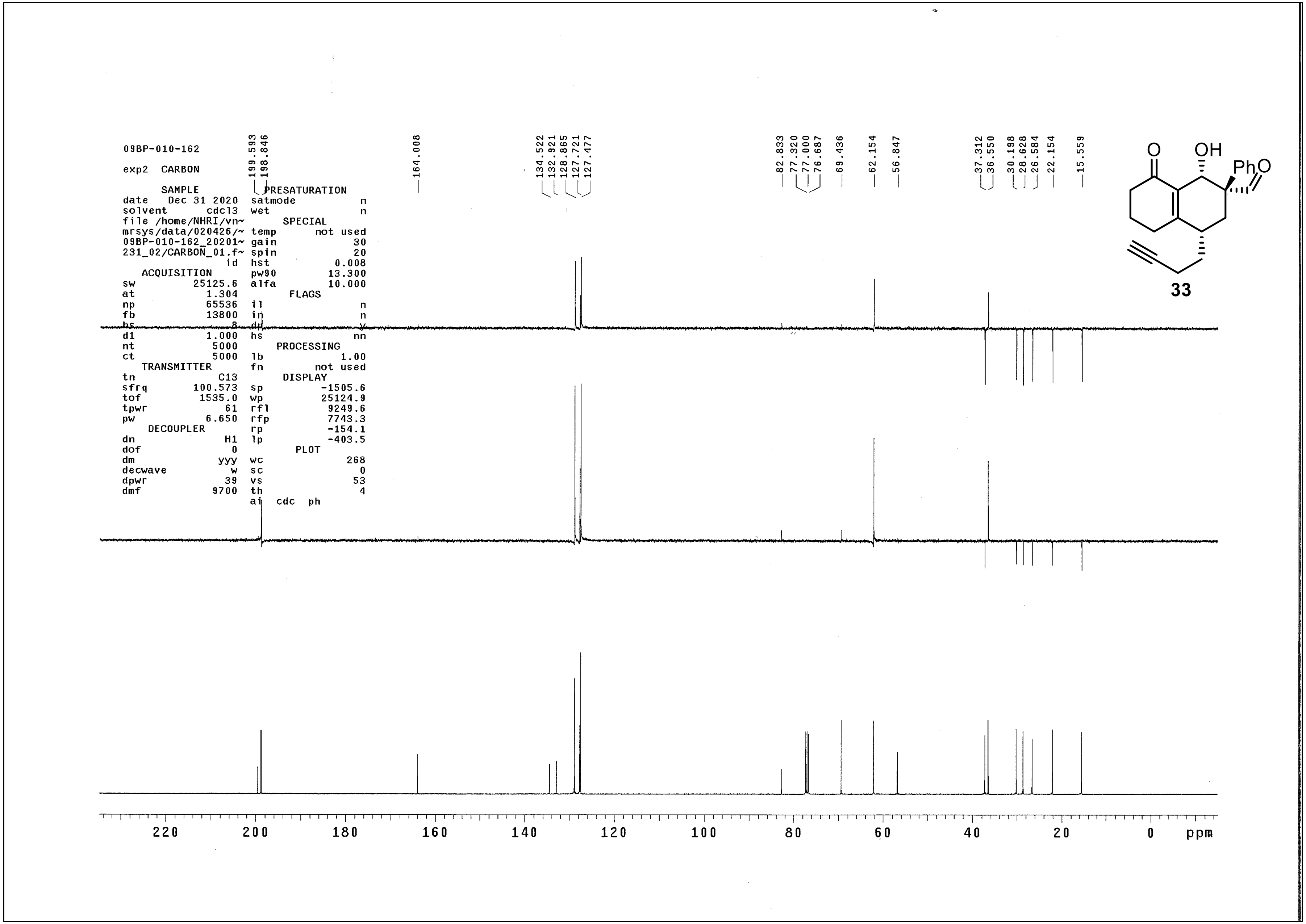

$^{13}$C NMR + DEPT spectra for compound **33**

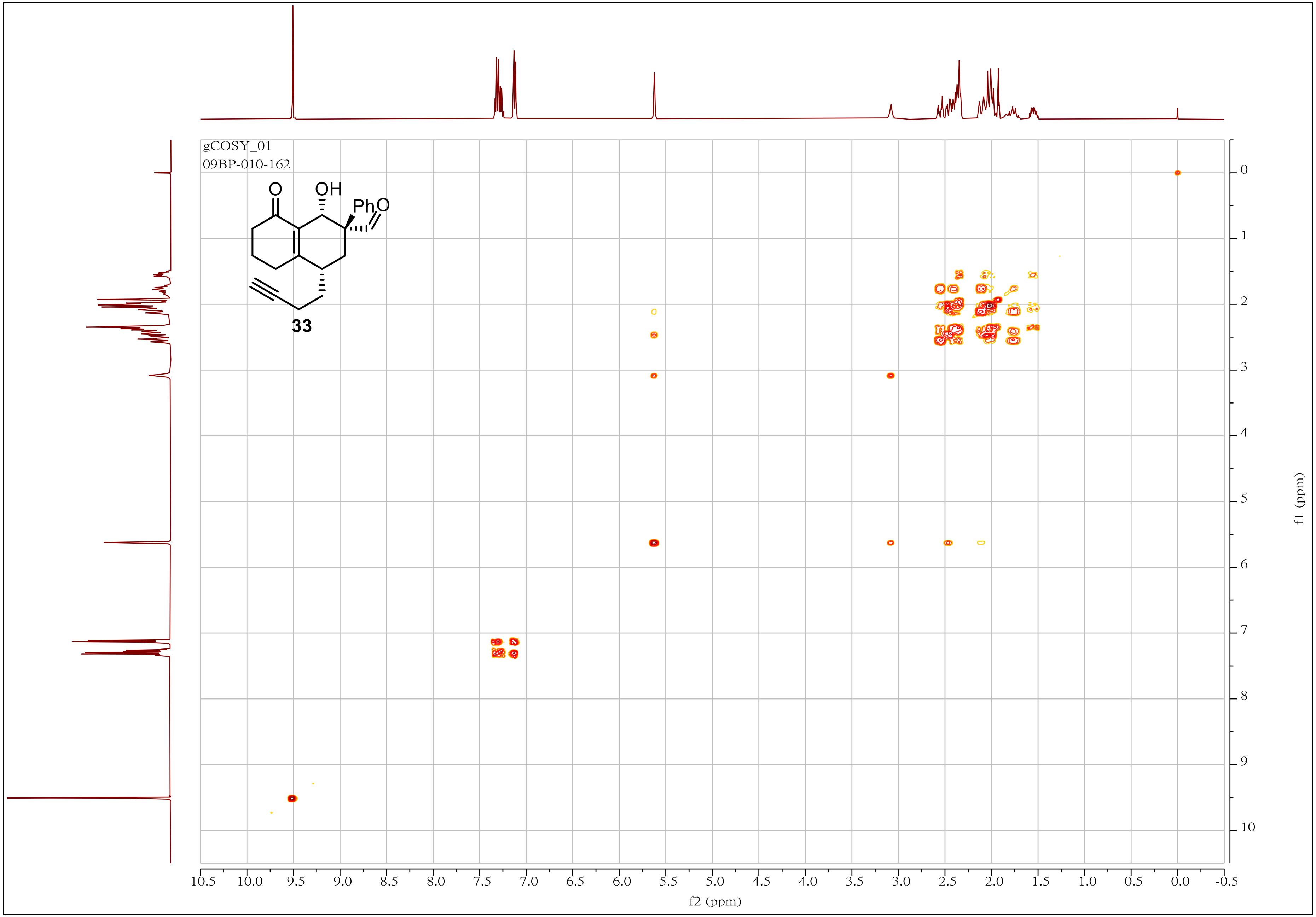


$^{1}$H-$^{1}$H COSY spectrum for compound **33**

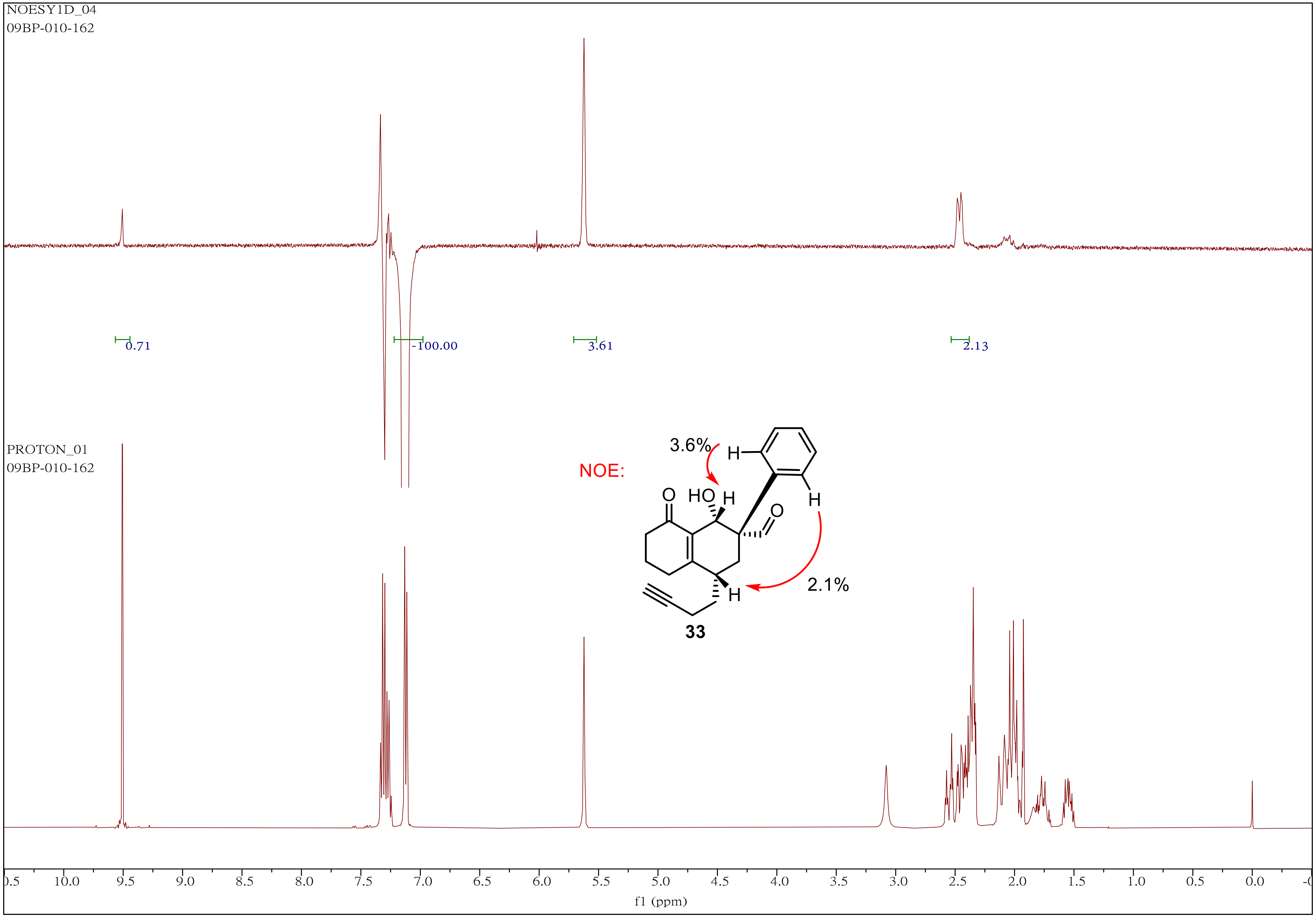


1D NOESY spectra for compound **33**

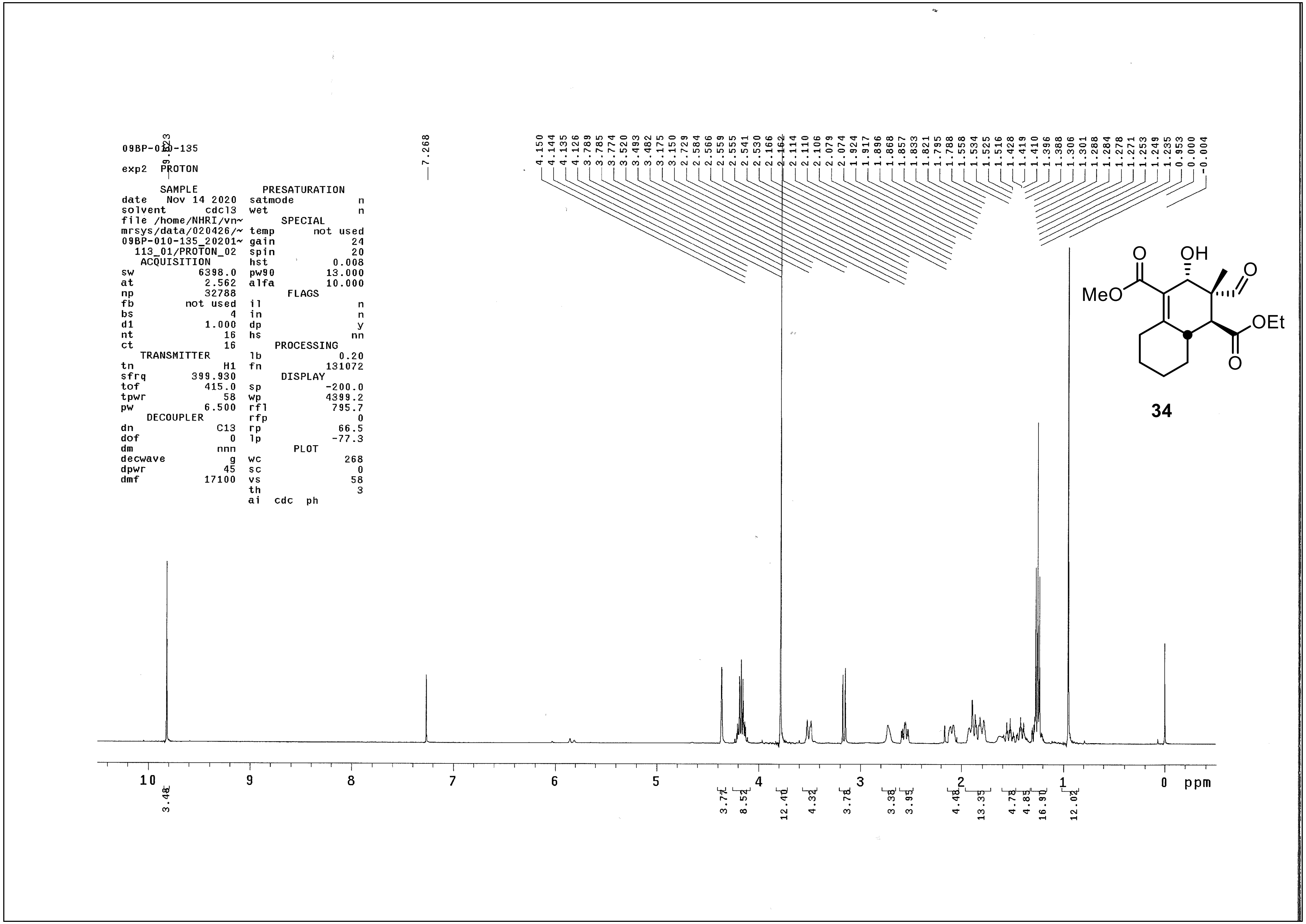

$^{1}$H NMR spectrum for compound **34**

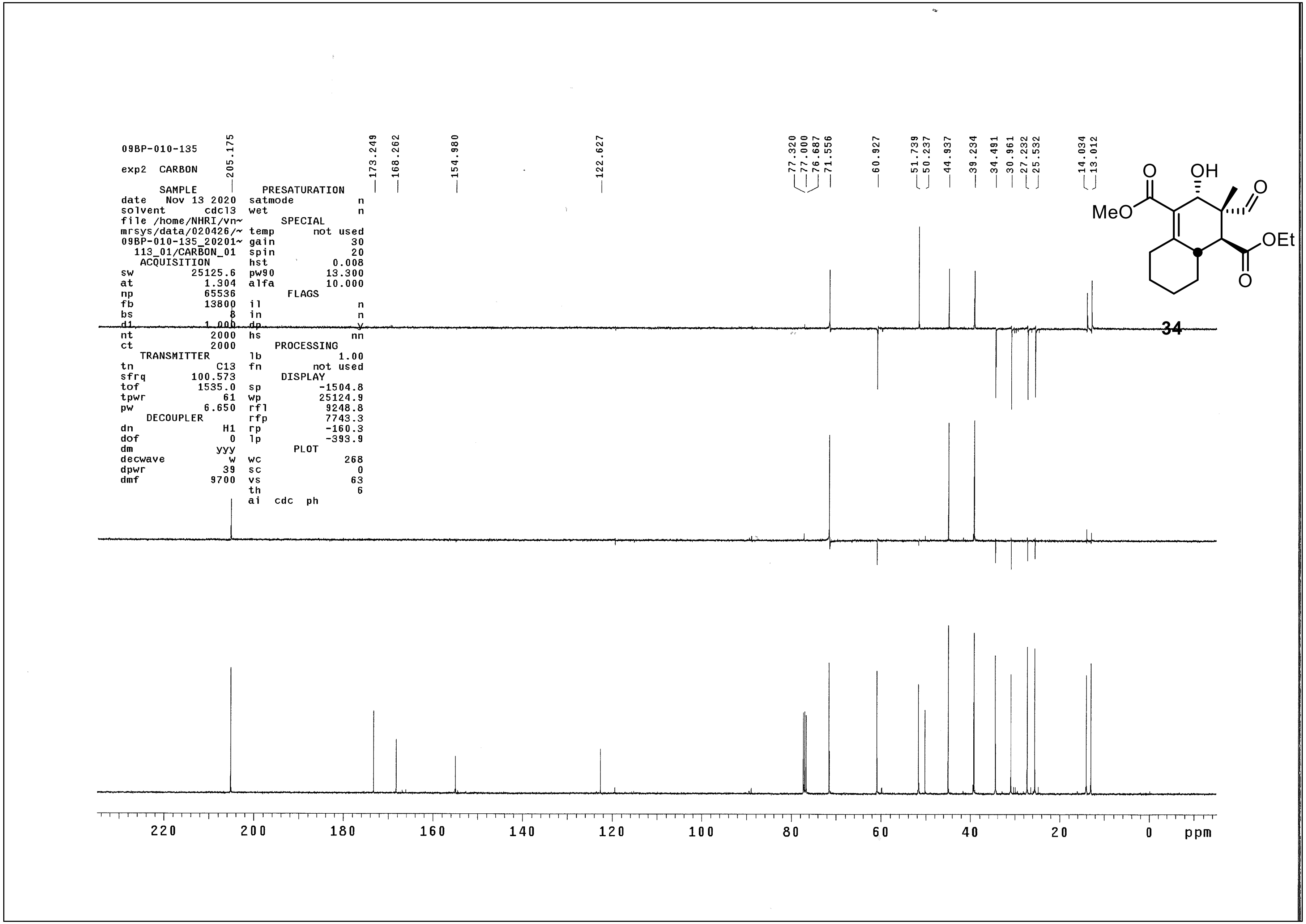


$^{13}$C NMR + DEPT spectra for compound **34**

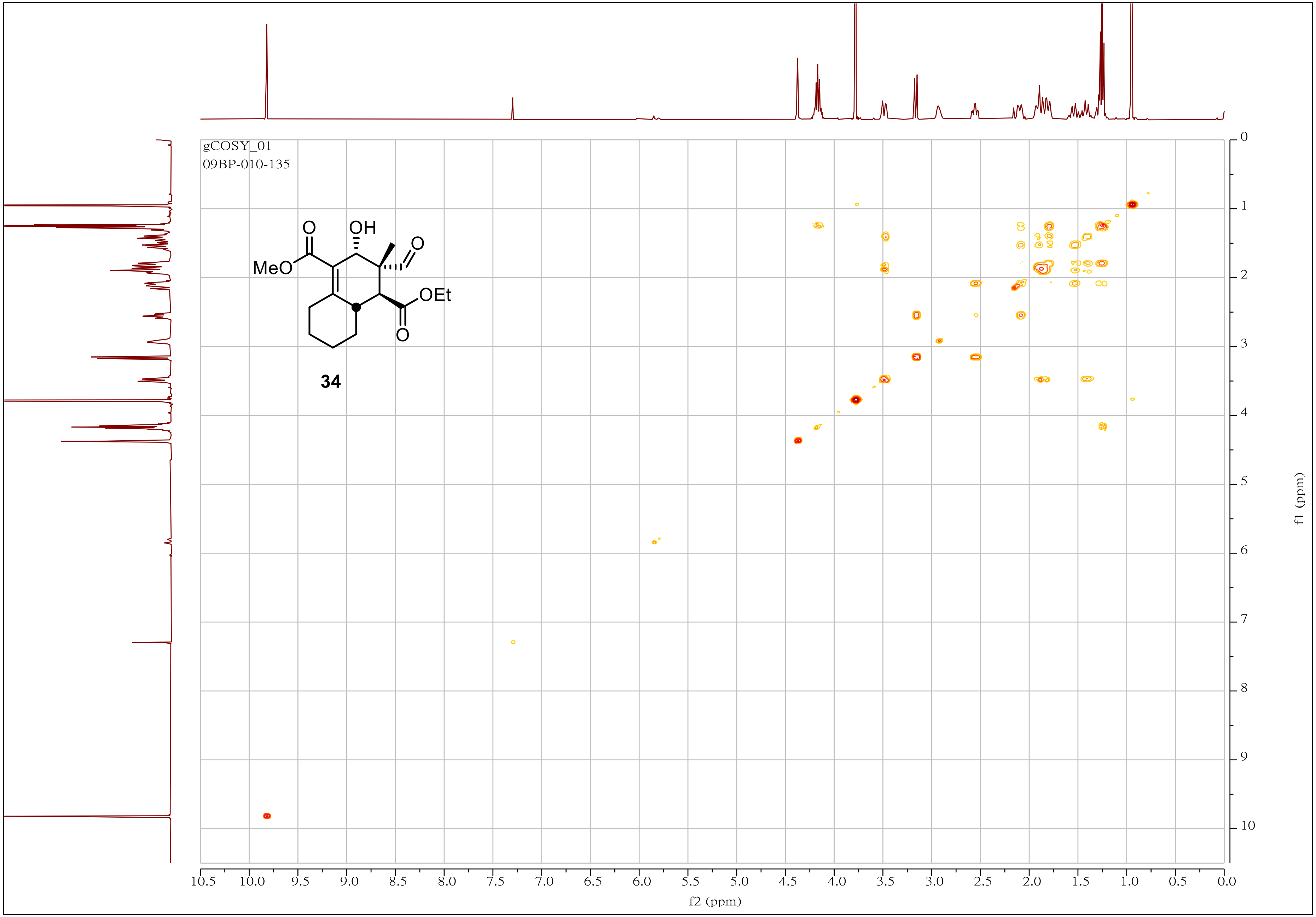


$^{1}$H-$^{1}$H COSY spectrum for compound **34**

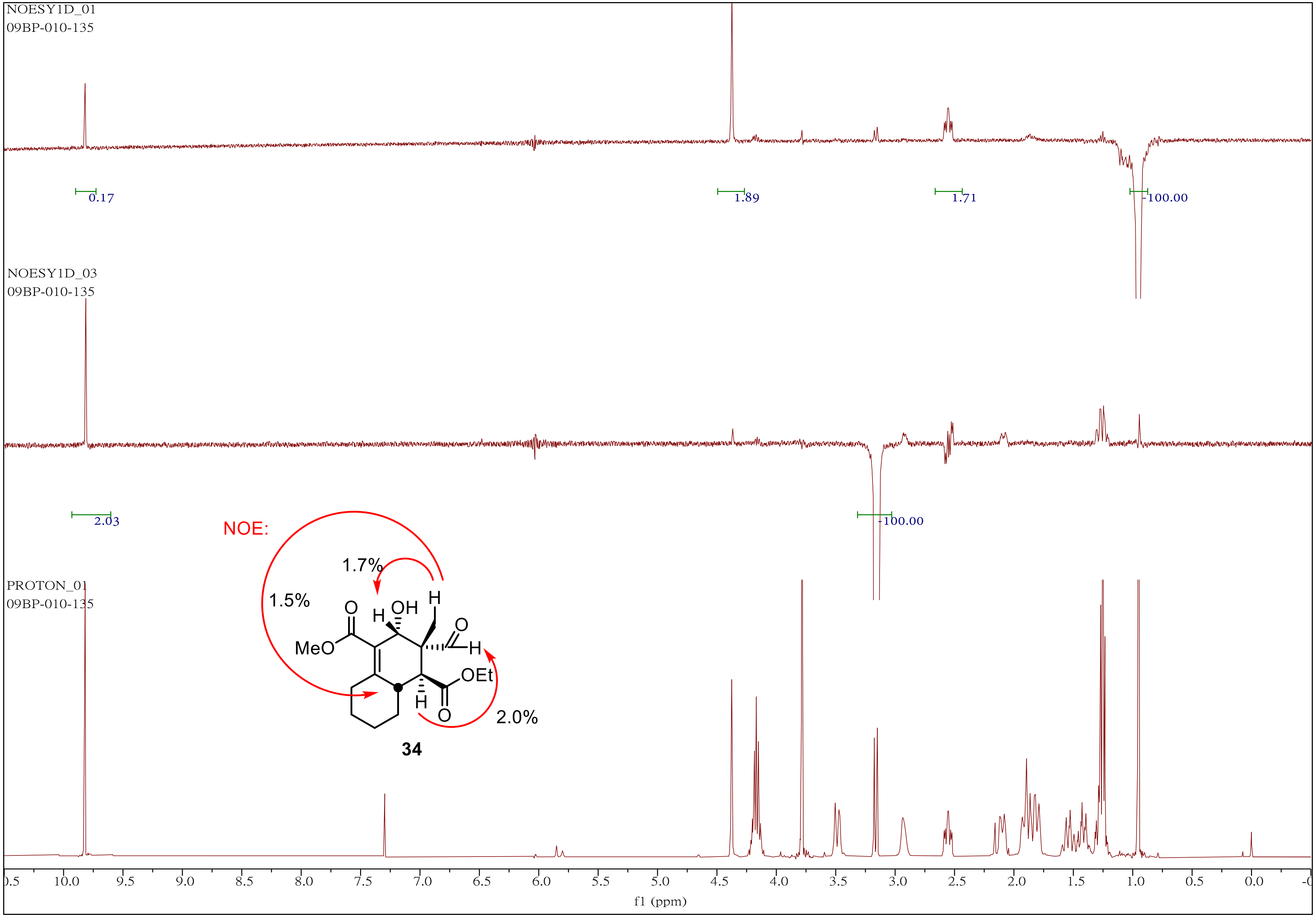


1D NOESY spectra for compound **34**

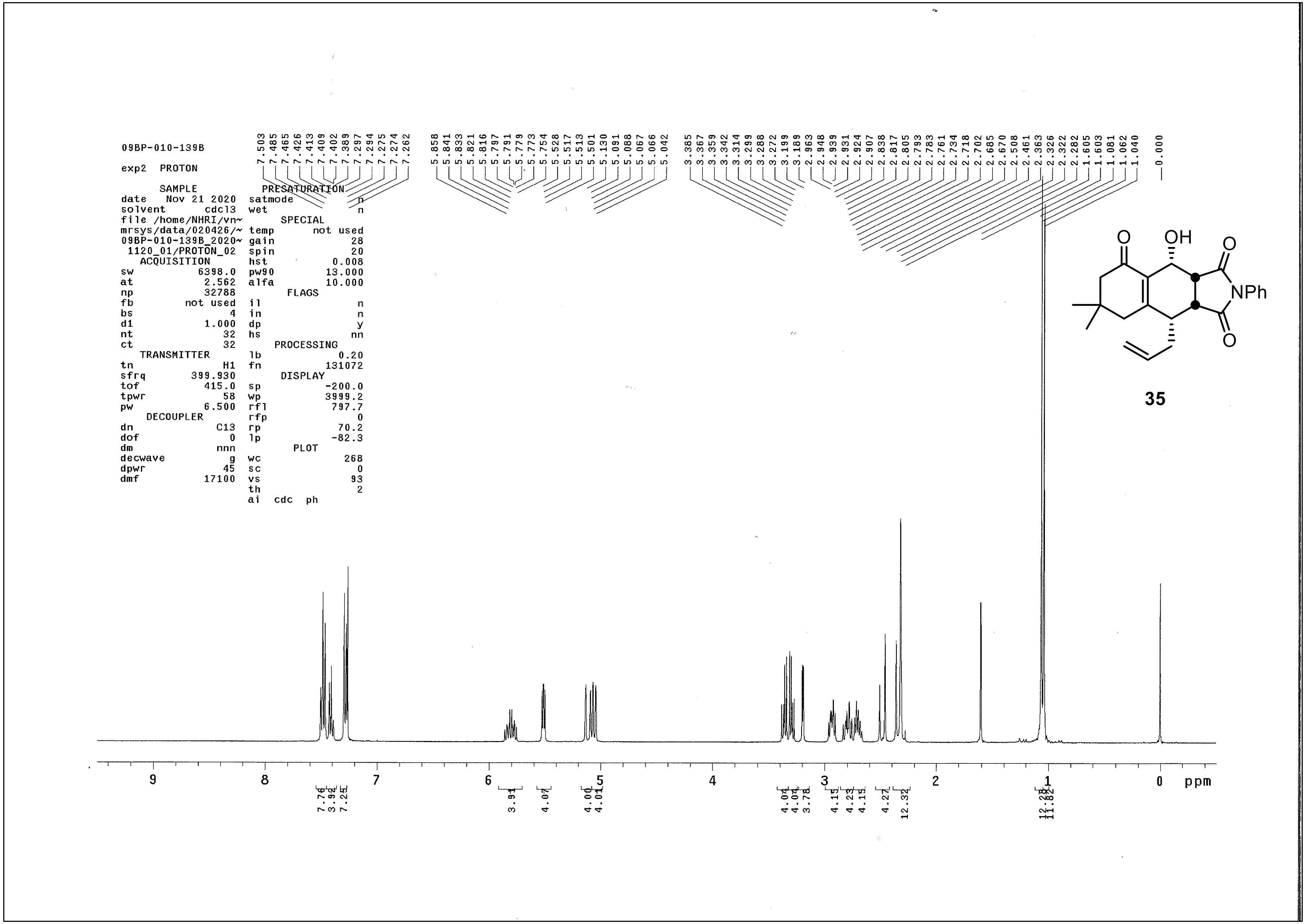

$^{1}$H NMR spectrum for compound **35**

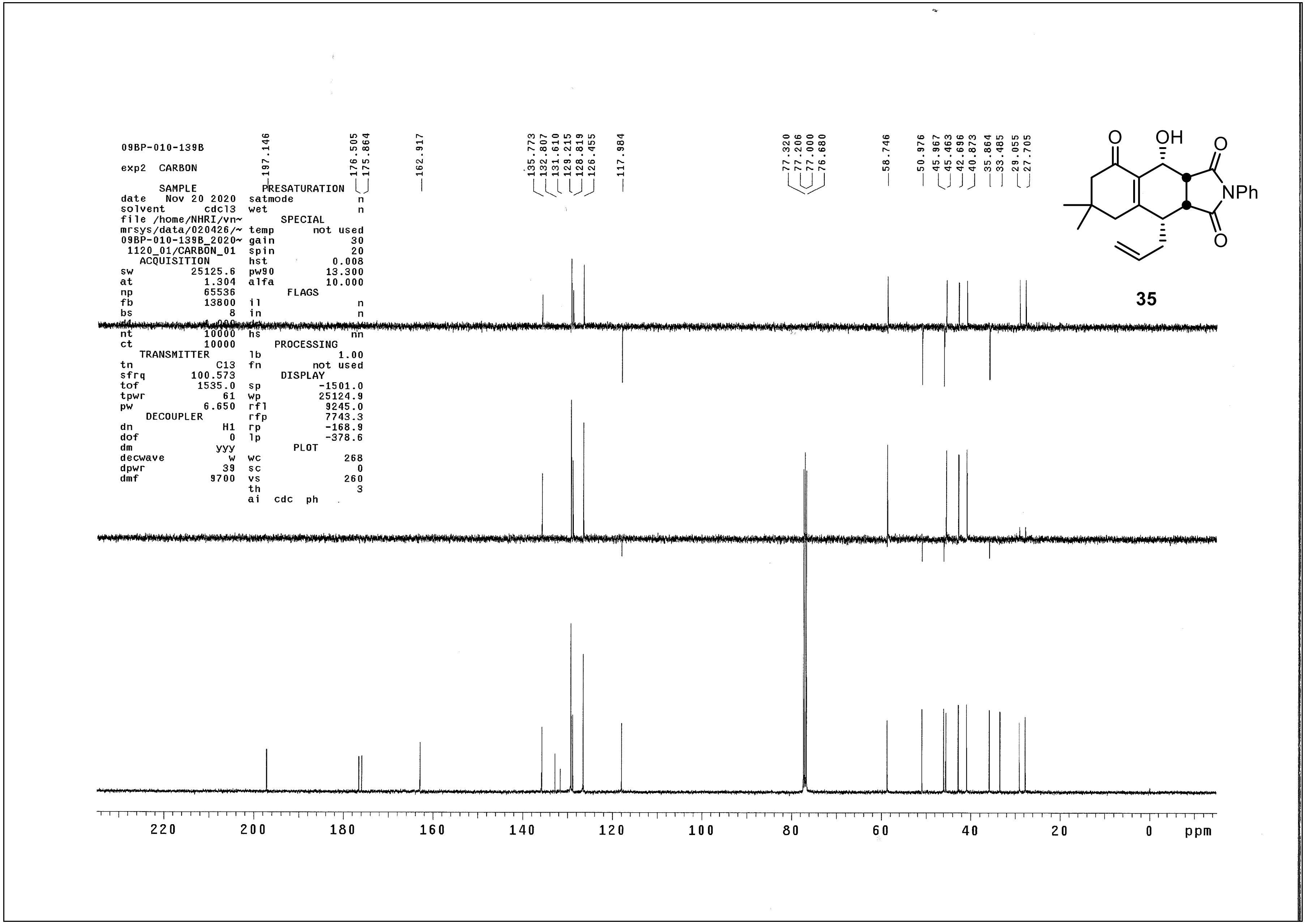


$^{13}$C NMR + DEPT spectra for compound **35**

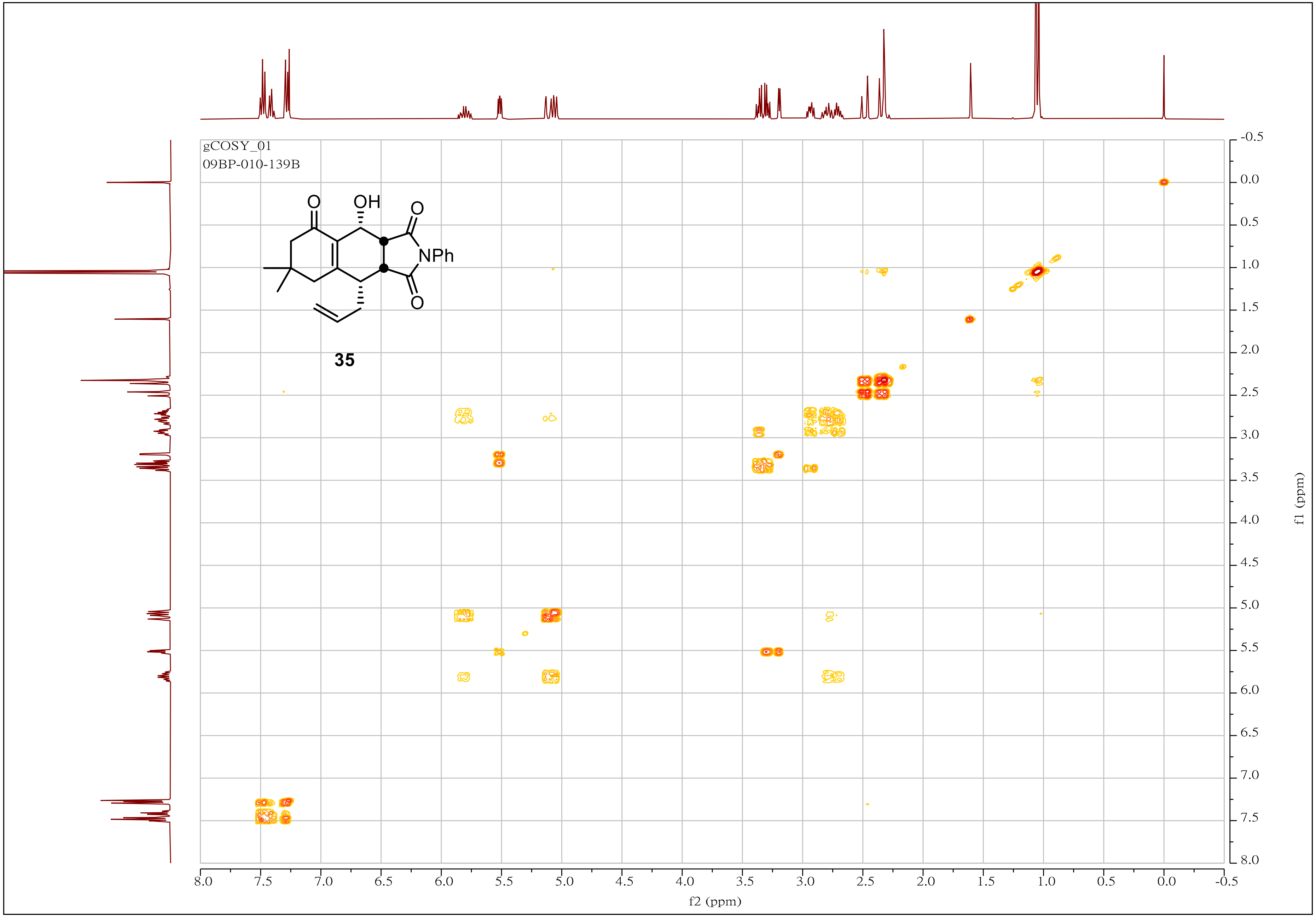


$^1$H-$^1$H COSY spectrum for compound **35**

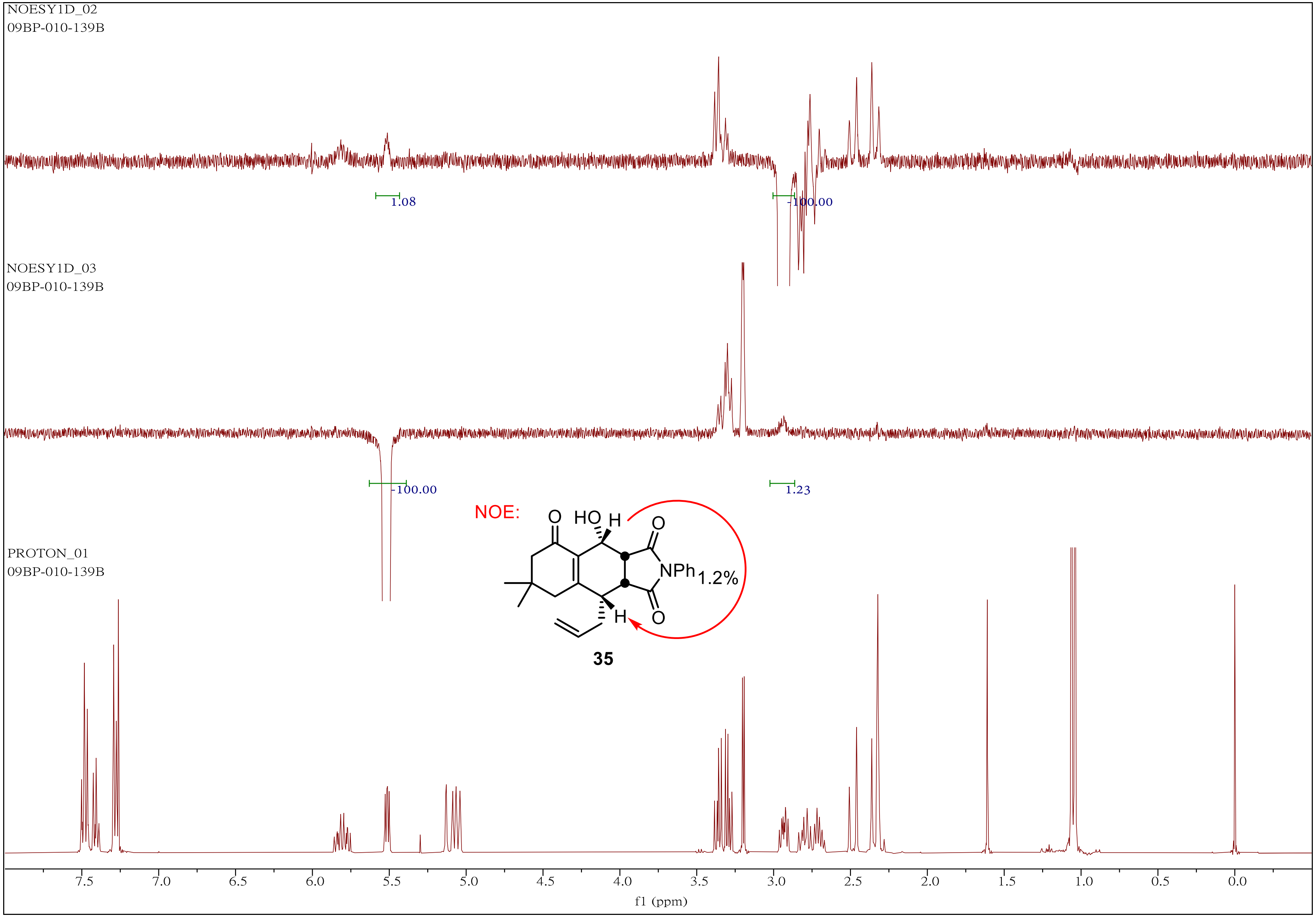


1D NOESY spectra for compound **35**

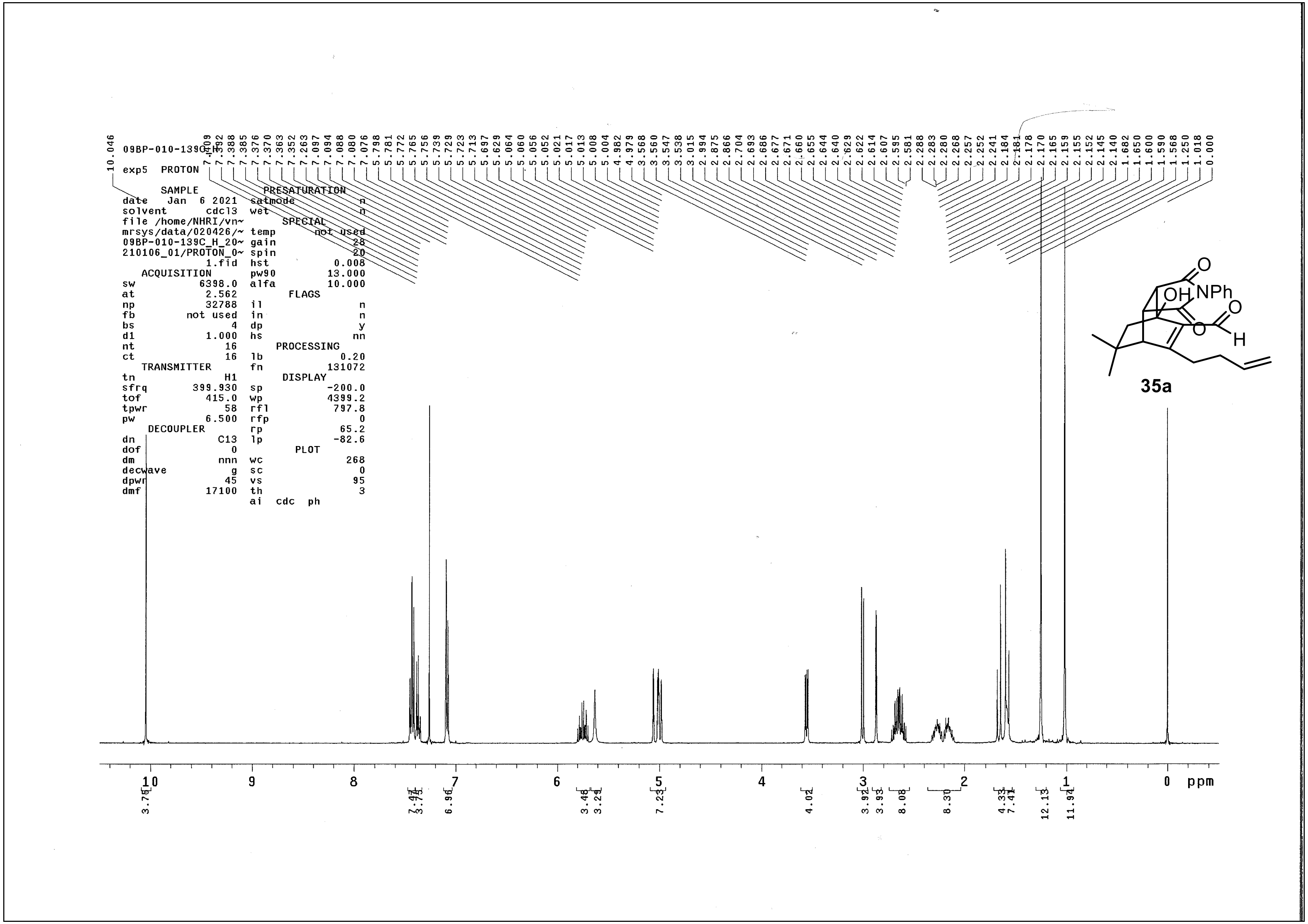

$^{1}$H NMR spectrum for compound **35a**

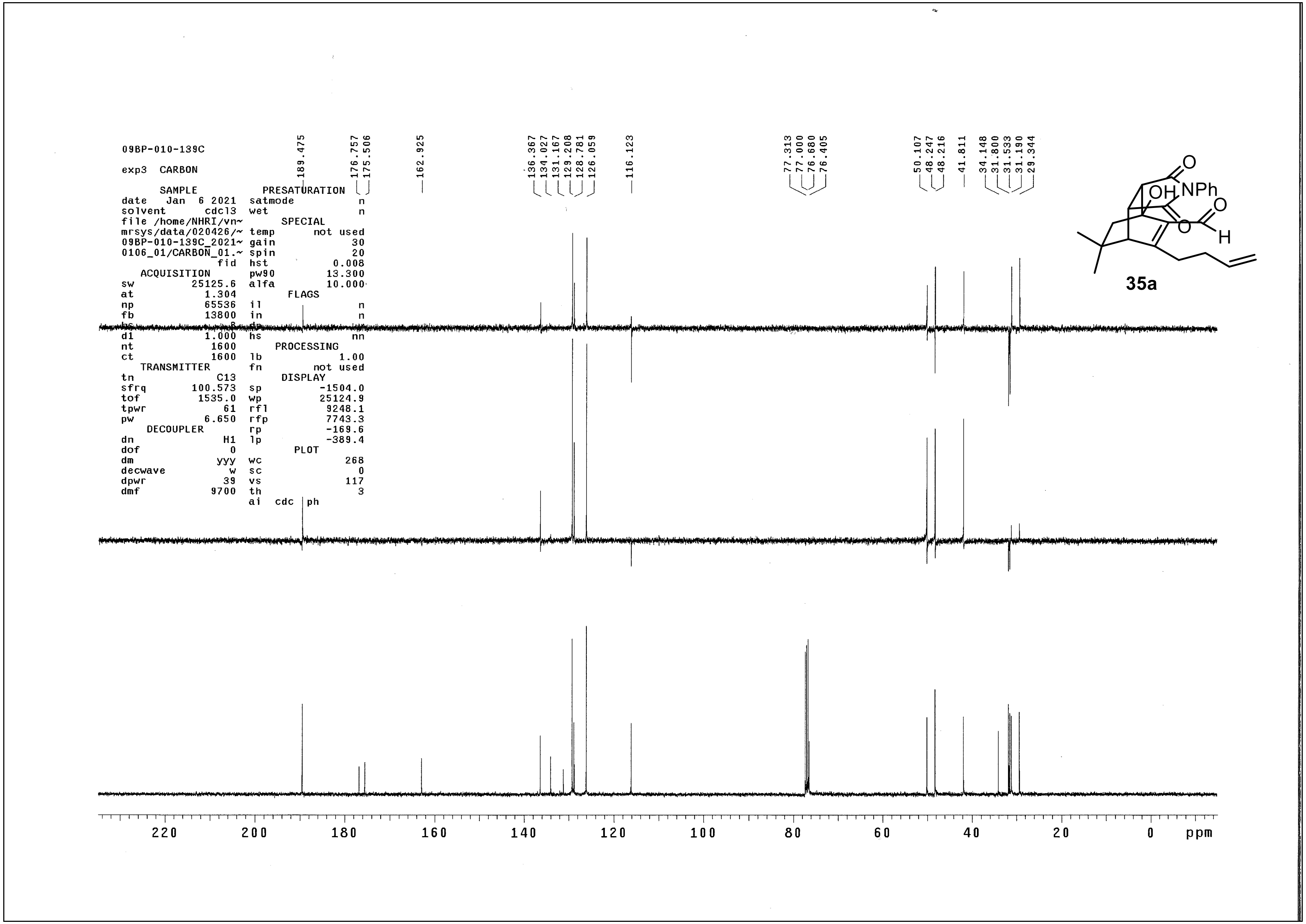


$^{13}$C NMR + DEPT spectra for compound **35a**

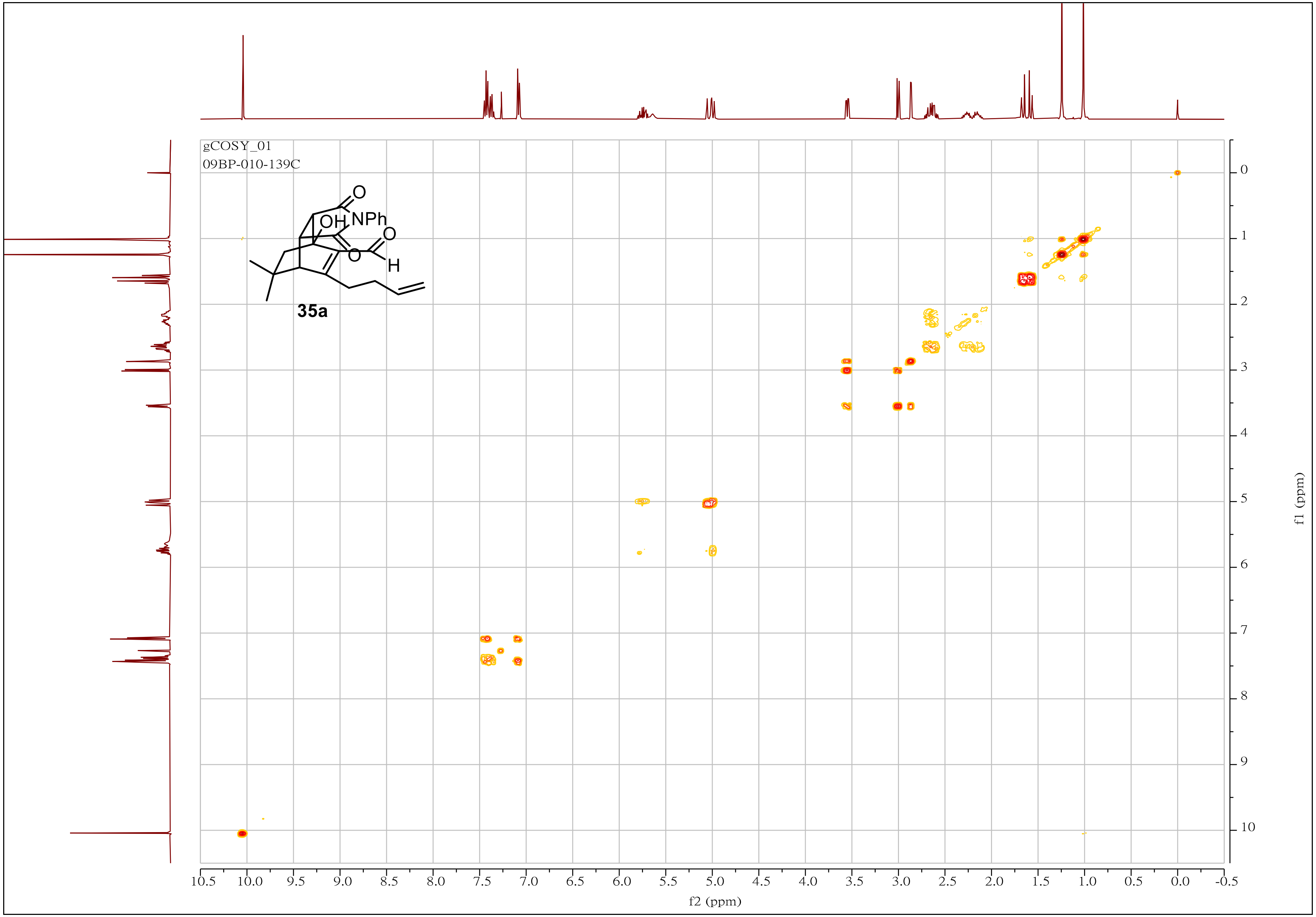


$^{1}$H-$^{1}$H COSY spectrum for compound **35a**

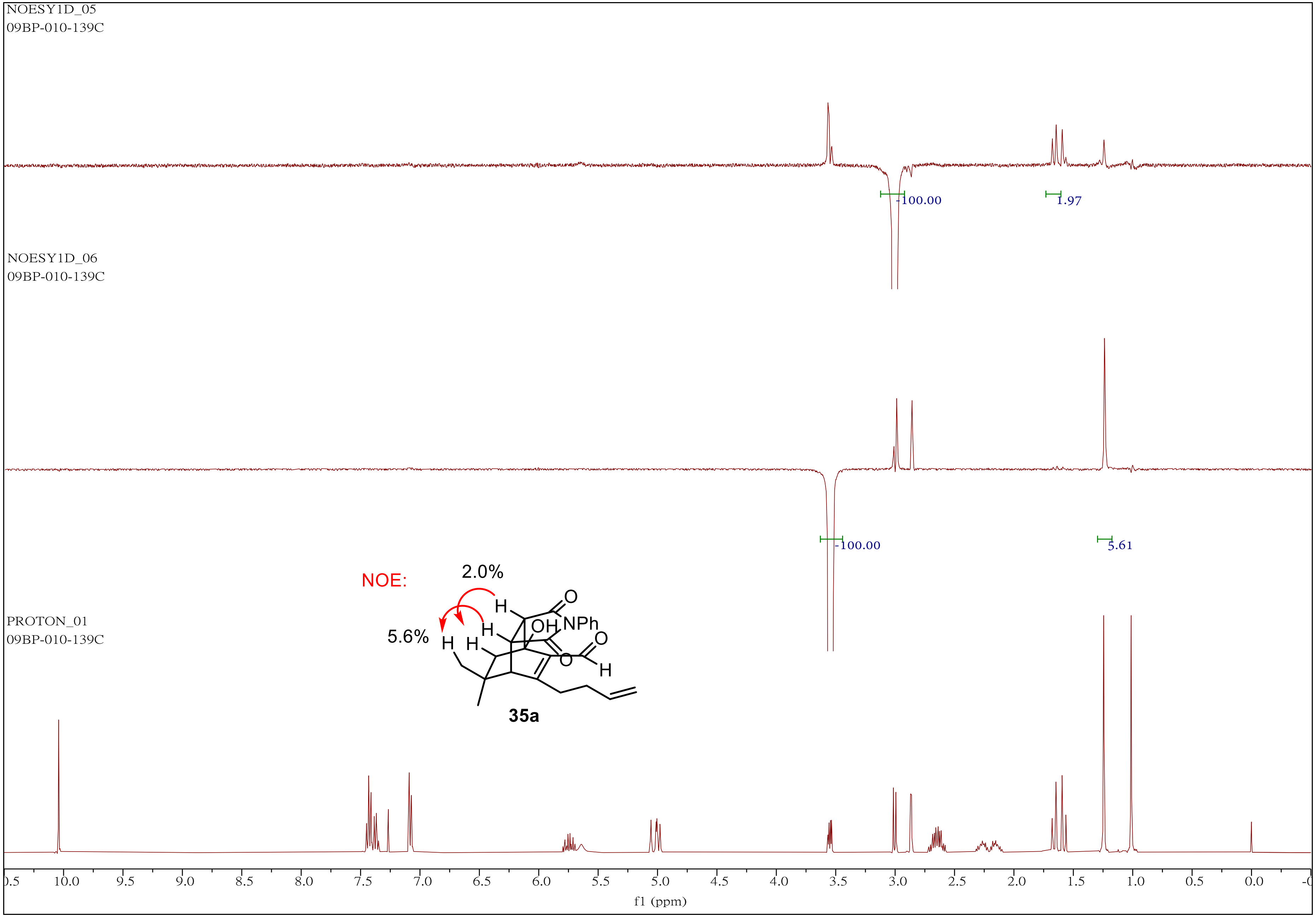


1D NOESY spectra for compound **35a**

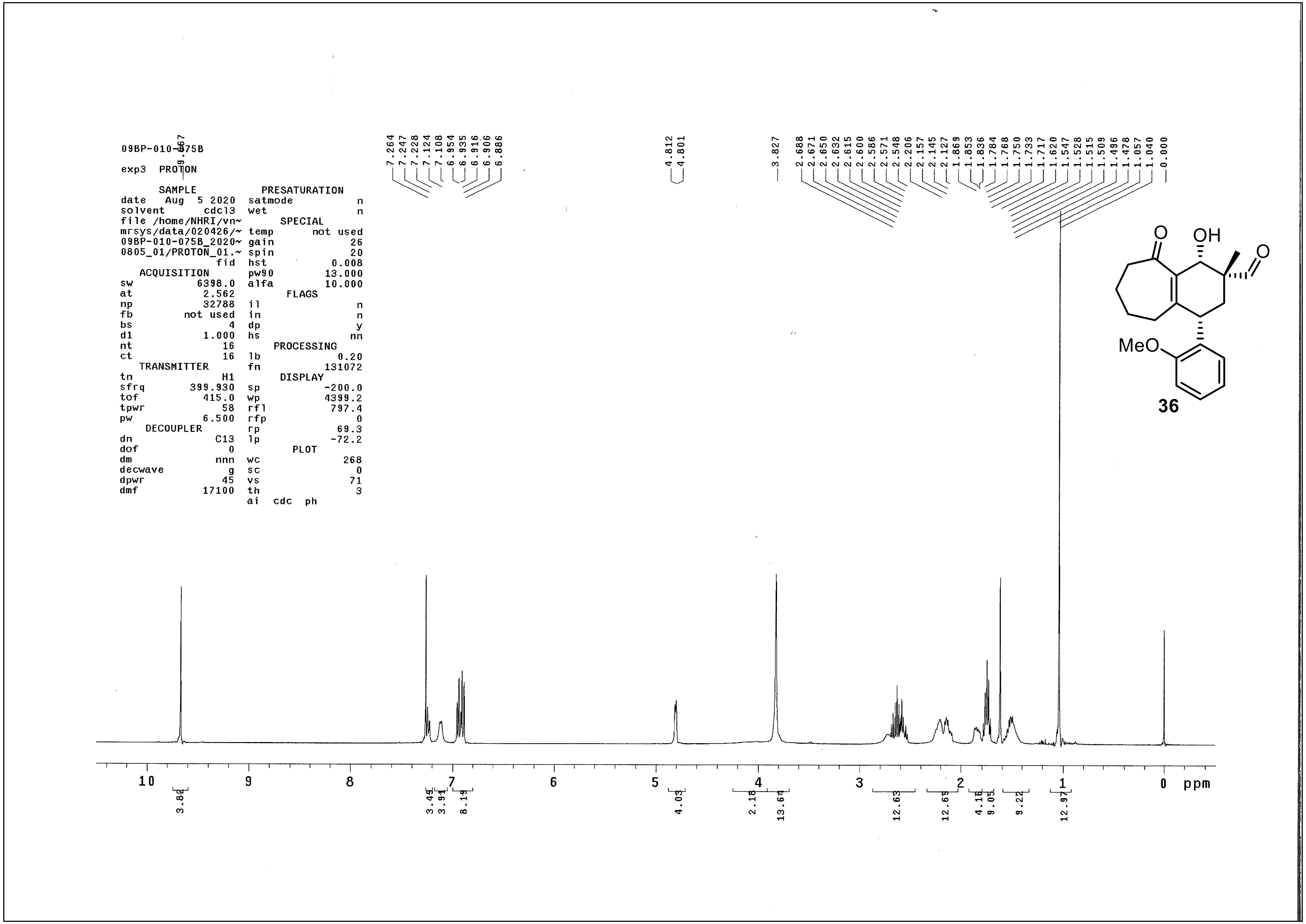


$^{1}$H NMR spectrum for compound **36**

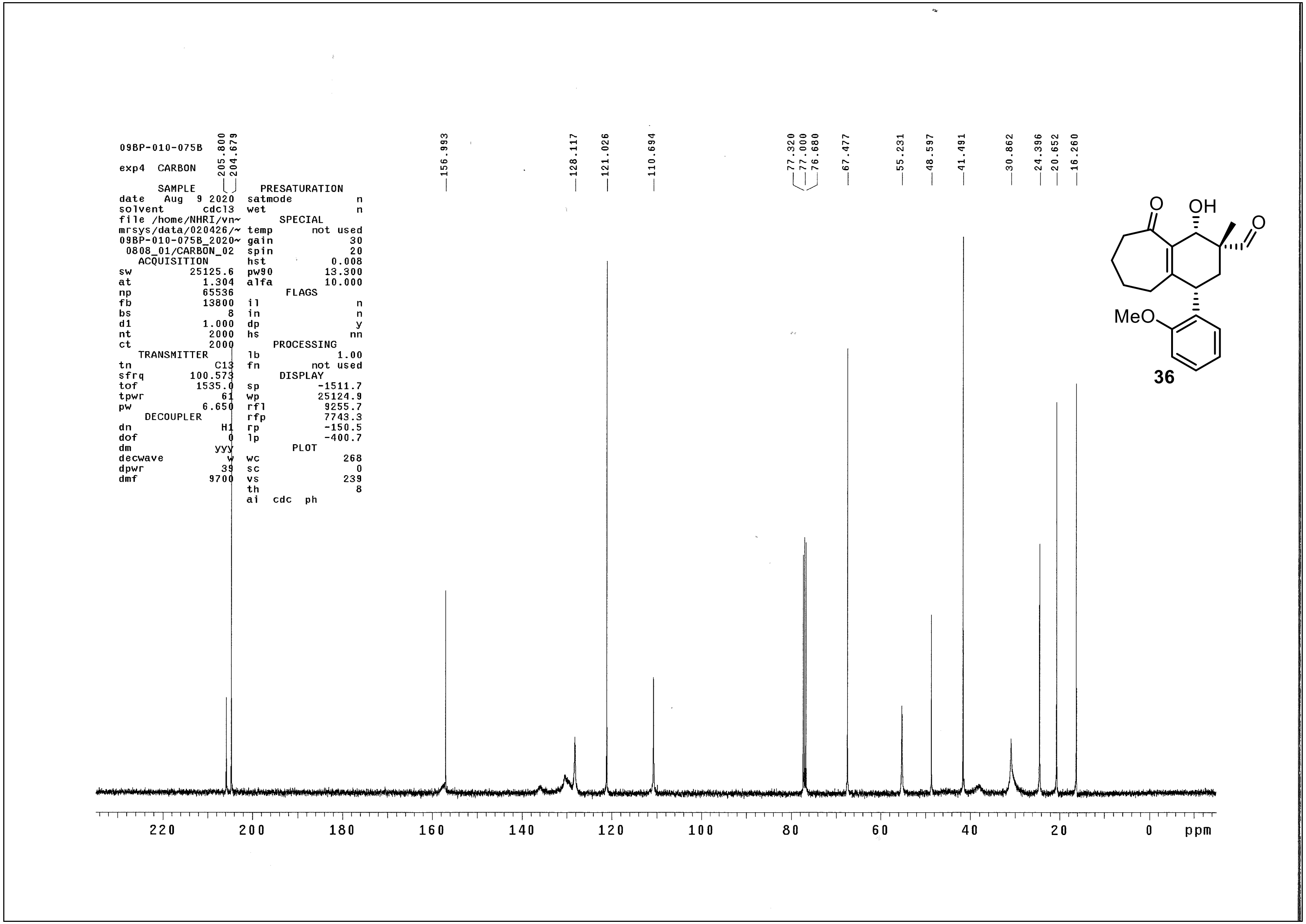


$^{13}$C NMR spectrum for compound **36**

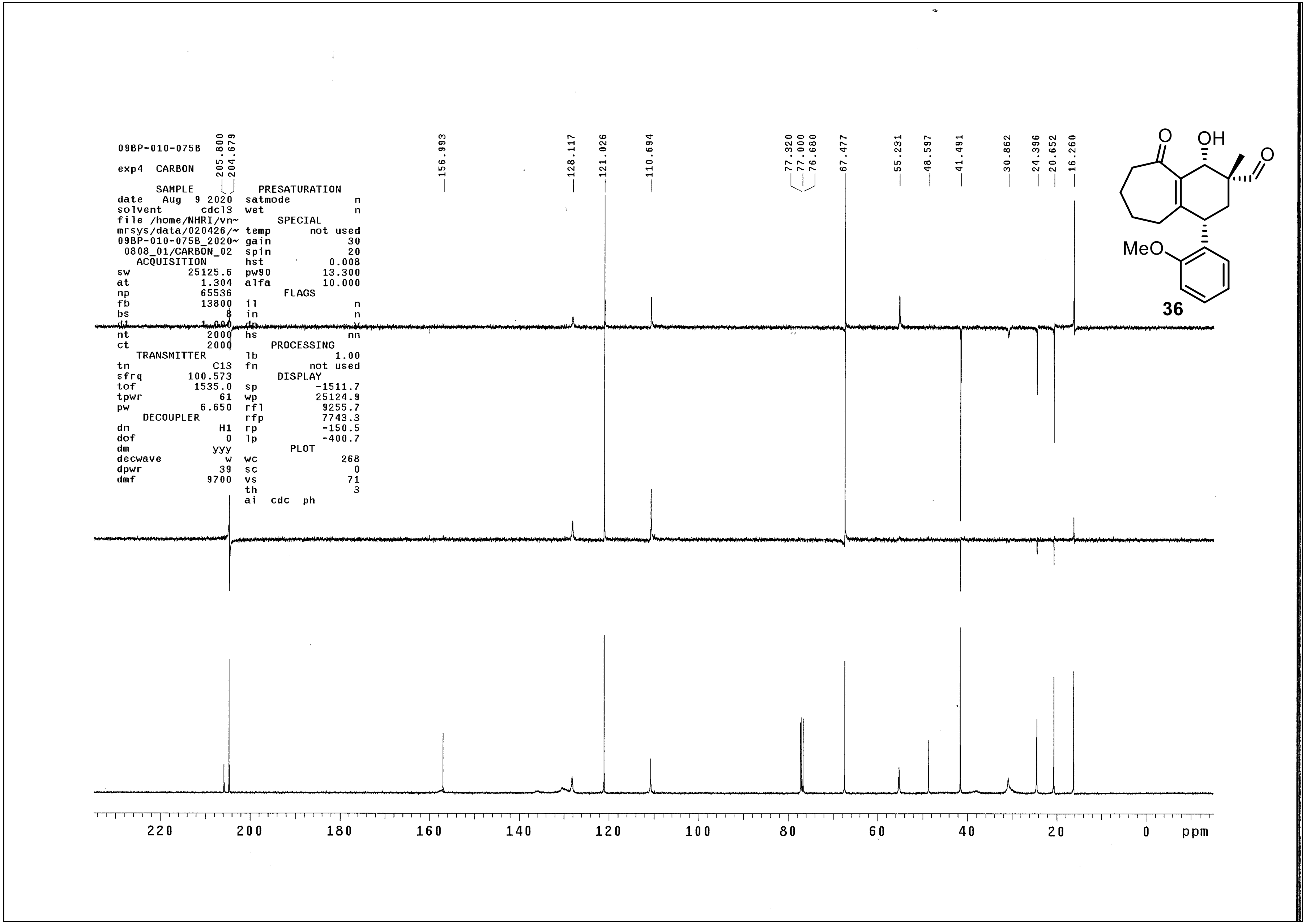


$^{13}C$ NMR + DEPT spectra for compound **36**

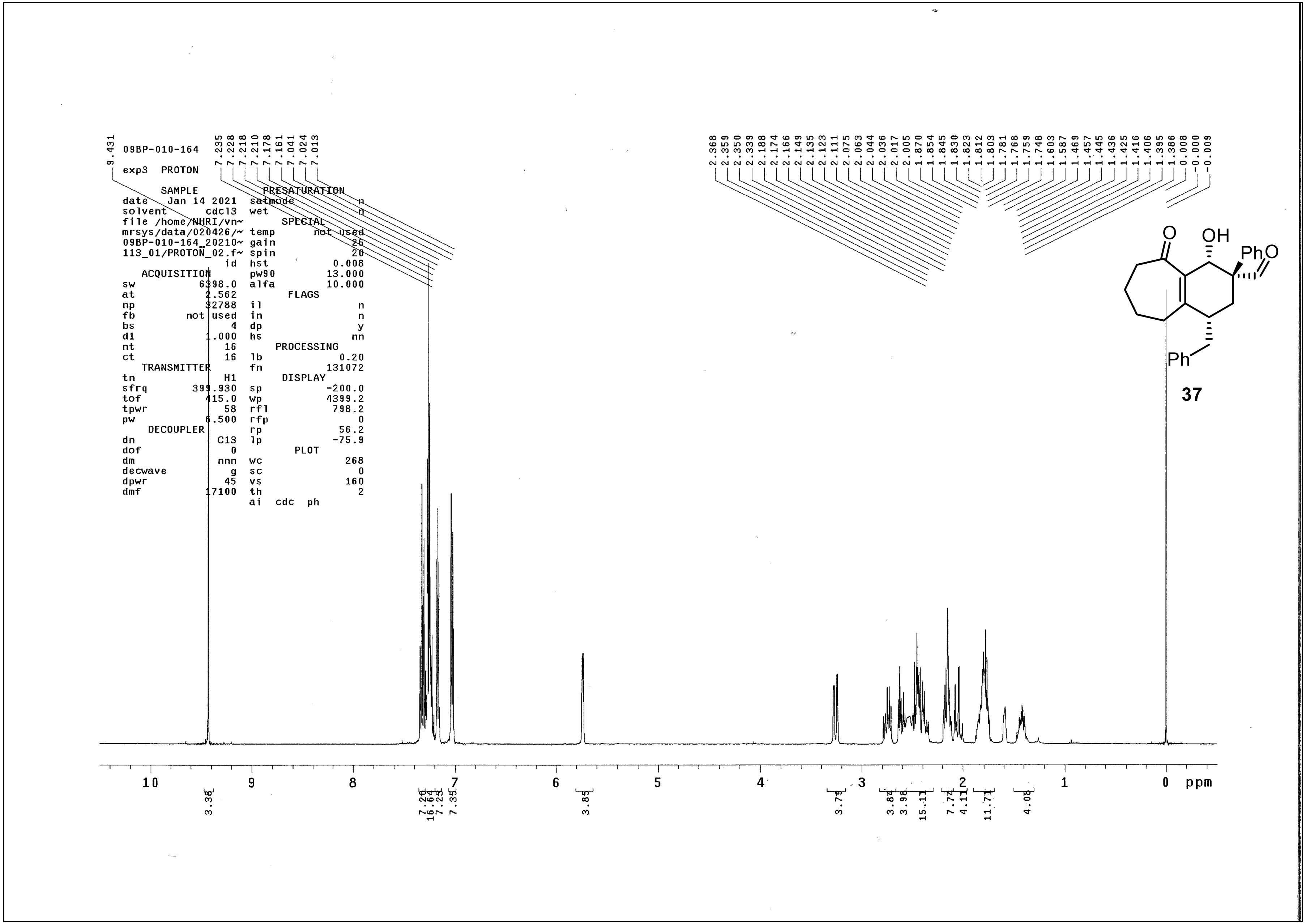

$^{1}$H NMR spectrum for compound **37**

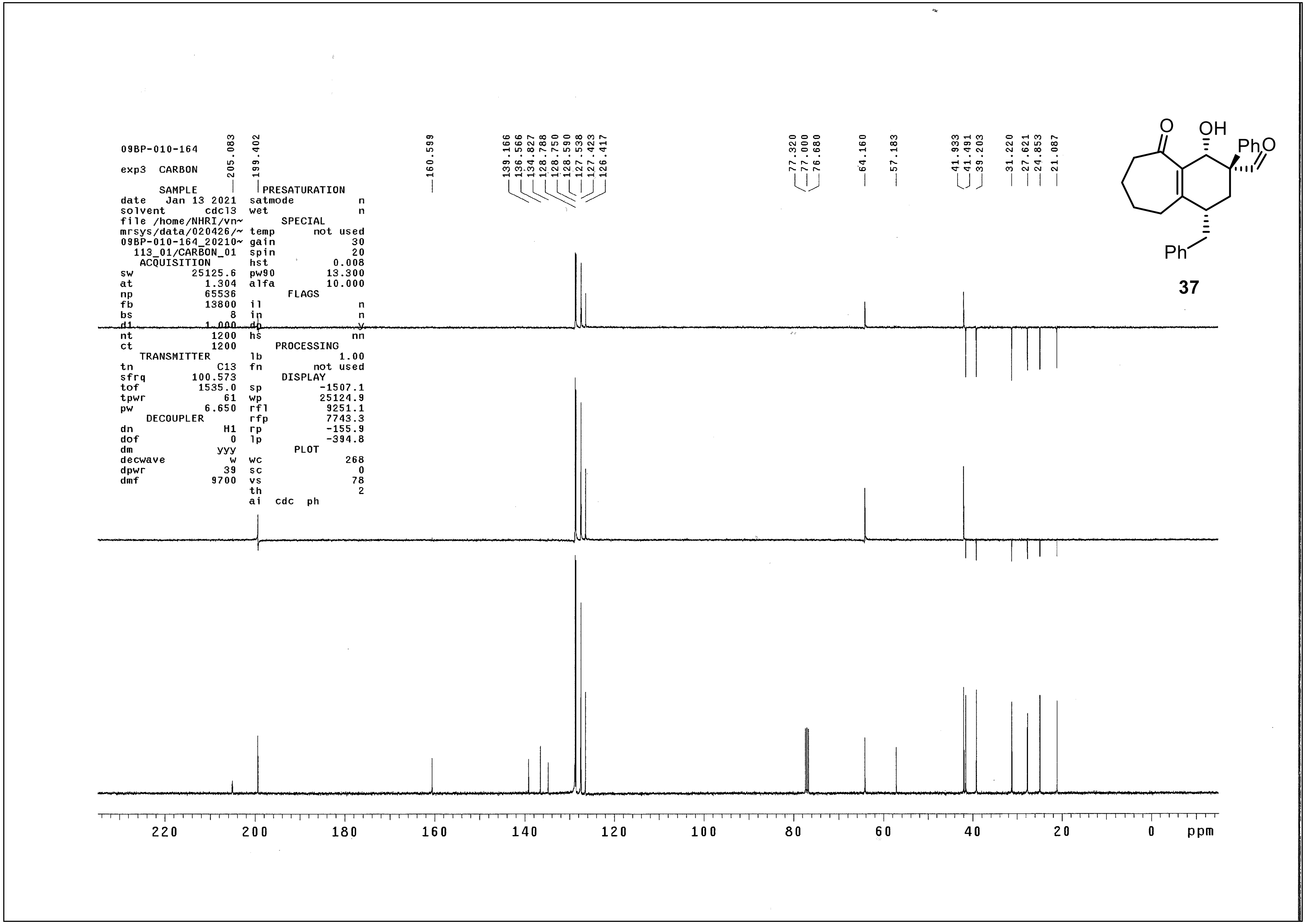

$^{13}$C NMR + DEPT spectra for compound **37**

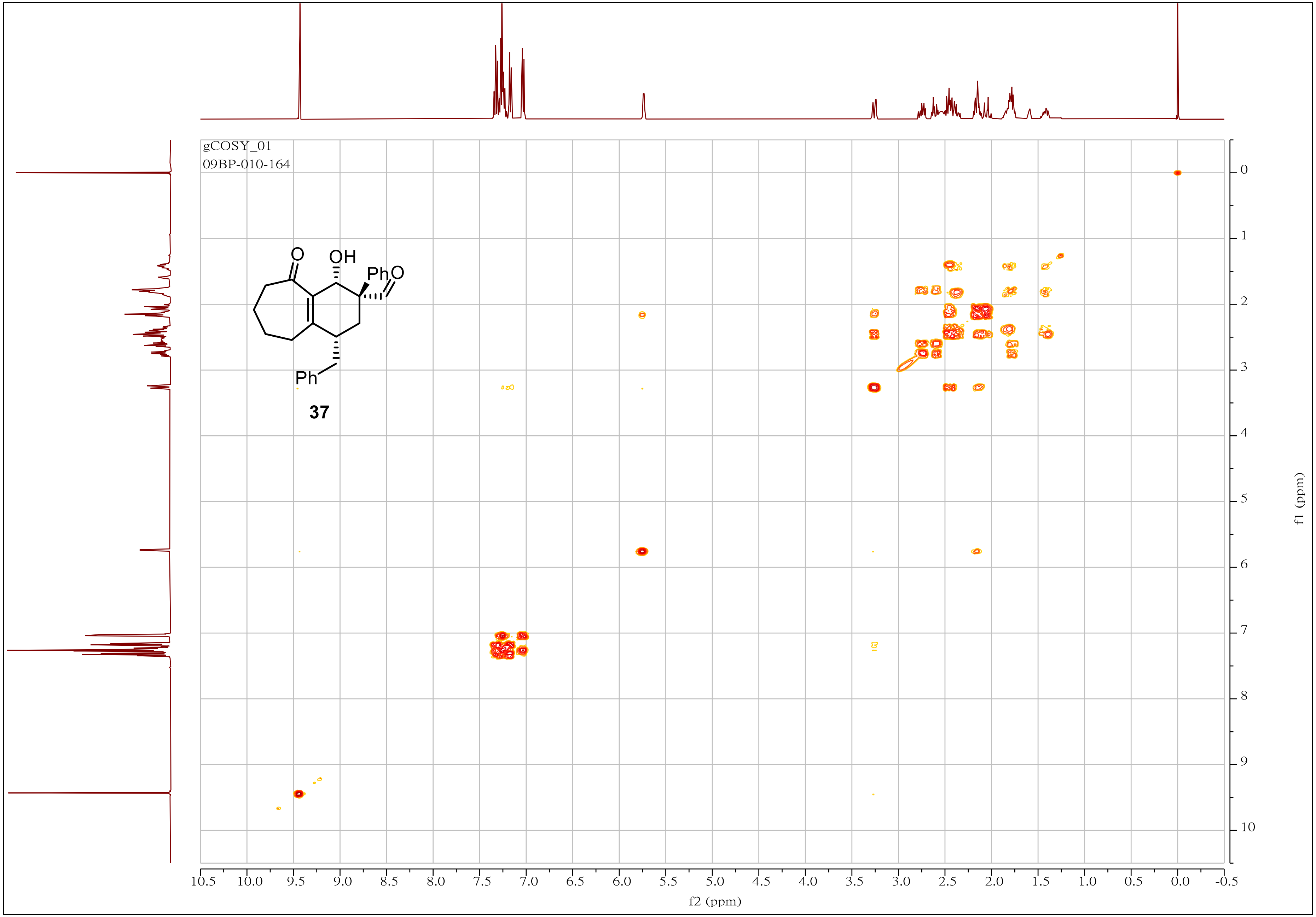


$^{1}$H-$^{1}$H COSY spectrum for compound **37**

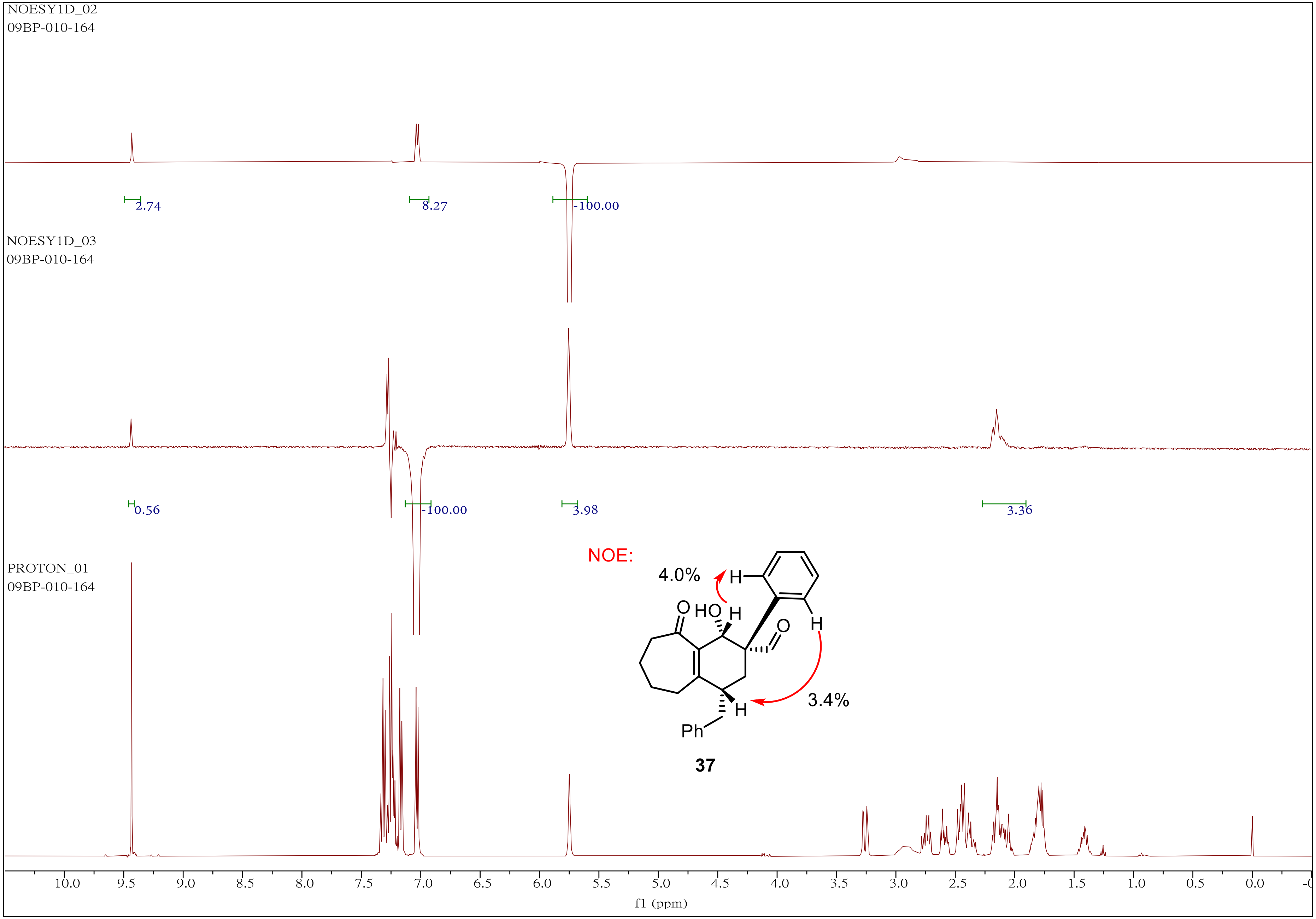


1D NOESY spectra for compound **37**

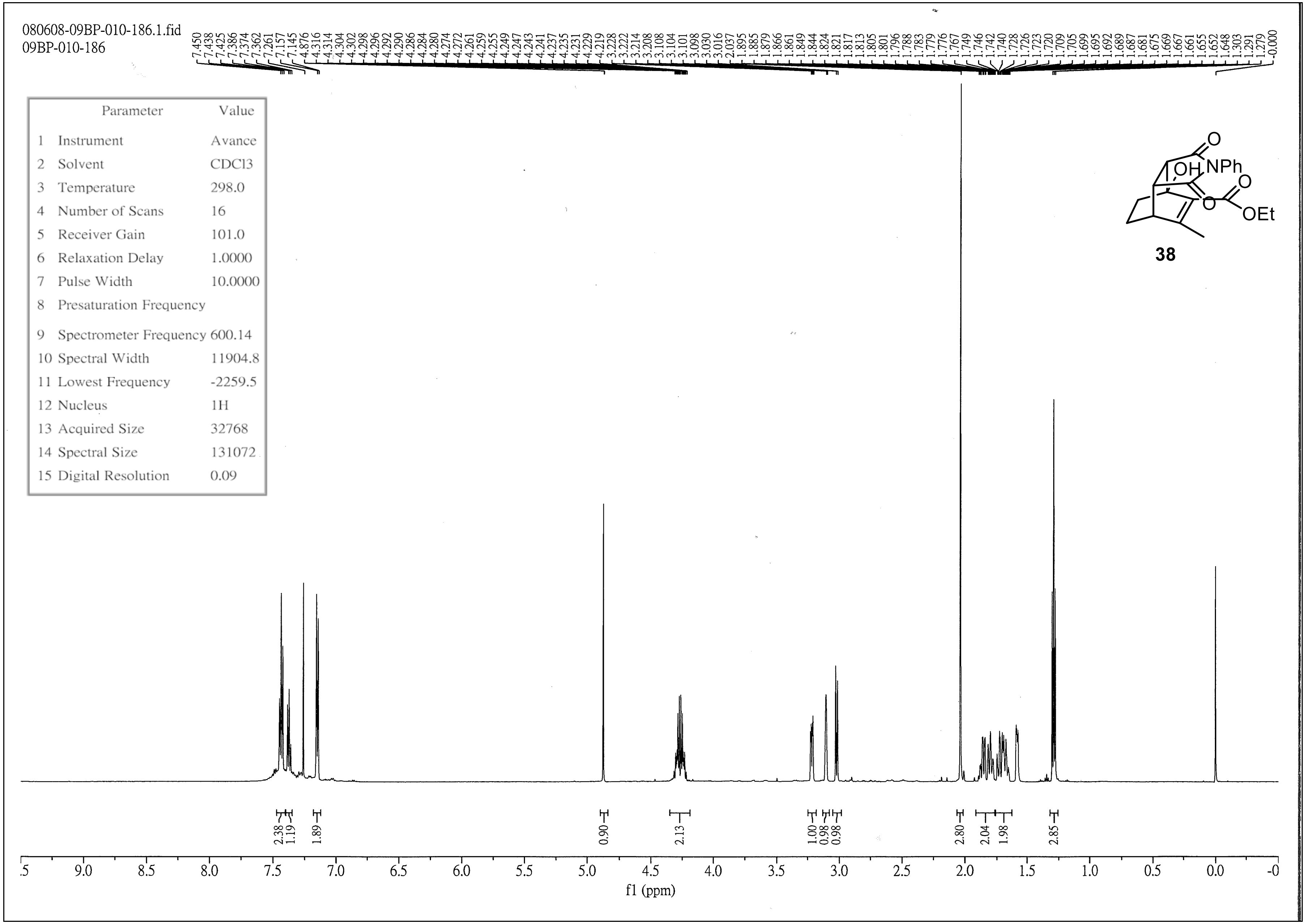


$^1$H NMR spectrum for compound **38**

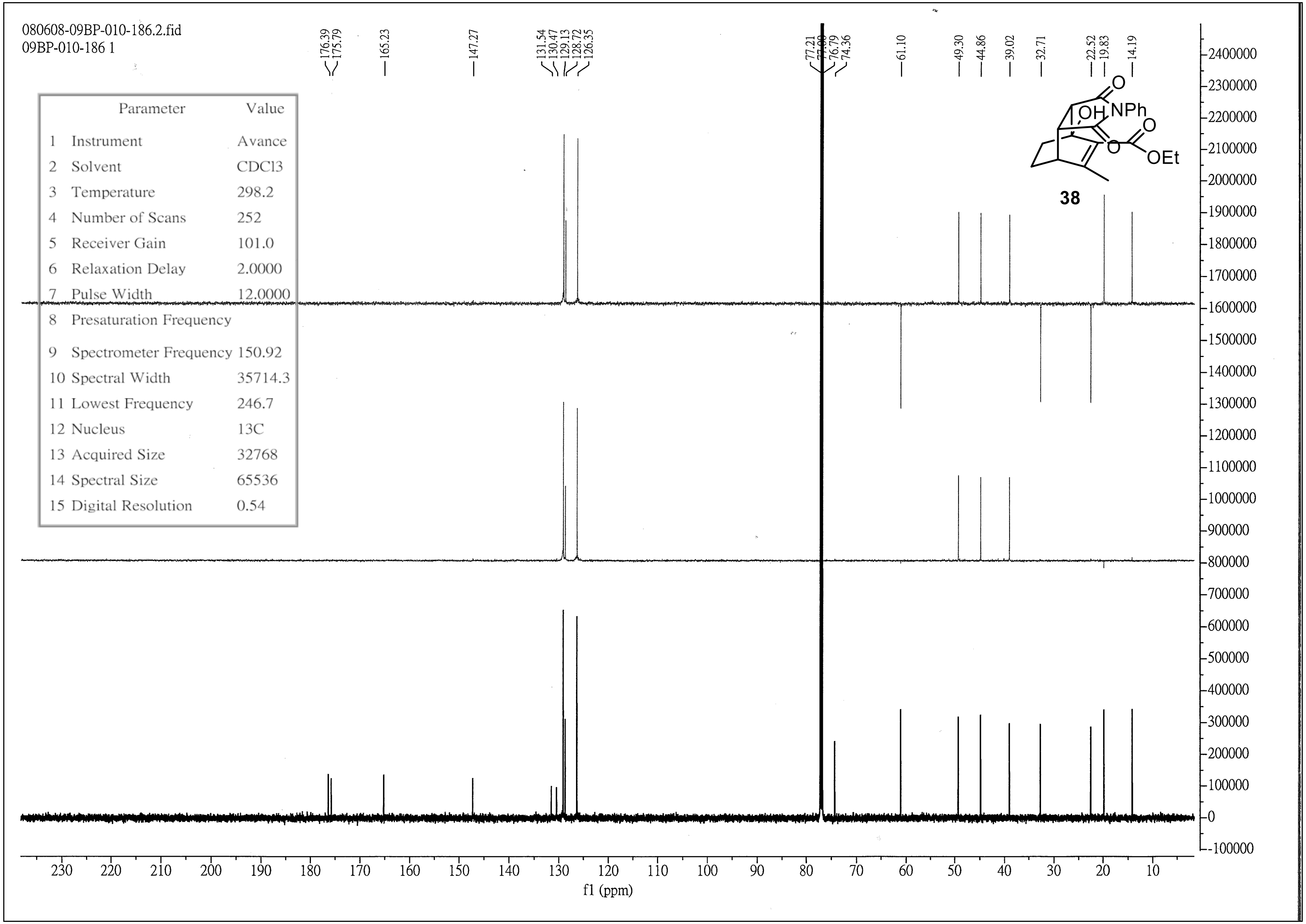


$^{13}$C NMR + DEPT spectra for compound **38**

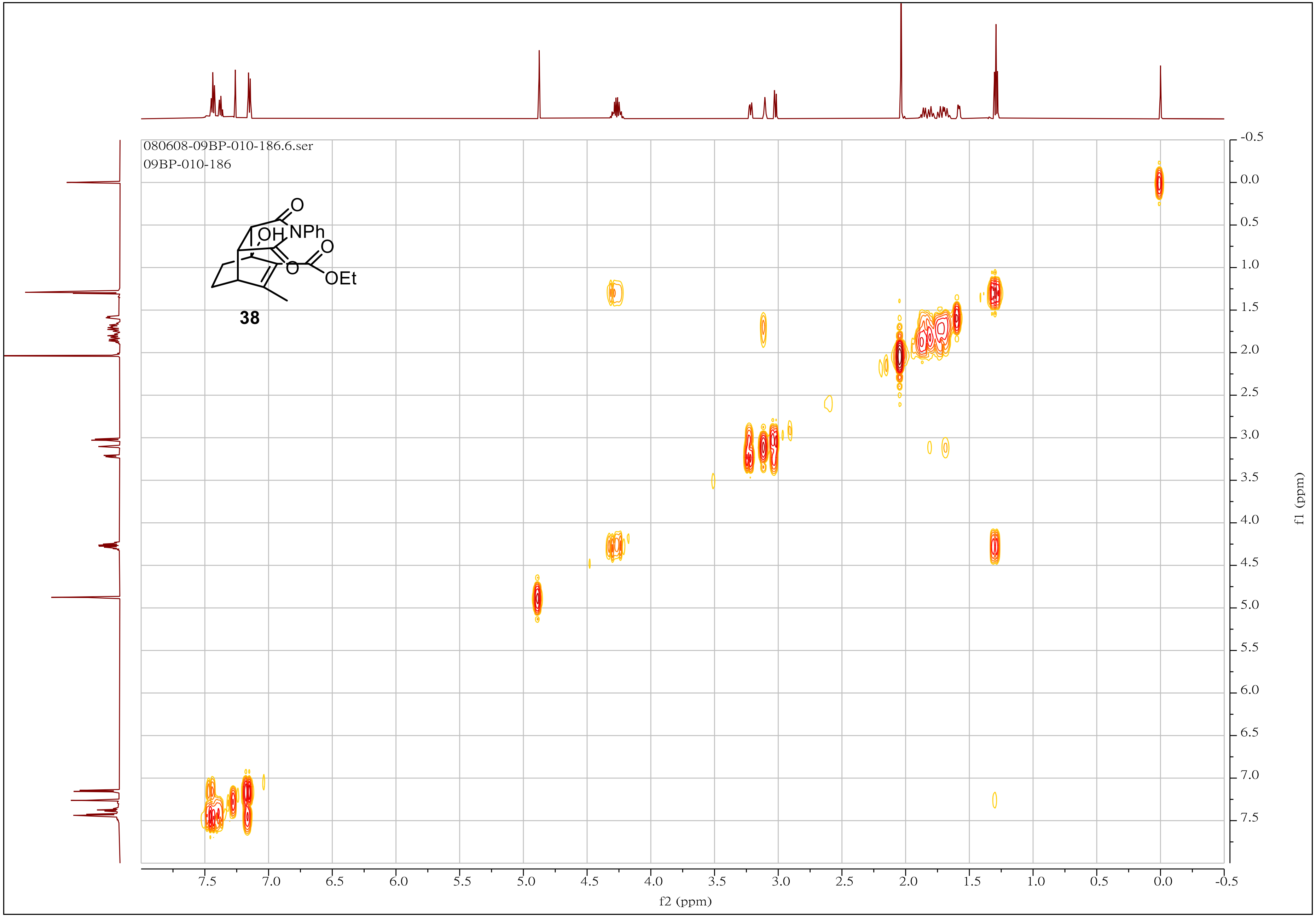


$^1$H-$^1$H COSY spectrum for compound **38**

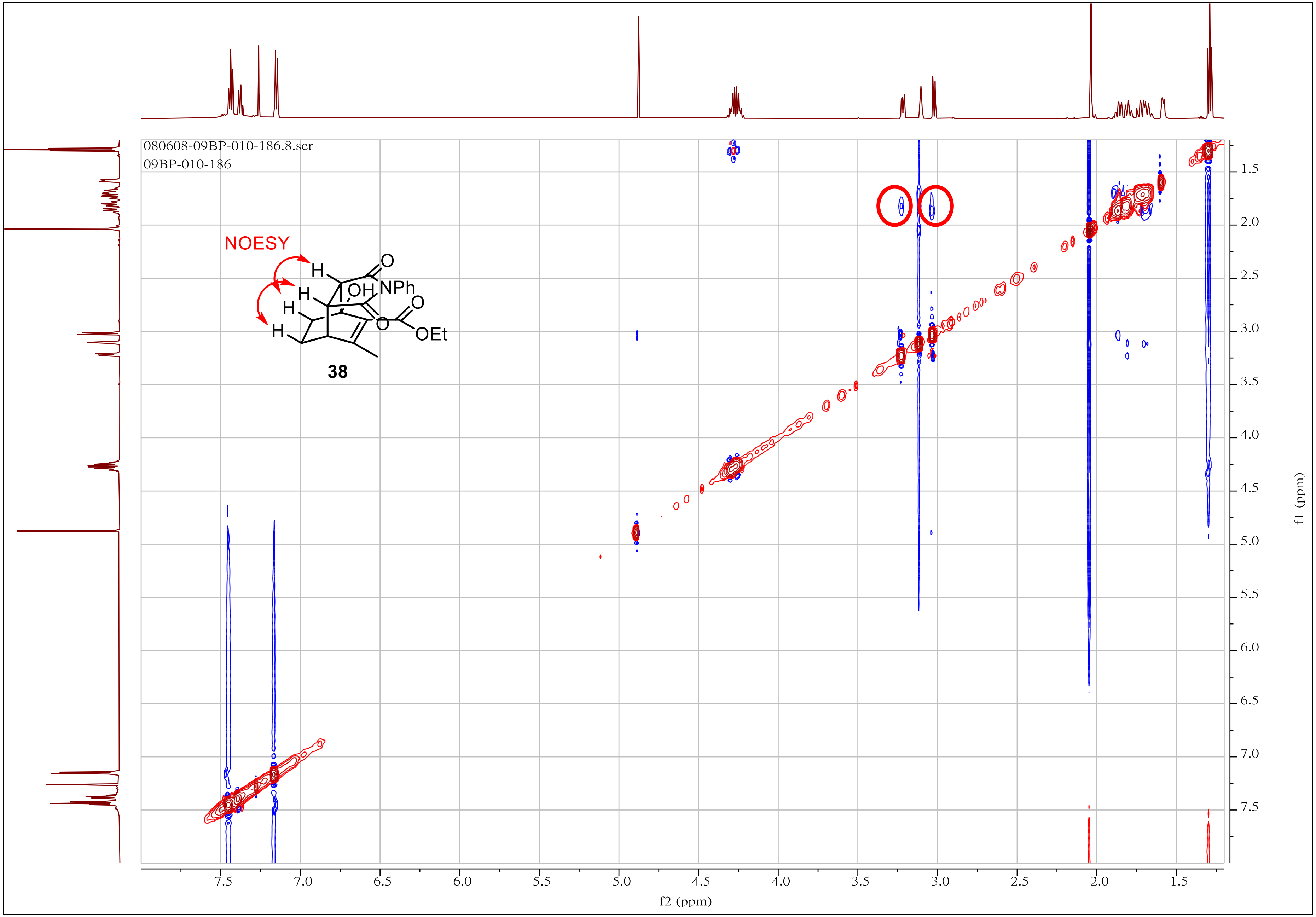


2D NOESY spectrum for compound **38**

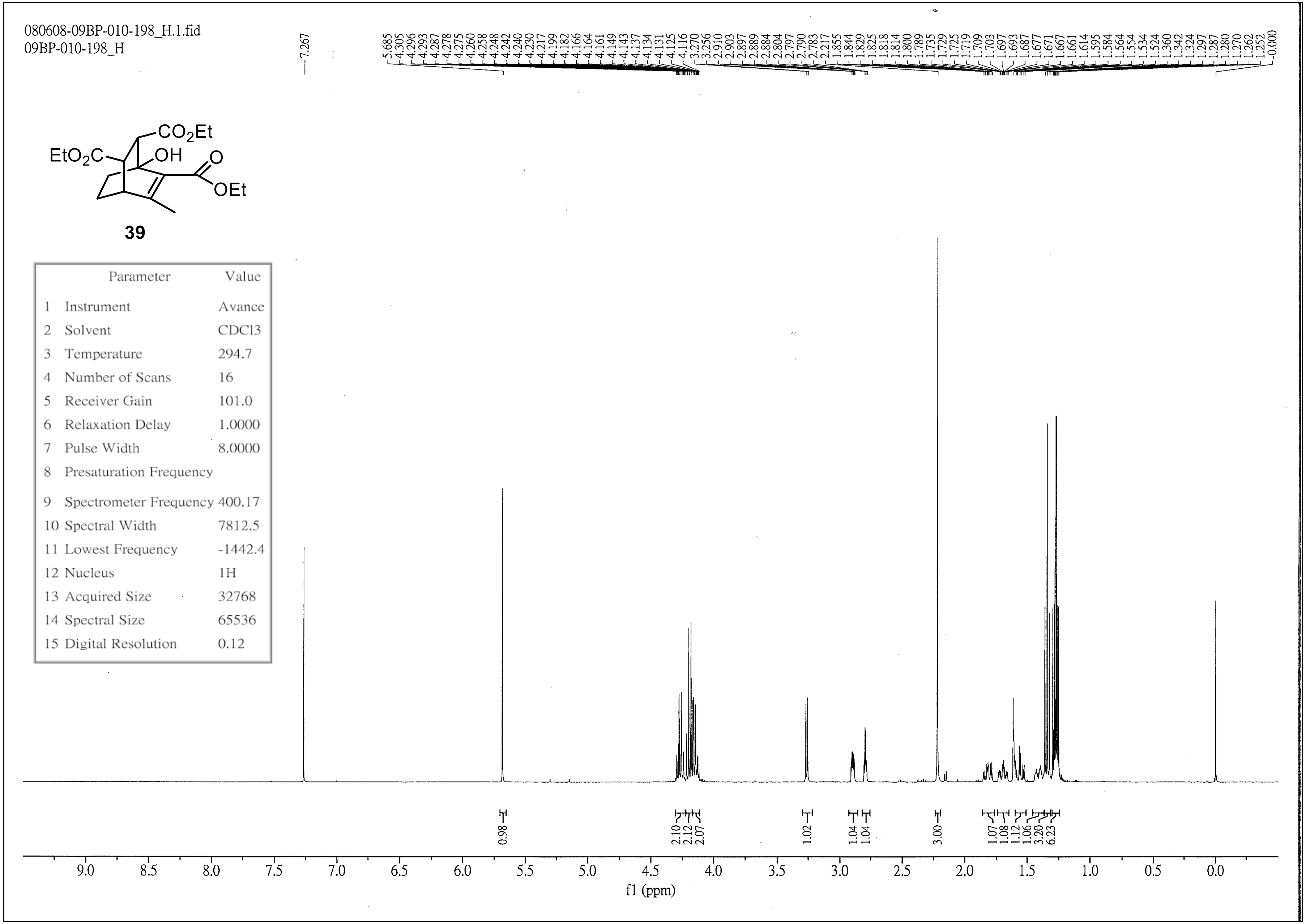


$^1$H NMR spectrum for compound **39**

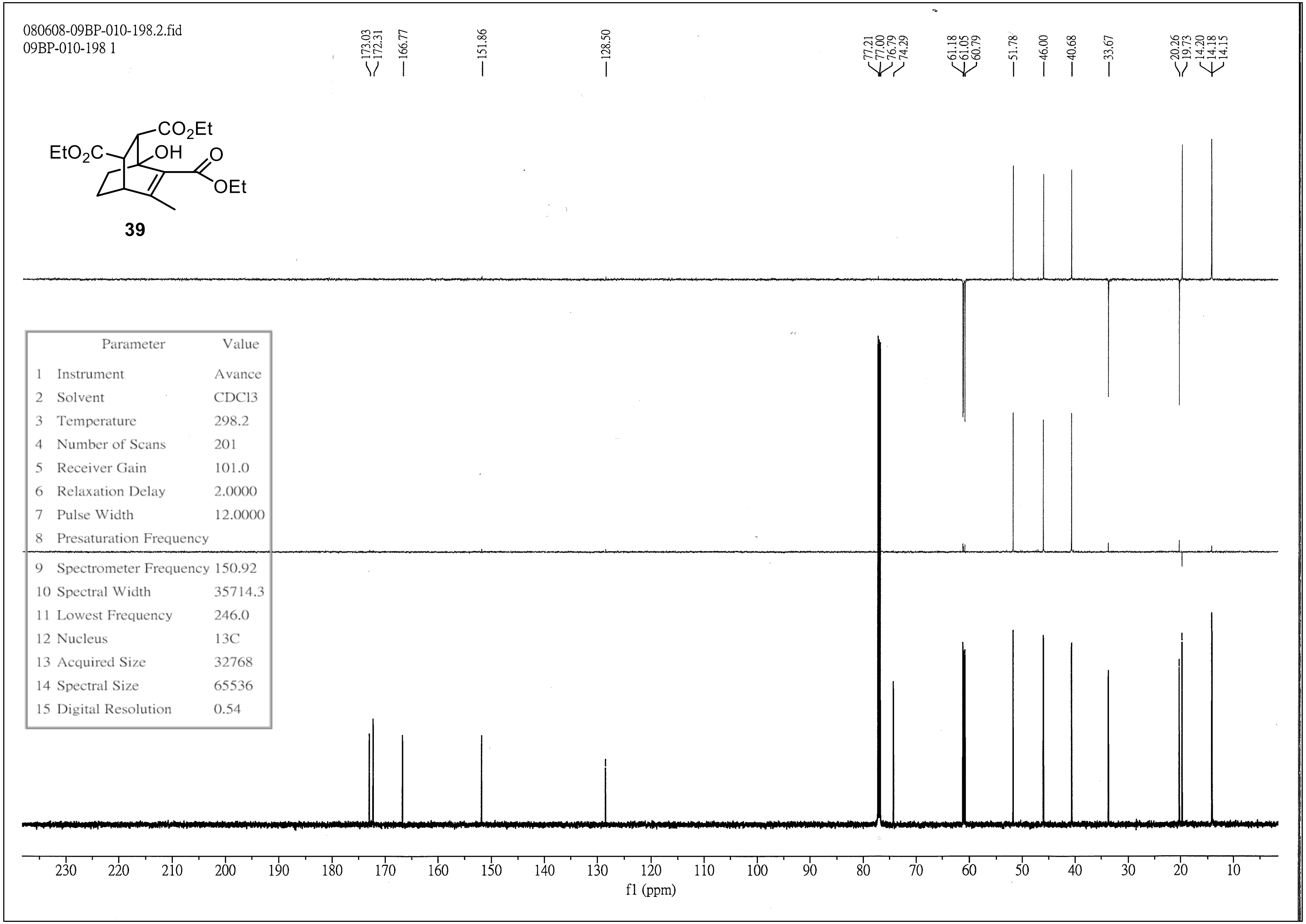


$^{13}$C NMR + DEPT spectra for compound **39**

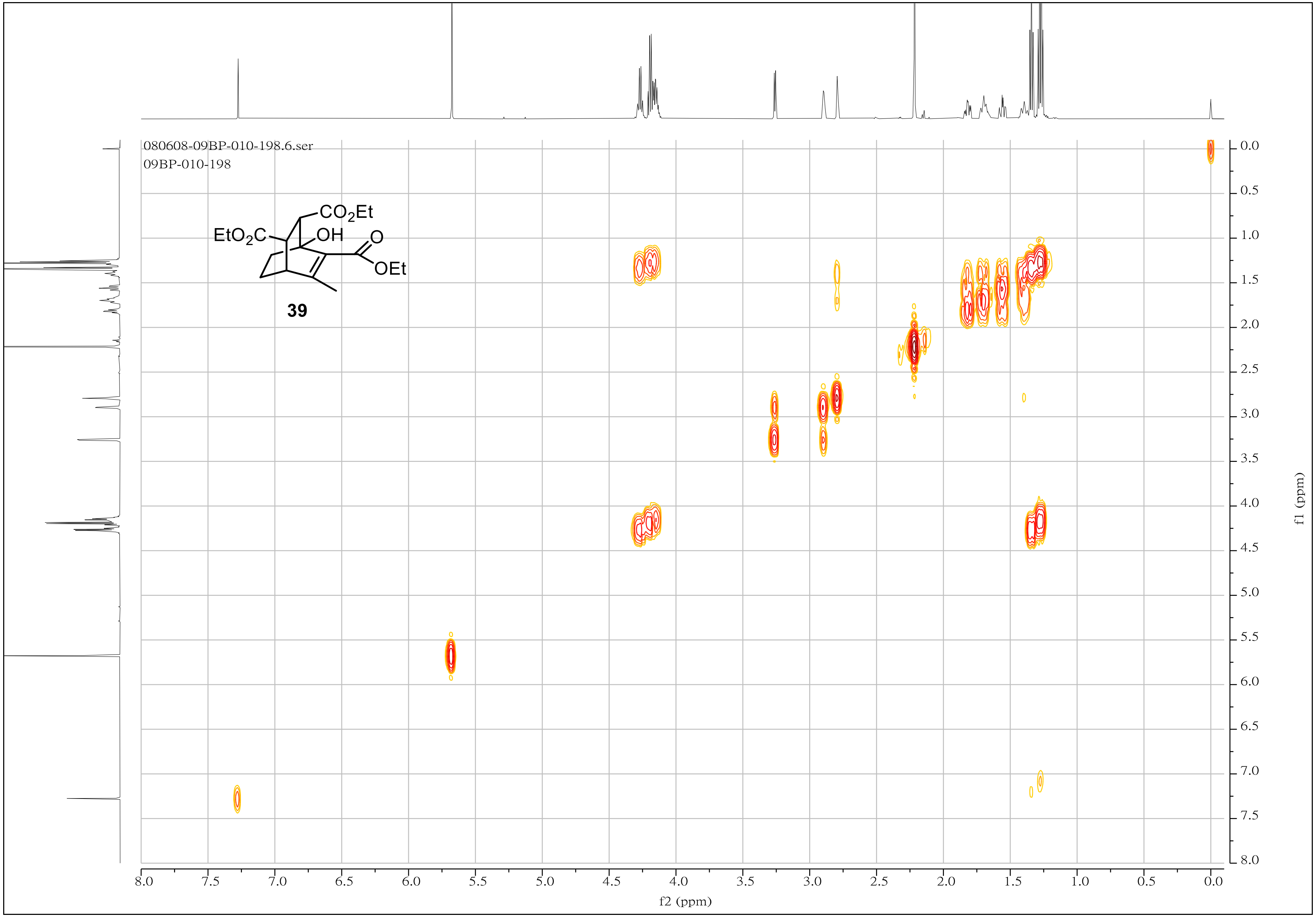


$^1$H-$^1$H COSY spectrum for compound **39**

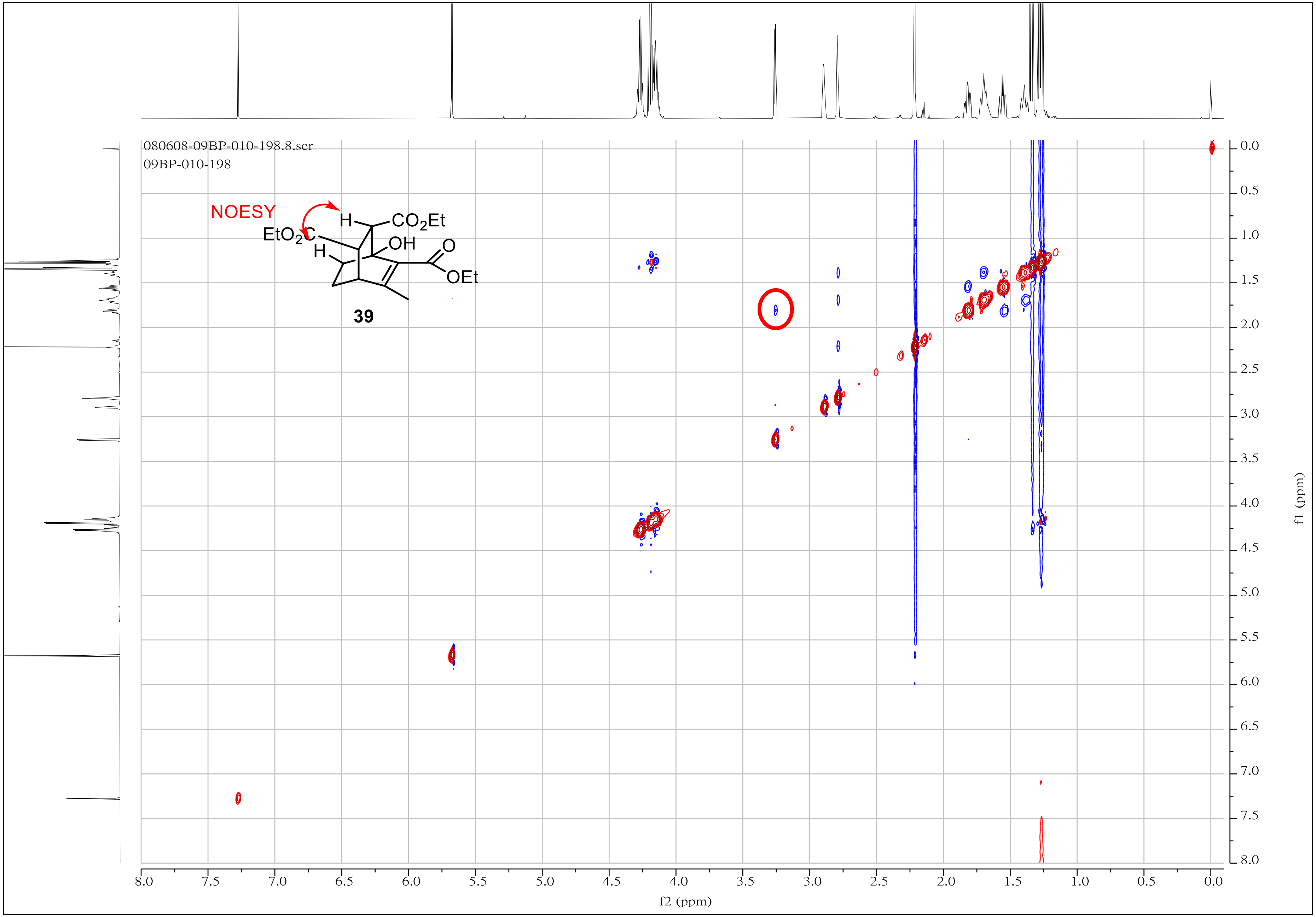


2D NOESY spectrum for compound **39**

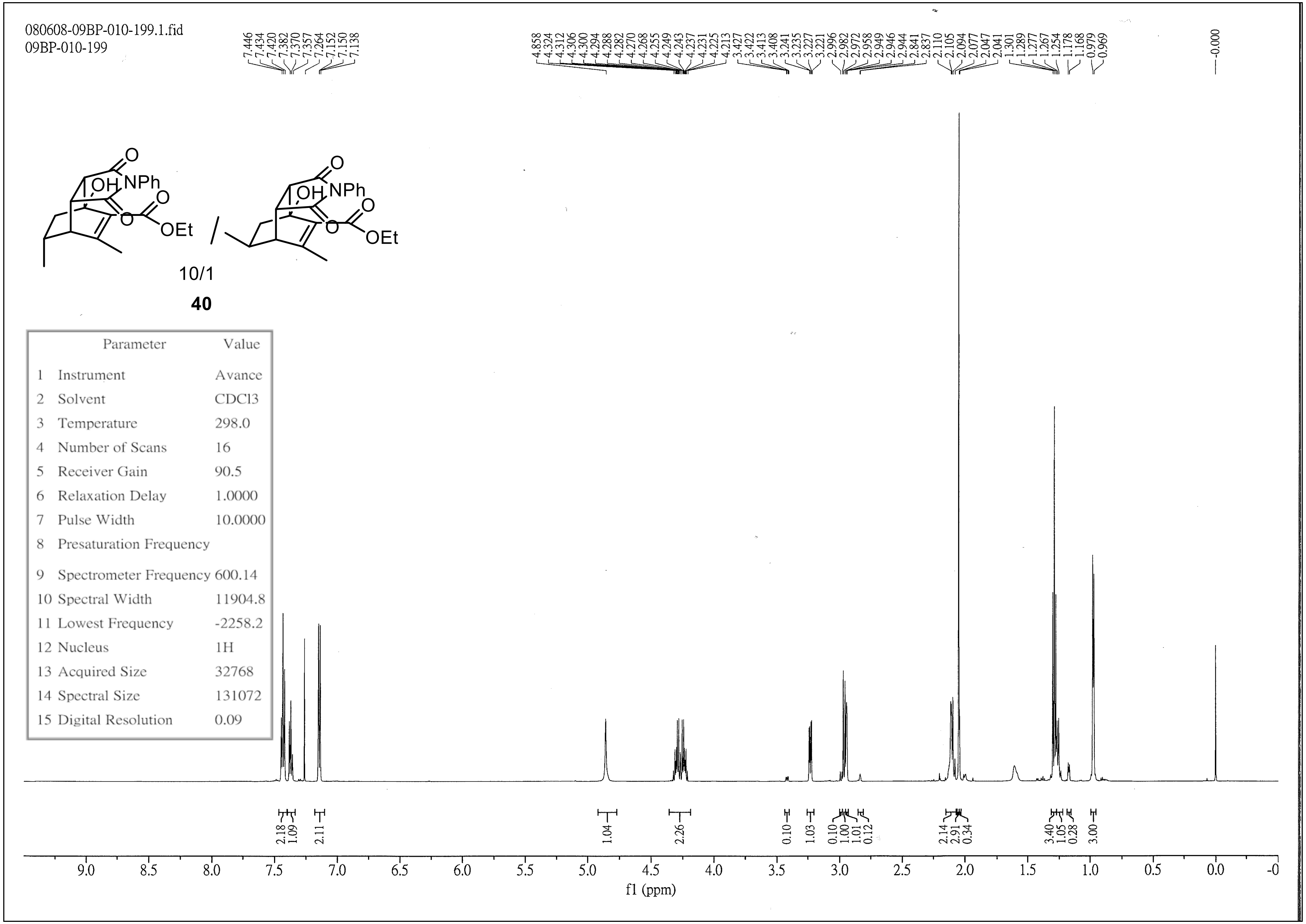


$^{1}$H NMR spectrum for compound **40**

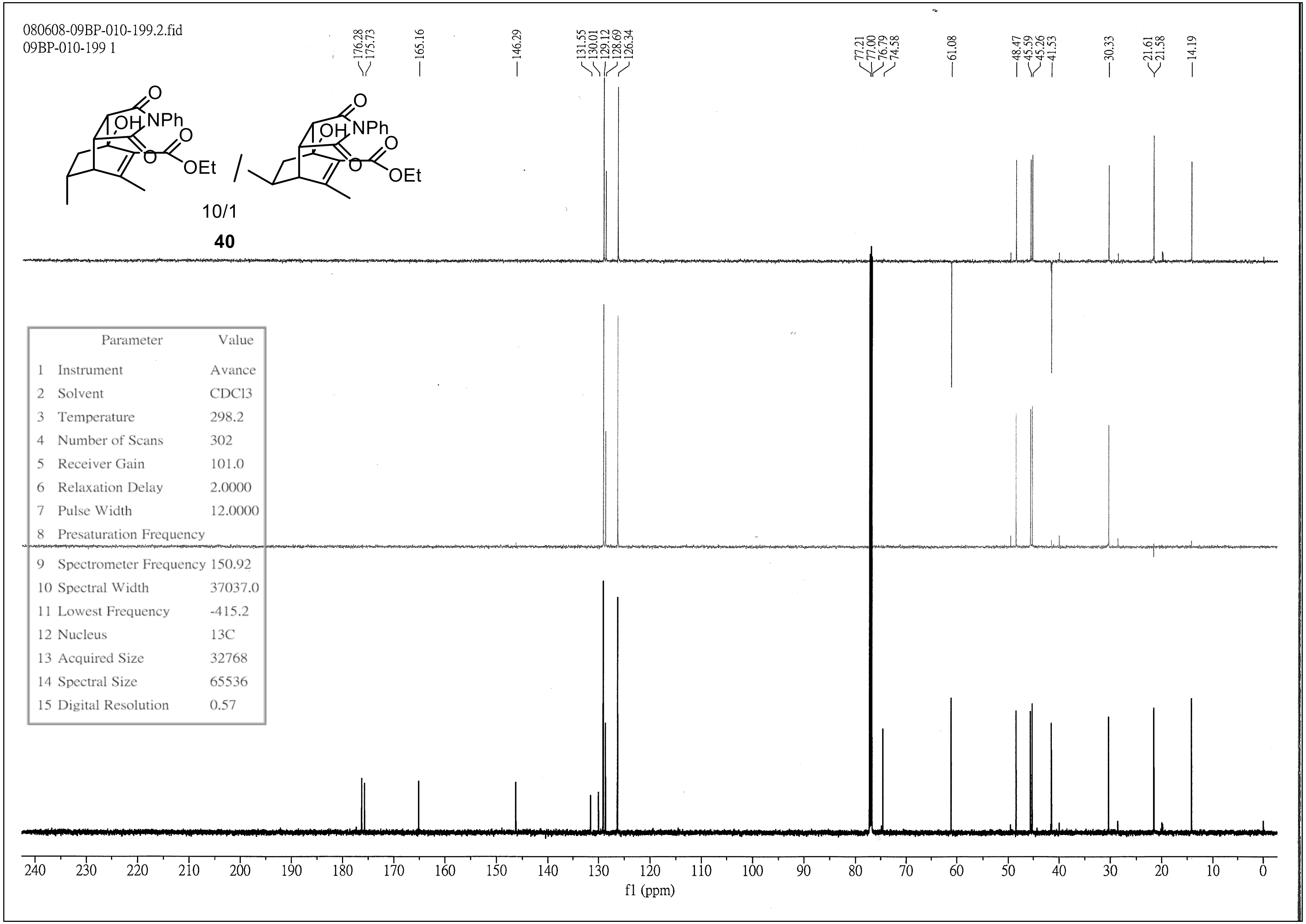


$^{13}$C NMR + DEPT spectra for compound **40**

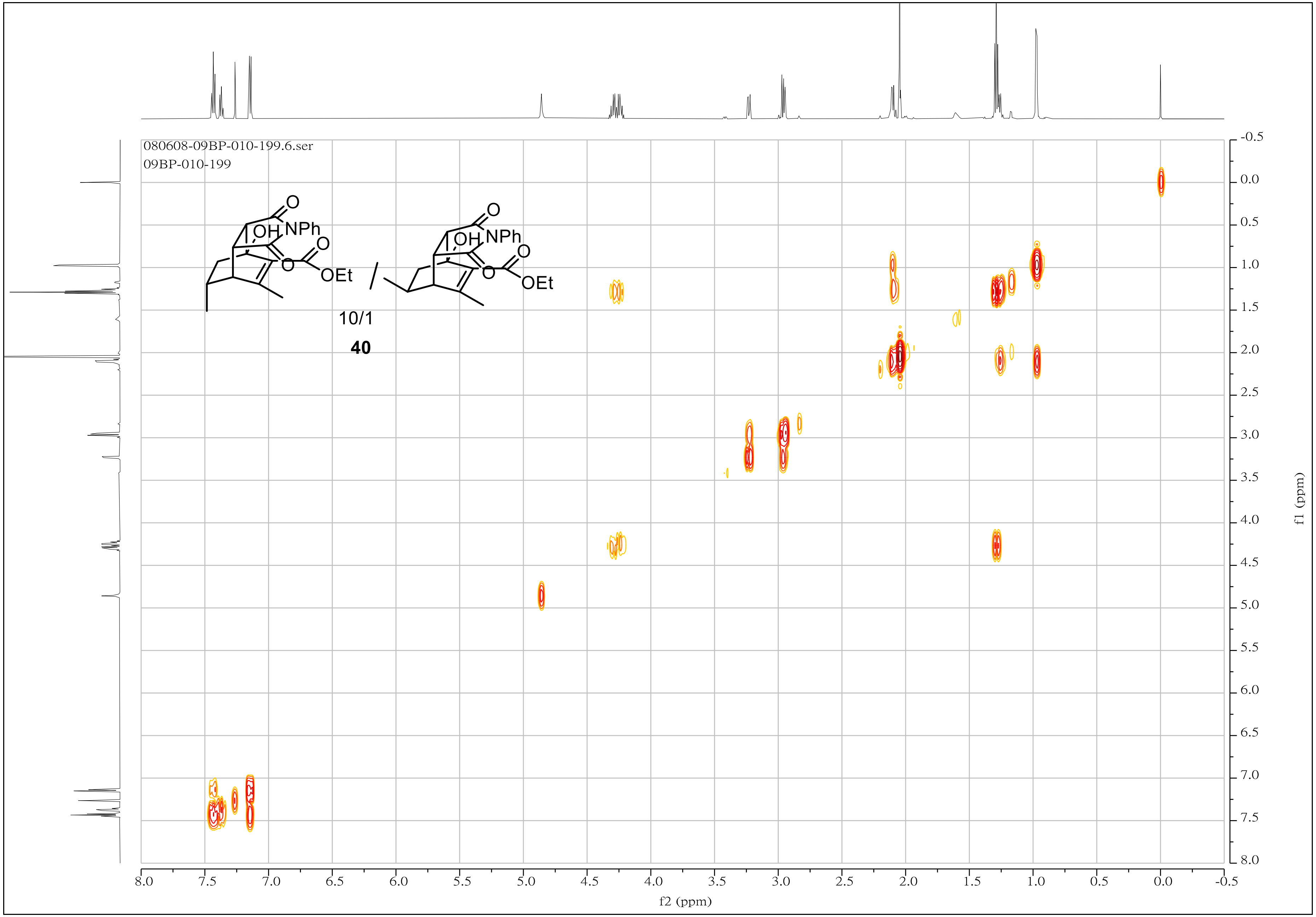


$^{1}H$-$^{1}H$ COSY spectrum for compound **40**

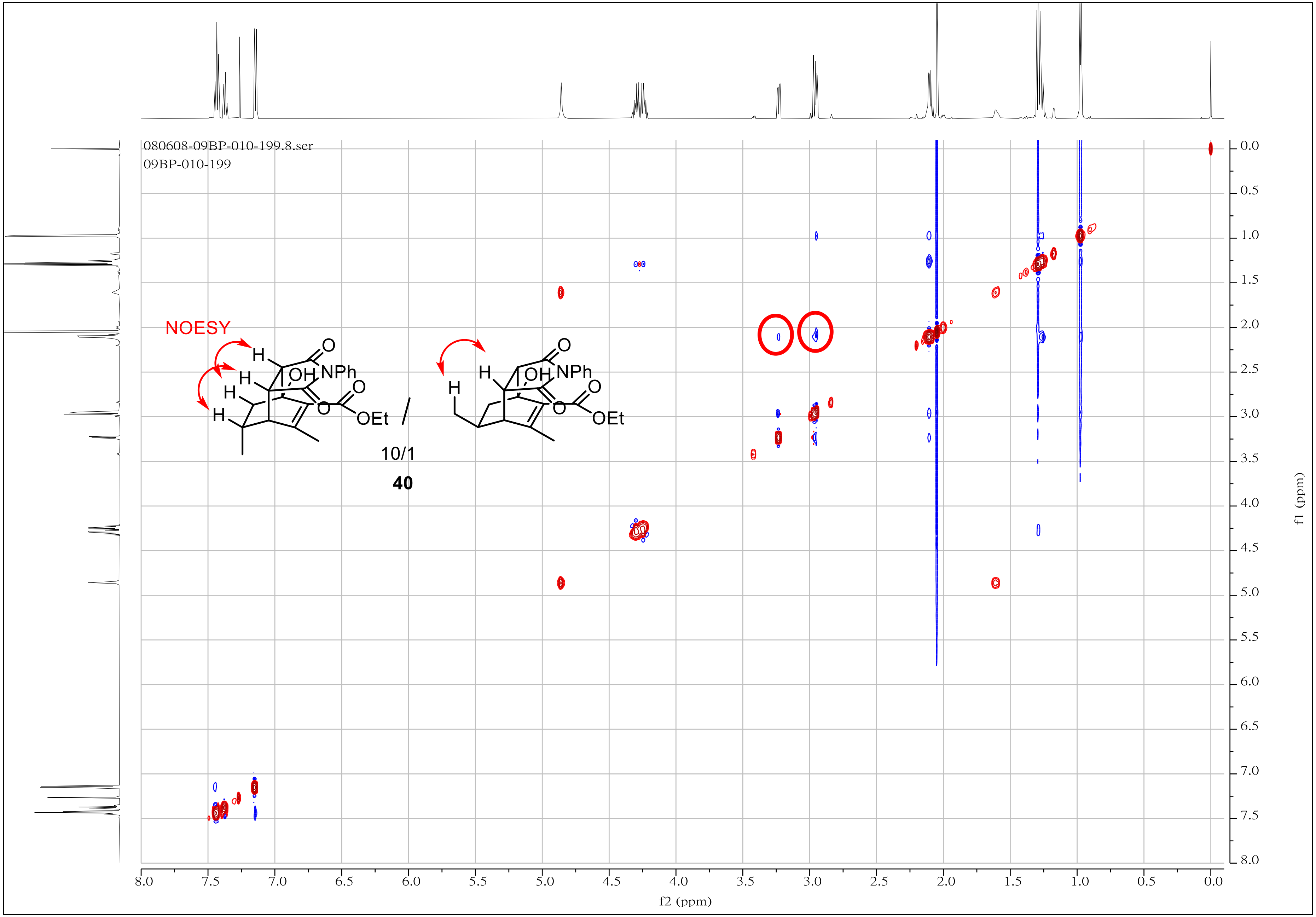


2D NOESY spectrum for compound **40**

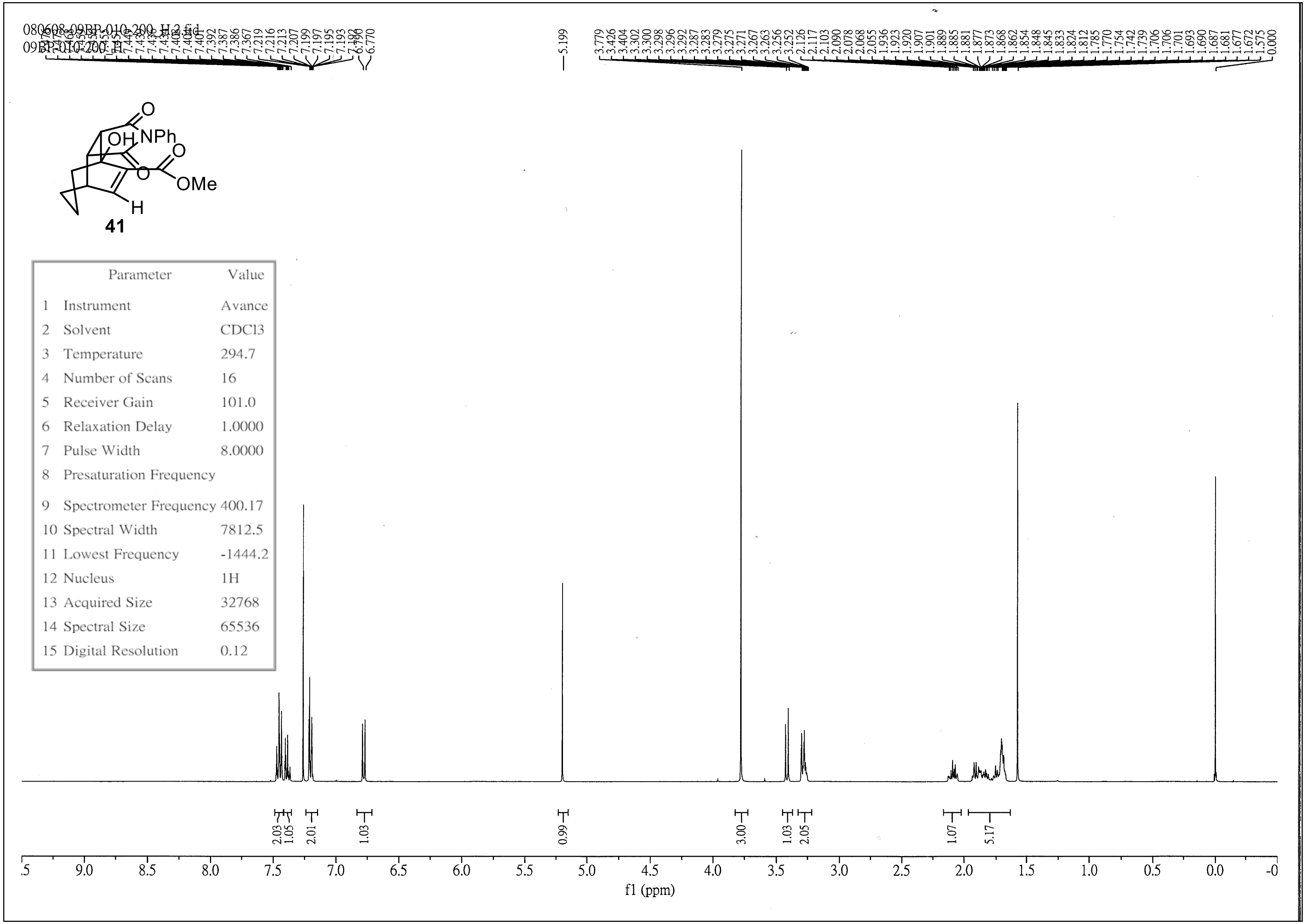


$^{1}$H NMR spectrum for compound **41**

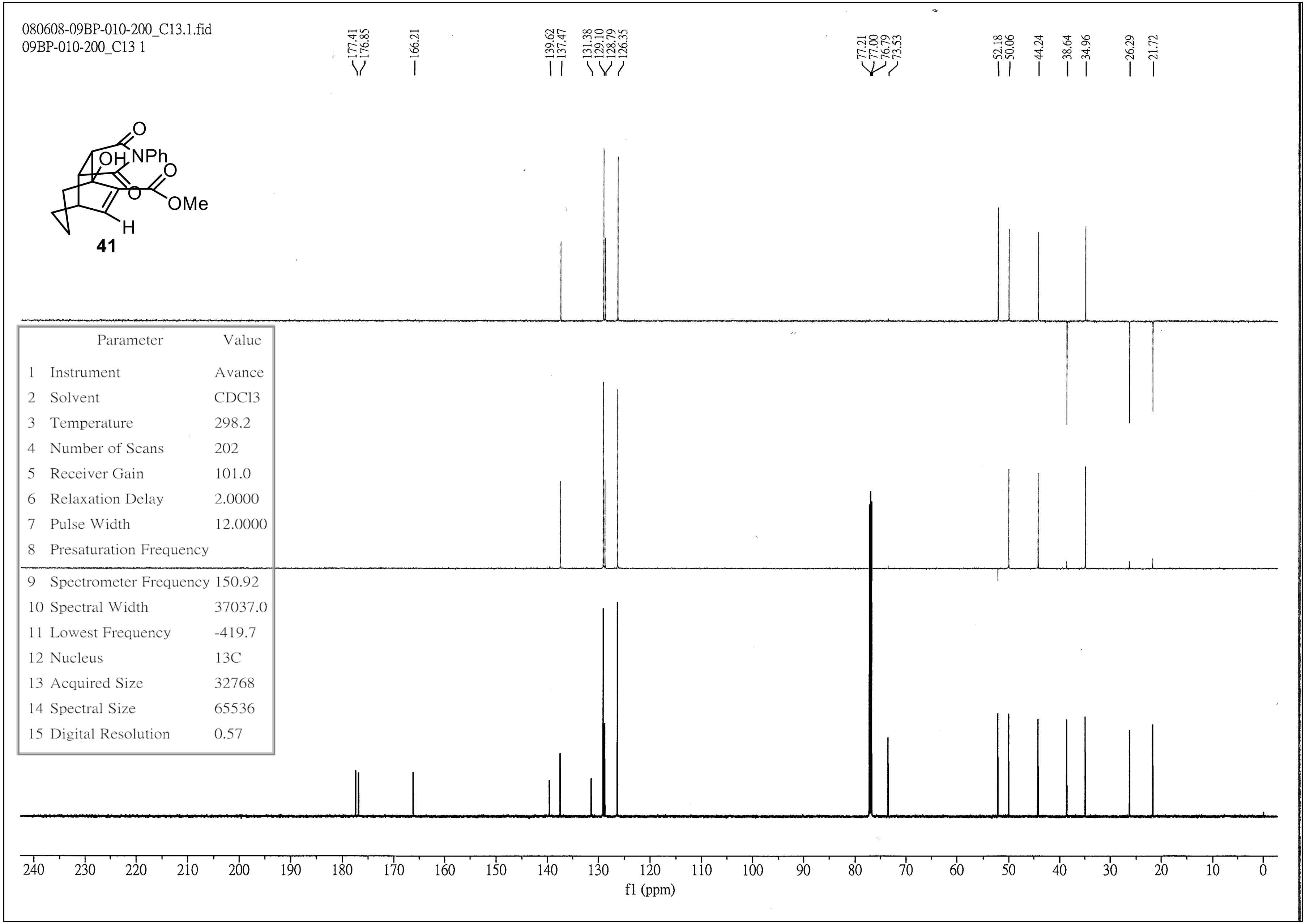


$^{13}$C NMR + DEPT spectra for compound **41**

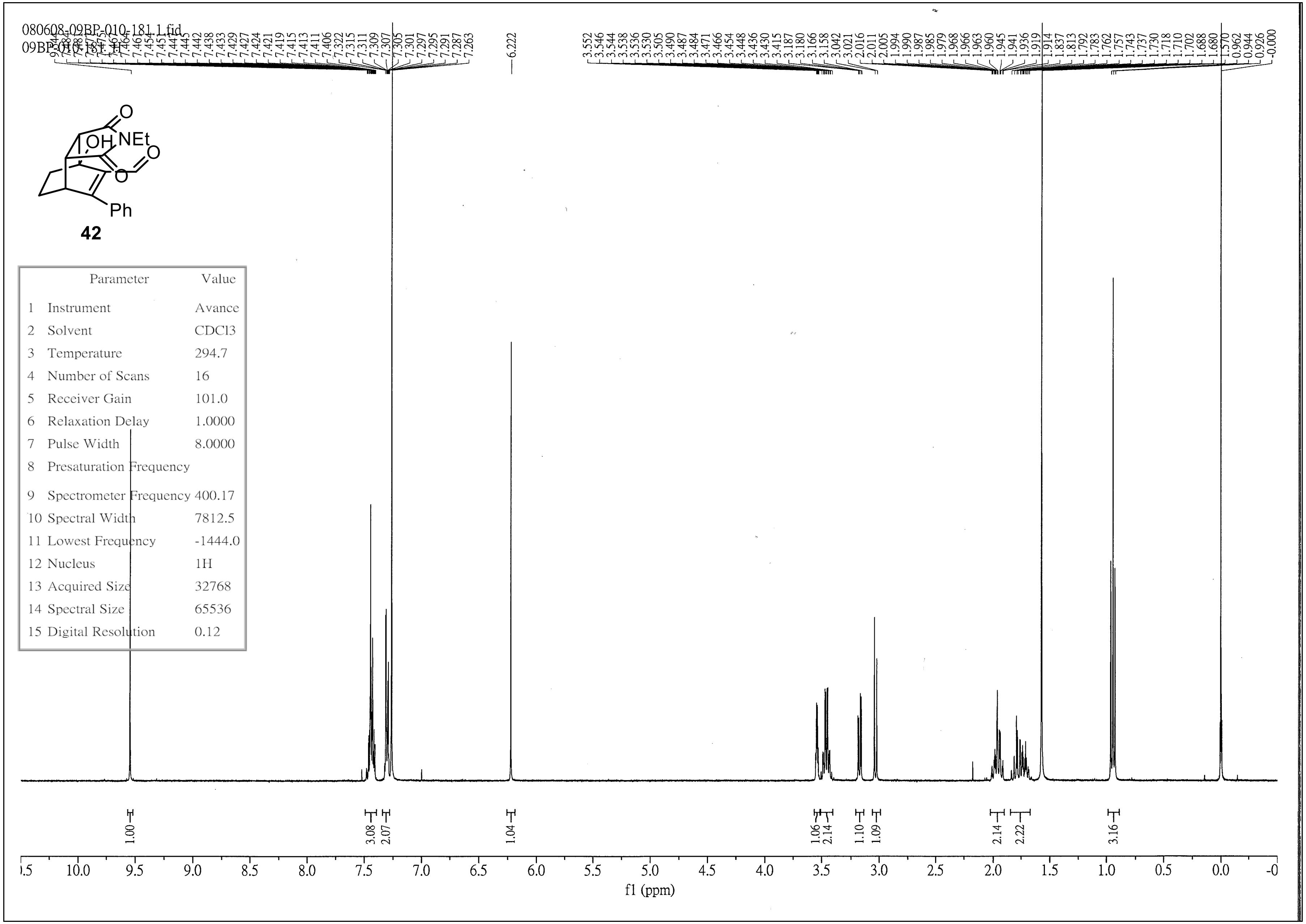


$^{1}$H NMR spectrum for compound **42**

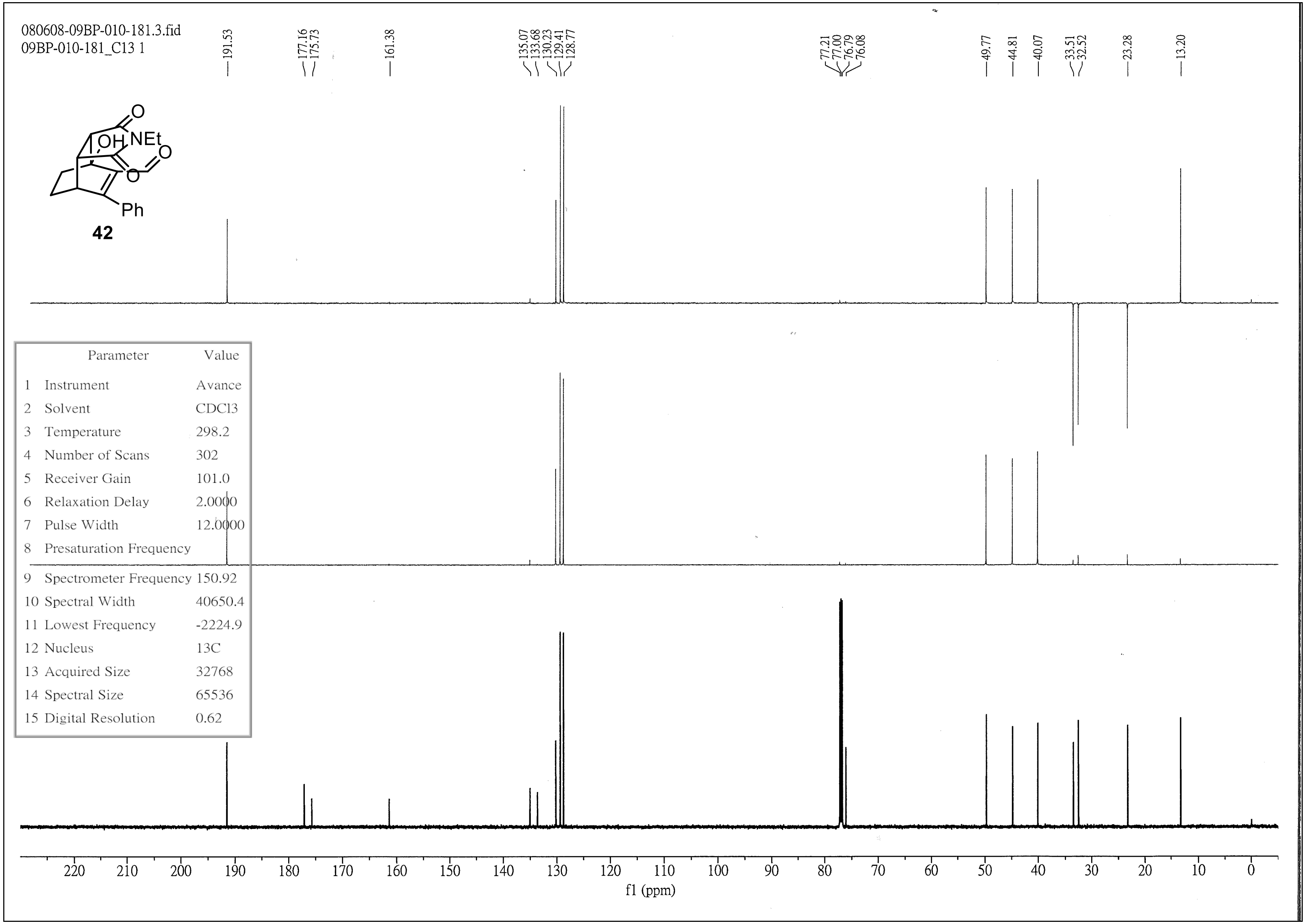


$^{13}C$ NMR + DEPT spectra for compound **42**

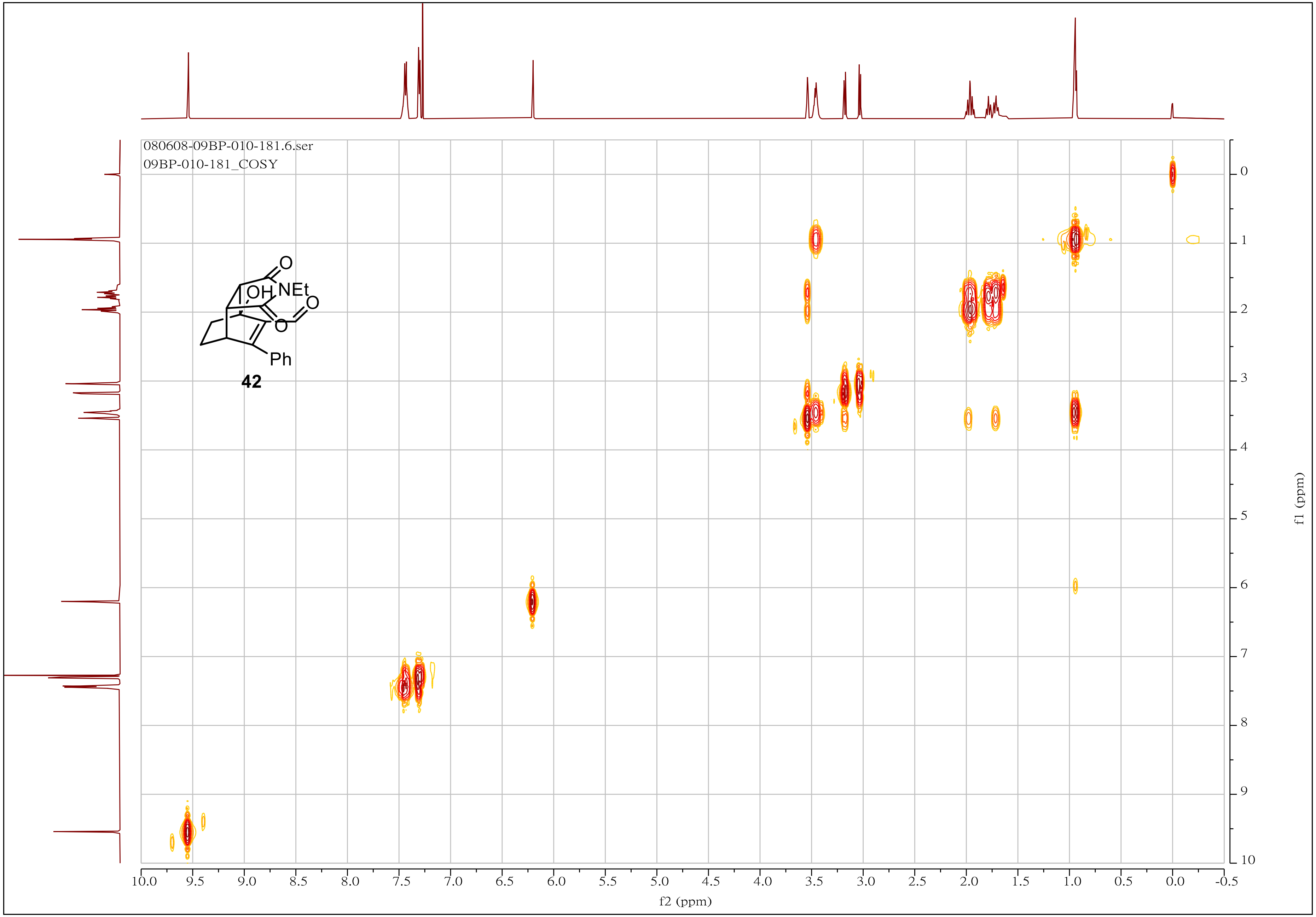


$^1$H-$^1$H COSY spectrum for compound **42**

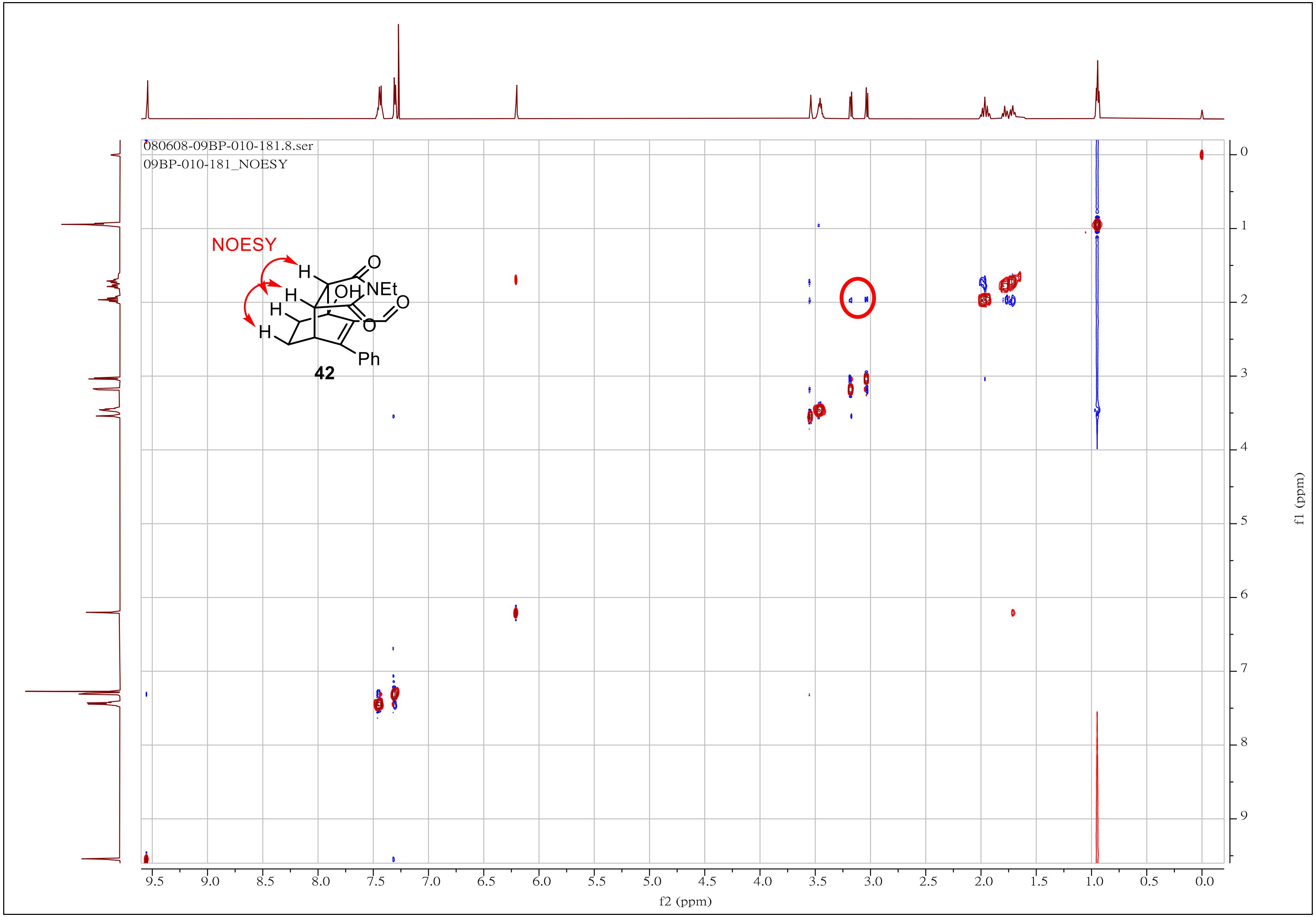


2D NOESY spectrum for compound **42**

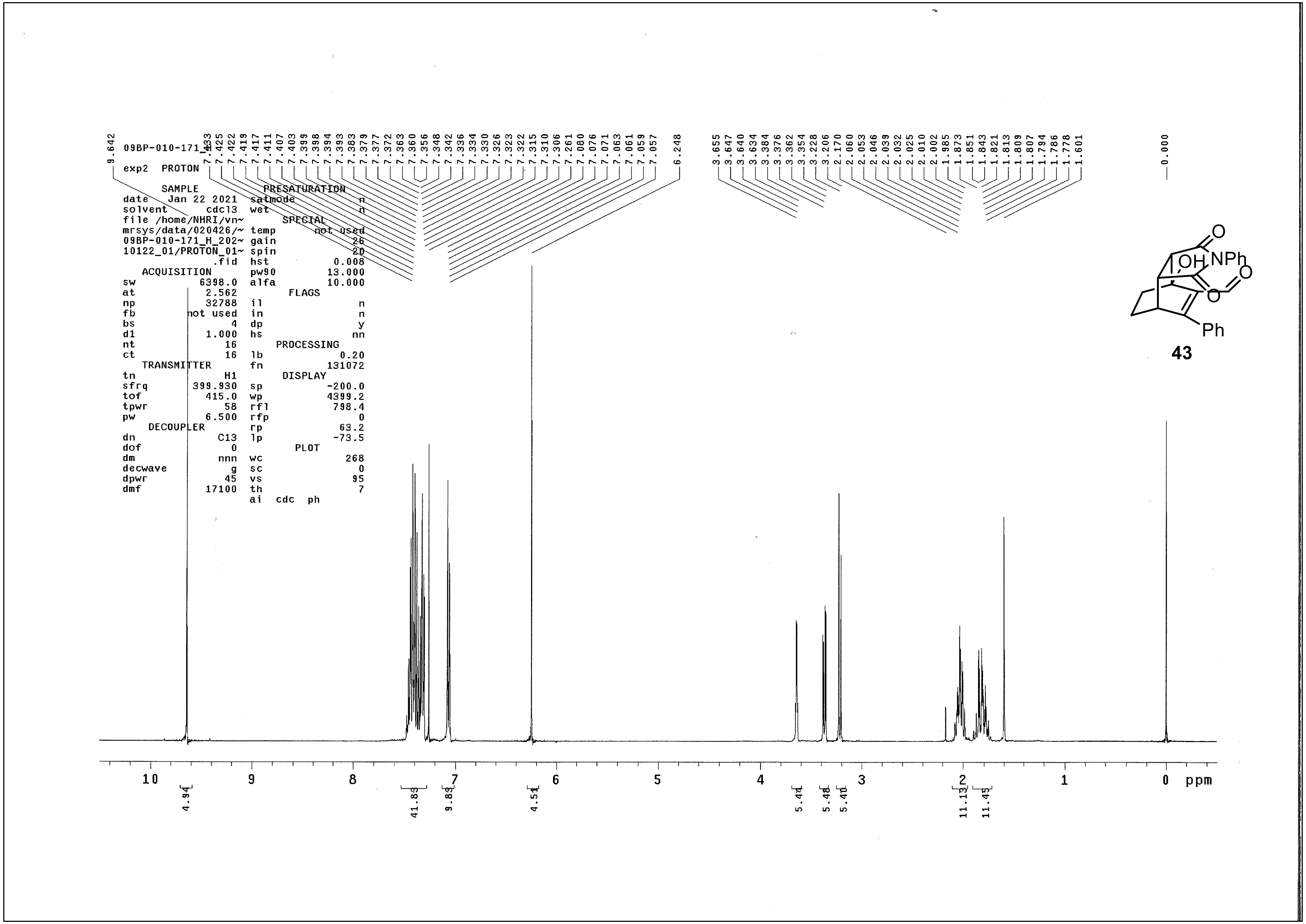

$^{1}$H NMR spectrum for compound **43**

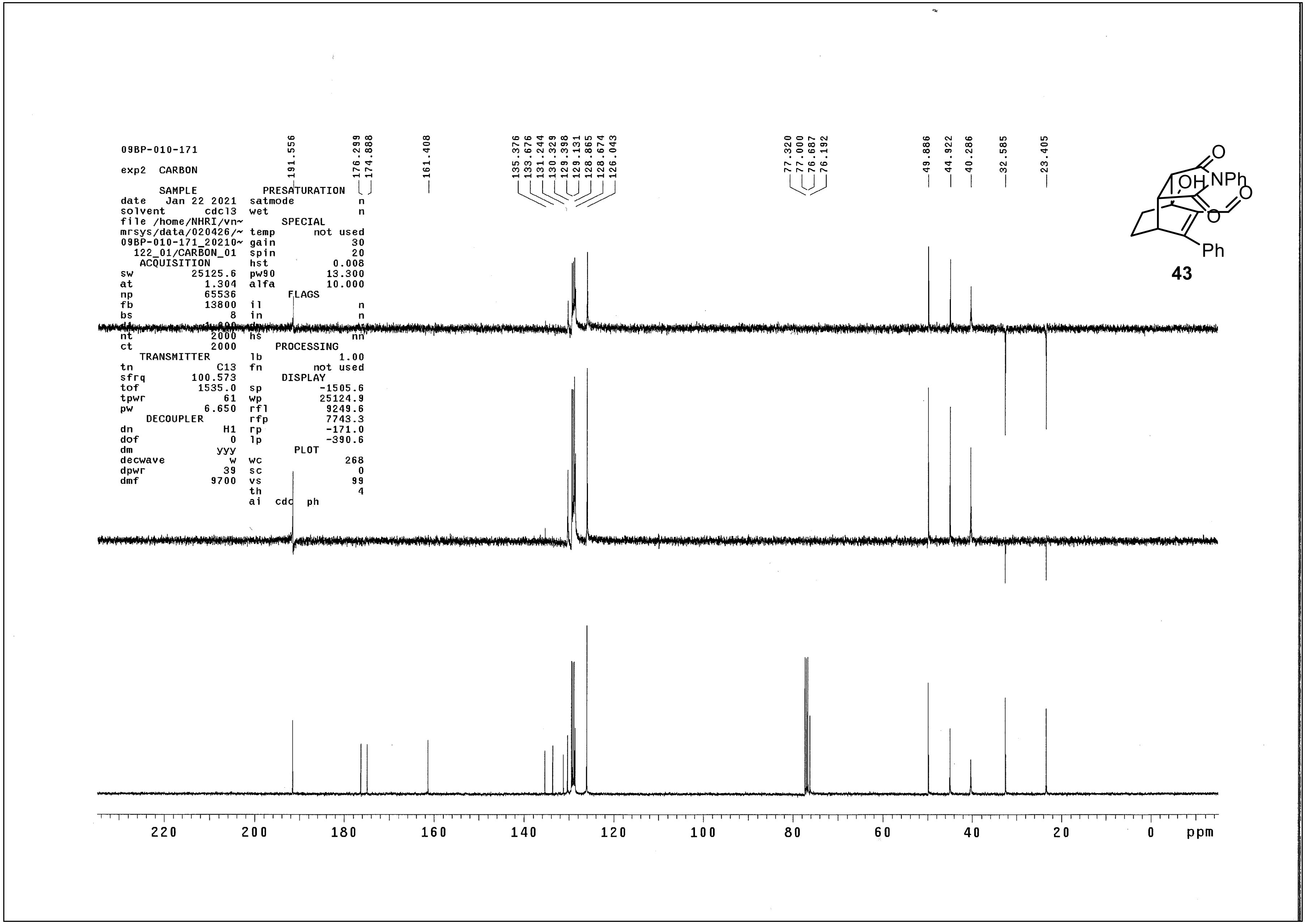


$^{13}$C NMR + DEPT spectra for compound **43**

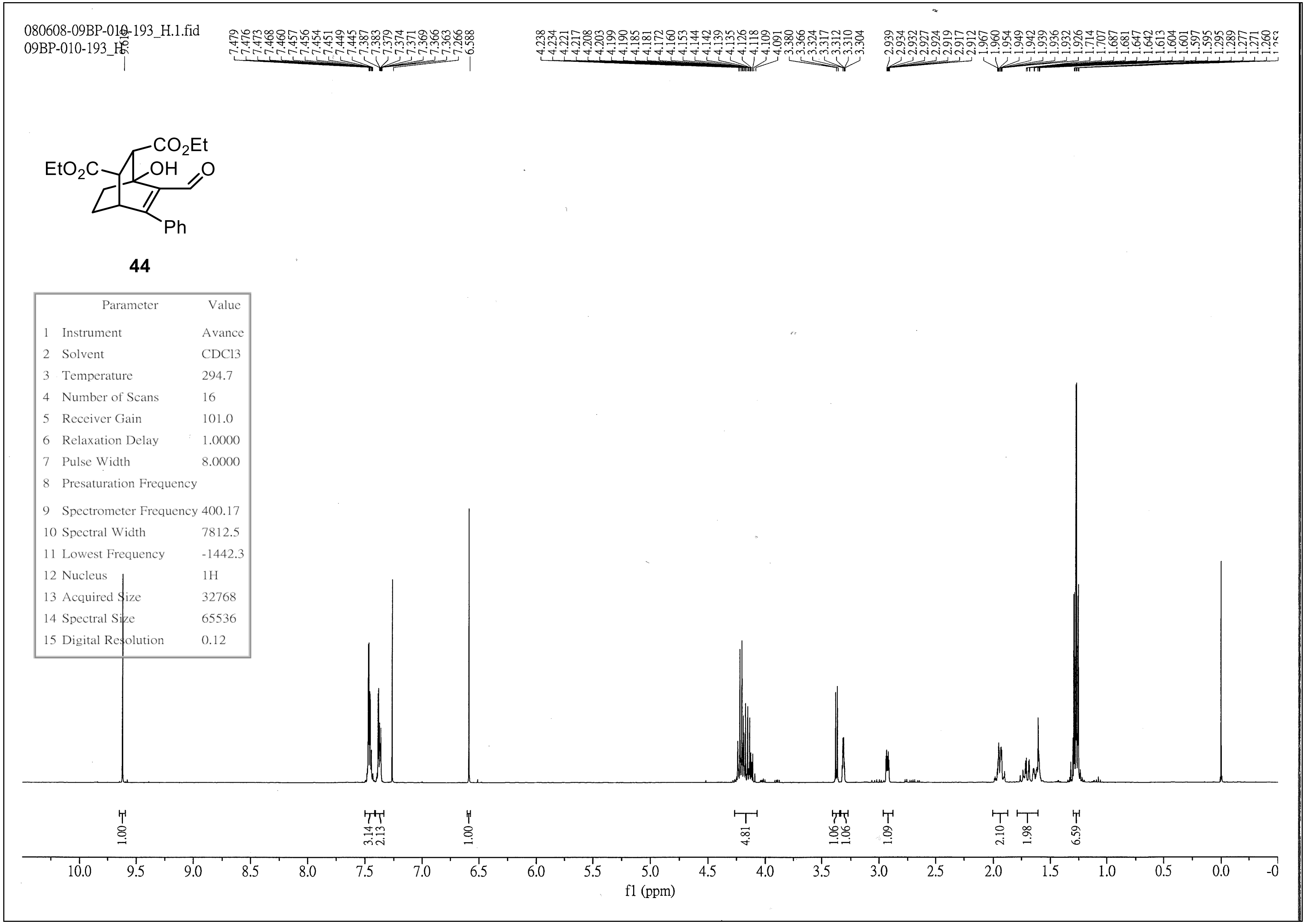

| Parameter | Value |
|---|---|
| 1 Instrument | Avance |
| 2 Solvent | CDCl3 |
| 3 Temperature | 294.7 |
| 4 Number of Scans | 16 |
| 5 Receiver Gain | 101.0 |
| 6 Relaxation Delay | 1.0000 |
| 7 Pulse Width | 8.0000 |
| 8 Presaturation Frequency | |
| 9 Spectrometer Frequency | 400.17 |
| 10 Spectral Width | 7812.5 |
| 11 Lowest Frequency | -1442.3 |
| 12 Nucleus | 1H |
| 13 Acquired Size | 32768 |
| 14 Spectral Size | 65536 |
| 15 Digital Resolution | 0.12 |

$^{1}$H NMR spectrum for compound **44**

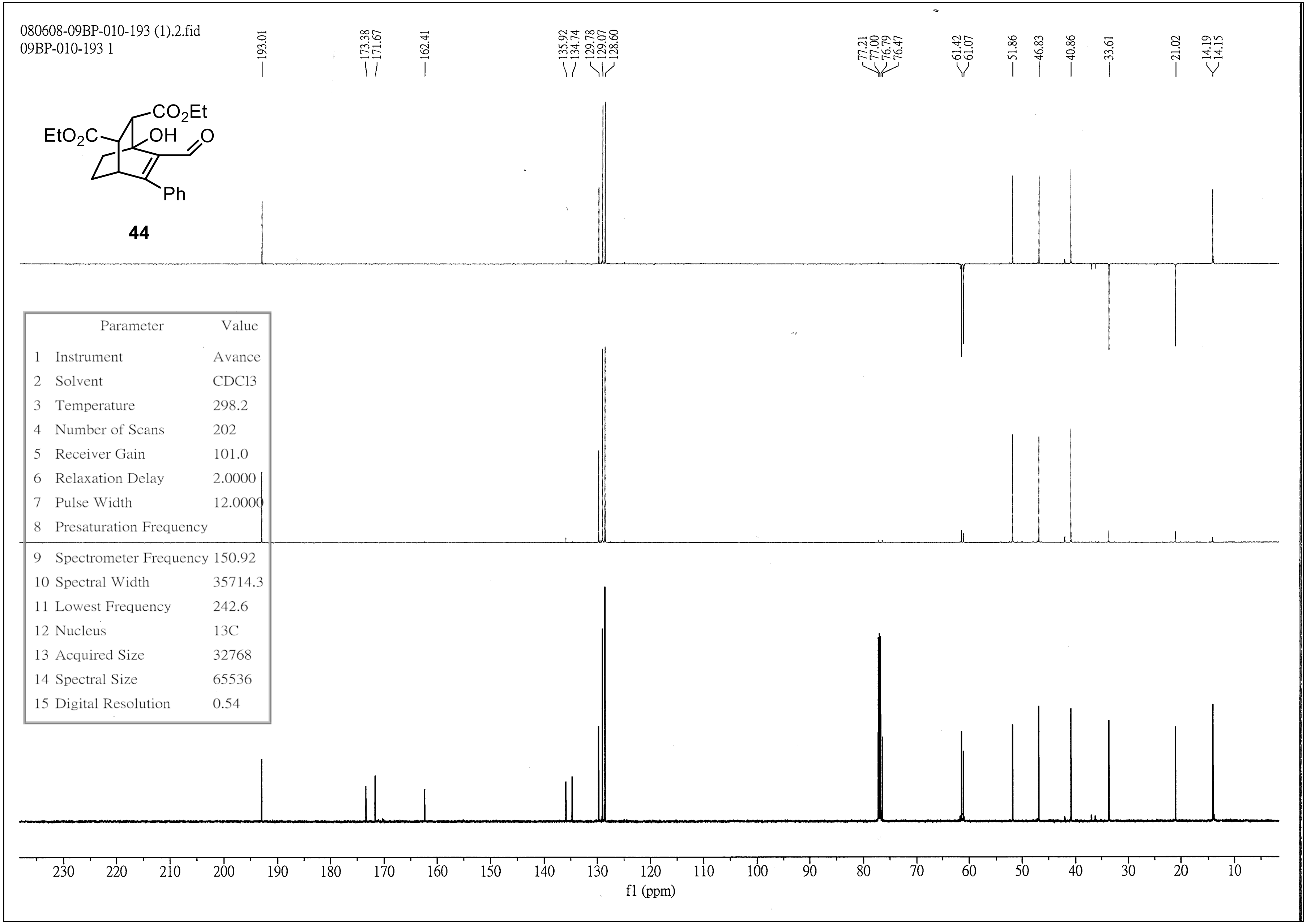


$^{13}$C NMR + DEPT spectra for compound **44**

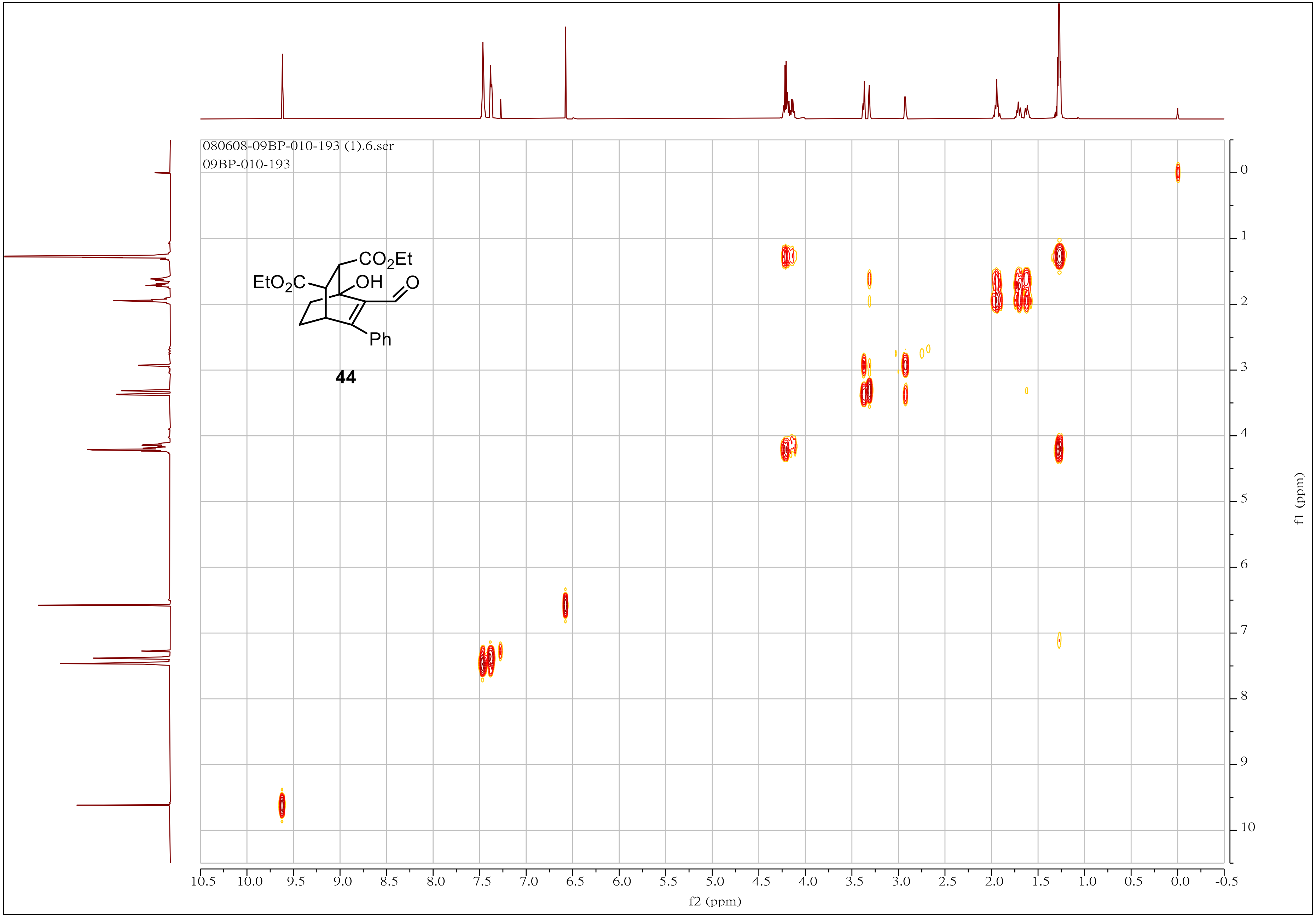


$^1$H-$^1$H COSY spectrum for compound **44**

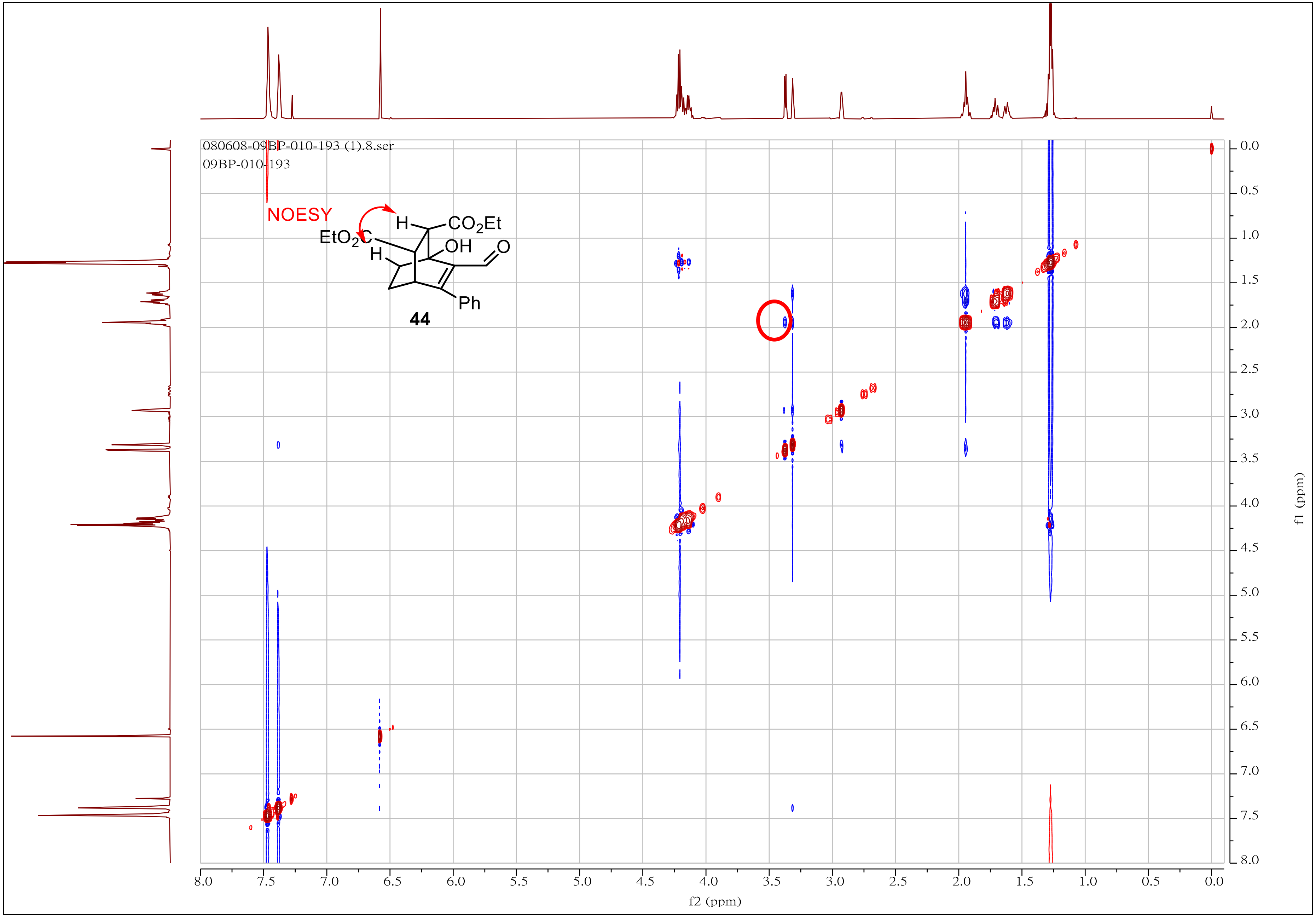


2D NOESY spectrum for compound **44**

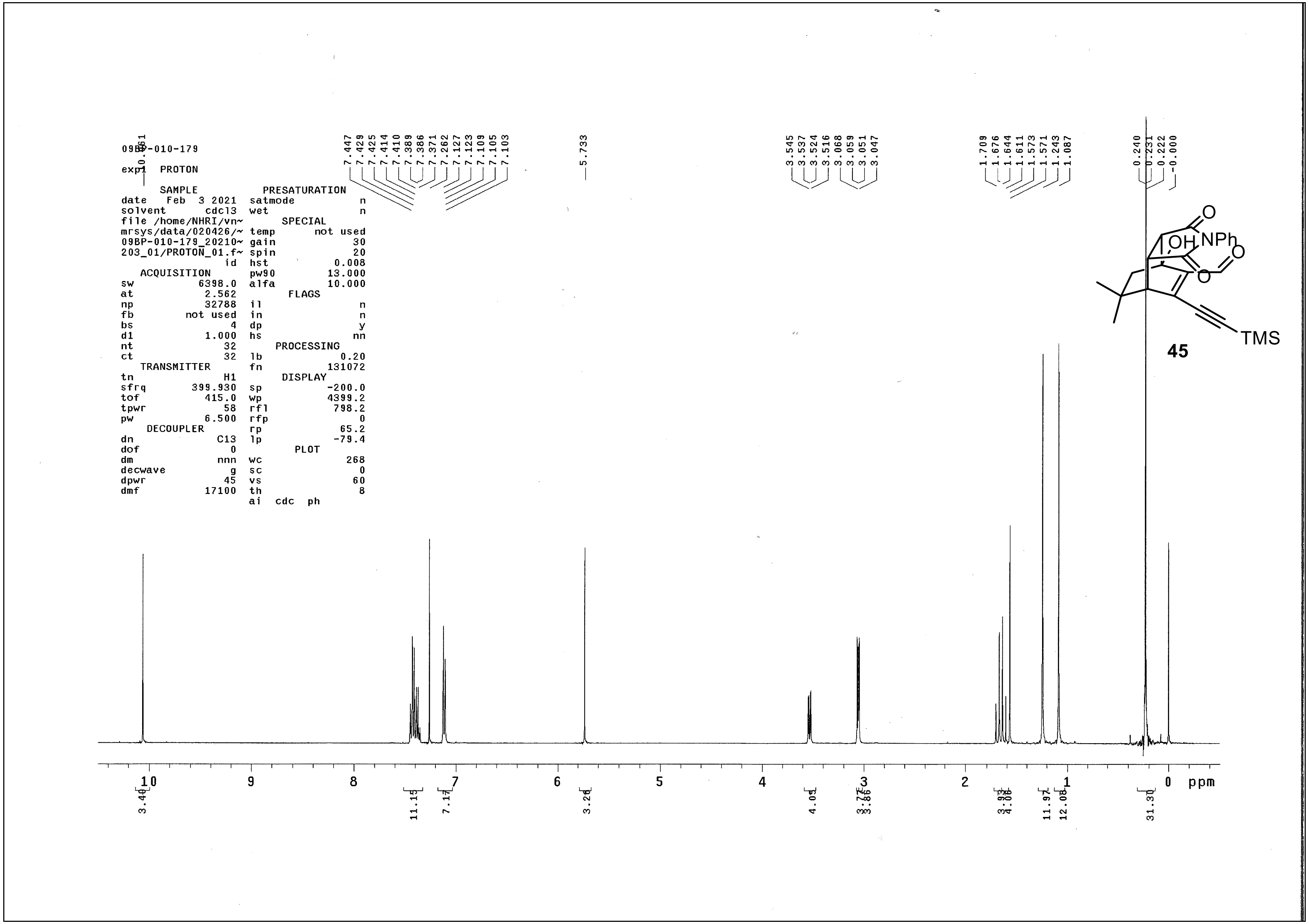

$^{1}$H NMR spectrum for compound **45**

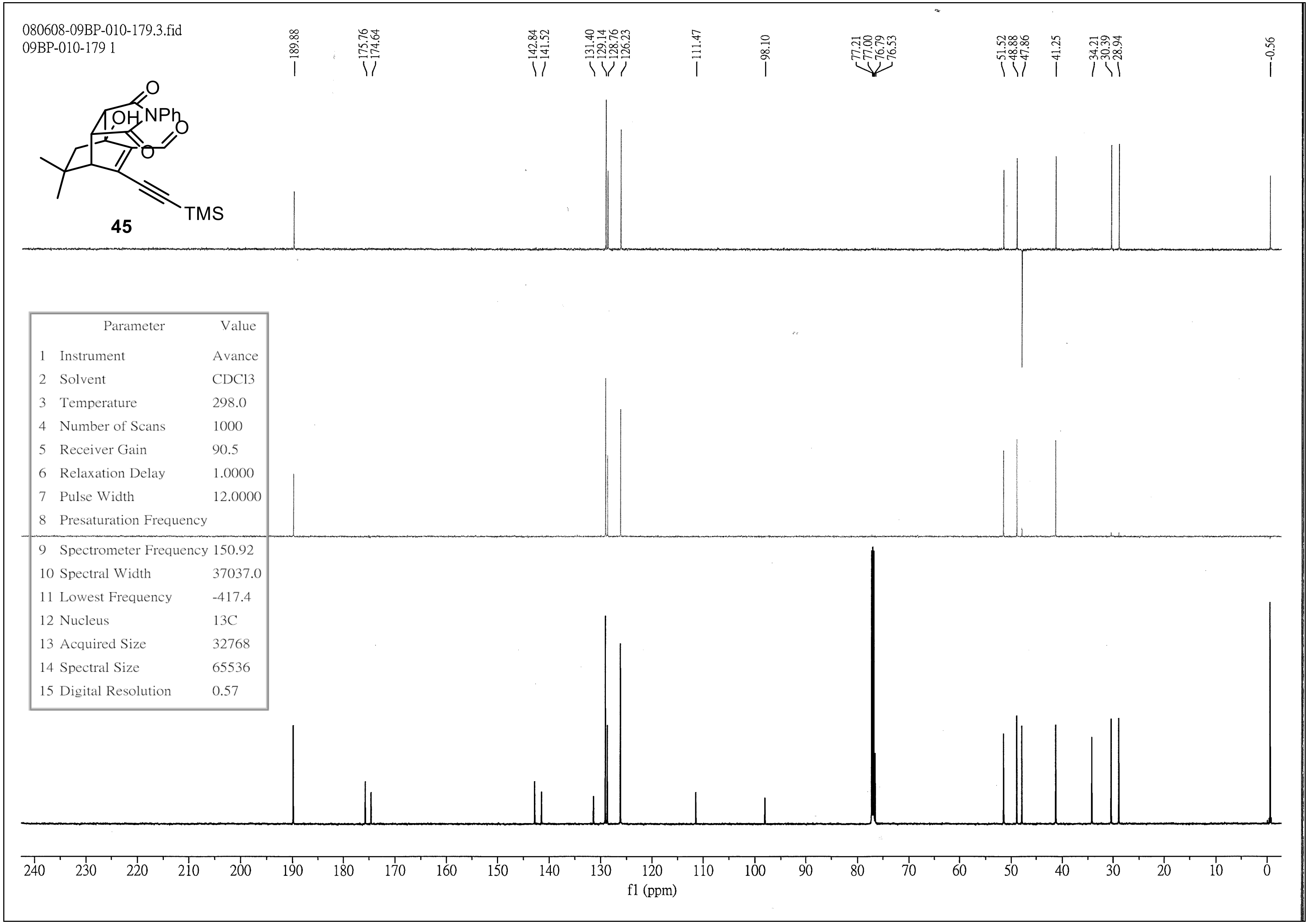


$^{13}C$ NMR + DEPT spectra for compound **45**

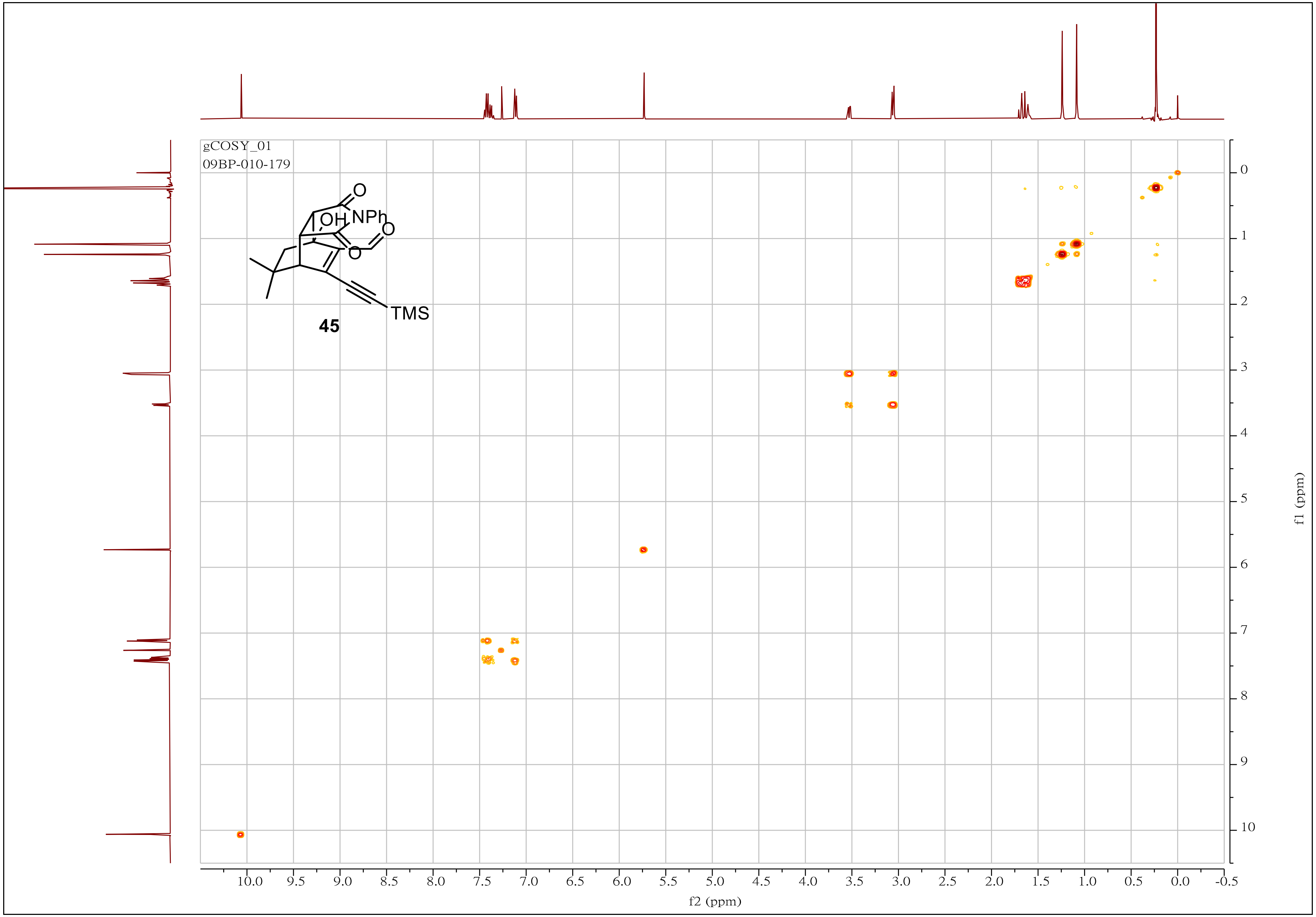


$^{1}$H-$^{1}$H COSY spectrum for compound **45**

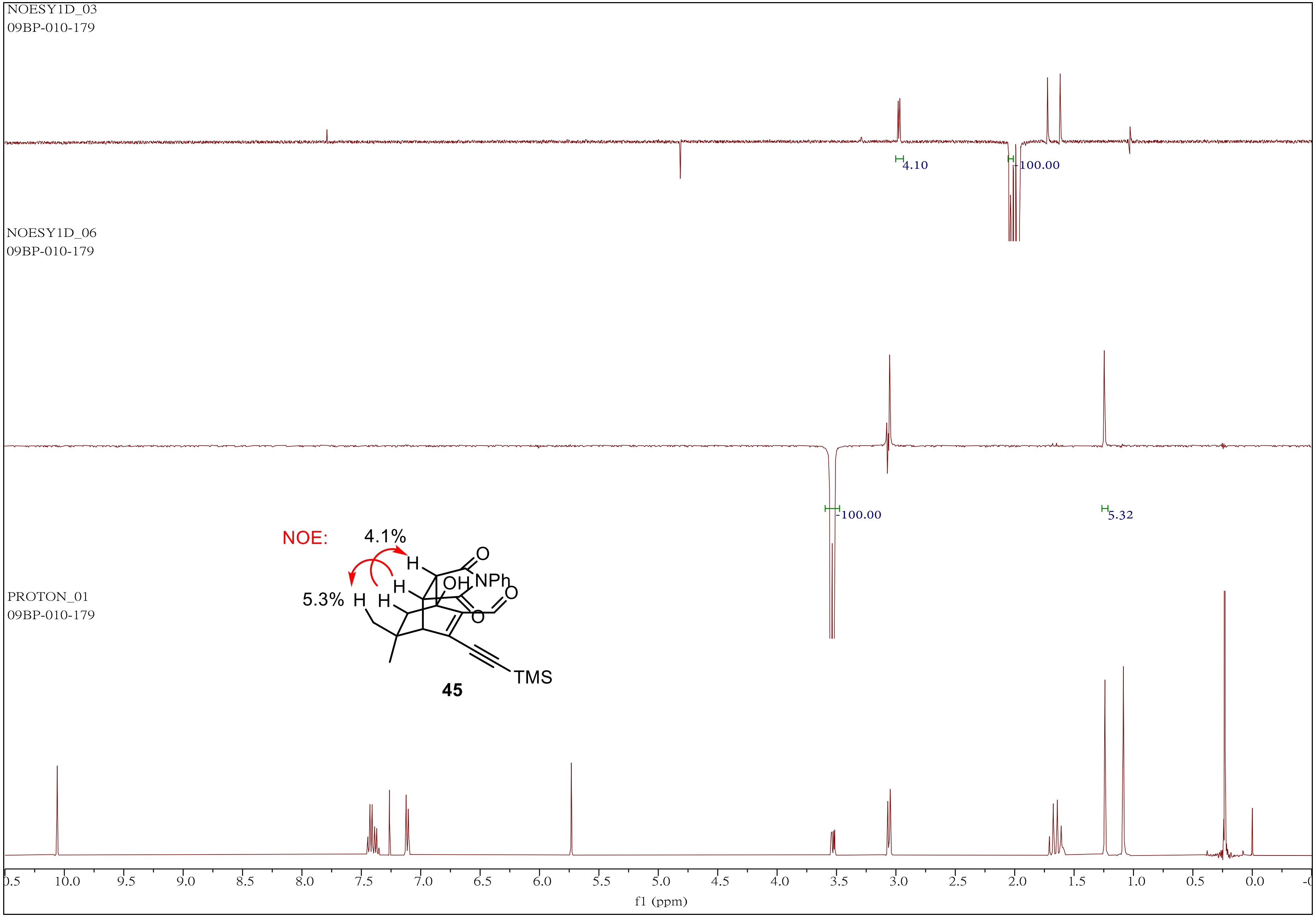


1D NOESY spectra for compound **45**

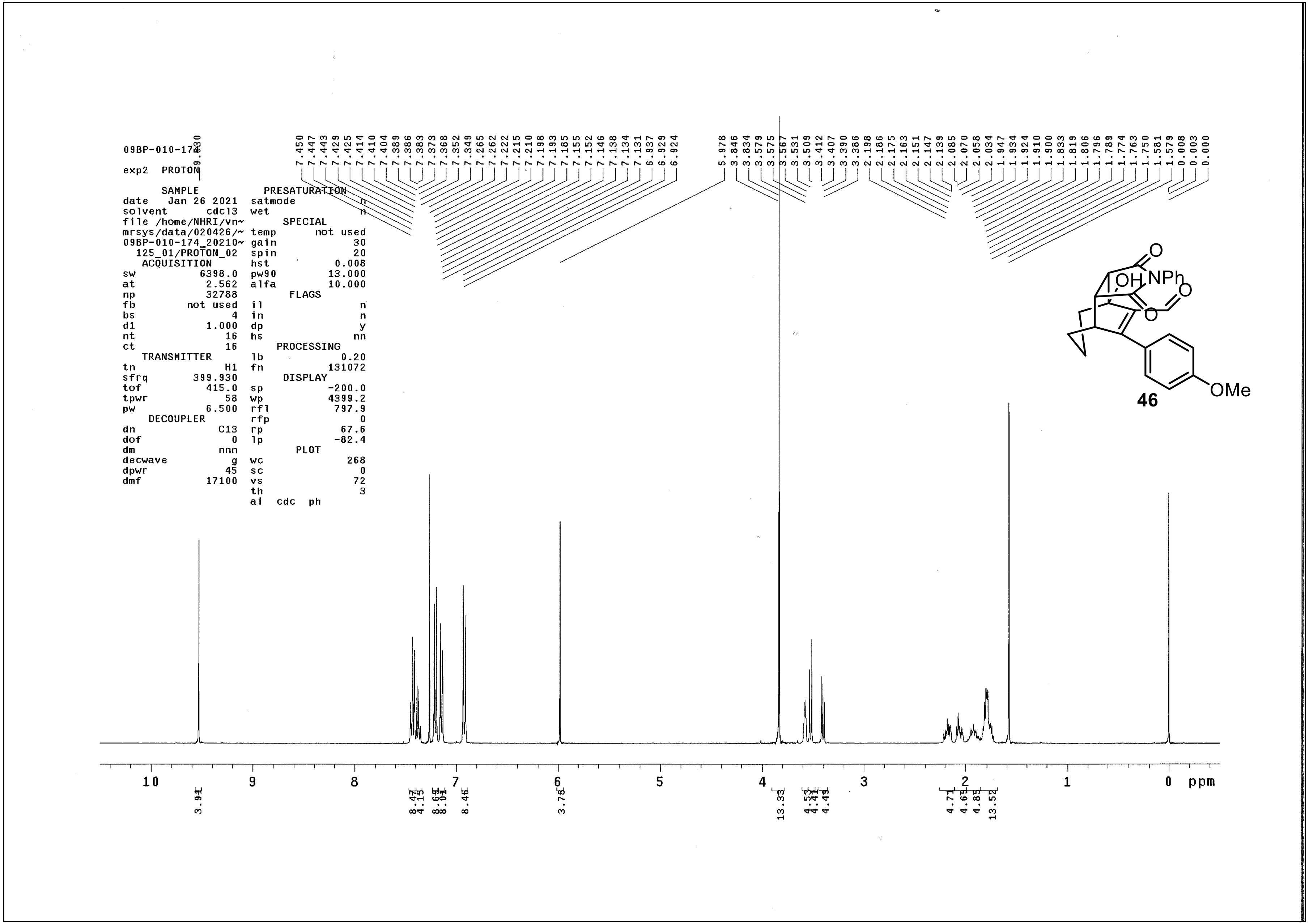

$^{1}$H NMR spectrum for compound **46**

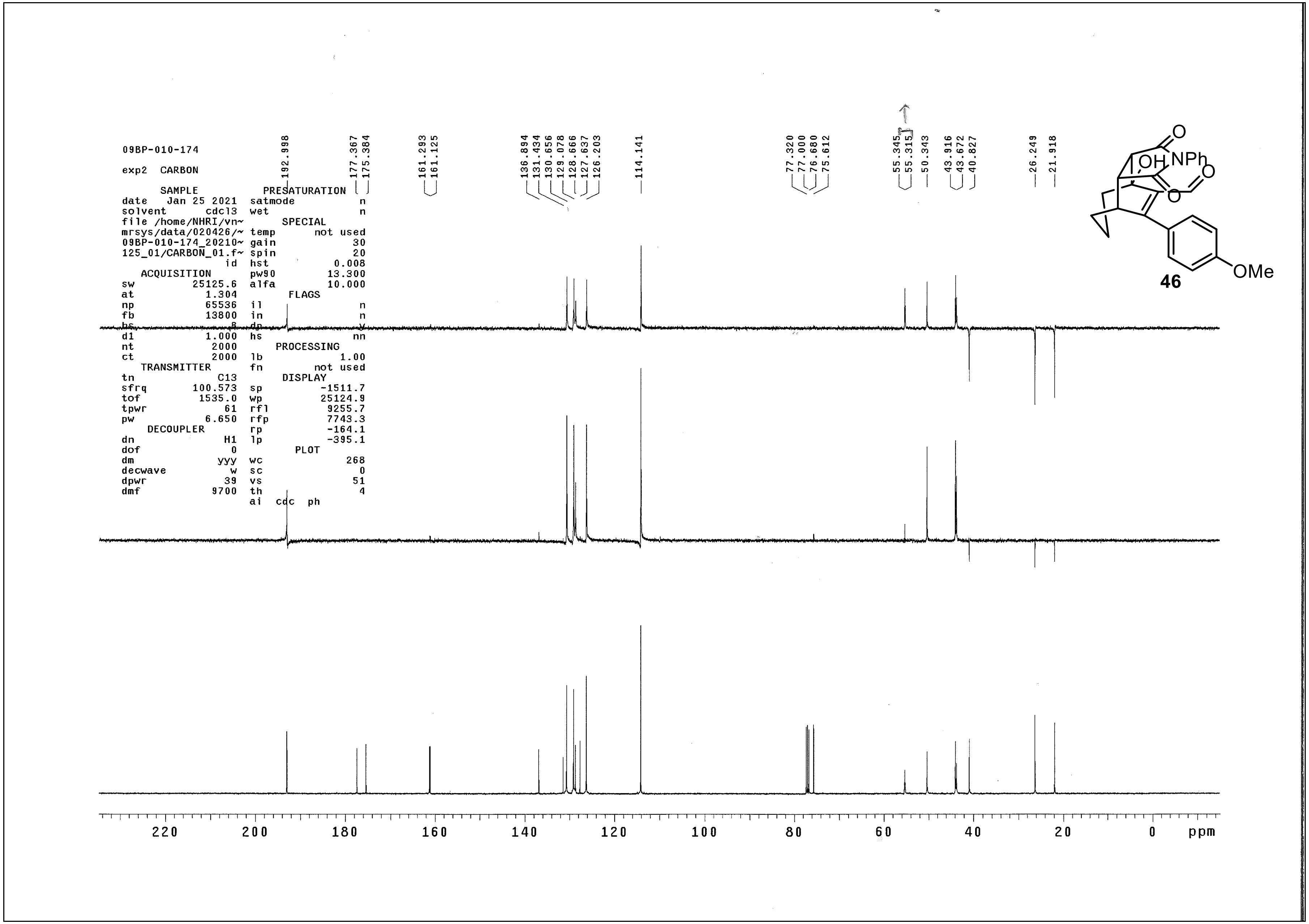


$^{13}$C NMR + DEPT spectra for compound **46**

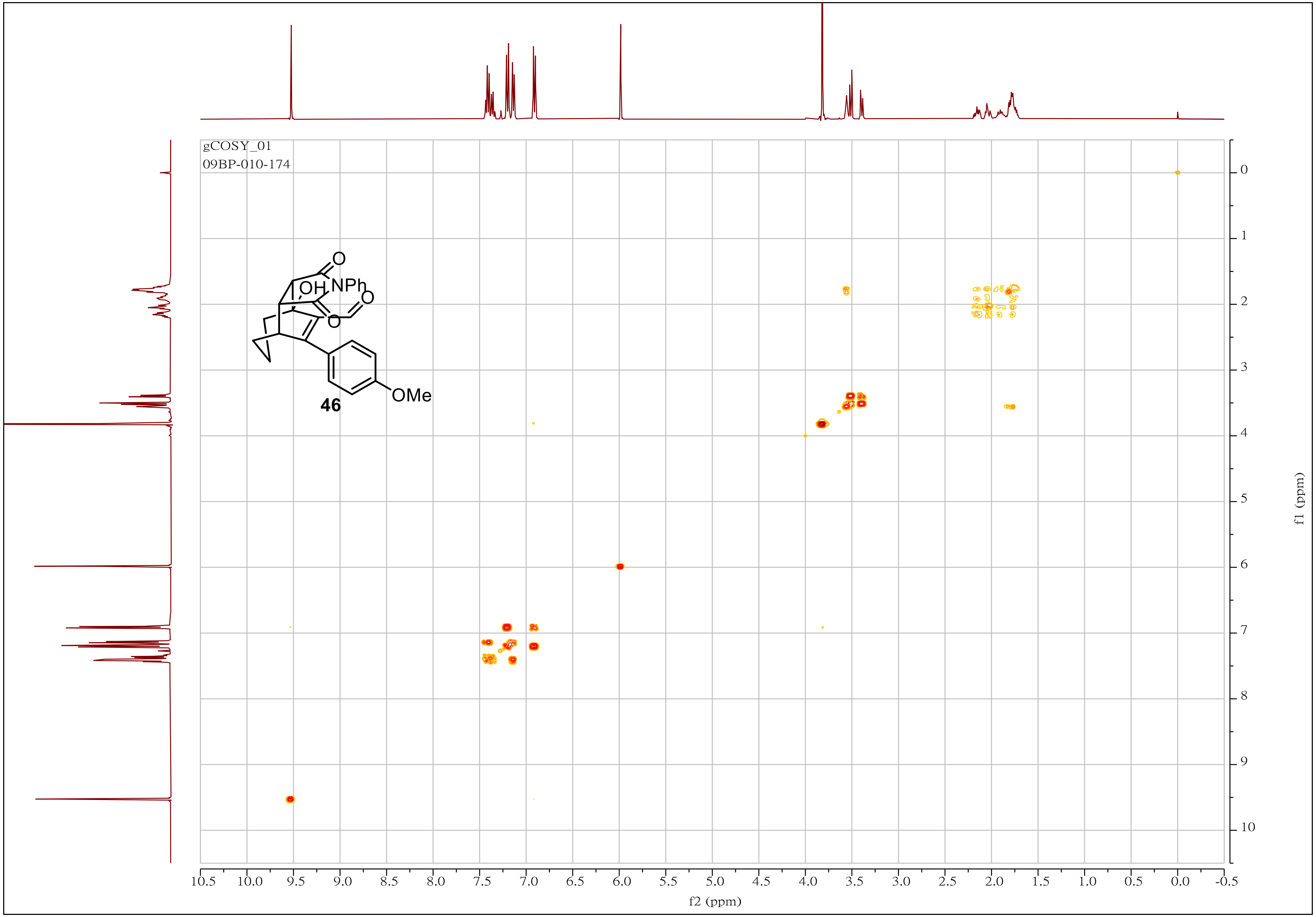


$^{1}$H-$^{1}$H COSY spectrum for compound **46**

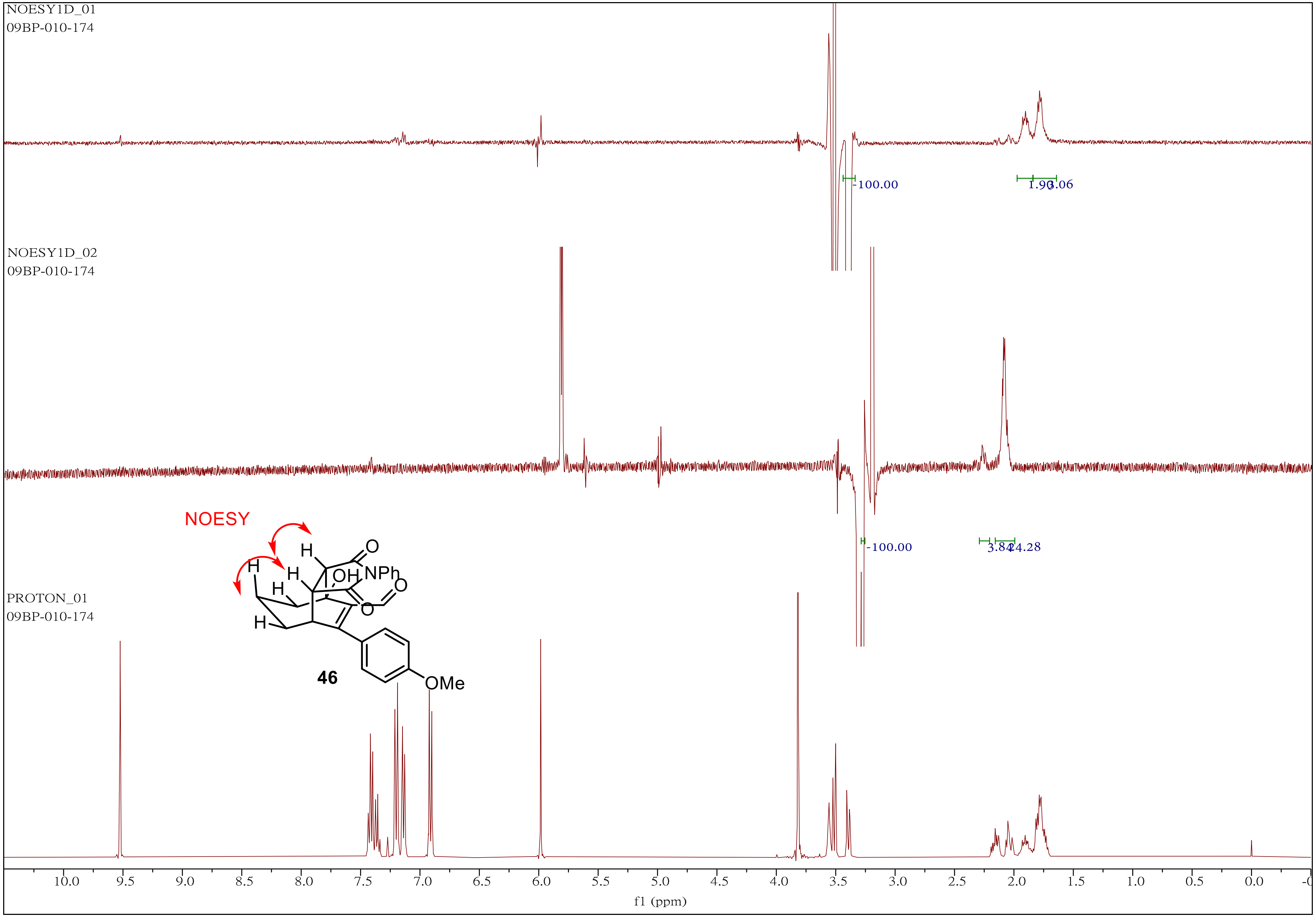


1D NOESY spectra for compound **46**

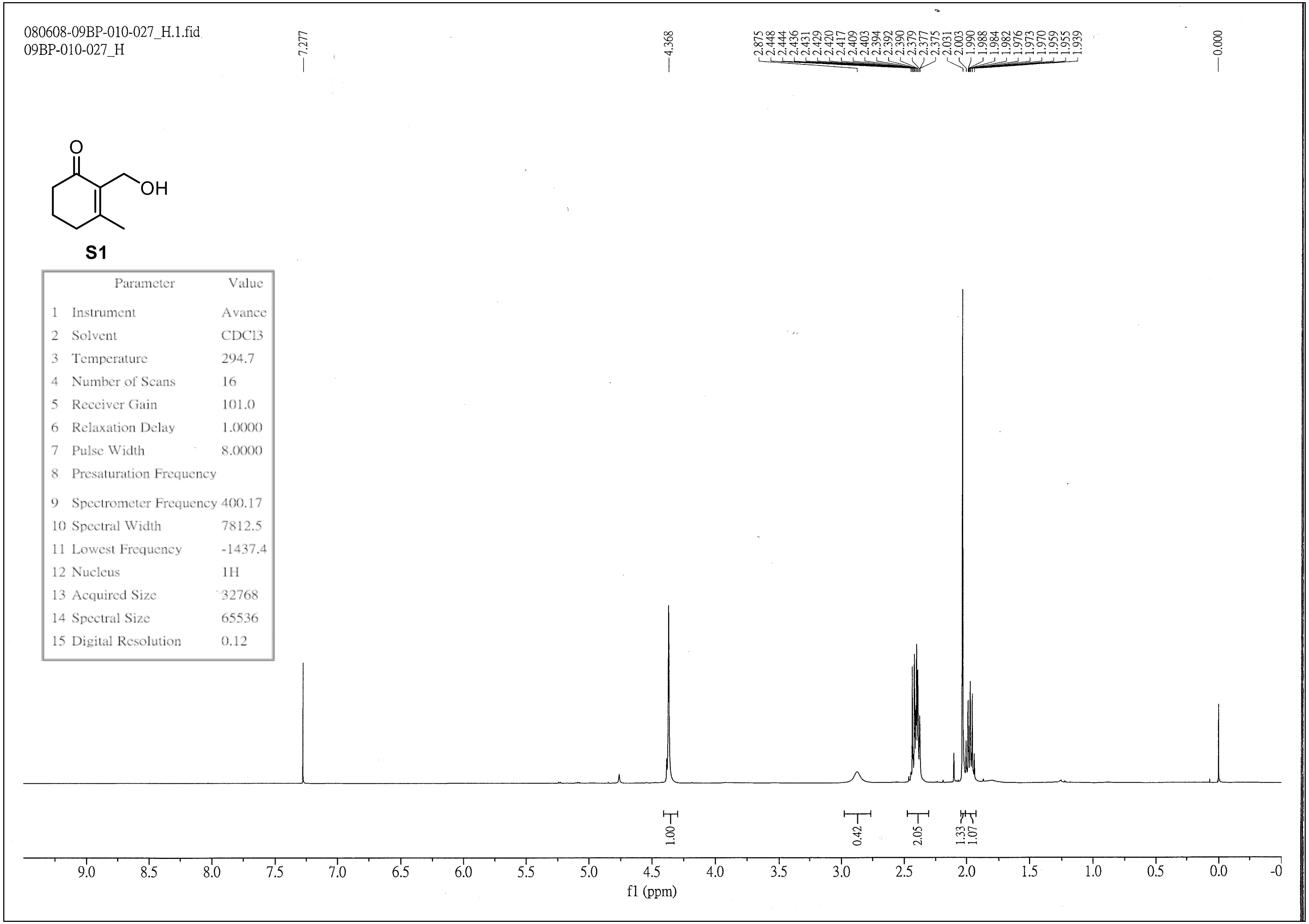


| Parameter | | Value |
|---|---|---|
| 1 | Instrument | Avance |
| 2 | Solvent | CDCl3 |
| 3 | Temperature | 294.7 |
| 4 | Number of Scans | 16 |
| 5 | Receiver Gain | 101.0 |
| 6 | Relaxation Delay | 1.0000 |
| 7 | Pulse Width | 8.0000 |
| 8 | Presaturation Frequency | |
| 9 | Spectrometer Frequency | 400.17 |
| 10 | Spectral Width | 7812.5 |
| 11 | Lowest Frequency | -1437.4 |
| 12 | Nucleus | 1H |
| 13 | Acquired Size | 32768 |
| 14 | Spectral Size | 65536 |
| 15 | Digital Resolution | 0.12 |

$^{1}$H NMR spectrum for compound **S1**

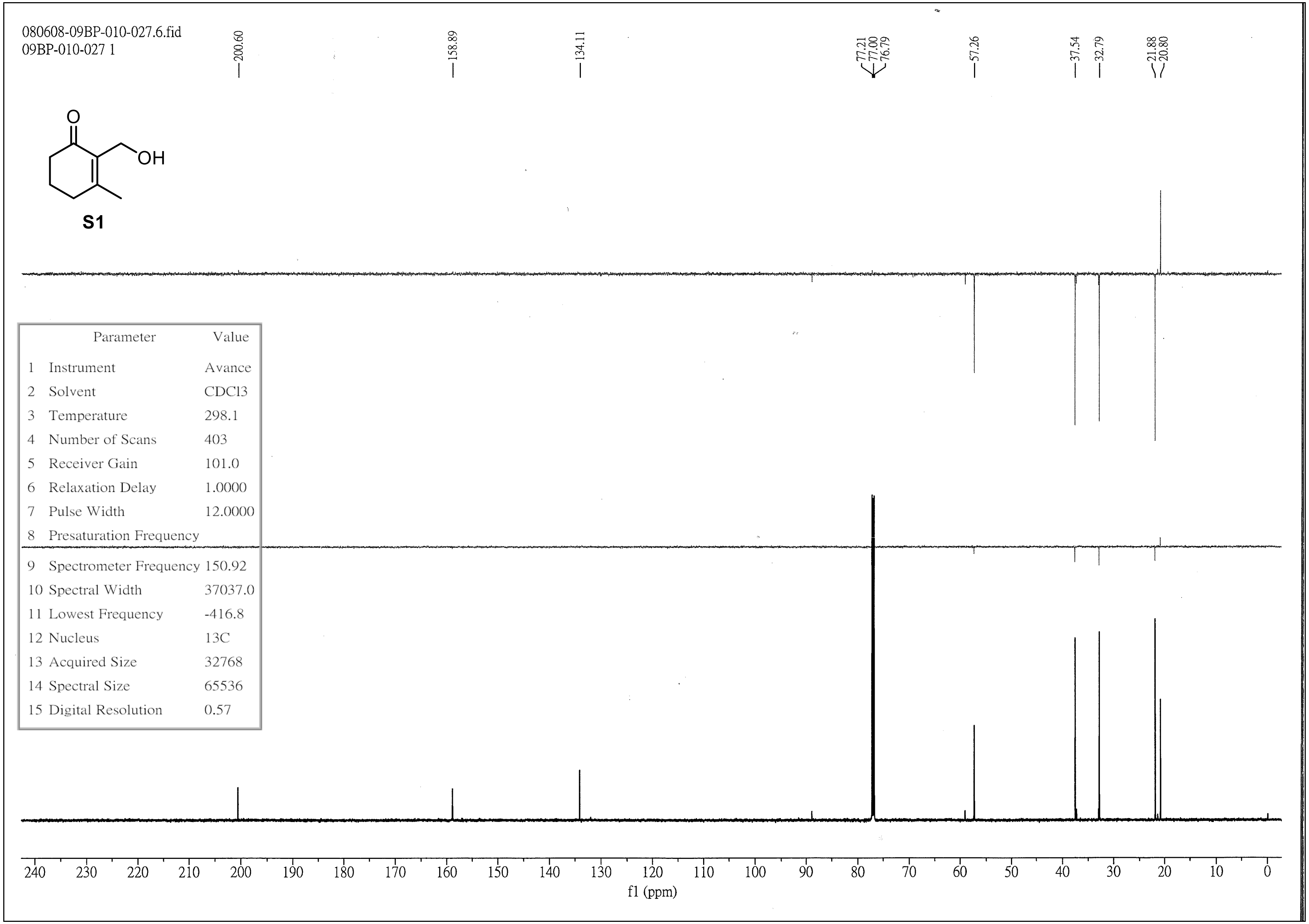


$^{13}$C NMR + DEPT spectra for compound **S1**

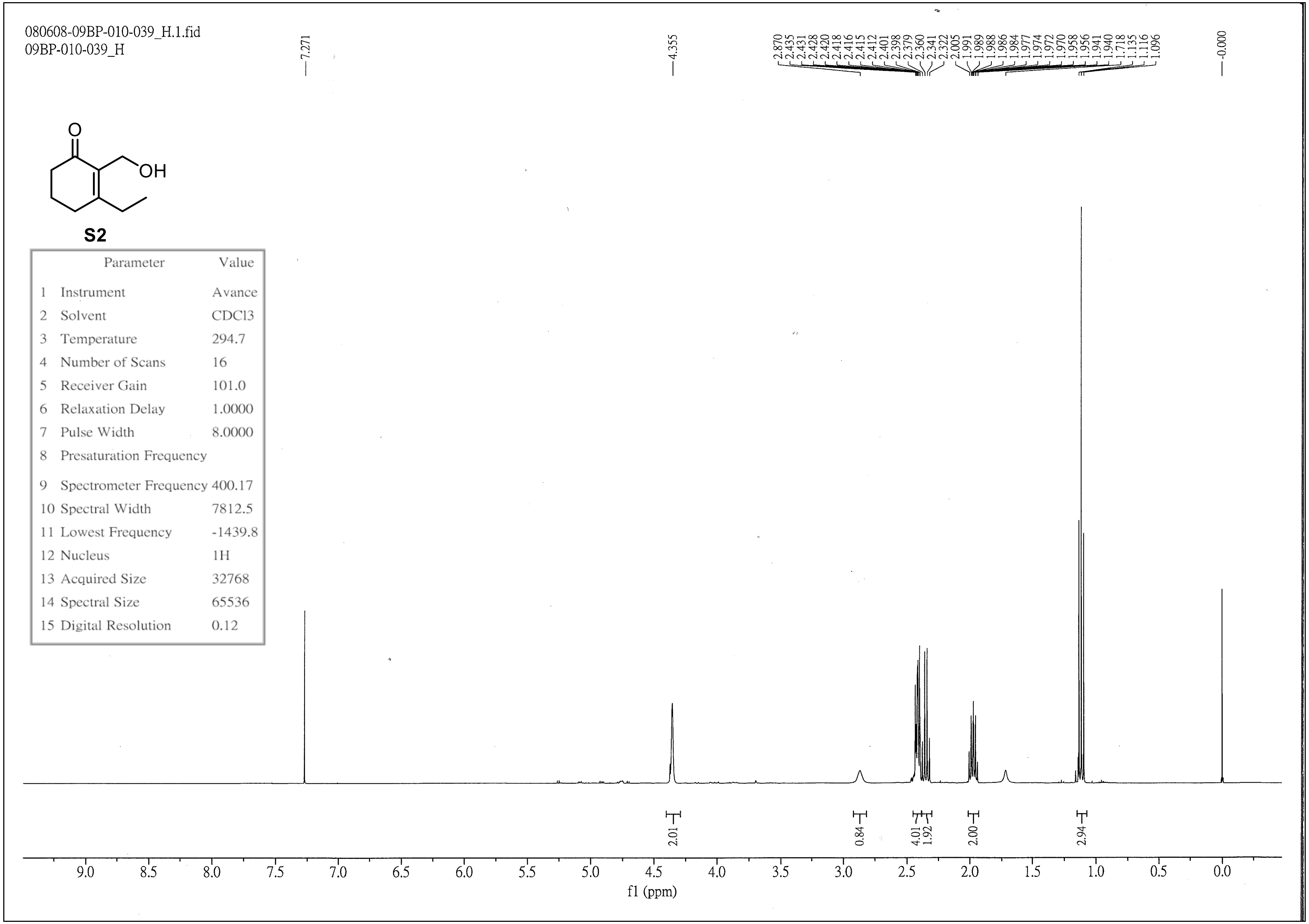


$^{1}$H NMR spectrum for compound **S2**

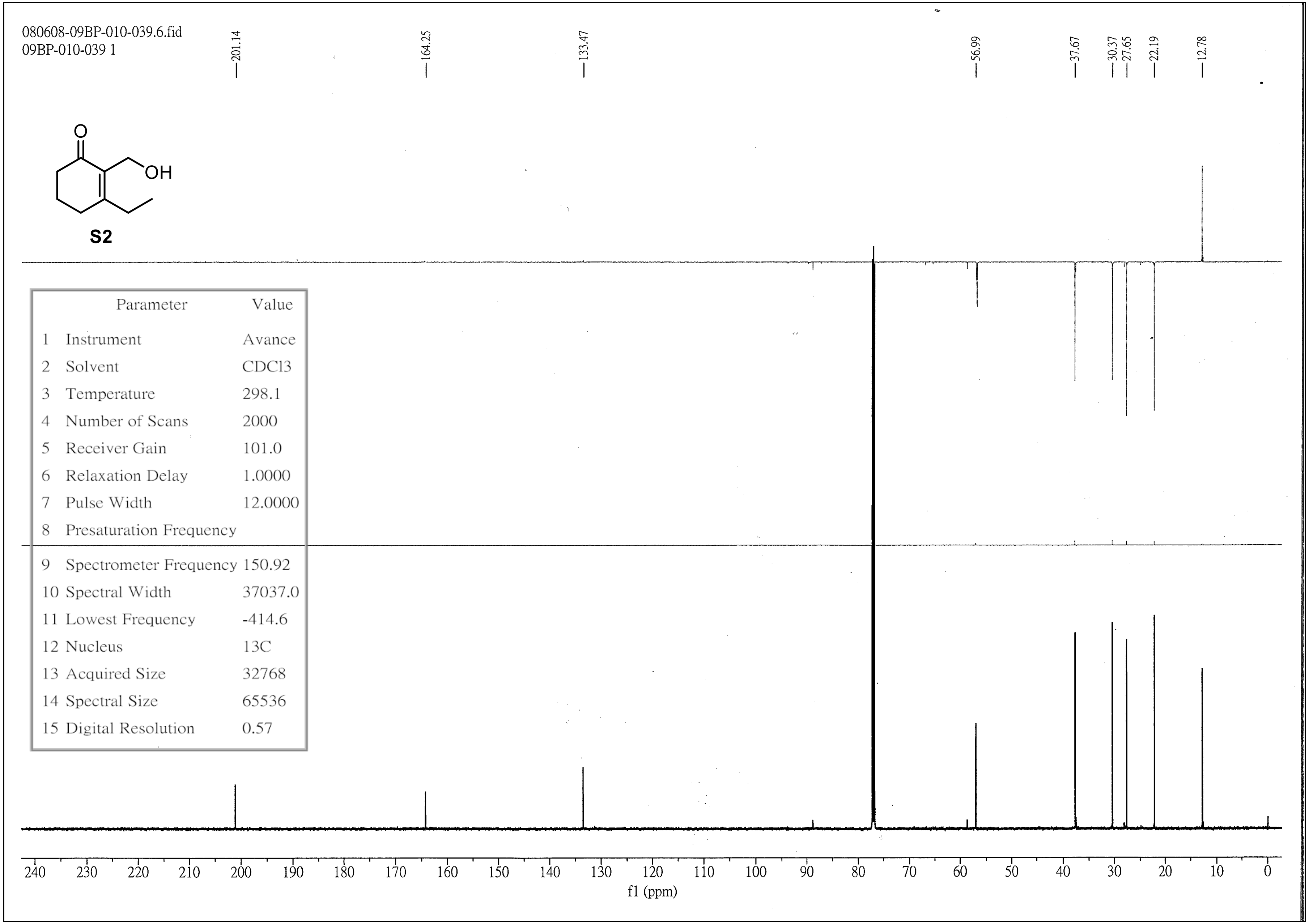


$^{13}$C NMR + DEPT spectra for compound **S2**

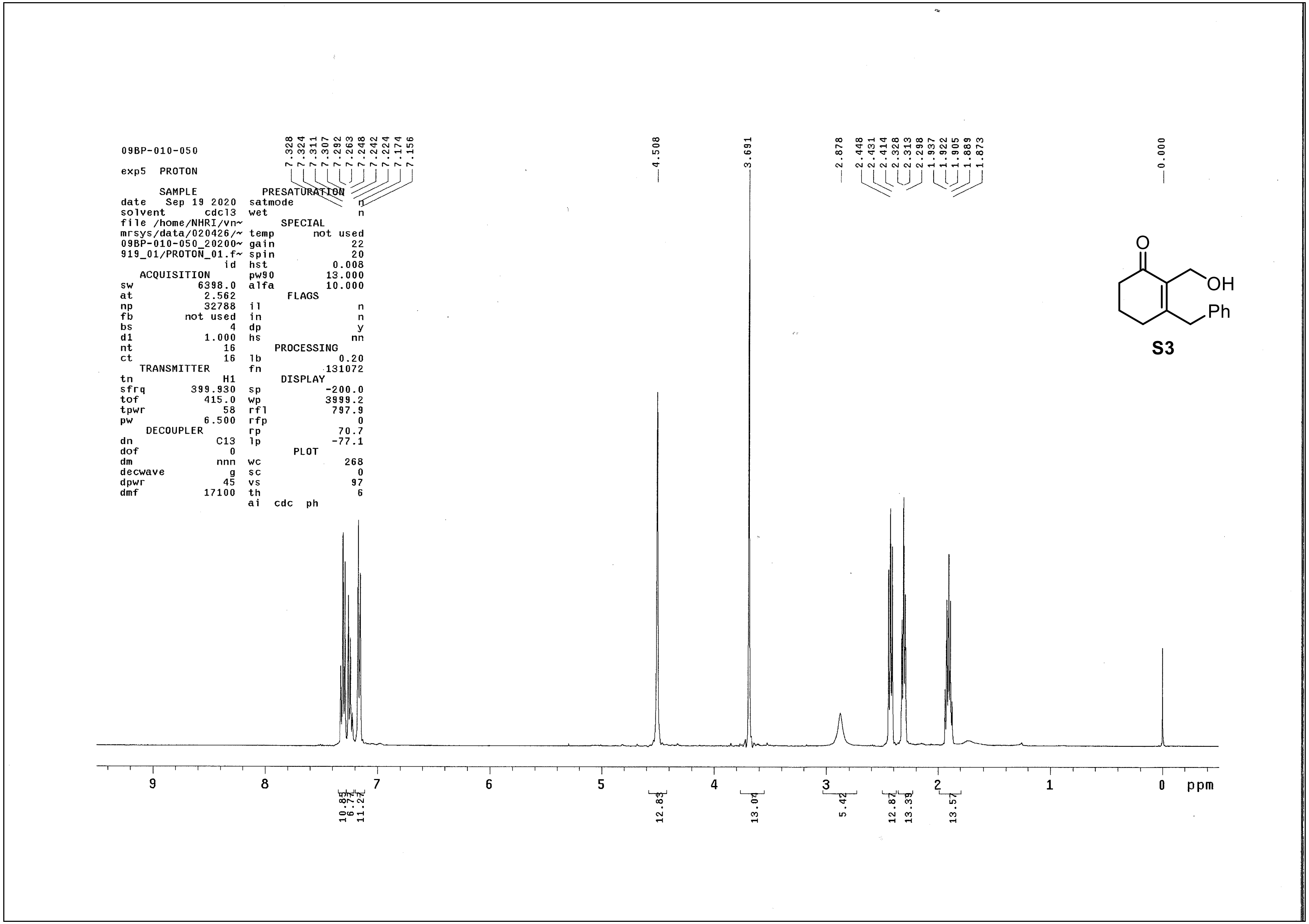


$^{1}$H NMR spectrum for compound **S3**

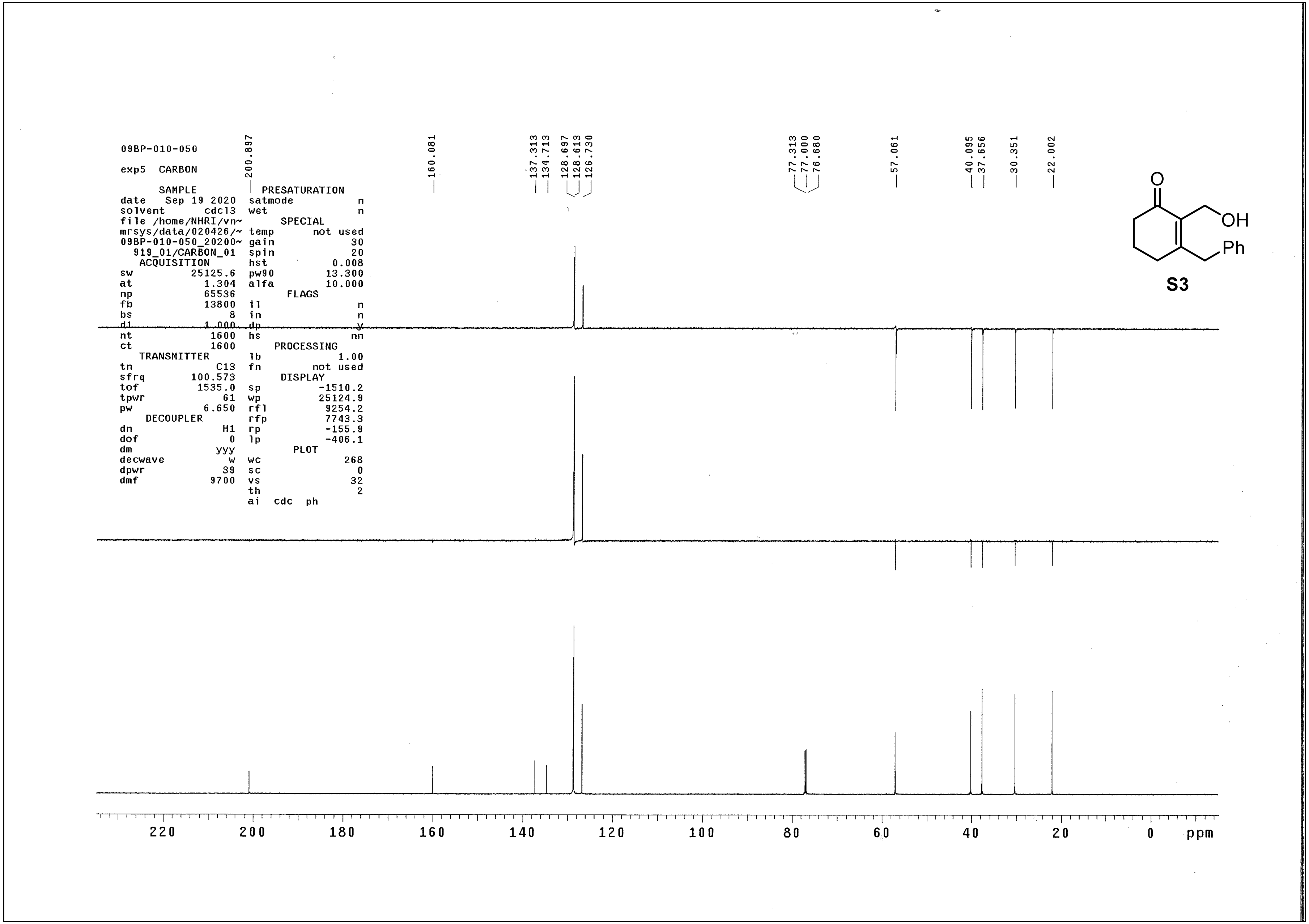


$^{13}$C NMR + DEPT spectra for compound **S3**

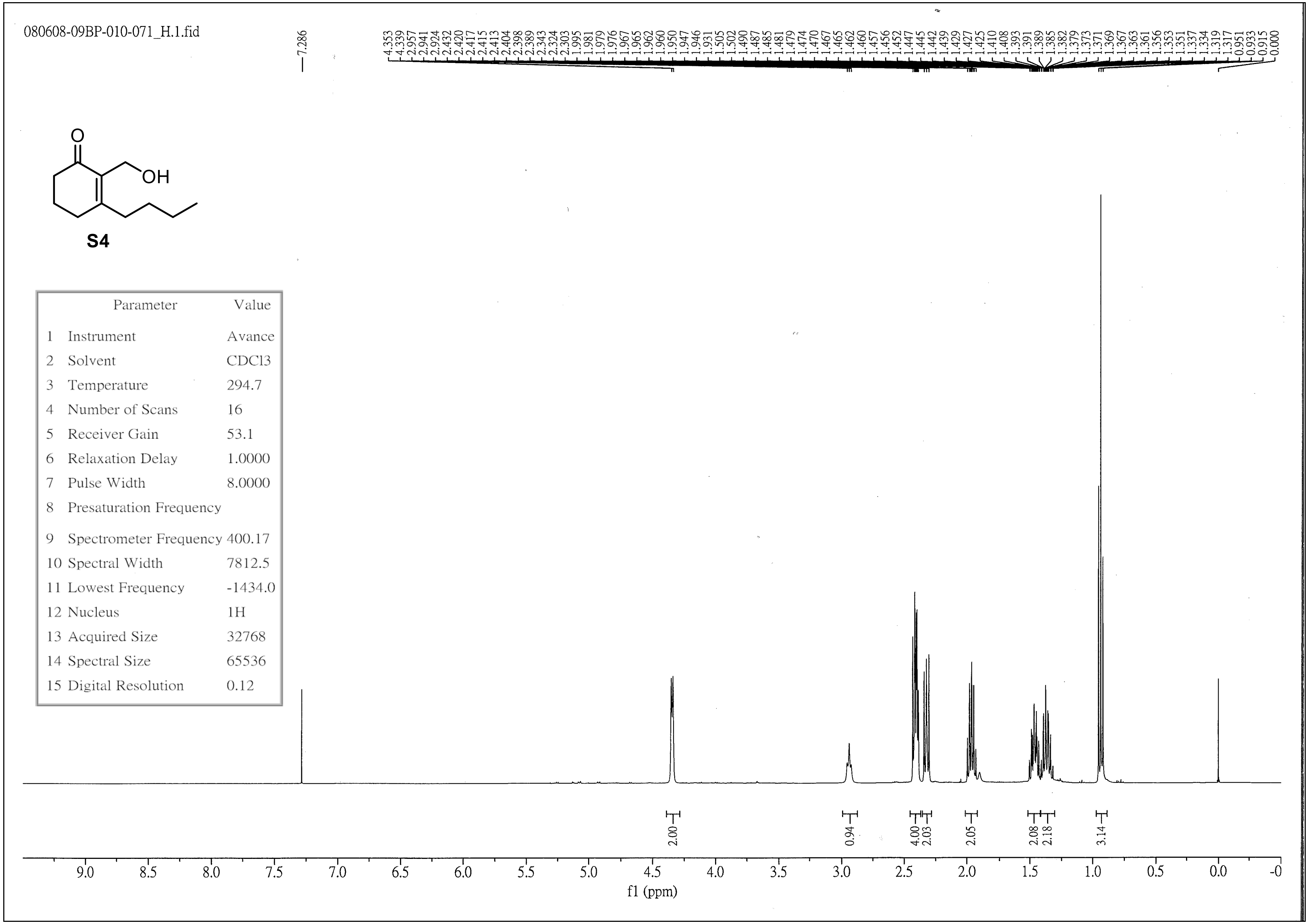


$^{1}$H NMR spectrum for compound **S4**

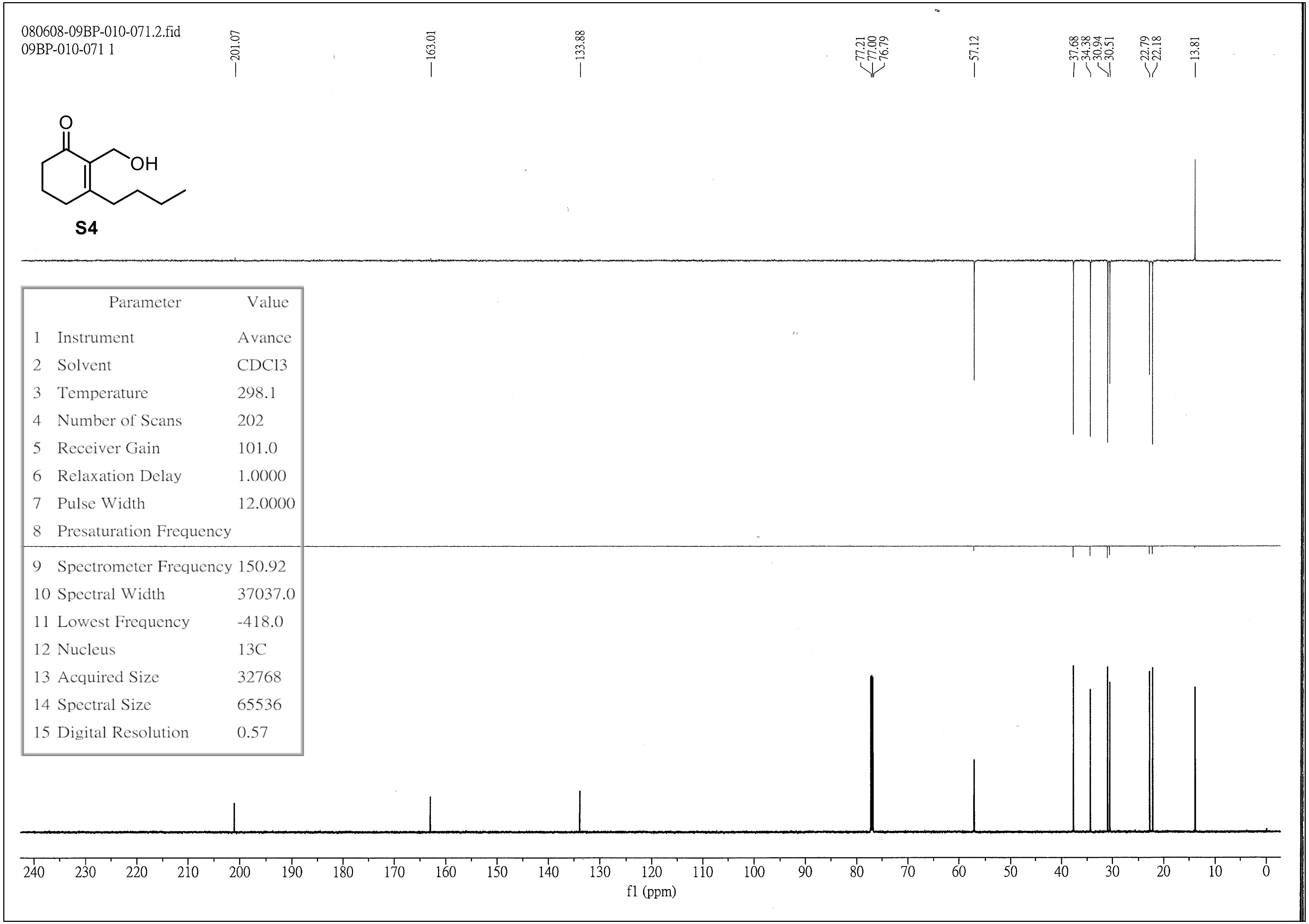


$^{13}$C NMR + DEPT spectra for compound **S4**

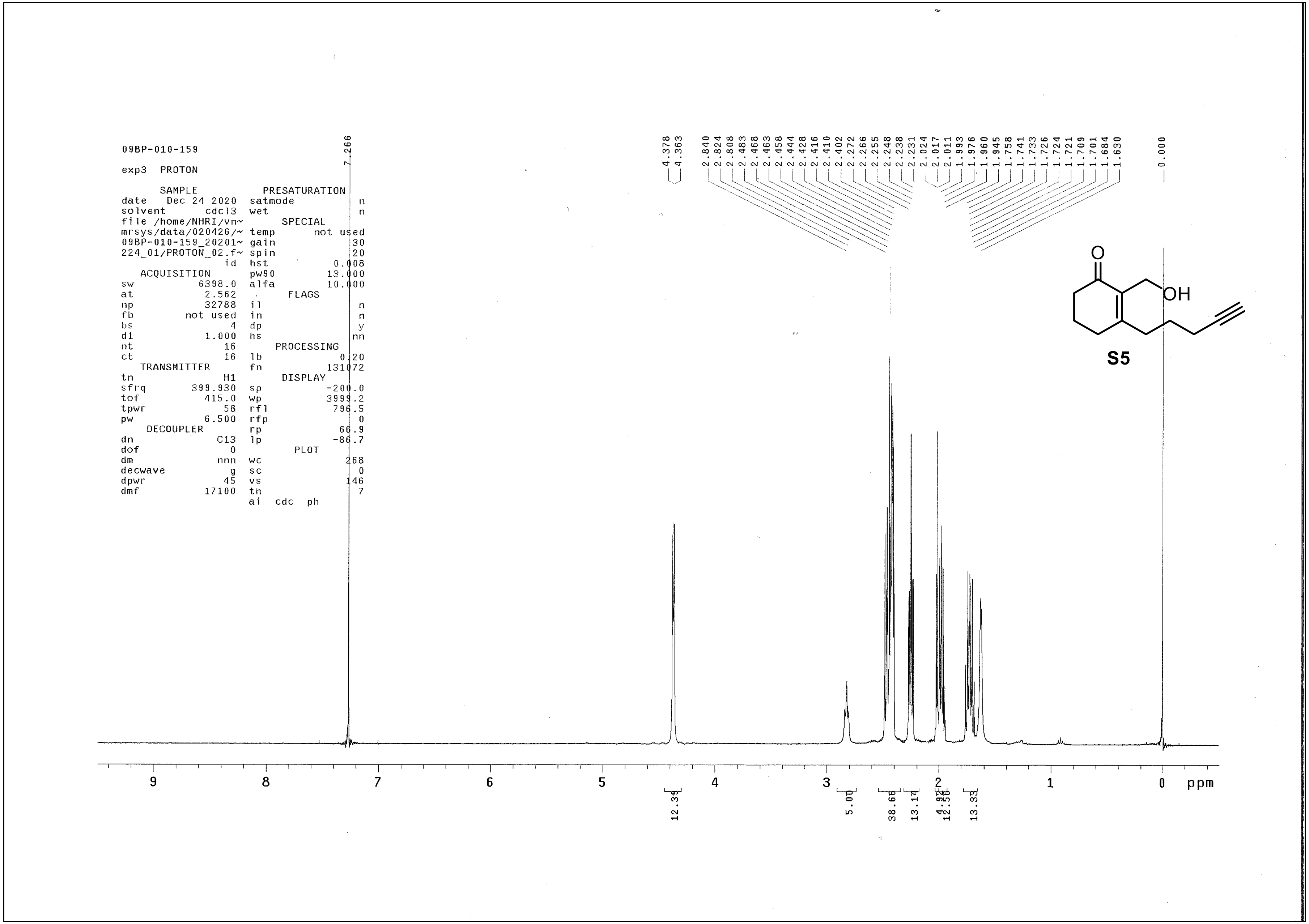


$^{1}$H NMR spectrum for compound **S5**

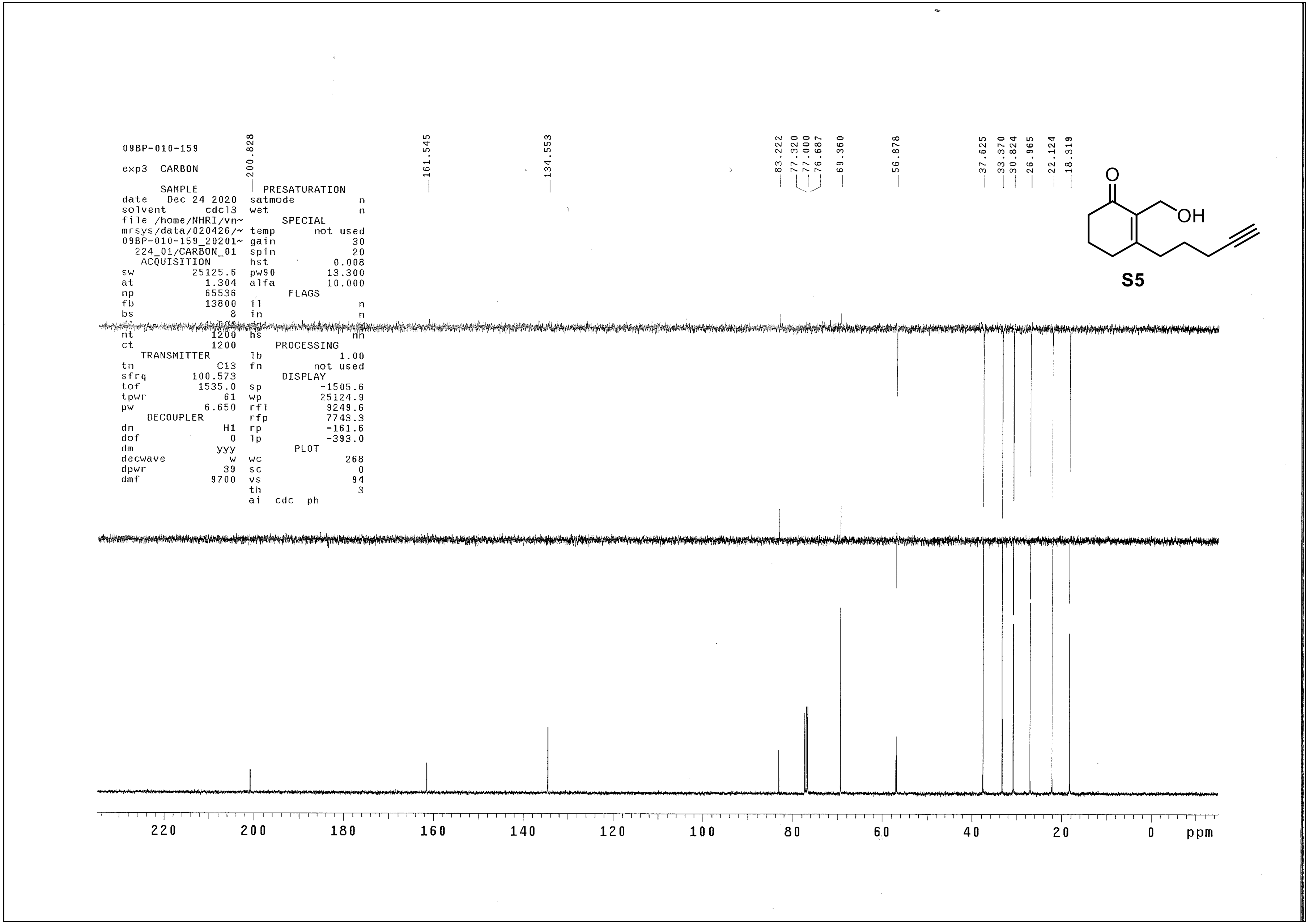


$^{13}$C NMR + DEPT spectra for compound **S5**

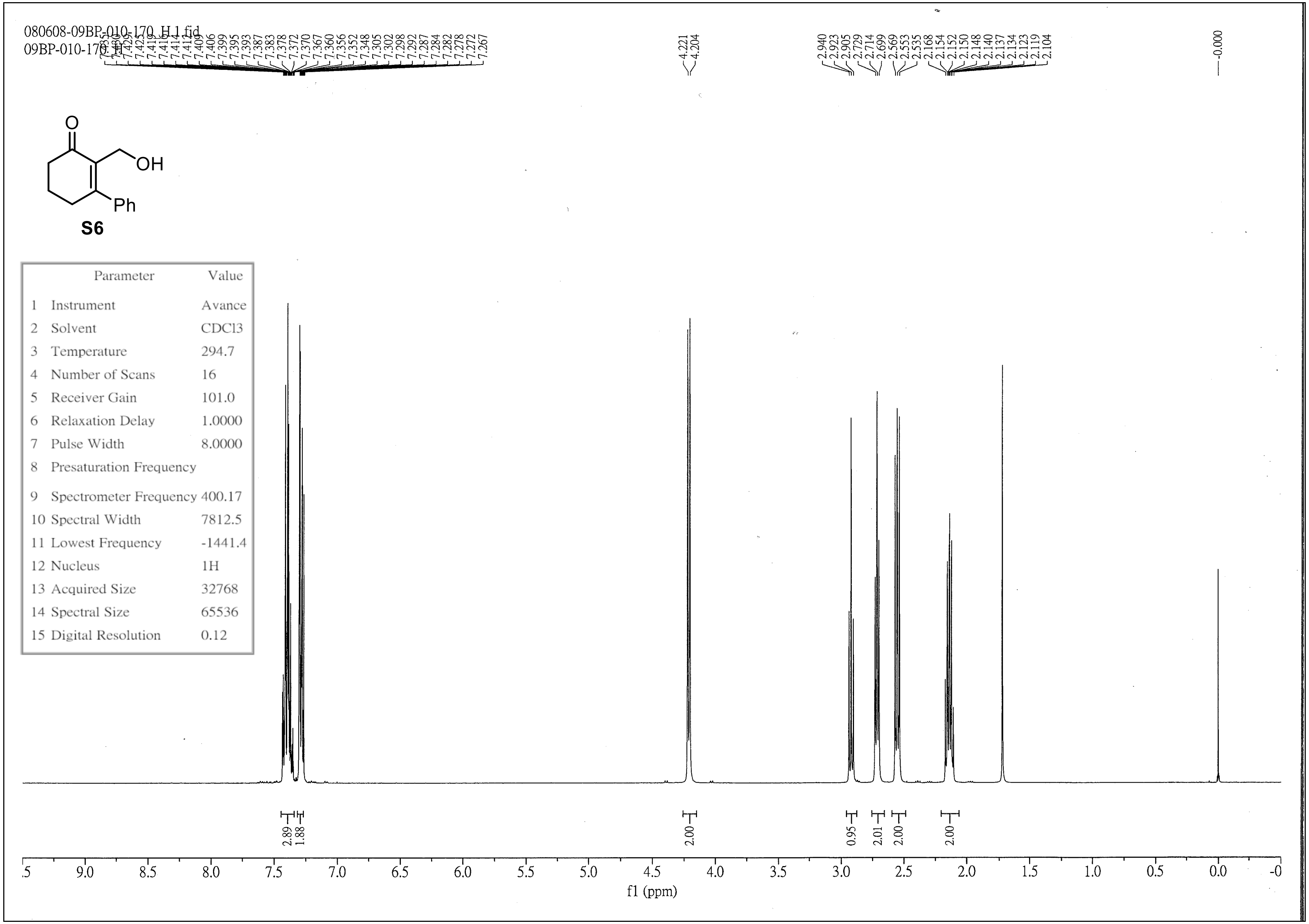


$^{1}$H NMR spectrum for compound **S6**

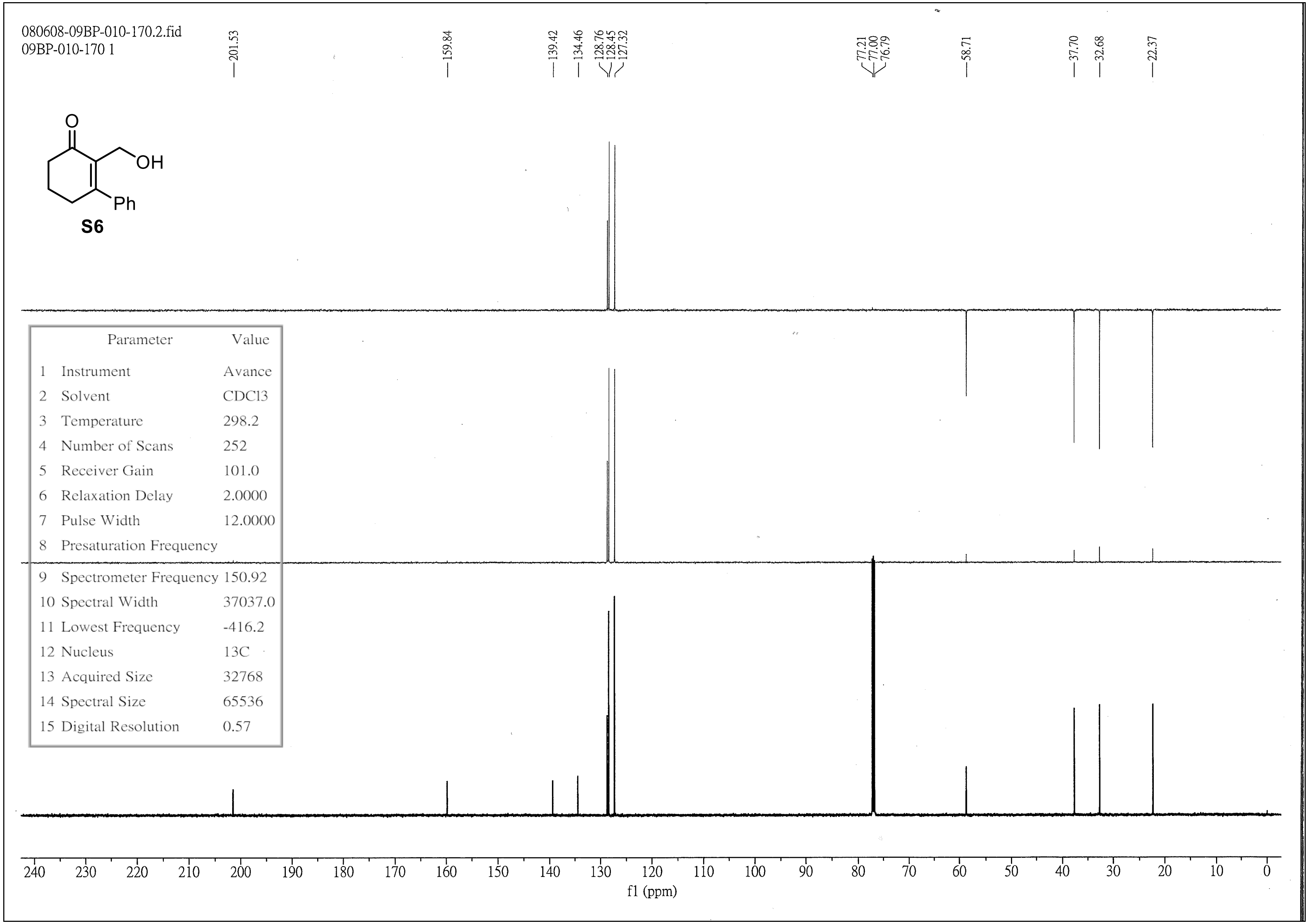


$^{13}$C NMR + DEPT spectra for compound **S6**

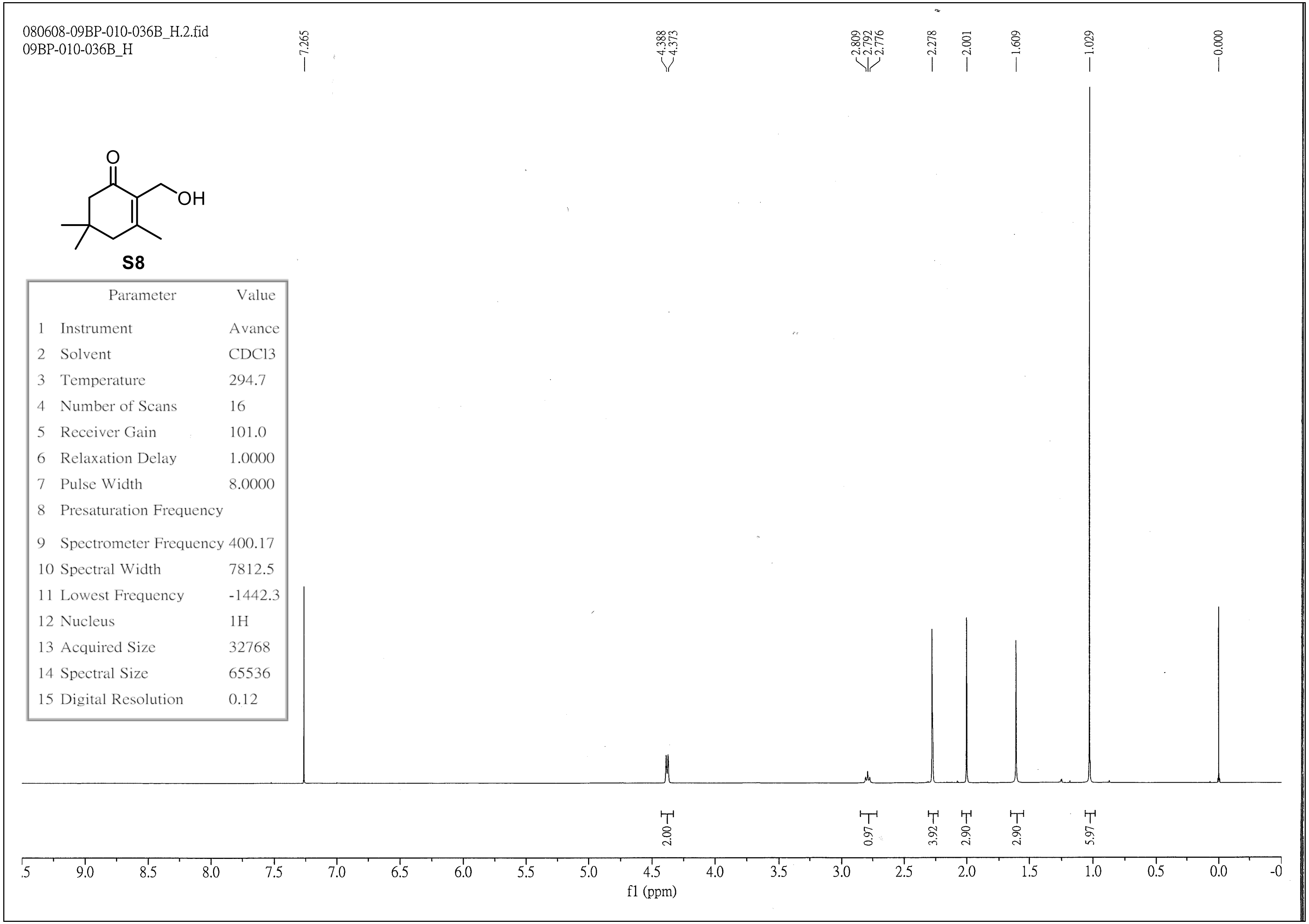


| | Parameter | Value |
|---|---|---|
| 1 | Instrument | Avance |
| 2 | Solvent | CDCl3 |
| 3 | Temperature | 294.7 |
| 4 | Number of Scans | 16 |
| 5 | Receiver Gain | 101.0 |
| 6 | Relaxation Delay | 1.0000 |
| 7 | Pulse Width | 8.0000 |
| 8 | Presaturation Frequency | |
| 9 | Spectrometer Frequency | 400.17 |
| 10 | Spectral Width | 7812.5 |
| 11 | Lowest Frequency | -1442.3 |
| 12 | Nucleus | 1H |
| 13 | Acquired Size | 32768 |
| 14 | Spectral Size | 65536 |
| 15 | Digital Resolution | 0.12 |

$^{1}$H NMR spectrum for compound **S8**

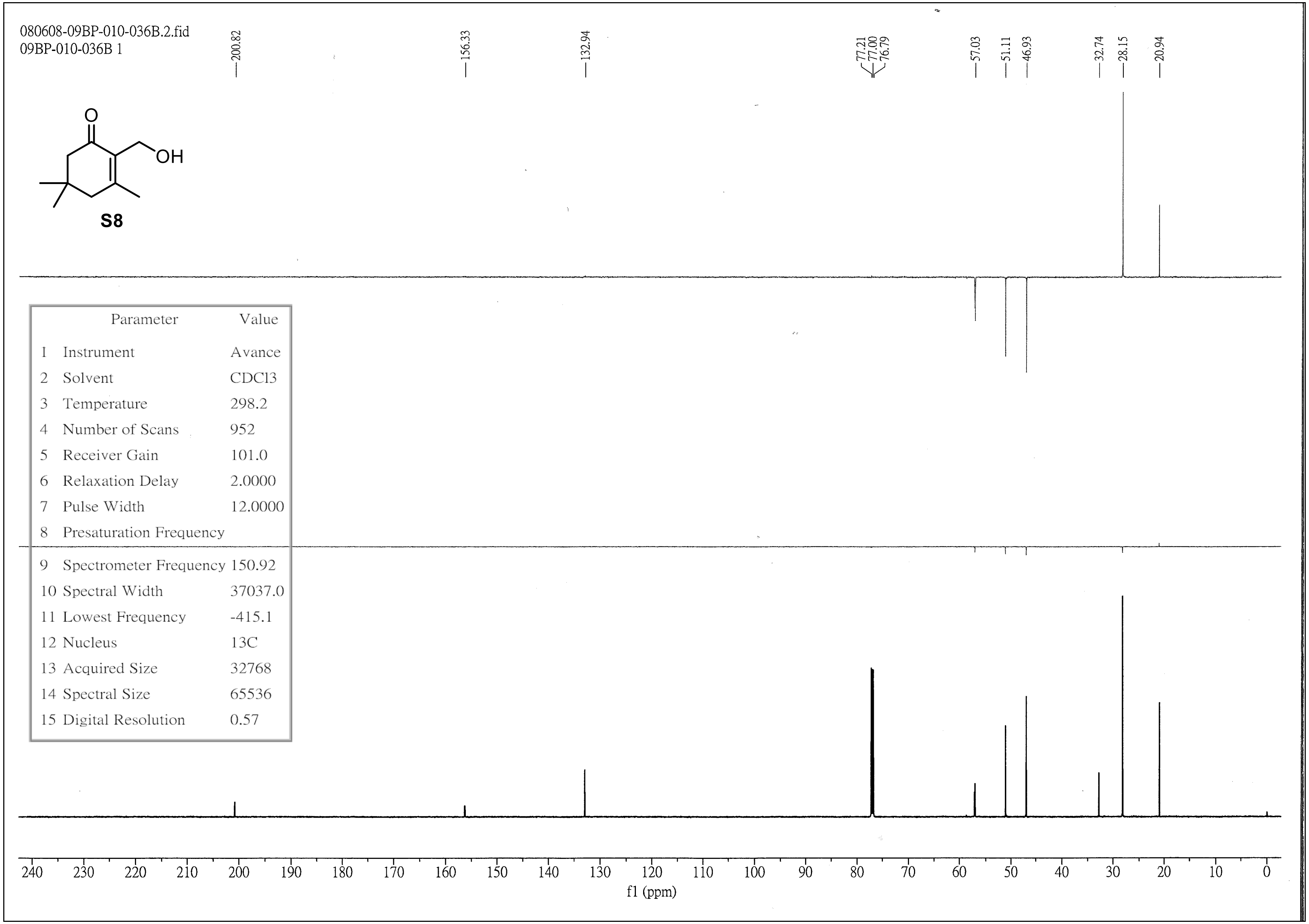


$^{13}$C NMR + DEPT spectra for compound **S8**

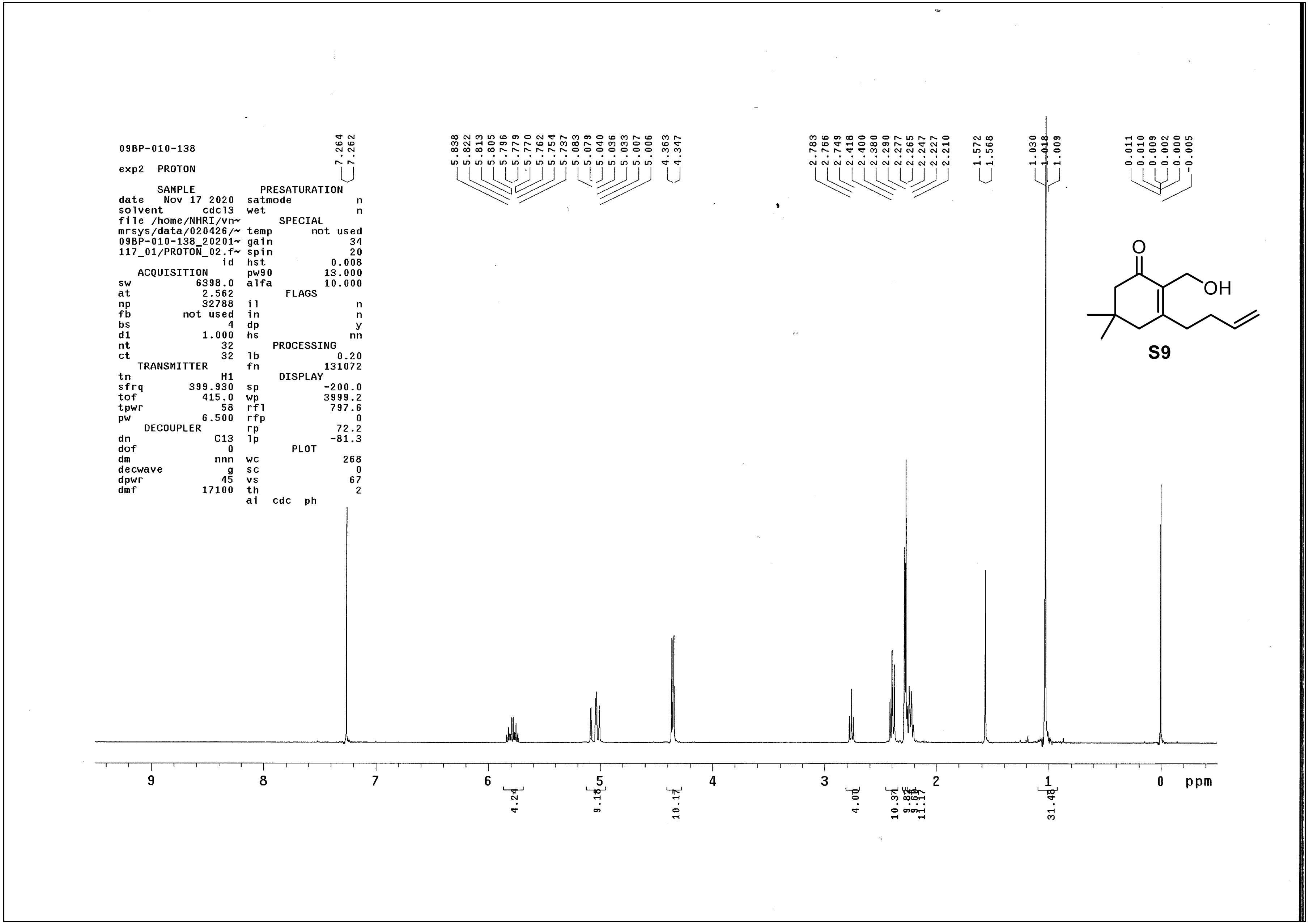

$^{1}$H NMR spectrum for compound **S9**

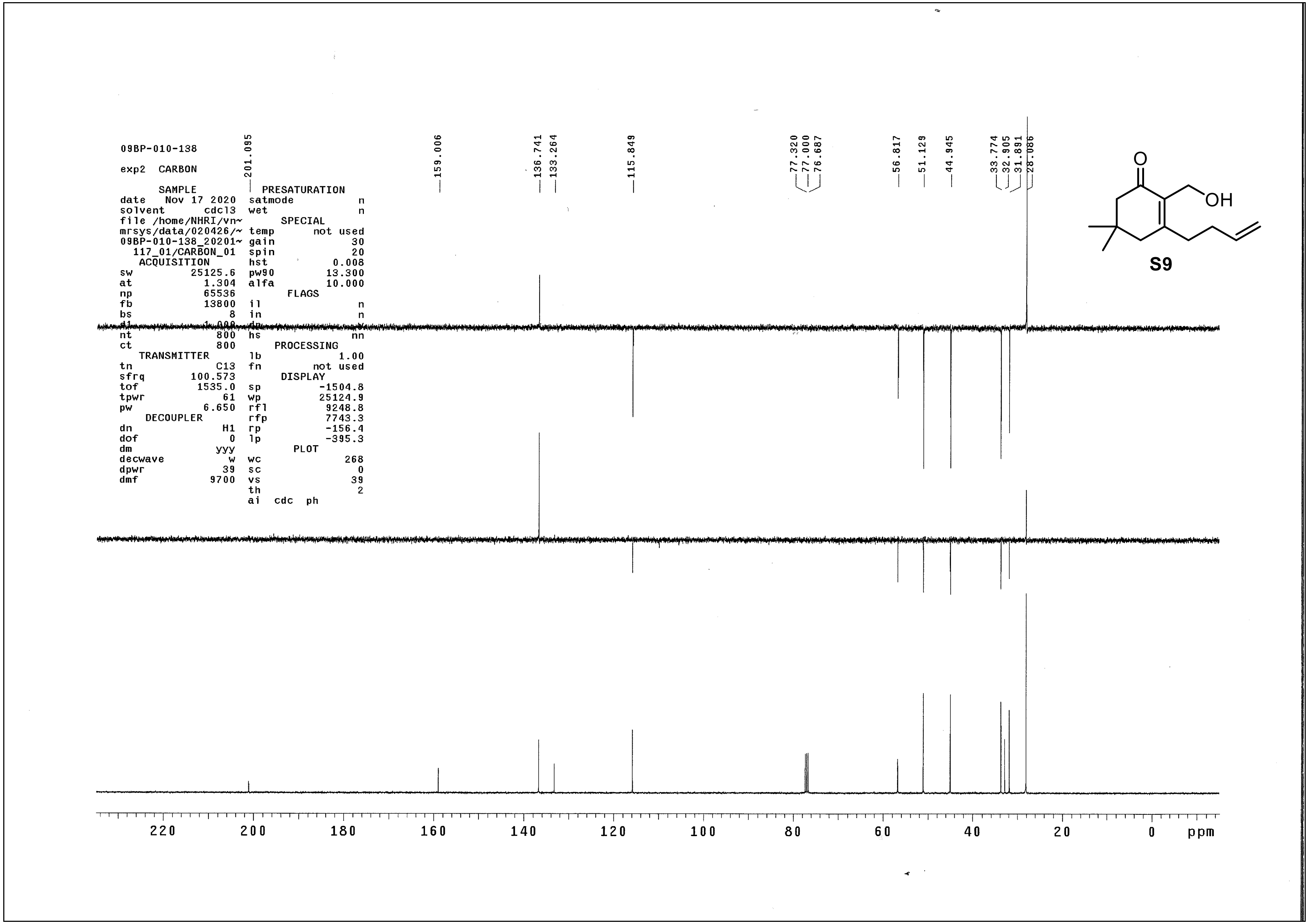


$^{13}$C NMR + DEPT spectra for compound **S9**

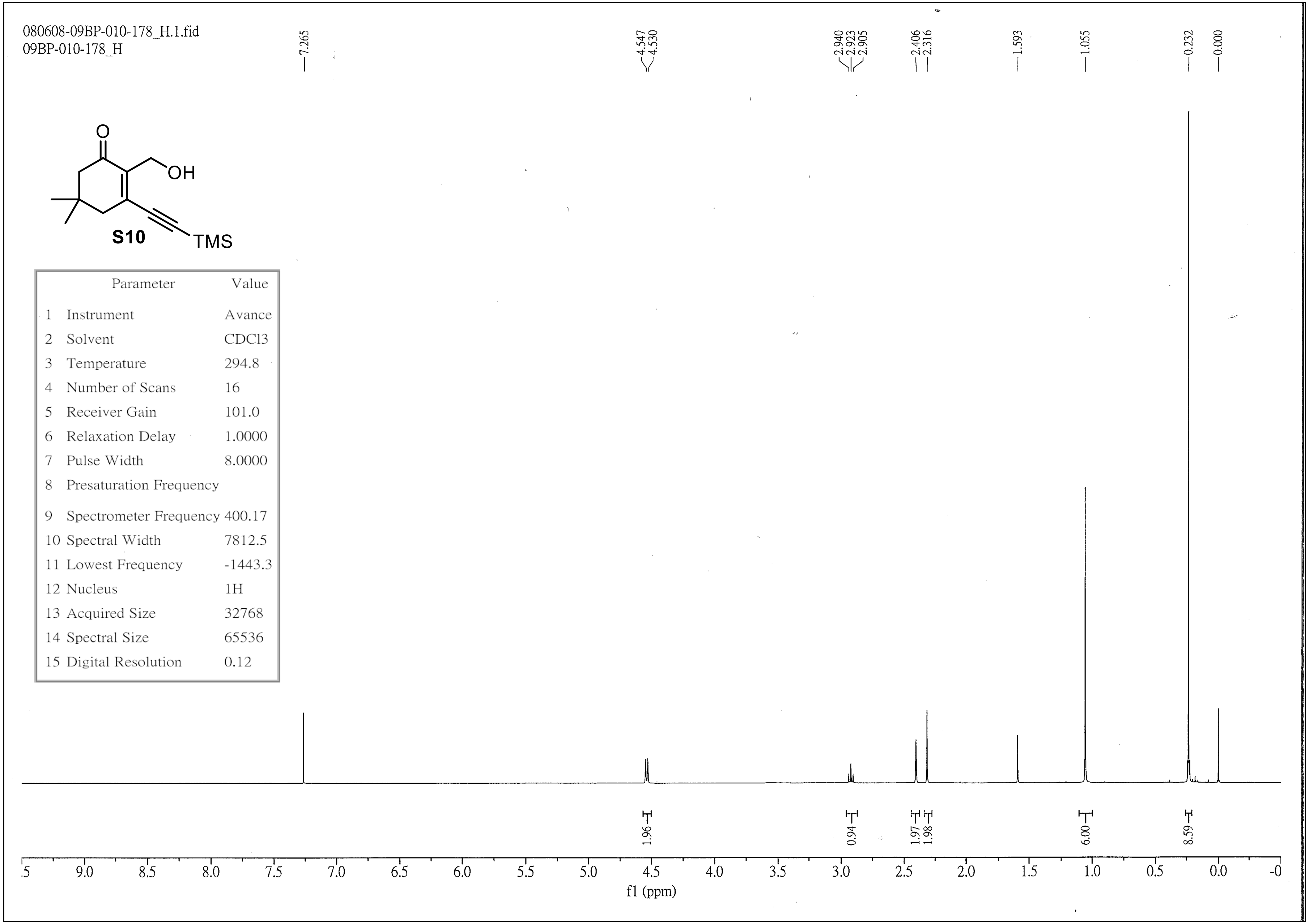


$^{1}$H NMR spectrum for compound **S10**

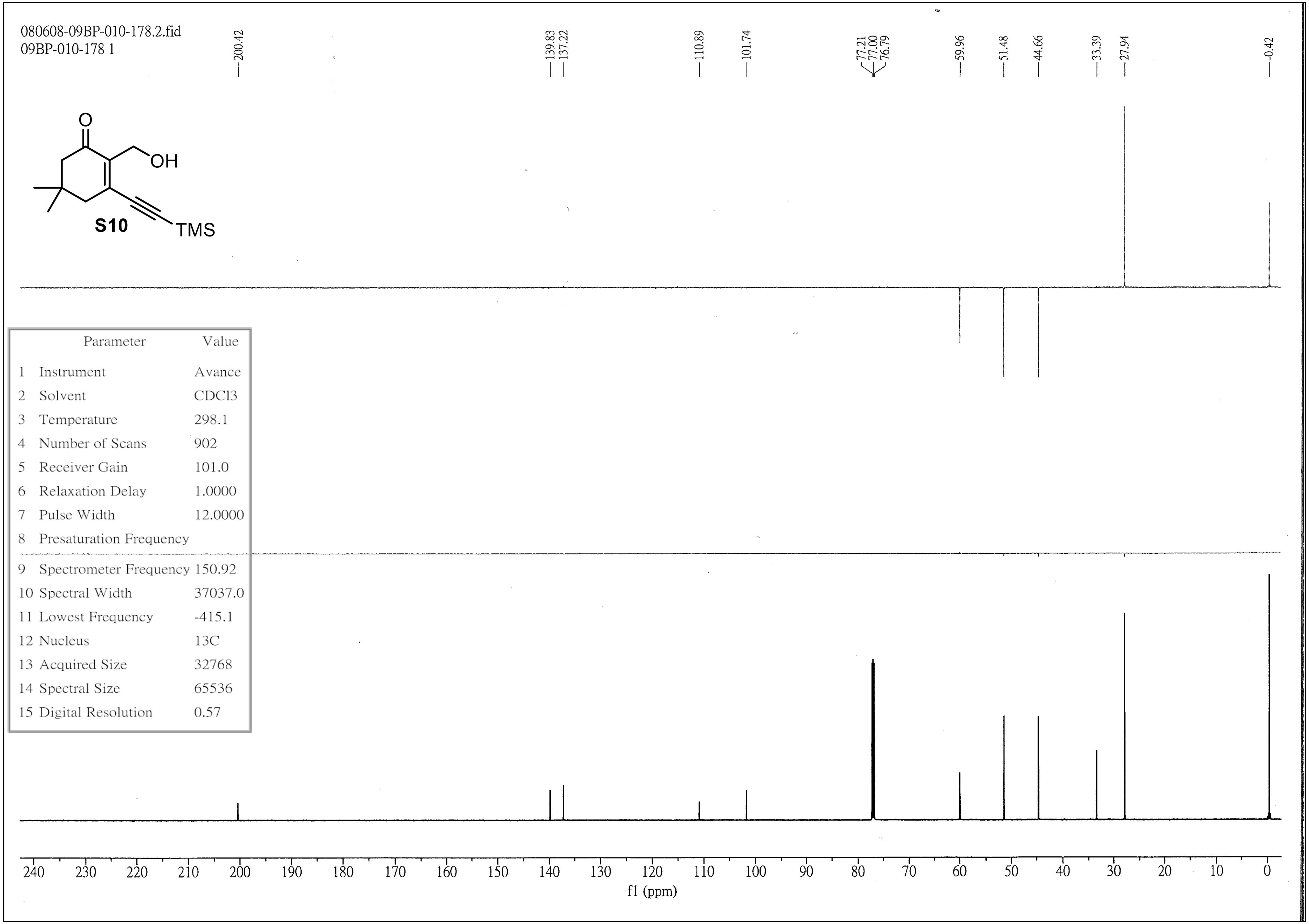


$^{13}$C NMR + DEPT spectra for compound **S10**

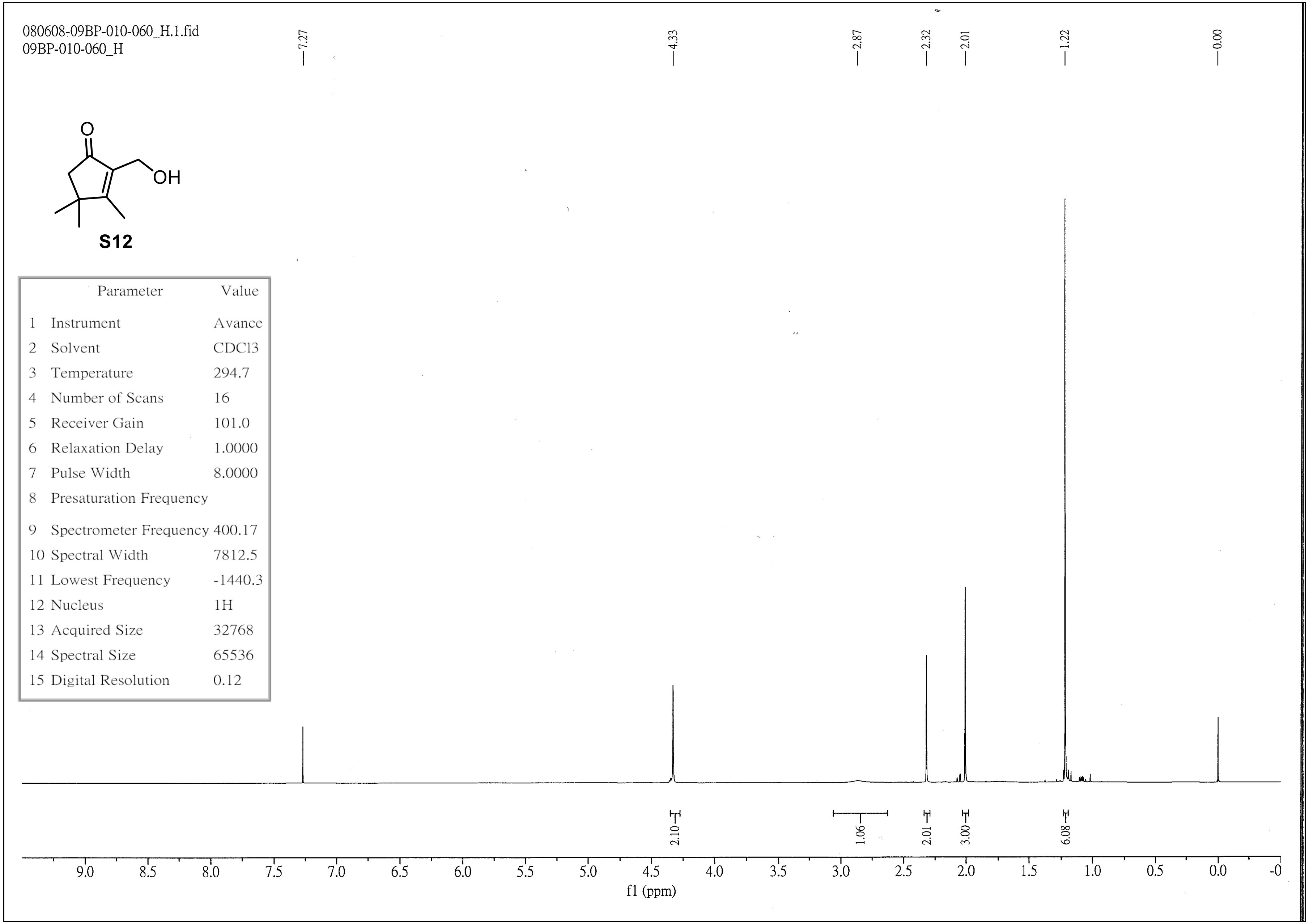


$^1$H NMR spectrum for compound **S12**

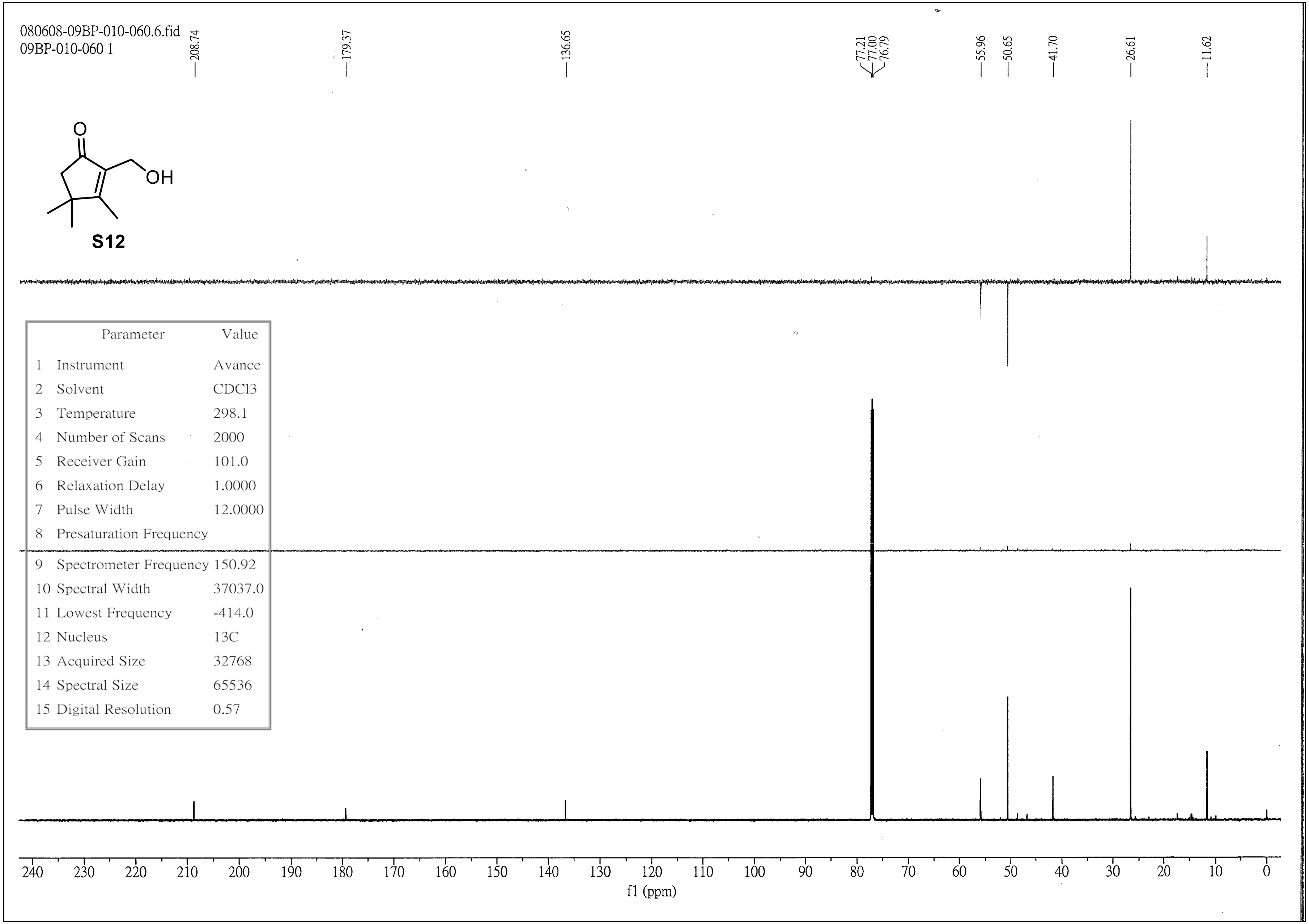


$^{13}$C NMR + DEPT spectra for compound **S12**

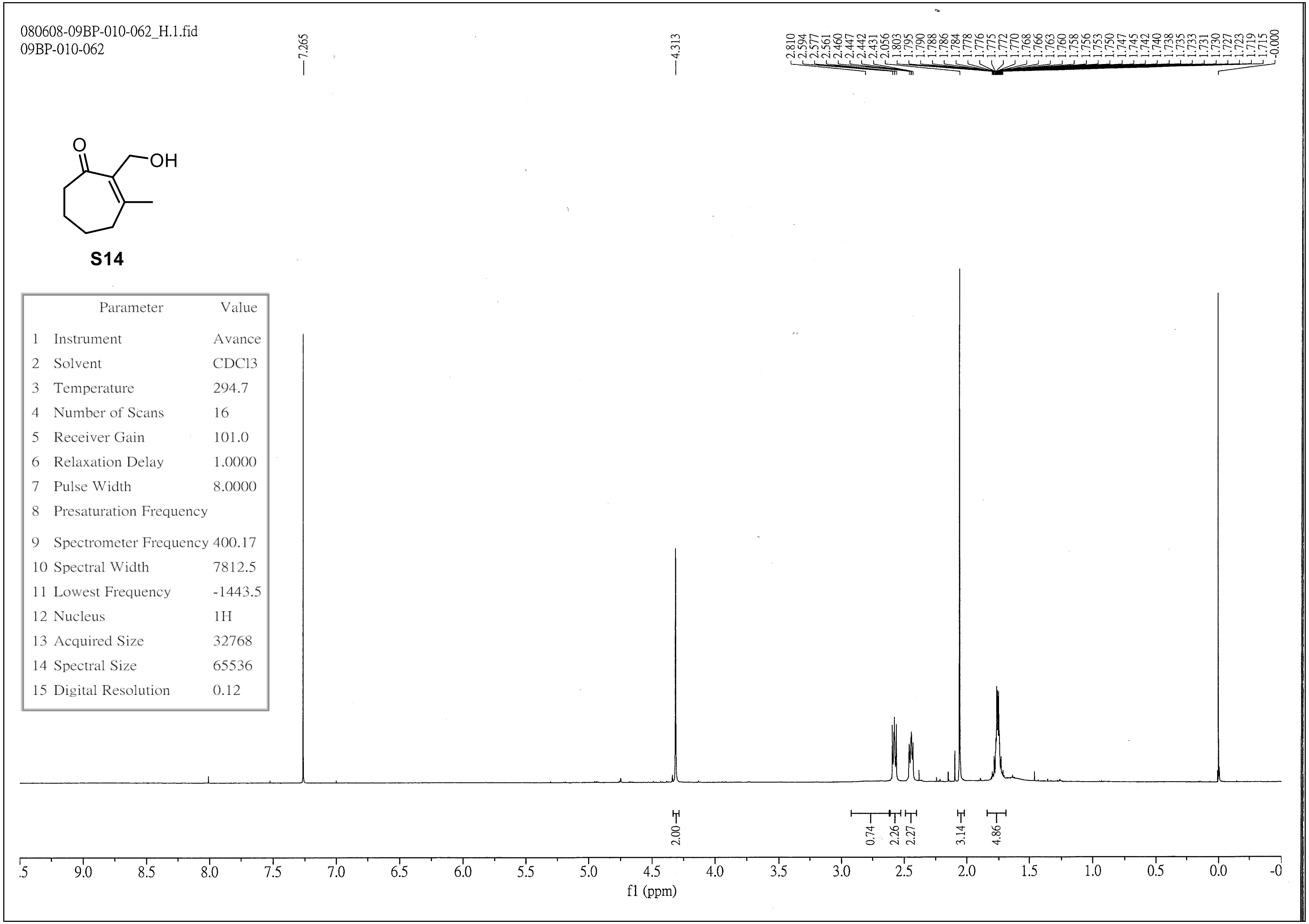


$^{1}$H NMR spectrum for compound **S14**

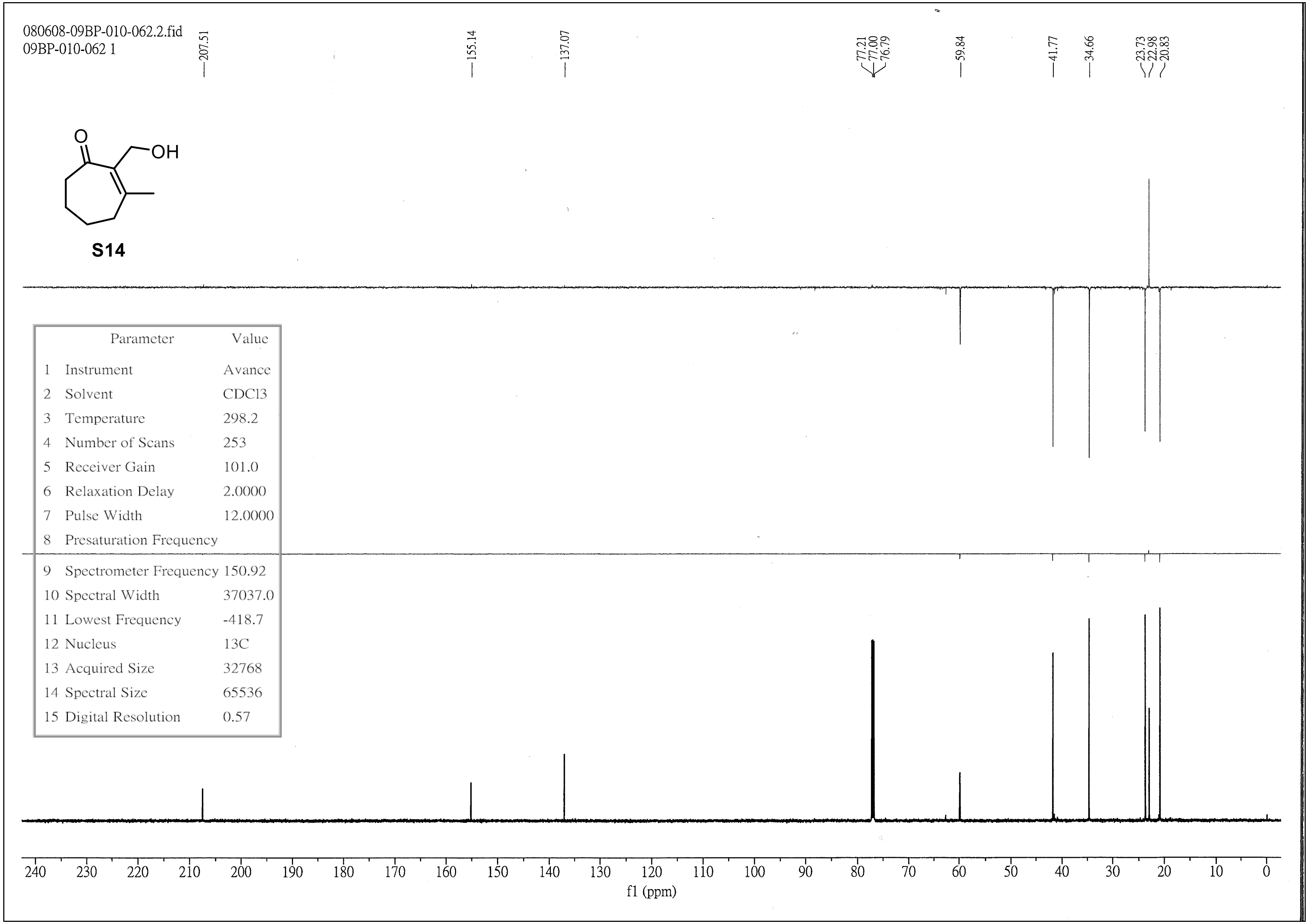


$^{13}C$ NMR + DEPT spectra for compound **S14**

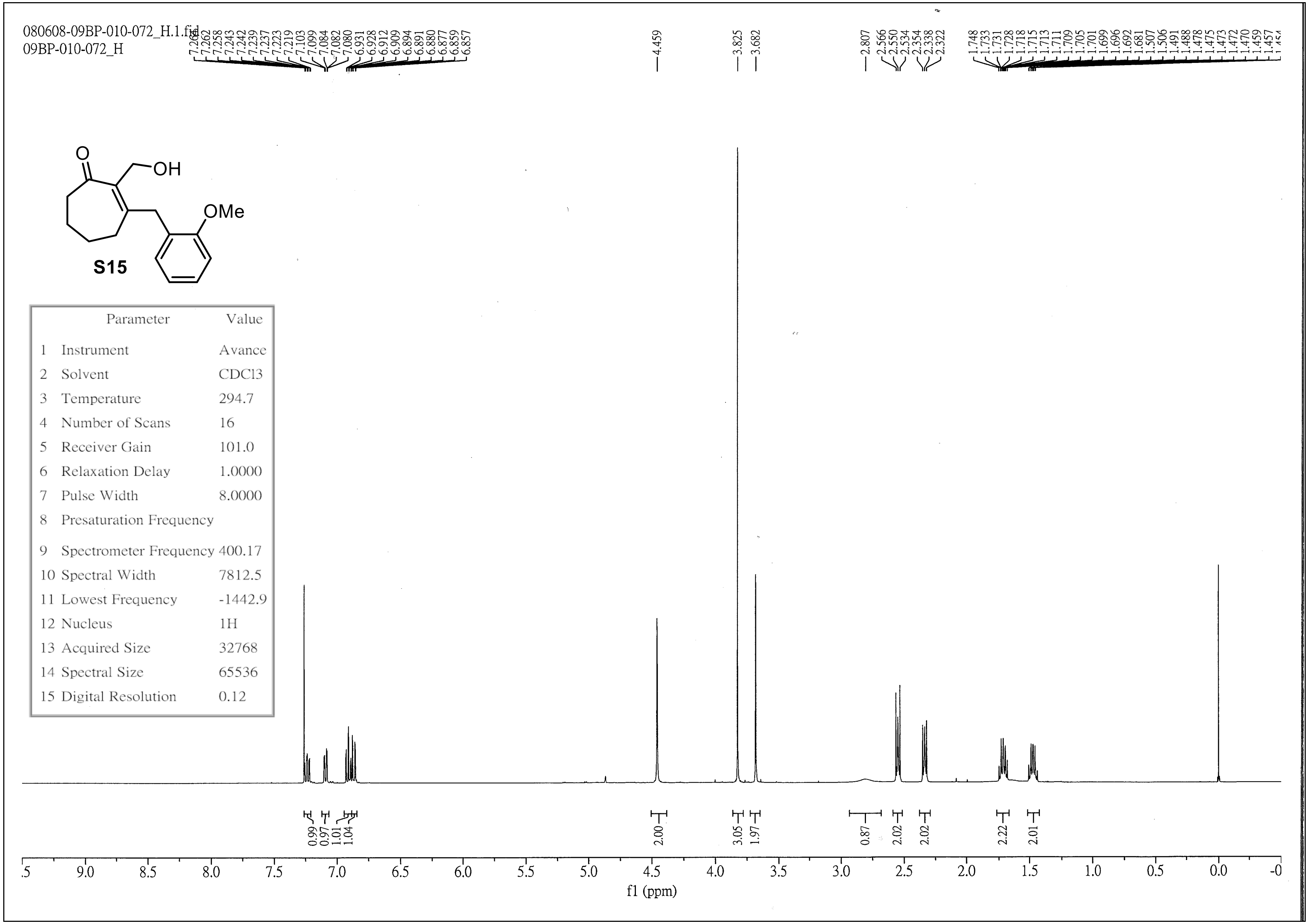

$^{1}$H NMR spectrum for compound **S15**

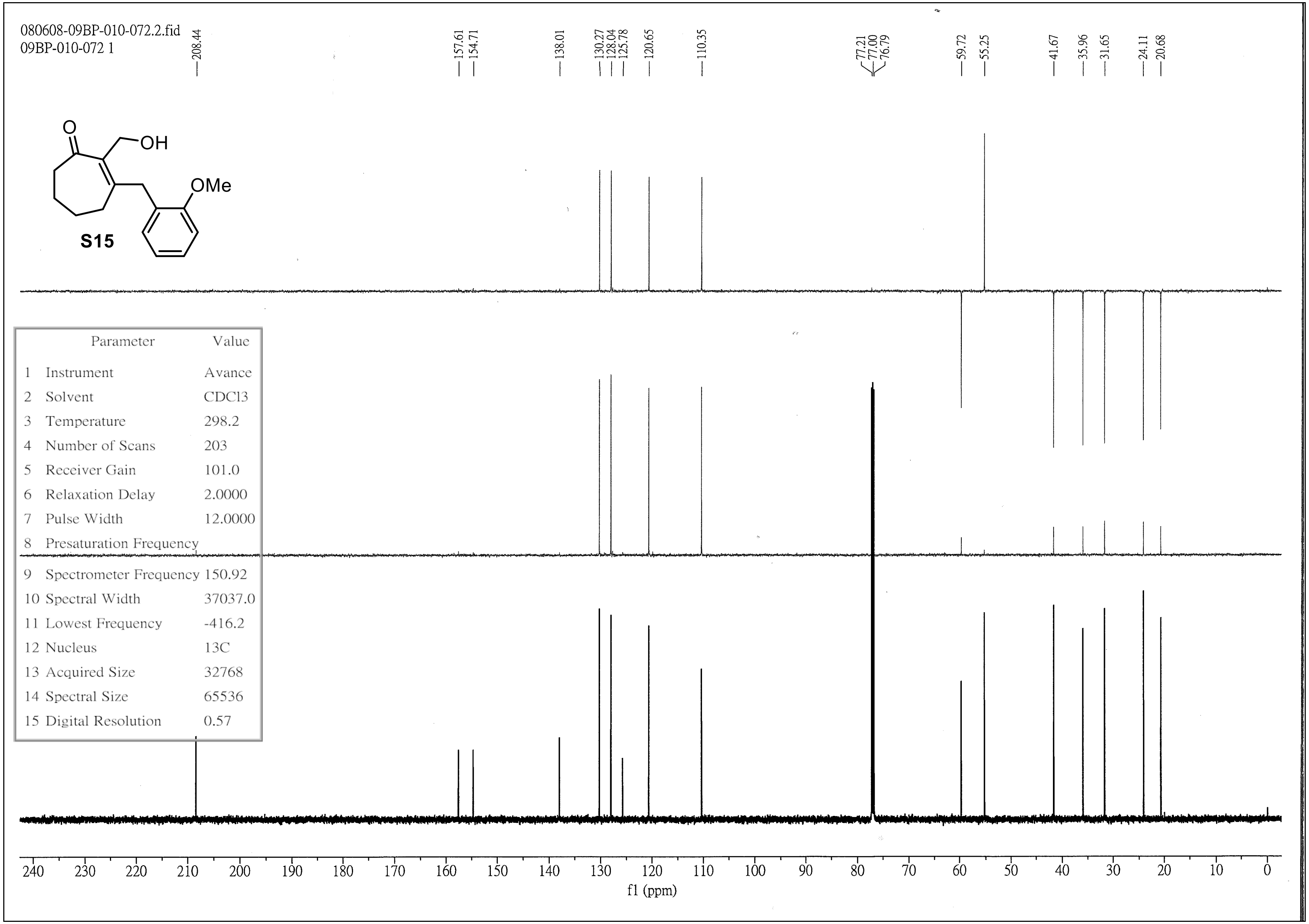


$^{13}$C NMR + DEPT spectra for compound **S15**

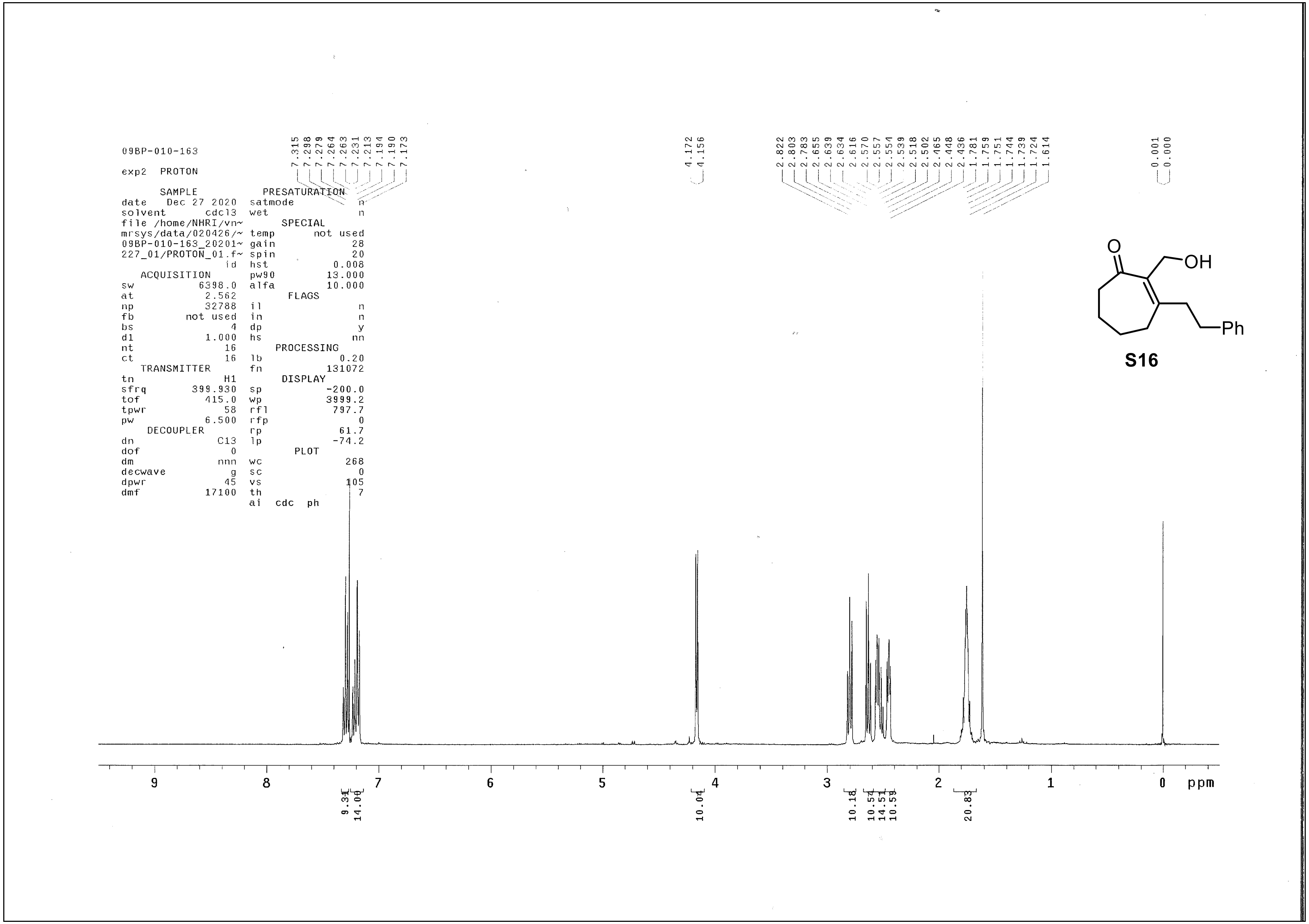


$^{1}$H NMR spectrum for compound **S16**

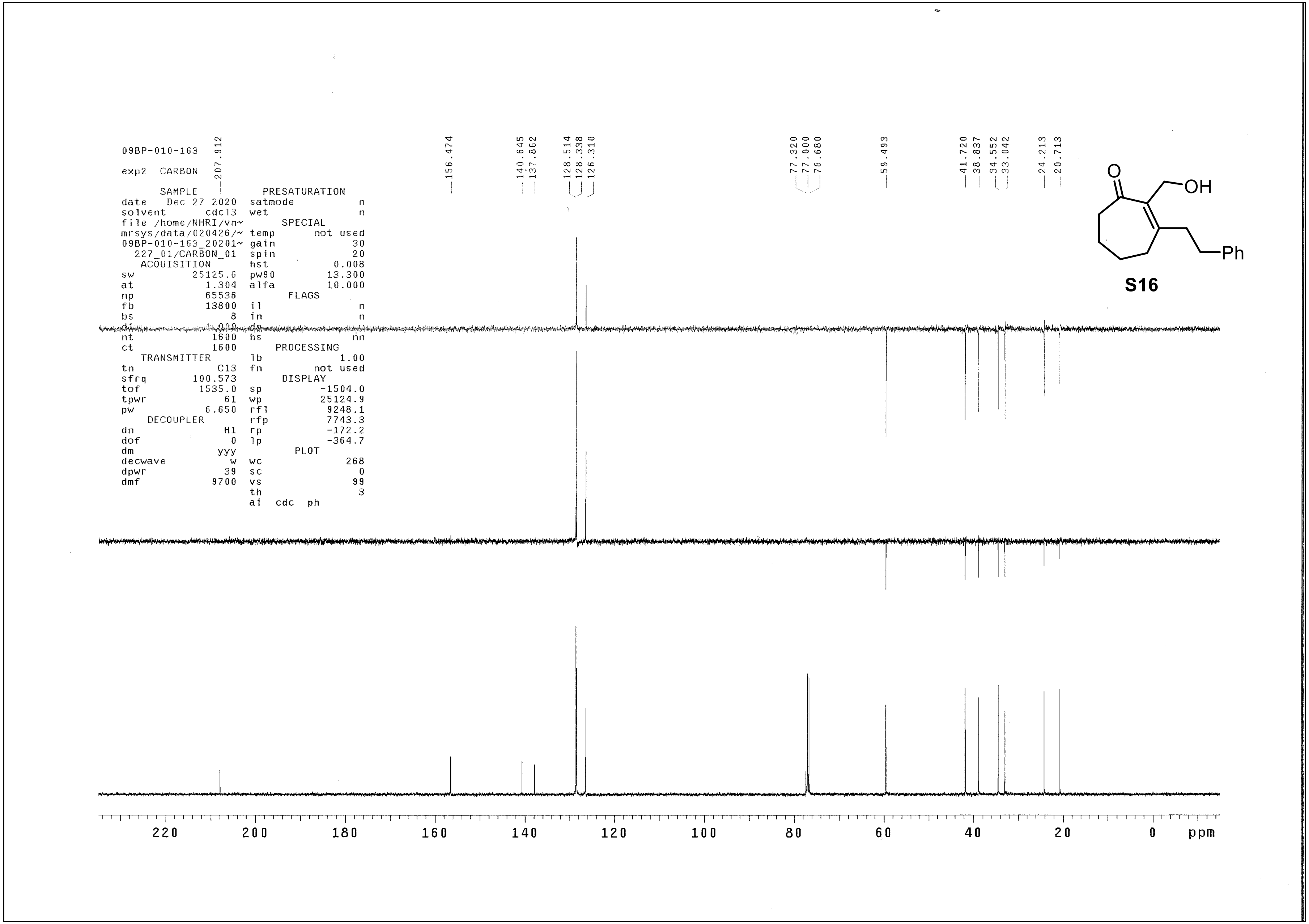


$^{13}$C NMR + DEPT spectra for compound **S16**

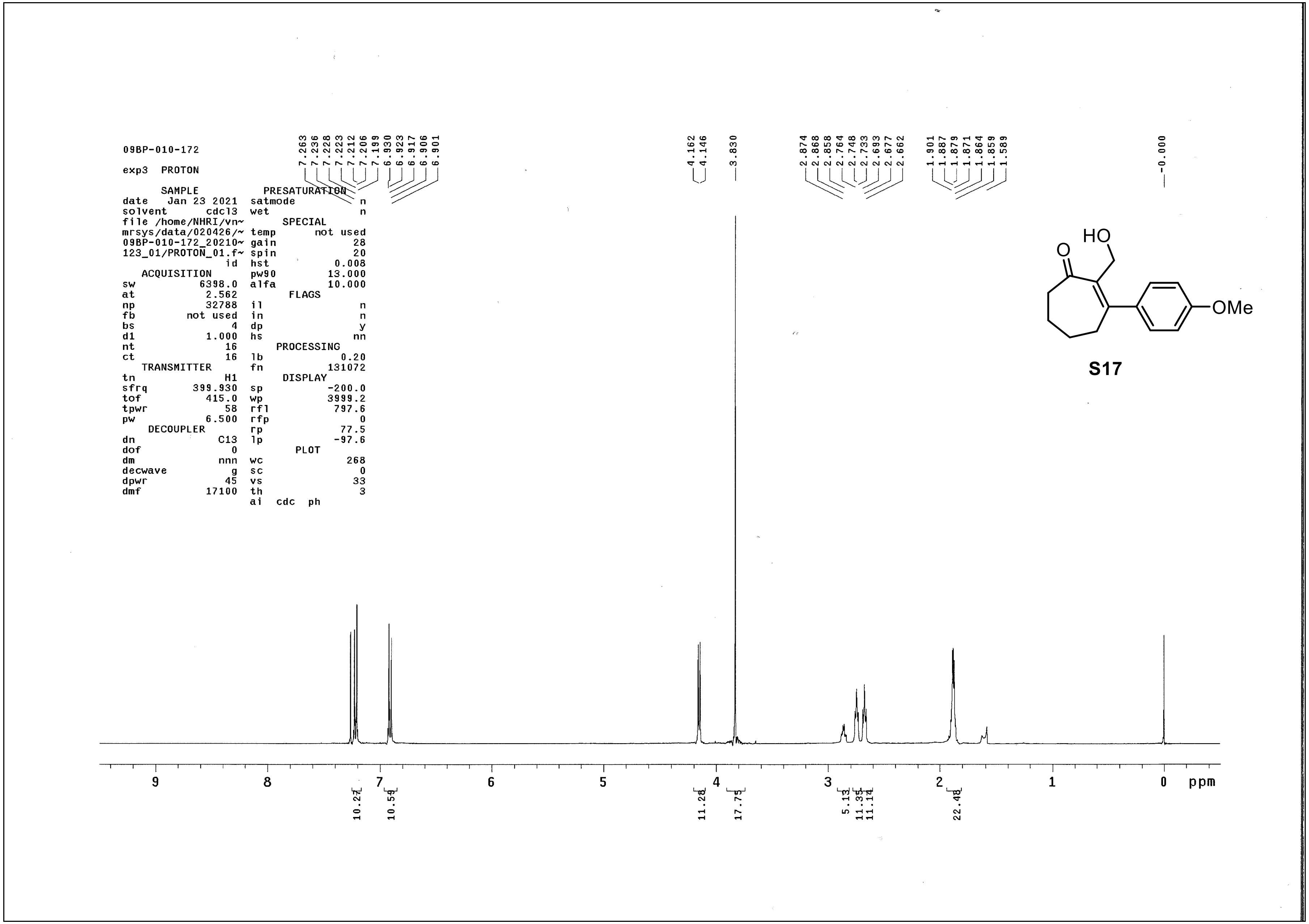


$^{1}$H NMR spectrum for compound **S17**

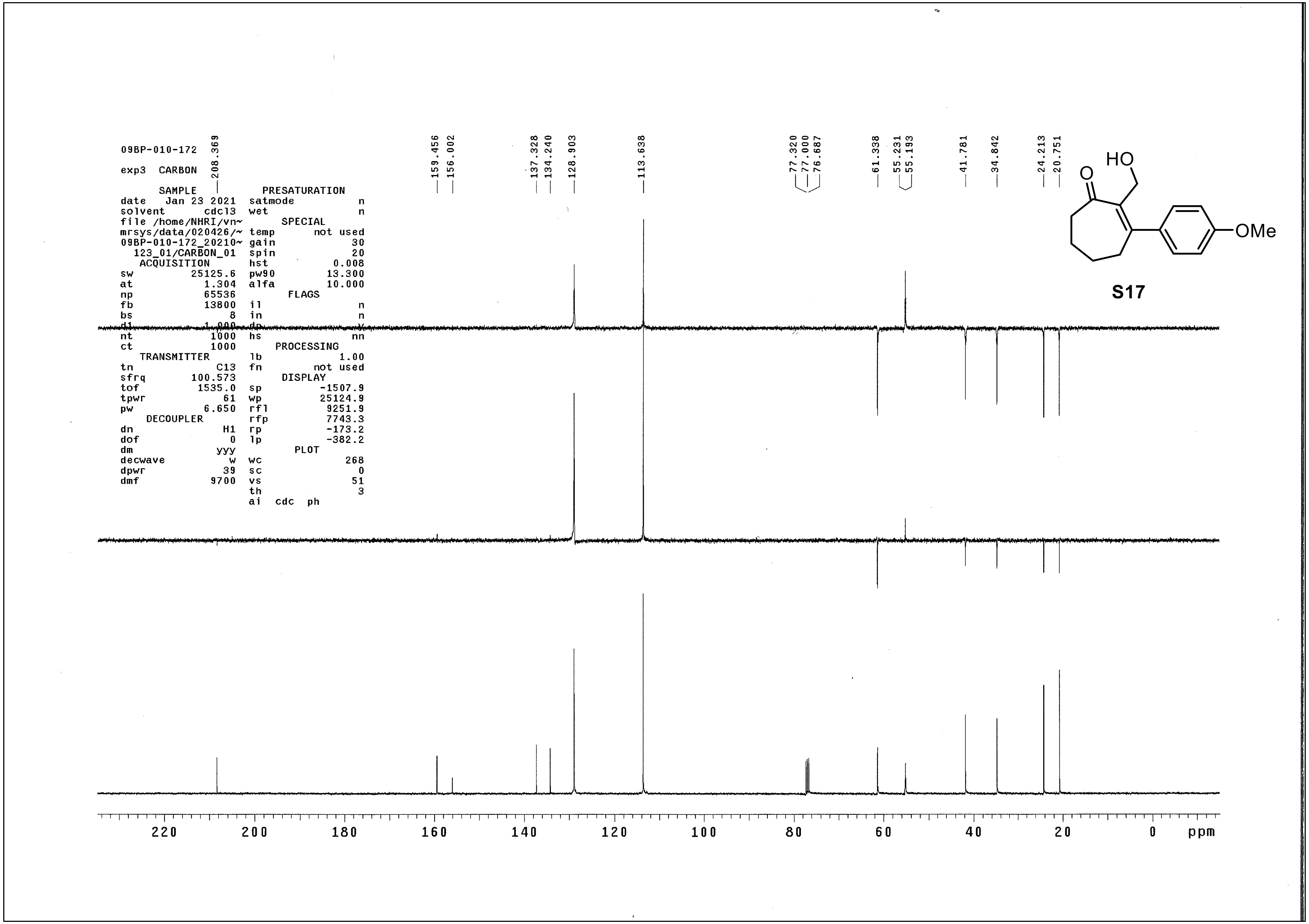


$^{13}$C NMR + DEPT spectra for compound **S17**

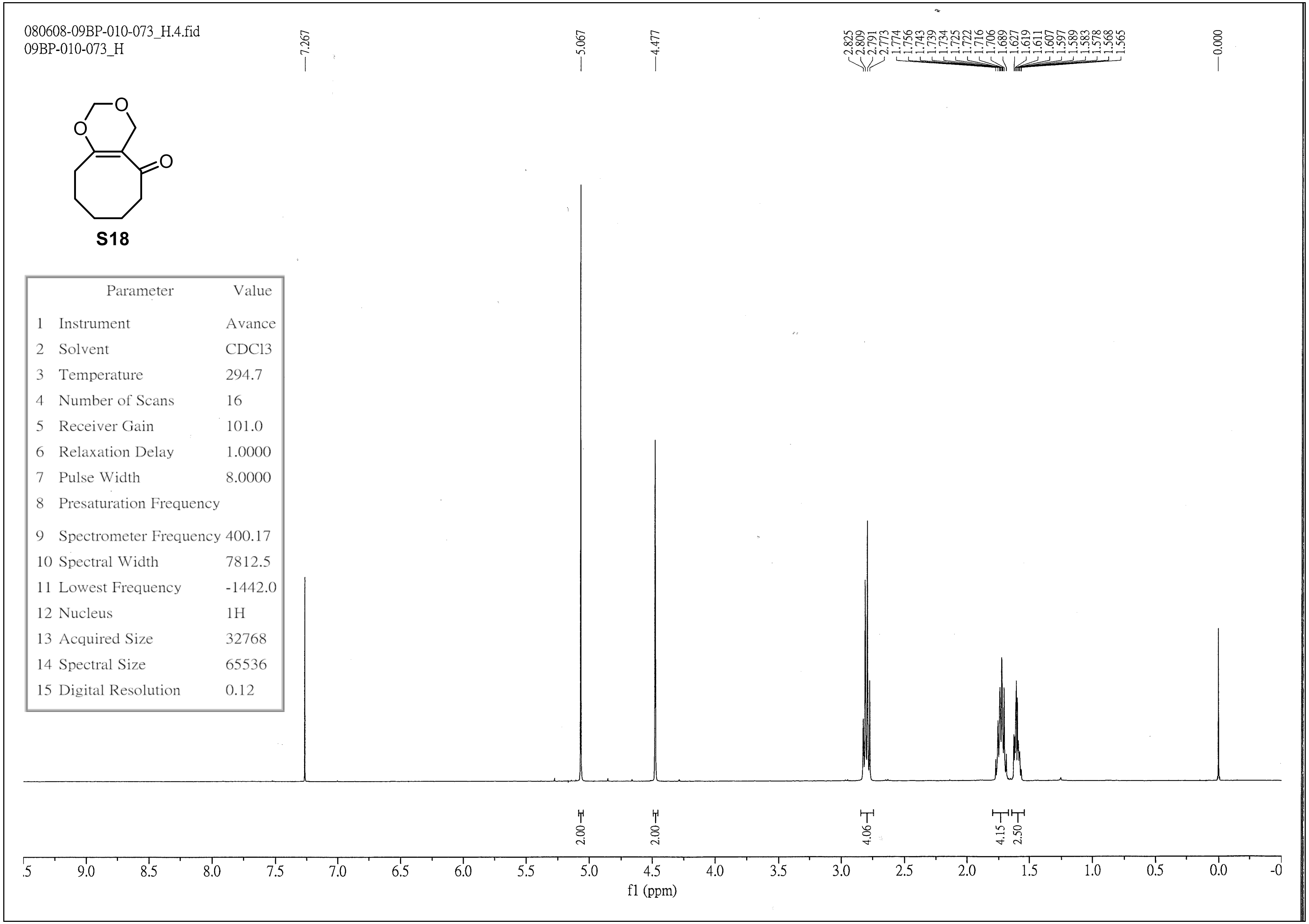


$^{1}$H NMR spectrum for compound **S18**

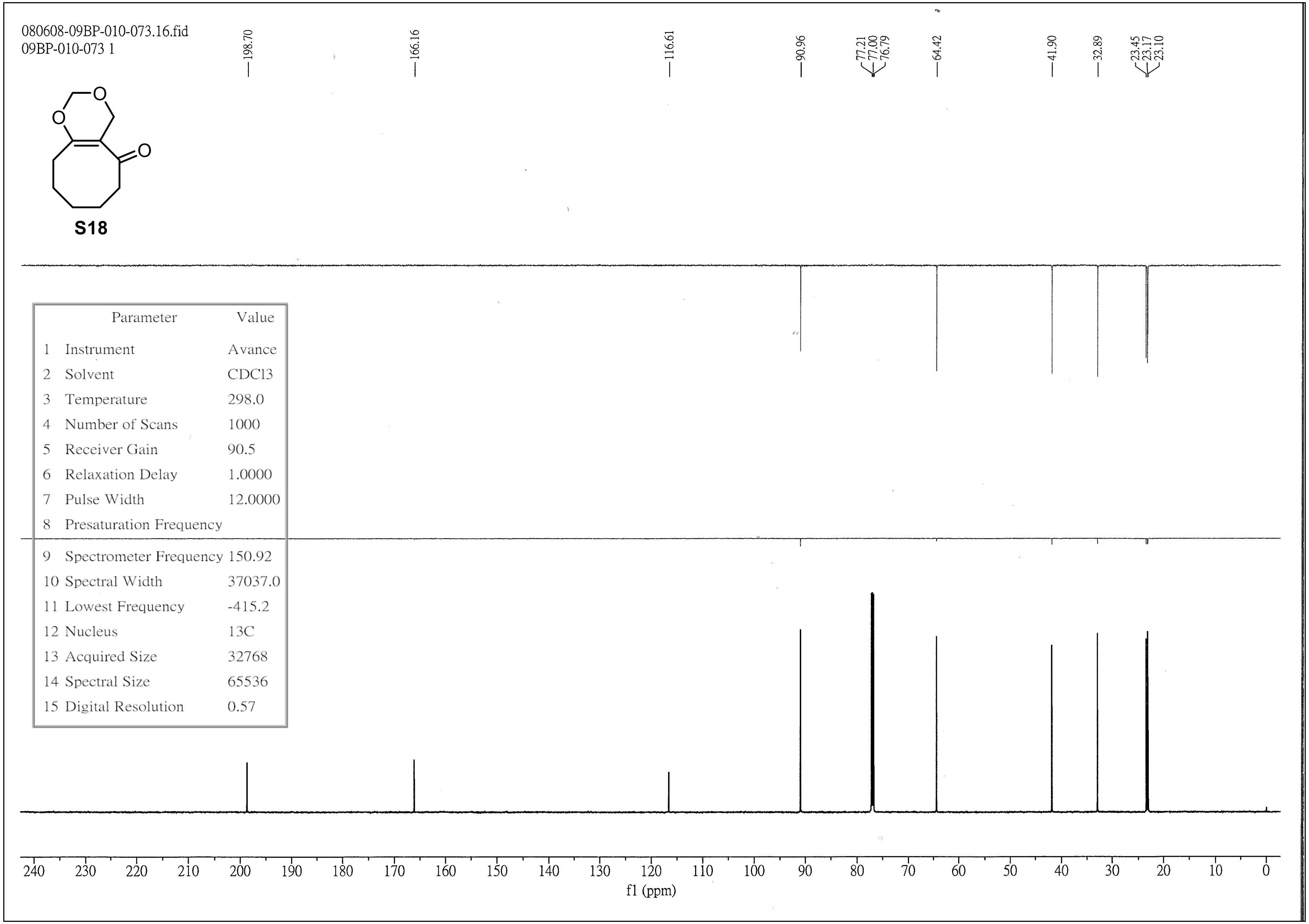


$^{13}$C NMR + DEPT spectra for compound **S18**

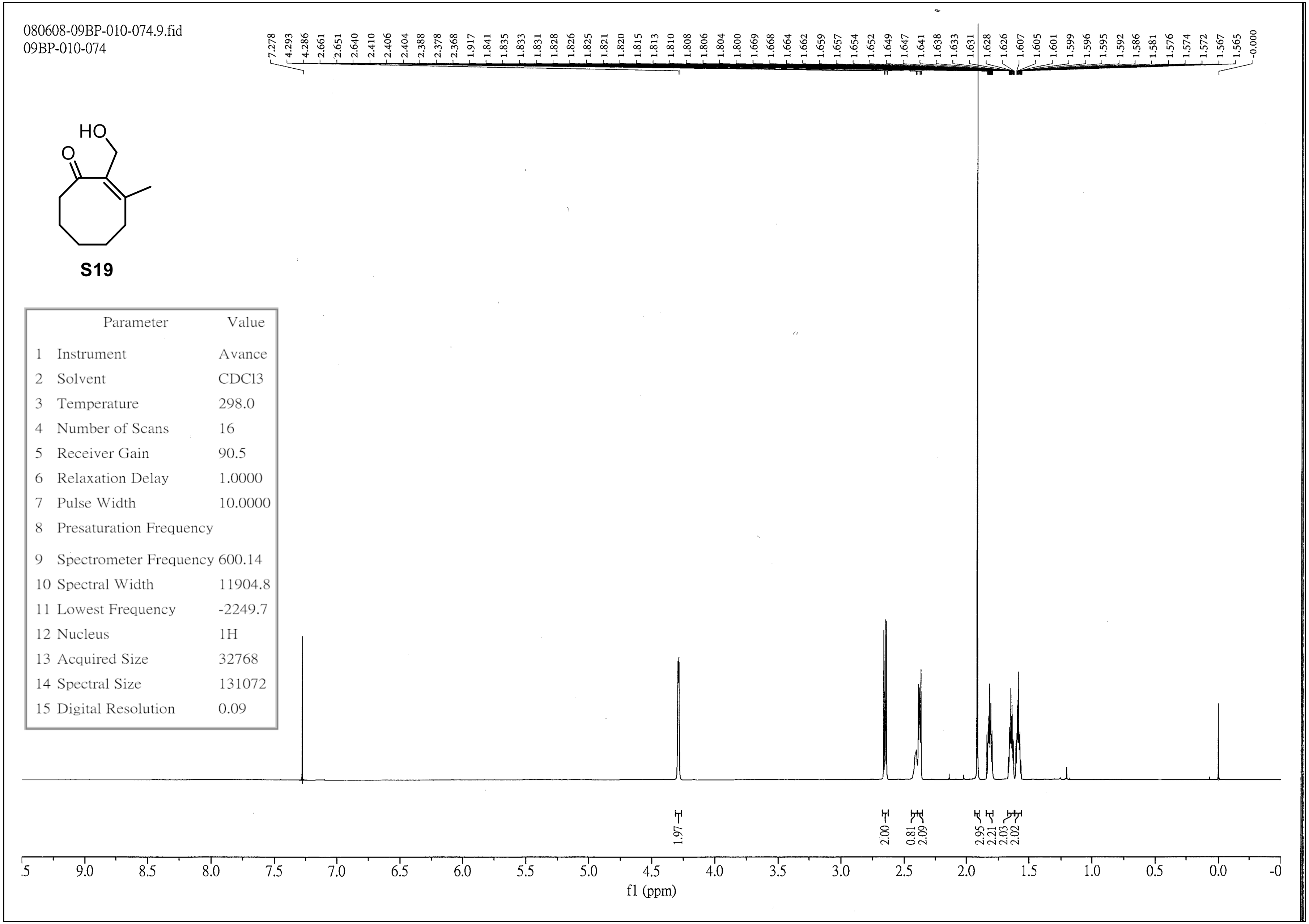


$^{1}$H NMR spectrum for compound **S19**

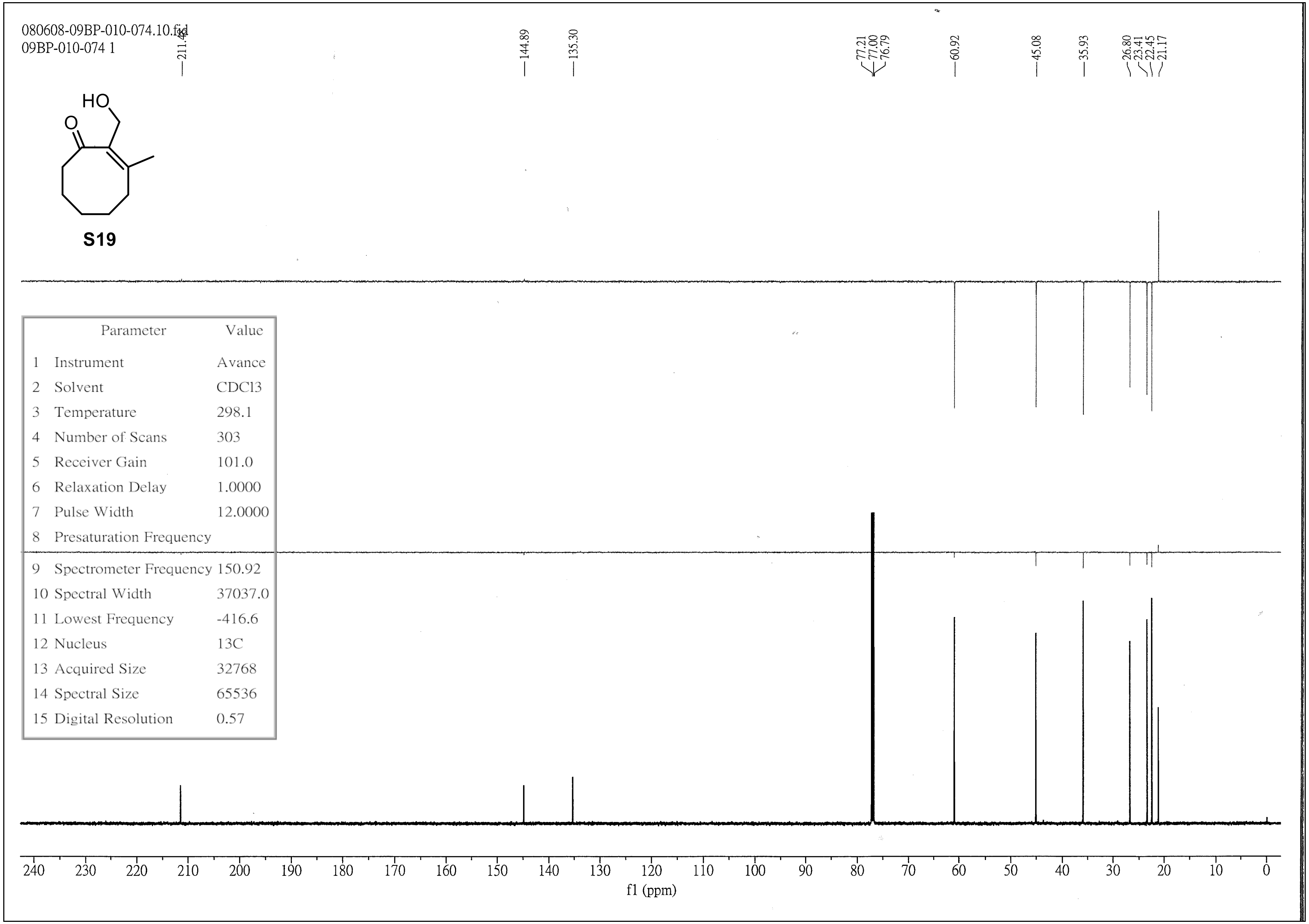


$^{13}$C NMR + DEPT spectra for compound **S19**

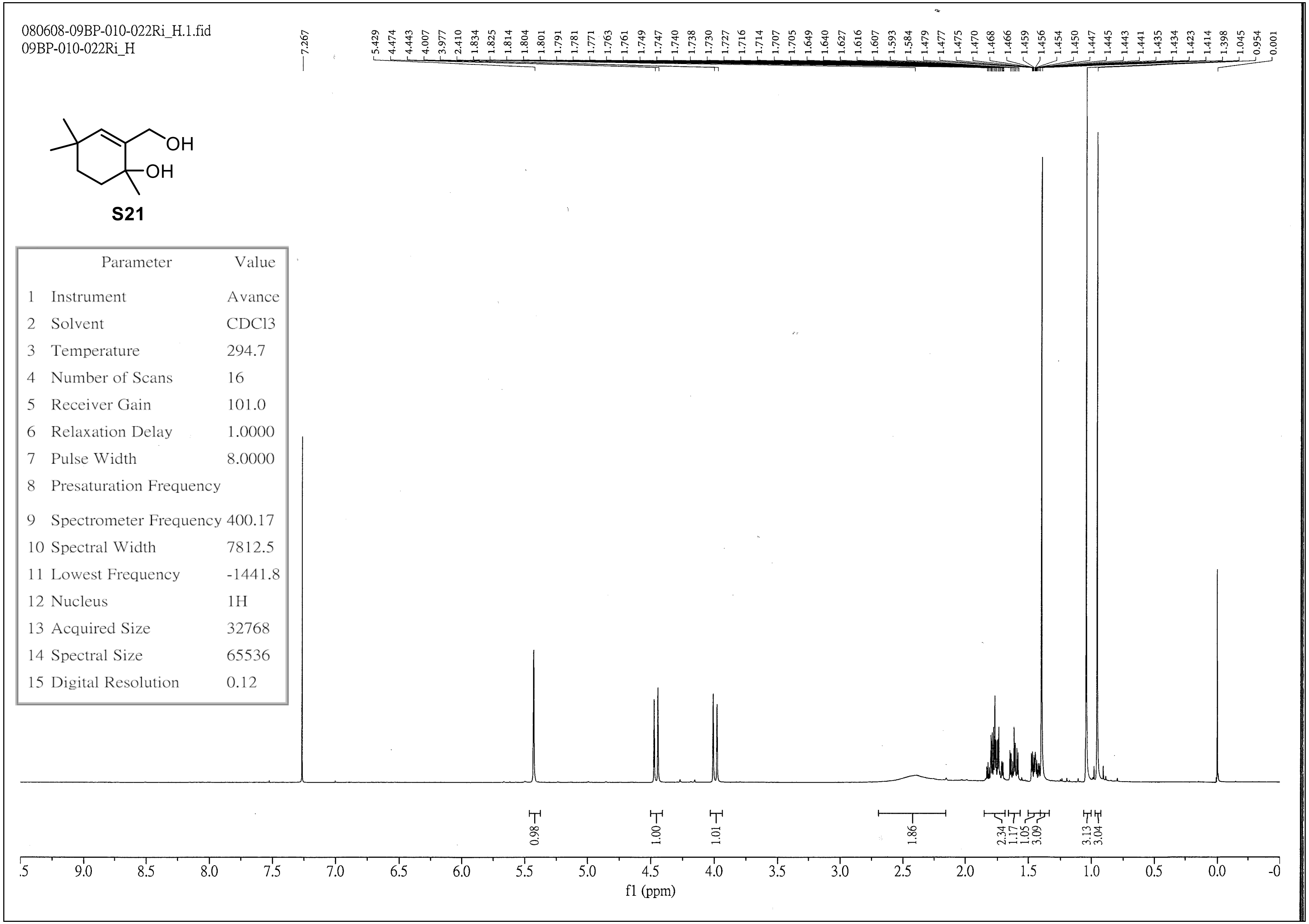


| | Parameter | Value |
|---|---|---|
| 1 | Instrument | Avance |
| 2 | Solvent | CDCl3 |
| 3 | Temperature | 294.7 |
| 4 | Number of Scans | 16 |
| 5 | Receiver Gain | 101.0 |
| 6 | Relaxation Delay | 1.0000 |
| 7 | Pulse Width | 8.0000 |
| 8 | Presaturation Frequency | |
| 9 | Spectrometer Frequency | 400.17 |
| 10 | Spectral Width | 7812.5 |
| 11 | Lowest Frequency | -1441.8 |
| 12 | Nucleus | 1H |
| 13 | Acquired Size | 32768 |
| 14 | Spectral Size | 65536 |
| 15 | Digital Resolution | 0.12 |

$^{1}$H NMR spectrum for compound **S21**

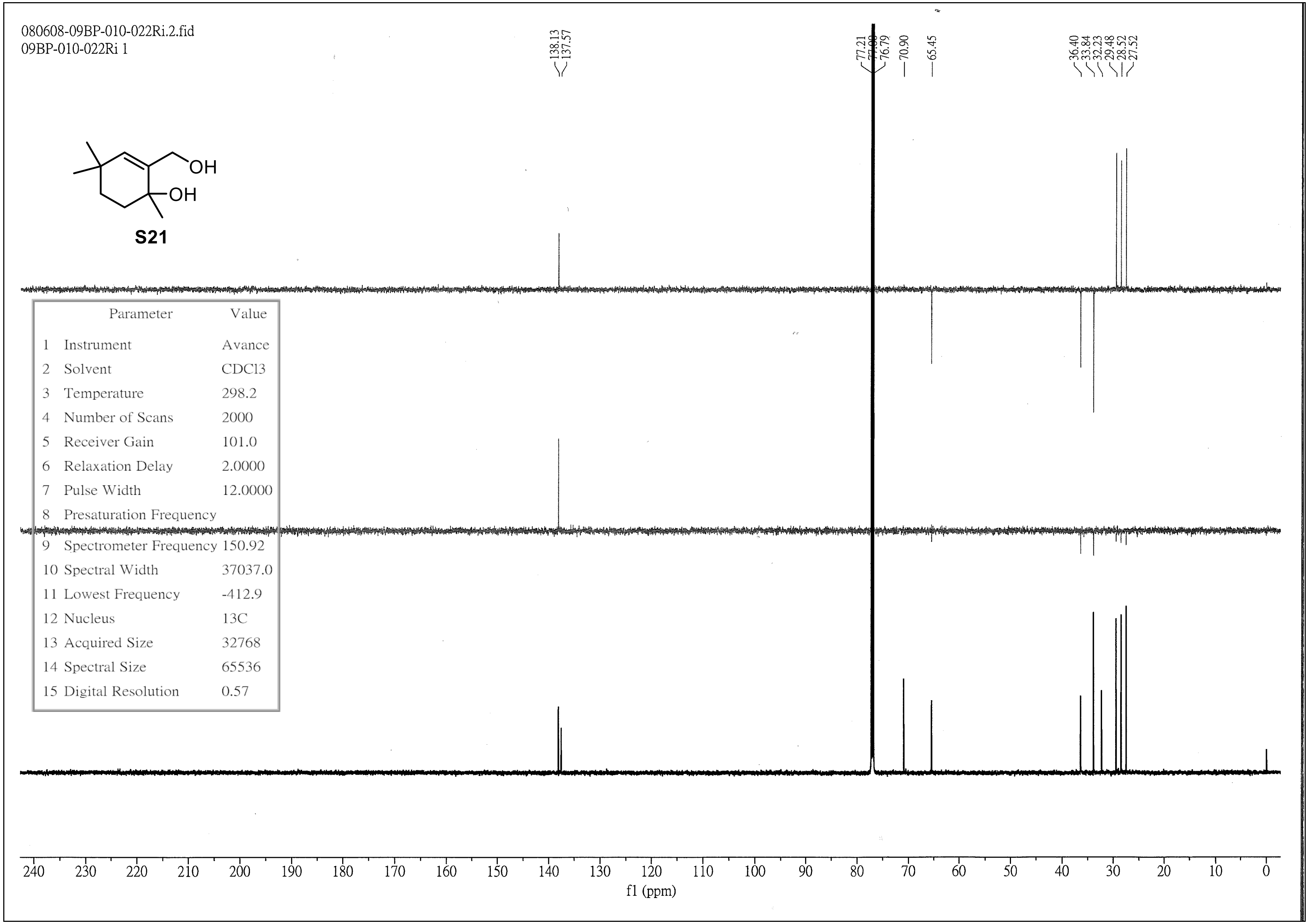


$^{13}$C NMR + DEPT spectra for compound **S21**

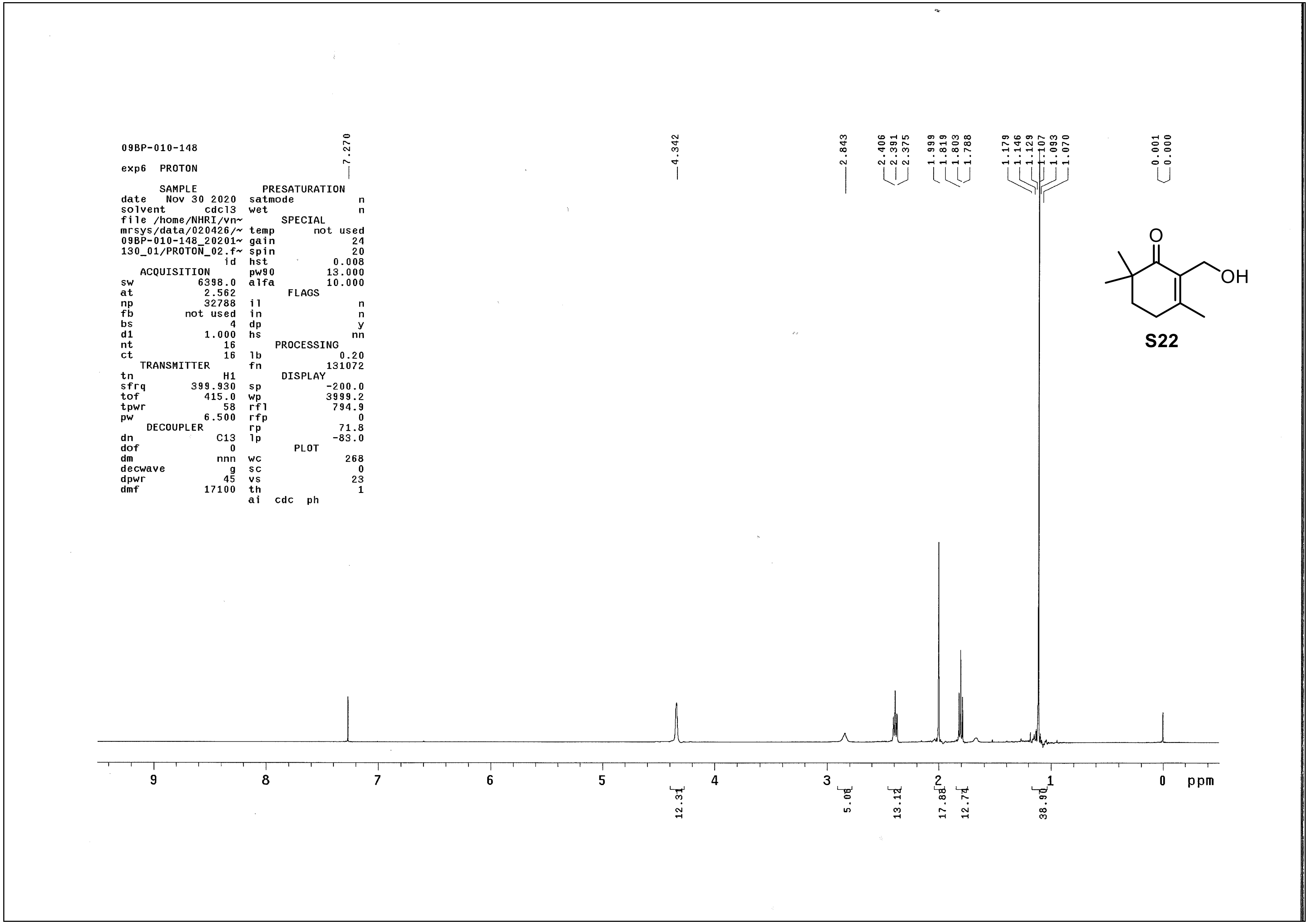


$^{1}$H NMR spectrum for compound **S22**

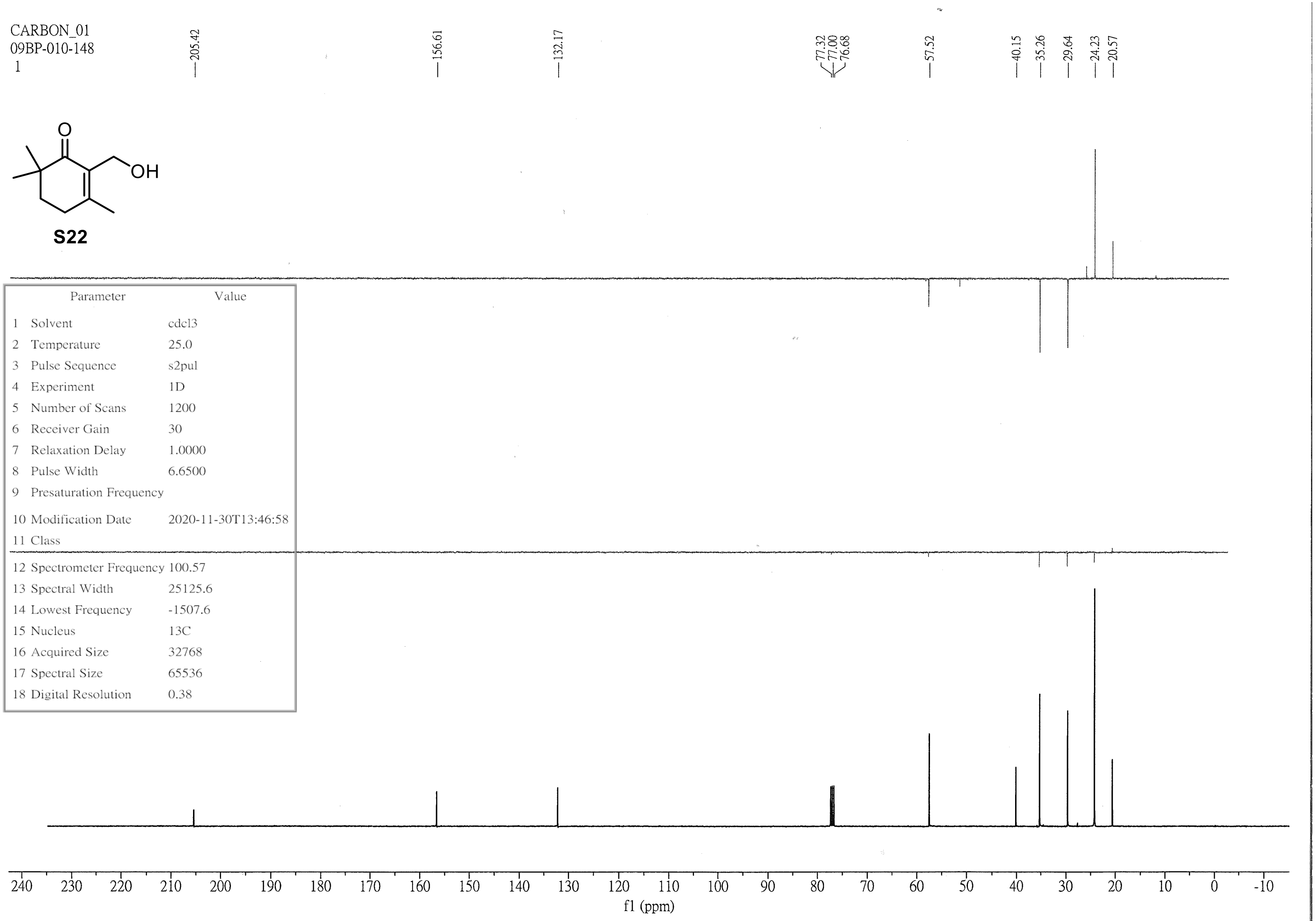


**S22**

| | Parameter | Value |
|---|---|---|
| 1 | Solvent | cdcl3 |
| 2 | Temperature | 25.0 |
| 3 | Pulse Sequence | s2pul |
| 4 | Experiment | 1D |
| 5 | Number of Scans | 1200 |
| 6 | Receiver Gain | 30 |
| 7 | Relaxation Delay | 1.0000 |
| 8 | Pulse Width | 6.6500 |
| 9 | Presaturation Frequency | |
| 10 | Modification Date | 2020-11-30T13:46:58 |
| 11 | Class | |
| 12 | Spectrometer Frequency | 100.57 |
| 13 | Spectral Width | 25125.6 |
| 14 | Lowest Frequency | -1507.6 |
| 15 | Nucleus | 13C |
| 16 | Acquired Size | 32768 |
| 17 | Spectral Size | 65536 |
| 18 | Digital Resolution | 0.38 |

$^{13}$C NMR + DEPT spectra for compound **S22**

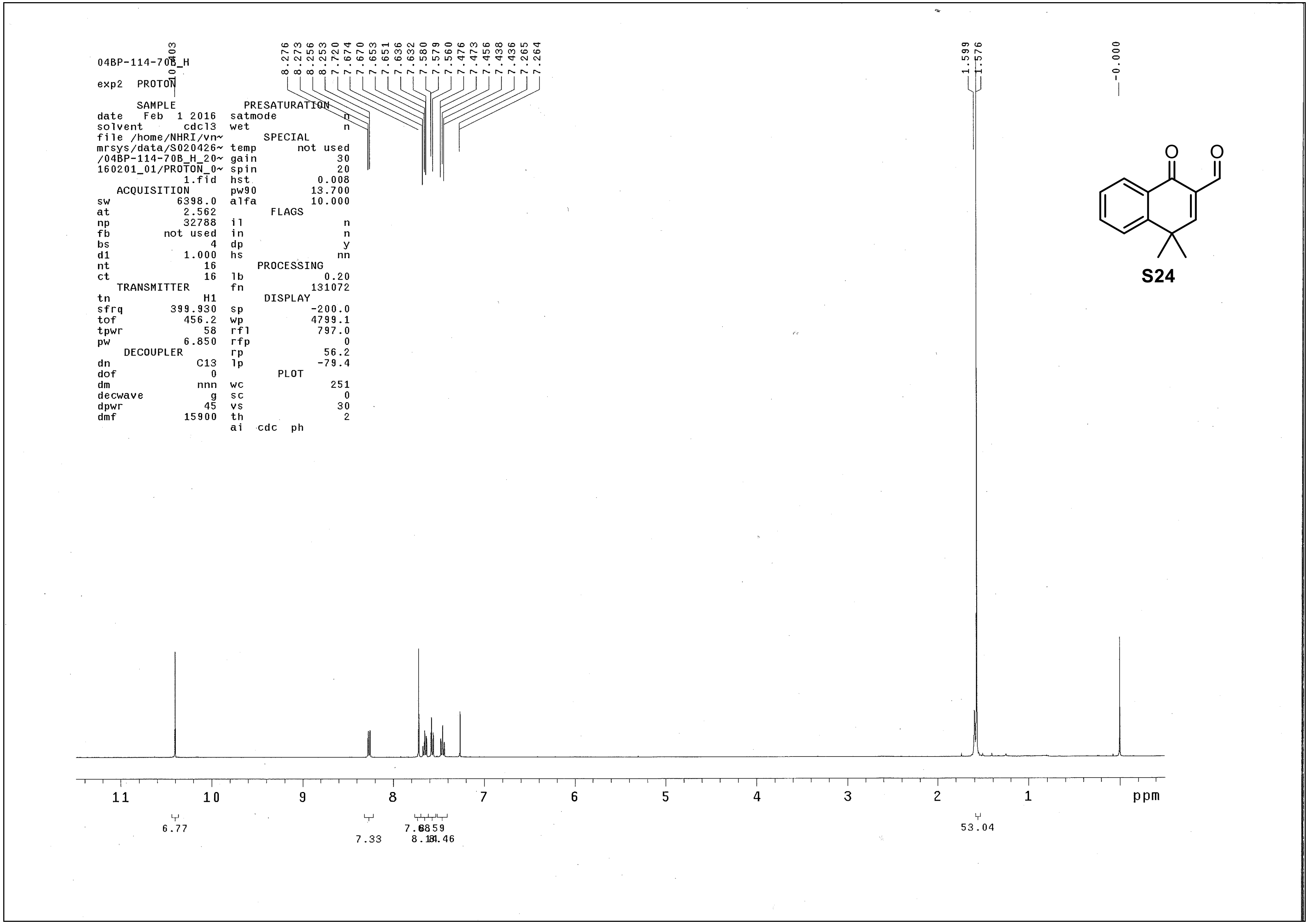


$^{1}$H NMR spectrum for compound **S24**

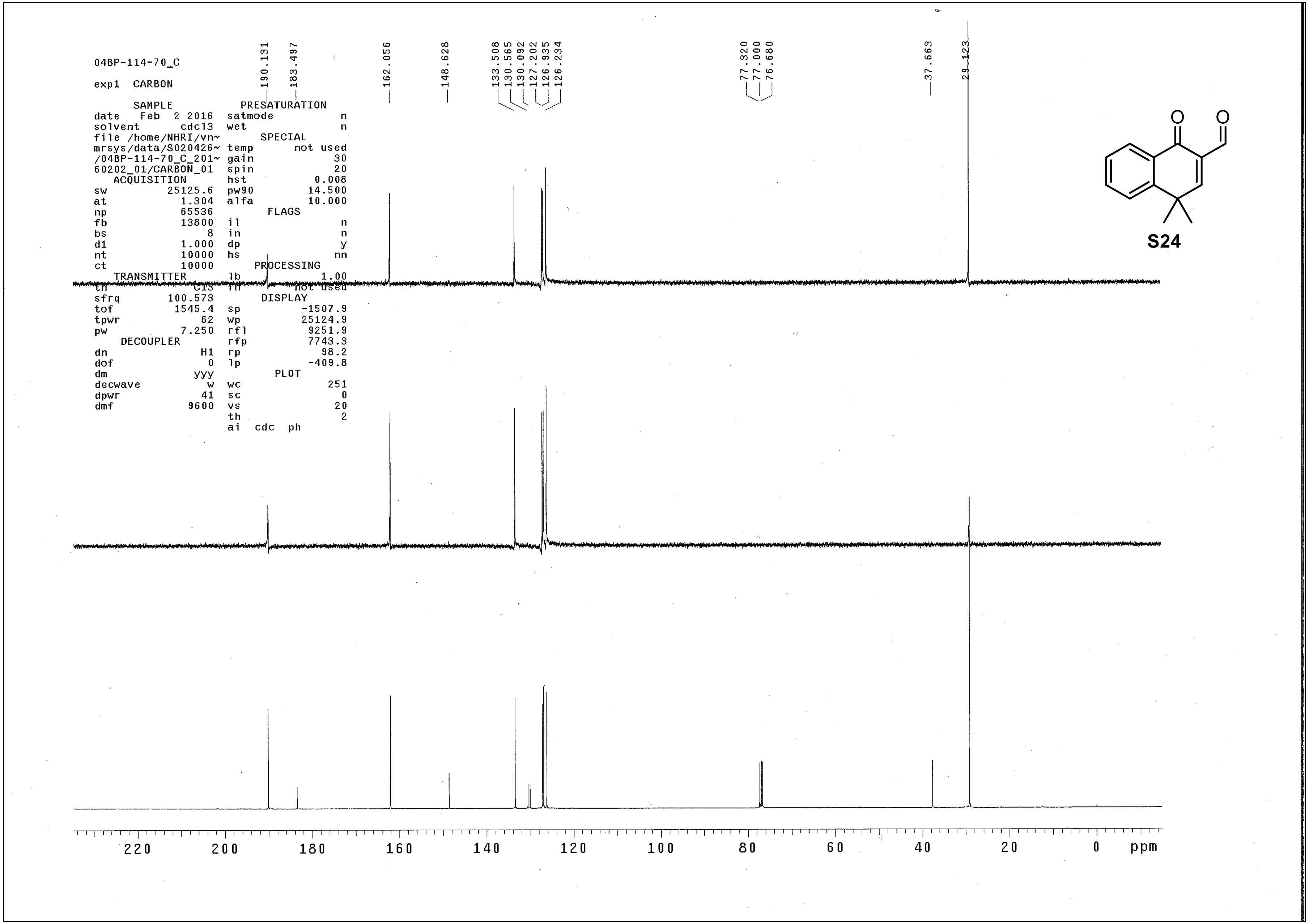

$^{13}$C NMR + DEPT spectra for compound **S24**

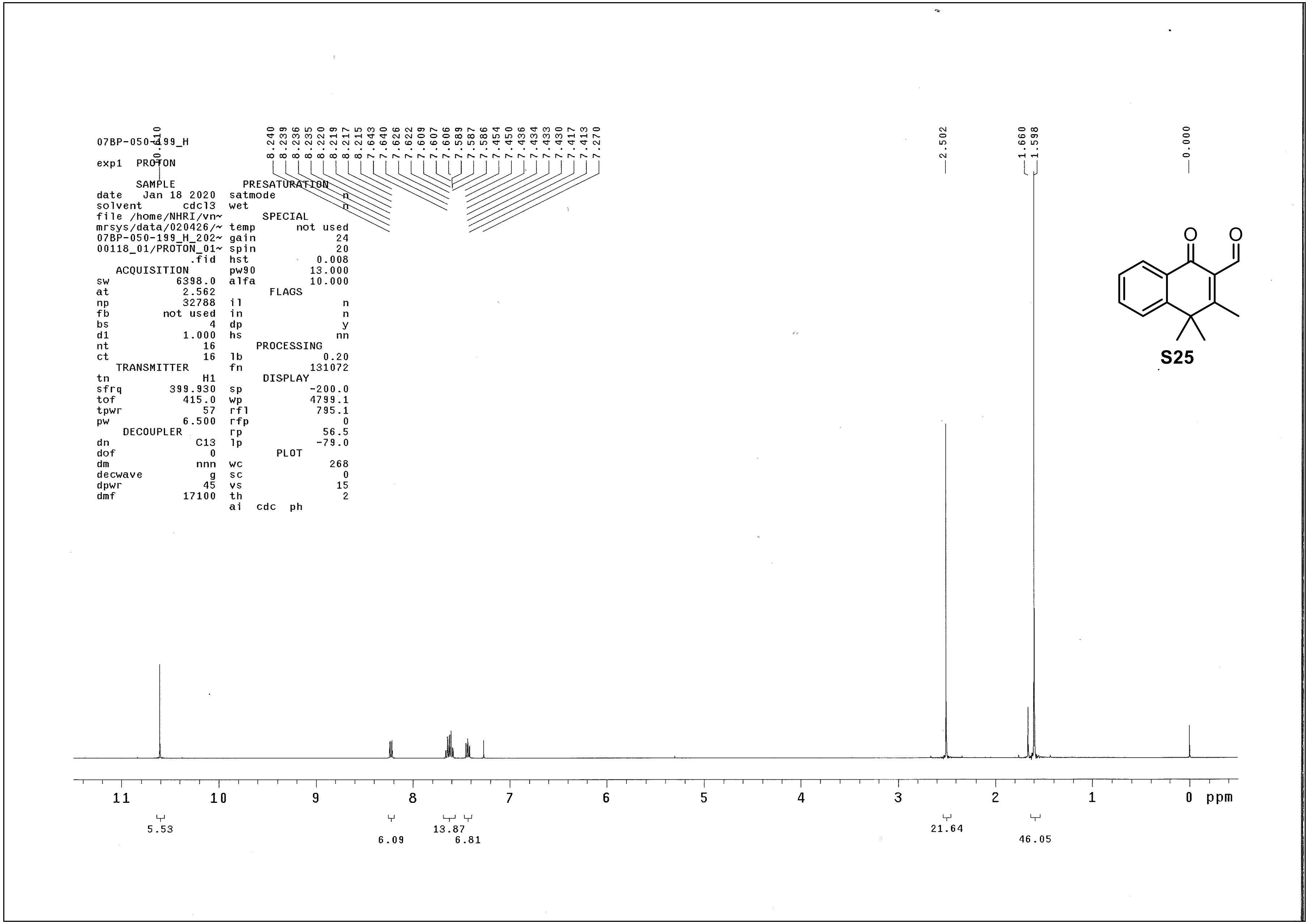


$^{1}$H NMR spectrum for compound **S25**

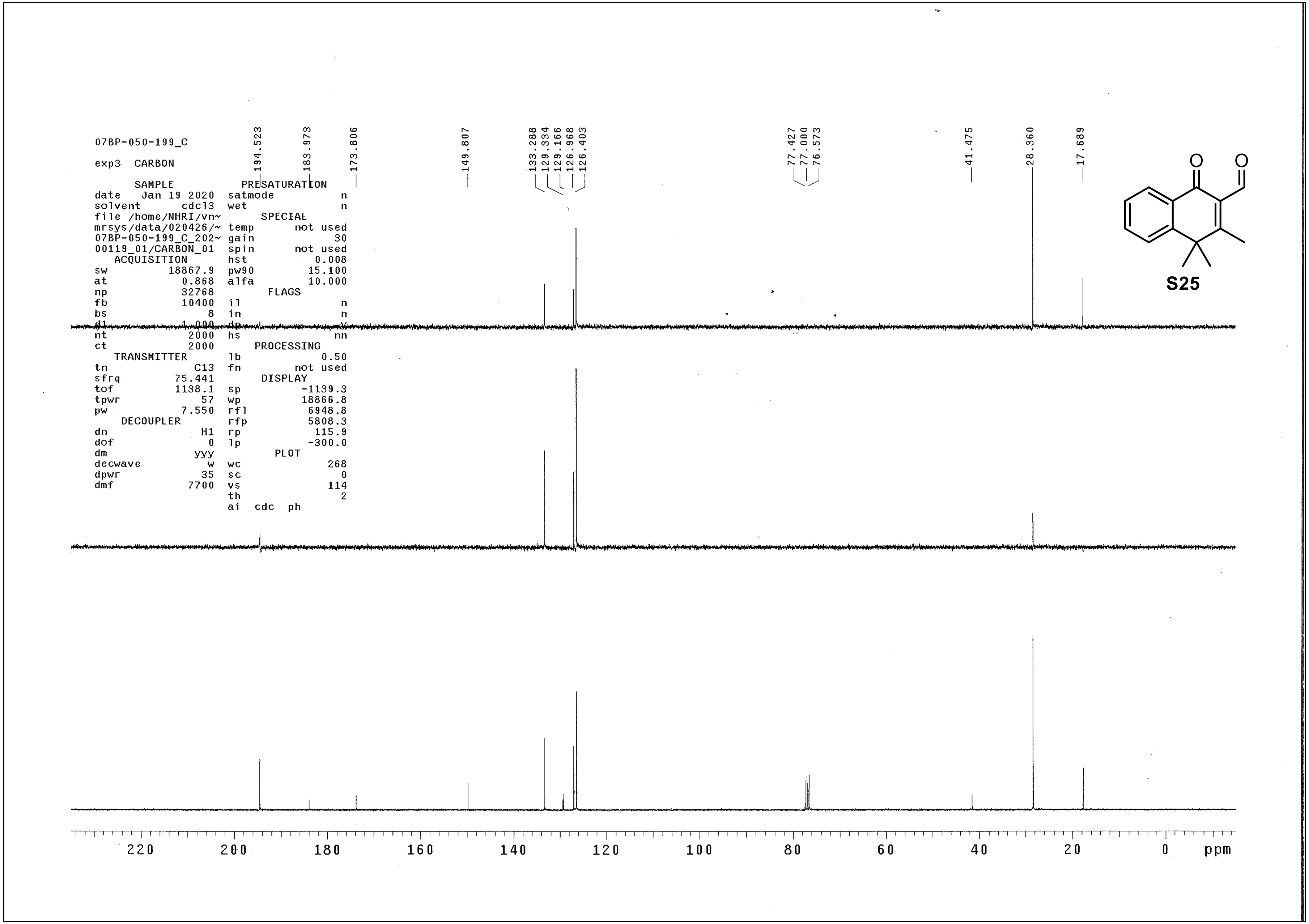


$^{13}$C NMR + DEPT spectra for compound **S25**

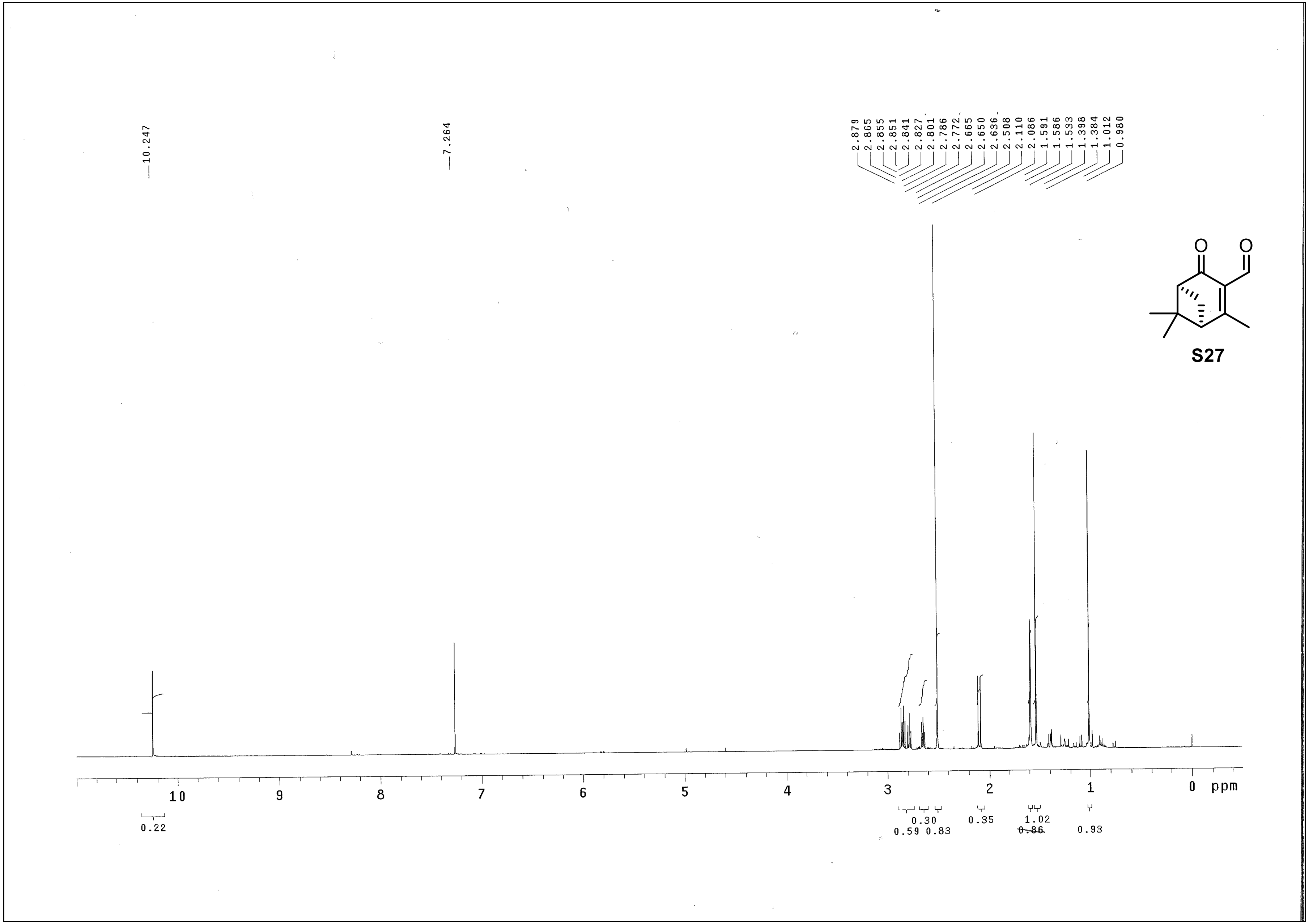


$^1$H NMR spectrum for compound **S27**

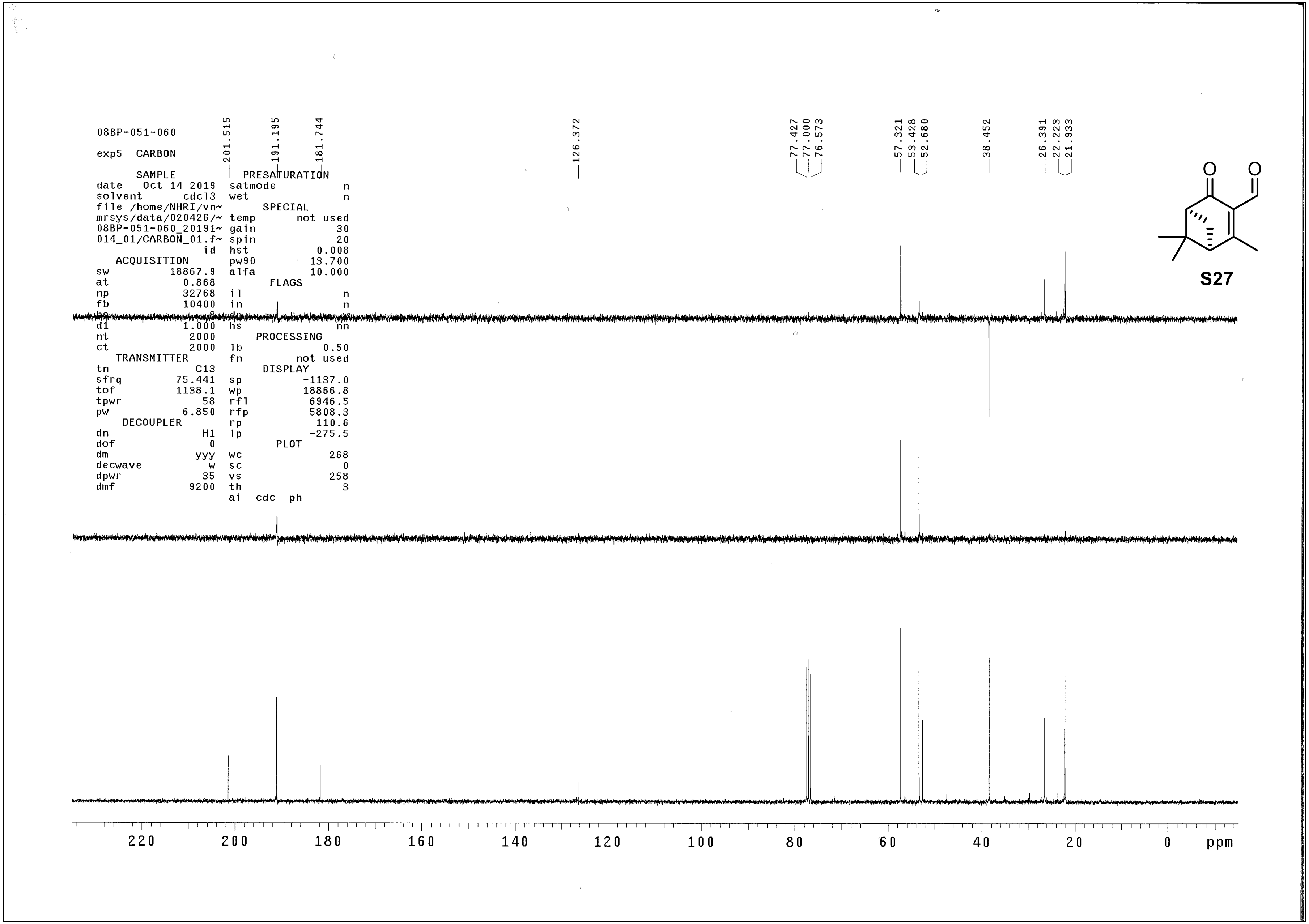


$^{13}$C NMR + DEPT spectra for compound **S27**

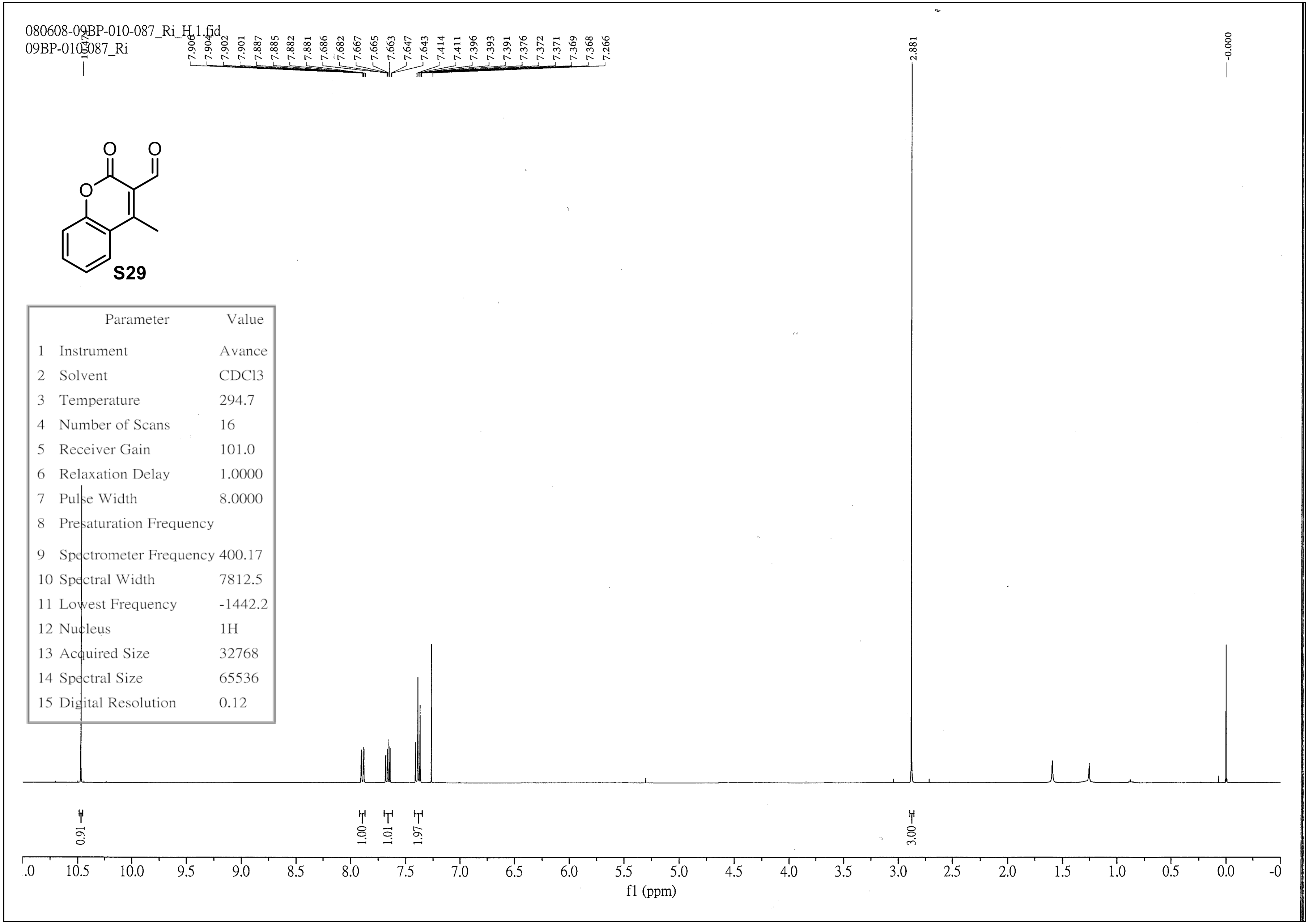


$^{1}$H NMR spectrum for compound **S29**

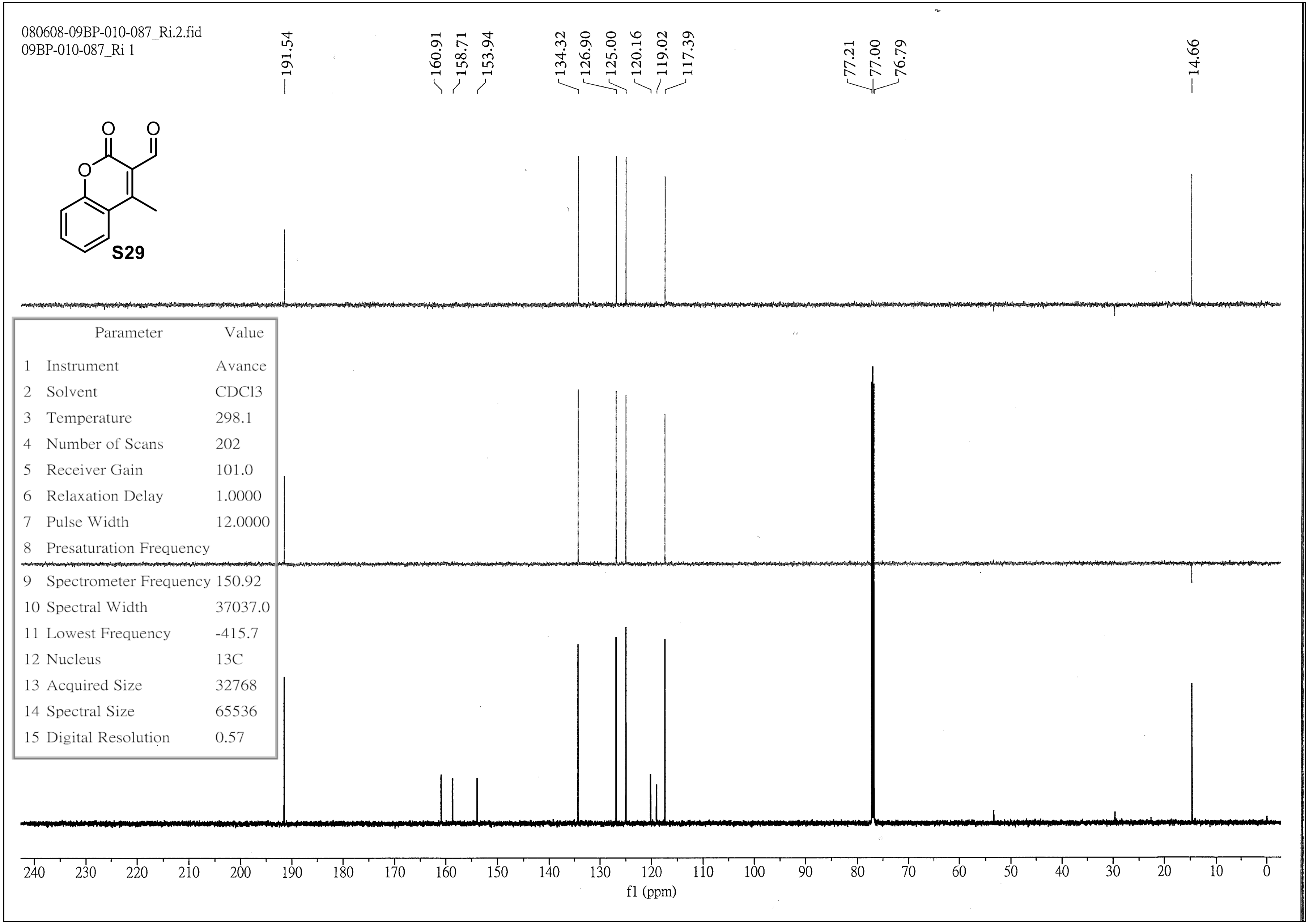


$^{13}$C NMR + DEPT spectra for compound **S29**

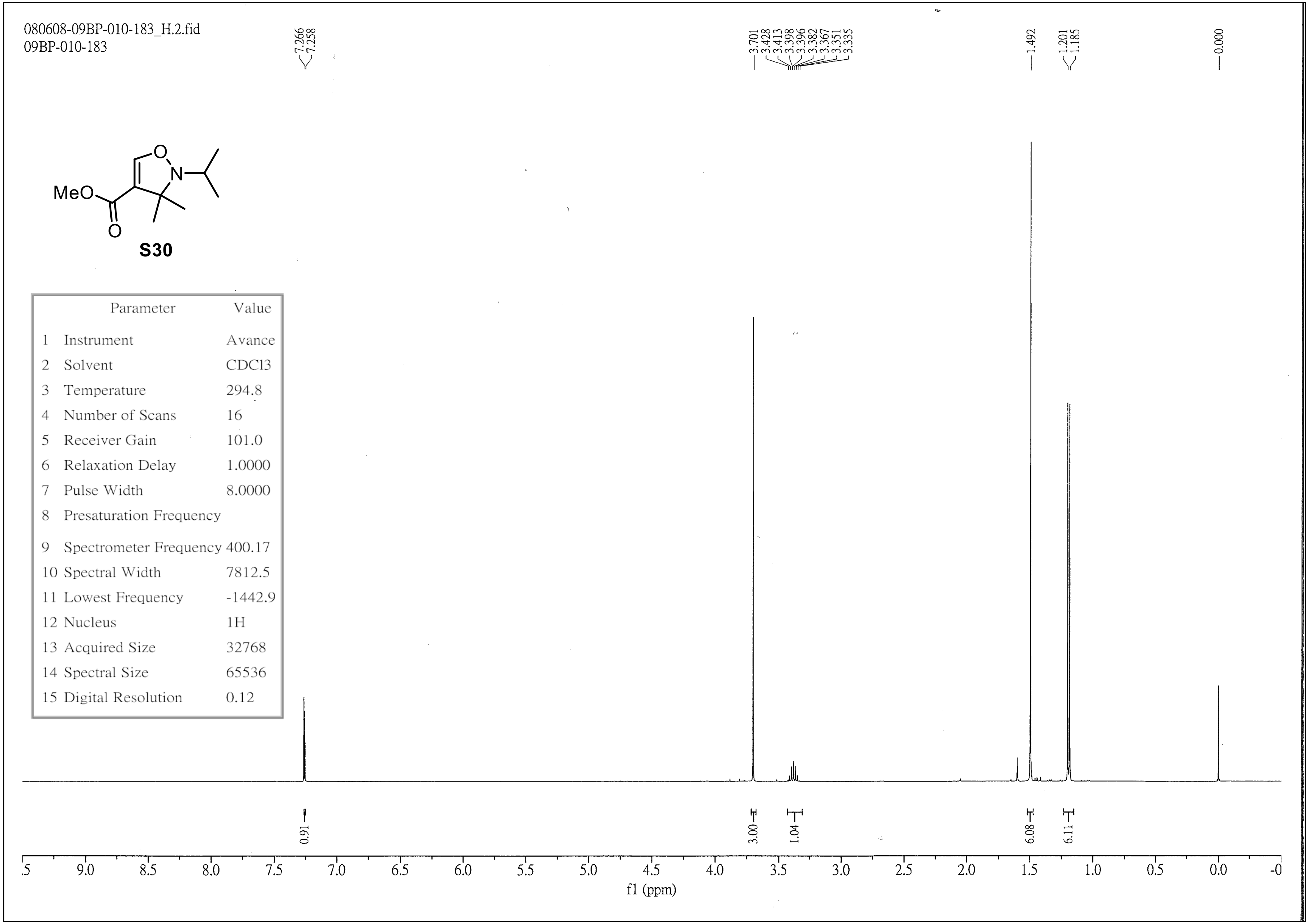


$^{1}$H NMR spectrum for compound **S30**

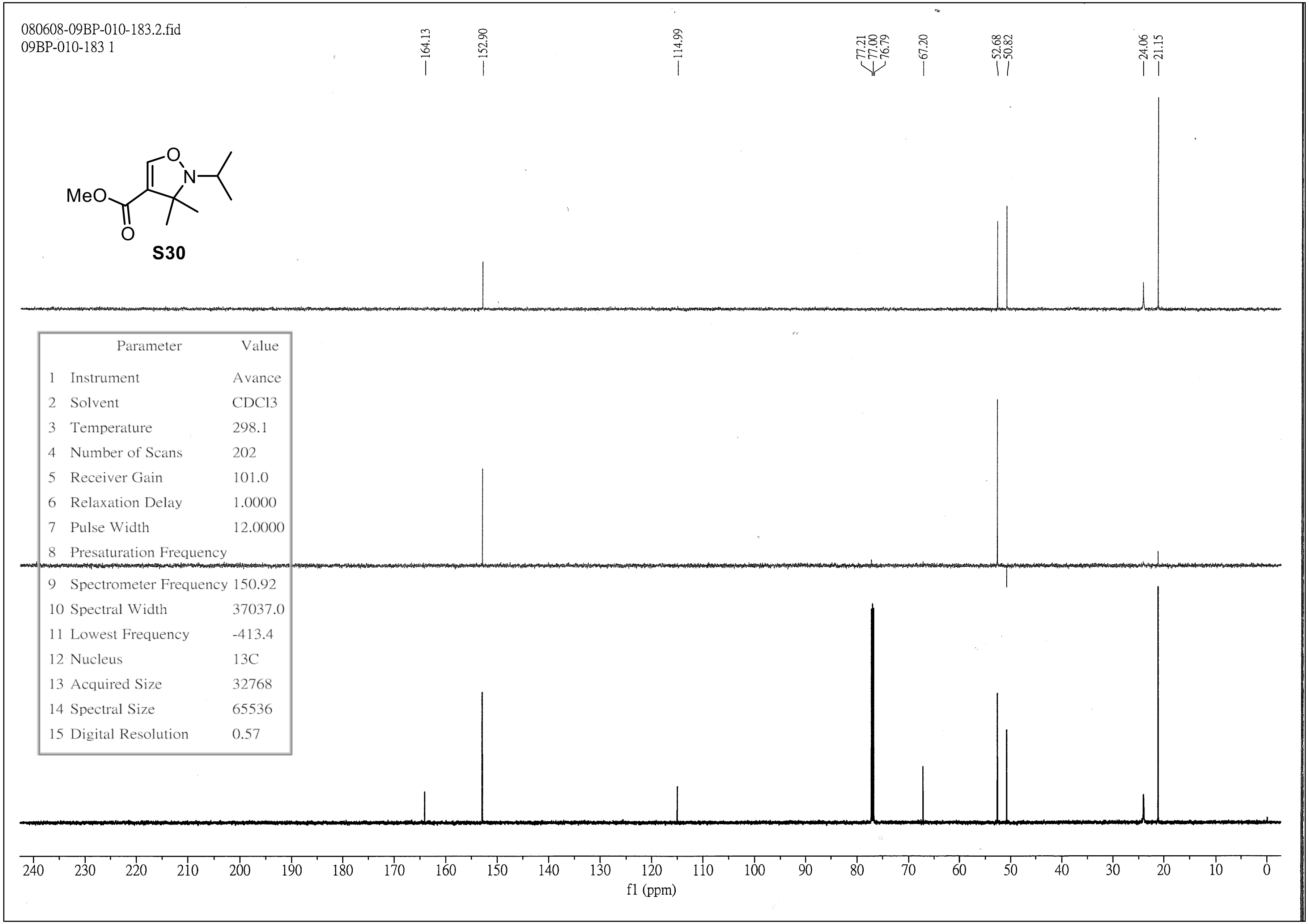


$^{13}$C NMR + DEPT spectra for compound **S30**

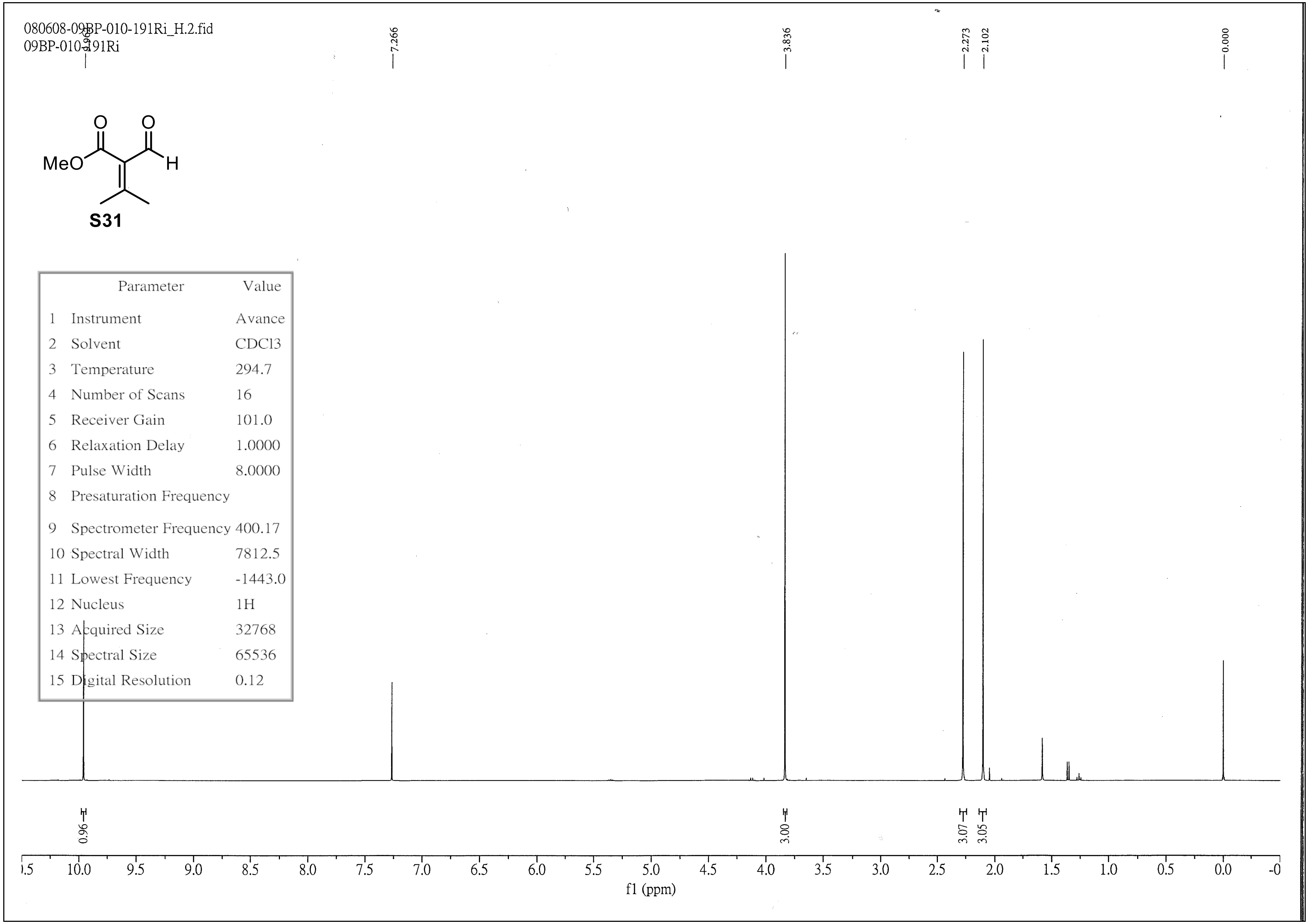


$^{1}$H NMR spectrum for compound **S31**

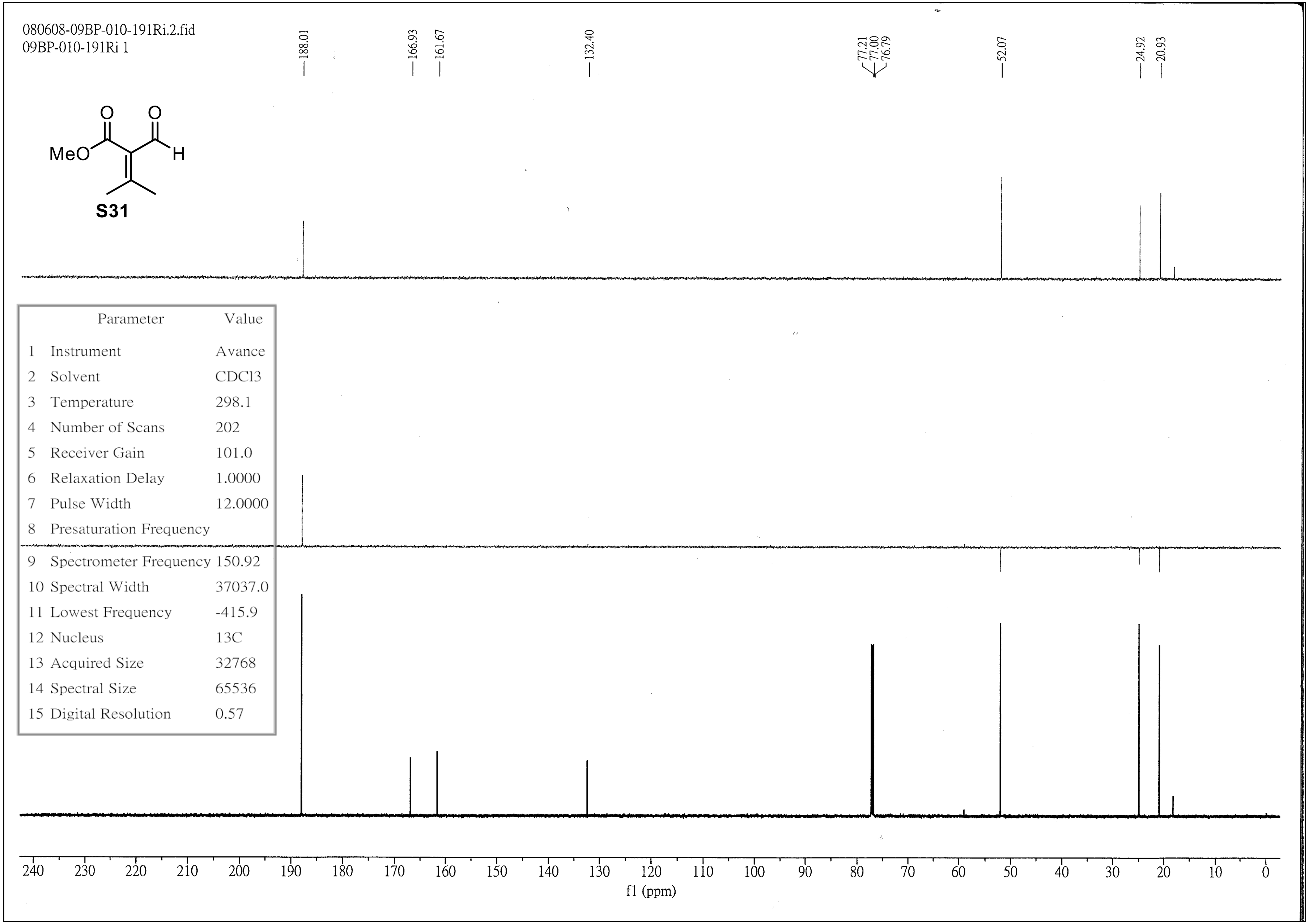


$^{13}C$ NMR + DEPT spectra for compound **S31**

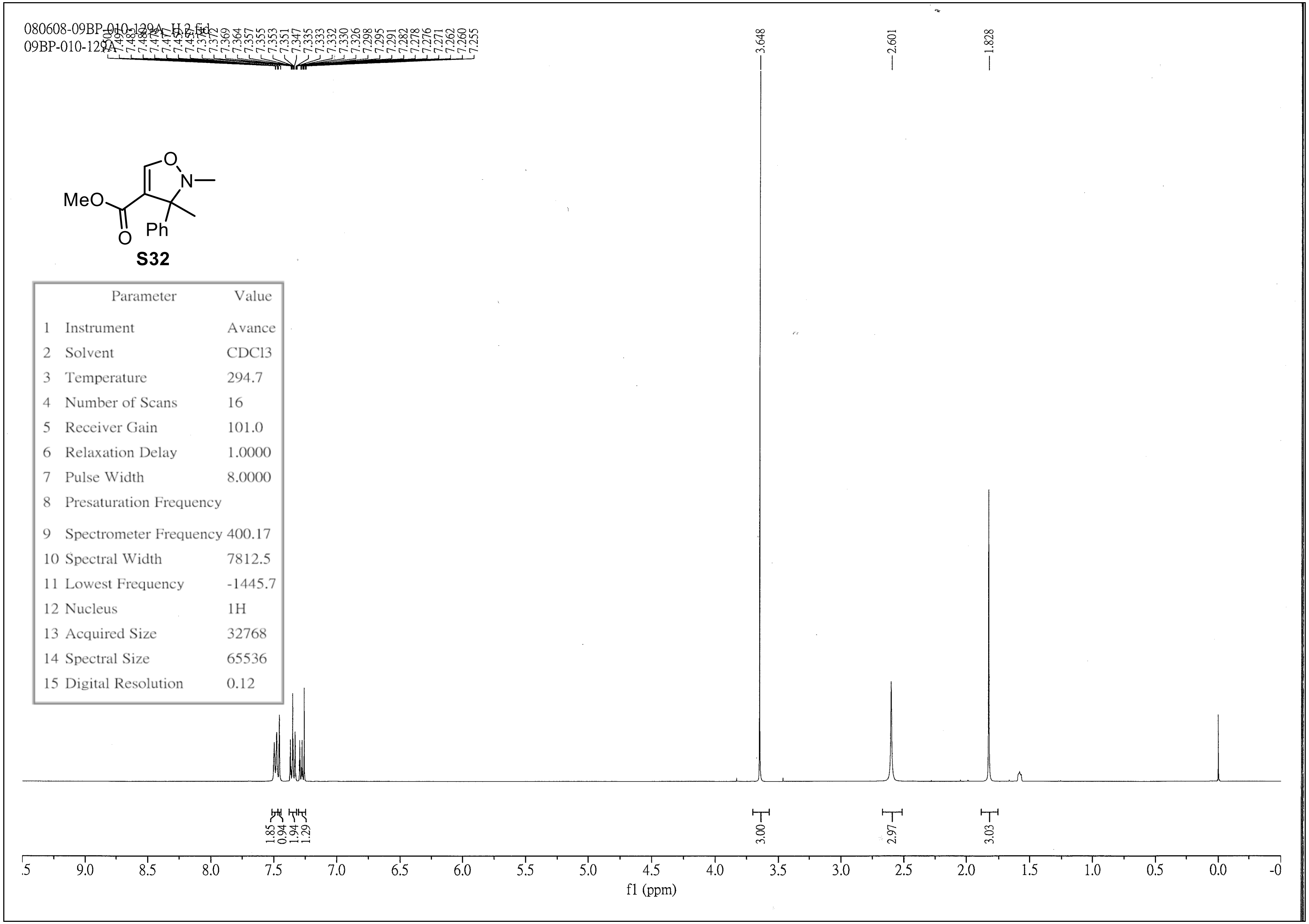


$^{1}$H NMR spectrum for compound **S32**

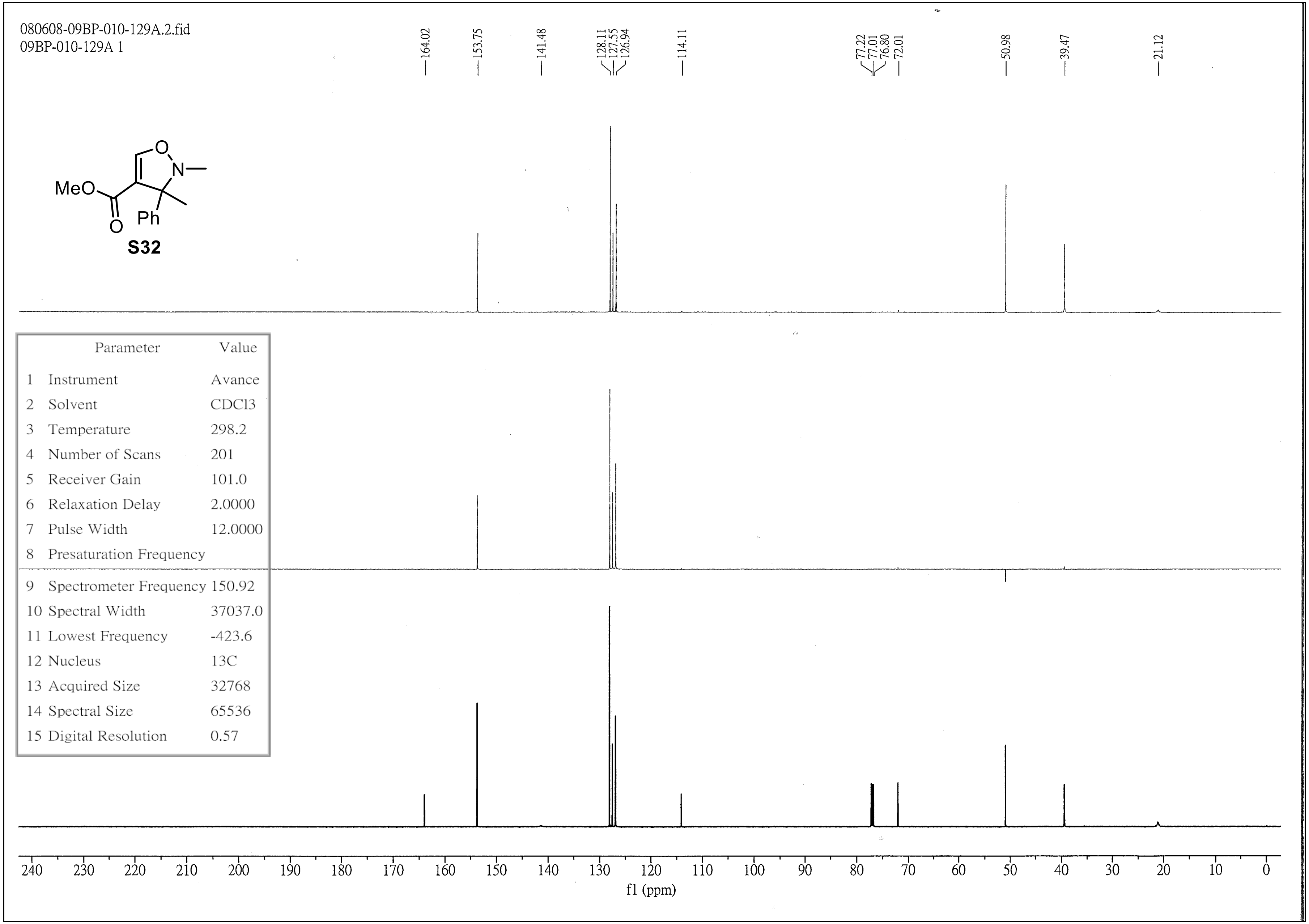


| | Parameter | Value |
|---|---|---|
| 1 | Instrument | Avance |
| 2 | Solvent | CDCl3 |
| 3 | Temperature | 298.2 |
| 4 | Number of Scans | 201 |
| 5 | Receiver Gain | 101.0 |
| 6 | Relaxation Delay | 2.0000 |
| 7 | Pulse Width | 12.0000 |
| 8 | Presaturation Frequency | |
| 9 | Spectrometer Frequency | 150.92 |
| 10 | Spectral Width | 37037.0 |
| 11 | Lowest Frequency | -423.6 |
| 12 | Nucleus | 13C |
| 13 | Acquired Size | 32768 |
| 14 | Spectral Size | 65536 |
| 15 | Digital Resolution | 0.57 |

$^{13}$C NMR + DEPT spectra for compound **S32**

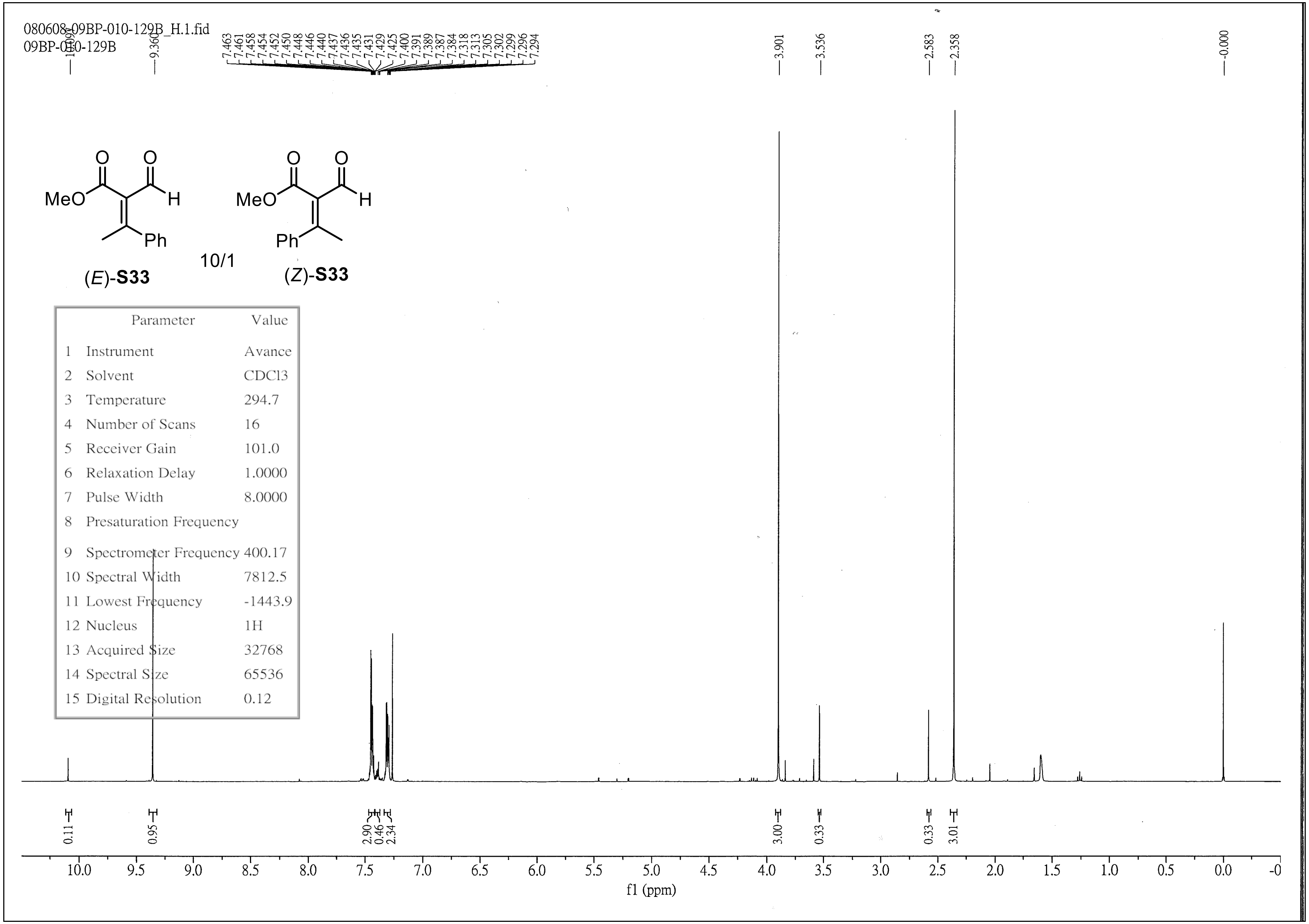


$^{1}$H NMR spectrum for compounds (*E*)-**S33** and (*Z*)-**S33**

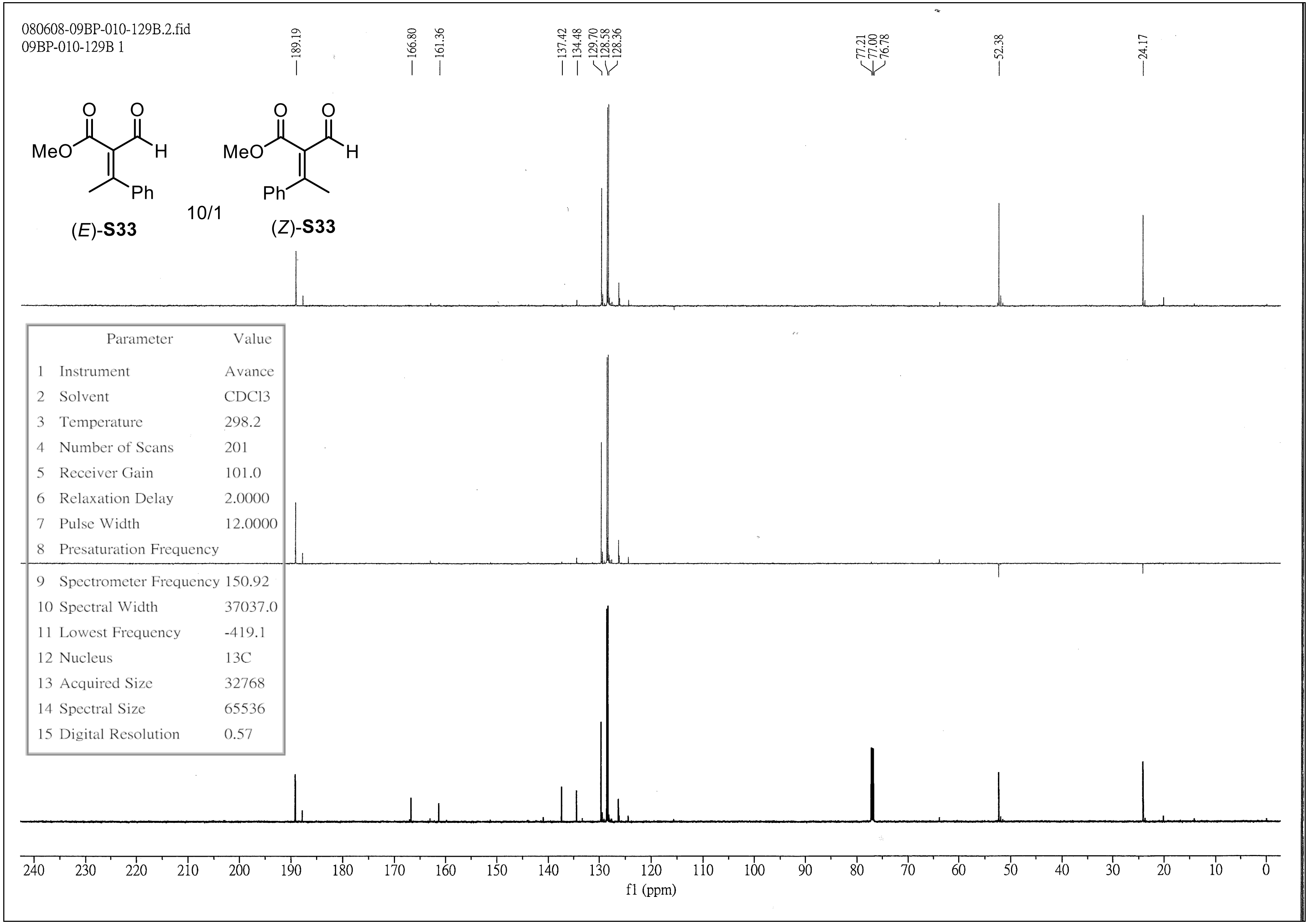


$^{13}$C NMR + DEPT spectra for compounds (*E*)-**S33** and (*Z*)-**S33**

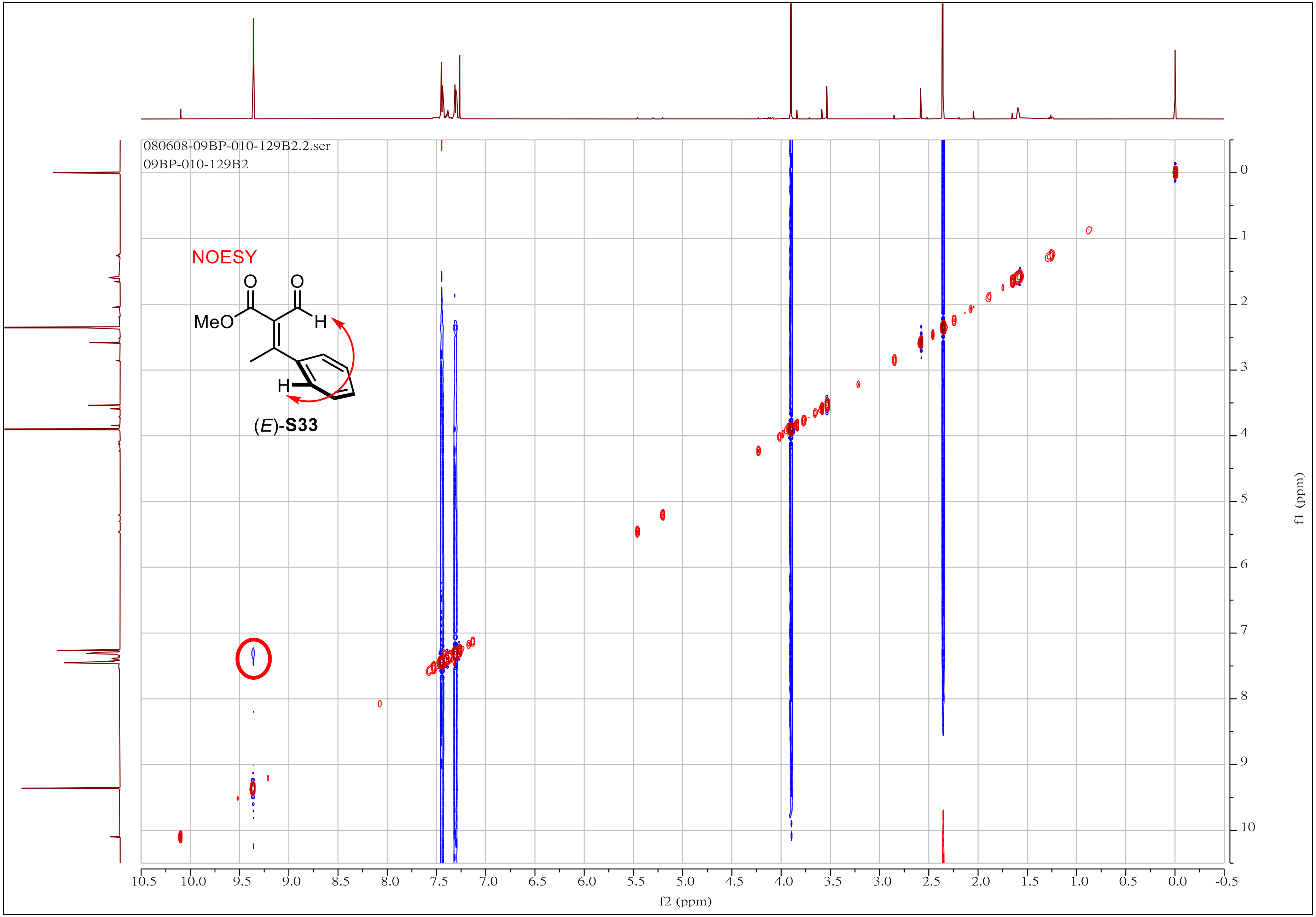


2D NOESY spectrum for compound **S33**

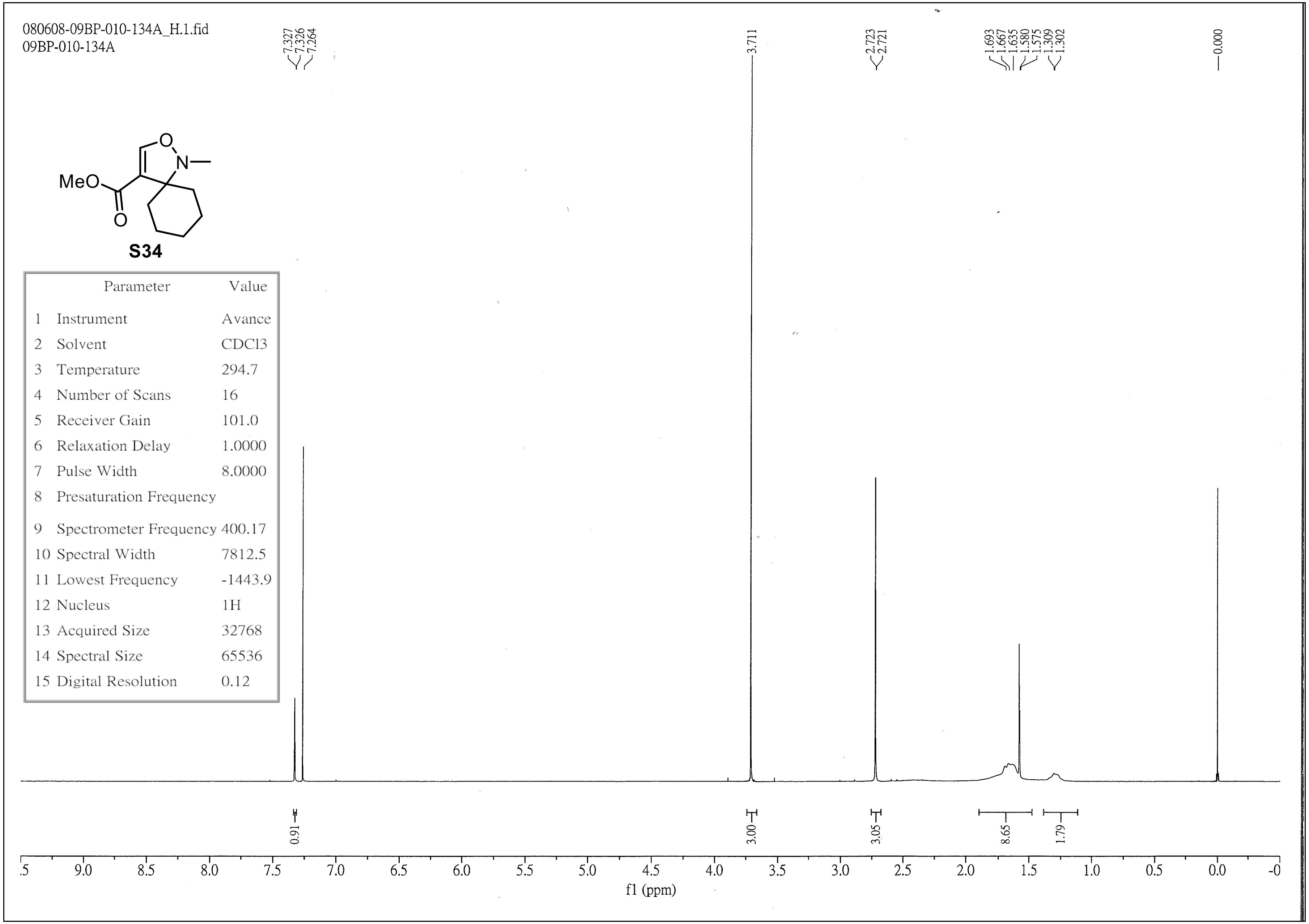


$^1$H NMR spectrum for compound **S34**

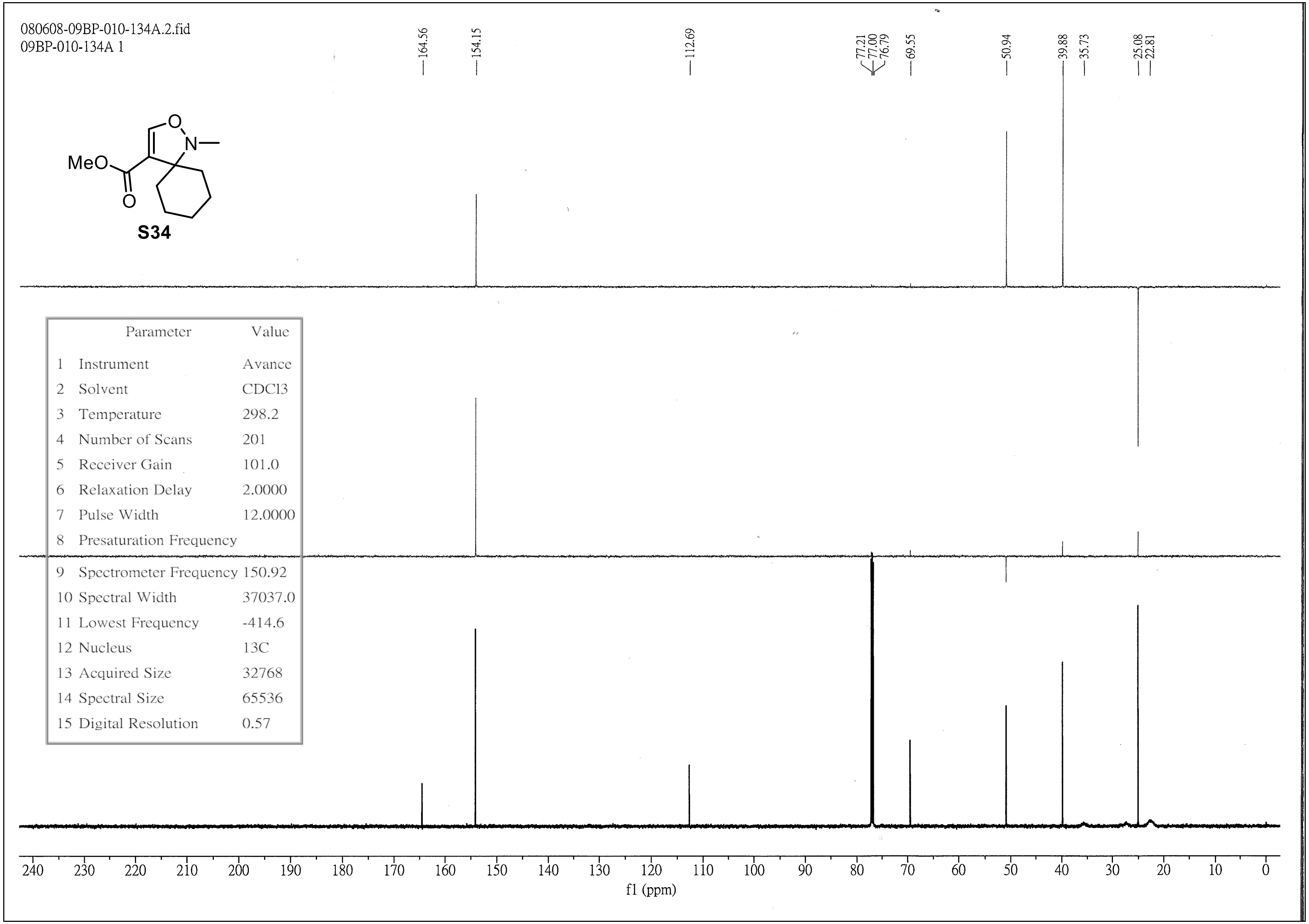


$^{13}$C NMR + DEPT spectra for compound **S34**

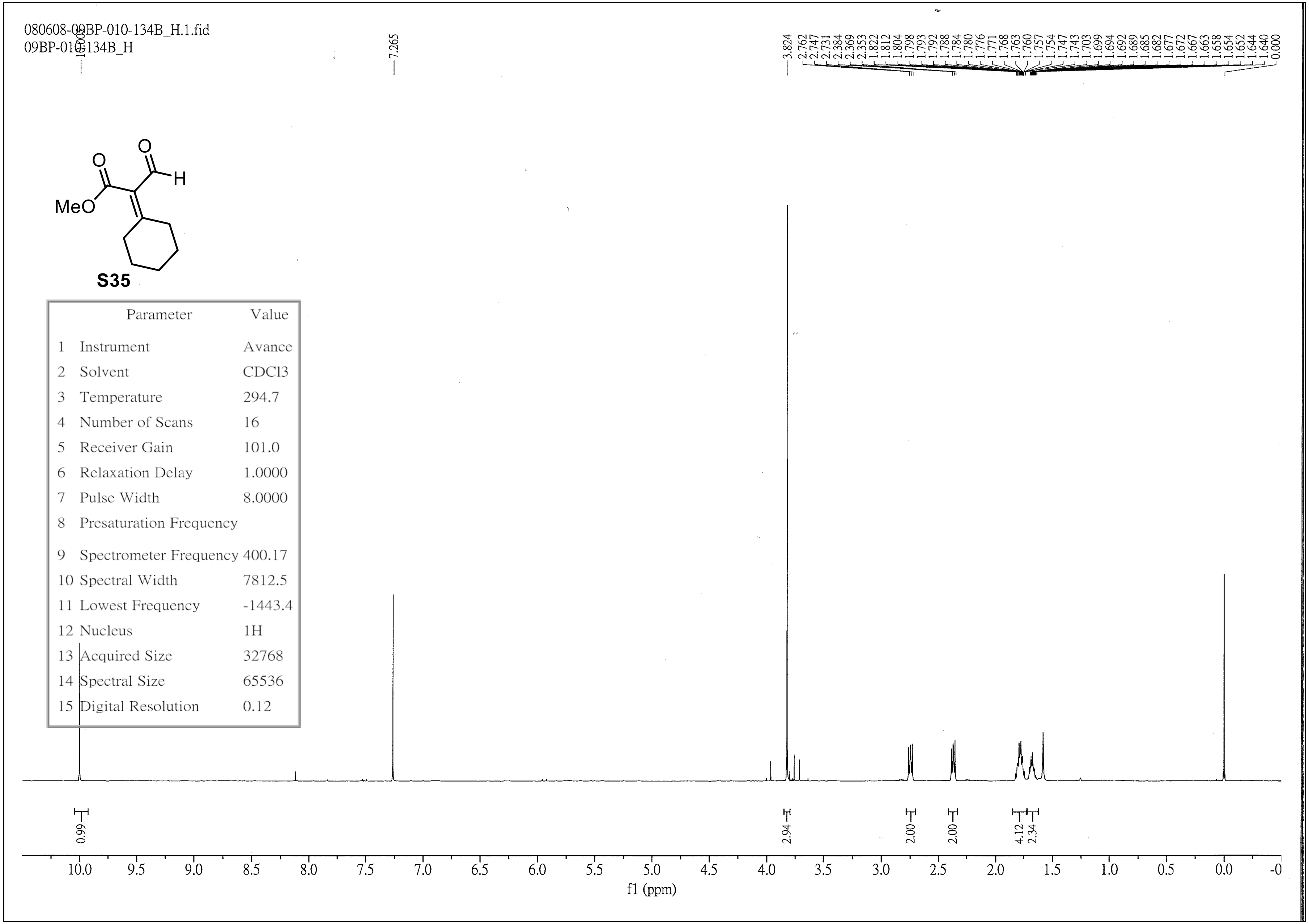


$^{1}$H NMR spectrum for compound **S35**

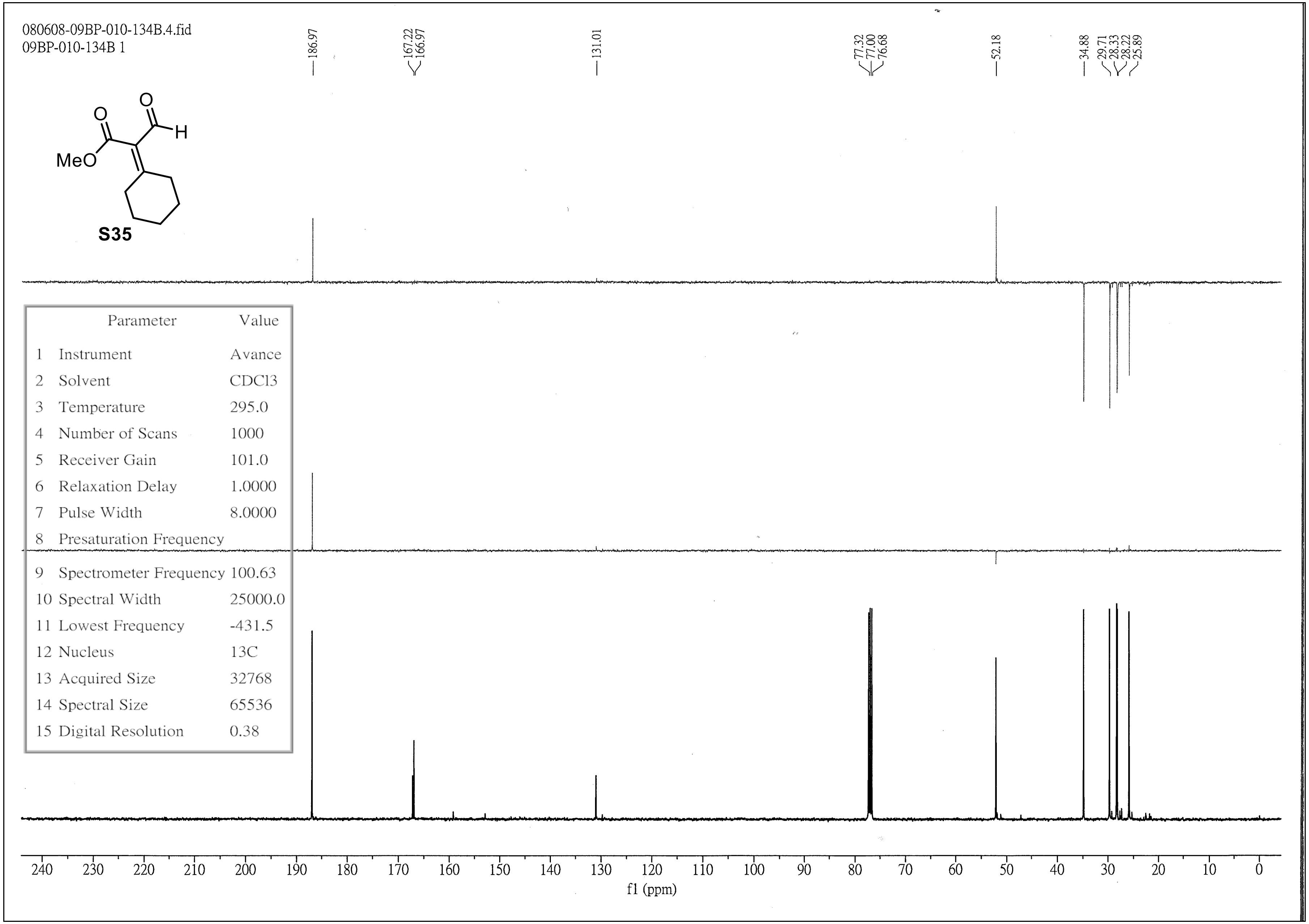


| | Parameter | Value |
|---|---|---|
| 1 | Instrument | Avance |
| 2 | Solvent | CDCl3 |
| 3 | Temperature | 295.0 |
| 4 | Number of Scans | 1000 |
| 5 | Receiver Gain | 101.0 |
| 6 | Relaxation Delay | 1.0000 |
| 7 | Pulse Width | 8.0000 |
| 8 | Presaturation Frequency | |
| 9 | Spectrometer Frequency | 100.63 |
| 10 | Spectral Width | 25000.0 |
| 11 | Lowest Frequency | -431.5 |
| 12 | Nucleus | 13C |
| 13 | Acquired Size | 32768 |
| 14 | Spectral Size | 65536 |
| 15 | Digital Resolution | 0.38 |

$^{13}$C NMR + DEPT spectra for compound **S35**
